# Generating Molecular Diversity via Addition of Nucleophiles to Electron-Deficient [3]Dendralenes: An Exploratory Study

**DOI:** 10.1021/acs.joc.5c02397

**Published:** 2026-01-14

**Authors:** Stefanie Magela Perdomo, Ondřej Kratochvíl, Rastislav Antal, Michal Kadaník, Petr Matouš, Jiří Kuneš, Aleš Růžička, Adam Kurčina, Lubomír Rulíšek, Erik Andris, Pavel Kočovský, Milan Pour

**Affiliations:** † Department of Organic and Bioorganic Chemistry, 69727 Charles University, Faculty of Pharmacy in Hradec Králové, Heyrovského 1203, 500 05 Hradec Králové, Czech Republic; ‡ Department of General and Inorganic Chemistry, 48252 University of Pardubice, Faculty of Chemical Technology, 532 10 Pardubice, Czech Republic; § Institute of Organic Chemistry and Biochemistry of the Czech Academy of Sciences, Flemingovo náměstí 542/2, 160 00 Prague 6, Czech Republic; ∥ Department of Organic Chemistry, Charles University, Faculty of Science, Hlavova 8, 128 43 Prague 2, Czech Republic

## Abstract

Electron-deficient dendralenes, bearing enone substructures and possessing an unfavorable disposition of like charges at the neighboring carbons, undergo nucleophilic 1,4-addition (Michael) or 1,6-addition (anti-Michael). Diverse products are obtained, including those of simple addition as well as cyclic and *ortho*-fused systems arising via multistep sequences, depending on the structure of the substrate and the nature of the nucleophile. Attack of a hydride at an enone fragment triggers the formation of multisubstituted pyranones and furans; furan formation was also initiated by thiolates. A notable exception is the derivative with a five-membered cyclic enone, which prefers simple additions followed by the reshuffling of the double bonds for both H^–^ and RS^–^ nucleophiles. By contrast, the latter enone is the only one that can react with stabilized C-nucleophiles, yielding bicyclic compounds. Domino cyclizations can also be induced by the enolization of the enone with DBU, giving mostly polysubstituted furans. However, the dendralene with a five-membered cyclic enone and its analogue with a six-membered ring behave differently: The former gives a mixture, while the latter prefers the formation of an isocoumarin derivative, which is driven by aromatization. DFT calculations have shown that the additions of thiolates are mostly governed by the thermodynamic stability of possible products arising from complex equilibrium processes.

## Graphical abstract

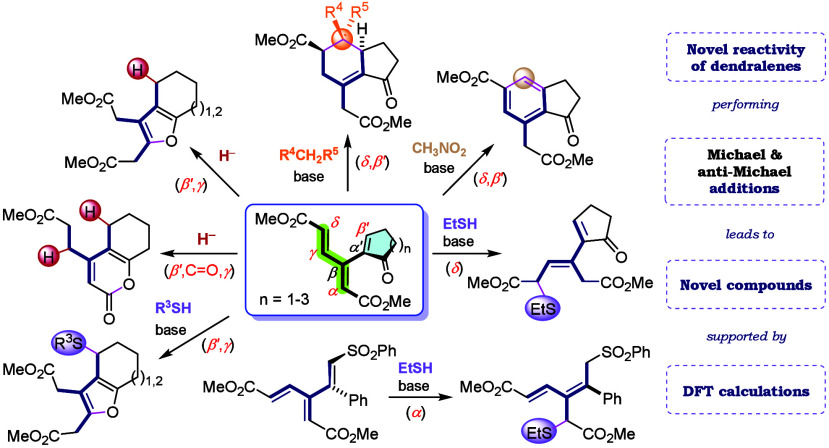

## Introduction

Multi-bond-forming reactions are among the most efficient tools enabling organic chemists to increase molecular complexity in a single operation. A unique opportunity to develop such processes is offered by conjugated olefins, of which the double bonds react in an interconnected manner. A classic example of such a process is the venerable Diels–Alder reaction. Consequently, conjugated sp^2^ hydrocarbons possessing multiple reaction sites have an extraordinary potential to undergo multi-bond-forming processes, enabling a rapid construction of complex structures. In this realm, dendralenes stand out as a distinct class of polyenes,^1,2^ since their cross-conjugated nature allows the Diels–Alder (DA) reaction to proceed as a series of consecutive transformations.^3^ Here, reorganization of the π-electrons in a DA process results in the formation of an intermediate diene that can undergo another DA reaction, a process known as diene-transmissive Diels–Alder reaction (DTDA).^3^ While DTDA, capable of constructing two or more cycles and numerous chiral centers, is apparently the most attractive transformation, other reactions are also conceivable, but very few have been examined to date. The first example,^4^ reported as early as in 1975, was cyclopropanation;^5^ a few other examples, such as higher cycloadditions,^6^ anionic polymerization,^7^ and selective hydroboration,^8^ appeared more recently. However, the potential of, e.g., photochemical processes and electrophilic, nucleophilic, radical, and transition-metal-promoted additions remains virtually unexplored. Hence, following up on our recently reported^9^ facile synthesis of highly polarized, electron-deficient dendralenes (see Figure 1 for a general structure) and their reactivity in DTDA processes,^9^ investigation of nucleophilic additions (A_N_) to these systems appears to be particularly attractive in view of their undisputable potential to unlock new multistep complex transformations.

**Figure 1:**
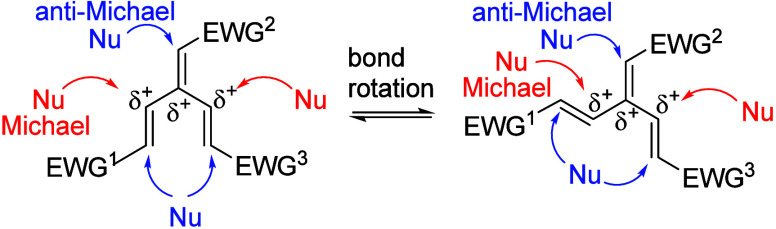
Conceivable nucleophilic additions to electron-deficient [3]­dendralenes (represented by two conformers allowing cross-conjugation). EWG stands for electron-withdrawing group.

These dendralenes, decorated at their termini with electron-withdrawing groups, possess an unfavorable disposition of like charges at the neighboring carbons^10^ (Figure 1). Therefore, we expected the reversal of this apparently thermodynamically inconvenient state to become the major driving force of their reactions with nucleophiles. Notably, the arrows in Figure 1 show that both Michael and anti-Michael (or vinylogous Michael) additions^11^ can initiate a change in the distribution of charges, with the participation of the remaining double bonds being more than conceivable. In addition, steric factors also need to be considered since a polysubstituted dendralene molecule can hardly be expected to assume a conformation that would position all three C=C bonds in the same plane and thus allow an overlap of all of the p orbitals of the π-systems. In this work, we report, for the first time, that electron-deficient dendralenes are capable of structure-dependent nucleophilic additions, giving rise to a variety of structurally diverse products. We also demonstrate that the reactivity can vary from simple additions to multistep domino cyclizations, which afford *ortho*-fused bicyclic products. We envisaged that these A_N_ sequences would parallel those reported for DTDA reactions and offer as yet undescribed opportunities for the synthesis of relevant natural products.

## Results and Discussion

For our investigation, we have selected a set of model compounds, most of which were recently prepared by us^9^ (Figure 2), namely, enones **1a**–**1d**, connected to the dienoate moiety via the α-carbon; conjugated lactone **1e**, also connected by the α-carbon; isomeric enones **1f** and **1g**, connected by the β-carbon; triester **1h**; and sulfone **1i**.

**Figure 2:**
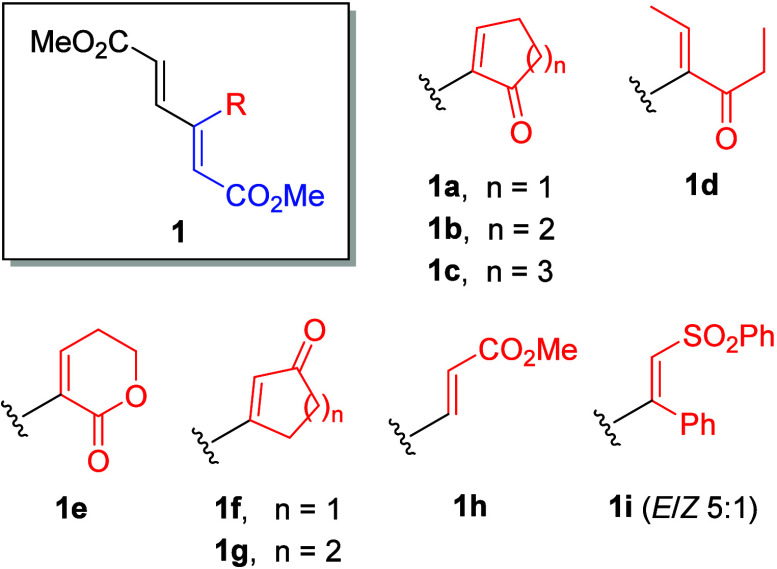
Model dendralenes **1a**–**1i** synthesized for this study.

Calculations have demonstrated that in the lowest-energy conformation of selected dendralenes **1a**, **1b**, and **1i** the dienoate moiety is invariably planar, whereas the bulky appendage R is positioned almost perpendicular to that plane. This is illustrated by the structure of **1b** in the top row of Figure 3. The same conformation is demonstrated for **1i** by single-crystal X-ray analysis (see the Supporting Information). Therefore, the C–C bond connecting the dienoate moiety with the appendage R can be regarded, in principle, as a chiral axis. However, according to calculations, the rotation barrier here is too low (e.g., 13 kcal mol^–1^ for **1b**) to allow isolation of the atropisomers (Note S1 of the Supporting Information).

**Figure 3:**
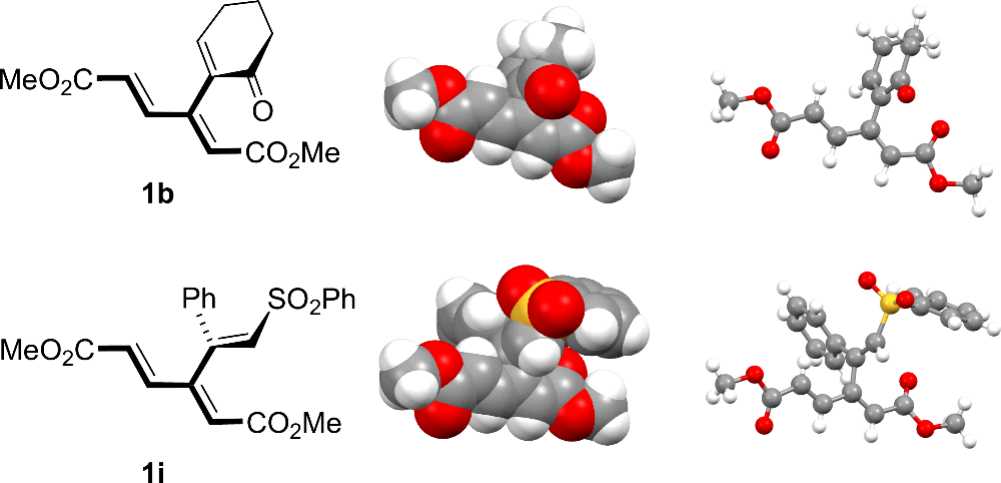
Spatial representation of the structures of dendralene **1b** (top row), obtained by calculations, and dendralene **1i** (bottom row), obtained by X-ray crystallography. In this and the following schemes, the bonds held in the plane as part of the conjugated π-system are portrayed as bold lines. Bonds out of that plane (approximately perpendicular) are highlighted with wedges.

### Hydride as a Nucleophile

The first nucleophile that we chose to investigate was a hydride. Initial attempts with sodium borohydride as its source revealed remarkable differences between keto-diester **1a** with the cyclopentenone appendage and the rest of the dendralenes. Thus, reduction of **1a** with NaBH_4_ in aqueous methanol proceeded via a conjugate addition of H^–^ at the β′-position of the cyclopentenone moiety, affording alcohol **5** in 71% yield (Scheme 1). The presence of water in the reaction mixture proved to be beneficial since in the strictly anhydrous methanol the product was obtained in a lower yield (65%). This reactivity can be rationalized by assuming an initial borohydride attack at the enone moiety (approximately perpendicular to the dienoate segment), generating enolate **2**, which can assume multiple conformations, such as **2a**, **2a′**, and **2a″**, by rotation about the single bonds, as shown. Of these, **2a″**, which would lead to enolate **3**, is apparently too congested, so that the pathway via **2a′** dominates, giving intermediate **4**, which undergoes protonation and reduction of the keto group (rather than any other conjugate addition), giving rise to alcohol **5** as the final product.^12^ It is pertinent to note that the alternative 6­(O)-*endo*-trig cyclization of enolate **2a** (highlighted by a blue double-crossed curved arrow) was not observed.

**Scheme 1:**
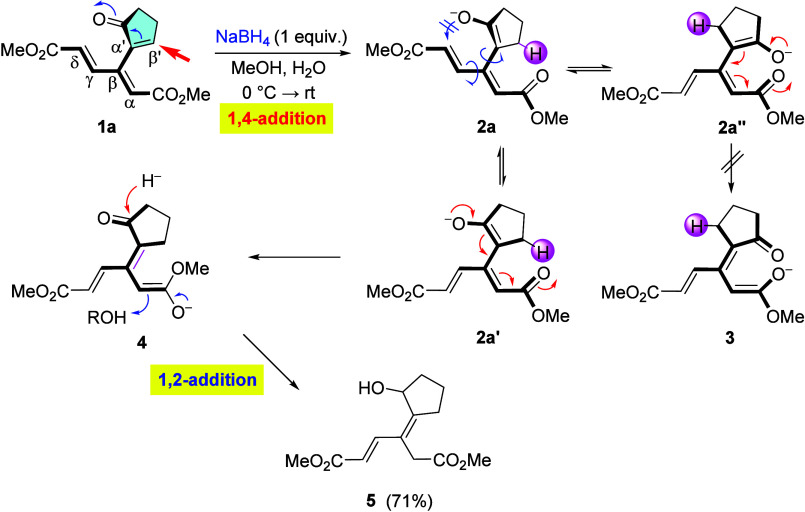
Reduction of Cyclopentenone **1a** ^a^

a In the 3D structure, the cyclopentenone ring is approximately perpendicular to the plane of the dienoate moiety.

By contrast, reduction of cyclohexenone derivative **1b** followed a different pathway (Scheme 2). Here, enolate **2b**, generated by conjugate hydride addition at the enone unit, was found to exhibit dichotomous behavior, affording two products, arising via 1,4- and 1,2-addition (at 0 °C within 60 min). Pathway *i* involves an intramolecular 5­(O)-*exo*-trig 1,4-addition (Michael) to produce, after aromatization of intermediate **6b**, furan derivative **7b** (41%). The competing pathway *ii* generated lactone **8b** as a result of the 6­(O)-*exo*-trig 1,2-attack at the ester group; the reaction was completed via a predominant 1,6-reduction (anti-Michael) of the external acrylate moiety to produce lactone **9b** (27%), as evidenced by isotopic labeling, using NaBD_4_ (see the Supporting Information for details). Under the same conditions, but over an extended reaction time (5 h), cycloheptenone analogue **1c** furnished *ortho*-fused cyclohepta­[*b*]­furan derivative **7c** in 50% yield; formation of lactone **8c**/**9c** was not observed in this instance.

**Scheme 2:**
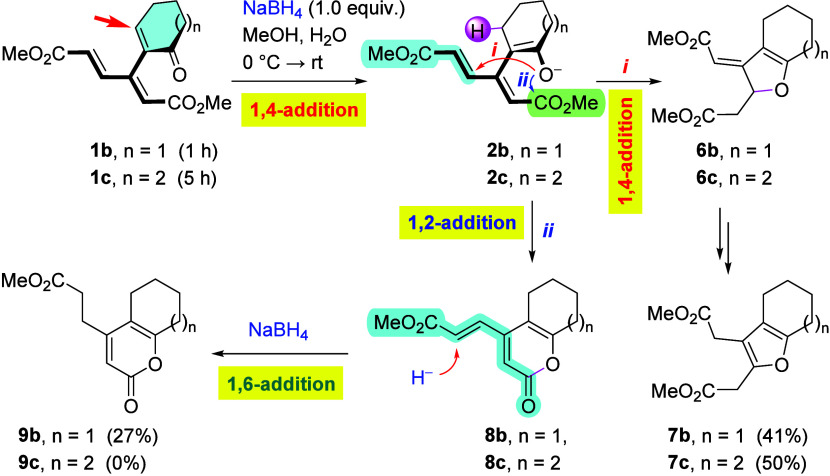
Competing Pathways in the Hydride Reduction of Enones **1b** and **1c**

Acyclic enone **1d** followed the pattern set by cyclohexenone and cycloheptenone analogues **1b** and **1c**, respectively, to some extent (Scheme 3). Pathway *i*, triggered by the 1,4-addition (Michael) of H^–^ to the enone fragment, was found to dominate, giving rise to furan derivative **7d** (41%). The competing pathway (*ii*) involved a direct C=O reduction, followed by the formation of lactone **8d** that was further reduced in the side chain to give **9d** (21%) as the second major product. In analogy with the reduction of **8b**, where the mechanism is evidenced by isotopic labeling, the latter reduction of **8d** can be assumed to proceed in the same way.^13^

**Scheme 3:**
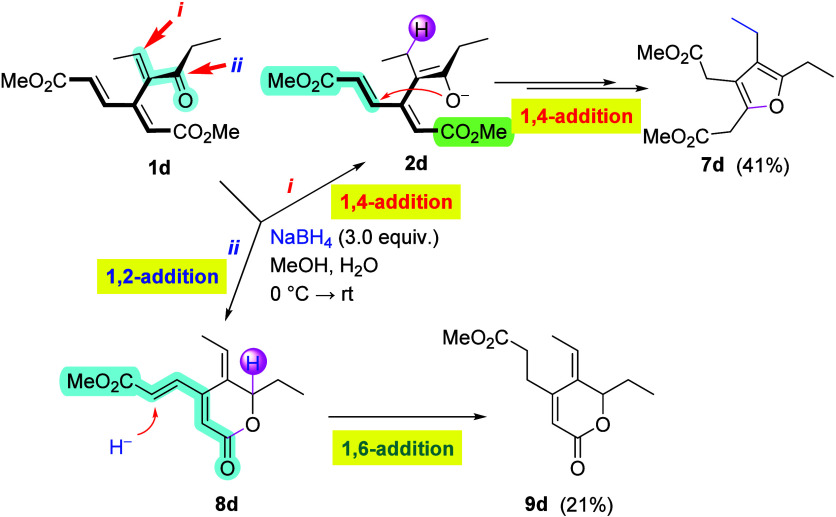
Reduction of Acyclic Enone **1d**

Under Luche conditions (NaBH_4_/CeCl_3_), cyclohexenone derivative **1b** was found to produce a mixture of dendralenic lactone **10** and diol **11** (Scheme 4). Lactone **10** (see the Supporting Information for X-ray analysis) apparently arose via reduction of the carbonyl in enone **1b**, followed by lactonization, the product of which became the substrate for further CeCl_3_-assisted reduction to generate the corresponding lactol that underwent another reduction to give diol **11**. The latter diol became the sole product obtained in high yield (94%), when an excess of borohydride was used.

**Scheme 4:**
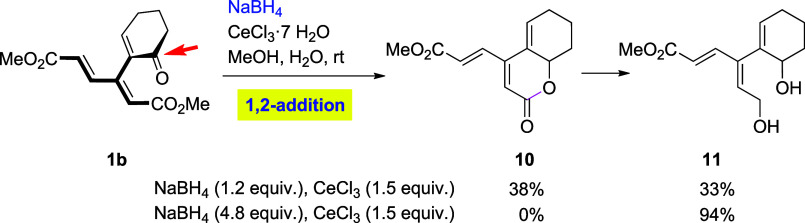
Reduction of **1b** in the Presence of CeCl_3_

Not surprisingly, reduction of β-substituted cyclic enones **1f** and **1g** (Scheme 5), isomeric to **1a** and **1b**, was found to proceed solely at the carbonyl bond since conjugate addition to the tertiary β-position is precluded by increased level of steric congestion. This reduction thus gave rise to alcohols **12f** and **12g** (35% and 56%, respectively). Unstable cyclopentenol derivative **12f** was isolated as the only product from a rather complex mixture. In these instances, the propensity for conjugate 1,4-reduction (Michael) is obviously diminished by the enhanced steric hindrance at the β′-carbon of the enone moiety and by the weak propensity of the dienoate moiety to undergo reduction (vide infra).

**Scheme 5:**
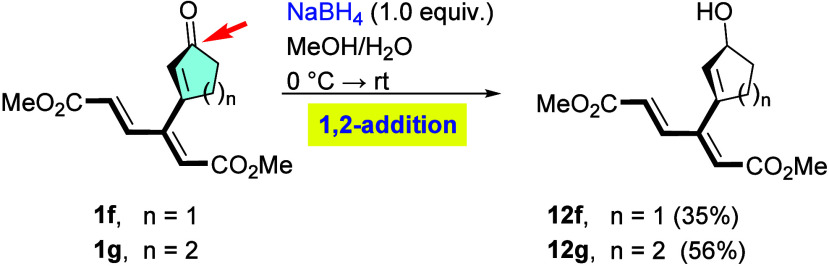
Reduction of β-Substituted Enones **1f** and **1g**

In conflict with general expectations that the acrylate moiety should readily undergo Michael addition, triester **1h** was found to be inert to NaBH_4_, as was lactone **1e**. Finally, attempted reduction of sulfonyl derivative **1i** afforded an intractable mixture of products under the same conditions.

Altogether, the results of hydride additions have shown that, like a dienophile in DTDA sequences, hydride attack can initiate multiple bond-forming processes, typically proceeding in a Michael or anti-Michael fashion.

### Thiolate Nucleophiles

Thiolates, recognized as strong nucleophiles, were the next reagent types to be explored. In contrast to hydride addition, treatment of cyclopentenone derivative **1a** with ethanethiolate (generated by deprotonation of ethanethiol with DBU) initially afforded an unstable major product, which was assigned structure **14** based on NMR spectroscopy (Scheme 6). The latter compound apparently arises via a 1,6-addition (anti-Michael), starting with an attack at the sterically less hindered terminus of the dienoate moiety followed by protonation of intermediate enolate **13**. Adduct **14** then underwent isomerization to thermodynamically more stable isomer **15** upon purification by chromatography. Interestingly, the enone moiety turned out to be inert, even in the presence of an excess (3 equiv) of the thiol.

**Scheme 6:**
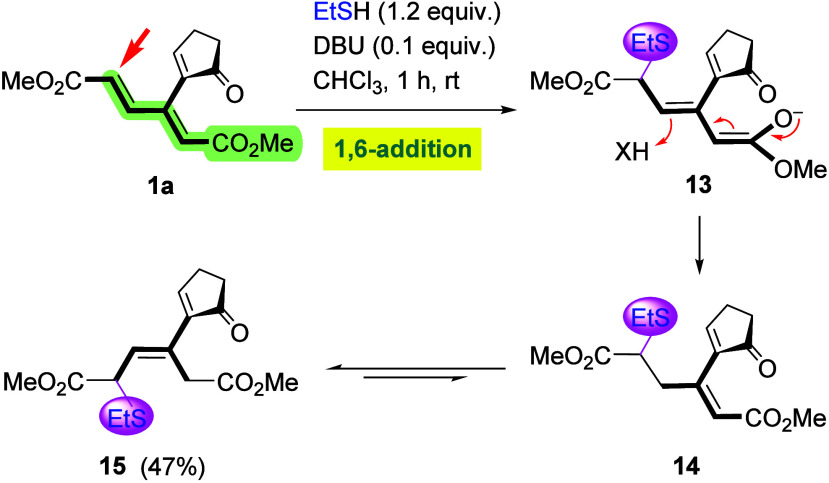
Anti-Michael Addition of Ethanethiolate to **1a**

On the other hand, as outlined in Scheme 7 and Table 1, enones **1b** and **1c** followed the same pathway as that for hydride addition (Scheme 2), with a preferential attack at the enone ring. Interestingly, while the combination of EtSH and DBU produced an inseparable mixture of furan **17b** and its not fully aromatized counterparts (Table 1, entry 1), addition of I_2_ (10 mol %) as a catalyst^14,15^ steered the reaction to completion, giving furan **17b** as the only isolable product in 60% yield (entry 2). Replacing EtSH with 2-mercaptoethanol resulted in an increased yield of final product **18b** to 66% (entry 3). Similarly, homologous cycloheptenone derivative **1c** gave *ortho*-fused furans **17c** and **18c** in 45% and 53% yields, respectively (entries 4 and 5).

**Scheme 7:**
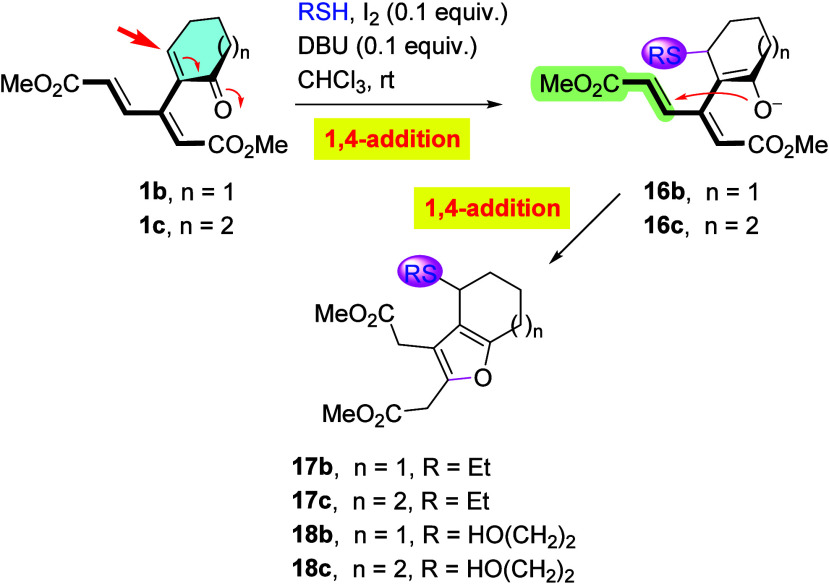
Addition of Thiolate to **1b** and **1c**

**Table 1:** Addition of Thiolates to Cyclic Dendralenes **1b** and **1c**

entry	starting material	time (h)	Nu (equiv)	product	yield (%)
1^a^	**1b**	1	EtSH (1.2)	**17b** ^b^	45
2	**1b**	1	EtSH (1.5)	**17b**	60
3	**1b**	5	HO(CH_2_)_2_SH (1.5)	**18b**	66
4	**1c**	5	EtSH (1.5)	**17c**	45
5	**1c**	8	HO(CH_2_)_2_SH (1.5)	**18c**	53

a No I_2_ added.

b Isolated as part of an inseparable mixture with not fully aromatized furans.

Since thiolate additions unveiled a further dramatic difference between five-membered enone **1a** and the rest of the cyclic dendralenes, it was of interest to explore the reactivity of acyclic dendralenes, namely, **1d** and **1i**. While **1d** was found to afford an inseparable mixture of products, sulfone **1i** reacted in an anti-Michael fashion (1,6-addition) at the more hindered α-site of the dienoate fragment to give adduct **20** (Scheme 8), which is in stark contrast to the reaction of **1a**, where the addition of the same nucleophile occurs at the opposite terminus (δ-position) of the dienoate moiety (Scheme 6). Whether this difference can be attributed to the stronger electron-withdrawing power of the phenylsulfonyl moiety is questionable since the vinyl sulfone group is bent away from planarity (as demonstrated by X-ray crystallography in Figure 3), which would prevent the involvement of its π-system. The reaction outcome can be rationalized by assuming a nucleophilic attack at the planar dienoate segment to generate enolate **19**, which would then afford **20** upon protonation. However, this mechanism would not explain the difference in the sites of the attack in **1i** and **1a**. An alternative scenario can thus be proposed, which would require planarization of the α,β,β′,α′-segment by rotation of the vinyl sulfone moiety together with an out-of-plane rotation of both the ester and phenyl group and also rotation of the acrylate moiety, as in 1i*, thereby minimizing conformational strain. Nucleophilic 1,6-attack at the now planarized segment (highlighted by color), followed by protonation, would then readily produce adduct **20**. A detailed description of this scenario is given in the computational section (*vide infra*).

**Scheme 8:**
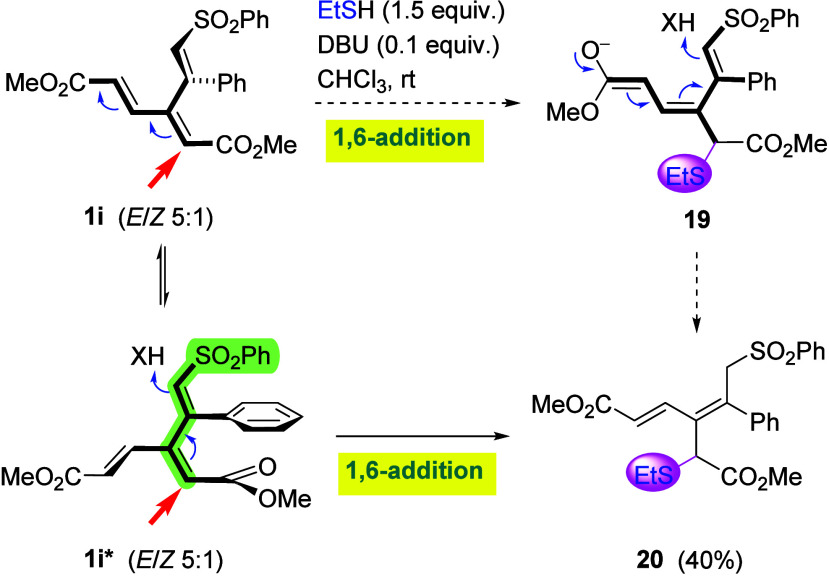
Addition of EtSH to Sulfone **1i**

### Carbon Nucleophiles

Rather surprisingly, derivatives **1a** and **1b** were found to be inert to freshly prepared Me_2_CuLi and several other organometallic combinations, known to favor conjugate additions. In all of these cases, the starting material was recovered, suggesting that the organometallics could not properly coordinate to the enone moiety due to steric hindrance and served merely as bases, effecting the formation of enolates, which were converted back to the starting enones upon workup. However, interesting results were obtained with malonate-type nucleophiles. This time, only cyclopentenone-derived dendralene **1a** was found to react, while its homologues, **1b** an**d 1c**, with larger rings and acyclic enone **1d** turned out to be inert again. Treatment of **1a** with deprotonated dimethyl malonate, as a representative stabilized carbon nucleophile, furnished bicyclic derivative **24x** (Scheme 9 and Table 2). In light of the previous experiments with EtS^–^ (Scheme 6), the latter reaction can be considered to start with the anti-Michael 1,6-addition to generate enolate (**21x**), followed by a second deprotonation of the malonate moiety and the 6­(C)-*exo*-trig Michael cyclization of resulting intermediate **22x** toward the enone moiety. Protonation of arising bicyclic intermediate **23x** would then afford **24x** as the final product. A reversed scenario, i.e., one commencing with a Michael addition to the cyclopentenone moiety, followed by a 6­(C)-*endo*-trig cyclization to the dienoate segment seems less likely in view of the reaction of **1a** with EtS^–^, which occurs at the dienoate unit to give **15**. Optimization of the base (Table 2, entries 1–4) identified the mixture of KO*t*Bu and NaOMe as the optimum. Hence, while the enone moiety was inert to thiolates, the intramolecular nature of the latter reaction rendered the attack possible (as a result of the second deprotonation of the malonate moiety). A brief extension to other activated nucleophiles, namely, to CH_2_(CN)_2_ and CH_2_(CN)­(SO_2_Ph), was also successful, affording bicyclic derivatives **24y** and **24z**, respectively (entries 5 and 6). The structure of bicyclic products **24** was corroborated by X-ray crystallography of **24x** (see the Supporting Information), while NOESY measurement showed that the signal of the phenyl ring of the PhSO_2_ moiety in **24z** correlates to that of the bridgehead hydrogen, demonstrating their *cis* relationship.

**Scheme 9:**
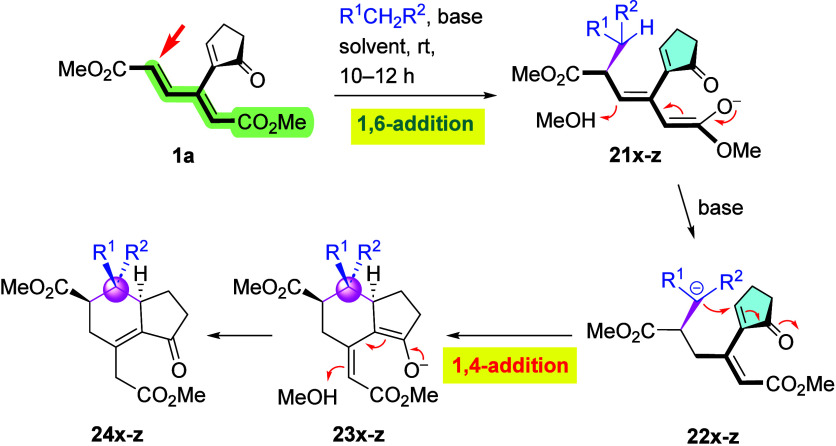
Addition of Stabilized C-Nucleophiles to Enone **1a** ^a^

a For R^1^ and R^2^, see Table 2.

**Table 2:** Reaction of **1a** with Stabilized C-Nucleophiles

entry	R^1^	R^2^	Nu (equiv)	base (equiv)	solvent	product	yield (%)
1	CO_2_Me	CO_2_Me	1	NaOMe (0.4)	MeOH	**24x**	14
2	CO_2_Me	CO_2_Me	3	NaOMe (1.0)	4:1 CHCl_3_/MeOH	**24x**	42
3	CO_2_Me	CO_2_Me	3	NaH (1.0)	CHCl_3_	**24x**	25
4	CO_2_Me	CO_2_Me	3	KO*t*Bu (0.5), NaOMe (0.5)	4:1 DCM/MeOH	**24x**	50
5	CN	CN	3	KO*t*Bu (0.5), NaOMe (0.5)	4:1 DCM/MeOH	**24y**	60
6	CN	SO_2_Ph	3	NaOMe (1.0)	4:1 CHCl_3_/MeOH	**24z**	50

Unlike its analogues with larger rings, cyclopentenone **1a** was also found to react with nitromethane in the presence of 2 equiv of NaOH (Scheme 10). In this case, the only product, isolated in a moderate yield of 42%, was diester **28** with a fully aromatized six-membered ring lacking the nitro group. Here, the reaction can be assumed to commence with a 1,6-addition (anti-Michael) at the δ-site. Resulting primary adduct **25** would then undergo second deprotonation, generating **26**, followed by an intramolecular 1,4-addition (Michael) to form bicyclic nitro derivative **27**, from which indenone **28** is obtained via a base-catalyzed nitrous acid elimination^16,17^ with ensuing air oxidation.

**Scheme 10:**
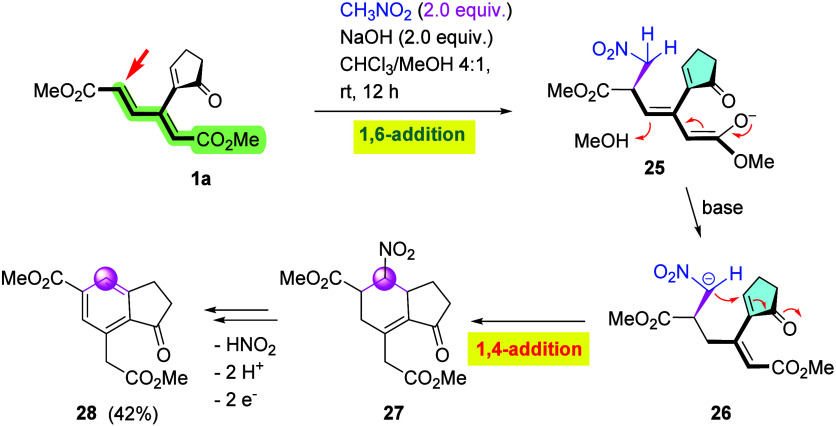
Cyclization of **1a** with CH_3_NO_2_

### Enolization

Finally, since many of these reactions encompass cyclization of enolate intermediates arising by an initial nucleophile attack, we attempted to generate enolates via simple deprotonation at the γ′-position of the enone segment. To this end, enones **1a–1d** were treated with DBU at room temperature. As summarized in Scheme 11, the three cyclic derivatives (**1a–1c**) were found to differ in behavior from each other, depending on the ring size. Thus, while five-membered cyclic enone **1a** afforded a complex mixture of products under these conditions, the initial steps in the case of its six- and seven-membered homologues (**1b** and **1c**, respectively) mirrored those observed for their reduction with NaBH_4_ and thiolate addition (as in Schemes 2, 3, and 7). Cyclohexenone derivative **1b** preferred lactonization via a 1,2-addition (as in pathway *ii* of Scheme 2), followed by an extensive reshuffling of the double bonds (via protonation/deprotonation) under the basic conditions, furnishing coumarin **31** in a high yield (87%) at room temperature in less than 1 h. Since no furan derivative (vide infra) was detected, it can be assumed that aromatization of the six-membered ring was the decisive driving force of the sequence. The reaction also proceeded when the base was used as a catalyst (0.1 equiv) but more slowly (over a period of 12 h) to give **31** in the same yield (87%) under an Ar atmosphere. In principle, enolization of **1b** could also occur at the α″-position, but this scenario is less likely in view of the behavior of the other members of this group, namely, **1c** and **1d**, where the product structure would be incompatible with that pathway (vide infra).

**Scheme 11:**
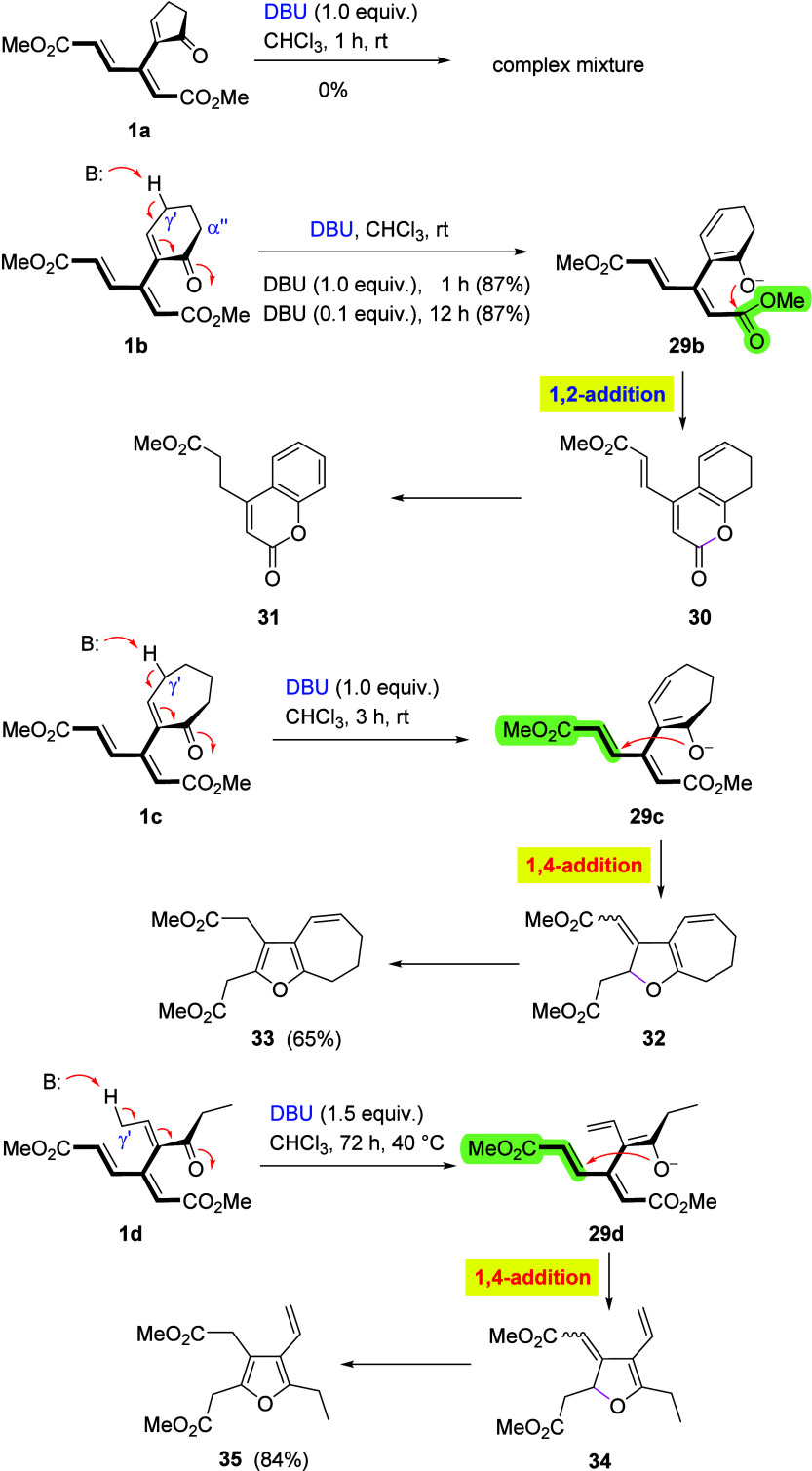
Cyclization of Dendralenic Enones **1a–1d** via Enolate Formation

Seven-membered cyclic enone **1c**, not capable of the formation of a benzene ring, afforded 7,8-dihydro-6*H*-cyclohepta­[*b*]­furan derivative **33** (65%), presumably via γ′-enolization, 5­(O)-*exo*-trig Michael cyclization, and subsequent aromatization, generating the furan ring (as in pathway *i* of Scheme 2). Acyclic dendralene **1d** behaved in the same way, giving rise to tetrasubstituted furan derivative **35**. In all of these instances, γ′-enolization is assumed, since the alternative α″-enolization would not lead to the formation of the products obtained.

### Mechanistic Considerations

For the computational investigation of the nucleophilic reactivity of dendralenes, we chose to focus on the thiol addition reaction as it showed the most variable experimental behavior, with attacks at different positions of individual substrates. The hydride addition occurred invariably at the β′-position (Figure 4), which renders this reaction mechanistically less revealing.

**Figure 4:**
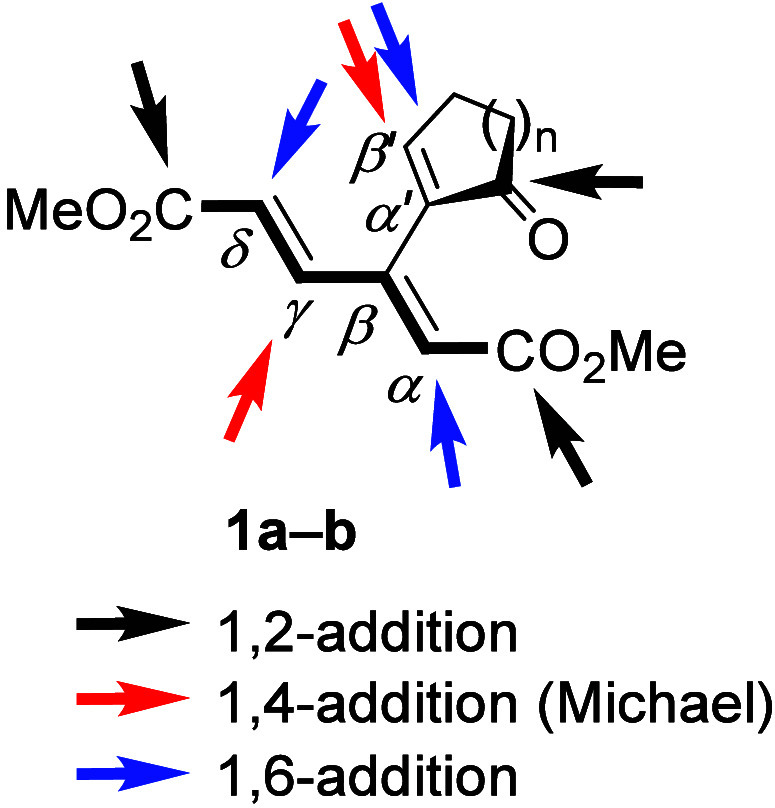
Sites for potential nucleophilic attack at dendralenes **1a** and **1b**.

Since the seminal work of Thomas and Kollman,^18^ the addition of thiolates to conjugated carbon systems has been studied by theoretical methods often in the context of biological chemistry^19^ (e.g., covalent kinase inhibitors) or materials chemistry.^20^ The most common approach relied on density functional theory (DFT) methods. Many computational contributions to the field have been summarized by Roseli et al. in a recent review.^21^ The choice of DFT functional has been a matter of debate, and it has been claimed that some popular functionals cannot predict stable minima for the gas phase addition of methanethiol to methyl vinyl ketone.^19b,22^ However, it has also been noted that moving from vacuum to implicit solvent often cures these problems.^21^

Since dendralenes can be regarded as cross-conjugated systems, it is of note that addition of a thiol to cross-conjugated enones has often been found to be close to thermoneutral compared to non-cross-conjugated variants.^23^

Nucleophilic attacks on dendralenes themselves, in general (not just thiols), have previously received a limited amount of attention. An important exception is the study of Takagi et al.,^24^ which was focused on polymerization of phenyl-substituted [3]­dendralenes. These were generally most reactive at the central methylene group (C4 in their paper; corresponding to the α-position in compounds studied here). It is noteworthy that the reactivity at this position could not be attributed to electronic effects (LUMO orbital location). The α-position was also not particularly reactive in the dendralenes studied in this work, showing the effect of substitutions with electron-withdrawing carbonyl groups.

Our model dendralenes possess nine sp^2^ carbons, as shown for cyclo-enones **1a** and **1b** (Figure 4), all of which can, *a priori*, undergo a nucleophilic attack, except perhaps β- and α′-positions that are sterically hindered. Thus, one can anticipate three types of attacks: 1,2-additions (black arrows), 1,4-additions (red arrows), and 1,6-additions (blue arrows). To investigate the differences in reactivity among the seven likely attack sites, we chose to computationally model the reactions of **1a**, **1b**, and **1i** with thiolate, as this reaction exhibited the most variability. While the addition of thiolate to **1a** proceeds at the δ-position (Scheme 6), the product observed in the reaction of **1b** requires thiolate addition at the β′-position (Scheme 7). We did not expect such variation of reactivity to occur upon the extension of the ring by one methylene group. Yet another variation was observed for **1i**, which was found to prefer an α-position attack, possibly reflecting the strong electron-withdrawing effect of the sulfone group.

Based on the inherent molecular properties of **1a** and **1b** (see the discussion of the Fukui function in the Supporting Information), we would not have expected any preferential site for the thiol addition. Therefore, to explain the experimental products, we performed extensive DFT calculations of the complete reaction pathways. Herein, we discuss only the relevant stable species, calculated at the ωB97M-V^25^/def2-TZVPD^26^//B3LYP^27^-D3/def2-TZVP level of theory. We chose the high-level ωB97M-V functional by comparing the predicted reaction energies to the DLPNO-CCSD­(T) calculation on selected systems. Despite the inability of the B3LYP functional to correctly identify local enolate minima in the gas phase,^19b,22^ we did not observe such behavior in the present study. For the full reaction scheme and free energies calculated at various levels of theory, see Figure S8.

Our DFT study was carried out for the reaction with MeSH instead of EtSH, as it reduced the computational cost. Calculations suggest that thiolate attacks at all relevant positions (α, δ, and β′) have no significant barriers. The only barriers are entropic, which are compensated by favorable interaction enthalpies, resulting in an effective barrier (see Figure S8) of ∼7–8 kcal mol^–1^, which is not affecting the reaction outcomes in any meaningful way. Therefore, the preference for the product is dictated by the overall free reaction energies (i.e., thermodynamically controlled). The exclusive isolation of product **15** from the addition of EtSH to **1a** (Scheme 6) is noteworthy, given the many electrophilic sites of **1a**. By comparing the free energies of anionic thiolate addition intermediates ^**1a**^ **INT1**, ^**1a**^ **INT2**, and ^**1a**^ **INT3** (Scheme 12), we observed an energetic preference for ^**1a**^ **INT2**, corresponding to attack at the β′-site. However, despite the relative stability of the latter intermediate, the reaction takes a different turn, namely, the formation of less stable ^**6**^ **INT1** (arising by δ-attack), whose protonation yields ^**1a**^ **P1** as the most stable product (corresponding to **15** in the experiment). However, the calculated free energy differences among products ^**1a**^ **P1**–**^1a^P3** are very small, falling within the typical error range of DFT calculations (2–3 kcal mol^–1^), and the reactions are close to being thermoneutral (ca. −4 kcal mol^–1^). Furthermore, the most stable product varied across different DFT methods, making the results uncertain. In conflict with the experimental findings, our calculations suggest that double addition product ^**1a**^ **P5** should be even more stable than single addition product ^**1a**^ **P1**, but the energy difference between them (2 kcal mol^–1^) is also rather small.

**Scheme 12:**
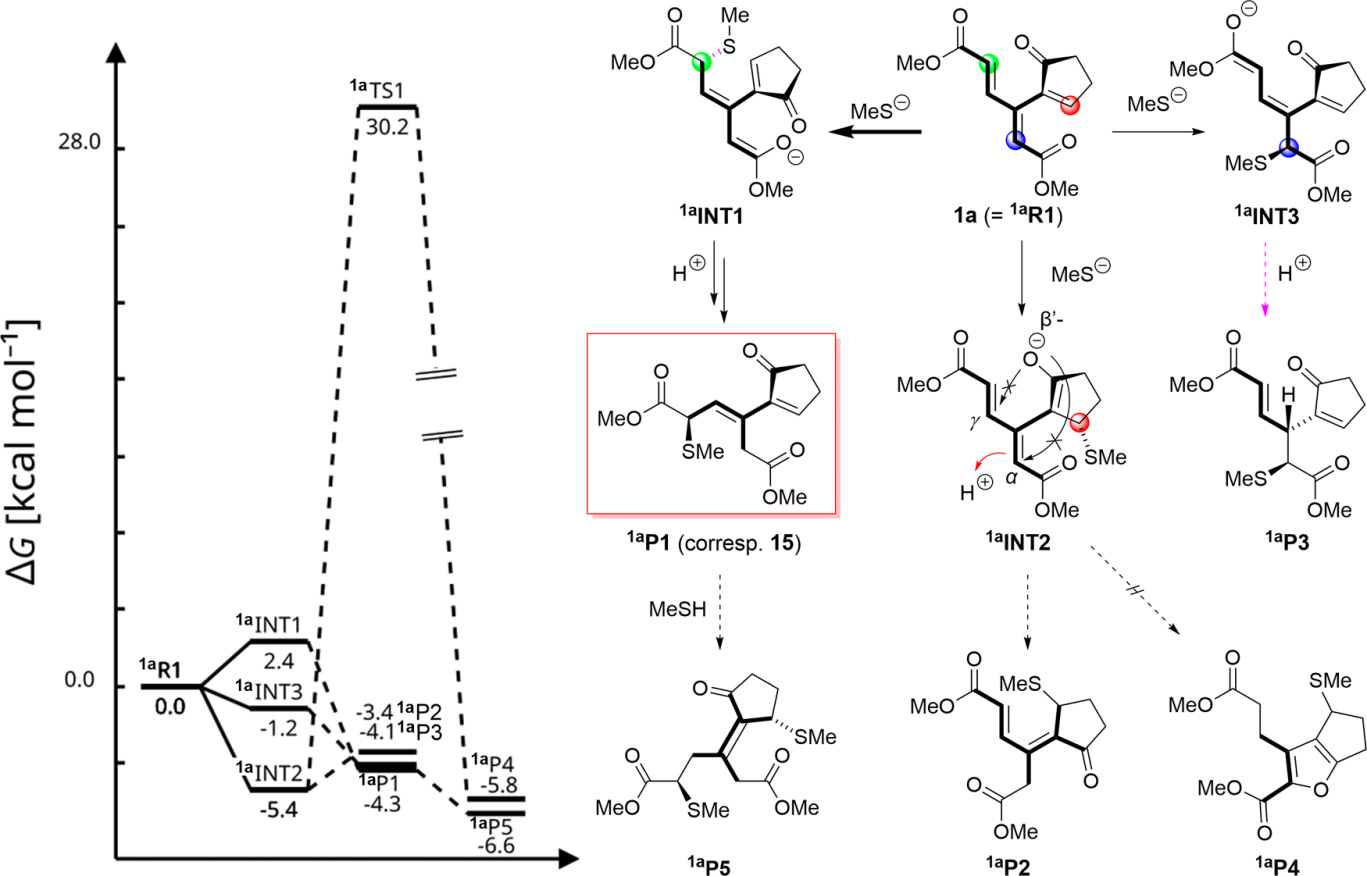
Illustration of Plausible Pathways for the Reaction of Dendralene **1a** (^**1a**^ **R1** in this scheme) with Methanethiolate^a^

a The Gibbs free energies under the standard conditions (298.15 K and 1 M) were calculated at the ωB97M-V/def2-TZVPD//B3LYP-D3BJ/def2-TZVP level of theory. The experimentally observed pathway is highlighted by a bold arrow.

Since **1b**, a homologue of **1a**, undergoes preferential β′-attack, followed by cyclization (Scheme 7), we attempted to find a rationale for the δ-attack in the case of **1a**. Here, the β′-attack would generate enolate ^**1a**^ **INT2**, whose 5­(O)-*endo*-trig cyclization toward the α-carbon is precluded by a high energy barrier (>35 kcal mol^–1^), in consonance with the Baldwin rules. Furthermore, the alternative 5­(O)-*exo*-trig cyclization toward the γ-carbon would yield unstable products, which cannot be optimized by DFT. The fact that the structure reverts back to reactants is presumably due to the strain in the [3.3.0] system that the latter ring formation would generate. Hence, cyclization is not preferred in either of the instances. Interestingly, after a potential aromatization by formation of the furan ring, product ^**1a**^ **P4** would be of practically the same energy as the product of the simple addition, ^**1a**^ **P1** (Scheme 12). This finding indicates that it is mainly the strain in the [3.3.0] potential intermediate that disfavors this pathway. Thus, due to the lack of stabilization by subsequent cyclization, and with a view of the principal reversibility of each step in all possible conjugate additions, it is understandable that the reaction of **1a** takes a path different from that of **1b**. This scenario thus results in a simple δ-attack, followed by protonation, which affords **15** (^**1a**^ **P1**) as a major isolable product.

By contrast, addition of thiolate to the cyclohexenone (**1b**) and cycloheptenone homologues (**1c**) occurs at the enone moiety. Subsequent Michael-type cyclization, analogous to pathway *i* in Scheme 2, then gives nonchiral furan derivatives **17b** and **17c**, respectively. In analogy to dendralene **1a**, a computational investigation of the addition of thiolate to dendralene **1b** (^**1b**^ **R1**;Scheme 13) indicates a thermodynamically controlled thiolate attack at the selected electrophilic sites. Furthermore, the reaction free energies for the formation of protonated products ^**1b**^ **P1**, ^**1b**^ **P2**, and ^**1b**^ **P5** were found to be consistent with those calculated for dendralene **1a** (ca. −4 kcal mol^–1^). However, in contrast to the inability of **1a** (specifically ^**1a**^ **INT2**) to cyclize (Scheme 12), the nucleophilic 5­(O)-*exo*-trig cyclization of enolate ^**1b**^ **INT2** toward the γ-carbon becomes plausible. This process was found to proceed through intermediate ^**1b**^ **INT4**, which is 4 kcal mol^–1^ uphill in energy (Figure S8), to ultimately reach cyclization product ^**1b**^ **P4**, which is consistent with the experiment (**17b** in Scheme 7). While the energy of alternative cyclization product ^**1b**^ **P3** is predicted to be similar to that of ^**1b**^ **P4**, the reaction barrier of the required 5­(O)-*endo*-trig enolate cyclization toward the α-carbon is prohibitively high (28 kcal mol^–1^), which is consistent with the Baldwin rules. Notably, the reaction free energy of the cyclization product from dendralene **1b** (−18 kcal mol^–1^) is significantly lower than that of any addition product (^**1b**^ **P1** or ^**1b**^ **P2**; Scheme 13) by −14 kcal mol^–1^, making product ^**1b**^ **P4** (and its experimental counterpart **17b**) a thermodynamic sink.

**Scheme 13:**
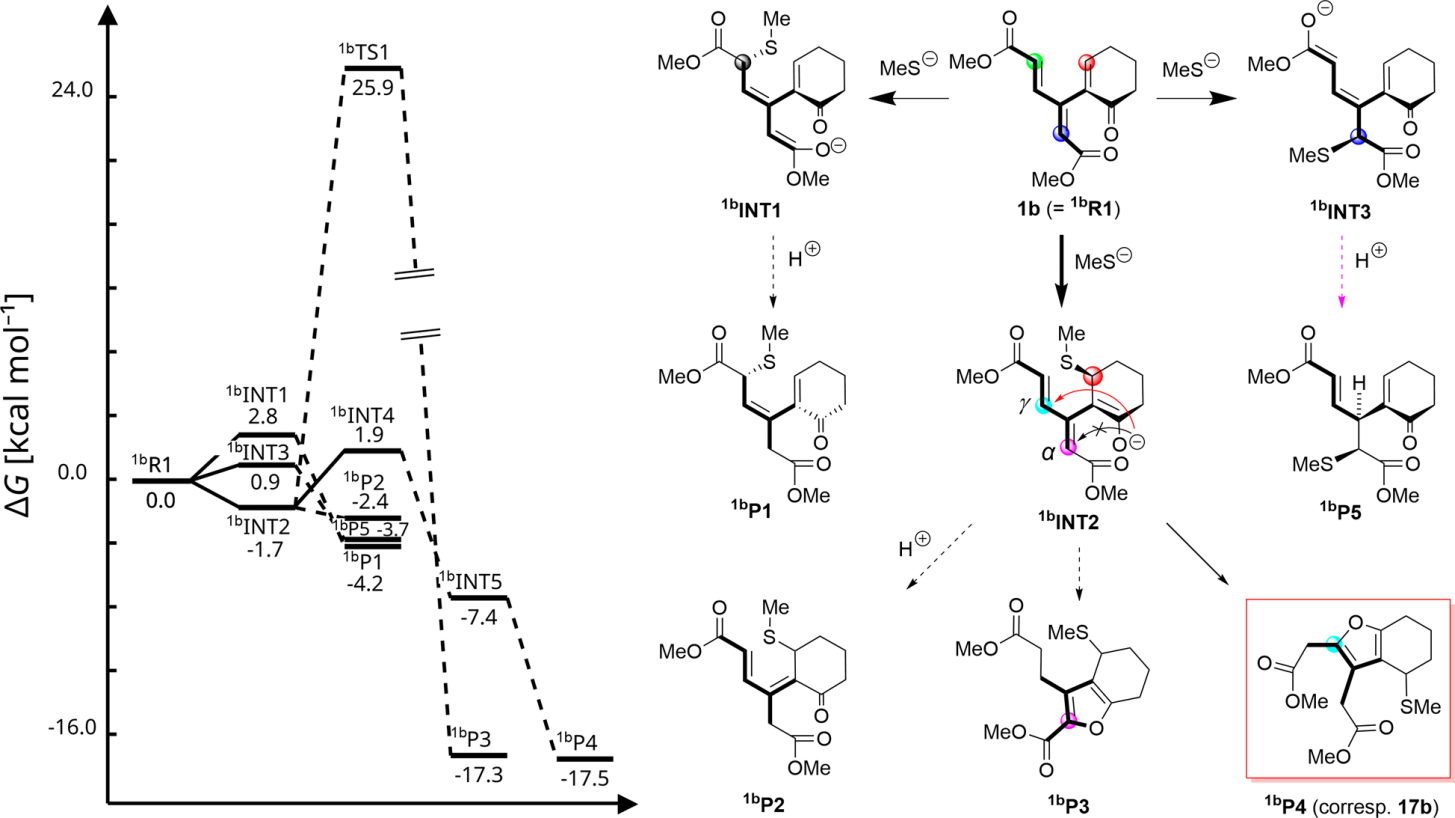
Illustration of Plausible Pathways for the Reaction of Dendralene **1b** (^**1b**^ **R1** in this scheme) with Methanethiolate^a^

a The Gibbs free energies under the standard conditions (298.15 K and 1 M) were calculated at the ωB97M-V/def2-TZVPD//B3LYP-D3BJ/def2-TZVP level of theory. The experimentally observed pathway is highlighted by a bold arrow.

To qualitatively explain the energy difference between the α- and preferred γ-cyclization pathways (Scheme 13), we employed the intrinsic bond orbital (IBO) approach. The IBO approach transforms the DFT wave function into a set of orthogonal orbitals localized at a minimal number of atoms,^28^ which has been shown to provide intuitive interpretation of chemical bonding. In our analysis, we examined the three IBO orbitals, which undergo the most changes during the enolate cyclization (Scheme 14). Consistent with the Baldwin rules, the γ-attack was found to exhibit a favorable orbital overlap, due to the nearly perpendicular orientation of the interacting orbitals, which is more favorable than that for the α-attack, which would require partial breaking of the C_α_=C_β_ bond.

**Scheme 14:**
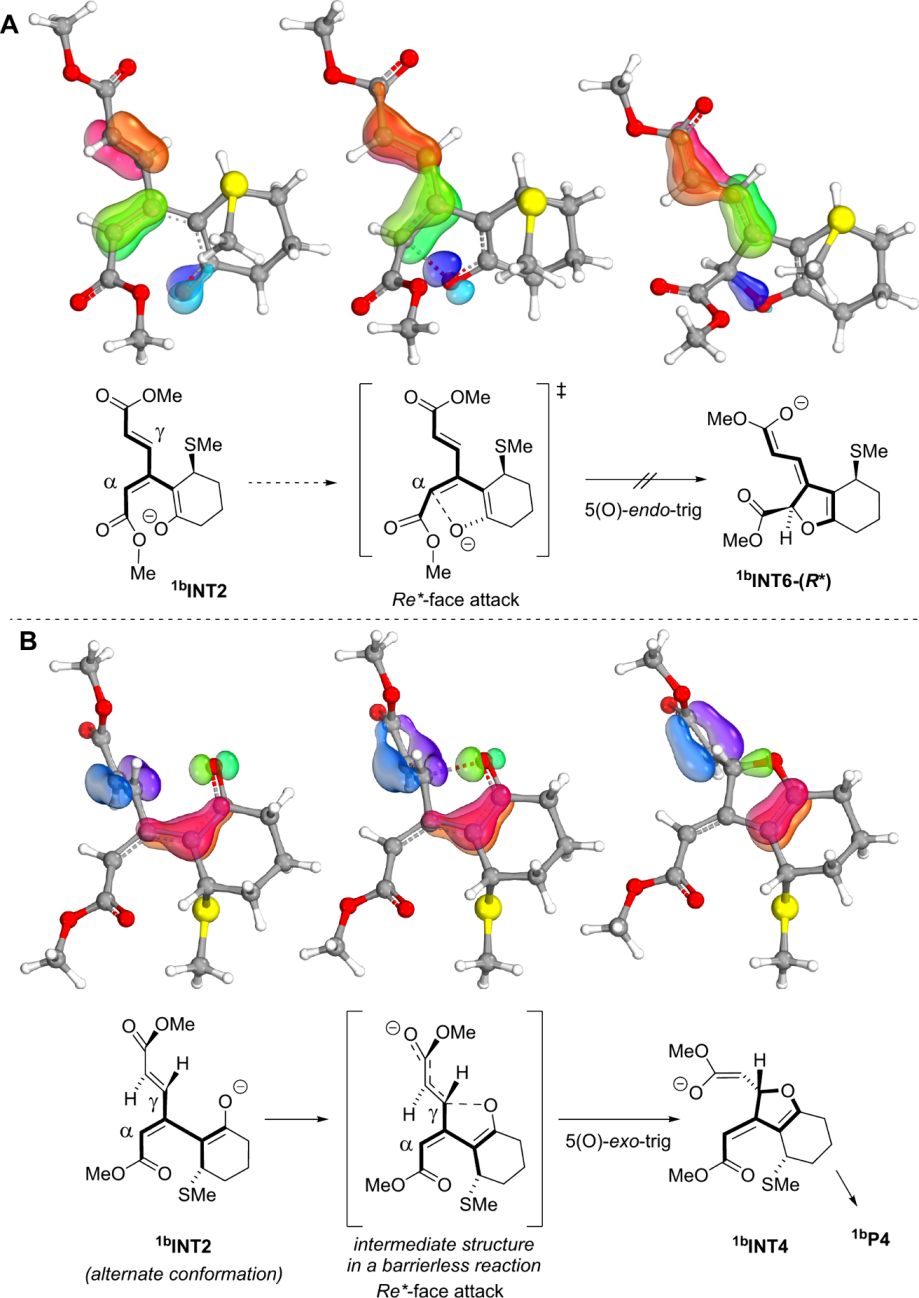
Relevant Occupied Intrinsic Bond Orbitals (IBOs, exponent 2) Depicting Orbital Changes during the Enolate Attack at the Less Favorable *α*-Position (**A**) and the More Favorable *γ*-Position (**B**) of the Dienoate Unit

Finally, we turned to sulfone **1i** and investigated its reaction with thiolate (Scheme 15) in a manner similar to what we used for enones **1a** and **1b**. The most energetically favorable anionic intermediates were predicted to correspond to an attack at the dienoate α-carbon. Depicted intermediate ^**1i**^ **INT1** (Figure 5B) represents the nucleophilic attack at the most stable conformer of **1i** (Figure 5A), whereas ^**1i**^ **INT4** (in fact lower in energy and more closely resembling product ^**1i**^ **P1**) would represent nucleophilic attack at hypothetical conformer 1i*, which was predicted to be unstable (at the B3LYP/TZVP level of theory). These intermediates are lower in energy than ^**1i**^ **INT2** and ^**1i**^ **INT3**, which stands in sharp contrast with dendralenes **1a** and **1b**, where the most stable anionic intermediate corresponds to thiolate attack at the β′- and δ-sites, respectively (Schemes 12 and 13). This α-preference could be attributed to the electron-withdrawing effect of the phenyl sulfone group being stronger than that of the enone and acrylate moieties.^29^ Subsequent protonation of anionic intermediate ^**1i**^ **INT1** or ^**1i**^ **INT4** yields product ^**1i**^ **P1**, an energetically most stable species among the possible products (Scheme 15), which is consistent with the experimental finding (**20** in Scheme 8).

**Figure 5:**
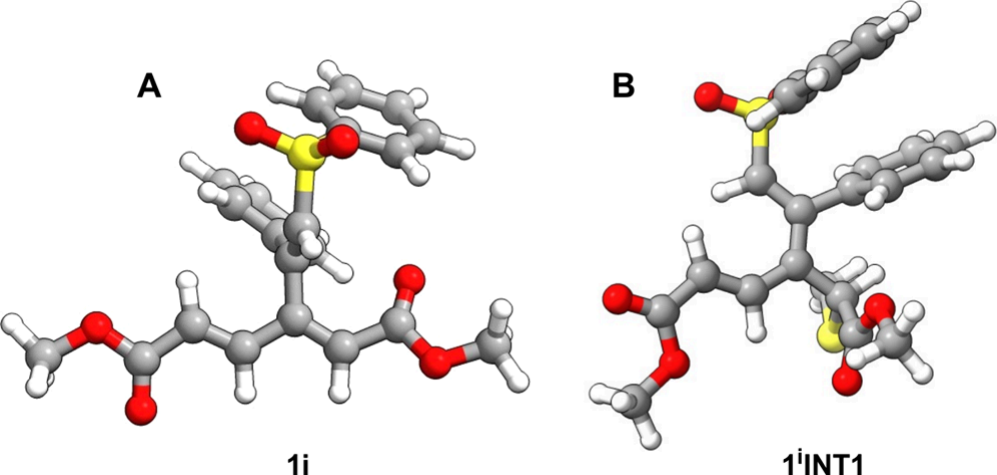
Most stable DFT*-*optimized conformers of (A) sulfone **1i** and (B) intermediate ^**1i**^ **INT1**.

**Scheme 15:**
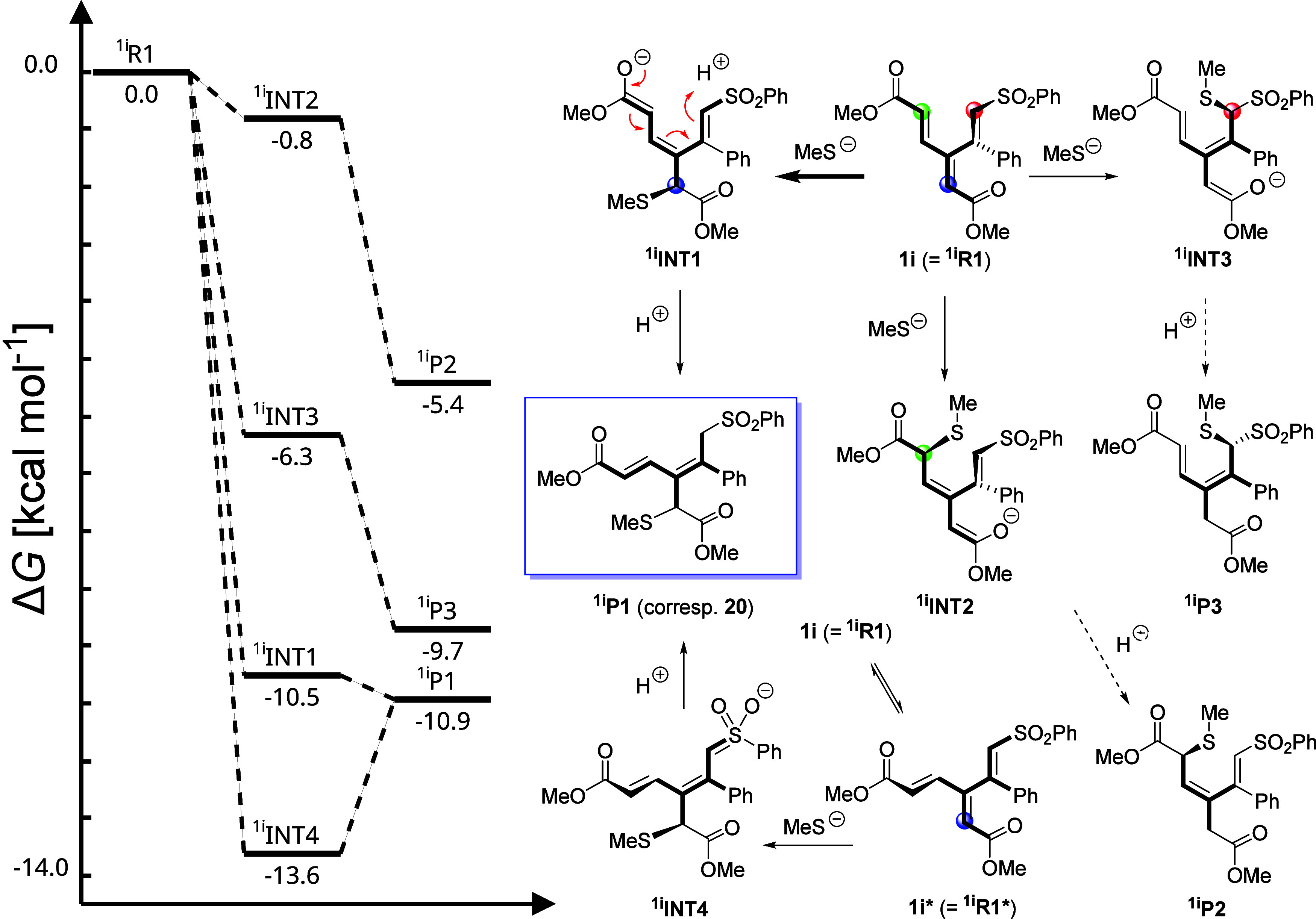
Illustration of Plausible Pathways for the Reaction of Sulfone **1i** (^**1i**^ **R1** in this Scheme) with Methanethiolate^a^

a The Gibbs free energies under the standard conditions (298.15 K and 1 M) were calculated at the ωB97M-V/def2-TZVPD//B3LYP-D3BJ/def2-TZVP level of theory. The experimentally observed pathway is highlighted by a bold arrow.

To conclude, reactions of thiolates with dendralenes were found to offer several pathways with negligible barriers, where the overall reactivity is governed by the thermodynamic stability of all possible products, with the exception of some cyclization reactions that are kinetically prohibited. In the case of **1a**, the DFT methods are not sufficiently accurate to provide useful selectivity predictions of these vast reaction networks (Figure S8). In other instances, such as in the cyclization product originating from **1b** or the addition of thiolate to **1i**, we were able to rationalize the observed outcomes based on the DFT results.

## Conclusions

In conclusion, this exploratory study of the chemistry of electron-deficient dendralenes with an enone fragment has, for the first time, demonstrated the variability of nucleophilic addition reactions to these intriguing compounds, proceeding in both Michael and anti-Michael fashions. The results showed that the initial nucleophilic attack triggers a variety of reaction paths, including simple additions on one hand or attractive multiple bond-forming cascades on the other. The actual reaction path depends on both the structure of the starting dendralene and the nature of the nucleophile. In general, the reactions gave rise to products, which no longer possessed like charges at the neighboring carbons or for which this “dissonant” disposition^10^ was diminished, as in **12f** and **12g**. Another factor with a profound influence, exercised in these reactions, seems to be the formation of a stable aromatic or bicyclic system. Specifically, hydride (from NaBH_4_) prefers Michael addition at the enone fragment, regardless of the substrate. However, in the case of compound **1a** with the five-membered cyclic enone, a simple double bond shift in the ensuing enolate gave rise to product **5** with alternating charges at the neighboring carbons. On the other hand, dendralenes **1b** and **1c** with larger rings and acyclic analogue **1d** afforded enolates that are capable of further cyclization to afford polysubstituted furans **7b**–**7d**, respectively, as the major products. Switching to thiolate nucleophiles showed a remarkable difference between cyclopentenone derivative **1a** and the rest of the series again. While **1a** underwent a simple 1,6-addition (anti-Michael) at the less hindered δ-position of the dienoate moiety, thiolate attack at **1b** and **1c** produced *ortho*-fused furans **17b** and **17c**, respectively, as a result of an initial β′-attack followed by cyclization. Density functional theory calculations suggest that nucleophilic additions of thiolates to dendralenes proceed (mostly) in a reversible fashion, making them mostly thermodynamically controlled (with the exception of some kinetically controlled cyclization reactions). For **1b**, furan ring formation serves as a thermodynamic sink, resulting in the formation of **17b** as the stable product. With stabilized C-nucleophiles, **1a** reacted to deliver bicyclo[4.3.0]­nonane systems **24x**–**24z** in a diastereoselective manner, while unexpectedly, **1b**–**1d** turned out to be inert. Finally, generating enolates via deprotonation confirmed the trend, with dendralene **1a** giving a complex mixture, while **1c** and **1d** afforded furan derivatives **33** and **35**, respectively, in high preparative yields. By seeming contrast, six-membered enone **1b** furnished coumarin derivative **31** with DBU, both as a stoichiometric base and as a catalyst. However, this cyclization mode was almost certainly dictated by the spontaneous aromatization of the six-membered carbocyclic ring even in the absence of air oxygen, an option not available with **1c** and **1d**. The sequences developed in this study are ready to be applied for the synthesis of natural products with the corresponding carbon skeletons or their analogues. Specifically, the facile formation of polysubstituted furans from simple, easy-to-make precursors is particularly notable, since a plethora of natural products, in which the furan core is fused to a six- or seven-membered saturated ring, such as zedoarol,^30^ crotoxides A and B,^31^ gnididione,^32^ and others,^33^ can be viewed as viable synthetic targets.

## Supplementary Material





## Data Availability

The data underlying this study are available in the published article and its .

## Notes

FAIR data, including the primary NMR FID files, for compounds **1a–1g**, **1i**, **5**, **7b–7d**, **9b**, **9d**, **10**, **11**, **12f**, **12g**, **14**, **15**, **17b**, **17c**, **18b**, **18c**, **24x–24z**, **28**, **31**, **33**, and **35** (ZIP)
Experimental procedures and spectroscopic data for all compounds, X-ray crystallographic data, computational details, and copies of NMR spectra (PDF)
