# Supplementary material for: Generating Molecular Diversity via Addition of Nucleophiles to Electron-Deficient [3]Dendralenes: An Exploratory Study
Source: J Org Chem. 2026 Jan 14;91(4):1588–601. doi: 10.1021/acs.joc.5c02397 (PMC12865776; doi:10.1021/acs.joc.5c02397)
Supplement: Supplementary file 2 [file jo5c02397_si_002.pdf]

Supporting information for

Generating Molecular Diversity via Addition of Nucleophiles to Electron-Deficient [3]Dendralenes: An Exploratory Study

Stefanie Magela Perdomo,[a] Ondřej Kratochvíl,[a] Rastislav Antal,[a] Michal Kadaník,[a] Petr Matouš,[a] Jiří Kuneš,[a] Aleš Růžička[b], Adam Kurčina[c], Lubomír Rulíšek,[c] Erik Andris,[c,*] Pavel Kočovský,[a,d,*] and Milan Pour[a,*]

[a.] *Department of Organic and Bioorganic Chemistry, Charles University, Faculty of Pharmacy in Hradec Králové, Heyrovského 1203, 500 05 Hradec Králové, Czech Republic; E-mail: pour@faf.cuni.cz*
[b.] *Department of General and Inorganic Chemistry, University of Pardubice, Faculty of Chemical Technology, 532 10 Pardubice, Czech Republic*
[c.] *Institute of Organic Chemistry and Biochemistry of the Czech Academy of Sciences, Flemingovo náměstí 542/2, 160 00 Prague 6, Czech Republic; E-mail: erik.andris@uochb.cas.cz*
[d.] *Department of Organic Chemistry, Charles University, Faculty of Science, Hlavova 8, 128 43 Prague 2, Czech Republic*

1. Table of Contents

1. Table of Contents S2
2. General information S3
3. Synthetic procedures and characterizations........ S3
    3.1 Synthesis and characterization of dendralenes 1a–1g, 1i........ S3
    3.2 Nucleophilic additions using $NaBH_4$ as nucleophile S7
        3.2.1 Reaction with dendralene **1a** S7
        3.2.2 Reaction with dendralene **1b** S8
        3.2.3 Reaction with dendralene **1c**........ S9
        3.2.4 Reaction with dendralene **1d** S10
        3.2.5 Reaction with dendralenes **1f** and **1g**........ S11
        3.2.6 Luche reduction S12
    3.3 Thiolates as nucleophiles S13
        3.3.1 Reaction with dendralene **1a** S13
        3.3.2 Reaction with dendralenes **1b** and **1c** S14
        3.3.3 Reaction with dendralene **1i** S16
    3.1 Active methylenes as nucleophiles S17
    3.2 Reaction with nitromethane S19
    3.3 Reaction with DBU........ S20
    3.4 Deuterium labeling experiments S21
4. X-Ray analysis S23
5. Computational details........ S30
    Geometry of reactants S32
    Fukui function S32
6. Copies of NMR spectra S61
7. References S104

2. General information

Reagents and solvents were purchased from Sigma-Aldrich (Merck, KGaA, Darmstadt, Germany) and used without further purification. Dendralene **1h** and (2*Z*,4*E*)-3-(tributylstannyl)hexa-2,4-dienedioate were used from the laboratory's stock of previously prepared compounds according to the established methodology.[1] Solvents (DCM, THF) were dried prior to use (PureSolv PS-Micro, Innovative Technologies). The reactions were carried out in oven-dried glassware using Schlenk line techniques with magnetic stirring and dried solvents under Ar. TLC analyses were performed using Merck TLC Silica gel F254 TLC plates and visualized by UV (254 nm) in combination with staining (using the solution of $Ce(SO_4)_2 \cdot 4H_2O$ (2 g), $H_3[P(Mo_3O_{10})_4]$ (4 g), conc. H_2SO_4 (10 mL) and H_2O (200 mL) with subsequent heating). Column chromatography was carried out on Merck Silica gel 60 (0.040–0.063 mm). ^1H and ^{13}C NMR spectra were recorded with a Varian VNMR S500 or Jeol JNM-ECZ600R instrument. The chemical shifts were recorded as δ values in parts per million (ppm), reported relative to TMS and referenced to the residual solvent peaks. Coupling constants (J) are given in Hz. The following abbreviations were used to designate the multiplicities: s = singlet; d = doublet; t = triplet; m = multiplet; dt = doublet of triplets; dd = doublet of doublets; ddd = doublet of doublets of doublets; br = broad. Structural assignments were made with additional information from gCOSY, gHSQC and gHMBC experiments. IR spectra were recorded on a NICOLET 6700 FT-IR equipped with an ATR device. HR-MS data were recorded on a QTOF mass spectrometer using the electrospray ionization. ESI-MS spectra were obtained on an Agilent LC/MSD SL spectrometer or Expression^L CMS in connection with Plate Express®, Advion, Inc. (USA) instrument. Crystals for X-Ray analysis of compounds **(*E*)-1i**, **10**, and **24x** were grown using vapor diffusion technique in the solvent system specified below. Crystallographic data were obtained from a Bruker D8 Venture diffractometer. Melting points were determined on a Stuart SMP30 apparatus without correction. Heating of reaction mixtures was provided by an oil bath, unless stated otherwise. Cooling to subzero temperatures was provided by Julabo FT902 FT Immersion Cooler combined with an ethanol bath.

3. Synthetic procedures and characterizations

3.1 Synthesis and characterization of dendralenes 1a–1g, 1i

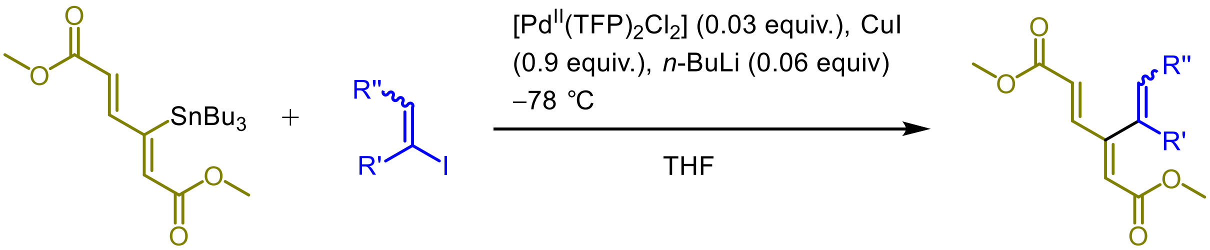

General procedure: a solution of *n*-BuLi in THF (2.5 M; 0.06 equiv.; 48 µL) was added dropwise to a suspension of $Pd(TFP)_2Cl_2$ (0.03 equiv.; 0.06 mmol; 39 mg) and CuI (0.9 equiv.; 1.8 mmol; 343 mg) in THF (1 mL/mmol; 2 mL) at −78 °C. The cooling was stopped, and the mixture was gradually heated up to −30 °C for 3 h. After 15 min of stirring at this temperature, the solution of the corresponding iodoalkene (1.25 equiv.; 2.5 mmol) in DMF (1 mL/mmol; 2 mL) was transferred to the reaction mixture and after another 15 min, the solution of dimethyl (2*Z*,4*E*)-3-(tributylstannyl)hexa-2,4-dienedioate[1]

(1 equiv.; 2 mmol) in anhydrous DMF (1 mL/mmol; 2 mL) was added. The cooling was stopped, and the reaction mixture was stirred to achieve RT overnight, then diluted with EtOAc (20 mL) and washed with a saturated solution of NH_4Cl (2 × 20 mL). The aqueous phases were extracted with EtOAc (10 mL) and the combined organic phases were further washed with 4% solution of NaF (2 × 30 mL). The precipitate formed was removed from the organic phase using filtration through a cotton pad over a Büchner funnel. The organic phase was dried over Na_2SO_4, the inorganic residues were filtered off and the solution was evaporated to dryness. The residue was purified by chromatography on a column of silica gel using the mobile phase giving below to afford the corresponding [3]dendralene.[1]

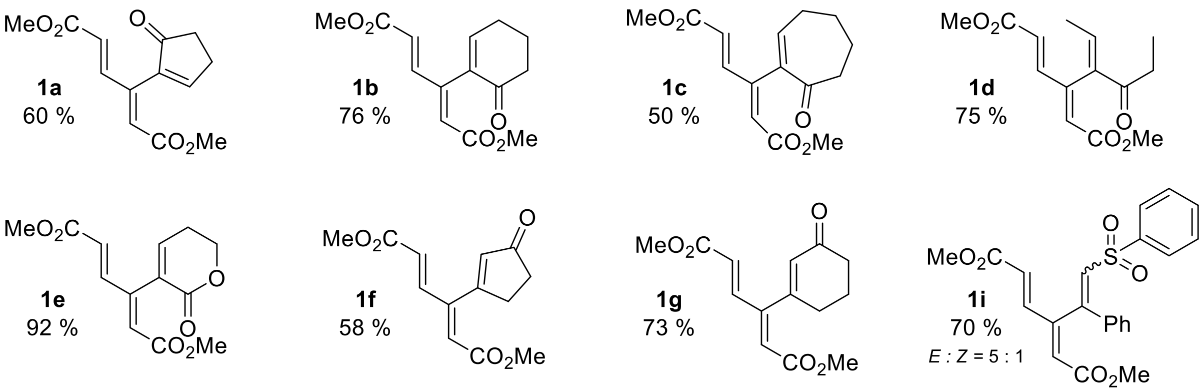

Figure S1. Structure of [3]dendralenes synthesized

Dimethyl (2*Z*,4*E*)-3-(5-oxocyclopent-1-en-1-yl)hexa-2,4-dienedioate (1a): Synthesized following the general procedure, using 2-iodocyclopent-2-en-1-one[3] (2.5 mmol, 0.520 g), (2*Z*,4*E*)-3-(tributylstannyl)hexa-2,4-dienedioate (2 mmol, 0.9 g). Purification by column chromatography (hx → 70 : 30 hx/EtOAc) gave the title compound as white crystalline solid, m. p. 93.8–94.2 °C (recrystallized from hexane, vapor diffusion tech); 60% yield (0.298 g). **^{1}H NMR** (600 MHz, $CDCl_3$): δ 7.45 (t, *J* = 2.8 Hz, 1H), 7.38 (d, *J* = 15.6 Hz, 1H), 6.24 (s, 1H), 5.93 (d, *J* = 15.6 Hz, 1H), 3.75 (s, 3H), 3.66 (s, 3H), 2.79–2.77 (m, 2H), 2.56–2.53 (m, 2H). **^{13}C{^{1}H} NMR** (151 MHz, $CDCl_3$): δ 205.3, 166.3, 165.3, 160.2, 144.4, 142.9, 141.5, 127.1, 125.3, 52.1, 51.8, 34.4, 27.4. The recorded values were in agreement with the published data.[1]

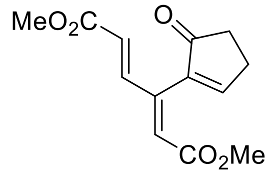

$C_{13}H_{14}O_5$
MW: 250.25

Dimethyl (2*Z*,4*E*)-3-(6-oxocyclohex-1-en-1-yl)hexa-2,4-dienedioate (1b): Synthesized following the general procedure, using 2-iodocyclohex-2-en-1-one[3] (2.5 mmol, 0.555 g), (2*Z*,4*E*)-3-(tributylstannyl)hexa-2,4-dienedioate[1] (2 mmol, 0.9 g). Purification by column chromatography (hx → 70 : 30 hx/EtOAc) gave the title compound as white crystalline solid, m. p. 108.3–109.4 °C (recrystallized from hexane); 76% yield (0.401 g). **^{1}H NMR** (500 MHz, $CDCl_3$): δ 7.39 (d, *J* = 15.7 Hz, 1H), 6.67 (t, *J* = 4.1 Hz, 1H), 6.19 (s, 1H), 5.98 (d, *J* = 15.7 Hz, 1H), 3.76 (s, 3H), 3.67 (s, 3H), 2.61–2.57 (m, 2H), 2.53–2.48 (m, 2H), 2.18–2.11 (m, 2H). **^{13}C{^{1}H} NMR** (126 MHz, $CDCl_3$): δ 196.3, 166.4, 165.3, 147.5, 147.0, 145.1, 136.2, 126.4, 125.2, 51.9, 51.5, 38.3, 26.0, 22.6. The recorded values were in agreement with the published data.[1]

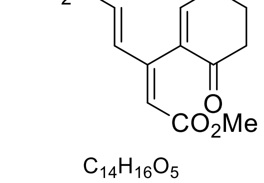

$C_{14}H_{16}O_5$
MW: 264.28

Dimethyl (2*Z*,4*E*)-3-(7-oxocyclohept-1-en-1-yl)hexa-2,4-dienedioate (1c): Synthesized following the general procedure, using 2-iodocyclohept-2-en-1-one[4] (2.5 mmol, 0.590 g), (2*Z*,4*E*)-3-(tributylstannyl)hexa-2,4-dienedioate[1] (2 mmol, 0.9 g). Purification by column chromatography (hx → 70 : 30 hx/EtOAc) gave the title compound as white amorphous solid; 50% yield (0.348 g). **^{1}H NMR** (500 MHz, $CDCl_3$): δ 7.41 (d, *J* = 15.6 Hz, 1H), 6.39 (t, *J* = 6.1 Hz, 1H), 6.12 (s, 1H), 6.02 (d, *J* = 15.6 Hz, 1H), 3.76 (s, 3H), 3.68 (s, 3H), 2.82–2.74 (m, 2H), 2.55 (dt, *J* = 6.1 Hz, *J* = 5.5 Hz, 2H), 1.98–1.84 (m, 4H). **^{13}C{^{1}H} NMR** (126 MHz, $CDCl_3$): δ 202.2, 166.5, 165.5, 150.6, 146.0, 144.0, 139.4, 125.5, 125.0, 51.9, 51.6, 43.0, 28.3, 25.8, 21.6. **IR** (ATR-Ge): ν_{max} [cm^{-1}] 2953, 1712, 1659, 1434, 1252, 1191. **LR-MS** (APCI$^+$): *m/z* $[M+H]^+$ calcd. for $C_{15}H_{19}O_5^+$ 279.3, found 279.2. **HR-MS** (TOF-ESI$^+$): *m/z* $[M+Na]^+$ calcd. for $C_{15}H_{18}O_5Na^+$ 301.1046, found 301.1053; $[M+H-CH_3OH]^+$ calcd. $C_{14}H_{15}O_4^+$ 247.0965, found 247.0973.

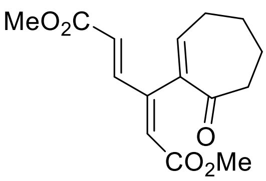

$C_{15}H_{18}O_5$
MW: 278.30

Dimethyl (2*Z*,4*E*)-3-((*E*)-4-oxohex-2-en-3-yl)hexa-2,4-dienedioate (1d): Synthesized following the modified general procedure, using (*Z*)-4-iodohex-4-en-3-one[2] (13.84 mmol, 3.1 g, 1.2 equiv.), (2*Z*,4*E*)-3-(tributylstannyl)hexa-2,4-dienedioate (11.53 mmol, 5.295 g, 1 equiv.). Purification by column chromatography (hx → 70 : 30 hx/EtOAc) gave the title compound as orangish oil, 75% yield (2.285 g). **^{1}H NMR** (500 MHz, $CDCl_3$): δ 7.39 (d, *J* = 15.6 Hz, 1H), 6.95 (q, *J* = 7.0 Hz, 1H), 6.29 (s, 1H), 5.92 (d, *J* = 15.6 Hz, 1H), 3.75 (s, 3H), 3.66 (s, 3H), 2.75–2.62 (m, 2H), 1.66 (d, *J* = 7.0 Hz, 3H), 1.10 (t, *J* = 7.3 Hz, 3H). **^{13}C{^{1}H} NMR** (126 MHz, $CDCl_3$): δ 198.7, 166.4, 165.1, 148.1, 143.8, 138.3, 137.7, 127.1, 125.2, 51.9, 51.6, 31.1, 15.5, 8.1. The recorded values were in agreement with the published data.[1]

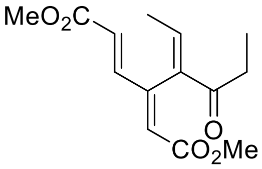

$C_{14}H_{18}O_5$
MW: 264.28

Dimethyl (2*Z*,4*E*)-3-(2-oxo-5,6-dihydro-2*H*-pyran-3-yl)hexa-2,4-dienedioate (1e): Synthesized following the general procedure, using 3-iodo-5,6-dihydro-2*H*-pyran-2-one[4] (2.5 mmol, 0.560 g), (2*Z*,4*E*)-3-(tributylstannyl)hexa-2,4-dienedioate[1] (2 mmol, 0.9 g). Purification by column chromatography (hx → 50 : 50 hx/EtOAc) gave the title compound as white amorphous solid; 92% yield (0.61 g). **^{1}H NMR** (500 MHz, $CDCl_3$): δ 7.41 (d, *J* = 15.6 Hz, 1H), 6.62 (t, *J* = 4.4 Hz, 1H), 6.21 (s, 1H), 6.09 (d, *J* = 15.6 Hz, 1H), 4.57 (t, *J* = 6.1 Hz, 2H), 3.77 (s, 3H), 3.71 (s, 3H), 2.59 (td, *J* = 6.1 Hz, *J* = 4.4 Hz, 2H). **^{13}C{^{1}H} NMR** (126 MHz, $CDCl_3$): δ 166.2, 165.4, 162.6, 146.4, 144.1, 140.9, 129.5, 126.5, 125.8, 66.5, 52.0, 51.8, 24.2. **IR** (ATR-Ge): ν_{max} [cm^{-1}] 1712, 1593, 1431, 1235, 1175. **LR-MS** (APCI$^+$): *m/z* $[M+H]^+$ calcd. for $C_{13}H_{15}O_6^+$ 267.3, found 267.3. **HR-MS** (TOF-ESI$^+$): *m/z* $[M+Na]^+$ calcd. for $C_{13}H_{14}O_6Na^+$ 289.0683, found 289.0685; $[M+H-CH_3OH]^+$ calcd. for $C_{12}H_{11}O_5$ 235.0601, found 235.0604.

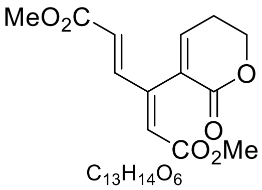

$C_{13}H_{14}O_6$
MW: 266.25

Dimethyl (2*Z*,4*E*)-3-(3-oxocyclopent-1-en-1-yl)hexa-2,4-dienedioate (1f): Synthesized following the general procedure, using 3-iodocyclopent-2-en-1-one[5] (2.5 mmol, 0.520 g), (2*Z*,4*E*)-3-(tributylstannyl)hexa-2,4-dienedioate (2 mmol, 0.9 g). Purification by column chromatography (hx → 70 : 30 hx/EtOAc), gave the title compound as beige crystalline solid, m.p. 122.7–124.2 °C (recrystallized from hexane); 58% yield (0.292 g). **^{1}H NMR** (500 MHz, $CDCl_3$): δ 7.36 (d, *J* = 15.8 Hz, 1H), 6.12 (s, 1H), 6.02 (s, 1H), 6.00 (d, overlap, *J* = 15.8 Hz, 1H), 3.79 (s, 3H), 3.73 (s, 3H), 2.86–2.80 (m, 2H), 2.60–2.55 (m, 2H). **^{13}C{^{1}H} NMR** (126 MHz, $CDCl_3$): δ 208.3, 172.7, 166.0, 164.7, 148.4, 142.1, 132.4, 126.1, 124.5, 52.1, 52.0, 35.2, 31.8. The recorded values were in agreement with the published data.[1]

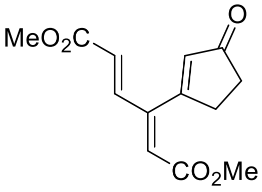

$C_{13}H_{14}O_5$
MW: 250.25

Dimethyl (2*Z*,4*E*)-3-(3-oxocyclohex-1-en-1-yl)hexa-2,4-dienedioate (1g): Synthesized following the general procedure, using 3-iodocyclohex-2-en-1-one[5] (2.5 mmol, 0.555 g), (2*Z*,4*E*)-3-(tributylstannyl)hexa-2,4-dienedioate (2 mmol, 0.9 g). Purification by column chromatography (hx → 70 : 30 hx/EtOAc), gave the title compound as beige crystalline solid, m.p. 103.1–103.9 °C (recrystallized from EtOAc/hexane mixture); 73% yield (0.385 g). **^{1}H NMR** (600 MHz, $CDCl_3$): δ 7.29 (d, *J* = 15.8 Hz, 1H), 6.02 (s, 1H), 6.02 (d, overlap, *J* = 15.8 Hz, 1H), 5.76 (s, 1H), 3.79 (s, 3H), 3.73 (s, 3H), 2.51–2.46 (m, 2H), 2.46–2.42 (m, 2H), 2.19–2.13 (m, 2H). **^{13}C{^{1}H} NMR** (151 MHz, $CDCl_3$): δ 198.4, 166.1, 165.0, 158.5, 151.3, 142.7, 127.8, 126.1, 124.1, 52.2, 52.0, 38.3, 26.0, 22.6. The recorded values were in agreement with the published data.[1]

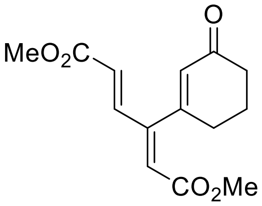

$C_{14}H_{16}O_5$
MW: 264.28

Dimethyl (2*Z*,4*E*)-3-((*E*)-1-phenyl-2-(phenylsulfonyl)vinyl)hexa-2,4-dienedioate (1i): Synthesized following the general procedure, using (*E*)-(1-iodo-2-(phenylsulfonyl)vinyl)benzene[6] (2.5 mmol, 0.925 g), (2*Z*,4*E*)-3-(tributylstannyl)hexa-2,4-dienedioate[1] (2 mmol, 0.9 g). Purification by column chromatography (hx → 70 : 30 hx/EtOAc) gave a mixture of two diastereoisomers in the ratio (*E*) : (*Z*) = 5 : 1 (70 %), after precipitation in hexane the major isomer (*E*) was obtained as white crystalline solid (50% yield after precipitation), m. p. 117.2–118 °C (recrystallized from hexane). (*Z*)-isomer: **^{1}H NMR** (500 MHz, $CDCl_3$): δ 7.75–7.69 (m, 2H), 7.52–7.48 (m, 1H), 7.46–7.45 (m, 1H), 7.44–7.42 (m, 2H), 7.41–7.36 (m, 2H), 7.35–7.30 (m, 1H), 7.30–7.25 (m, 2H), 7.23 (d, *J* = 15.9 Hz, 1H), 6.32–6.31 (m, 1H), 5.91–5.87 (dd, *J* =15.7 Hz, *J* = 0.4 Hz, 1H), 3.76 (s, 3H), 3.68 (s, 3H). (*E*)-isomer: **^{1}H NMR** (500 MHz, $CDCl_3$): δ 7.75–7.69 (m, 2H), 7.52–7.48 (m, 1H), 7.45–7.42 (m, 2H), 7.41–7.36 (m, 2H), 7.35–7.30 (m, 1H), 7.30–7.25 (m, 2H), 7.23 (d, *J* = 15.9 Hz, 1H), 6.47 (s, 1H), 6.15–6.14 (m, 1H), 6.10 (dd, *J* = 15.9 Hz, *J* = 0.6 Hz, 1H), 3.74 (s, 3H), 3.67 (s, 3H). **^{13}C{^{1}H} NMR** (126 MHz, $CDCl_3$): δ 165.9, 164.5, 149.3, 149.0, 142.9, 140.3, 133.2, 133.0, 131.3, 129.8, 129.7, 128.6, 128.0, 127.9, 126.9, 126.0, 52.0, 51.9. **IR** (ATR-Ge): ν_{max} [cm^{-1}] 1722, 1609, 1302, 1285, 1229, 1170, 1147. **LR-MS** (APCI$^+$): *m/z* [M+H]$^+$ calcd. for $C_{22}H_{21}O_6S^+$ 413.5, found 413.1. **HR-MS** (TOF-ESI$^+$): *m/z* [M+H]$^+$ calcd. for $C_{22}H_{21}O_6S^+$ 413.1053, found 413.1056; [M+Na]$^+$ calcd. for $C_{22}H_{20}O_6SNa^+$ 435.0873, found 435.0877.

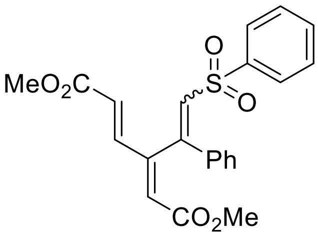

$C_{22}H_{20}O_6S$
MW: 412.46

3.2 Nucleophilic additions using $NaBH_4$ as nucleophile

3.2.1 Reaction with dendralene **1a**

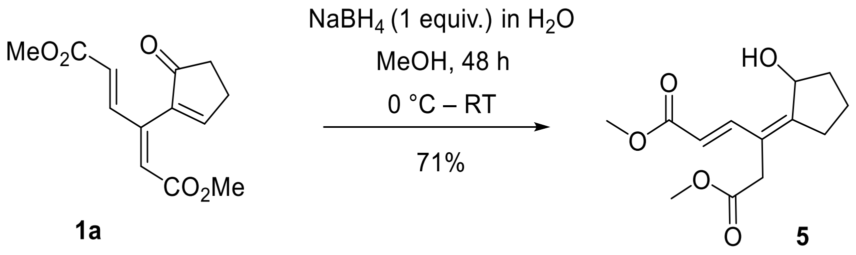

Dimethyl (2*E*,4*Z*)-4-(2-hydroxycyclopentylidene)hex-2-enedioate (5): The solution of $NaBH_4$ (1 mmol, 0.038 g) in water (0.1 mL) was added to the stirred solution of dendralene **1a** (1 mmol, 0.250 g) in methanol (10 mL) at 0 °C.[7] Then, the reaction mixture was stirred at RT for 48h. When TLC indicated completion, 5% HCl (20 mL) was added and the resulting solution was extracted with ethyl acetate (20 mL × 2). Subsequently, the combined organic layers were washed with saturated NaCl solution (40 mL), dried over anhydrous Na_2SO_4, filtered and concentrated in vacuo. The residue was purified by chromatography on a column of silica gel using the mobile phase hx/EtOAc (90 : 10 → 70 : 30) to afford the product **5** as yellowish oil, 71% yield (0.181 g). **^{1}H NMR** (500 MHz, $CDCl_3$): δ 7.86 (d, J = 15.8 Hz, 1H, H2), 5.87 (d, J = 15.8 Hz, 1H, H3), 5.03–4.97 (m, 1H, H2'), 3.75 (s, 3H, 6-OCH_3), 3.68 (s, 3H, 1-OCH_3), 3.47 (s, 1H, OH), 3.29 (s, 2H, H5), 2.67–2.57 (m, 1H, H5'), 2.42–2.31 (m, 1H, H5'), 2.01–1.71 (m, 4H, H4', H3'). **^{13}C{^{1}H} NMR** (126 MHz, $CDCl_3$): δ 171.0 (C6), 167.7 (C1), 158.1 (C1'), 142.2 (C3), 125.2 (C4), 117.0 (C2), 71.6 (C2'), 52.1 (1-OCH_3), 51.6 (6-OCH_3), 36.1 (C3'), 35.3 (C5), 31.1 (C5'), 22.5 (C4'). **IR** (ATR-Ge): ν_{max} [cm^{-1}] 3492, 2953, 1717, 1435, 1172. **LR-MS** (APCI$^+$): m/z $[M+H]^+$ calcd. for $C_{13}H_{19}O_5^+$ 255.3, found 255.1. **HR-MS**: (TOF-ESI$^+$): m/z $[M+Na]^+$ calcd. for $C_{13}H_{18}O_5Na^+$ 277.1046, found 277.1052.

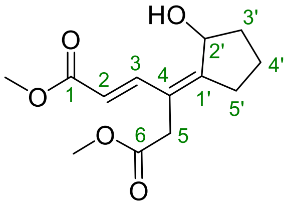

$C_{13}H_{18}O_5$
MW: 254.28

3.2.2 Reaction with dendralene **1b**

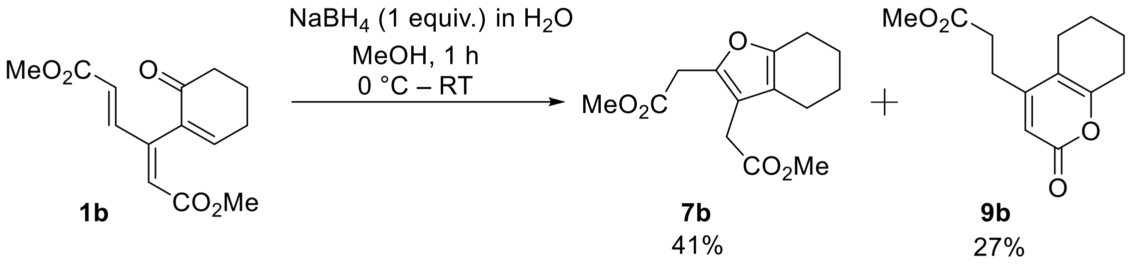

Procedure: Solution of $NaBH_4$ (0.5 mmol, 0.019 g) in water (0.05 mL) was added to the stirred solution of dendralene **1b** (0.5 mmol, 0.132 g) in methanol (5 mL) at 0 °C.[7] The reaction mixture was then stirred at RT for 1h. When TLC indicated the completion, 5% HCl (10 mL) was added and the resulting solution was extracted with ethyl acetate (10 mL × 2). Subsequently, the combined organic layers were washed with saturated NaCl solution (20 mL), dried over anhydrous Na_2SO_4, filtered and concentrated in vacuo. The residue was purified by chromatography on a column of silica gel using the mobile phase (hx → 70 : 30 hx/EtOAc) affording **7b** as colorless oil, 41% yield (0.055 g) and **9b** as white amorphous solid, 27% yield (0.032 g).

Dimethyl 2,2'-(4,5,6,7-tetrahydrobenzofuran-2,3-diyl)diacetate (7b): **^{1}H NMR** (500 MHz, $CDCl_3$): δ 3.71 (s, 3H, 2'-OCH_3), 3.69 (s, 3H, 2''-OCH_3), 3.65 (s, 2H, H1'), 3.34 (s, 2H, H1''), 2.55 (tt, J = 6.3 Hz, J = 2.0 Hz, 2H, H7), 2.34 (tt, J = 6.0 Hz, J = 1.9 Hz, 2H, H4), 1.84–1.78 (m, 2H, H6), 1.75–1.69 (m, 2H, H5). **^{13}C{^{1}H} NMR** (126 MHz, $CDCl_3$): δ 171.4 (C2''), 170.0 (C2'), 150.0 (C7a), 142.3 (C2), 118.1 (C3a), 114.3 (C3), 52.2 (2'-OCH_3), 52.0 (C2''-OCH_3), 32.4 (C1'), 29.4 (C1''), 23.0 (C7), 22.8 (C6), 22.7 (C5), 20.6 (C4). **IR** (ATR-Ge): ν_{max} [cm^{-1}] 2927, 2851, 1738, 1435, 1270, 1166. **LR-MS** (TOF-ESI+): m/z $[M+H]^+$ calcd. for $C_{14}H_{19}O_5^+$ 267.3, found 267.2. **HR-MS** (TOF-ESI+): m/z $[M+H]^+$ calcd. for $C_{14}H_{19}O_5^+$ 267.1227, found 267.1230; $[M+Na]^+$ calcd. for $C_{14}H_{18}O_5Na^+$ 289.1046, found 289.1056.

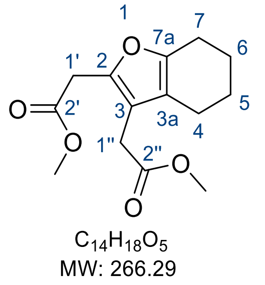

Methyl 3-(2-oxo-5,6,7,8-tetrahydro-2*H*-chromen-4-yl)propanoate (9b): **^{1}H NMR** (500 MHz, $CDCl_3$): δ 5.94 (s, 1H, H3), 3.71 (s, 3H, 3'-OCH_3), 2.74–2.67 (m, 2H, H1'), 2.63–2.56 (m, 2H, H2'), 2.56–2.49 (m, 2H, H8), 2.42–2.35 (m, 2H, H5), 1.84–1.71 (m, 4H, H7, H6). **^{13}C{^{1}H} NMR** (126 MHz, $CDCl_3$): δ 172.3 (C3'), 162.6 (C2), 159.2 (C8a), 158.3 (C4), 112.5 (C4a), 109.9 (C3), 52.0 (C3'-OCH_3), 31.5 (C2'), 27.7 (C8), 26.6 (C1'), 22.7 (C5), 22.0 (C7), 21.5 (C6). **IR**: (ATR-Ge) ν_{max} [cm^{-1}] 2950, 1736, 1706, 1431, 1188, 1165. **LR-MS** (TOF-ESI$^+$): m/z $[M+H]^+$ calcd. for $C_{13}H_{17}O_4^+$ 237.3, found 237.1. **HR-MS**: (TOF-ESI$^+$): m/z calcd. for $C_{13}H_{17}O_4^+$ $[M+H]^+$ 237.1121, found 237.1131; $[M+Na]^+$ calcd. for $C_{13}H_{16}O_4Na^+$ 259.0941, found 259.0949.

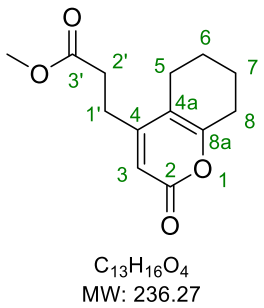

3.2.3 Reaction with dendralene **1c**

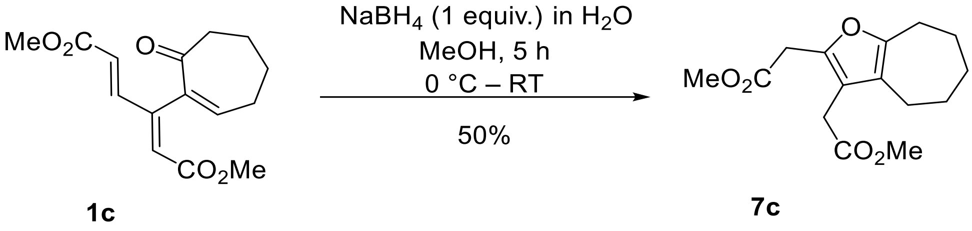

Dimethyl 2,2'-(5,6,7,8-tetrahydro-4*H*-cyclohepta[*b*]furan-2,3-diyl)diacetate (7c): The solution of

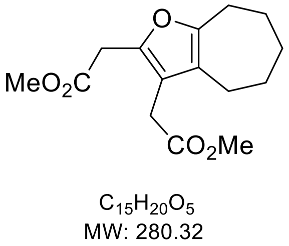

$NaBH_4$ (0.5 mmol, 0.019 g) in water (0.05 ml) was added to the stirred solution of dendralene **1c** (0.5 mmol, 0.139 g) in methanol (5 mL) at 0 °C.[7] Then, the reaction mixture was stirred at RT. When TLC indicated completion (5 h), 5% HCl (10 mL) was added and the resulting solution was extracted with ethyl acetate (10 mL × 2). Subsequently, the combined organic layers were washed with saturated NaCl solution (20 mL), dried over anhydrous Na_2SO_4 , filtered and concentrated in vacuo. The residue was purified by chromatography on a column of silica gel using the mobile phase (hx → 70 : 30 hx/EtOAc) giving the title compound as colorless oil, 50% yield (0.07 g). **^{1}H NMR** (600 MHz, $CDCl_3$): δ 3.66 (s, 3H), 3.64 (s, 3H), 3.57 (s, 2H), 3.30 (s, 2H), 2.71–2.66 (m, 2H), 2.36–2.30 (m, 2H), 1.76–1.60 (m, 6H). **^{13}C{^{1}H} NMR** (151 MHz, $CDCl_3$): δ 171.6, 170.1, 152.6, 141.0, 121.4, 115.8, 52.2, 52.0, 32.4, 30.7, 29.5, 28.9, 28.4, 26.4, 24.1. **IR** (ATR-Ge): ν_{max} [cm^{-1}] 2922, 2847, 1738, 1435, 1262, 1167. **LR-MS** (APCI$^+$): *m*/*z* [M+H]$^+$ calcd. for $C_{15}H_{21}O_5^+$ 281.3, found 281.4. **HR-MS** (TOF-ESI$^+$): *m/z* [M+H]$^+$ calcd. for $C_{15}H_{21}O_5^+$ 281.1384, found 281.1387.

3.2.4 Reaction with dendralene **1d**

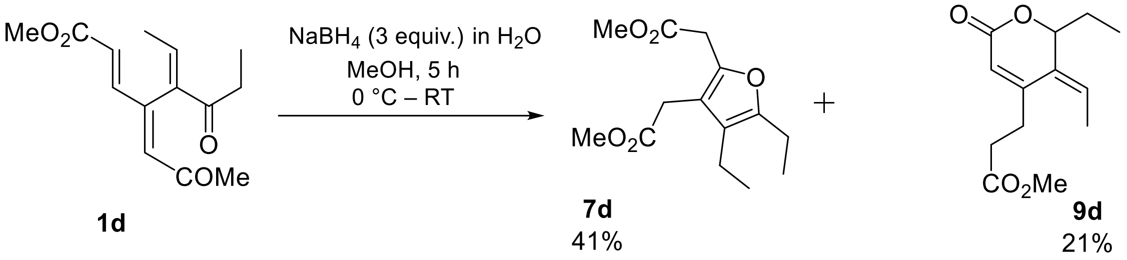

The solution of $NaBH_4$ (0.057 g, 1.5 mmol) in water (0.2 ml) was added to the stirred solution of dendralene **1d** (0.132 g, 0.5 mmol) in methanol (5 mL) at 0 °C.[7] The reaction mixture was stirred at RT for 5 h until the TLC indicated the completion. Then, 5% HCl (10 mL) was added and the resulting solution was extracted with ethyl acetate (10 mL × 2). Subsequently, the combined organic layers were washed with saturated NaCl solution (20 ml), dried over anhydrous Na_2SO_4, filtered and evaporated. The residue was purified by chromatography on a column of silica gel using the mobile phase (hx→ 70 : 30 hx/EtOAc) giving the compound **7d** as yellowish oil, 41% yield (0.055 g), and the compound **9d** as yellowish oil, 21% yield (0.025 g).

Dimethyl 2,2'-(4,5-diethylfuran-2,3-diyl)diacetate (7d): **^{1}H NMR** (500 MHz, $CDCl_3$): δ 3.70 (s, 3H), 3.68 (s, 3H), 3.64 (s, 2H), 3.36 (s, 2H), 2.56 (q, J = 7.6 Hz, 2H), 2.32 (q, J = 7.6 Hz, 2H), 1.18 (t, J = 7.6 Hz, 3H), 1.06 (t, J = 7.6 Hz, 3H). **^{13}C{^{1}H} NMR**: (126 MHz, $CDCl_3$): δ 171.6, 170.0, 151.6, 142.2, 120.4, 114.9, 52.1, 52.0, 32.5, 29.4, 19.6, 16.6, 15.3, 13.2. **IR** (ATR-Ge): ν_{max} [cm^{-1}] 2966, 1739, 1435, 1269, 1163. **LR-MS** (APCI$^+$): m/z [M+H]$^+$ calcd. for $C_{14}H_{21}O_5^+$ 269.3, found 269.1. **HR-MS** (TOF-ESI$^+$): m/z [M+Na]$^+$ calcd. for $C_{14}H_{21}O_5Na^+$ 291.1203, found 291.1217.

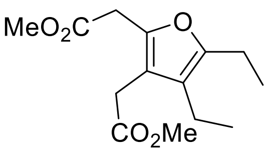

$C_{14}H_{20}O_5$
MW: 268.31

Methyl (*E*)-3-(2-ethyl-3-ethylidene-6-oxo-3,6-dihydro-2*H*-pyran-4-yl)propanoate (9d):

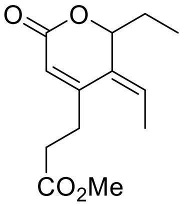

$C_{13}H_{18}O_4$
MW: 238.28

^{1}H NMR (500 MHz, $CDCl_3$): δ 5.84 (q, overlap, J = 7.6 Hz, 1H), 5.81 (s, overlap, 1H), 4.58 (t, J = 7.3 Hz, 1H), 3.71 (s, 3H), 3.01–2.91 (m, 1H), 2.89–2.80 (m, 1H), 2.59–2.53 (m, 2H), 1.96 (d, J = 7.6 Hz, 3H), 1.69–1.59 (m, 2H), 0.92 (t, J = 7.5 Hz, 3H). **^{13}C{^{1}H} NMR**: (126 MHz, $CDCl_3$): δ 172.3, 163.8, 152.8, 130.9, 130.5, 117.8, 86.2, 51.9, 31.8, 29.9, 27.1, 15.4, 10.1. **IR**: (ATR-Ge) ν_{max} [cm^{-1}] 2923, 2853, 1733, 1458, 1166. **LR-MS** (APCI$^+$): m/z [M+H]$^+$ calcd. for $C_{13}H_{19}O_5^+$ 239.3, found 239.1. **HR-MS** (TOF-ESI$^+$): m/z calcd. for $C_{13}H_{19}O_4^+$ [M+H]$^+$ 239.1278, found 239.1282; m/z [M+Na]$^+$ calcd. for $C_{13}H_{18}O_4Na^+$ 261.1097, found 261.1102.

3.2.5 Reaction with dendralenes **1f** and **1g**

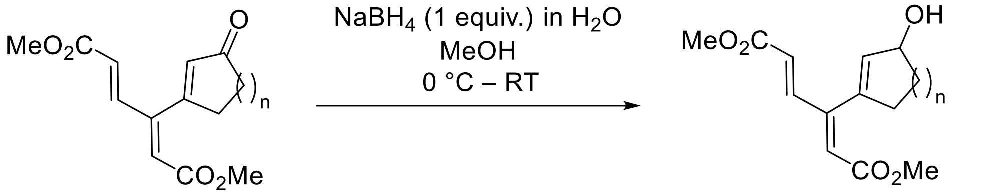

1f, n = 1
1g, n = 2

12f, n = 1 (35% mixture)
12g, n = 2 (56%)

General method: The solution of $NaBH_4$ in water was added to the stirred solution of dendralene **1f/1g** in methanol at 0 °C.[7] Then, the reaction mixture was stirred at room temperature. When TLC indicated completion, 5% HCl (20 mL) was added and the resulting solution was extracted with ethyl acetate (20 mL × 2). Subsequently, the combined organic layers were washed with saturated NaCl solution (40 mL), dried over anhydrous Na_2SO_4, filtered and concentrated in vacuo. The residue was purified by chromatography on a column of silica gel using the mobile phase specified below.

Dimethyl (2*Z*,4*E*)-3-(3-hydroxycyclopent-1-en-1-yl)hexa-2,4-dienedioate (12f): Synthesized following the general method, using **1f** (0.5 mmol, 0.125 g), $NaBH_4$ (0.5 mmol, 0.019 g) in H_2O (0.5 mL) and MeOH (5 mL). Reaction time: 60 min. Purification by column chromatography (hx → 60 : 40 hx/EtOAc) gave a mixture, 35% yield (0.045 g). Unfortunately, the compound has been characterized only through proton NMR, due to the impurities. **^{1}H NMR** (600 MHz, $CDCl_3$) δ 7.31–7.22 (m, 1H), 6.14–6.04 (m, 1H), 6.03–5.97 (m, 1H), 5.63–5.60 (m, 1H), 4.93–4.86 (m, 1H), 3.72 (s, 3H), 3.67 (s, 3H), 2.69–2.61 (m, 1H), 2.46–2.43 (m, 2H), 2.41–2.29 (m, 2H).

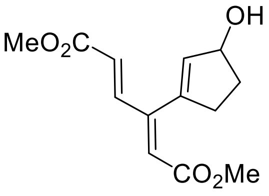

$C_{13}H_{16}O_5$
MW: 252.27

Dimethyl (2*Z*,4*E*)-3-(3-hydroxycyclohex-1-en-1-yl)hexa-2,4-dienedioate (12g): Synthesized following the general method, using **1g** (1 mmol, 0.264 g), $NaBH_4$ (1 mmol, 0.038 g) in H_2O (1 mL) and MeOH (10 mL). Reaction time: 30 min. Purification by column chromatography (hx → 70 : 30 hx/EtOAc) gave the title compound as white amorphous solid, 56% yield (0.150 g). **^{1}H NMR** (600 MHz, $CDCl_3$): δ 7.24 (dd, *J* = 15.6 Hz, *J* = 0.8 Hz, 2H), 6.12 (dd, *J* = 15.6 Hz, *J* = 0.8 Hz, 1H), 5.95 (s, 1H), 5.52–5.45 (m, 1H), 4.30–4.24 (m, 1H), 3.75 (s, 3H), 3.69 (s, 3H), 2.11–1.99 (m, 2H), 1.94–1.81 (m, 2H), 1.75–1.65 (m, 2H). **^{13}C{^{1}H} NMR** (151 MHz, $CDCl_3$): δ 166.7, 165.5, 154.0, 144.6, 137.1, 128.2, 125.5, 124.2, 65.2, 52.0, 51.6, 31.3, 28.7, 19.0. **LRMS** (APCI+): *m/z* $[M+H]^+$ calcd. for $C_{14}H_{19}O_5^+$ 267.3, found 267.2. **HR-MS**: (TOF-ESI+): *m/z* $[M+Na]^+$ calcd. for $C_{14}H_{18}O_5Na^+$ 289.1046, found 289.1055.

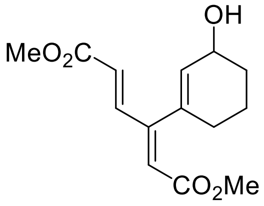

$C_{14}H_{18}O_5$
MW: 266.29

3.2.6 Luche reduction

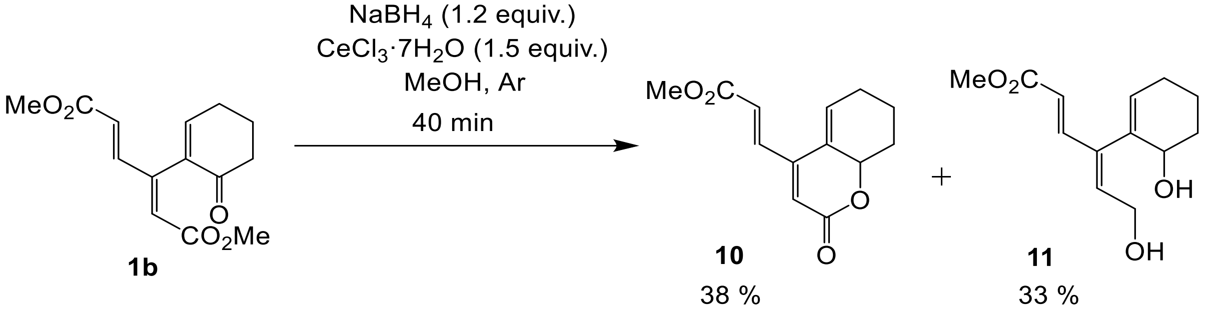

The reaction was performed according to the literature[8]: $CeCl_3 \cdot 7H_2O$ (0.75 mmol, 0.279 g) was added to the stirred solution of dendralene (0.5 mmol, 0.132 g) in methanol (8 mL). After 30 min, $NaBH_4$ (0.6 mmol, 0.023 g) was added, and ($H_2\uparrow$) evolved. After 10 min, that the reaction was stirred at room temperature, the reaction was quenched with 5% HCl (10 ml) and then extracted with ethyl acetate (20 mL × 2). Subsequently, the combined organic layers were washed with saturated NaCl solution (30 mL), dried over anhydrous Na_2SO_4, filtered and concentrated in vacuo. The residue was purified by chromatography on a column of silica gel using mobile phase given below.

Methyl (*E*)-3-(2-oxo-6,7,8,8a-tetrahydro-2*H*-chromen-4-yl)acrylate (10): Synthesized following the general procedure. Purification by column chromatography (hx → 80 : 20 hx/EtOAc) gave the title compound as white amorphous solid, 38% yield (0.044 g). ^{1}**H NMR** (500 MHz, $CDCl_3$): δ 7.40 (dd, *J* = 15.9 Hz, *J* = 0.8 Hz, 1H), 6.38 (d, *J* = 15.9 Hz, overlap, 1H) 6.38–6.35 (m, 1H, overlap), 6.04 (s, 1H), 5.02–4.96 (m, 1H), 3.81 (s, 3H), 2.35–2.24 (m, 3H), 1.95–1.85 (m, 2H), 1.72–1.61 (m, 1H). ^{13}**C{^{1}H} NMR** (126 MHz, $CDCl_3$): δ 165.9, 164.6, 147.8, 138.4, 135.2, 129.1, 126.0, 116.0, 75.0, 52.1, 28.5, 25.9, 19.5. **IR** (ATR-Ge): ν_{max} [cm^{-1}] 2954, 1720, 1698, 1429, 1195, 1173. **LR-MS** ($APCI^+$): *m/z* $[M+H]^+$ calcd. for $C_{13}H_{15}O_4^+$ 235.3, found 235.0. **HR-MS**: (TOF-ESI+): *m/z* $[M+H]^+$ calcd. for $C_{13}H_{15}O_4^+$ 235.0965, found 235.0970; $[M+Na]^+$ calcd. for $C_{13}H_{14}O_4Na^+$ 257.0784, found 257.0786.

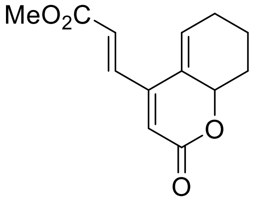

$C_{13}H_{14}O_4$
MW: 234.25

Methyl (2*E*,4*Z*)-6-hydroxy-4-(6-hydroxycyclohex-1-en-1-yl)hexa-2,4-dienoate (11): Synthesized following the general procedure. Purification by column chromatography (hx → 50 : 50 hx/EtOAc) gave the title compound as pale white oil; 33% yield (0.036 g). Using an excess of $NaBH_4$ (4.8 equiv., 2.4 mmol, 0.091 g), the reaction resulted only in compound **11**, 94% yield (0.112 g). ^{1}**H NMR** (600 MHz, $CDCl_3$) δ 7.27 (dd, *J* = 15.7 Hz, *J* = 0.7 Hz, 1H), 6.19–6.14 (m, 1H), 5.74 (d, *J* = 15.6 Hz, 1H), 5.61 (t, *J* = 3.7 Hz, 1H), 4.28 (dd, *J* = 12.7 Hz, *J* = 8.3 Hz, 1H), 4.12–4.04 (m, 1H) 3.99 (dd, *J* = 12.7 Hz, *J* = 5.9 Hz, 1H), 3.73 (s, 3H), 2.96 (s, 2H, OH), 2.22–2.13 (m, 1H), 2.10–2.01 (m, 1H), 1.89–1.75 (m, 3H), 1.67–1.60 (m, 1H). ^{13}**C{^{1}H} NMR** (151 MHz, $CDCl_3$) δ 167.6, 147.3, 141.4, 139.1, 134.4, 131.8, 119.6, 65.4, 58.7, 51.7, 31.7, 25.5, 17.9. **IR** (ATR-Ge): ν_{max} [cm^{-1}] 3496, 3343, 2937, 1716, 1699, 1435, 1194. **LRMS** ($APCI^+$): *m/z* $[M-H_2O+H]^+$ calcd. for $C_{13}H_{17}O_3^+$ 221.3, found 221.2. **HR-MS**: (TOF-ESI+): *m/z* $[M+Na]^+$ calcd. for $C_{13}H_{18}O_4Na^+$ 261.1097, found 261.1102.

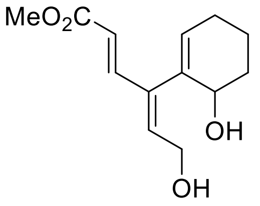

$C_{13}H_{18}O_4$
MW: 238.28

3.3 Thiolates as nucleophiles

3.3.1 Reaction with dendralene **1a**

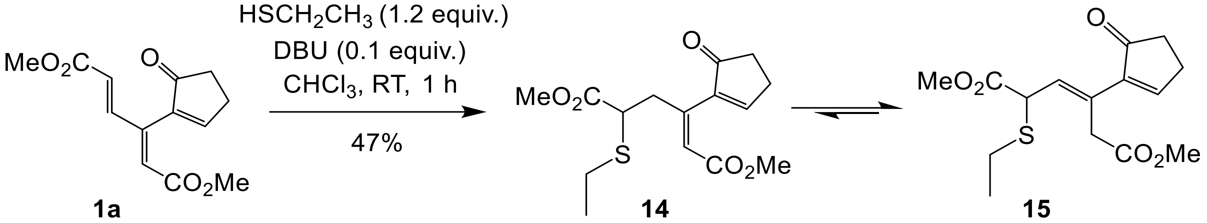

Dendralene **1a** (0.5 mmol, 0.125 g) was dissolved in $CHCl_3$. Ethanethiol (0.6 mmol, 43 µL) and DBU (0.05 mmol, 8 µL) were added subsequently to the stirred solution at RT. Reaction time: 1h. When TLC indicated completion, the reaction was extracted twice with H_2O (20 mL) and DCM (20 mL), the combined organic layers were washed with saturated NaCl solution (20 mL) and dried over Na_2SO_4. The crude NMR analysis proved the formation of unstable major product **14**. Purification by chromatography on a column of silica gel using the mobile phase (hx → 70 : 30 hx/EtOAc) gave the impure product of isomerization **15** as yellowish oil; further precipitation in hexane gave the pure compound **15** as yellowish amorphous precipitate, 47% yield (0.074 g).

Dimethyl (*Z*)-5-(ethylthio)-3-(5-oxocyclopent-1-en-1-yl)hex-2-enedioate (14):

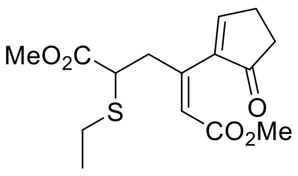

$C_{15}H_{20}O_5S$
MW: 312.38

^{1}H NMR (500 MHz, $CDCl_3$): δ 7.86 (t, J = 3.0 Hz, 1H), δ 6.78 (s, 1H), 3.74 (s, 3H), 3.68 (s, 3H), 3.57 (t, J = 7.7 Hz, 1H), 3.41–3.30 (m, 2H), 2.70–2.66 (m, 2H), 2.62 (qd, J = 7.4 Hz, J = 2.0 Hz, 2H), 2.55–2.53 (m, 2H), 1,21 (t, J = 7.4 Hz, 3H). **^{13}C{^{1}H} NMR** (126 MHz, $CDCl_3$): δ 206.1, 172.7, 166.8, 163.1, 145.6, 142.8, 120.7, 52.2, 51.4, 46.0, 35.9, 32.0, 26.1, 25.8, 14.3.

Dimethyl (*E*)-2-(ethylthio)-4-(5-oxocyclopent-1-en-1-yl)hex-3-enedioate (15):

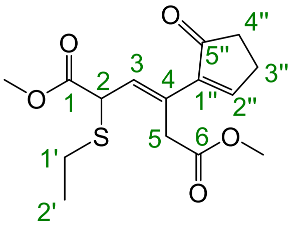

$C_{15}H_{20}O_5S$
MW: 312.38

^{1}H NMR (500 MHz, $CDCl_3$) δ 7.62 (t, J = 3.1 Hz, 1H, H2"), 6.86 (d, J = 9.8 Hz, 1H, H3), 4.30 (d, J = 9.8 Hz, 1H, H2), 3.77 (s, 3H, 1-OCH_3), 3.68 (s, 3H, 6-OCH_3), 3.53 (d, J = 15.9 Hz, 1H, H5), 3.42 (d, J = 15.9 Hz, 1H, H5), δ 2.81–2.65 (m, 2H, H1'), 2.64–2.47 (m, 4H, H3", H4"), 1.26 (t, J = 7.4 Hz, 3H, H2'). **^{13}C{^{1}H} NMR** (126 MHz, $CDCl_3$) δ 207.1 (C5"), 170.9 (C1), 170.8 (C6), 159.2 (C2"), 142.6 (C1"), 128.0 (C3), 127.7 (C4), 52.6 (1-OCH_3), 52.1 (6-OCH_3), 44.3 (C2), 35.9 (C4"), 34.7 (C5), 25.9 (C3"), 25.1 (C1'), 14.3 (C2'). **IR** (ATR-Ge): ν_{max} [cm^{-1}] 2954, 1693, 1436, 1198, 1181. **LRMS** (APCI$^+$): m/z $[M+H]^+$ calcd. for $C_{15}H_{21}O_5S^+$ 313.4, found 313.3. **HRMS** (TOF-ESI+) m/z $[M+H]^+$ calcd. for $C_{15}H_{21}O_5S^+$ 313.1104, found 313.1107; $[M+Na]^+$ calcd. for $C_{15}H_{20}O_5SNa^+$ 335.0924, found 335.0931.

3.3.2 Reaction with dendralenes **1b** and **1c**

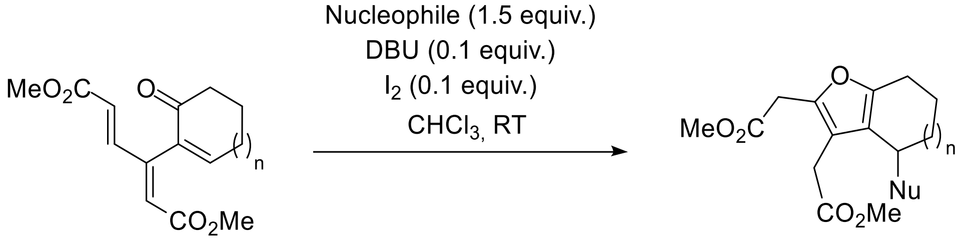

1b: n = 1
1c: n = 2

17b; **18b**
17c; **18c**

General procedure: Nucleophile (1.5 equiv., 0.75 mmol), solution of I_2 (0.1 equiv., 0.05 mmol, 0.013 g in 1 mL of $CHCl_3$) and DBU (0.1 equiv., 0.05 mmol, 8 µL) were added to the solution of dendralene (1.0 equiv., 0.5 mmol) in $CHCl_3$ (8mL/mmol, 4 mL).[9] When TLC indicated completion, the reaction was extracted twice with 10% Na_2SO_3 solution (20 mL) and DCM (20 mL), the combined organic layers were washed with saturated NaCl solution (20 mL) and dried over Na_2SO_4. The solution was evaporated to dryness, and the residue was purified by chromatography on a column of silica gel using mobile phase given below.

Dimethyl 2,2'-(4-(ethylthio)-4,5,6,7-tetrahydrobenzofuran-2,3-diyl)diacetate (17b): Synthesized following the general procedure, using **1b** (0.5 mmol, 0.132 g) and ethanethiol (0.75 mmol, 54 µL) as nucleophile. Reaction time: 1h. Purification by column chromatography (hx → 80 : 20 hx/EtOAc) gave the title compound as yellow oil, 60% yield (0.098 g). **^{1}H NMR** (500 MHz, $CDCl_3$): δ 3.93–3.88 (m, 1H), 3.72 (d, overlap, J = 16.4 Hz, 1H), 3.70 (s, overlap, 3H), 3.69 (s, 3H), 3.62 (s, 2H), 3.41 (d, J = 16.4 Hz, 1H), 2.66–2.45 (m, 4H), 2.12–2.03 (m, 1H), 2.01–1.91 (m, 2H), 1.87–1.80 (m, 1H), 1.29 (t, J = 7.4 Hz, 3H). **^{13}C{^{1}H} NMR** (126 MHz, $CDCl_3$): δ 171.6, 169.8, 151.5, 143.1, 117.9, 114.2, 52.2, 52.0, 37.3, 32.3, 29.6, 29.3, 25.6, 22.9, 18.7, 15.0. **IR** (ATR-Ge): ν_{max} [cm^{-1}] 2951, 1734, 1436, 1170. **LR-MS** (APCI$^-$): m/z [M−H]$^-$ calcd. for $C_{16}H_{21}O_5S^-$ 325.4, found 325.2. **HR-MS** (TOF-ESI+) m/z [M+Na]$^+$ calcd. for $C_{16}H_{22}O_5SNa^+$ 349.1080, found 349.1085.

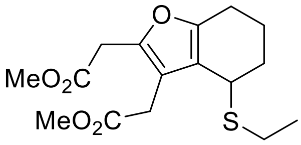

$C_{16}H_{22}O_5S$
MW: 326.41

Dimethyl 2,2'-(4-((2-hydroxyethyl)thio)-4,5,6,7-tetrahydrobenzofuran-2,3-diyl)diacetate (18b): Synthesized following the general procedure, using **1b** (0.5 mmol, 0.132 g) and 2-mercaptoethanol (0.75 mmol, 53 µL) as nucleophile. Reaction time: 5 h. Purification by column chromatography (hx → 60 : 40 hx/EtOAc) gave the title compound as pale oil, 66% yield (0.113 g). **^{1}H NMR** (500 MHz, $CDCl_3$): δ 3.96–3.93 (m, 1H), 3.83–3.70 (m, 2H), 3.69 (s, 3H), 3.68 (s, 3H), 3.66 (d, J = 15.9 Hz, 1H), 3.62 (d, J = 2.4 Hz, 2H), 3.38 (d, J = 15.9 Hz, 1H), 2.80–2.73 (m, 1H), 2.71–2.62 (m, 1H), 2.61–2.54 (m, 1H), 2.53–2.44 (m, 1H), 2.13–2.03 (m, 1H), 1.98–1.90 (m, 2H), 1.86–1.79 (m, 1H). **^{13}C{^{1}H} NMR** (126 MHz, $CDCl_3$): δ 171.9, 169.7, 151.8, 143.4, 117.5, 113.9, 60.9, 52.2, 52.2, 37.3, 34.8, 32.2, 29.6, 29.5, 22.8, 18.3. **IR** (ATR-Ge): ν_{max} [cm^{-1}] 3448, 2950, 1736, 1436, 1256, 1195, 1171. **LR-MS** (APCI$^-$): m/z [M−HSCH$_2$CH$_2$OH−H]$^-$ calcd. for $C_{14}H_{17}O_5^-$ 265.3, found 265.5. **HR-MS** (TOF-ESI$^+$) m/z [M+Na]$^+$ calcd. for $C_{16}H_{22}O_6SNa^+$ 365.1029, found 365.1032.

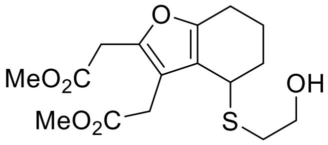

$C_{16}H_{22}O_6S$
MW: 342.41

Dimethyl 2,2'-(4-(ethylthio)-5,6,7,8-tetrahydro-4*H*-cyclohepta[*b*]furan-2,3-diyl)diacetate (17c):

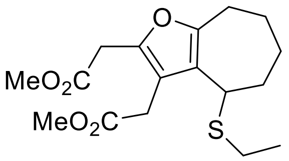

$C_{17}H_{24}O_5S$
MW: 340.43

Synthesized following the general procedure, using **1c** (0.5 mmol, 0.139 g) and ethanethiol (0.75 mmol, 54 µL) as nucleophile. Reaction time: 5h. Purification by column chromatography (hx → 80 : 20 hx/EtOAc) gave the title compound as yellowish oil, 45% yield (0.075 g). **^{1}H NMR** (500 MHz, $CDCl_3$): δ 4.02–3.98 (m, 1H), 3.69 (s, 3H), 3.67 (s, 3H), 3.59 (s, 2H), 3.49 (d, *J* = 16.1 Hz, 1H), 3.37 (d, *J* = 16.1 Hz, 1H), 2.81–2.66 (m, 2H), 2.58–2.40 (m, 2H), 2.19–2.07 (m, 2H), 1.94–1.78 (m, 3H), 1.55–1.43 (m, 1H), 1.25 (t, *J* = 7.4 Hz, 3H). **^{13}C{^{1}H} NMR** (126 MHz, $CDCl_3$): δ 171.5, 169.8, 153.5, 141.5, 121.3, 115.1, 52.2, 52.0, 38.8, 32.3, 31.7, 29.2, 28.3, 25.9, 25.4, 24.1, 14.5. **IR** (ATR-Ge): ν_{max} [cm^{-1}] 2924, 1738, 1435, 1258, 1194, 1168. **LR-MS** (APCI$^-$): *m*/*z* [M−H]$^-$ calcd. for $C_{17}H_{23}O_5S^-$ 339.4, found 339.2. **HR-MS** (TOF-ESI$^+$) *m*/*z* [M+K]$^+$ calcd. for $C_{17}H_{24}O_5SK^+$ 379.0976, found 379.0978.

Dimethyl 2,2'-(4-((2-hydroxyethyl)thio)-5,6,7,8-tetrahydro-4*H*-cyclohepta[*b*]furan-2,3-diyl)diacetate (18c):

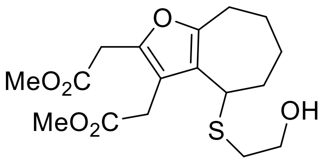

$C_{17}H_{24}O_6S$
MW: 356.43

Synthesized following the general procedure, using **1c** (0.5 mmol, 0.139 g) and 2-mercaptoethanol (0.75 mmol, 53 µL) as nucleophile. Reaction time: 8 h. Purification by column chromatography (hx → 70 : 30 hx/EtOAc) gave the title compound as pale oil, 53% yield (0.095 g). **^{1}H NMR** (500 MHz, $CDCl_3$): δ 4.07 (dd, *J* = 4.6 Hz, *J* = 2.7 Hz, 1H), 3.80–3.72 (m, 2H), 3.71 (s, 3H), 3.69 (s, 3H), 3.62 (d, *J* = 3.2 Hz, 2H), 3.49 (d, *J* = 15.7 Hz, 1H), 3.35 (d, *J* = 15.7 Hz, 1H), 2.78–2.69 (m, 3H), 2.63 (ddd, *J* = 14.0 Hz, *J* = 7.3 Hz, *J* = 5.1 Hz, 1H), 2.23–2.12 (m, 2H), 1.96–1.90 (m, 1H), 1.86 (tt, *J* = 12.6 Hz, *J* = 2.5 Hz, 2H), 1.54–1.44 (m, 1H). **^{13}C{^{1}H} NMR** (126 MHz, $CDCl_3$): 171.8, 169.6, 153.9, 141.8, 121.0, 114.9, 60.6, 52.2, 52.2, 38.6, 34.5, 32.3, 31.6, 29.4, 28.3, 25.8, 24.0. **IR** (ATR-Ge): ν_{max} [cm^{-1}] 3449, 2924, 1737, 1435, 1258, 1196, 1170. **LR-MS** (APCI$^-$): *m*/*z* [M−$HSCH_2CH_2OH$−H]$^-$ calcd. for $C_{15}H_{19}O_5^-$ 279.3, found 279.5. **HR-MS** (TOF-ESI$^+$): *m*/*z* [M+Na]$^+$ calcd. for $C_{17}H_{24}O_6SNa^+$ 379.1186, found 379.1193.

3.3.3 Reaction with dendralene **1i**

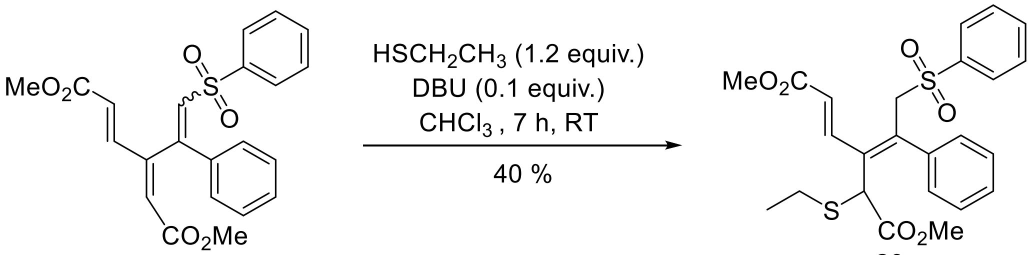

1i **20**

Dimethyl (2*E*,4*E*)-5-(ethylthio)-4-(1-phenyl-2-(phenylsulfonyl)ethylidene)hex-2-enedioate (20):

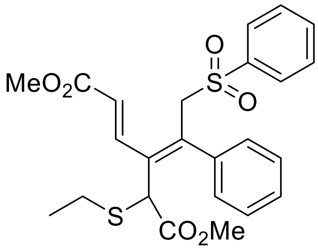

$C_{24}H_{26}O_6S_2$
MW: 474.59

Synthesized following the general procedure without the use of I_2 as catalyst, using **1i** (0.5 mmol, 0.125 g), ethanethiol (0.6 mmol, 43 µL), DBU (0.05 mmol, 8 µL). Reaction time: 7 h. Purification by column chromatography (hx → 80 : 20 hx/EtOAc), gave the title compound as yellow oil, 40% yield (0.094 g). **^{1}H NMR** (500 MHz, $CDCl_3$): δ 7.90–7.83 (m, 2H), 7.61–7.54 (m, 1H), 7.53–7.46 (m, 2H), 7.42–7.31 (m, 5H), 7.10 (d, J = 16.0 Hz, 1H), 6.12 (d, J = 16.0 Hz, 1H), 4.56 (s, 1H), 4.49 (s, 2H), 3.74 (s, 3H), 3.71 (s, 3H), 2.41–2.35 (m, 2H), 0.95 (t, J = 7.4 Hz, 3H). **^{13}C{^{1}H} NMR** (126 MHz, $CDCl_3$): δ 170.4, 166.6, 139.4, 138.2, 137.5, 136.3, 136.1, 133.8, 129.4, 128.7, 128.7, 128.6, 128.5, 122.9, 61.8, 53.1, 51.7, 49.0, 26.6, 13.9. **IR** (ATR-Ge): ν_{max} [cm^{-1}] 2927, 1716, 1308, 1156, 1137. **LR-MS** (APCI$^+$): m/z $[M+H]^+$ calcd. for $C_{24}H_{27}O_6S_2^+$ 475.6, found 475.6; $[M+H-CH_3OH]^+$ calcd. for $C_{23}H_{23}O_5S_2^+$ 443.6, found 443.4. **HR-MS** (TOF-ESI$^+$) m/z $[M+H]^+$ calcd. for $C_{24}H_{27}O_6S_2^+$ 475.1244, found 475.1245.

3.1 Active methylenes as nucleophiles

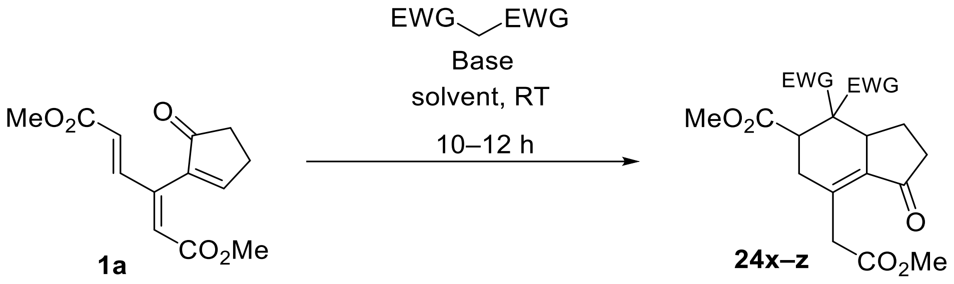

Trimethyl 5-methoxycarbonylmethyl-7-oxobicyclo[4.3.0]non-5-ene-2,2,3-tricarboxylate (24x):

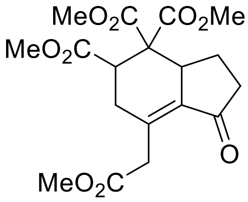

$C_{18}H_{22}O_9$
MW: 382.37

NaOMe (0.5 mmol, 0.027 g) in 1 mL of MeOH, and dimethyl malonate (1.5 mmol, 173 μL) were added to the solution of **1a** (0.5 mmol, 0.125 g) in $CHCl_3$ (4 mL). After 10h, TLC indicated completion, and the reaction was extracted with 5% HCl (20 mL) and DCM (20 mL) twice, the combined organic layers were washed with saturated NaCl solution (20 mL) and dried over Na_2SO_4. The solution was evaporated to dryness, and the residue was purified by chromatography on a column of silica gel using the mobile phase (hx → 80 : 20 hx/EtOAc) to afford the desired product as white crystalline solid, 42% yield (0.08 g), m.p.: 103.5–105 °C (recrystallized from hexane).

Optimized procedure: Dimethyl malonate (1.5 mmol, 173 μL) and a solution of KO*t*Bu (0.25 mmol, 0.03 g) in methanol (500 μL) were added to the solution of **1a** (0.5 mmol, 0.125 g) in DCM (4 mL). After 1h TLC indicated remaining of starting material, so the solution of NaOMe (0.25 mmol, 0.013 g) in methanol (500 μL) was added to the reaction mixture. The reaction showed completion after 10 h, then the reaction was extracted with 5% HCl (20 mL) and DCM (20 mL) twice, the combined organic layers were washed with saturated NaCl solution (20 mL) and dried over Na_2SO_4. The solution was evaporated to dryness, and the residue was purified by chromatography on a column of silica gel using the mobile phase (hx → 80: 20 hx/EtOAc) to afford the desired product, 50% yield (0.096 g). **^{1}H NMR** (500 MHz, $CDCl_3$): δ 3.92 (dd, *J* = 16.2 Hz, *J* = 1.6 Hz, 1H), 3.84 (s, 3H), 3.74 (s, 3H), 3.73 (s, 3H), 3.68 (s, 3H), 3.61 (dd, *J* = 16.2 Hz, *J* = 1.6 Hz, 1H), 3.33–3.26 (m, 1H), 3.23 (dd, *J* = 10.2 Hz, *J* = 7.5 Hz, 1H), 2.83–2.70 (m, 2H), 2.47–2.39 (m, 1H), 2.35–2.11 (m, 3H). **^{13}C{^{1}H} NMR** (126 MHz, $CDCl_3$): δ 204.7, 172.3, 171.1, 170.4, 168.4, 139.8, 132.0, 57.7, 53.0, 52.5, 52.3, 52.0, 46.1, 45.8, 38.5, 35.6, 33.3, 22.2. **IR** (ATR-Ge): ν_{max} [cm^{-1}] 2954, 1728, 1709, 1652, 1436, 1204, 1175. **LR-MS** ($APCI^+$): *m/z* $[M+H]^+$ calcd. for $C_{18}H_{23}O_9^+$ 383.4, found 383.2. **HR-MS** (TOF-ESI^+): *m/z* $[M+Na]^+$ calcd. for $C_{18}H_{22}O_9Na^+$ 405.1156, found 405.1156.

Methyl 2,2-dicyano-5-methoxycarbonylmethyl-7-oxobicyclo[4.3.0]non-5-ene-3-carboxylate (24y):

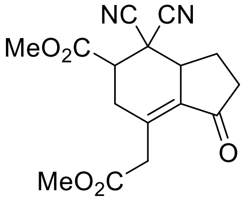

$C_{16}H_{16}N_2O_5$
MW: 316.31

NaOMe (0.5 mmol, 0.027 g) in 1 mL of MeOH, and malononitrile (1.5 mmol, 0.099 g) were added to the solution of **1a** (0.5 mmol, 0.125 g) in $CHCl_3$ (4 mL). After 10 h, TLC indicated completion, and the reaction was extracted with 5% HCl (20 mL) and DCM (20 mL) twice, the combined organic layers were washed with saturated NaCl solution (20 mL) and dried over Na_2SO_4. The solution was evaporated to dryness, and the residue was purified by chromatography on a column of silica gel using the mobile phase (hx → 70 : 30 hx/EtOAc) to afford the desired product as yellow oil, 50% yield (0.079 g).

Optimized procedure: Malononitrile (1.5 mmol, 0.099 g) and a solution of KO*t*Bu (0.25 mmol, 0.03 g) in methanol (500 μL) were added to the solution of **1a** (0.5 mmol, 0.125 g) in DCM (4 mL). After 1 h TLC indicated remaining of starting material, so the solution of NaOMe (0.25 mmol, 0.013 g) in methanol (500 μL) was added to the reaction mixture. The reaction showed completion after 10 h, then the reaction was extracted with 5% HCl (20 mL) and DCM (20 mL) twice, the combined organic layers were washed with saturated NaCl solution (20 mL) and dried over Na_2SO_4. The solution was evaporated to dryness, and the residue was purified by chromatography on a column of silica gel using the mobile phase (hx → 80 : 20 hx/EtOAc) to afford the desired product, 60% yield (0.095 g). **^{1}H NMR** (500 MHz, $CDCl_3$): δ 4.13 (d, *J* = 16.5 Hz, 1H), 3.90 (s, 3H), 3,82–3,75 (m, 1H), 3.72 (s, 3H), 3.49 (d, *J* = 16.5 Hz, 1H), 3.38–3.31 (m, 1H), 3.28 (dd, *J* = 11.6 Hz, *J* = 6.1 Hz, 1H), 3.07 (ddd, *J* = 19.9 Hz, *J* = 6.1 Hz, *J* = 2.5 Hz, 1H), 2.70 (ddd, *J* = 19.9 Hz, *J* = 11.6 Hz, *J* = 4.1 Hz, 1H), 2.62–2.53 (m, 2H), 2.47–2.35 (m, 1H). **^{13}C{^{1}H} NMR** (126 MHz, $CDCl_3$): δ 201.9, 169.7, 168.7, 141.0, 129.8, 114.0, 111.2, 53.4, 52.3, 46.8, 46.5, 37.7, 37.6, 35.4, 32.2, 23.2. **IR** (ATR-Ge): ν_{max} [cm^{-1}] 2956, 1733, 1652, 1233, 1206. **LR-MS** (APCI$^+$): *m*/*z* [M+H]$^+$ calcd. for $C_{16}H_{17}N_2O_5^+$ 317.3, found 317.1. **HR-MS** (TOF-ESI$^+$): *m*/*z* [M+Na]$^+$ calcd. for $C_{16}H_{16}N_2O_5Na^+$ 339.0951, found 339.0955.

Methyl 2-cyano-5-methoxycarbonylmethyl-7-oxo-2-phenylsulfonylbicyclo[4.3.0]non-5-ene-3-carboxylate (24z): (Phenylsulfonyl)acetonitrile (1.5 mmol, 0.271 g) and the solution of NaOMe (0.5 mmol, 0.027 g) in methanol (1 mL) were added to the solution of **1a** (0.5 mmol, 0.125 g) in $CHCl_3$ (4 mL). After 8 h, TLC indicated completion, and the reaction was extracted with 5% HCl (20 mL) and DCM (20 mL) twice, the combined organic layers were washed with saturated NaCl solution (20 mL) and dried over Na_2SO_4. The solution was evaporated to dryness, and the residue was purified by chromatography on a column of silica gel using the mobile phase (90 : 10 → 70 : 30 hx/EtOAc) to afford the desired product as yellow amorphous solid, 50% yield (0.108 g). **^{1}H NMR** (600 MHz, $CDCl_3$): δ 8.08–8.02 (m, 2H), 7.80–7.76 (m, 1H), 7.68–7.63 (m, 2H), 4.14 (d, *J* = 16.3 Hz, 1H), 3,73–3.67 (m, 1H), 3.67 (s, 3H), 3.57 (s, 3H), 3.42–3.37 (m, 1H), 3.34 (d, *J* = 16.3 Hz, 1H), 3.26 (dd, *J* = 11.3 Hz, *J* = 5.7 Hz, 1H), 2.90 (dd, *J* = 13.9 Hz, *J* = 2.4 Hz, 1H), 2.77–2.66 (m, 1H), 2.50–2.39 (m, 2H), 2.33–2.20 (m, 1H). **^{13}C{^{1}H} NMR** (151 MHz, $CDCl_3$): δ 202.7, 170.0 (2× C) 139.7, 135.7 (2 × C), 130.7 (2 ×C), 129.5, 112.7, 67.3, 52.9, 52.3, 44.3, 43.0, 37.8, 35.4, 35.1, 24.4. **IR** (ATR-Ge): ν_{max} [cm^{-1}] 2956, 1737, 1657, 1436, 1155. **LR-MS** (APCI$^+$): *m*/*z* [M+H]$^+$ calcd. for $C_{21}H_{22}NO_7S^+$ 432.5, found 432.2. **HR-MS** (TOF-ESI$^+$): *m*/*z* [M+H]$^+$ calcd. for $C_{21}H_{22}NO_7S^+$ 432.1111, found 432.1113.

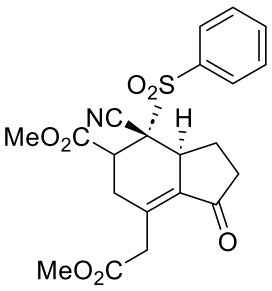

$C_{21}H_{21}NO_7S$
MW: 431.46

3.2 Reaction with nitromethane

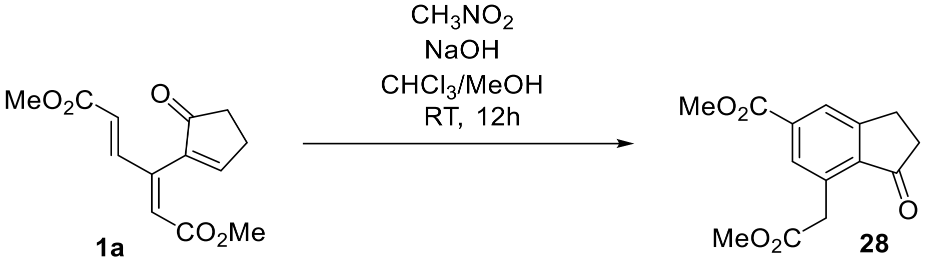

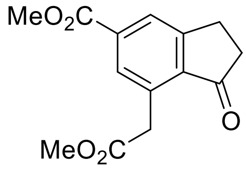

$C_{14}H_{14}O_5$
MW: 262.26

Methyl 7-methoxycarbonylmethyl-1-oxo-2,3-dihydro-1*H*-indene-5-carboxylate (28): Nitromethane (1 mmol, 52 μL) and the solution of NaOH (2 mmol, 0.04 g) in methanol (1 mL) were added to the solution of dendralene **1a** (0.5 mmol, 125 mg) in $CHCl_3$ (5 mL). When TLC indicated completion (approx. 12 h), the reaction was extracted with 5% HCl (20 mL) and DCM (20 mL) twice, the combined organic layers were washed with saturated NaCl solution (20 mL) and dried over Na_2SO_4. The solution was evaporated to dryness, and the residue was purified by chromatography on a column of silica gel using the mobile phase (90 : 10 → 70: 30 hx/EtOAc) to afford the desired product as white amorphous solid, 42% yield (0.055 g). **^{1}H NMR** (600 MHz, $CDCl_3$): δ 8.07–8.06 (m, 1H), 7.83–7.82 (m, 1H), 4.10 (s, 2H), 3.92 (s, 3H), 3.69 (s, 3H), 3.18–3.09 (m, 2H), 2.75–2.67 (m, 2H). **^{13}C{^{1}H} NMR** (151 MHz, $CDCl_3$): δ 206.8, 171.2, 166.2, 155.8, 137.8, 134.9, 133.7, 130.6, 127.0, 52.5, 52.0, 36.9, 36.5, 25.4. **IR** (ATR-Ge): ν_{max} [cm^{-1}] 2957, 1717, 1700, 1435, 1218. **LR-MS** (APCI$^+$): *m/z* $[M+H]^+$ calcd. for $C_{14}H_{15}O_5^+$ 263.3, found 263.1. **HR-MS** (TOF-ESI$^+$) *m/z* $[M+Na]^+$ calcd. for $C_{14}H_{14}O_5Na^+$ 285.0733, found 285.0738.

3.3 Reaction with DBU

General procedure: DBU (0.5 mmol, 74 µL) was added to the reaction mixture of dendralene (0.5 mmol) in $CHCl_3$ (5 mL). When TLC indicated completion, the reaction was extracted with water (20 mL) and DCM (20 mL) twice, the combined organic layers were washed with saturated NaCl solution (20 mL) and dried over Na_2SO_4. The solution was evaporated to dryness, and the residue was purified by chromatography on a column of silica gel using mobile phase given below.

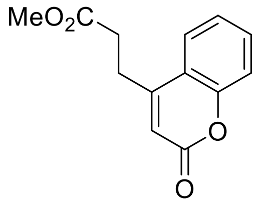

$C_{13}H_{12}O_4$
MW: 232.24

Methyl 3-(2-oxo-2*H*-chromen-4-yl)propanoate (31): Synthesized following the general procedure, using **1b** (0.5 mmol, 0.132 g). Reaction time: 1 h. Purification by column chromatography (hx → 90 : 10 hx/EtOAc) gave the title compound as white crystalline solid, 87% yield (0.101 g); m.p.: 81–83 ºC (recrystallized from hexane). **^{1}H NMR** (500 MHz, $CDCl_3$): δ 7.66 (dd, *J* = 8.0 Hz, *J* = 1.5 Hz, 1H), 7.58–7.51 (m, 1H), 7.37–7.30 (m, 2H), 6.28 (t, *J* = 1.2 Hz, 1H) 3.74 (s, 3H), 3.17–3.12 (m, 2H), 2.80–2.71 (m, 2H). **^{13}C{^{1}H} NMR** (126 MHz, $CDCl_3$): δ 172.1, 160.6, 153.9, 153.6, 131.9, 124.3, 123.9, 118.8, 117.4, 114.0, 52.1, 31.8, 26.4. **IR** (ATR-Ge): ν_{max} [cm^{-1}] 2918, 1731, 1686, 1435, 1271, 1169. **LR-MS** (APCI$^+$): *m*/*z* [M+H]$^+$ calcd. for $C_{13}H_{13}O_4^+$ 233.2, found 233.2. **HR-MS** (TOF-ESI$^+$) *m*/*z* [M+H]$^+$ calcd. for $C_{13}H_{13}O_4^+$ 233.0808, found 233.0818.

Dimethyl 2,2'-(7,8-dihydro-6*H*-cyclohepta[*b*]furan-2,3-diyl)diacetate (33): Synthesized following the general procedure, using **1c** (0.5 mmol, 0.139 g). Reaction time: 3 h. Purification by column chromatography (hx → 80 : 20 hx/EtOAc) gave the title compound as pale oil, 65% yield (0.09 g). **^{1}H NMR** (600 MHz, $CDCl_3$): δ 5.99 (dt, *J* = 11.4 Hz, *J* = 1.6 Hz, 1H), 5.78 (dt, 1H), 3.71 (s, 3H), 3.68 (s, 3H), 3.63 (s, 2H), 3.39 (s, 2H), 2.92 (t, *J* = 6.1 Hz, 2H), 2.41–2.37 (m, 2H), 1.92–1.87 (m, 2H). **^{13}C{^{1}H} NMR** (151 MHz, $CDCl_3$): δ 171.4, 169.9, 153.1, 142.6, 129.5, 118.6, 118.3, 114.6, 52.3, 52.2, 32.4, 29.9, 29.7, 29.1, 21.5. **IR** (ATR-Ge): ν_{max} [cm^{-1}] 2928, 1738, 1435, 1268, 1167. **LR-MS** (APCI$^+$): *m*/*z* [M+H]$^+$ calcd. for $C_{15}H_{19}O_5^+$ 279.3, found 279.1. **HR-MS** (TOF-ESI$^+$) *m*/*z* [M+H]$^+$ calcd. for $C_{15}H_{19}O_5^+$ 279.1227, found 279.1227.

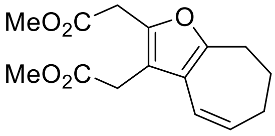

$C_{15}H_{18}O_5$
MW: 278.30

Dimethyl 2,2’-(5-ethyl-4-vinylfuran-2,3-diyl)diacetate (35): Synthesized following the general procedure, using **1d** (0.5 mmol, 0.132 g) and the excess of DBU (0.75 mmol, 111 µL). Reaction time: 48 h. Purification by column chromatography (hx → 80 : 20 hx/EtOAc) gave the title compound as yellowish oil (0.045 g, 34% yield). Optimized procedure: the reaction was warmed in an oil bath at 40 °C for 72 h, reaching the title compound as yellowish oil, 84% yield (0.112 g). **^{1}H NMR** (500 MHz, $CDCl_3$): δ 6.46 (dd, *J* = 17.9 Hz, *J* = 11.6 Hz, 1H, H1'''), 5.31 (dd, *J* = 17.9 Hz, *J* = 1.4 Hz, 1H, H2'''), 5.19 (dd, *J* = 11.6 Hz, *J* = 1.4 Hz, 1H, H2'''), 3.71 (s, 3H, 2'-OCH_3), 3.65 (s, 2H, H1'), 3.64 (s, 3H, 2''-OCH_3), 3.46 (s, 2H, H1''), 2.67 (q, *J* = 7.5 Hz, 2H, H1''''), 1.21 (t, *J* = 7.5 Hz, 3H, H2''''). **^{13}C{^{1}H} NMR** (126 MHz, $CDCl_3$): δ 171.3 (C2''), 169.7 (C2'), 153.8 (C5), 143.1 (C2), 127.1 (C1'''), 118.2 (C4), 114.7 (C2'''), 114.0 (C3), 52.2 (2'-OCH_3), 52.0 (3''-OCH_3), 32.2 (C1'), 29.8 (C2'), 20.1 (C1''''), 12.6 (C2''''). **IR** (ATR-Ge): ν_{max} [cm^{-1}] 2953, 1739, 1436, 1269, 1167. **LR-MS** (APCI$^+$): *m*/*z* [M+H]$^+$ calcd. for $C_{14}H_{19}O_5^+$ 267.3, found 267.2. **HR-MS**: (TOF-ESI$^+$): *m*/*z* [M+H]$^+$ calcd. for $C_{14}H_{19}O_5^+$ 267.1227, found 267.1230; [M+Na]$^+$ calcd. for $C_{14}H_{18}O_5Na^+$ 289.1046, found 289.1053.

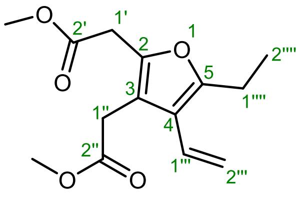

$C_{14}H_{18}O_5$
MW: 266.29

3.4 Deuterium labeling experiments

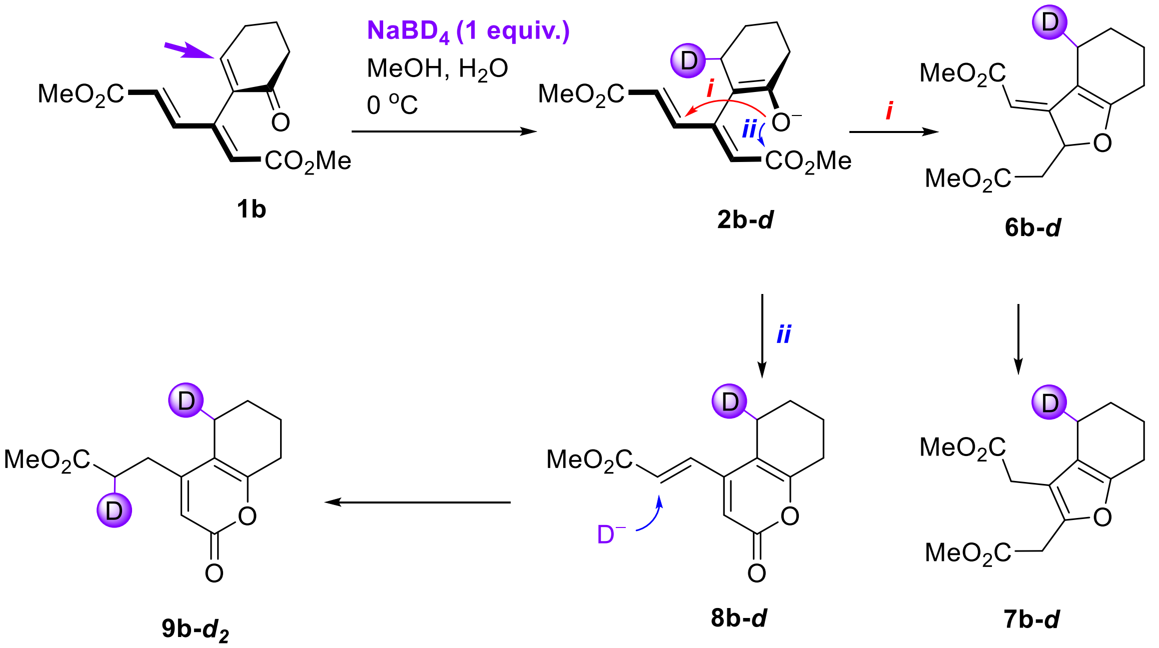

The solution of NaBD$_4$ (0.5 mmol, 0.019 g) in water (0.05 mL) was added to the stirred solution of dendralene **1b** (0.5 mmol, 0.132 g) in methanol (5 mL) at 0 °C. Then, the reaction mixture was stirred at room temperature for 1 h. When TLC indicated completion, 5% HCl (10 mL) was added and the resulting solution was extracted with ethyl acetate (10 mL × 2). Subsequently, the combined organic layers were washed with saturated NaCl solution (20 mL), dried over anhydrous Na$_2$SO$_4$, filtered and concentrated in vacuo. The residue was purified by chromatography on a column of silica gel using the mobile phase (90 : 10 → 70 : 30 hx/EtOAc).

Dimethyl 2,2'-(4,5,6,7-tetrahydrobenzofuran-2,3-diyl-4-*d*)diacetate (7b-*d*): Colorless oil, 50% yield (0.067 g). **^{1}H NMR** (600 MHz, CDCl$_3$): δ 3.69 (s, 3H), 3.66 (s, 3H), 3.62 (s, 2H), 3.31 (s, 2H), 2.52 (dd, *J* = 12.5 Hz, *J* = 1.8 Hz, 2H), 2.31–2.27 (m, 1H), 1.82–1.76 (m, 2H), 1.71–1.67 (m, 2H). **^{13}C{^{1}H} NMR** (151 MHz, CDCl$_3$): δ 171.6, 170.2, 150.1, 142.4, 118.1, 114.5, 52.3, 52.1, 32.5, 29.5, 23.1, 22.9, 22.7, 20.4 (t, *J* = 19.6 Hz). **IR** (ATR-Ge): ν_{max} [cm^{-1}] 2934, 1738, 1435, 1265, 1165. **LR-MS** (APCI$^+$): *m/z* [M+H]$^+$ calcd. for $C_{14}H_{18}DO_5^+$ 268.3, found 268.4. **HR-MS** (TOF-ESI$^+$): *m/z* [M+H]$^+$ calcd. for $C_{14}H_{18}DO_5^+$ 268.1290, found 268.1295.

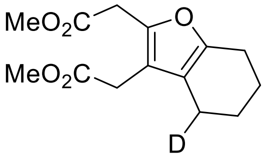

$C_{14}H_{17}DO_5$
MW: 267.30

Methyl (*E*)-3-(2-oxo-5,6,7,8-tetrahydro-2*H*-chromen-4-yl-5-*d*)acrylate (8b-*d*): Yellow amorphous solid, 6% yield (0.007 g). **^{1}H NMR** (600 MHz, CDCl$_3$): δ 7.48 (d, *J* = 15.8 Hz, 1H), 6.39 (d, *J* = 15.8 Hz, 1H), 6.26 (s, 1H), 3.80 (s, 3H), 2.58–2.49 (m, 2H), 2.41–2.38 (m, 1H), 1.83–1.70 (m, 4H). **^{13}C{^{1}H} NMR** (151 MHz, CDCl$_3$): δ 165.9, 162.4, 160.2, 151.3, 138.3, 125.8, 111.2, 109.9, 52.3, 27.8, 23.2 (t, *J* = 18.9 Hz), 21.9, 21.5. **IR** (ATR-Ge): ν_{max} [cm^{-1}] 2951, 1722, 1707, 1432, 1195, 1173. **LR-MS** (APCI$^+$): *m/z* [M+H]$^+$ calcd. for $C_{13}H_{14}DO_4^+$ 236.3, found 236.1. **HR-MS** (TOF-ESI$^+$): *m/z* [M+Na]$^+$ calcd. for $C_{13}H_{13}DO_4Na^+$ 258.0847, found 258.0849.

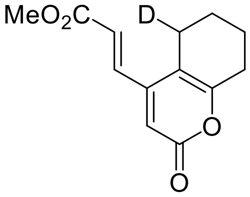

$C_{13}H_{13}DO_4$
MW: 235.26

Methyl 3-(2-oxo-5,6,7,8-tetrahydro-2*H*-chromen-4-yl-5-*d*)propanoate-2-*d* (9b-*d2*): White amorphous solid, 12% yield (0.014 g). **^{1}H NMR** (600 MHz, $CDCl_3$): δ 5.91–5.90 (m, 1H), 3.68 (s, 3H), 2.70–2.63 (m, 2H), 2.58–2.53 (m, 1H), 2.52–2.47 (m, 2H), 2.37–2.30 (m, 1H), 1.81–1.69 (m, 4H). **^{13}C{^{1}H} NMR** (151 MHz, $CDCl_3$): δ 172.4, 162.7, 159.3, 158.4, 112.5, 109.9, 52.1, 31.3 (t, *J* = 19.0 Hz), 27.8, 26.6, 22.5 (t, *J* = 19.0 Hz), 22.0, 21.6. **LR-MS** (APCI$^+$): *m/z* $[M+H]^+$ calcd. for $C_{13}H_{15}D_2O_4^+$ 239.3, found 239.2. **HR-MS** (TOF-ESI$^+$): *m/z* $[M+H]^+$ calcd. for $C_{13}H_{15}D_2O_4^+$ 239.1247, found 239.1248.

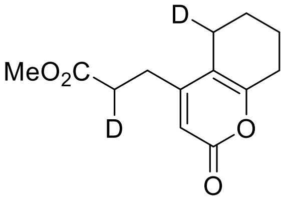

$C_{13}H_{14}D_2O_4$
MW: 238.27

4. X-Ray analysis

The X-ray data for colorless crystals of **(*E*)-1i**, **10**, and **24x** were obtained at 150K using Oxford Cryostream low-temperature device with a Bruker D8-Venture diffractometer equipped with Mo (Mo/K_α radiation; λ = 0.71073 Å) microfocus X-ray (IµS) source, Photon CMOS detector and Oxford Cryosystems cooling device was used for data collection. Obtained data were treated by XT-version 2014/5 and SHELXL-2019/1 (or higher) software implemented in APEX3 v2019.1-0 (Bruker AXS) system.[10] $R_{int} = \sum|F_o^2 - F_{o,mean}^2|/\sum F_o^2$, $S = [\sum(w(F_o^2 - F_c^2)^2)/(N_{diffrs} - N_{params})]^{½}$ for all data, $R(F) = \sum||F_o| - |F_c||/\sum|F_o|$ for observed data, $wR(F^2) = [\sum(w(F_o^2 - F_c^2)^2)/(\sum w(F_o^2)^2)]^{½}$ for all data. Crystallographic data for all structural analyses have been deposited with the Cambridge Crystallographic Data Centre, CCDC nos. 2376772-2376774. Copies of this information may be obtained free of charge from The Director, CCDC, 12 Union Road, Cambridge CB2 1EY, UK (fax: +44-1223-336033; e-mail: deposit@ccdc.cam.ac.uk or www: http://www.ccdc.cam.ac.uk).

The frames for all compounds were integrated with the Bruker SAINT software package using a narrow-frame algorithm. Data were corrected for absorption effects using the Multi-Scan method (SADABS). The structures were solved and refined using the Bruker SHELXTL Software Package. Hydrogen atoms were mostly localized on a difference Fourier map, however to ensure uniformity of treatment of crystal, all hydrogen atoms except of those involved in H-bonds were recalculated into idealized positions (riding model) and assigned temperature factors $H_{iso}(H) = 1.2\ U_{eq}$ (pivot atom) or of $1.5U_{eq}$ (methyl). H atoms in methyl, methylene and methine moieties and C-H in aromatic rings as well as C=C bonds were placed with C-H distances of 0.96, 0.97, 0.98 and 0.93 Å.

Table S1: Experimental details for **(*E*)-1i**.

Crystal data	
Chemical formula	$C_{22}H_{20}O_6S$
M_r	412.44
Crystal system, space group	Triclinic, *P*-1
Temperature (K)	150
a, *b*, *c* (Å)	7.9142(2), 10.5654(2), 13.2909(3)
α, β, γ (°)	73.308(1), 75.715(1), 73.132(1)
V (Å^3)	1002.40(4)
Z	2
Radiation type	Mo*K*α
μ (mm^{-1})	0.20
Crystal size (mm)	0.29 × 0.23 × 0.13
Data collection	
Diffractometer	Bruker D8 - Venture
Absorption correction	Multi-scan *SADABS2016*/2 - Bruker AXS area detector scaling and absorption correction
T_{min}, T_{max}	0.697, 0.746
No. of measured, independent and observed [$I > 2\sigma(I)$] reflections	51693, 4926, 4597
R_{int}	0.026
$(\sin \theta/\lambda)_{max}$ (Å^{-1})	0.668
Refinement	
$R[F^2 > 2\sigma(F^2)]$, $wR(F^2)$, S	0.035, 0.096, 1.05
No. of reflections	4926
No. of parameters	264
No. of restraints	203
H-atom treatment	H-atom parameters constrained
$\Delta\rho_{max}$, $\Delta\rho_{min}$ (e Å^{-3})	0.40, −0.29

Computer programs: Bruker Instrument Service vV6.2.3, *APEX3* v2016.5-0 (Bruker AXS), *SAINT* V8.37A (Bruker AXS Inc., 2015), XT, VERSION 2014/5, *SHELXL2019*/1 (Sheldrick, 2019), *PLATON* (Spek, 2009).

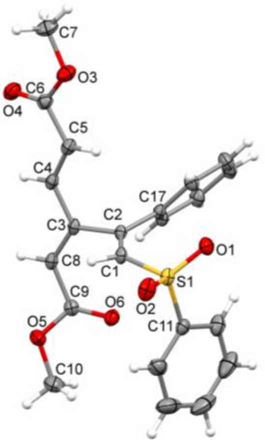

Figure S2. The molecular structure of **(*E*)-1i** (crystal obtained using vapor diffusion technique from hexane / ethyl acetate solvent system), one of the disordered molecules is shown for clarity, ORTEP view 50% probability level. Interatomic distances (Å): C1—C2 1.3394(15), C2—C3 1.5010(14), C9—C8 1.4842(16), C3—C8 1.3416(16), C11—C16 1.3846(18), C3—C4 1.4650(15), C11—C12 1.3861(19), C4—C5 1.3324(17), C12—C13 1.383(2), C5—C6 1.4834(16) document the alternation of single and double bond character of C…C connections.

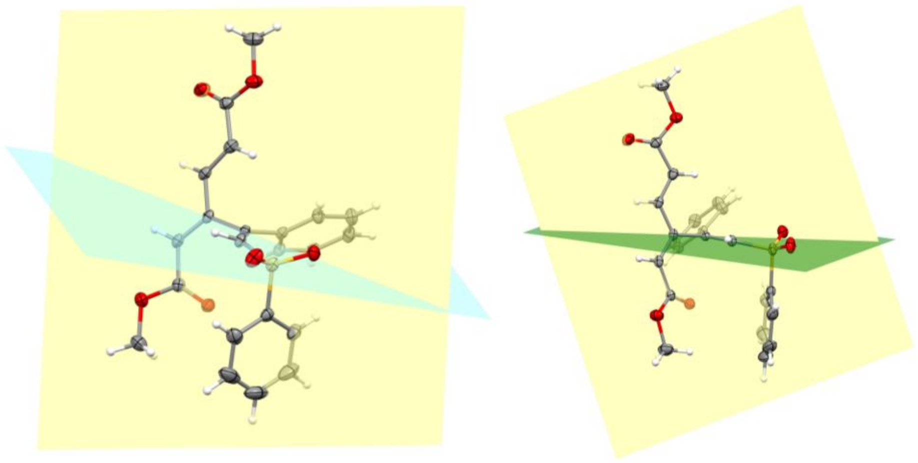

Figure S3. Visualization of planes (C2, C3, C4, C8) vs. (C1, C2, C3, C17) in **(*E*)-1i**, with interplanar angle of 75.09(12)°. The O6 atom of the carbonyl moiety is deviated by 0.441(5)Å from the (C2, C3, C4, C8) plane due a repulsion with the π-electron density of the phenyl ring.

Table S2: Experimental details for **10**

Crystal data	
Chemical formula	$C_{13}H_{14}O_4$
M_r	234.24
Crystal system, space group	Monoclinic, *P*2/*c*
Temperature (K)	150
a, *b*, *c* (Å)	14.8391(10), 11.6782(9), 14.6123(11)
β (°)	112.622(2)
V ($Å^3$)	2337.4(3)
Z	8
Radiation type	Mo*K*α
μ (mm^{-1})	0.10
Crystal size (mm)	0.22 × 0.21 × 0.05
Data collection	
Diffractometer	Bruker D8 - Venture
Absorption correction	Multi-scan<br>*SADABS2016*/2 - Bruker AXS area detector scaling and absorption correction
T_{min}, T_{max}	0.638, 0.746
No. of measured, independent and observed [$I > 2\sigma(I)$] reflections	72984, 4852, 3829
R_{int}	0.077
$(\sin \theta/\lambda)_{max}$ ($Å^{-1}$)	0.628
Refinement	
$R[F^2 > 2\sigma(F^2)]$, $wR(F^2)$, S	0.067, 0.194, 1.06
No. of reflections	4852
No. of parameters	309
No. of restraints	252
H-atom treatment	H-atom parameters constrained
$\Delta\rho_{max}$, $\Delta\rho_{min}$ (e $Å^{-3}$)	0.78, −0.30

Computer programs: Bruker Instrument Service vV6.2.3, *APEX3* v2016.5-0 (Bruker AXS), *SAINT* V8.37A (Bruker AXS Inc., 2015), XT, VERSION 2014/5, *SHELXL2019*/1 (Sheldrick, 2019), *PLATON* (Spek, 2009).

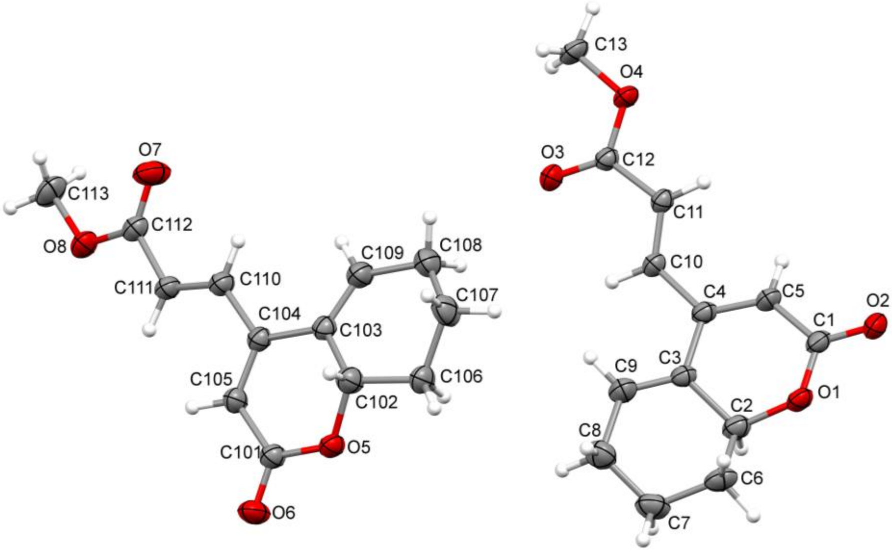

Figure S4. The molecular structure of **10** (crystal obtained using vapor diffusion technique from hexane / ethyl acetate solvent system), ORTEP view of both (*S*)-isomers - 50% probability level. Interatomic distances (Å) document the alternation of single and double bond character of C…C connections, values for one of the molecules are presented, the second exhibits essentially close values: O2—C1 1.208(3), C4—C10 1.476(3), O3—C12 1.206(3), C6—C7 1.516(4), O4—C12 1.335(3), C7—C8 1.500(4), O4—C13 1.445(3), C8—C9 1.496(3), C10—C11 1.319(3), C11—C12 1.480(3), O1—C1 1.341(3), O1—C2 1.452(3), C1—C5 1.464(3), C2—C6 1.499(3), C2—C3 1.513(3), C3—C9 1.322(3), C3—C4 1.466(3), C4—C5 1.343(3).

Table S3: Experimental details for **24x**.

Crystal data	
Chemical formula	$C_{18}H_{22}O_9$
M_r	382.35
Crystal system, space group	Triclinic, *P*-1
Temperature (K)	150
a, *b*, *c* (Å)	8.4915(3), 11.2481(3), 21.0153(6)
α, β, γ (°)	102.559(1), 92.772(1), 108.938(1)
V (Å^3)	1837.72(10)
Z	4
Radiation type	Mo*K*α
μ (mm^{-1})	0.11
Crystal size (mm)	0.59 × 0.33 × 0.23
Data collection	
Diffractometer	Bruker D8 - Venture
Absorption correction	Multi-scan<br>*SADABS2016*/2 - Bruker AXS area detector scaling and absorption correction
T_{min}, T_{max}	0.709, 0.746
No. of measured, independent and observed [$I > 2\sigma(I)$] reflections	50408, 7189, 5477
R_{int}	0.040
$(\sin \theta/\lambda)_{max}$ (Å^{-1})	0.617
Refinement	
$R[F^2 > 2\sigma(F^2)]$, $wR(F^2)$, S	0.051, 0.158, 1.08
No. of reflections	7189
No. of parameters	495
No. of restraints	415
H-atom treatment	H-atom parameters constrained
$\Delta\rho_{max}$, $\Delta\rho_{min}$ (e Å^{-3})	0.78, −0.28

Computer programs: Bruker Instrument Service vV6.2.3, *APEX3* v2016.5-0 (Bruker AXS), *SAINT* V8.37A (Bruker AXS Inc., 2015), XT, VERSION 2014/5, *SHELXL2019*/1 (Sheldrick, 2019), *PLATON* (Spek, 2009).

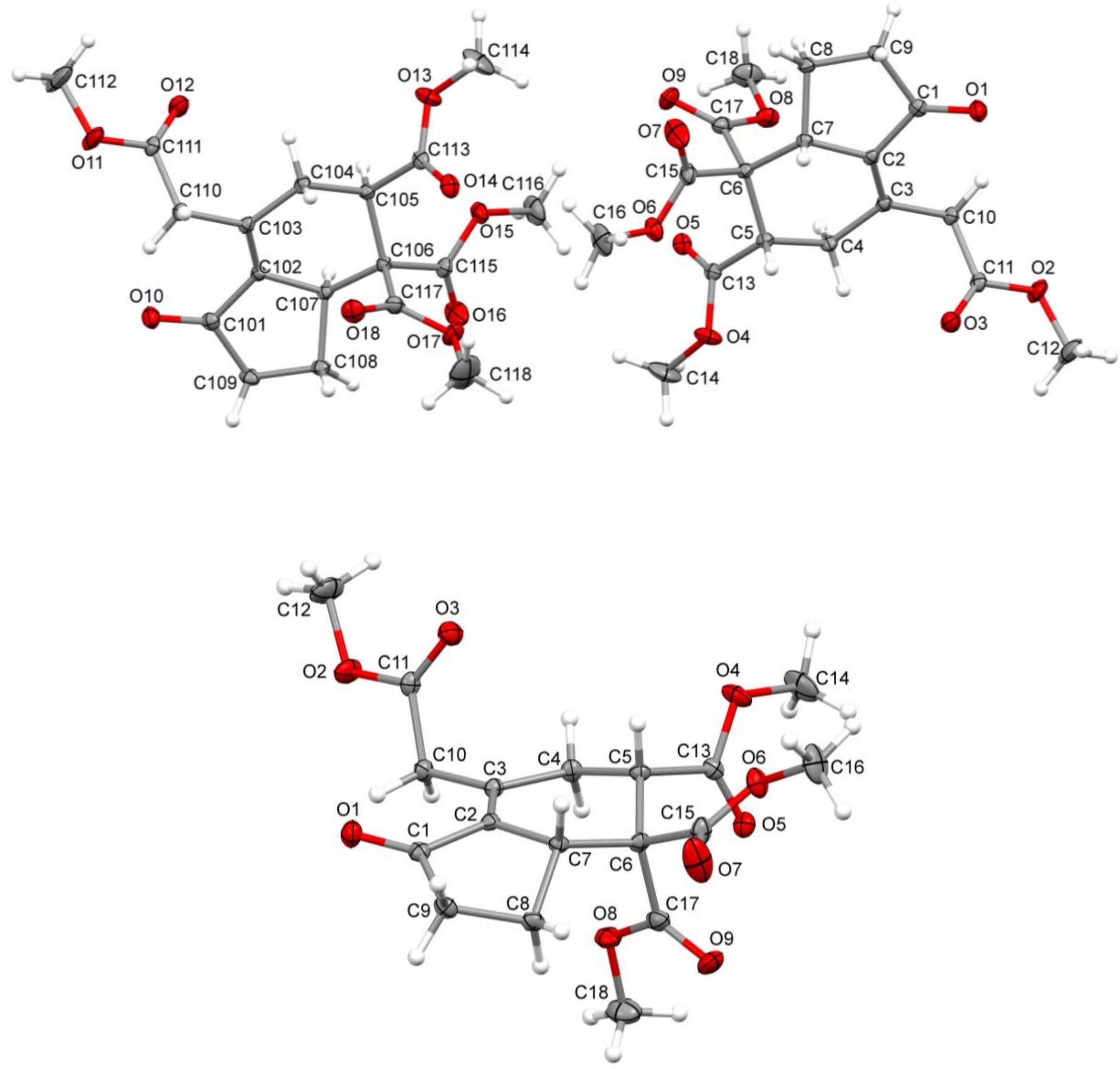

Figure S5. The molecular structure of **24x** (crystal obtained using vapor diffusion technique from hexane / ethyl acetate solvent system), diastereomeric pair (upper part) and one of the isomers (lower part) are shown, ORTEP view 50% probability level. Interatomic distances (Å, the second molecule exhibits nearly the same values): O1—C1 1.222(2), C1—C2 1.485(2), C1—C9 1.514(2), O2—C11 1.326(2), O2—C12 1.446(2), C2—C3 1.341(2), C2—C7 1.510(2), O3—C11 1.201(2), C3—C4 1.496(2), C3—C10 1.503(2), C4—C5 1.538(2), O4—C13 1.334(2), O4—C14 1.445(2), O5—C13 1.202(2), C5—C13 1.511(2), C5—C6 1.553(2), O6—C15 1.326(2), O6—C16 1.451(2), C6—C17 1.524(2), C6—C15 1.541(2), C6—C7 1.552(2), C7—C8 1.542(2), O7—C15 1.194(2), O8—C17 1.330(2), O8—C18 1.449(2), C8—C9 1.532(2).

All studied compounds ***(E)*-1i**, **10** and **24x** crystallize within achiral space groups.

5. Computational details

Our computational study employed Orca 6.0.1,[11] Turbomole7.8,[12] Crest 2.12,[13] and xTB[14] 6.6.1 software. The geometry optimizations of described stationary points were performed at B3LYP[15]-D3BJ/def2-TZVP level of theory using implicit description of chloroform solvent using conductor-like PCM (CPCM)[16] model, as implemented in Orca 6.0.1 (referred as method1). The stationary points were characterized by vibrational frequency calculations (having 0 imaginary frequencies for minima, 1 imaginary frequency for transition-states). The method1-optimized geometry was used for subsequent single point calculations with different computational methods in two kinds of implicit solvent models (CPCM or SMD): B3LYP-D3BJ/def2-TZVPD (with CPCM = method2, with SMD = method 3), M06-2X[17]/def2-TZVPD (with CPCM = method4, with SMD = method5), ωB97M-V[18]/def2-TZVPD (with CPCM = method6, with SMD = method 7) and DLPNO-CCSD(T)[19]/cc-pVTZ with "NormalPNO" settings and SMD solvent model (method8). For the meta-GGA functionals (M06-2X, wB97M-V) we used a dense integration grid by specifying the "DEFGRID3" keyword. For all functionals, we utilized RI and COSX[20] approximations. In addition to CPCM and SMD solvation model, we also report ωB97M-V[18]/def2-TZVPD energies calculated with the COSMO-RS solvation model method. In that case, we used the same geometries from method1 to perform both gas phase and COSMO (ε_{rel} = infinity; FINE cavity) calculation with the BP86 functional and FINE cavity. The gas-phase energy and the out.cosmo file from the COSMO calculation) were then used to calculate COSMO-RS Gibbs free energy of solvation using the COSMOTherm 2023 program (Dassault Systemes BIOVIA) with the BP_TZVPD_FINE_22.ctd parameter file. This energy of solvation was then added to gas-phase energy at ωB97M-V[18]/def2-TZVPD level (still at the same geometry from method1) to obtain the "single point" energy, which we refer to as method9.

For each compound, including the transition states (TSs), we performed conformer sampling using the modified Crest/xTB procedure: geometries were optimized at the GFN-2 in chloroform solvent modeled by the ALPB[21] implicit solvation model (for TSs, we fixed the key bond lengths for each reaction, as determined by initial TS optimization of a random conformer). Then the DFT single point calculations were performed on these geometries with the BP86[22] density functional with dgauss-dzvp basis[23] (as implemented in Turbomole, with RI approximation of the Coulomb integrals and D3-BJ dispersion correction parametrized for accurate conformational energies, parameters: (s6 = 1.0, a1 = 0.7182, s8 = 3.2176, and a2 = 3.8572)[24] with COSMO[25] (ε_{rel} = inf) and FINE cavity[26]. The chloroform solvent was modeled by COSMO-RS implicit solvent model (using external program BIOVIA COSMOtherm 2023 and BP_TZVPD_FINE_22.ctd parameter file). Typically, the 10 most stable conformers from this sampling were then reoptimized by method1 and the lowest energy conformer was taken for the single point calculations with other methods. For all reaction paths, except for protonations and deprotonations, which we assume should have very low barriers, we either found a transition state or we performed a

relaxed reaction coordinate scan to verify that the reaction proceeds without barrier on the electronic energy potential surface (which was the case for most nucleophilic attacks). All calculations were conducted assuming the molecules were in a closed shell singlet spin state, except the Fukui functions, where the anions were calculated as doublets. The IBO analysis[27] was performed using IboView software (version= v20211019-RevA), using method1 wavefunctions as input. The IBOs were generated with exponent = 2 and were visualized with threshold value = 50. The anionic Fukui functions (f^+)[28] were calculated by subtracting the SCF-density (using Chemcraft software) of the method1-CPCM obtained wavefunction of anion and neutral of corresponding dendralenes (**1a**, **1b**, and **1i**):

$f^+(r)=\rho_{(N+1)}(r)-\rho_N(r)$[28]

The f^+ visualizations were using UCSF ChimeraX[29] with SEQCROW plugin[30].

The molar Gibbs free energies of the described stationary points (G), which were used to calculate the reported reaction energies, were calculated as:

$$G_{\text{method } n} = E_{\text{SP,method } n} + E_{\text{ZPVE,method1}} - \mathrm{R}T \ln(q_{\text{trans,method1}}\, q_{\text{rot,method1}}\, q_{\text{vib,method1}}) + \mathrm{R}T, \qquad \text{(Eq. S1)}$$

where the E_{SP} is the electronic energy, which includes the solvation energy, E_{ZPVE} is the zero-point vibrational energy q_{trans}, q_{rot}, q_{vib} are the translational, rotational (rigid rotor) and vibrational (harmonic) partition functions, respectively T is the temperature and R is the gas constant.

Since the ORCA software reports the Gibbs free energies with the 1 atm, 298.15 K standard state, we applied the addition of R(298.15 K) ln((R(298.15 K)/(0.001 m^3))/(101325 Pa)) ~ 1.89 kcal mol^{-1} to convert the energies to the 1 M, 298.15 K standard state.

Note S1. Rotational barriers for atropoisomers of **1b** – the structure corresponding to the 12.8 kcal mol^{-1} value mentioned in the main text is **R6_rot_barrier2_0_1**.

Table S4. Correspondence between structures in Figure S8 and the names used in the main text.

Sulf_SMe1_c80	^{1i}INT2
Sulf_SMe4_c13	^{1i}INT4
Sulf_SMe6	^{1i}INT3
Sulf_SMe4_2	^{1i}INT1
Sulf_SMe1_P2_0_1	^{1i}P2
Sulf_SMe6_P2_0_1	^{1i}P3
Sulf_SMe4_P2_0_1	^{1i}P1
R5	^{1a}R1
R5_SMe1	^{1a}INT1
R5_SMe4	^{1a}INT3
R5_SMe6	^{1a}INT2
R5_SMe6_P2_0_1	^{1a}P2
R5_SMe4_P1SR_0_1	^{1a}P3

R5_SMe1_P2A	^{1a}P1
R5_SMe1_P2A_SMe6S_P2	^{1a}P5
R5_SMe6_cyc4RTS	^{1a}TS1
R5_SMe6_cyc4P2	^{1a}P4
R6_SMe1	^{1b}INT1
R6_SMe4	^{1b}INT3
R6_SMe6	^{1b}INT2
R6_SMe1_P2_0_1	^{1b}P1
R6_SMe6_P2_0_1	^{1b}P2
R6_SMe4_P1SS_0_1	^{1b}P5
R6_SMe6_cyc2SP1	^{1b}INT5
R6_SMe6_cyc4RTS	^{1b}TS1
R6_SMe6_cyc4P2	^{1b}P3
R6_SMe6_cyc2P2	^{1b}P4

In the manuscript the depiction of structures ^{1a}INT1, 1a, ^{1b}INT1, ^{1b}P1, 1i correspond to enantiomers.

Geometry of reactants

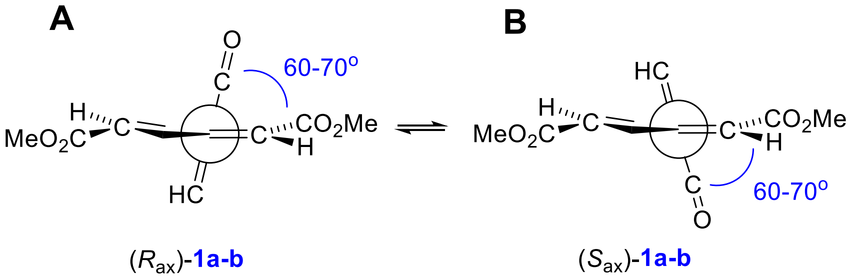

Figure S6. (**A** and **B**) Newman projection of the inseparable atropisomers of **1a**–**1b**.

As a starting point, we focused on the properties of the reactants themselves. The dienoate moiety assumes an approximately planar conformation (being lowest in energy), while the cyclic appendage is bent from planarity by 60–70° (local axially chiral minima in Figure S6A and S4B (also shown in Figure S7), separated by a 13 kcal mol^{-1} barrier for, e.g., **1b**. The situation is similar in the case of **1i**, where the vinylsulfone moiety is roughly perpendicular to the dienoate.

Fukui function

We examined the reactivity of the most stable conformers of **1a**, **1b**, and **1i** using the anionic Fukui function ($f^{+}(r)$)[31]. The $f^{+}(r)$ function is a reactivity descriptor which quantifies the response of the electron density to an increase in the number of electrons: the greater increase of electron density at a reaction site is generally attributed to higher nucleophilic reactivity and vice versa.The calculated $f^{+}(r)$ (Figure S7) shows a general preference for nucleophilic attack at the conjugated sp^{2}carbons (α-δ) over the enone β'-site. However, the variation of $f^{+}(r)$ among the α-δ sites is minimal, meaning that the Fukui function cannot definitively predict the most favorable site of nucleophilic attack. As discussed below, we found that the Fukui function of the most stable conformers of **1a**, **1b**, and **1i** does not correlate with the preferred electrophilic site.

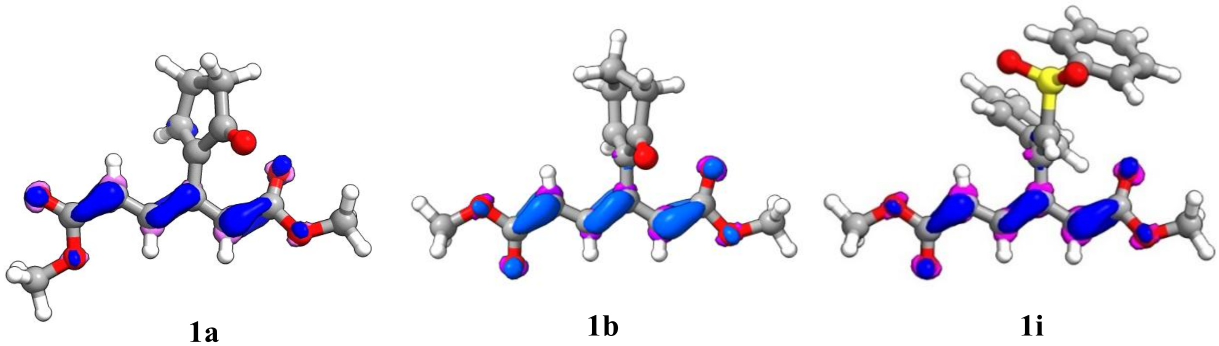

1a **1b** **1i**

Figure S7. The anionic Fukui function (B3LYP-D3BJ/def2-TZVP in chloroform modeled by CPCM implicit solvent model; isovalue 0.0048) depicts the locations of electrophilic sites of dendralenes **1a**, **1b**, and **1i**. Blue regions of increased electron density (as opposed to violet regions of decreased electron density)

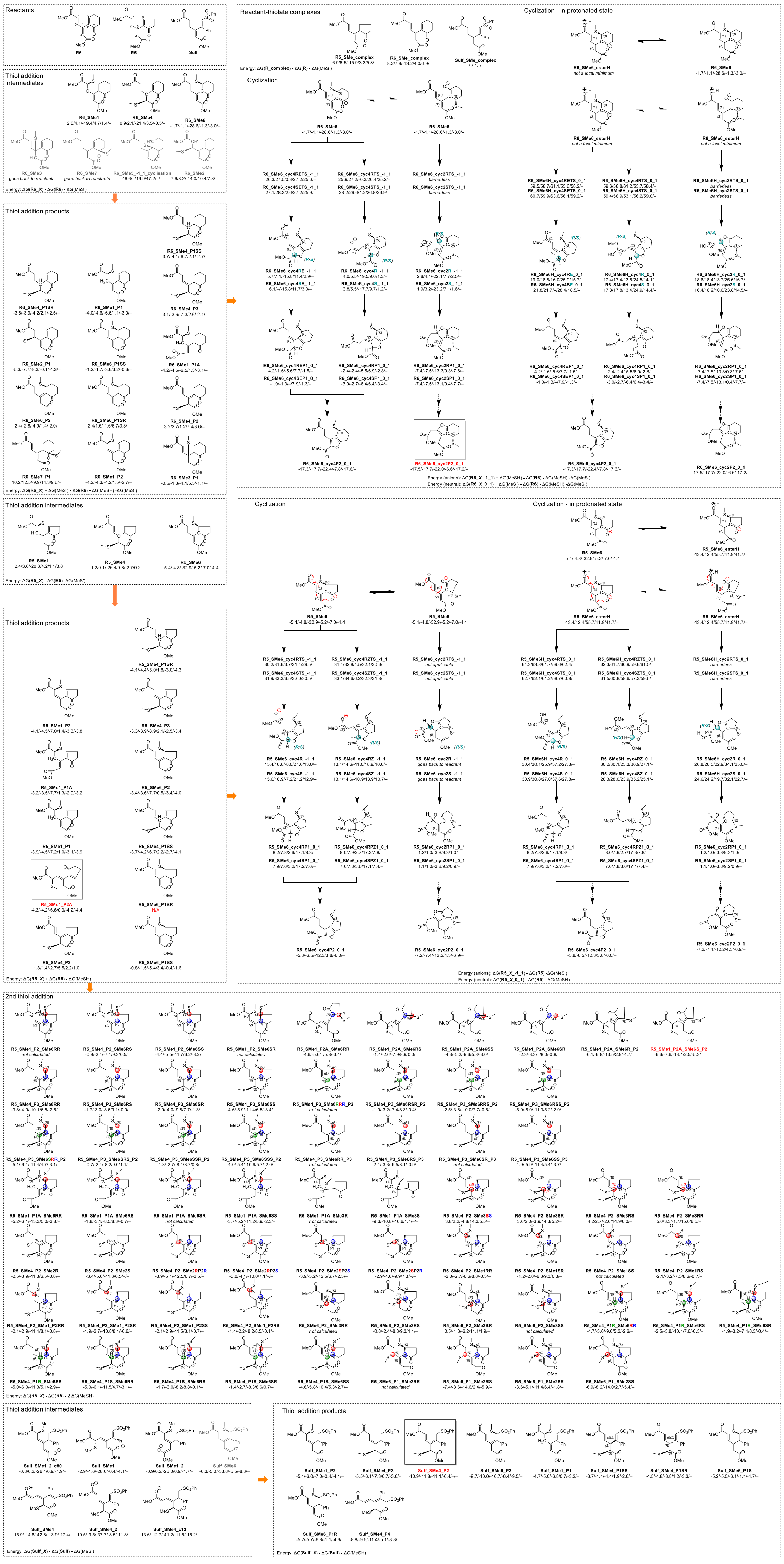

Figure S8. Relative Gibbs free energies of the complete reaction networks of **1a** (= **R5**), 1b (= **R6**) and **1i** (= **Sulf**). The numbers below the name of each structure are Gibbs free energies at 298 K and 1 M, relative to reactants, separated by forward slashes and calculated with method7 method6, method9, method3, method5, and method8, respectively. The naming in this Figure can be cross-referenced to the main text names using Table S4.

XYZ coordinates and energies (in Hartree) of the structures reported in this work

The B3LYP_TZVP_CPCM_chcl3_freq value refers to the non-single point energy contributions from Equation S1.

41
R6_SMe6H_cyc2S_0_1 charge=0 multiplicity=1
B3LYP_D3BJ_TZVPD_SMD_chcl3=-1357.81722728101,
B3LYP_TZVP_CPCM_chcl3_freq=0.2830042,
B3LYP_TZVPD_CPCM_chcl3=-1357.810650658305,
M062X_TZVPD_SMD_chcl3=-1357.923622865139, M062X_TZVPD=-1357.899698076415, COSMORS=-0.006270471064776325,
wB97MV_TZVPD_CPCM_chcl3=-1357.956342868751,
wB97MV_TZVPD_SMD_chcl3=-1357.963105765204, wB97MV_TZVPD=-1357.939387442151, M062X_TZVPD_CPCM_chcl3=-1357.917026670028
O -5.103969000000000 -0.182009000000000 -0.194148000000000
C -3.924510000000000 -0.423912000000000 0.399805000000000
C 0.567507000000000 2.424891000000000 -0.345802000000000
H -4.509948000000000 0.886654000000000 -1.878517000000000
C -1.478257000000000 -0.747547000000000 0.562941000000000
C -2.724645000000000 -0.506034000000000 -0.195936000000000
H -4.756266000000000 -0.865383000000000 -2.129036000000000
O -4.082271000000000 -0.576379000000000 1.729670000000000
C -5.120960000000000 0.023067000000000 -1.611442000000000
H -6.160159000000000 0.205386000000000 -1.869156000000000
H 1.071366000000000 4.822946000000000 -1.401452000000000
H -1.320953000000000 1.866454000000000 0.492191000000000
C -0.284669000000000 0.093697000000000 0.126769000000000
H 2.101042000000000 4.592413000000000 0.028801000000000
C -0.396167000000000 1.444369000000000 0.119515000000000
C 0.764243000000000 -0.839899000000000 -0.174459000000000
C 2.195907000000000 -0.621096000000000 -0.537937000000000
H 0.655810000000000 5.636556000000000 0.134272000000000
S 3.081660000000000 -0.045368000000000 0.981593000000000
C 0.260523000000000 -2.092302000000000 0.000904000000000
H 2.975076000000000 -4.014986000000000 -0.726772000000000
H 2.280112000000000 0.208310000000000 -1.233335000000000
C 0.978096000000000 -3.372131000000000 -0.216868000000000
O -1.017968000000000 -2.145482000000000 0.389111000000000
H 3.892973000000000 -1.768556000000000 -1.218802000000000
C 2.489959000000000 -3.135834000000000 -0.300491000000000
H 0.722224000000000 -4.073299000000000 0.580933000000000
H 0.602706000000000 -3.812958000000000 -1.148016000000000
C 2.812988000000000 -1.893676000000000 -1.128455000000000
H 2.889866000000000 -3.004009000000000 0.707745000000000
H 2.420582000000000 -2.018498000000000 -2.144238000000000
C 4.474914000000000 0.834585000000000 0.222457000000000
H 4.104254000000000 1.640306000000000 -0.409906000000000
H 5.067200000000000 1.254391000000000 1.034735000000000
H 5.101158000000000 0.158556000000000 -0.359041000000000
O 0.237840000000000 3.661016000000000 0.116260000000000
O 1.525017000000000 2.256768000000000 -1.081234000000000
C 1.077284000000000 4.740890000000000 -0.314495000000000
H -1.645186000000000 -0.639849000000000 1.634791000000000
H -2.648113000000000 -0.386055000000000 -1.264670000000000
H -5.006348000000000 -0.398894000000000 1.957786000000000
41
R6_SMe6H_cyc4R_0_1 charge=0 multiplicity=1
B3LYP_D3BJ_TZVPD_SMD_chcl3=-1357.815913056845,
B3LYP_TZVP_CPCM_chcl3_freq=0.28290965,
B3LYP_TZVPD_CPCM_chcl3=-1357.808951971651,
M062X_TZVPD_SMD_chcl3=-1357.924172422968, M062X_TZVPD=-1357.899415008678, COSMORS=-0.004043476799739315,
wB97MV_TZVPD_CPCM_chcl3=-1357.954250341272,
wB97MV_TZVPD_SMD_chcl3=-1357.961405049429, wB97MV_TZVPD=-1357.936986205368, M062X_TZVPD_CPCM_chcl3=-1357.917161818005
O -4.816023000000000 1.143820000000000 -0.199079000000000
C -3.490136000000000 1.358550000000000 -0.123679000000000
C -0.734598000000000 -2.382772000000000 -0.094263000000000
H -4.905527000000000 -0.923061000000000 0.029558000000000
C -1.110561000000000 0.804100000000000 -0.321068000000000
C -2.508230000000000 0.488125000000000 -0.441675000000000
H -6.354869000000000 -0.095867000000000 -0.606135000000000
O -3.230952000000000 2.603594000000000 0.328025000000000
C -5.270037000000000 -0.140610000000000 -0.637889000000000
H -4.932399000000000 -0.337041000000000 -1.656997000000000
H -0.672116000000000 -4.488453000000000 1.526066000000000
H -0.803108000000000 -1.543496000000000 -2.044617000000000
C -0.086637000000000 -0.015123000000000 -0.631918000000000
H -1.050145000000000 -3.024256000000000 2.467904000000000
C -0.176822000000000 -1.442916000000000 -1.157765000000000
C 1.335929000000000 0.255833000000000 -0.577946000000000
C 2.028057000000000 1.489139000000000 -0.115877000000000
H 0.598275000000000 -3.717095000000000 2.521820000000000
S 1.954768000000000 1.546086000000000 1.731024000000000
C 1.994634000000000 -0.796417000000000 -1.088018000000000
H 5.206780000000000 0.262310000000000 -0.857904000000000
H 1.480371000000000 2.373286000000000 -0.450820000000000
C 3.455740000000000 -0.936469000000000 -1.275476000000000
O 1.174806000000000 -1.813084000000000 -1.502189000000000
H 4.031610000000000 2.311337000000000 -0.154517000000000
C 4.176577000000000 0.181569000000000 -0.508048000000000
H 3.784399000000000 -1.921597000000000 -0.932770000000000
H 3.687884000000000 -0.887919000000000 -2.346299000000000
C 3.464277000000000 1.527237000000000 -0.657933000000000
H 4.215978000000000 -0.079827000000000 0.552227000000000
H 3.415381000000000 1.800030000000000 -1.717895000000000
C 2.279872000000000 3.308252000000000 2.017707000000000
H 1.518326000000000 3.921245000000000 1.535166000000000
H 2.236138000000000 3.467248000000000 3.094567000000000
H 3.267763000000000 3.594359000000000 1.658461000000000
O 0.177414000000000 -2.723647000000000 0.811131000000000
O -1.894245000000000 -2.725871000000000 -0.064301000000000
C -0.278308000000000 -3.546532000000000 1.904592000000000
H -0.856244000000000 1.788931000000000 0.053137000000000
H -2.795851000000000 -0.492296000000000 -0.785244000000000
H -4.070108000000000 3.045719000000000 0.521594000000000
37
R5_SMe_complex charge=-1 multiplicity=1 wB97MV_TZVPD_SMD_chcl3=-1318.163493961059, B3LYP_D3BJ_TZVPD=-1317.958504290937,
CCSDT_SMD_chcl3=-1316.252383373766, CCSDT=-1316.177931749193,
B3LYP_TZVP_CPCM_chcl3_freq=0.23670052,
B3LYP_TZVPD_CPCM_chcl3=-1318.030357286723, M062X_TZVPD=-1318.043283122281, COSMORS=-0.00867983707833625,
M062X_TZVPD_CPCM_chcl3=-1318.119087744092,
M062X_TZVPD_SMD_chcl3=-1318.118209440026,
wB97MV_TZVPD_CPCM_chcl3=-1318.164082197552,
B3LYP_D3BJ_TZVPD_SMD_chcl3=-1318.030134088707,
wB97MV_TZVPD=-1318.088586312529
C 1.800270000000000 0.239431000000000 0.883459000000000
C 0.826899000000000 0.992956000000000 0.315723000000000
C -0.538854000000000 0.572801000000000 0.131096000000000
C -1.450918000000000 1.453975000000000 -0.363893000000000
C -0.912054000000000 -0.801644000000000 0.508008000000000
C -0.413747000000000 -1.938804000000000 0.002662000000000
C -1.871740000000000 -1.122794000000000 1.587456000000000
O -2.426967000000000 -0.337634000000000 2.331572000000000
C -1.005593000000000 -3.172472000000000 0.612441000000000
C -2.007602000000000 -2.638084000000000 1.642610000000000
C 4.798975000000000 2.346596000000000 0.642638000000000
O 3.466123000000000 1.851872000000000 0.468505000000000
O 3.916601000000000 0.224737000000000 1.958969000000000
O -3.384739000000000 0.059578000000000 -0.661254000000000
C -4.870871000000000 2.106585000000000 -1.421905000000000
O -3.493602000000000 2.274699000000000 -1.063143000000000
C -2.833388000000000 1.146182000000000 -0.684554000000000
C 3.158612000000000 0.727128000000000 1.148905000000000
H 1.584825000000000 -0.715684000000000 1.332603000000000
H 1.073983000000000 1.984307000000000 -0.042335000000000
H -1.133224000000000 2.466255000000000 -0.576975000000000
H 0.357136000000000 -1.984209000000000 -0.755884000000000
H -1.475332000000000 -3.793786000000000 -0.154916000000000
H -0.222068000000000 -3.785083000000000 1.067432000000000
H -3.040119000000000 -2.900058000000000 1.402584000000000
H -1.811920000000000 -2.983627000000000 2.658527000000000
H 4.986680000000000 2.597615000000000 1.686521000000000
H 4.865350000000000 3.236144000000000 0.021554000000000
H 5.522745000000000 1.600287000000000 0.313931000000000
H -5.229319000000000 3.098585000000000 -1.684412000000000
H -5.440577000000000 1.707453000000000 -0.582698000000000
H -4.967319000000000 1.432067000000000 -2.273006000000000
S 3.137378000000000 -1.281975000000000 -1.148157000000000
C 2.088762000000000 -0.449828000000000 -2.376892000000000
H 1.360405000000000 0.193301000000000 -1.864961000000000
H 2.673378000000000 0.181734000000000 -3.048711000000000
H 1.526859000000000 -1.166003000000000 -2.981249000000000
44
R5_SMe4_P2_SMe3SS charge=0 multiplicity=1 B3LYP_D3BJ_TZVPD=-1757.188216989889, B3LYP_D3BJ_TZVPD_SMD_chcl3=-1757.21286549482, B3LYP_TZVP_CPCM_chcl3_freq=0.30087718,
B3LYP_TZVPD_CPCM_chcl3=-1757.207178954822,
M062X_TZVPD_SMD_chcl3=-1757.338230060303, M062X_TZVPD=-1757.313210953322, COSMORS=-0.004870986218259894,
wB97MV_TZVPD_CPCM_chcl3=-1757.370376366968,
wB97MV_TZVPD_SMD_chcl3=-1757.376245124298, wB97MV_TZVPD=-1757.351349919187, M062X_TZVPD_CPCM_chcl3=-1757.332583336922
C -5.842857000000000 -0.086211000000000 0.462162000000000
C 1.687316000000000 4.277069000000000 -1.262160000000000
C 3.320659000000000 0.675350000000000 2.177192000000000
C -0.464473000000000 1.009546000000000 -2.742644000000000
C 0.709969000000000 -3.998798000000000 0.415316000000000
C -0.188399000000000 -2.755789000000000 0.333265000000000
C 1.936607000000000 -3.616838000000000 -0.414543000000000
C -2.236851000000000 -0.216970000000000 -0.141793000000000
C -1.057031000000000 0.063723000000000 0.402506000000000
C 0.792563000000000 -1.575864000000000 0.400007000000000
C 2.099778000000000 -2.129462000000000 -0.193741000000000
C -3.484353000000000 0.046521000000000 0.595476000000000
C 1.312407000000000 0.902898000000000 0.193406000000000
C 0.905985000000000 2.278875000000000 -0.278242000000000
C 0.299839000000000 -0.214325000000000 -0.179623000000000
O 3.108822000000000 -1.495365000000000 -0.403688000000000
O -3.572675000000000 0.505342000000000 1.715291000000000
O -0.188338000000000 2.768181000000000 -0.119851000000000
O -4.555543000000000 -0.294860000000000 -0.145683000000000
O 1.929687000000000 2.912829000000000 -0.861023000000000
S 1.567258000000000 1.119505000000000 2.015077000000000
S 0.285415000000000 -0.475697000000000 -2.016355000000000
H -5.980828000000000 0.966684000000000 0.704904000000000
H -5.934098000000000 -0.682989000000000 1.369023000000000
H -6.570072000000000 -0.405486000000000 -0.278752000000000
H 0.880784000000000 4.315146000000000 -1.992767000000000
H 2.619257000000000 4.619069000000000 -1.701795000000000
H 1.425645000000000 4.880725000000000 -0.394435000000000
H 3.936334000000000 1.329340000000000 1.562547000000000
H 3.488511000000000 -0.363558000000000 1.906854000000000
H 3.567209000000000 0.829205000000000 3.227219000000000
H -0.748595000000000 0.712279000000000 -3.751621000000000
H 0.242397000000000 1.832894000000000 -2.802956000000000
H -1.351914000000000 1.312351000000000 -2.194080000000000
H 0.211536000000000 -4.895150000000000 0.049002000000000
H 1.004935000000000 -4.180180000000000 1.451875000000000
H -0.929107000000000 -2.718589000000000 1.128842000000000
H -0.722292000000000 -2.745006000000000 -0.618507000000000
H 1.732030000000000 -3.746559000000000 -1.484287000000000
H 2.855943000000000 -4.154076000000000 -0.185033000000000
H -2.328334000000000 -0.644586000000000 -1.129988000000000
H -1.049097000000000 0.487851000000000 1.400988000000000
H 1.029636000000000 -1.383871000000000 1.453024000000000
H 2.278711000000000 0.641626000000000 -0.220207000000000
44
R5_SMe4_P1R_SMe6SS charge=0 multiplicity=1 B3LYP_D3BJ_TZVPD=-1757.200857058494, B3LYP_D3BJ_TZVPD_SMD_chcl3=-1757.22785418843, B3LYP_TZVP_CPCM_chcl3_freq=0.30135417,
B3LYP_TZVPD_CPCM_chcl3=-1757.221235210757,
M062X_TZVPD_SMD_chcl3=-1757.352070643421, M062X_TZVPD=-1757.32512410645, COSMORS=-0.0032194733043815335,
wB97MV_TZVPD_CPCM_chcl3=-1757.384016207119,
wB97MV_TZVPD_SMD_chcl3=-1757.390751143075, wB97MV_TZVPD=-1757.363791316177, M062X_TZVPD_CPCM_chcl3=-1757.345607832261
C -5.924831000000000 0.721498000000000 -0.510088000000000
C 4.815442000000000 1.608312000000000 0.350597000000000
C 2.072790000000000 -2.656805000000000 -2.014682000000000
C 0.309952000000000 2.930458000000000 -1.763557000000000
C -1.223147000000000 0.532322000000000 0.439265000000000
C 0.219831000000000 -2.876916000000000 2.177304000000000
C 0.311370000000000 -3.498551000000000 0.778712000000000
C -2.278608000000000 0.424807000000000 -0.363988000000000
C 0.182736000000000 0.261550000000000 0.002970000000000
C 0.552860000000000 -1.409730000000000 1.983379000000000
C -0.043266000000000 -2.330214000000000 -0.152672000000000
C -3.647748000000000 0.661398000000000 0.130311000000000
C 0.657621000000000 -1.122622000000000 0.487679000000000
C 1.126130000000000 1.375051000000000 0.474679000000000
C 2.525326000000000 1.215554000000000 -0.072302000000000
O 0.705881000000000 -0.600308000000000 2.869767000000000
O -3.955850000000000 0.948736000000000 1.267785000000000
O 2.785309000000000 0.796050000000000 -1.179460000000000
O -4.544613000000000 0.515207000000000 -0.862025000000000
O 3.446958000000000 1.622986000000000 0.804168000000000
S 0.261638000000000 -2.581933000000000 -1.924266000000000
S 0.550977000000000 3.061036000000000 0.028984000000000
H -6.485936000000000 0.561005000000000 -1.426059000000000
H -6.073608000000000 1.736248000000000 -0.142956000000000
H -6.230345000000000 0.009422000000000 0.255565000000000
H 4.929515000000000 2.258858000000000 -0.515314000000000
H 5.114694000000000 0.594110000000000 0.090471000000000
H 5.401087000000000 1.977499000000000 1.186951000000000
H 2.470914000000000 -3.446725000000000 -1.379728000000000
H 2.522843000000000 -1.699180000000000 -1.758941000000000
H 2.306567000000000 -2.889723000000000 -3.052814000000000
H 1.219188000000000 2.592461000000000 -2.256475000000000
H 0.071345000000000 3.938940000000000 -2.099028000000000
H -0.520201000000000 2.268524000000000 -2.002874000000000
H -1.379945000000000 0.796670000000000 1.479750000000000
H 0.866636000000000 -3.328098000000000 2.929768000000000
H -0.805345000000000 -2.916563000000000 2.560799000000000
H -0.357981000000000 -4.344995000000000 0.638323000000000
H 1.330572000000000 -3.836695000000000 0.583409000000000
H -2.171797000000000 0.165830000000000 -1.409956000000000
H 0.215463000000000 0.220189000000000 -1.086233000000000
H -1.123691000000000 -2.169076000000000 -0.116604000000000
H 1.729150000000000 -1.212788000000000 0.271218000000000
H 1.167541000000000 1.401038000000000 1.561320000000000
55
Sulf_SMe1_P1_0_1 charge=0 multiplicity=1 B3LYP_D3BJ_TZVPD=-2138.405838999486, B3LYP_D3BJ_TZVPD_SMD_chcl3=-2138.440770995357, B3LYP_TZVP_CPCM_chcl3_freq=0.3706355,
B3LYP_TZVPD_CPCM_chcl3=-2138.430133346561,
M062X_TZVPD_SMD_chcl3=-2138.592628067585, M062X_TZVPD=-2138.555918607043, COSMORS=-0.008763421473740192,
wB97MV_TZVPD_CPCM_chcl3=-2138.65834328072,
wB97MV_TZVPD_SMD_chcl3=-2138.669357133817, wB97MV_TZVPD=-2138.633375276888, M062X_TZVPD_CPCM_chcl3=-2138.581773172944
C 1.177335000000000 -3.603215000000000 0.707558000000000
C 1.315336000000000 -4.430119000000000 -0.399420000000000
C 0.199188000000000 -4.775925000000000 -1.153195000000000
C -1.062465000000000 -4.305020000000000 -0.799900000000000
C -1.212269000000000 -3.483332000000000 0.306942000000000
C -0.085962000000000 -3.137629000000000 1.045697000000000
O -1.377021000000000 -2.605025000000000 3.261416000000000
O 1.007543000000000 -1.914405000000000 3.092540000000000
S -0.285621000000000 -2.084900000000000 2.458067000000000
C 3.155098000000000 4.359391000000000 0.818640000000000
S 1.353389000000000 4.535001000000000 0.934759000000000
C 1.894503000000000 -0.439549000000000 0.363245000000000
C 2.950866000000000 -0.762818000000000 -0.475871000000000
C 2.730639000000000 -0.948585000000000 -1.835187000000000
C 1.450497000000000 -0.791709000000000 -2.355525000000000
C 0.396616000000000 -0.442134000000000 -1.524201000000000
C 0.606810000000000 -0.283577000000000 -0.152956000000000
C 2.233392000000000 2.656312000000000 -3.099158000000000
O 2.051547000000000 2.610697000000000 -1.671445000000000
O -0.082718000000000 3.272734000000000 -1.925503000000000
O -2.776096000000000 -0.930612000000000 -0.805644000000000
C -5.325663000000000 -0.509205000000000 -1.426527000000000
O -4.464622000000000 0.547459000000000 -0.964310000000000
C -3.196822000000000 0.199356000000000 -0.679620000000000
C 0.840347000000000 2.947329000000000 -1.215419000000000
C -2.438290000000000 1.378801000000000 -0.227149000000000
C -0.886547000000000 -0.540987000000000 1.842506000000000
C -0.518253000000000 0.098632000000000 0.730849000000000
C -1.250507000000000 1.352217000000000 0.384301000000000
C -0.612071000000000 2.643553000000000 0.837871000000000
C 0.796252000000000 2.903797000000000 0.296022000000000
H 2.033476000000000 -3.313189000000000 1.297498000000000
H 2.295252000000000 -4.795799000000000 -0.676403000000000
H 0.311808000000000 -5.413564000000000 -2.020741000000000
H -1.929141000000000 -4.573386000000000 -1.389387000000000
H -2.184200000000000 -3.099849000000000 0.579219000000000
H 3.492418000000000 3.502726000000000 1.400711000000000
H 3.474397000000000 4.260423000000000 -0.216158000000000
H 3.572655000000000 5.272008000000000 1.242476000000000
H 2.063380000000000 -0.303497000000000 1.421485000000000
H 3.945639000000000 -0.875843000000000 -0.064552000000000
H 3.553506000000000 -1.210273000000000 -2.488070000000000
H 1.272887000000000 -0.934155000000000 -3.413638000000000
H -0.597480000000000 -0.318254000000000 -1.927582000000000
H 3.254211000000000 2.329548000000000 -3.271776000000000
H 2.086278000000000 3.671928000000000 -3.463770000000000
H 1.530499000000000 1.983039000000000 -3.586072000000000
H -4.928053000000000 -0.946998000000000 -2.341308000000000
H -6.286980000000000 -0.039974000000000 -1.613502000000000
H -5.418773000000000 -1.281540000000000 -0.663944000000000
H -2.912602000000000 2.333627000000000 -0.411596000000000
H -1.703610000000000 -0.185093000000000 2.457323000000000
H -1.264282000000000 3.472397000000000 0.564096000000000
H -0.537806000000000 2.620832000000000 1.928242000000000
H 1.505294000000000 2.165377000000000 0.658869000000000
44
R5_SMe4_P3_SMe6SR charge=0 multiplicity=1 B3LYP_D3BJ_TZVPD=-1757.197763412798, B3LYP_D3BJ_TZVPD_SMD_chcl3=-1757.223777090172, B3LYP_TZVP_CPCM_chcl3_freq=0.30142261,
B3LYP_TZVPD_CPCM_chcl3=-1757.217215324873,
M062X_TZVPD_SMD_chcl3=-1757.349601531969, M062X_TZVPD=-1757.323339953216, COSMORS=-0.0033256390321567578,
wB97MV_TZVPD_CPCM_chcl3=-1757.380904493746,
wB97MV_TZVPD_SMD_chcl3=-1757.387573021383, wB97MV_TZVPD=-1757.36133976858, M062X_TZVPD_CPCM_chcl3=-1757.343220536061
C -4.935896000000000 -1.445292000000000 1.039815000000000
C 2.956516000000000 -3.215156000000000 -0.933793000000000
C -0.755065000000000 -2.191482000000000 -1.363906000000000
C 2.432148000000000 1.953479000000000 2.671897000000000
C -0.340431000000000 0.031458000000000 1.494386000000000
C -1.036933000000000 2.727593000000000 -1.820864000000000
C -0.449067000000000 1.712000000000000 -2.803238000000000
C -1.779751000000000 0.426336000000000 1.467603000000000
C 0.614097000000000 0.323179000000000 0.609853000000000
C -0.339460000000000 2.447870000000000 -0.505457000000000
C -0.080070000000000 0.487091000000000 -1.941741000000000

C -2.703632000000000 -0.770554000000000 1.422474000000000
C 0.442298000000000 1.143010000000000 -0.636699000000000
C 2.021436000000000 -0.149418000000000 0.913461000000000
C 2.593324000000000 -0.997219000000000 -0.207390000000000
O -0.354885000000000 3.160619000000000 0.471057000000000
O -2.386240000000000 -1.915181000000000 1.648722000000000
O 3.063819000000000 -0.565891000000000 -1.234307000000000
O -3.953005000000000 -0.395012000000000 1.118944000000000
O 2.483081000000000 -2.297122000000000 0.074785000000000
S -1.537627000000000 -0.609583000000000 -1.778552000000000
S 3.184115000000000 1.252403000000000 1.179530000000000
H -5.025404000000000 -1.953907000000000 1.998697000000000
H -4.655848000000000 -2.162521000000000 0.269554000000000
H -5.867932000000000 -0.951781000000000 0.780742000000000
H 2.412656000000000 -3.062796000000000 -1.864189000000000
H 2.763745000000000 -4.205993000000000 -0.534515000000000
H 4.022006000000000 -3.066892000000000 -1.101394000000000
H -0.011648000000000 -2.450277000000000 -2.117203000000000
H -1.549295000000000 -2.936438000000000 -1.373081000000000
H -0.305022000000000 -2.166471000000000 -0.375627000000000
H 1.426740000000000 2.311346000000000 2.459852000000000
H 3.063117000000000 2.791809000000000 2.964225000000000
H 2.412101000000000 1.218673000000000 3.476256000000000
H -0.057415000000000 -0.570446000000000 2.350815000000000
H -2.105420000000000 2.549145000000000 -1.658862000000000
H -0.921178000000000 3.771367000000000 -2.110460000000000
H 0.467233000000000 2.109348000000000 -3.245043000000000
H -1.127910000000000 1.454928000000000 -3.613677000000000
H -2.040248000000000 1.073733000000000 0.638061000000000
H -2.025979000000000 0.977811000000000 2.383620000000000
H 0.698416000000000 -0.108755000000000 -2.411816000000000
H 1.441519000000000 1.518889000000000 -0.904509000000000
H 2.006795000000000 -0.749460000000000 1.820090000000000
41
R6_SMe6H_cyc2R_0_1 charge=0 multiplicity=1 B3LYP_D3BJ_TZVPD_SMD_chcl3=-1357.813917569485, B3LYP_TZVP_CPCM_chcl3_freq=0.28271786, B3LYP_TZVPD_CPCM_chcl3=-1357.807151964809, M062X_TZVPD_SMD_chcl3=-1357.919806045448, M062X_TZVPD=-1357.895032701919, COSMORS=-0.005591023155826388, wB97MV_TZVPD_CPCM_chcl3=-1357.952495091023, wB97MV_TZVPD_SMD_chcl3=-1357.959457388211, wB97MV_TZVPD=-1357.934913240097, M062X_TZVPD_CPCM_chcl3=-1357.91301634565
O -4.416007000000000 -0.473839000000000 -0.446590000000000
C -3.767930000000000 -0.292390000000000 0.710416000000000
C 0.779857000000000 2.442001000000000 -0.481906000000000
H -6.441804000000000 -0.255633000000000 0.030071000000000
C -1.562791000000000 -0.649220000000000 -0.361094000000000
C -2.433243000000000 -0.411705000000000 0.808222000000000
H -5.603770000000000 1.244787000000000 -0.452543000000000
O -4.593634000000000 -0.012664000000000 1.741098000000000
C -5.689652000000000 0.172310000000000 -0.630295000000000
H -5.954562000000000 -0.013346000000000 -1.667413000000000
H 0.895560000000000 5.612876000000000 0.215828000000000
H -1.280232000000000 1.951283000000000 -0.160093000000000
C -0.262317000000000 0.144696000000000 -0.384677000000000
H 1.638346000000000 4.855800000000000 -1.222213000000000
C -0.311438000000000 1.498151000000000 -0.329101000000000
C 0.791805000000000 -0.827527000000000 -0.448478000000000
C 2.274928000000000 -0.666267000000000 -0.391420000000000
H 2.273468000000000 4.491214000000000 0.397070000000000
S 2.727057000000000 -0.176262000000000 1.335793000000000
C 0.212597000000000 -2.060027000000000 -0.459097000000000
H 2.949040000000000 -4.086412000000000 -0.490978000000000
H 2.581425000000000 0.177144000000000 -1.002653000000000
C 0.914317000000000 -3.365498000000000 -0.518853000000000
O -1.123064000000000 -2.064692000000000 -0.417458000000000
H 4.050057000000000 -1.872184000000000 -0.623838000000000
C 2.399279000000000 -3.197514000000000 -0.178635000000000
H 0.423191000000000 -4.072266000000000 0.154100000000000
H 0.792805000000000 -3.767352000000000 -1.531722000000000
C 2.983288000000000 -1.949825000000000 -0.838552000000000
H 2.513277000000000 -3.111457000000000 0.904582000000000
H 2.881987000000000 -2.029159000000000 -1.926997000000000
C 4.305722000000000 0.661176000000000 1.022795000000000
H 4.150873000000000 1.504709000000000 0.351273000000000
H 4.670225000000000 1.024045000000000 1.983173000000000
H 5.040226000000000 -0.024597000000000 0.601517000000000
O 0.405397000000000 3.669476000000000 -0.030154000000000
O 1.879303000000000 2.255056000000000 -0.974351000000000
C 1.373079000000000 4.717703000000000 -0.173966000000000
H -2.112850000000000 -0.490967000000000 -1.288393000000000
H -1.972230000000000 -0.334612000000000 1.784073000000000
H -4.074357000000000 0.156329000000000 2.539293000000000
44
R5_SMe4_P2_SMe1_P2SS charge=0 multiplicity=1 B3LYP_D3BJ_TZVPD=-1757.199578675031, B3LYP_D3BJ_TZVPD_SMD_chcl3=-1757.223459148923, B3LYP_TZVP_CPCM_chcl3_freq=0.30164622, B3LYP_TZVPD_CPCM_chcl3=-1757.216399922642, M062X_TZVPD_SMD_chcl3=-1757.348880970955, M062X_TZVPD=-1757.324235237458, COSMORS=-0.005631548440385439, wB97MV_TZVPD_CPCM_chcl3=-1757.379262848401, wB97MV_TZVPD_SMD_chcl3=-1757.386473636134, wB97MV_TZVPD=-1757.362047076444, M062X_TZVPD_CPCM_chcl3=-1757.341904582946
C 2.438195000000000 -1.319024000000000 3.050700000000000
C -4.102928000000000 0.906458000000000 1.817555000000000
C 4.376208000000000 -0.032892000000000 -1.604810000000000
C -2.144859000000000 3.546635000000000 -0.793826000000000
C -1.690043000000000 -3.323203000000000 -1.744551000000000
C -1.209067000000000 -1.913344000000000 -2.131104000000000
C -2.155256000000000 -3.182749000000000 -0.290279000000000
C 1.148618000000000 0.462033000000000 -1.223386000000000
C -0.535263000000000 -1.403557000000000 -0.841346000000000
C -0.240179000000000 0.070636000000000 -0.777584000000000
C -1.330279000000000 -2.051272000000000 0.288384000000000
C 2.098744000000000 0.689154000000000 -0.039285000000000
C -1.110157000000000 0.998817000000000 -0.334576000000000
C 2.275102000000000 -0.535092000000000 0.830642000000000
C -2.480178000000000 0.631984000000000 0.129961000000000
O -1.273120000000000 -1.739194000000000 1.456818000000000
O 2.438348000000000 -1.659068000000000 0.407150000000000
O -3.232975000000000 -0.099505000000000 -0.477112000000000
O 2.250698000000000 -0.226239000000000 2.128400000000000
O -2.797443000000000 1.211449000000000 1.289039000000000
S 3.759396000000000 1.292482000000000 -0.529834000000000
S -0.651103000000000 2.705068000000000 -0.190602000000000
H 3.405675000000000 -1.789958000000000 2.883505000000000
H 1.642858000000000 -2.051911000000000 2.924338000000000
H 2.394085000000000 -0.872950000000000 4.039508000000000
H -4.179297000000000 1.473502000000000 2.740264000000000
H -4.185064000000000 -0.161143000000000 2.014533000000000
H -4.874841000000000 1.211624000000000 1.112610000000000
H 3.782417000000000 -0.119632000000000 -2.512631000000000
H 4.401298000000000 -0.983769000000000 -1.077270000000000
H 5.391083000000000 0.261413000000000 -1.870261000000000
H -2.382173000000000 3.226274000000000 -1.806378000000000
H -1.901241000000000 4.608115000000000 -0.796938000000000
H -2.988218000000000 3.374948000000000 -0.128074000000000
H -0.856435000000000 -4.027064000000000 -1.802361000000000
H -2.476975000000000 -3.685377000000000 -2.405055000000000
H -2.054918000000000 -1.272375000000000 -2.375477000000000
H -0.521874000000000 -1.920096000000000 -2.976546000000000
H -2.063946000000000 -4.083743000000000 0.316343000000000
H -3.197556000000000 -2.852851000000000 -0.239417000000000
H 1.111011000000000 1.393599000000000 -1.788079000000000
H 1.549868000000000 -0.317730000000000 -1.868758000000000
H 0.427627000000000 -1.925051000000000 -0.768975000000000
H 1.728095000000000 1.500059000000000 0.585432000000000
41
R6_SMe1_P1_0_1 charge=0 multiplicity=1 B3LYP_D3BJ_TZVPD=-1357.825604848998, B3LYP_D3BJ_TZVPD_SMD_chcl3=-1357.850552075588, B3LYP_TZVP_CPCM_chcl3_freq=0.28024053, B3LYP_TZVPD_CPCM_chcl3=-1357.844396169238, M062X_TZVPD_SMD_chcl3=-1357.948731538461, M062X_TZVPD=-1357.923487795292, COSMORS=-0.0025899369924583158, wB97MV_TZVPD_CPCM_chcl3=-1357.986606195234, wB97MV_TZVPD_SMD_chcl3=-1357.992925968779, wB97MV_TZVPD=-1357.967790561315, M062X_TZVPD_CPCM_chcl3=-1357.942644563996
O 4.039057000000000 -0.547734000000000 -0.275092000000000
C 3.199940000000000 0.465051000000000 -0.520655000000000
O 3.529324000000000 1.498349000000000 -1.058378000000000
H 5.508875000000000 -0.156865000000000 -1.702772000000000
H 5.822381000000000 0.513608000000000 -0.082930000000000
C 1.807958000000000 0.169343000000000 -0.013740000000000
C 0.733769000000000 0.763571000000000 -0.924701000000000
C 5.419638000000000 -0.336137000000000 -0.632412000000000
H 5.934260000000000 -1.250678000000000 -0.353470000000000
H 1.689515000000000 -0.909749000000000 0.067987000000000
S 1.711892000000000 0.721720000000000 1.735637000000000
H 0.865542000000000 0.325259000000000 -1.918698000000000
C -0.670760000000000 0.467201000000000 -0.470588000000000
H 0.879294000000000 1.837037000000000 -1.025712000000000
C 1.792653000000000 2.527593000000000 1.583277000000000
H 1.817610000000000 2.903644000000000 2.605358000000000
H 2.697418000000000 2.836746000000000 1.063785000000000
H 0.913301000000000 2.923735000000000 1.079707000000000
C -1.543170000000000 1.467728000000000 -0.303823000000000
C -0.997177000000000 -0.959950000000000 -0.234729000000000
C -2.971314000000000 1.320027000000000 0.029970000000000
H -1.207940000000000 2.488971000000000 -0.432782000000000
C -1.194885000000000 -1.438513000000000 1.002977000000000
H -1.168395000000000 -0.743207000000000 1.834383000000000
O -0.903359000000000 -1.433496000000000 -2.545252000000000
C -1.461667000000000 -2.871580000000000 1.318538000000000
H -1.154771000000000 -3.891490000000000 -1.986629000000000
H -2.600800000000000 -3.295770000000000 -1.176640000000000
C -1.010412000000000 -3.799406000000000 0.194577000000000
H -0.977369000000000 -3.131483000000000 2.262830000000000
H -2.540726000000000 -2.983550000000000 1.494611000000000
C -1.503657000000000 -3.281389000000000 -1.153164000000000
H 0.082691000000000 -3.844231000000000 0.185899000000000
H -1.373216000000000 -4.813986000000000 0.365508000000000
C -1.092400000000000 -1.850270000000000 -1.411446000000000
O -3.484450000000000 2.519509000000000 0.376868000000000
O -3.639150000000000 0.308784000000000 -0.007750000000000
C -4.883320000000000 2.539414000000000 0.709981000000000
H -5.479848000000000 2.207963000000000 -0.139188000000000
H -5.078942000000000 1.893047000000000 1.564883000000000
H -5.110032000000000 3.573181000000000 0.954527000000000
44
R5_SMe6_P1_SMe2SS charge=0 multiplicity=1 B3LYP_D3BJ_TZVPD=-1757.206096759128, B3LYP_D3BJ_TZVPD_SMD_chcl3=-1757.232013902378, B3LYP_TZVP_CPCM_chcl3_freq=0.30153547, B3LYP_TZVPD_CPCM_chcl3=-1757.225859994056, M062X_TZVPD_SMD_chcl3=-1757.35617538059, M062X_TZVPD=-1757.329906348898, COSMORS=-0.0036601837819594143, wB97MV_TZVPD_CPCM_chcl3=-1757.387674972315, wB97MV_TZVPD_SMD_chcl3=-1757.393971739164, wB97MV_TZVPD=-1757.367837230034, M062X_TZVPD_CPCM_chcl3=-1757.350137121983
C 3.167729000000000 -4.186563000000000 0.868745000000000
C -5.799349000000000 -0.419712000000000 0.786794000000000
C 2.764387000000000 2.069222000000000 2.115027000000000
C -1.506714000000000 -1.205969000000000 -2.446934000000000
C 1.990385000000000 1.942473000000000 -2.400674000000000
C 2.078156000000000 2.811904000000000 -1.141787000000000
C 0.633964000000000 -1.599908000000000 0.424485000000000
C -2.116630000000000 -0.795231000000000 0.788178000000000
C 0.310518000000000 -0.346048000000000 0.072677000000000
C 1.669449000000000 0.547823000000000 -1.896912000000000
C 1.091036000000000 2.141589000000000 -0.175026000000000
C -1.125528000000000 0.099521000000000 0.052072000000000
C 1.990526000000000 -2.165492000000000 0.503584000000000
C -3.460124000000000 -0.117743000000000 0.914292000000000
C 1.330714000000000 0.642972000000000 -0.400126000000000
O 1.728157000000000 -0.471685000000000 -2.539428000000000
O 3.052040000000000 -1.584588000000000 0.391840000000000
O -3.617015000000000 1.038013000000000 1.233954000000000
O 1.915469000000000 -3.487623000000000 0.755539000000000
O -4.467669000000000 -0.959583000000000 0.658429000000000
S 1.151674000000000 2.686552000000000 1.557935000000000
S -1.657518000000000 0.430500000000000 -1.683593000000000
H 3.736595000000000 -4.095326000000000 -0.055696000000000
H 2.905624000000000 -5.224013000000000 1.054858000000000
H 3.751577000000000 -3.785423000000000 1.696325000000000
H -5.970212000000000 -0.078962000000000 1.806859000000000
H -6.468709000000000 -1.237892000000000 0.538760000000000
H -5.934413000000000 0.410570000000000 0.095434000000000
H 2.878988000000000 2.423654000000000 3.138565000000000
H 3.575972000000000 2.471259000000000 1.510615000000000
H 2.799274000000000 0.981001000000000 2.112296000000000
H -1.805884000000000 -1.072007000000000 -3.485826000000000
H -2.174854000000000 -1.925552000000000 -1.977390000000000
H -0.476806000000000 -1.554443000000000 -2.412037000000000
H 2.887669000000000 1.931190000000000 -3.019045000000000
H 1.152885000000000 2.249927000000000 -3.034336000000000
H 1.820526000000000 3.854244000000000 -1.318803000000000
H 3.089185000000000 2.777032000000000 -0.732085000000000
H -0.141645000000000 -2.301074000000000 0.693703000000000
H -2.238442000000000 -1.764286000000000 0.310773000000000
H -1.754740000000000 -0.972116000000000 1.805865000000000
H 0.081713000000000 2.417232000000000 -0.482284000000000
H -1.188136000000000 1.088856000000000 0.504609000000000
H 2.287435000000000 0.379200000000000 0.059570000000000
40
R6_SMe5_-1_1_cyclisation charge=-1 multiplicity=1 wB97MV_TZVPD_SMD_chcl3=-1357.407084364401, B3LYP_D3BJ_TZVPD=-1357.199466684854, B3LYP_TZVP_CPCM_chcl3_freq=0.26507745, B3LYP_TZVPD_CPCM_chcl3=-1357.264321129411, COSMORS=-0.011530790050264757, B3LYP_D3BJ_TZVPD_SMD_chcl3=-1357.266602002321, wB97MV_TZVPD=-1357.33728493726
O 4.879214000000000 -0.691538000000000 0.494037000000000
C 3.918515000000000 0.088393000000000 -0.080805000000000
C -1.449255000000000 -2.284851000000000 -0.098241000000000
H 6.381442000000000 0.731781000000000 0.747482000000000
C 1.484342000000000 0.205479000000000 -0.261357000000000
C 2.593579000000000 -0.441205000000000 0.168611000000000
H 6.846955000000000 -0.990589000000000 0.832892000000000
O 4.205932000000000 1.096894000000000 -0.707481000000000
C 6.232006000000000 -0.258161000000000 0.315544000000000
H 6.492608000000000 -0.227636000000000 -0.742878000000000
H -3.452513000000000 -3.497466000000000 1.175253000000000
H 0.696285000000000 -2.289173000000000 -0.068444000000000
C 0.111561000000000 -0.284321000000000 -0.168465000000000
H -2.414003000000000 -4.899046000000000 1.562317000000000
C -0.144650000000000 -1.606729000000000 -0.110551000000000
C -0.870956000000000 0.848430000000000 -0.067096000000000
C -0.266353000000000 1.888328000000000 0.860407000000000
H -2.861773000000000 -4.547933000000000 -0.132075000000000
S -1.011288000000000 1.615362000000000 -1.745705000000000
C -2.145292000000000 0.653445000000000 0.710438000000000
H -2.415025000000000 2.744957000000000 2.539360000000000
H 0.013133000000000 1.441867000000000 1.811391000000000
C -3.089248000000000 1.843908000000000 0.704873000000000
O -2.329952000000000 -0.297628000000000 1.477921000000000
H -1.025398000000000 3.725177000000000 0.014305000000000
C -2.439882000000000 3.008538000000000 1.477070000000000
H -3.294320000000000 2.157827000000000 -0.320878000000000
H -4.024140000000000 1.533694000000000 1.172469000000000
C -0.992299000000000 3.204445000000000 0.977932000000000
H -3.038791000000000 3.923375000000000 1.379027000000000
H -0.456076000000000 3.863213000000000 1.667828000000000
C -1.860165000000000 0.344162000000000 -2.714909000000000
H -2.855445000000000 0.148572000000000 -2.322488000000000
H -1.935093000000000 0.744885000000000 -3.726189000000000
H -1.293383000000000 -0.584006000000000 -2.729072000000000
O -1.419311000000000 -3.334068000000000 0.760509000000000
O -2.405056000000000 -2.061395000000000 -0.810697000000000
C -2.619630000000000 -4.114512000000000 0.838527000000000
H 1.600272000000000 1.145778000000000 -0.782250000000000
H 2.531702000000000 -1.347530000000000 0.756624000000000
38
R5_SMe6_cyc4RPZ1_0_1 charge=0 multiplicity=1 B3LYP_D3BJ_TZVPD_SMD_chcl3=-1318.520565289961, B3LYP_TZVP_CPCM_chcl3_freq=0.25414149, B3LYP_TZVPD_CPCM_chcl3=-1318.514410366959, M062X_TZVPD_SMD_chcl3=-1318.623381257841, M062X_TZVPD=-1318.600112898583, COSMORS=-0.003367917278297207, wB97MV_TZVPD_CPCM_chcl3=-1318.662855235948, wB97MV_TZVPD_SMD_chcl3=-1318.669169931197, wB97MV_TZVPD=-1318.646006952088, M062X_TZVPD_CPCM_chcl3=-1318.617263596359
C -2.463453000000000 -0.140510000000000 0.201244000000000
C -1.167564000000000 0.589530000000000 0.070512000000000
C -0.064886000000000 0.126588000000000 -0.525381000000000
C 0.137499000000000 -1.254023000000000 -1.176981000000000
C 1.212795000000000 0.769255000000000 -0.723009000000000
C 1.924029000000000 2.009653000000000 -0.321095000000000
C 1.993355000000000 -0.032868000000000 -1.450389000000000
O 1.439446000000000 -1.214158000000000 -1.819482000000000
C 3.248816000000000 1.921075000000000 -1.153544000000000
C 3.356775000000000 0.475833000000000 -1.727742000000000
C -6.034501000000000 0.775484000000000 -0.049299000000000
O -4.803186000000000 0.039779000000000 0.087754000000000
O -3.647328000000000 1.952335000000000 -0.137516000000000
O -0.902251000000000 -2.967257000000000 0.146191000000000
C 1.298628000000000 -3.428310000000000 1.604265000000000
O 1.262671000000000 -2.480914000000000 0.515758000000000
C 0.100648000000000 -2.346477000000000 -0.114608000000000
C -3.669977000000000 0.754982000000000 0.026541000000000
H -2.549434000000000 -0.967047000000000 -0.506155000000000
H -1.127245000000000 1.582157000000000 0.501927000000000
H -0.610238000000000 -1.493021000000000 -1.931268000000000
H 1.373038000000000 2.922834000000000 -0.551111000000000
H 3.190157000000000 2.635624000000000 -1.974363000000000
H 4.114636000000000 2.185663000000000 -0.549967000000000
H 3.602681000000000 0.469559000000000 -2.791711000000000
H 4.112955000000000 -0.127717000000000 -1.216874000000000
H -6.069207000000000 1.277469000000000 -1.015241000000000
H -6.125331000000000 1.511799000000000 0.748134000000000
H -6.825155000000000 0.034521000000000 0.023898000000000
H 2.313457000000000 -3.387462000000000 1.987276000000000
H 0.584036000000000 -3.138514000000000 2.372852000000000
H 1.064344000000000 -4.426600000000000 1.238770000000000
S 2.162080000000000 2.151083000000000 1.503419000000000
C 2.995750000000000 0.583308000000000 1.871719000000000
H 3.971178000000000 0.532635000000000 1.388642000000000
H 3.133753000000000 0.553126000000000 2.951665000000000
H 2.384600000000000 -0.262610000000000 1.562665000000000
H -2.563048000000000 -0.605287000000000 1.190158000000000
5
SMe_-1_1 charge=-1 multiplicity=1 wB97MV_TZVPD_SMD_chcl3=-438.178549703293, B3LYP_D3BJ_TZVPD=-438.081491989237, CCSDT_SMD_chcl3=-437.678703335648, CCSDT=-437.598965867096, B3LYP_TZVP_CPCM_chcl3_freq=0.01197857, B3LYP_TZVPD_CPCM_chcl3=-438.164809743467, M062X_TZVPD=-438.108263297644, COSMORS=0.0001589139353609338, M062X_TZVPD_CPCM_chcl3=-438.193242939456, M062X_TZVPD_SMD_chcl3=-438.188268383211, wB97MV_TZVPD_CPCM_chcl3=-438.183138335647, B3LYP_D3BJ_TZVPD_SMD_chcl3=-438.160305743689, wB97MV_TZVPD=-438.099199482832
C -7.104995000000000 2.710040000000000 -0.078790000000000
S -5.693518000000000 2.266194000000000 1.015097000000000
H -6.854952000000000 3.509215000000000 -0.807009000000000
H -7.469914000000000 1.854849000000000 -0.684529000000000
H -7.988382000000000 3.079822000000000 0.481925000000000
44
R5_SMe4_P3_SMe6SSR_P2 charge=0 multiplicity=1 B3LYP_D3BJ_TZVPD=-1757.195319025526, B3LYP_D3BJ_TZVPD_SMD_chcl3=-1757.221186143641, B3LYP_TZVP_CPCM_chcl3_freq=0.30032016, B3LYP_TZVPD_CPCM_chcl3=-1757.215072753293, M062X_TZVPD_SMD_chcl3=-1757.34514592787, M062X_TZVPD=-1757.319179528283, COSMORS=-0.00348150918877181, wB97MV_TZVPD_CPCM_chcl3=-1757.377627296956,

wB97MV_TZVPD_SMD_chcl3=-1757.383909604008, wB97MV_TZVPD=-1757.357959054923, M062X_TZVPD_CPCM_chcl3=-1757.339129894092
C -5.045073000000000 -2.524747000000000 -0.071743000000000
C 2.021166000000000 -4.231899000000000 -0.243482000000000
C 0.350321000000000 3.092271000000000 -2.648418000000000
C 4.407704000000000 -0.272249000000000 0.542773000000000
C -0.784375000000000 -0.376927000000000 0.391397000000000
C -0.744827000000000 2.469234000000000 2.370497000000000
C -1.523661000000000 2.743736000000000 1.080482000000000
C -1.819563000000000 -0.809554000000000 -0.321104000000000
C 0.417113000000000 0.314520000000000 -0.181272000000000
C 0.502505000000000 1.721121000000000 1.939869000000000
C -0.427630000000000 2.796486000000000 0.001353000000000
C -2.961736000000000 -1.479332000000000 0.329043000000000
C 0.613755000000000 1.732010000000000 0.415161000000000
C 1.661340000000000 -0.565337000000000 0.060354000000000
C 1.495482000000000 -1.946847000000000 -0.534000000000000
O 1.299459000000000 1.198299000000000 2.686532000000000
O -3.088814000000000 -1.673453000000000 1.520344000000000
O 0.977075000000000 -2.184702000000000 -1.599687000000000
O -3.874715000000000 -1.859643000000000 -0.581138000000000
O 2.039938000000000 -2.879594000000000 0.257595000000000
S -1.124662000000000 2.691167000000000 -1.671033000000000
S 3.172545000000000 0.139193000000000 -0.721300000000000
H -4.764183000000000 -3.444343000000000 0.440208000000000
H -5.657060000000000 -2.744034000000000 -0.941698000000000
H -5.579480000000000 -1.872921000000000 0.618226000000000
H 2.501791000000000 -4.831568000000000 0.523539000000000
H 2.572659000000000 -4.291603000000000 -1.180659000000000
H 0.994631000000000 -4.559386000000000 -0.399944000000000
H 0.742169000000000 4.068914000000000 -2.364793000000000
H 0.041014000000000 3.118010000000000 -3.692219000000000
H 1.123450000000000 2.334219000000000 -2.524799000000000
H 4.155135000000000 0.204718000000000 1.488352000000000
H 5.357462000000000 0.118016000000000 0.178981000000000
H 4.486101000000000 -1.350310000000000 0.669887000000000
H -0.793746000000000 -0.545026000000000 1.464871000000000
H -1.290752000000000 1.898889000000000 3.122481000000000
H -0.414468000000000 3.399629000000000 2.844072000000000
H -2.096034000000000 3.668882000000000 1.115475000000000
H -2.216467000000000 1.927236000000000 0.875011000000000
H -1.864528000000000 -0.681215000000000 -1.393982000000000
H 0.274467000000000 0.424262000000000 -1.255812000000000
H 0.066966000000000 3.768587000000000 0.065499000000000
H 1.621598000000000 2.048405000000000 0.147208000000000
H 1.865625000000000 -0.642511000000000 1.125411000000000
44
R5_SMe4_P1S_SMe6SS charge=0 multiplicity=1 B3LYP_D3BJ_TZVPD=-1757.199712174998, B3LYP_D3BJ_TZVPD_SMD_chcl3=-1757.22632889069, B3LYP_TZVP_CPCM_chcl3_freq=0.30009082, B3LYP_TZVPD_CPCM_chcl3=-1757.219965059741, M062X_TZVPD_SMD_chcl3=-1757.350472281414, M062X_TZVPD=-1757.323711763923, COSMORS=-0.00230294530955468, wB97MV_TZVPD_CPCM_chcl3=-1757.382351785982, wB97MV_TZVPD_SMD_chcl3=-1757.388871421019, wB97MV_TZVPD=-1757.362132330409, M062X_TZVPD_CPCM_chcl3=-1757.344228337831
C -5.877906000000000 0.526318000000000 0.205482000000000
C -0.495835000000000 -4.643962000000000 0.900493000000000
C 2.007716000000000 2.371443000000000 3.297664000000000
C 2.279334000000000 -1.824721000000000 -2.437347000000000
C -1.102699000000000 0.065554000000000 0.145127000000000
C 0.716478000000000 3.558867000000000 -1.116313000000000
C 1.554894000000000 3.390452000000000 0.153650000000000
C -2.296540000000000 0.111286000000000 -0.439456000000000
C 0.190238000000000 -0.133566000000000 -0.586721000000000
C 0.533178000000000 2.151880000000000 -1.645203000000000
C 1.146660000000000 2.002808000000000 0.677184000000000
C -3.525549000000000 0.321831000000000 0.350928000000000
C 1.028416000000000 1.154311000000000 -0.598348000000000
C 0.926296000000000 -1.342203000000000 0.022518000000000
C 0.120027000000000 -2.613121000000000 -0.134759000000000
O 0.069499000000000 1.851076000000000 -2.720463000000000
O -3.580975000000000 0.473791000000000 1.553520000000000
O -0.479229000000000 -2.924917000000000 -1.138982000000000
O -4.608948000000000 0.327723000000000 -0.444229000000000
O 0.174306000000000 -3.368967000000000 0.966822000000000
S 2.288751000000000 1.266272000000000 1.885850000000000
S 2.596641000000000 -1.666372000000000 -0.657450000000000
H -5.894244000000000 1.488032000000000 0.716779000000000
H -6.063165000000000 -0.271087000000000 0.924088000000000
H -6.618117000000000 0.503692000000000 -0.588866000000000
H -0.054616000000000 -5.258444000000000 0.116988000000000
H -1.556768000000000 -4.501096000000000 0.701291000000000
H -0.346780000000000 -5.100228000000000 1.874400000000000
H 0.953557000000000 2.374035000000000 3.574487000000000
H 2.338563000000000 3.384954000000000 3.077342000000000
H 2.594376000000000 1.976238000000000 4.125629000000000
H 1.936140000000000 -0.884453000000000 -2.865100000000000
H 1.557960000000000 -2.614284000000000 -2.635439000000000
H 3.239295000000000 -2.089043000000000 -2.879197000000000
H -1.049509000000000 0.175109000000000 1.223928000000000
H 1.149975000000000 4.211915000000000 -1.873454000000000
H -0.283713000000000 3.941817000000000 -0.886032000000000
H 1.379888000000000 4.174945000000000 0.887065000000000
H 2.618987000000000 3.382800000000000 -0.090863000000000
H -2.410314000000000 -0.004074000000000 -1.509609000000000
H -0.035477000000000 -0.364809000000000 -1.627576000000000
H 0.171016000000000 2.079958000000000 1.161731000000000
H 2.045162000000000 0.893213000000000 -0.923685000000000
H 1.121447000000000 -1.174724000000000 1.080388000000000
41
R6_SMe6H_cyc4STS_0_1 charge=0 multiplicity=1 B3LYP_D3BJ_TZVPD=-1357.740885715292, B3LYP_D3BJ_TZVPD_SMD_chcl3=-1357.763733856127, B3LYP_TZVP_CPCM_chcl3_freq=0.28123893, B3LYP_TZVPD_CPCM_chcl3=-1357.757450453238, M062X_TZVPD_SMD_chcl3=-1357.850882767448, M062X_TZVPD=-1357.827276717898, COSMORS=-0.006577255277535384, wB97MV_TZVPD_CPCM_chcl3=-1357.886443736084, wB97MV_TZVPD_SMD_chcl3=-1357.892937600941, wB97MV_TZVPD=-1357.869576160507, M062X_TZVPD_CPCM_chcl3=-1357.844580679602
O 1.855887000000000 -3.671607000000000 -0.405625000000000
C 1.068459000000000 -2.622182000000000 -0.220282000000000
C -3.343829000000000 0.029586000000000 -0.144939000000000
H 2.181660000000000 -4.338455000000000 1.546545000000000
C -0.834096000000000 -1.315741000000000 -1.148098000000000
C 0.156371000000000 -2.311236000000000 -1.210845000000000
H 3.270812000000000 -4.941002000000000 0.265645000000000
O 1.198012000000000 -2.023402000000000 0.943786000000000
C 2.747290000000000 -4.073262000000000 0.654671000000000
H 3.450764000000000 -3.274160000000000 0.882382000000000
H -5.876797000000000 -0.131798000000000 -0.971731000000000
H -1.992760000000000 1.713937000000000 -0.333691000000000
C -0.830346000000000 -0.100319000000000 -0.476069000000000
H -6.269276000000000 1.405850000000000 -0.146042000000000
C -1.995412000000000 0.642859000000000 -0.198187000000000
C 0.307284000000000 0.512052000000000 0.227031000000000
C 1.691038000000000 0.687415000000000 -0.311762000000000
H -5.817513000000000 -0.041215000000000 0.805034000000000
S 1.618634000000000 1.998518000000000 -1.620668000000000
C -0.132249000000000 1.101595000000000 1.403880000000000
H 2.891117000000000 2.305865000000000 2.526500000000000
H 2.027880000000000 -0.203199000000000 -0.846526000000000
C 0.840954000000000 1.644395000000000 2.419091000000000
O -1.390654000000000 1.143585000000000 1.676841000000000
H 3.635499000000000 1.340135000000000 0.370808000000000
C 2.150017000000000 2.083030000000000 1.756428000000000
H 0.371679000000000 2.473342000000000 2.950275000000000
H 1.030301000000000 0.858864000000000 3.158939000000000
C 2.687406000000000 1.019176000000000 0.804436000000000
H 1.978596000000000 3.007496000000000 1.199312000000000
H 2.890623000000000 0.098350000000000 1.361198000000000
C 3.179021000000000 1.693132000000000 -2.497640000000000
H 3.203522000000000 2.370864000000000 -3.350230000000000
H 4.040538000000000 1.894401000000000 -1.861853000000000
H 3.217898000000000 0.665003000000000 -2.858629000000000
O -4.293702000000000 0.974268000000000 -0.187614000000000
O -3.574262000000000 -1.157988000000000 -0.070397000000000
C -5.655751000000000 0.510429000000000 -0.120200000000000
H -1.701220000000000 -1.510298000000000 -1.767036000000000
H 0.108432000000000 -3.031416000000000 -2.016067000000000
H 0.741814000000000 -1.129456000000000 0.940885000000000
44
R5_SMe1_P2A_SMe6S_P2 charge=0 multiplicity=1 B3LYP_D3BJ_TZVPD=-1757.206048708403, B3LYP_D3BJ_TZVPD_SMD_chcl3=-1757.23241514017, B3LYP_TZVP_CPCM_chcl3_freq=0.30169055, B3LYP_TZVPD_CPCM_chcl3=-1757.225758226454, M062X_TZVPD_SMD_chcl3=-1757.356212911107, M062X_TZVPD=-1757.329747712105, COSMORS=-0.003027237162959658, wB97MV_TZVPD_CPCM_chcl3=-1757.386835395374, wB97MV_TZVPD_SMD_chcl3=-1757.393655333847, wB97MV_TZVPD=-1757.367167008991, M062X_TZVPD_CPCM_chcl3=-1757.349650445923
C 1.808718000000000 -3.898238000000000 1.968438000000000
C -4.978253000000000 1.609551000000000 1.206295000000000
C 1.743051000000000 1.087366000000000 -2.905507000000000
C -2.698816000000000 -1.805495000000000 -1.617852000000000
C 2.504186000000000 2.587379000000000 1.457847000000000
C 3.598753000000000 1.732658000000000 0.812355000000000
C -0.663302000000000 -0.480331000000000 0.691526000000000
C 1.115463000000000 -1.520027000000000 -0.791383000000000
C 0.702794000000000 -0.357177000000000 0.067730000000000
C 1.213500000000000 1.824681000000000 1.213509000000000
C 2.873698000000000 0.939265000000000 -0.291089000000000
C -1.721774000000000 0.362296000000000 -0.030271000000000
C 1.164658000000000 -2.838820000000000 -0.045294000000000
C 1.499844000000000 0.707462000000000 0.270423000000000
C -3.034047000000000 0.360899000000000 0.719637000000000
O 0.145569000000000 2.095624000000000 1.731215000000000
O 0.798561000000000 -3.896150000000000 -0.501796000000000
O -3.465660000000000 -0.583941000000000 1.341480000000000
O 1.695990000000000 -2.699565000000000 1.174390000000000
O -3.676853000000000 1.525705000000000 0.592268000000000
S 2.794445000000000 2.034835000000000 -1.777329000000000
S -2.049074000000000 -0.117156000000000 -1.771117000000000
H 2.461319000000000 -4.615996000000000 1.473842000000000
H 0.825155000000000 -4.339699000000000 2.120612000000000
H 2.236404000000000 -3.579466000000000 2.914150000000000
H -5.645476000000000 0.864914000000000 0.774382000000000
H -5.334847000000000 2.612650000000000 0.992789000000000
H -4.898149000000000 1.451447000000000 2.280724000000000
H 1.605637000000000 1.711943000000000 -3.787425000000000
H 0.769880000000000 0.887252000000000 -2.459351000000000
H 2.220251000000000 0.153718000000000 -3.200127000000000
H -1.963102000000000 -2.487040000000000 -1.195401000000000
H -2.926618000000000 -2.118448000000000 -2.636209000000000
H -3.609840000000000 -1.824285000000000 -1.023330000000000
H 2.411590000000000 3.563996000000000 0.976003000000000
H 2.641035000000000 2.762001000000000 2.524926000000000
H 4.000038000000000 1.020754000000000 1.535953000000000
H 4.431298000000000 2.317621000000000 0.422805000000000
H -0.632660000000000 -0.137377000000000 1.725116000000000
H -0.980164000000000 -1.522442000000000 0.705415000000000
H 2.099109000000000 -1.374971000000000 -1.235830000000000
H 0.404506000000000 -1.644170000000000 -1.608692000000000
H 3.407154000000000 0.033010000000000 -0.563398000000000
H -1.376679000000000 1.389631000000000 -0.118622000000000
37
R5_SMe6_cyc4STS_-1_1 charge=-1 multiplicity=1 wB97MV_TZVPD_SMD_chcl3=-1318.126174012384, B3LYP_D3BJ_TZVPD=-1317.916397868648, B3LYP_TZVP_CPCM_chcl3_freq=0.23916934, B3LYP_TZVPD_CPCM_chcl3=-1317.984759368179, M062X_TZVPD=-1318.008143112691, COSMORS=-0.010002637823673699, M062X_TZVPD_CPCM_chcl3=-1318.078970749094, M062X_TZVPD_SMD_chcl3=-1318.08119060258, wB97MV_TZVPD_CPCM_chcl3=-1318.123726565469, B3LYP_D3BJ_TZVPD_SMD_chcl3=-1317.986784909235, wB97MV_TZVPD=-1318.054073160387
C -1.430400000000000 1.666293000000000 -0.228866000000000
C -1.190559000000000 0.314062000000000 -0.008011000000000
C 0.035665000000000 -0.325776000000000 0.098040000000000
C 0.181497000000000 -1.734240000000000 0.383052000000000
C 1.361944000000000 0.140020000000000 -0.271558000000000
C 2.054288000000000 1.435466000000000 -0.484302000000000
C 2.096543000000000 -0.904783000000000 -0.730462000000000
O 1.631582000000000 -2.136977000000000 -0.622206000000000
C 3.267052000000000 1.055037000000000 -1.389966000000000
C 3.360273000000000 -0.492449000000000 -1.416041000000000
C -5.073958000000000 1.921588000000000 -0.169658000000000
O -3.762072000000000 1.369196000000000 -0.107402000000000
O -2.963889000000000 3.453771000000000 -0.485894000000000
O -1.678295000000000 -2.623302000000000 -0.869110000000000
C -1.837194000000000 -4.789666000000000 0.658052000000000
O -0.873160000000000 -3.753522000000000 0.904731000000000
C -0.898652000000000 -2.704773000000000 0.055322000000000
C -2.720206000000000 2.257752000000000 -0.288930000000000
H -0.613388000000000 2.363167000000000 -0.350737000000000
H -2.063063000000000 -0.309287000000000 0.146119000000000
H 0.726876000000000 -2.014796000000000 1.279245000000000
H 1.439743000000000 2.199728000000000 -0.959362000000000
H 3.077645000000000 1.430584000000000 -2.396369000000000
H 4.188113000000000 1.520230000000000 -1.040506000000000
H 3.411303000000000 -0.887794000000000 -2.434463000000000
H 4.240045000000000 -0.871002000000000 -0.885110000000000
H -5.754511000000000 1.087711000000000 -0.006842000000000
H -5.268125000000000 2.373560000000000 -1.143971000000000
H -5.223119000000000 2.678298000000000 0.602625000000000
H -1.662176000000000 -5.541272000000000 1.422830000000000
H -1.691606000000000 -5.213642000000000 -0.335209000000000
H -2.849414000000000 -4.393781000000000 0.736841000000000
S 2.529829000000000 2.292749000000000 1.099192000000000
C 3.501510000000000 0.994986000000000 1.908432000000000
H 4.427231000000000 0.796456000000000 1.367838000000000
H 3.742557000000000 1.353376000000000 2.908372000000000
H 2.917289000000000 0.078500000000000 1.982221000000000
44
R5_SMe4_P2_SMe3RS charge=0 multiplicity=1 B3LYP_D3BJ_TZVPD=-1757.184223670807, B3LYP_D3BJ_TZVPD_SMD_chcl3=-1757.211759363594, B3LYP_TZVP_CPCM_chcl3_freq=0.30079525, B3LYP_TZVPD_CPCM_chcl3=-1757.205779810062, M062X_TZVPD_SMD_chcl3=-1757.337290991266, M062X_TZVPD=-1757.309455648404, COSMORS=-0.0037197526036981997, wB97MV_TZVPD_CPCM_chcl3=-1757.369517480022, wB97MV_TZVPD_SMD_chcl3=-1757.375629062182, wB97MV_TZVPD=-1757.347889905491, M062X_TZVPD_CPCM_chcl3=-1757.331429774525
C -5.731315000000000 0.040079000000000 0.664044000000000
C 2.912802000000000 -3.320506000000000 1.640019000000000
C 0.789228000000000 -3.309563000000000 -2.289136000000000
C -0.390089000000000 2.605266000000000 -2.001618000000000
C 3.436185000000000 2.427556000000000 0.588800000000000
C 2.818464000000000 1.235663000000000 -0.160741000000000
C 2.280193000000000 3.423019000000000 0.717780000000000
C -2.174993000000000 0.263689000000000 -0.164001000000000
C -0.958618000000000 0.296860000000000 0.372185000000000
C 1.403176000000000 1.129332000000000 0.419647000000000
C 1.032832000000000 2.565434000000000 0.799227000000000
C -3.369509000000000 0.168741000000000 0.693260000000000
C 0.900319000000000 -1.131971000000000 -0.544551000000000
C 1.362562000000000 -1.777459000000000 0.745722000000000
C 0.347955000000000 0.320415000000000 -0.376625000000000
O -0.060655000000000 2.939915000000000 1.153705000000000
O -3.382076000000000 0.122514000000000 1.905527000000000
O 0.835417000000000 -1.661391000000000 1.827118000000000
O -4.489164000000000 0.136650000000000 -0.054618000000000
O 2.433823000000000 -2.548891000000000 0.519051000000000
S -0.325196000000000 -2.296168000000000 -1.276503000000000
S 0.175421000000000 0.889681000000000 -2.130928000000000
H -6.507422000000000 0.026446000000000 -0.095776000000000
H -5.856038000000000 0.898995000000000 1.322412000000000
H -5.757718000000000 -0.875692000000000 1.253564000000000
H 3.756994000000000 -3.887830000000000 1.260308000000000
H 2.129066000000000 -3.987077000000000 1.996338000000000
H 3.225900000000000 -2.657913000000000 2.445181000000000
H 0.157931000000000 -4.052382000000000 -2.775340000000000
H 1.529364000000000 -3.813579000000000 -1.671375000000000
H 1.278386000000000 -2.698166000000000 -3.046403000000000
H -0.704813000000000 2.871390000000000 -3.010323000000000
H 0.404905000000000 3.280168000000000 -1.693543000000000
H -1.238185000000000 2.690882000000000 -1.326791000000000
H 4.297316000000000 2.843514000000000 0.067775000000000
H 3.766977000000000 2.111627000000000 1.581059000000000
H 3.397180000000000 0.321997000000000 -0.032665000000000
H 2.759853000000000 1.449936000000000 -1.229490000000000
H 2.186369000000000 4.026208000000000 -0.192377000000000
H 2.339434000000000 4.110573000000000 1.560765000000000
H -2.333380000000000 0.305227000000000 -1.231909000000000
H -0.870894000000000 0.252306000000000 1.449668000000000
H 1.474872000000000 0.646446000000000 1.402728000000000
H 1.739314000000000 -1.100248000000000 -1.235809000000000
38
R5_SMe6_P2_0_1 charge=0 multiplicity=1 B3LYP_D3BJ_TZVPD=-1318.523204513805, CCSDT_SMD_chcl3=-1316.780517705564, CCSDT=-1316.757504016322, B3LYP_D3BJ_TZVPD_SMD_chcl3=-1318.546978189763, B3LYP_TZVP_CPCM_chcl3_freq=0.25370383, B3LYP_TZVPD_CPCM_chcl3=-1318.541089089525, M062X_TZVPD_SMD_chcl3=-1318.640855657722, M062X_TZVPD=-1318.61656180342, COSMORS=-0.0027796711796231046, wB97MV_TZVPD_CPCM_chcl3=-1318.680813923511, wB97MV_TZVPD_SMD_chcl3=-1318.686896499712, wB97MV_TZVPD=-1318.662721067633, M062X_TZVPD_CPCM_chcl3=-1318.635002178626
C -2.261689000000000 -0.608719000000000 0.642086000000000
C -0.939620000000000 -0.833406000000000 0.644081000000000
C 0.102111000000000 0.169581000000000 0.731359000000000
C -0.282659000000000 1.597388000000000 1.011514000000000
C 1.398578000000000 -0.180686000000000 0.544412000000000
C 1.952269000000000 -1.538356000000000 0.239266000000000
C 2.558267000000000 0.751922000000000 0.650668000000000
O 2.511558000000000 1.967136000000000 0.668649000000000
C 3.355392000000000 -1.520985000000000 0.876404000000000
C 3.830408000000000 -0.070910000000000 0.736597000000000
C -5.498092000000000 -2.295522000000000 0.373176000000000
O -4.474780000000000 -1.291883000000000 0.507739000000000
O -2.905114000000000 -2.904861000000000 0.410620000000000
O -0.246575000000000 2.047466000000000 -1.370313000000000
C -0.419059000000000 4.682562000000000 -1.014461000000000
O -0.386849000000000 3.751781000000000 0.086269000000000
C -0.291043000000000 2.457344000000000 -0.234381000000000
C -3.204802000000000 -1.732566000000000 0.509801000000000
H -2.697070000000000 0.377770000000000 0.710386000000000
H -0.626620000000000 -1.864271000000000 0.547500000000000
H 0.401307000000000 2.055834000000000 1.721880000000000
H 1.348023000000000 -2.370216000000000 0.588121000000000
H 3.254591000000000 -1.788801000000000 1.929532000000000
H 4.026455000000000 -2.242007000000000 0.411264000000000
H 4.445304000000000 0.278962000000000 1.565977000000000
H 4.399361000000000 0.090204000000000 -0.181863000000000
H -6.439613000000000 -1.754483000000000 0.386911000000000
H -5.448225000000000 -2.999095000000000 1.203270000000000
H -5.378167000000000 -2.832425000000000 -0.566941000000000
H 0.492889000000000 4.596919000000000 -1.603283000000000
H -0.489743000000000 5.666246000000000 -0.560000000000000
H -1.284239000000000 4.489086000000000 -1.646967000000000
S 2.199008000000000 -1.707859000000000 -1.586188000000000
C 0.530613000000000 -1.466919000000000 -2.246303000000000
H 0.632486000000000 -1.516972000000000 -3.330078000000000
H -0.144563000000000 -2.253693000000000 -1.914154000000000
H 0.143368000000000 -0.487255000000000 -1.971177000000000
H -1.278087000000000 1.654700000000000 1.452343000000000
44
R5_SMe1_P1A_SMe3S charge=0 multiplicity=1 B3LYP_D3BJ_TZVPD=-1757.208236686805, B3LYP_D3BJ_TZVPD_SMD_chcl3=-1757.233976340058, B3LYP_TZVP_CPCM_chcl3_freq=0.3015743, B3LYP_TZVPD_CPCM_chcl3=-1757.22804111866, COSMORS=-0.0038287071339892856, wB97MV_TZVPD_CPCM_chcl3=-1757.391851524318, wB97MV_TZVPD_SMD_chcl3=-1757.39791408786, wB97MV_TZVPD=-1757.371817513722
C -5.449966000000000 0.681863000000000 -0.062348000000000

C 3.703221000000000 -1.863916000000000 1.514571000000000
C 3.352297000000000 1.964899000000000 -1.893109000000000
C -1.325883000000000 2.905613000000000 1.155671000000000
C -0.106092000000000 -3.196306000000000 1.153192000000000
C -0.379926000000000 -1.732818000000000 1.306401000000000
C -0.070182000000000 -3.412339000000000 -0.362417000000000
C 0.510155000000000 1.046313000000000 -0.676427000000000
C -1.904035000000000 0.616386000000000 -1.111853000000000
C -0.519569000000000 -1.080449000000000 0.144706000000000
C -0.332525000000000 -2.045174000000000 -0.968590000000000
C 1.729970000000000 0.988513000000000 0.234365000000000
C -3.233364000000000 0.095496000000000 -0.634638000000000
C 2.451011000000000 -0.339537000000000 0.218454000000000
C -0.763059000000000 0.387689000000000 -0.092062000000000
O -0.372175000000000 -1.804583000000000 -2.160685000000000
O -3.472747000000000 -1.064286000000000 -0.386857000000000
O 2.642040000000000 -1.008908000000000 -0.771916000000000
O -4.143987000000000 1.074637000000000 -0.526773000000000
O 2.902991000000000 -0.668737000000000 1.434780000000000
S 2.976694000000000 2.280658000000000 -0.145737000000000
S -1.233669000000000 1.137365000000000 1.539913000000000
H -5.378634000000000 0.252898000000000 0.936260000000000
H -5.890100000000000 -0.045981000000000 -0.742430000000000
H -6.038949000000000 1.594133000000000 -0.045004000000000
H 3.139202000000000 -2.721356000000000 1.151868000000000
H 3.944693000000000 -1.981896000000000 2.566642000000000
H 4.610657000000000 -1.746669000000000 0.923639000000000
H 4.122086000000000 2.687899000000000 -2.160659000000000
H 2.477653000000000 2.124552000000000 -2.520594000000000
H 3.737079000000000 0.956928000000000 -2.032345000000000
H -0.352984000000000 3.308447000000000 0.879766000000000
H -1.653965000000000 3.381199000000000 2.079231000000000
H -2.057175000000000 3.112765000000000 0.376959000000000
H -0.886669000000000 -3.783173000000000 1.644695000000000
H 0.834261000000000 -3.464938000000000 1.641334000000000
H -0.452707000000000 -1.275215000000000 2.283402000000000
H -0.834117000000000 -4.107606000000000 -0.714076000000000
H 0.893391000000000 -3.777250000000000 -0.720894000000000
H 0.731525000000000 0.556542000000000 -1.624390000000000
H 0.290217000000000 2.089569000000000 -0.897847000000000
H -1.982878000000000 1.677246000000000 -1.335171000000000
H -1.647372000000000 0.089601000000000 -2.031427000000000
H 1.445614000000000 1.217856000000000 1.260522000000000
38
R5_SMe1_P2_conformer57_0_1 charge=0 multiplicity=1 B3LYP_D3BJ_TZVPD=-1318.51281629079, B3LYP_D3BJ_TZVPD_SMD_chcl3=-1318.540412884057, B3LYP_TZVP_CPCM_chcl3_freq=0.25144559, B3LYP_TZVPD_CPCM_chcl3=-1318.534155240349, M062X_TZVPD_SMD_chcl3=-1318.63535209737, M062X_TZVPD=-1318.607545645713, COSMORS=9.786306428514786e-05, wB97MV_TZVPD_CPCM_chcl3=-1318.676807986258, wB97MV_TZVPD_SMD_chcl3=-1318.683234735828, wB97MV_TZVPD=-1318.655420522178, M062X_TZVPD_CPCM_chcl3=-1318.629161252153
O -2.490495000000000 -2.439719000000000 -0.460351000000000
C -1.853107000000000 -1.934271000000000 0.599296000000000
H -2.114300000000000 -4.470295000000000 -0.167052000000000
H -3.659888000000000 -3.879477000000000 0.491239000000000
C -0.072873000000000 -0.202603000000000 0.835548000000000
C -1.457554000000000 -0.490728000000000 0.367136000000000
O 0.949983000000000 3.262183000000000 -0.957078000000000
O -1.631165000000000 -2.549519000000000 1.615210000000000
C -2.955913000000000 -3.800399000000000 -0.335634000000000
H -3.444058000000000 -4.026379000000000 -1.278677000000000
H -1.601006000000000 -0.248747000000000 -0.680760000000000
S -2.678798000000000 0.529636000000000 1.319370000000000
C 0.863102000000000 0.443438000000000 0.135523000000000
H 0.122345000000000 -0.504639000000000 1.856986000000000
H 3.697220000000000 -0.737384000000000 4.181222000000000
C 0.666694000000000 0.884510000000000 -1.255556000000000
H -2.946962000000000 2.865412000000000 1.103438000000000
H -1.265105000000000 2.413247000000000 0.756571000000000
C 2.218269000000000 0.807538000000000 0.711165000000000
C 0.404064000000000 0.125506000000000 -2.330694000000000
H -0.355666000000000 3.008723000000000 -3.375258000000000
C 0.260127000000000 0.903914000000000 -3.601871000000000
H 0.302663000000000 -0.952300000000000 -2.302321000000000
C 0.498180000000000 2.358673000000000 -3.178050000000000
H 0.978102000000000 0.557638000000000 -4.349861000000000
H -0.732496000000000 0.749470000000000 -4.034095000000000
C 0.745259000000000 2.304333000000000 -1.678533000000000
H 1.365010000000000 2.808181000000000 -3.665687000000000
C -2.307904000000000 2.147569000000000 0.591037000000000
H -2.535195000000000 2.154129000000000 -0.474177000000000
H 3.334561000000000 -2.359389000000000 3.520840000000000
H 2.015100000000000 -1.318684000000000 4.132686000000000
C 2.386797000000000 0.416717000000000 2.156794000000000
O 2.128522000000000 1.129950000000000 3.098199000000000
C 2.978859000000000 -1.338822000000000 3.626114000000000
O 2.842302000000000 -0.837469000000000 2.281301000000000
H 2.348098000000000 1.887666000000000 0.635645000000000
H 2.997226000000000 0.332386000000000 0.110335000000000
44
R5_SMe4_P2_SMe1SR charge=0 multiplicity=1 B3LYP_D3BJ_TZVPD=-1757.193375539758, B3LYP_D3BJ_TZVPD_SMD_chcl3=-1757.220660013654, B3LYP_TZVP_CPCM_chcl3_freq=0.3007733, B3LYP_TZVPD_CPCM_chcl3=-1757.213762335047, M062X_TZVPD_SMD_chcl3=-1757.346330835143, M062X_TZVPD=-1757.318600859953, COSMORS=-0.002718381268331674, wB97MV_TZVPD_CPCM_chcl3=-1757.377061389325, wB97MV_TZVPD_SMD_chcl3=-1757.384092225048, wB97MV_TZVPD=-1757.356508872087, M062X_TZVPD_CPCM_chcl3=-1757.339541191956
C 4.472329000000000 -2.197490000000000 -0.175111000000000
C -1.858635000000000 2.887306000000000 -3.054771000000000
C 1.670988000000000 3.082514000000000 0.748383000000000
C -1.448921000000000 -2.455301000000000 -1.169348000000000
C -1.753903000000000 -0.126125000000000 3.862948000000000
C -1.900573000000000 0.623935000000000 2.528727000000000
C -1.270162000000000 -1.518696000000000 3.445981000000000
C 0.455832000000000 0.352414000000000 -0.493950000000000
C -0.681123000000000 0.161673000000000 1.718830000000000
C -0.670877000000000 0.306334000000000 0.220758000000000
C -0.443566000000000 -1.282769000000000 2.198382000000000
C 1.841616000000000 0.395425000000000 0.046738000000000
C -2.009894000000000 0.250771000000000 -0.475269000000000
C 2.721862000000000 -0.668855000000000 -0.580802000000000
C -1.963978000000000 0.840774000000000 -1.876277000000000
O 0.319542000000000 -2.066542000000000 1.685512000000000
O 2.680712000000000 -0.991498000000000 -1.744280000000000
O -1.901178000000000 0.225473000000000 -2.911586000000000
O 3.561626000000000 -1.191040000000000 0.314778000000000
O -1.987490000000000 2.177908000000000 -1.805415000000000
S 2.635313000000000 2.021542000000000 -0.359762000000000
S -2.735460000000000 -1.430621000000000 -0.407580000000000
H 3.914387000000000 -3.044392000000000 -0.571064000000000
H 5.062902000000000 -2.495639000000000 0.685682000000000
H 5.108793000000000 -1.778763000000000 -0.953076000000000
H -0.908483000000000 2.640682000000000 -3.526346000000000
H -1.894782000000000 3.940747000000000 -2.794249000000000
H -2.679174000000000 2.627375000000000 -3.721458000000000
H 0.608877000000000 3.010346000000000 0.517462000000000
H 2.009871000000000 4.101847000000000 0.568408000000000
H 1.850330000000000 2.819929000000000 1.790312000000000
H -1.821483000000000 -3.477439000000000 -1.109339000000000
H -0.517777000000000 -2.377591000000000 -0.612723000000000
H -1.302872000000000 -2.176394000000000 -2.208947000000000
H -2.684881000000000 -0.153210000000000 4.427279000000000
H -0.999363000000000 0.360868000000000 4.485572000000000
H -1.931985000000000 1.706444000000000 2.647504000000000
H -2.827444000000000 0.308470000000000 2.049151000000000
H -2.113463000000000 -2.151243000000000 3.149543000000000
H -0.697138000000000 -2.062374000000000 4.196610000000000
H 0.408334000000000 0.382986000000000 -1.575306000000000
H 0.184323000000000 0.696791000000000 2.125409000000000
H 1.888522000000000 0.281350000000000 1.124578000000000
H -2.729398000000000 0.849838000000000 0.082058000000000
41
R6_SMe6_esterH_0_1 charge=0 multiplicity=1 B3LYP_TZVP_CPCM_chcl3_freq=0.28378168, B3LYP_TZVPD_CPCM_chcl3=-1357.817768850125
O -4.015173000000000 -1.195226000000000 1.107355000000000
C -3.161191000000000 -0.712064000000000 0.182894000000000
C -0.888429000000000 2.312671000000000 -0.615970000000000
H -4.832877000000000 0.617923000000000 1.745495000000000
C -0.892292000000000 -0.782793000000000 -0.863735000000000
C -1.859572000000000 -1.033922000000000 0.237821000000000
H -5.851234000000000 -0.315382000000000 0.618074000000000
O -3.734738000000000 0.054000000000000 -0.757266000000000
C -5.151628000000000 -0.383058000000000 1.449436000000000
H -5.621561000000000 -0.885729000000000 2.290974000000000
H -2.545743000000000 4.336247000000000 -0.048935000000000
H 1.135154000000000 1.972237000000000 0.009980000000000
C 0.269671000000000 0.122061000000000 -0.501109000000000
H -1.915465000000000 4.535888000000000 -1.700402000000000
C 0.238759000000000 1.467019000000000 -0.321321000000000
C 1.415831000000000 -0.723701000000000 -0.410685000000000
C 2.813730000000000 -0.374237000000000 -0.033570000000000
H -1.249509000000000 5.524256000000000 -0.368817000000000
S 2.878289000000000 -0.169223000000000 1.801168000000000
C 1.051557000000000 -1.961293000000000 -0.840097000000000
H 4.027992000000000 -3.587626000000000 -0.627633000000000
H 3.076682000000000 0.603078000000000 -0.444272000000000
C 1.951283000000000 -3.127378000000000 -0.998015000000000
O -0.234755000000000 -2.076289000000000 -1.179104000000000
H 4.771522000000000 -1.295370000000000 -0.105465000000000
C 3.287476000000000 -2.860249000000000 -0.293131000000000
H 1.461916000000000 -4.023330000000000 -0.608577000000000
H 2.103166000000000 -3.298288000000000 -2.070077000000000
C 3.788734000000000 -1.439263000000000 -0.555206000000000
H 3.162038000000000 -3.000304000000000 0.783189000000000
H 3.907178000000000 -1.286454000000000 -1.633603000000000
C 4.428032000000000 0.758506000000000 1.975607000000000
H 5.283976000000000 0.171483000000000 1.645080000000000
H 4.379123000000000 1.692435000000000 1.415472000000000
H 4.541815000000000 0.983225000000000 3.035408000000000
O -0.616679000000000 3.607274000000000 -0.358525000000000
O -1.979638000000000 1.982518000000000 -1.082965000000000
C -1.658480000000000 4.555607000000000 -0.641879000000000
H -1.385450000000000 -0.462989000000000 -1.779897000000000
H -1.491561000000000 -1.562022000000000 1.106135000000000
H -3.083206000000000 0.749895000000000 -1.023106000000000
54
Sulf_SMe6_-1_1 charge=-1 multiplicity=1 wB97MV_TZVPD_SMD_chcl3=-2138.167104315396, B3LYP_D3BJ_TZVPD=-2137.866410848211, B3LYP_TZVP_CPCM_chcl3_freq=0.35579415, B3LYP_TZVPD_CPCM_chcl3=-2137.933688038445, M062X_TZVPD=-2138.017449374643, COSMORS=-0.015092345839248957, M062X_TZVPD_CPCM_chcl3=-2138.087783352989, M062X_TZVPD_SMD_chcl3=-2138.094886959509, wB97MV_TZVPD_CPCM_chcl3=-2138.159832859081, B3LYP_D3BJ_TZVPD_SMD_chcl3=-2137.940465995101, wB97MV_TZVPD=-2138.091015338201
C 2.995422000000000 -0.542180000000000 0.920892000000000
C 3.105631000000000 -1.723742000000000 1.641115000000000
C 2.231945000000000 -1.980638000000000 2.692248000000000
C 1.254236000000000 -1.052006000000000 3.037675000000000
C 1.145072000000000 0.137762000000000 2.332337000000000
C 2.016710000000000 0.378802000000000 1.276710000000000
O 1.343428000000000 2.905078000000000 1.198064000000000
O 3.085165000000000 2.096943000000000 -0.389035000000000
S 1.828085000000000 1.859799000000000 0.309284000000000
C 1.740726000000000 2.201450000000000 -3.347868000000000
S 0.422306000000000 2.703558000000000 -2.201529000000000
C -1.634382000000000 3.165393000000000 0.226283000000000
C -2.675203000000000 4.086165000000000 0.305950000000000
C -3.964407000000000 3.732644000000000 -0.068396000000000
C -4.197432000000000 2.439000000000000 -0.535509000000000
C -3.160148000000000 1.525521000000000 -0.619040000000000
C -1.847575000000000 1.858245000000000 -0.235968000000000
C 2.966954000000000 -4.332147000000000 -1.517724000000000
O 1.944796000000000 -3.386165000000000 -1.177703000000000
O 1.768400000000000 -2.833157000000000 -3.351432000000000
O -1.551884000000000 -3.300428000000000 0.726581000000000
C -3.492533000000000 -3.748994000000000 2.435991000000000
O -3.202454000000000 -2.421179000000000 2.011937000000000
C -2.153888000000000 -2.287541000000000 1.106795000000000
C 1.420001000000000 -2.676111000000000 -2.193949000000000
C -1.943996000000000 -0.934944000000000 0.768329000000000
C 0.537360000000000 1.361186000000000 -0.973941000000000
C -0.758390000000000 0.882184000000000 -0.424208000000000
C -0.911279000000000 -0.460618000000000 -0.064916000000000
C 0.080598000000000 -1.466642000000000 -0.499728000000000
C 0.413079000000000 -1.700118000000000 -1.774091000000000
H 3.652013000000000 -0.334415000000000 0.088147000000000
H 3.861442000000000 -2.449119000000000 1.370983000000000
H 2.307440000000000 -2.911037000000000 3.240414000000000
H 0.568409000000000 -1.258920000000000 3.848509000000000
H 0.384415000000000 0.862722000000000 2.582554000000000
H 2.715534000000000 2.279683000000000 -2.874386000000000
H 1.568612000000000 1.183943000000000 -3.698103000000000
H 1.684738000000000 2.886477000000000 -4.193448000000000
H -0.645021000000000 3.463176000000000 0.537279000000000
H -2.470620000000000 5.086057000000000 0.670300000000000
H -4.774497000000000 4.448312000000000 -0.003104000000000
H -5.192667000000000 2.146260000000000 -0.848845000000000
H -3.356290000000000 0.532791000000000 -1.000363000000000
H 2.582929000000000 -5.080077000000000 -2.210925000000000
H 3.255277000000000 -4.798345000000000 -0.579535000000000
H 3.820920000000000 -3.826435000000000 -1.968265000000000
H -4.333428000000000 -3.665510000000000 3.122567000000000
H -3.765588000000000 -4.386544000000000 1.592871000000000
H -2.640181000000000 -4.198234000000000 2.949775000000000
H -2.624327000000000 -0.223384000000000 1.209428000000000
H 1.083035000000000 0.551301000000000 -1.445592000000000
H 0.535863000000000 -2.066125000000000 0.274181000000000
H -0.045071000000000 -1.150451000000000 -2.586881000000000
41
R6_SMe4_P3_0_1 charge=0 multiplicity=1 B3LYP_D3BJ_TZVPD=-1357.824144268906, B3LYP_D3BJ_TZVPD_SMD_chcl3=-1357.848043408792, B3LYP_TZVP_CPCM_chcl3_freq=0.28011128, B3LYP_TZVPD_CPCM_chcl3=-1357.841739328303, M062X_TZVPD_SMD_chcl3=-1357.947142892572, M062X_TZVPD=-1357.922883538183, COSMORS=-0.004056576203556673, wB97MV_TZVPD_CPCM_chcl3=-1357.984928289706, wB97MV_TZVPD_SMD_chcl3=-1357.991414830011, wB97MV_TZVPD=-1357.967210410204, M062X_TZVPD_CPCM_chcl3=-1357.940863408729
O 3.865183000000000 1.827312000000000 1.078196000000000
C 2.732709000000000 1.544576000000000 0.419942000000000
O 2.590311000000000 1.724007000000000 -0.767648000000000
H 4.663675000000000 3.289169000000000 -0.177090000000000
H 5.769626000000000 2.504824000000000 0.988479000000000
C 1.698775000000000 0.944838000000000 1.350706000000000
C 0.306854000000000 1.004533000000000 0.806716000000000
C 4.953408000000000 2.348742000000000 0.289368000000000
H 5.239162000000000 1.631888000000000 -0.479217000000000
H 1.762565000000000 1.470390000000000 2.305579000000000
H 2.014925000000000 -0.084892000000000 1.535340000000000
O -3.834885000000000 0.652926000000000 0.630027000000000
C -0.350879000000000 -0.016730000000000 0.256561000000000
O -2.368186000000000 2.337799000000000 0.403177000000000
C -4.813749000000000 1.587639000000000 1.125790000000000
H -5.695536000000000 0.993388000000000 1.345615000000000
H -4.445243000000000 2.075958000000000 2.026599000000000
H -5.034320000000000 2.336771000000000 0.366553000000000
C -1.753039000000000 0.089835000000000 -0.288571000000000
C 0.268026000000000 -1.351639000000000 0.056369000000000
C -2.645561000000000 1.165164000000000 0.287852000000000
H -0.188748000000000 1.962884000000000 0.878181000000000
C 1.278819000000000 -1.536652000000000 -0.809453000000000
H 1.629711000000000 -0.683050000000000 -1.378894000000000
O -1.191843000000000 -2.348845000000000 1.620029000000000
C 1.958205000000000 -2.843810000000000 -1.050314000000000
H -0.348553000000000 -4.236338000000000 -0.391954000000000
H 0.040259000000000 -4.535307000000000 1.300384000000000
C 1.719428000000000 -3.835175000000000 0.084523000000000
H 1.584879000000000 -3.252744000000000 -1.999243000000000
H 3.026401000000000 -2.670986000000000 -1.204269000000000
C 0.239966000000000 -3.871037000000000 0.459487000000000
H 2.062729000000000 -4.831726000000000 -0.197071000000000
H 2.303554000000000 -3.526876000000000 0.956708000000000
C -0.300007000000000 -2.500396000000000 0.797347000000000
S -1.752590000000000 0.275734000000000 -2.129278000000000
H -2.260215000000000 -0.862311000000000 -0.153620000000000
C -0.784580000000000 1.788335000000000 -2.364839000000000
H -0.670085000000000 1.896145000000000 -3.443158000000000
H -1.308585000000000 2.653341000000000 -1.964315000000000
H 0.196342000000000 1.693292000000000 -1.902555000000000
44
R5_SMe4_P2_SMe2SP2R charge=0 multiplicity=1 B3LYP_D3BJ_TZVPD=-1757.198076828591, B3LYP_D3BJ_TZVPD_SMD_chcl3=-1757.223709306408, B3LYP_TZVP_CPCM_chcl3_freq=0.30060722, B3LYP_TZVPD_CPCM_chcl3=-1757.217182868761, COSMORS=-0.00347801920162339, wB97MV_TZVPD_CPCM_chcl3=-1757.379962974445, wB97MV_TZVPD_SMD_chcl3=-1757.386666429926, wB97MV_TZVPD=-1757.36059279495
C 5.682180000000000 0.178858000000000 -1.656834000000000
C -4.306774000000000 1.635071000000000 -1.235675000000000
C 1.254870000000000 -0.431583000000000 3.102847000000000
C -1.657334000000000 3.297743000000000 1.559102000000000
C -1.934113000000000 -3.527165000000000 0.345181000000000
C -1.221137000000000 -2.366627000000000 1.060498000000000
C -2.540422000000000 -2.891484000000000 -0.910114000000000
C 2.060488000000000 -0.114505000000000 -0.956178000000000
C -0.638174000000000 -1.532568000000000 -0.099354000000000
C -1.645374000000000 -1.714313000000000 -1.237405000000000
C 1.308766000000000 0.060019000000000 0.366159000000000
C 3.463266000000000 0.444652000000000 -0.888922000000000
C -0.177775000000000 -0.129072000000000 0.197115000000000
C -0.999536000000000 0.939028000000000 0.238350000000000
C -2.466154000000000 0.806294000000000 -0.017400000000000
O -1.697687000000000 -1.042447000000000 -2.242305000000000
O 3.775221000000000 1.455995000000000 -0.305121000000000
O -3.186083000000000 -0.008755000000000 0.518031000000000
O 4.322226000000000 -0.298335000000000 -1.597159000000000
O -2.905362000000000 1.698695000000000 -0.906428000000000
S 2.033658000000000 -1.084889000000000 1.601875000000000
S -0.398099000000000 2.594446000000000 0.452833000000000
H 5.713912000000000 1.168420000000000 -2.110315000000000
H 6.215023000000000 -0.541191000000000 -2.270606000000000
H 6.108916000000000 0.220476000000000 -0.655890000000000
H -4.542944000000000 0.667340000000000 -1.675139000000000
H -4.469464000000000 2.432166000000000 -1.954617000000000
H -4.910673000000000 1.791972000000000 -0.343147000000000
H 0.171557000000000 -0.530289000000000 3.050690000000000
H 1.527389000000000 0.612824000000000 3.248097000000000
H 1.629583000000000 -1.024080000000000 3.936009000000000
H -1.310131000000000 4.302763000000000 1.794617000000000
H -1.729438000000000 2.715333000000000 2.475557000000000
H -2.625435000000000 3.360574000000000 1.066056000000000
H -2.681403000000000 -4.003911000000000 0.978304000000000
H -1.204213000000000 -4.289152000000000 0.061125000000000
H -0.440425000000000 -2.707438000000000 1.736717000000000
H -1.932927000000000 -1.764004000000000 1.620822000000000
H -3.528747000000000 -2.469829000000000 -0.702227000000000
H -2.639856000000000 -3.556267000000000 -1.768317000000000
H 1.535538000000000 0.446999000000000 -1.734904000000000
H 2.093004000000000 -1.154429000000000 -1.278139000000000
H 0.235343000000000 -2.093597000000000 -0.458144000000000
H 1.507848000000000 1.064111000000000 0.731820000000000
38
R5_SMe6_P1SS_0_1 charge=0 multiplicity=1 B3LYP_D3BJ_TZVPD=-1318.518866615415, CCSDT_SMD_chcl3=-1316.776405167694, CCSDT=-1316.754195397169, B3LYP_D3BJ_TZVPD_SMD_chcl3=-1318.542019633023, B3LYP_TZVP_CPCM_chcl3_freq=0.25343254, B3LYP_TZVPD_CPCM_chcl3=-1318.53682496698, M062X_TZVPD_SMD_chcl3=-1318.635790076101, M062X_TZVPD=-

1318.612346232607, COSMORS=-0.002385669160182298, wB97MV_TZVPD_CPCM_chcl3=-1318.677170063759, wB97MV_TZVPD_SMD_chcl3=-1318.6825388337, wB97MV_TZVPD=-1318.65919662511, M062X_TZVPD_CPCM_chcl3=-1318.630649295008
C 2.267550000000000 -0.456908000000000 -0.390267000000000
C 1.172113000000000 -1.155367000000000 -0.066970000000000
C -0.206385000000000 -0.688326000000000 -0.119125000000000
C -1.140282000000000 -1.540469000000000 0.356628000000000
C -0.497028000000000 0.673520000000000 -0.674770000000000
C -0.876109000000000 1.788674000000000 0.321924000000000
C -1.580817000000000 0.750487000000000 -1.759385000000000
O -1.789838000000000 -0.093387000000000 -2.595595000000000
C -1.504576000000000 2.870065000000000 -0.575436000000000
C -2.270223000000000 2.096232000000000 -1.653587000000000
C 5.919373000000000 -0.650956000000000 -0.531656000000000
O 4.559155000000000 -0.187421000000000 -0.620483000000000
O 3.844133000000000 -2.182829000000000 0.141957000000000
O -3.164283000000000 -0.263180000000000 0.173420000000000
C -4.652999000000000 -2.312052000000000 0.971593000000000
O -3.221348000000000 -2.406125000000000 0.853881000000000
C -2.585274000000000 -1.299749000000000 0.433753000000000
C 3.606934000000000 -1.059734000000000 -0.250560000000000
H 2.237292000000000 0.564066000000000 -0.741006000000000
H 1.315637000000000 -2.167322000000000 0.295943000000000
H -0.820788000000000 -2.506482000000000 0.724168000000000
H -1.614227000000000 1.414508000000000 1.027804000000000
H -2.136243000000000 3.543356000000000 0.000726000000000
H -0.711906000000000 3.466223000000000 -1.034820000000000
H -3.300879000000000 1.897077000000000 -1.353053000000000
H -2.296010000000000 2.587923000000000 -2.626195000000000
H 6.064370000000000 -1.518186000000000 -1.174604000000000
H 6.160966000000000 -0.915404000000000 0.497028000000000
H 6.532321000000000 0.180464000000000 -0.866637000000000
H -4.921890000000000 -1.545874000000000 1.697778000000000
H -4.981362000000000 -3.290635000000000 1.308693000000000
H -5.095060000000000 -2.070279000000000 0.005969000000000
S 0.496284000000000 2.488946000000000 1.285745000000000
C 0.648341000000000 1.282512000000000 2.633359000000000
H 1.349143000000000 1.706743000000000 3.351169000000000
H -0.316376000000000 1.135374000000000 3.117739000000000
H 1.036177000000000 0.330711000000000 2.277313000000000
H 0.388616000000000 1.031550000000000 -1.209176000000000
44
R5_SMe4_P3_SMe6SS_P3 charge=0 multiplicity=1 B3LYP_D3BJ_TZVPD=-1757.200285607112, B3LYP_D3BJ_TZVPD_SMD_chcl3=-1757.226572663766, B3LYP_TZVP_CPCM_chcl3_freq=0.30045034, B3LYP_TZVPD_CPCM_chcl3=-1757.219890868434, M062X_TZVPD_SMD_chcl3=-1757.352352500979, M062X_TZVPD=-1757.325652077584, COSMORS=-0.0031500082177150363, wB97MV_TZVPD_CPCM_chcl3=-1757.382867483698, wB97MV_TZVPD_SMD_chcl3=-1757.389719383762, wB97MV_TZVPD=-1757.363092077142, M062X_TZVPD_CPCM_chcl3=-1757.34576345427
C 6.045087000000000 0.460822000000000 1.039070000000000
C -3.711172000000000 2.835777000000000 0.879349000000000
C -0.408520000000000 -2.268025000000000 -2.738760000000000
C -1.147036000000000 3.035503000000000 -2.452145000000000
C 1.443015000000000 -0.116244000000000 -0.517097000000000
C -2.424893000000000 -1.912003000000000 2.065786000000000
C -2.049083000000000 -2.964540000000000 1.018001000000000
C 2.378343000000000 0.373836000000000 0.590053000000000
C -0.001122000000000 0.053943000000000 -0.130395000000000
C -1.422552000000000 -0.789830000000000 1.890710000000000
C -1.372975000000000 -2.161080000000000 -0.104312000000000
C 3.831901000000000 0.128643000000000 0.271530000000000
C -0.559480000000000 -1.094083000000000 0.664556000000000
C -0.688478000000000 1.174901000000000 -0.427552000000000
C -2.109242000000000 1.353790000000000 -0.012829000000000
O -1.294850000000000 0.166485000000000 2.618197000000000
O 4.249774000000000 -0.446897000000000 -0.706552000000000
O -2.958902000000000 0.490335000000000 -0.091715000000000
O 4.626549000000000 0.634746000000000 1.226144000000000
O -2.359126000000000 2.579010000000000 0.452735000000000
S -0.427569000000000 -3.248930000000000 -1.210936000000000
S 0.099560000000000 2.550751000000000 -1.221023000000000
H 6.366441000000000 0.946990000000000 0.118960000000000
H 6.293733000000000 -0.598785000000000 0.998494000000000
H 6.511144000000000 0.929418000000000 1.900851000000000
H -3.718413000000000 3.872032000000000 1.203284000000000
H -3.975655000000000 2.174287000000000 1.702677000000000
H -4.401788000000000 2.686386000000000 0.050692000000000
H 0.064048000000000 -2.888239000000000 -3.499245000000000
H 0.164675000000000 -1.351054000000000 -2.621316000000000
H -1.426181000000000 -2.032851000000000 -3.048073000000000
H -1.365644000000000 2.206074000000000 -3.122013000000000
H -0.699731000000000 3.850914000000000 -3.018967000000000
H -2.055876000000000 3.390011000000000 -1.970260000000000
H 1.635317000000000 -1.174336000000000 -0.699756000000000
H 1.658119000000000 0.430104000000000 -1.433938000000000
H -2.429955000000000 -2.271783000000000 3.094700000000000
H -3.408644000000000 -1.480203000000000 1.860403000000000
H -2.904123000000000 -3.525274000000000 0.645319000000000
H -1.336740000000000 -3.678529000000000 1.439620000000000
H 2.159731000000000 -0.119330000000000 1.541477000000000
H 2.245807000000000 1.442910000000000 0.770645000000000
H -2.126873000000000 -1.660401000000000 -0.707249000000000
H 0.292388000000000 -1.630782000000000 1.105159000000000
38
R5_SMe6H_cyc4R_0_1 charge=0 multiplicity=1 B3LYP_D3BJ_TZVPD_SMD_chcl3=-1318.48930212858, B3LYP_TZVP_CPCM_chcl3_freq=0.25448908, B3LYP_TZVPD_CPCM_chcl3=-1318.483304110046, M062X_TZVPD_SMD_chcl3=-1318.592785276142, M062X_TZVPD=-1318.568317605657, COSMORS=-0.002930473683666478, wB97MV_TZVPD_CPCM_chcl3=-1318.627801449913, wB97MV_TZVPD_SMD_chcl3=-1318.633967417022, wB97MV_TZVPD=-1318.609794560094, M062X_TZVPD_CPCM_chcl3=-1318.586770897207
C -1.890866000000000 1.009895000000000 -0.463253000000000
C -1.177197000000000 -0.213428000000000 -0.705272000000000
C 0.161652000000000 -0.341402000000000 -0.794199000000000
C 0.884554000000000 -1.684560000000000 -1.004062000000000
C 1.249553000000000 0.607665000000000 -0.729209000000000
C 1.506944000000000 2.039536000000000 -0.408379000000000
C 2.397514000000000 -0.040962000000000 -0.958717000000000
O 2.286474000000000 -1.372499000000000 -1.190517000000000
C 3.022274000000000 2.201608000000000 -0.764275000000000
C 3.630642000000000 0.775731000000000 -0.900154000000000
C -5.332301000000000 -0.010834000000000 -0.174927000000000
O -4.025589000000000 0.062342000000000 -0.771576000000000
O -3.933254000000000 2.223978000000000 -0.155529000000000
O 1.487341000000000 -2.533167000000000 1.187049000000000
C -0.742600000000000 -4.018703000000000 1.348156000000000
O -0.384273000000000 -3.266347000000000 0.170255000000000
C 0.735477000000000 -2.543028000000000 0.244254000000000
C -3.237747000000000 1.098229000000000 -0.446611000000000
H -1.336280000000000 1.918499000000000 -0.273070000000000
H -1.777882000000000 -1.108493000000000 -0.817916000000000
H 0.534339000000000 -2.235981000000000 -1.875569000000000
H 0.894181000000000 2.738687000000000 -0.978646000000000
H 3.098652000000000 2.721218000000000 -1.719748000000000
H 3.543279000000000 2.802247000000000 -0.021337000000000
H 4.250341000000000 0.673037000000000 -1.793729000000000
H 4.250242000000000 0.495233000000000 -0.042703000000000
H -5.260055000000000 0.047240000000000 0.912256000000000
H -5.728162000000000 -0.978982000000000 -0.469708000000000
H -5.977203000000000 0.783957000000000 -0.546448000000000
H 0.031327000000000 -4.749896000000000 1.575558000000000
H -0.874015000000000 -3.345555000000000 2.194021000000000
H -1.677243000000000 -4.513433000000000 1.102517000000000
S 1.082152000000000 2.485098000000000 1.334396000000000
C 2.021749000000000 1.240835000000000 2.260108000000000
H 1.731654000000000 0.234455000000000 1.963440000000000
H 3.094264000000000 1.374119000000000 2.122910000000000
H 1.777936000000000 1.390179000000000 3.311081000000000
H -3.321989000000000 2.911043000000000 0.142876000000000
37
R5_SMe1_-1_1 charge=-1 multiplicity=1 wB97MV_TZVPD_SMD_chcl3=-1318.171032419535, B3LYP_D3BJ_TZVPD=-1317.956819809508, CCSDT_SMD_chcl3=-1316.257042527591, CCSDT=-1316.183546824552, B3LYP_TZVP_CPCM_chcl3_freq=0.23707753, B3LYP_TZVPD_CPCM_chcl3=-1318.027379613989, M062X_TZVPD=-1318.050749247279, COSMORS=-0.007982493025241675, M062X_TZVPD_CPCM_chcl3=-1318.124214991524, M062X_TZVPD_SMD_chcl3=-1318.125992776536, wB97MV_TZVPD_CPCM_chcl3=-1318.168991895272, B3LYP_D3BJ_TZVPD_SMD_chcl3=-1318.029068389372, wB97MV_TZVPD=-1318.096690960145
C 1.808530000000000 -0.022315000000000 0.075205000000000
C 0.693043000000000 0.923736000000000 0.178390000000000
C -0.636069000000000 0.577258000000000 0.161392000000000
C -1.683545000000000 1.524280000000000 0.254109000000000
C -0.990632000000000 -0.854158000000000 0.018108000000000
C -0.921678000000000 -1.809192000000000 0.953454000000000
C -1.455070000000000 -1.455600000000000 -1.249208000000000
O -1.519900000000000 -0.914475000000000 -2.337825000000000
C -1.380751000000000 -3.157577000000000 0.481596000000000
C -1.797718000000000 -2.913829000000000 -0.974420000000000
C 4.784028000000000 0.046804000000000 -2.111351000000000
O 3.583063000000000 -0.367142000000000 -1.438251000000000
O 3.441761000000000 1.669355000000000 -0.497880000000000
O -3.646612000000000 0.153958000000000 0.075413000000000
C -5.231783000000000 2.229088000000000 0.302326000000000
O -3.821632000000000 2.403192000000000 0.348507000000000
C -3.058554000000000 1.237145000000000 0.211471000000000
C 3.009533000000000 0.544519000000000 -0.630739000000000
H 1.528918000000000 -0.965038000000000 -0.385605000000000
H 0.956117000000000 1.968778000000000 0.285578000000000
H -1.408945000000000 2.566412000000000 0.361107000000000
H -0.583680000000000 -1.639766000000000 1.967170000000000
H -0.577973000000000 -3.896233000000000 0.565875000000000
H -2.204761000000000 -3.524016000000000 1.100288000000000
H -1.283122000000000 -3.555246000000000 -1.691050000000000
H -2.870686000000000 -3.046623000000000 -1.127580000000000
H 5.554241000000000 0.305934000000000 -1.385266000000000
H 4.585303000000000 0.905422000000000 -2.751666000000000
H 5.094478000000000 -0.807554000000000 -2.706422000000000
H -5.551683000000000 1.794417000000000 -0.647200000000000
H -5.659964000000000 3.224719000000000 0.412027000000000
H -5.584744000000000 1.586692000000000 1.112292000000000
S 2.544768000000000 -0.660061000000000 1.744593000000000
C 2.633022000000000 0.892628000000000 2.672864000000000
H 2.897843000000000 0.634565000000000 3.697597000000000
H 3.387612000000000 1.558430000000000 2.256865000000000
H 1.660233000000000 1.383744000000000 2.663177000000000
41
R6_SMe6_cyc2SP1_0_1 charge=0 multiplicity=1 B3LYP_D3BJ_TZVPD=-1357.832232884021, B3LYP_D3BJ_TZVPD_SMD_chcl3=-1357.855242010708, B3LYP_TZVP_CPCM_chcl3_freq=0.28373249, B3LYP_TZVPD_CPCM_chcl3=-1357.848266043006, M062X_TZVPD_SMD_chcl3=-1357.959668485103, M062X_TZVPD=-1357.936059013531, COSMORS=-0.005707164828601206, wB97MV_TZVPD_CPCM_chcl3=-1357.994719002816, wB97MV_TZVPD_SMD_chcl3=-1358.001869086316, wB97MV_TZVPD=-1357.978445445017, M062X_TZVPD_CPCM_chcl3=-1357.952742925743
O -4.837048000000000 0.358538000000000 -0.864362000000000
C -3.979536000000000 0.380451000000000 0.161870000000000
C 1.563165000000000 2.291556000000000 -0.306580000000000
H -6.752237000000000 0.151299000000000 -1.484800000000000
C -1.517015000000000 0.113584000000000 0.618094000000000
C -2.575973000000000 0.686557000000000 -0.305634000000000
H -6.342622000000000 -0.831001000000000 -0.047424000000000
O -4.303484000000000 0.207566000000000 1.313486000000000
C -6.224287000000000 0.134741000000000 -0.536129000000000
H -6.587510000000000 0.924824000000000 0.119365000000000
H 2.970467000000000 5.160881000000000 0.262123000000000
H -0.358011000000000 2.495370000000000 0.617592000000000
C -0.101907000000000 0.463458000000000 0.192846000000000
H 2.941185000000000 4.334050000000000 -1.322116000000000
C 0.309164000000000 1.753653000000000 0.196392000000000
C 0.518506000000000 -0.795940000000000 -0.121008000000000
C 1.917574000000000 -1.125837000000000 -0.524933000000000
H 3.860828000000000 3.630175000000000 0.026728000000000
S 3.005093000000000 -0.878638000000000 0.951253000000000
C -0.396376000000000 -1.772582000000000 0.116352000000000
H 1.390984000000000 -4.572706000000000 -0.610146000000000
H 2.274440000000000 -0.403651000000000 -1.253004000000000
C -0.209988000000000 -3.230737000000000 -0.065663000000000
O -1.590085000000000 -1.337481000000000 0.553402000000000
H 3.048006000000000 -2.832542000000000 -1.208811000000000
C 1.279385000000000 -3.567247000000000 -0.202604000000000
H -0.669567000000000 -3.763986000000000 0.769814000000000
H -0.760146000000000 -3.531437000000000 -0.965181000000000
C 2.001538000000000 -2.550625000000000 -1.085038000000000
H 1.740695000000000 -3.567042000000000 0.787752000000000
H 1.551738000000000 -2.546573000000000 -2.084376000000000
C 4.593982000000000 -0.587182000000000 0.125841000000000
H 5.327654000000000 -0.393760000000000 0.907552000000000
H 4.908219000000000 -1.459348000000000 -0.446616000000000
H 4.519491000000000 0.282894000000000 -0.525202000000000
O 1.787572000000000 3.524273000000000 0.214745000000000
O 2.324202000000000 1.790168000000000 -1.114675000000000
C 2.969924000000000 4.197776000000000 -0.241155000000000
H -1.718981000000000 0.391798000000000 1.652306000000000
H -2.472173000000000 1.774869000000000 -0.331971000000000
H -2.429206000000000 0.323828000000000 -1.322765000000000
37
R5_SMe6_cyc4RZTS_-1_1 charge=-1 multiplicity=1 wB97MV_TZVPD_SMD_chcl3=-1318.126649556717, B3LYP_TZVP_CPCM_chcl3_freq=0.23889776, B3LYP_TZVPD_CPCM_chcl3=-1317.984373068865, M062X_TZVPD=-1318.010031362808, COSMORS=-0.01002139451259466, M062X_TZVPD_CPCM_chcl3=-1318.07866761483, M062X_TZVPD_SMD_chcl3=-1318.080747771497, wB97MV_TZVPD_CPCM_chcl3=-1318.124338646122, B3LYP_D3BJ_TZVPD_SMD_chcl3=-1317.986320131925, wB97MV_TZVPD=-1318.057034477898
C -2.701884000000000 0.249308000000000 -0.128953000000000
C -1.404820000000000 0.738316000000000 -0.191578000000000
C -0.217297000000000 0.017711000000000 -0.245604000000000
C -0.145286000000000 -1.383440000000000 -0.546157000000000
C 1.104138000000000 0.588622000000000 -0.445829000000000
C 1.743503000000000 1.900183000000000 -0.194610000000000
C 1.845918000000000 -0.198956000000000 -1.255041000000000
O 1.403343000000000 -1.398091000000000 -1.603791000000000
C 2.953729000000000 1.903145000000000 -1.180944000000000
C 3.087442000000000 0.468541000000000 -1.757663000000000
C -6.205353000000000 1.205747000000000 -0.040327000000000
O -5.017133000000000 0.417720000000000 -0.064131000000000
O -3.839834000000000 2.353180000000000 -0.127616000000000
O 1.072920000000000 -2.117311000000000 1.411632000000000
C 0.836245000000000 -4.675060000000000 0.751409000000000
O 0.262728000000000 -3.625448000000000 -0.043995000000000
C 0.477037000000000 -2.367826000000000 0.387503000000000
C -3.826412000000000 1.120290000000000 -0.111907000000000
H -2.896966000000000 -0.813028000000000 -0.063850000000000
H -1.305784000000000 1.820442000000000 -0.151539000000000
H -0.962625000000000 -1.821505000000000 -1.113040000000000
H 1.086759000000000 2.754463000000000 -0.374004000000000
H 2.746375000000000 2.608149000000000 -1.987191000000000
H 3.866387000000000 2.237412000000000 -0.688318000000000
H 3.131776000000000 0.464433000000000 -2.850903000000000
H 3.988213000000000 -0.044773000000000 -1.403542000000000
H -6.236266000000000 1.848427000000000 0.841230000000000
H -7.031419000000000 0.497538000000000 -0.010938000000000
H -6.283619000000000 1.830703000000000 -0.931652000000000
H 0.559098000000000 -5.602060000000000 0.256999000000000
H 1.920560000000000 -4.572763000000000 0.788685000000000
H 0.435376000000000 -4.648049000000000 1.764258000000000
S 2.215836000000000 2.176514000000000 1.584945000000000
C 3.222346000000000 0.700759000000000 1.895917000000000
H 3.492143000000000 0.718667000000000 2.951397000000000
H 2.646740000000000 -0.197819000000000 1.681412000000000
H 4.133242000000000 0.712848000000000 1.296709000000000
32
R5_0_1 charge=0 multiplicity=1 B3LYP_D3BJ_TZVPD=-879.834895991977, CCSDT_SMD_chcl3=-878.566437068801, CCSDT=-878.545092021997, B3LYP_D3BJ_TZVPD_SMD_chcl3=-879.857509764027, B3LYP_TZVP_CPCM_chcl3_freq=0.20417005, B3LYP_TZVPD_CPCM_chcl3=-879.853026733945, M062X_TZVPD_SMD_chcl3=-879.921590885674, M062X_TZVPD=-879.898542633742, COSMORS=8.693095842297373e-05, wB97MV_TZVPD_CPCM_chcl3=-879.9737328193, wB97MV_TZVPD_SMD_chcl3=-879.978401576177, wB97MV_TZVPD=-879.955510317081, M062X_TZVPD_CPCM_chcl3=-879.917130742343
C 2.190238000000000 0.155092000000000 -0.031678000000000
C 1.112226000000000 0.944867000000000 -0.111637000000000
C -0.268023000000000 0.487410000000000 -0.178393000000000
C -1.243789000000000 1.418506000000000 -0.150734000000000
C -0.544601000000000 -0.957091000000000 -0.209287000000000
C -0.228315000000000 -1.833702000000000 -1.170344000000000
C -1.210733000000000 -1.670923000000000 0.909046000000000
O -1.572013000000000 -1.186433000000000 1.961278000000000
C -0.665892000000000 -3.234759000000000 -0.869366000000000
C -1.332782000000000 -3.132528000000000 0.507816000000000
C 5.840761000000000 0.194099000000000 0.220397000000000
O 4.469601000000000 -0.237784000000000 0.139925000000000
O 3.817326000000000 1.914138000000000 0.004991000000000
O -3.197375000000000 0.063996000000000 -0.497152000000000
C -4.825083000000000 2.122398000000000 -0.069356000000000
O -3.393116000000000 2.253728000000000 -0.012121000000000
C -2.684309000000000 1.132806000000000 -0.240356000000000
C 3.545155000000000 0.732275000000000 0.036364000000000
H 2.113766000000000 -0.922485000000000 -0.005830000000000
H 1.260710000000000 2.019196000000000 -0.117521000000000
H -0.975469000000000 2.463486000000000 -0.068723000000000
H 0.275369000000000 -1.574715000000000 -2.093117000000000
H -1.349022000000000 -3.594883000000000 -1.643108000000000
H 0.190403000000000 -3.914708000000000 -0.872838000000000
H -2.390872000000000 -3.399596000000000 0.487147000000000
H -0.855320000000000 -3.755374000000000 1.265424000000000
H 6.427664000000000 -0.715753000000000 0.304245000000000
H 5.986074000000000 0.826113000000000 1.095516000000000
H 6.115557000000000 0.746213000000000 -0.677494000000000
H -5.167566000000000 1.402185000000000 0.672572000000000
H -5.134950000000000 1.798036000000000 -1.062155000000000
H -5.216631000000000 3.111585000000000 0.148698000000000
41
R6_SMe6_P1SS_0_1 charge=0 multiplicity=1 B3LYP_D3BJ_TZVPD=-1357.823141433696, B3LYP_D3BJ_TZVPD_SMD_chcl3=-1357.848715150676, B3LYP_TZVP_CPCM_chcl3_freq=0.28176477, B3LYP_TZVPD_CPCM_chcl3=-1357.842496251858, M062X_TZVPD_SMD_chcl3=-1357.946387427387, M062X_TZVPD=-1357.920416224185, COSMORS=-0.002793965784518688, wB97MV_TZVPD_CPCM_chcl3=-1357.983612395112, wB97MV_TZVPD_SMD_chcl3=-1357.989976111999, wB97MV_TZVPD=-1357.964195050654, M062X_TZVPD_CPCM_chcl3=-1357.940268010112
O -4.639285000000000 -0.228117000000000 0.841785000000000
C -3.756462000000000 -1.016991000000000 0.204172000000000
O -4.076352000000000 -2.000005000000000 -0.430508000000000
H -6.572733000000000 0.135099000000000 1.309899000000000
H -6.174053000000000 -1.601978000000000 1.161397000000000
C -2.383792000000000 -0.511539000000000 0.385011000000000
C -1.344182000000000 -1.147911000000000 -0.168343000000000
C -6.023940000000000 -0.608832000000000 0.739999000000000
H -6.340667000000000 -0.603796000000000 -0.302184000000000
H -1.556664000000000 -2.049040000000000 -0.733656000000000
H -2.282095000000000 0.378785000000000 0.988024000000000
O 2.976572000000000 -2.701467000000000 -0.953459000000000
C 0.059646000000000 -0.783029000000000 -0.061789000000000
O 3.060298000000000 -0.686815000000000 0.041780000000000
C 4.414791000000000 -2.750974000000000 -0.931750000000000
H 4.829532000000000 -1.920918000000000 -1.502422000000000
H 4.777912000000000 -2.705474000000000 0.094156000000000
H 4.679391000000000 -3.699434000000000 -1.389529000000000
C 0.947966000000000 -1.697195000000000 -0.503785000000000
C 0.447531000000000 0.559110000000000 0.510546000000000
C 2.410927000000000 -1.601604000000000 -0.427233000000000

H -1.917100000000000 2.344895000000000 -0.364581000000000
C 1.028992000000000 1.504720000000000 -0.565930000000000
C 1.289974000000000 0.428518000000000 1.787671000000000
O 1.207938000000000 -0.560222000000000 2.484476000000000
C 1.575363000000000 2.791305000000000 0.040792000000000
H 2.838308000000000 1.310196000000000 2.925840000000000
H 1.360624000000000 2.253092000000000 2.796984000000000
C 2.645523000000000 2.479090000000000 1.083974000000000
H 1.986955000000000 3.410711000000000 -0.757570000000000
H 0.766750000000000 3.360310000000000 0.508573000000000
C 2.071832000000000 1.642285000000000 2.225292000000000
H 3.468866000000000 1.939090000000000 0.612255000000000
H 3.055070000000000 3.406925000000000 1.487637000000000
H -0.452553000000000 1.050578000000000 0.891138000000000
H 1.845287000000000 0.992834000000000 -1.072166000000000
S -0.143793000000000 1.790044000000000 -1.945044000000000
C -1.423091000000000 2.837710000000000 -1.200719000000000
H -1.019596000000000 3.796709000000000 -0.881832000000000
H -2.159291000000000 3.006802000000000 -1.985677000000000
H 0.575792000000000 -2.616735000000000 -0.935310000000000
35
R6_rot_barrier1_0_1 charge=0 multiplicity=1 B3LYP_D3BJ_TZVPD=-919.116842576425, B3LYP_D3BJ_TZVPD_SMD_chcl3=-919.142204805122, B3LYP_TZVP_CPCM_chcl3_freq=0.23367003, B3LYP_TZVPD_CPCM_chcl3=-919.136032151233, M062X_TZVPD_SMD_chcl3=-919.2119354487, M062X_TZVPD=-919.186073234449, COSMORS=-0.002305112607509863, wB97MV_TZVPD_CPCM_chcl3=-919.2588050955, wB97MV_TZVPD_SMD_chcl3=-919.265157994121, wB97MV_TZVPD=-919.239421792546, M062X_TZVPD_CPCM_chcl3=-919.205794633201
C 2.176098000000000 0.337586000000000 0.040861000000000
C 1.228924000000000 0.776226000000000 -0.793391000000000
C -0.213929000000000 0.444237000000000 -0.789124000000000
C -0.977279000000000 1.541992000000000 -0.970940000000000
C -0.641198000000000 -0.958494000000000 -0.561231000000000
C 0.261052000000000 -1.967546000000000 -0.564496000000000
C -2.074550000000000 -1.353103000000000 -0.529515000000000
O -2.962894000000000 -0.596632000000000 -0.882699000000000
H -4.450649000000000 2.091992000000000 1.614565000000000
C -2.429990000000000 -2.780374000000000 -0.162755000000000
C 5.692797000000000 0.783578000000000 0.948637000000000
O 4.323845000000000 0.340075000000000 0.917548000000000
O 3.950105000000000 1.683674000000000 -0.851001000000000
O -3.089502000000000 2.170277000000000 -1.847134000000000
C -4.276317000000000 2.130033000000000 0.543186000000000
O -2.880907000000000 1.817297000000000 0.368615000000000
C -2.436245000000000 1.810090000000000 -0.894171000000000
C 3.545861000000000 0.874451000000000 -0.042095000000000
H 1.989029000000000 -0.388323000000000 0.818972000000000
H 1.522667000000000 1.545275000000000 -1.500026000000000
H -0.446336000000000 2.461442000000000 -1.201724000000000
C -0.037706000000000 -3.396844000000000 -0.268928000000000
H -4.494564000000000 3.123700000000000 0.154556000000000
H -4.888423000000000 1.391017000000000 0.028388000000000
C -1.318781000000000 -3.541325000000000 0.539143000000000
H -2.685068000000000 -3.275952000000000 -1.107421000000000
H 6.191364000000000 0.536991000000000 0.012024000000000
H 6.152198000000000 0.251931000000000 1.776834000000000
H 5.738135000000000 1.859506000000000 1.112500000000000
H 1.291734000000000 -1.746164000000000 -0.799489000000000
H -3.348438000000000 -2.740696000000000 0.424656000000000
H -0.118226000000000 -3.946303000000000 -1.217238000000000
H 0.819810000000000 -3.831528000000000 0.250953000000000
H -1.159754000000000 -3.128224000000000 1.539202000000000
H -1.589120000000000 -4.591189000000000 0.659363000000000
44
R5_SMe6_P1_SMe2SR charge=0 multiplicity=1 B3LYP_D3BJ_TZVPD=-1757.200864043056, B3LYP_D3BJ_TZVPD_SMD_chcl3=-1757.226159363397, B3LYP_TZVP_CPCM_chcl3_freq=0.30159827, B3LYP_TZVPD_CPCM_chcl3=-1757.220204900241, M062X_TZVPD_SMD_chcl3=-1757.350465711746, M062X_TZVPD=-1757.324419386469, COSMORS=-0.004441733735018607, wB97MV_TZVPD_CPCM_chcl3=-1757.382722937343, wB97MV_TZVPD_SMD_chcl3=-1757.388825167414, wB97MV_TZVPD=-1757.363066047183, M062X_TZVPD_CPCM_chcl3=-1757.344637113416
C 2.815569000000000 -3.391517000000000 1.691099000000000
C -3.755782000000000 -2.423889000000000 -1.841953000000000
C 3.119232000000000 3.081391000000000 0.305303000000000
C -0.112786000000000 2.424677000000000 2.649990000000000
C 1.533296000000000 -0.067669000000000 -2.782448000000000
C 1.644353000000000 1.423725000000000 -2.458132000000000
C 0.191256000000000 -0.895491000000000 1.246457000000000
C -2.850828000000000 0.579260000000000 0.121859000000000
C -0.383438000000000 0.047903000000000 0.488558000000000
C 0.413385000000000 -0.578511000000000 -1.899250000000000
C 1.233390000000000 1.545613000000000 -0.976358000000000
C -1.625747000000000 0.708657000000000 1.035843000000000
C 1.383233000000000 -1.681264000000000 0.909661000000000
C -3.177373000000000 -0.868422000000000 -0.164230000000000
C 0.091742000000000 0.502068000000000 -0.859502000000000
O -0.210531000000000 -1.601259000000000 -2.050382000000000
O 2.049312000000000 -1.597099000000000 -0.102069000000000
O -3.195274000000000 -1.741207000000000 0.672709000000000
O 1.666864000000000 -2.549822000000000 1.896983000000000
O -3.457304000000000 -1.066952000000000 -1.454828000000000
S 2.639580000000000 1.335663000000000 0.181788000000000
S -1.355074000000000 2.501480000000000 1.330895000000000
H 2.884631000000000 -4.011819000000000 2.579839000000000
H 3.712747000000000 -2.784437000000000 1.576349000000000
H 2.677517000000000 -4.007804000000000 0.803589000000000
H -4.634656000000000 -2.782844000000000 -1.308614000000000
H -2.902882000000000 -3.064498000000000 -1.625772000000000
H -3.944316000000000 -2.385093000000000 -2.910467000000000
H 2.307121000000000 3.674374000000000 0.726619000000000
H 3.403707000000000 3.480658000000000 -0.668060000000000
H 3.980189000000000 3.125497000000000 0.971287000000000
H 0.798988000000000 1.949412000000000 2.292419000000000
H -0.507490000000000 1.890414000000000 3.513414000000000
H 0.100551000000000 3.455486000000000 2.929593000000000
H 2.437232000000000 -0.602137000000000 -2.481385000000000
H 1.337400000000000 -0.294401000000000 -3.829640000000000
H 0.934743000000000 1.991911000000000 -3.066221000000000
H 2.638059000000000 1.828796000000000 -2.638283000000000
H -0.252794000000000 -1.136210000000000 2.202941000000000
H -3.711572000000000 1.012599000000000 0.636966000000000
H -2.725204000000000 1.122325000000000 -0.812752000000000
H 0.834837000000000 2.530946000000000 -0.750209000000000
H -1.867024000000000 0.260370000000000 1.997742000000000
H -0.749359000000000 1.019889000000000 -1.337284000000000
37
R5_SMe6_cyc4SZ_-1_1 charge=-1 multiplicity=1 wB97MV_TZVPD_SMD_chcl3=-1318.158096747922, B3LYP_TZVP_CPCM_chcl3_freq=0.24124236, B3LYP_TZVPD_CPCM_chcl3=-1318.007746117885, M062X_TZVPD=-1318.040662067655, COSMORS=-0.009002302329241636, M062X_TZVPD_CPCM_chcl3=-1318.112798380908, M062X_TZVPD_SMD_chcl3=-1318.114954770189, wB97MV_TZVPD_CPCM_chcl3=-1318.155735767233, B3LYP_D3BJ_TZVPD_SMD_chcl3=-1318.009794062458, wB97MV_TZVPD=-1318.084889261242
C -2.189626000000000 0.446543000000000 0.143625000000000
C -0.844503000000000 0.612516000000000 -0.246979000000000
C 0.228979000000000 -0.190070000000000 0.006210000000000
C 0.235066000000000 -1.495552000000000 0.812952000000000
C 1.625434000000000 0.008095000000000 -0.293865000000000
C 2.503247000000000 1.011557000000000 -0.950774000000000
C 2.356600000000000 -0.900043000000000 0.358676000000000
O 1.628581000000000 -1.789333000000000 1.107712000000000
C 3.925313000000000 0.379230000000000 -0.793648000000000
C 3.826729000000000 -0.745796000000000 0.278776000000000
C -5.487712000000000 1.951097000000000 0.058313000000000
O -4.430450000000000 1.038529000000000 0.322330000000000
O -3.056235000000000 2.472321000000000 -0.789607000000000
O 0.320561000000000 -3.305928000000000 -0.809649000000000
C -2.326240000000000 -3.681857000000000 -0.712347000000000
O -1.638009000000000 -2.761334000000000 0.153240000000000
C -0.318879000000000 -2.635075000000000 -0.033094000000000
C -3.170527000000000 1.404279000000000 -0.166658000000000
H -2.504723000000000 -0.435830000000000 0.681028000000000
H -0.627195000000000 1.515600000000000 -0.811102000000000
H -0.322726000000000 -1.437599000000000 1.750938000000000
H 2.264312000000000 1.194493000000000 -2.000372000000000
H 4.220095000000000 -0.055381000000000 -1.749934000000000
H 4.667120000000000 1.132942000000000 -0.533002000000000
H 4.348445000000000 -1.655063000000000 -0.034415000000000
H 4.259777000000000 -0.444559000000000 1.240585000000000
H -6.380476000000000 1.512955000000000 0.503248000000000
H -5.642141000000000 2.088616000000000 -1.014301000000000
H -5.296826000000000 2.928769000000000 0.506685000000000
H -1.948434000000000 -4.693153000000000 -0.566655000000000
H -2.194343000000000 -3.388563000000000 -1.753051000000000
H -3.373005000000000 -3.620006000000000 -0.429619000000000
S 2.337355000000000 2.709668000000000 -0.227235000000000
C 2.437816000000000 2.345923000000000 1.545674000000000
H 3.402324000000000 1.912061000000000 1.808062000000000
H 2.320952000000000 3.295841000000000 2.066007000000000
H 1.634946000000000 1.671557000000000 1.839584000000000
41
R6_SMe6H_cyc4RETS_0_1 charge=0 multiplicity=1 B3LYP_D3BJ_TZVPD=-1357.733946598371, B3LYP_D3BJ_TZVPD_SMD_chcl3=-1357.764233274483, B3LYP_TZVP_CPCM_chcl3_freq=0.28080744, B3LYP_TZVPD_CPCM_chcl3=-1357.758456531145, M062X_TZVPD_SMD_chcl3=-1357.851679768584, M062X_TZVPD=-1357.819801488496, COSMORS=-0.0023351679306236536, wB97MV_TZVPD_CPCM_chcl3=-1357.886403589074, wB97MV_TZVPD_SMD_chcl3=-1357.892302002472, wB97MV_TZVPD=-1357.860658955723, M062X_TZVPD_CPCM_chcl3=-1357.845955520888
O 3.902499000000000 2.543137000000000 -0.268713000000000
C 2.701415000000000 2.406303000000000 -0.811738000000000
C -2.868197000000000 1.219066000000000 -0.119785000000000
H 5.315826000000000 1.948369000000000 1.045039000000000
C 0.518218000000000 1.386707000000000 -1.111908000000000
C 1.795589000000000 1.417136000000000 -0.526827000000000
H 3.643855000000000 1.626565000000000 1.585911000000000
O 2.442002000000000 3.354748000000000 -1.709037000000000
C 4.331031000000000 1.608875000000000 0.740678000000000
H 4.389480000000000 0.605785000000000 0.319503000000000
H -3.873989000000000 1.931712000000000 2.248227000000000
H -1.898695000000000 1.733458000000000 -1.948412000000000
C -0.510306000000000 0.474742000000000 -0.865765000000000
H -3.161270000000000 0.477843000000000 3.007049000000000
C -1.781055000000000 1.060023000000000 -1.106080000000000
C -0.495389000000000 -0.890016000000000 -0.443308000000000
C 0.728160000000000 -1.614294000000000 0.022566000000000
H -4.463465000000000 0.315356000000000 1.792465000000000
S 0.935287000000000 -1.258361000000000 1.832882000000000
C -1.657687000000000 -1.551625000000000 -0.835351000000000
H -0.667749000000000 -4.793981000000000 -0.153936000000000
H 1.618823000000000 -1.196987000000000 -0.449448000000000
C -1.744488000000000 -3.059794000000000 -0.851518000000000
O -2.673786000000000 -0.913711000000000 -1.288715000000000
H 1.473633000000000 -3.629399000000000 0.268263000000000
C -0.677895000000000 -3.716020000000000 0.019788000000000
H -2.748615000000000 -3.347526000000000 -0.533951000000000
H -1.640669000000000 -3.385310000000000 -1.892943000000000
C 0.685692000000000 -3.113190000000000 -0.281654000000000
H -0.916747000000000 -3.562715000000000 1.075683000000000
H 0.909360000000000 -3.241562000000000 -1.346852000000000
C 2.624956000000000 -1.859543000000000 2.121894000000000
H 2.684784000000000 -2.944478000000000 2.048877000000000
H 3.317118000000000 -1.406322000000000 1.411422000000000
H 2.903314000000000 -1.557250000000000 3.130839000000000
O -2.555718000000000 0.788249000000000 1.106597000000000
O -3.927898000000000 1.742659000000000 -0.398642000000000
C -3.590325000000000 0.890438000000000 2.098470000000000
H 0.249209000000000 2.230926000000000 -1.738264000000000
H 2.062074000000000 0.693674000000000 0.223806000000000
H 3.181362000000000 3.981777000000000 -1.746113000000000
41
R6_SMe6_cyc4SEP1_0_1 charge=0 multiplicity=1 B3LYP_D3BJ_TZVPD_SMD_chcl3=-1357.842103082422, B3LYP_TZVP_CPCM_chcl3_freq=0.2825989, B3LYP_TZVPD_CPCM_chcl3=-1357.835528723879, M062X_TZVPD_SMD_chcl3=-1357.948390211697, M062X_TZVPD=-1357.924166756363, COSMORS=-0.0050278922158094085, wB97MV_TZVPD_CPCM_chcl3=-1357.983780659347, wB97MV_TZVPD_SMD_chcl3=-1357.990551846276, M062X_TZVPD_CPCM_chcl3=-1357.941814495379
O 3.333303000000000 2.718591000000000 -0.487793000000000
C 2.103853000000000 2.674226000000000 0.050855000000000
C -3.074364000000000 0.846740000000000 -0.011188000000000
H 3.676854000000000 4.775364000000000 -0.433678000000000
C -0.001005000000000 1.311677000000000 0.178704000000000
C 1.363255000000000 1.446079000000000 -0.419139000000000
H 4.317594000000000 3.861451000000000 0.952748000000000
O 1.679316000000000 3.519847000000000 0.803445000000000
C 4.152865000000000 3.845965000000000 -0.123788000000000
H 5.091974000000000 3.704726000000000 -0.650798000000000
H -3.518900000000000 2.935872000000000 -1.584106000000000
H -2.166763000000000 0.553155000000000 1.917549000000000
C -0.684219000000000 0.173508000000000 0.339788000000000
H -3.836983000000000 3.981742000000000 -0.167858000000000
C -2.103848000000000 0.129618000000000 0.916331000000000
C -0.394791000000000 -1.219119000000000 0.023587000000000
C 0.798363000000000 -1.829389000000000 -0.634319000000000
H -4.960429000000000 2.650927000000000 -0.576243000000000
S 2.183677000000000 -1.891580000000000 0.591627000000000
C -1.445346000000000 -1.965507000000000 0.410461000000000
H -0.488301000000000 -5.067726000000000 -0.578640000000000
H 1.156176000000000 -1.191235000000000 -1.443413000000000
C -1.623138000000000 -3.426130000000000 0.249944000000000
O -2.460687000000000 -1.259639000000000 0.980988000000000
H 1.357701000000000 -3.724137000000000 -1.516563000000000
C -0.298153000000000 -4.074923000000000 -0.169004000000000
H -2.001097000000000 -3.858464000000000 1.180117000000000
H -2.398260000000000 -3.595990000000000 -0.506560000000000
C 0.449128000000000 -3.216977000000000 -1.189694000000000
H 0.334196000000000 -4.205318000000000 0.712069000000000
H -0.174999000000000 -3.074048000000000 -2.078443000000000
C 3.601195000000000 -2.074167000000000 -0.526971000000000
H 3.658661000000000 -1.225538000000000 -1.209087000000000
H 4.494730000000000 -2.094129000000000 0.095552000000000
H 3.542046000000000 -3.002761000000000 -1.092929000000000
O -3.140786000000000 2.141141000000000 0.305128000000000
O -3.663113000000000 0.329139000000000 -0.926869000000000
C -3.922245000000000 2.978473000000000 -0.573570000000000
H -0.468950000000000 2.235915000000000 0.494084000000000
H 1.991682000000000 0.583220000000000 -0.187295000000000
H 1.323578000000000 1.505093000000000 -1.513464000000000
44
R5_SMe4_P3_SMe6SS charge=0 multiplicity=1 B3LYP_D3BJ_TZVPD=-1757.199244472317, B3LYP_D3BJ_TZVPD_SMD_chcl3=-1757.225191953998, B3LYP_TZVP_CPCM_chcl3_freq=0.30086815, B3LYP_TZVPD_CPCM_chcl3=-1757.219093661644, M062X_TZVPD_SMD_chcl3=-1757.352315384501, M062X_TZVPD=-1757.325731079642, COSMORS=-0.0035136680657832783, wB97MV_TZVPD_CPCM_chcl3=-1757.383390127266, wB97MV_TZVPD_SMD_chcl3=-1757.389670051873, wB97MV_TZVPD=-1757.36326577829, M062X_TZVPD_CPCM_chcl3=-1757.346281790765
C -5.282902000000000 -2.749235000000000 -1.369682000000000
C 2.672639000000000 -2.908750000000000 -0.670843000000000
C 3.099513000000000 0.652932000000000 -2.803942000000000
C 0.218397000000000 1.091465000000000 3.641597000000000
C -1.397180000000000 -0.762987000000000 0.787756000000000
C 0.330388000000000 3.561459000000000 -0.163494000000000
C 0.984707000000000 2.933046000000000 -1.397892000000000
C -2.708571000000000 -0.546889000000000 0.105717000000000
C -0.273197000000000 -0.065136000000000 0.613049000000000
C -0.549177000000000 2.465180000000000 0.403222000000000
C 1.117876000000000 1.449364000000000 -1.021234000000000
C -3.413019000000000 -1.839986000000000 -0.241837000000000
C -0.193067000000000 1.135719000000000 -0.287511000000000
C 0.931544000000000 -0.437180000000000 1.453004000000000
C 2.078382000000000 -1.054229000000000 0.658649000000000
O -1.433583000000000 2.606129000000000 1.213761000000000
O -3.073244000000000 -2.946169000000000 0.106976000000000
O 3.180509000000000 -0.576641000000000 0.526698000000000
O -4.498617000000000 -1.600570000000000 -0.990340000000000
O 1.706451000000000 -2.230196000000000 0.155709000000000
S 1.365144000000000 0.300718000000000 -2.407088000000000
S 1.579526000000000 0.955479000000000 2.452447000000000
H -5.660887000000000 -3.256965000000000 -0.483417000000000
H -4.680119000000000 -3.439601000000000 -1.957919000000000
H -6.102943000000000 -2.356882000000000 -1.963728000000000
H 2.192910000000000 -3.833930000000000 -0.975240000000000
H 3.578412000000000 -3.113041000000000 -0.102367000000000
H 2.906521000000000 -2.293889000000000 -1.538308000000000
H 3.385519000000000 -0.029693000000000 -3.602881000000000
H 3.723509000000000 0.467841000000000 -1.930249000000000
H 3.225788000000000 1.678172000000000 -3.147976000000000
H 0.490914000000000 1.896524000000000 4.322732000000000
H 0.109951000000000 0.164845000000000 4.204401000000000
H -0.712891000000000 1.344073000000000 3.139056000000000
H -1.387214000000000 -1.577142000000000 1.503032000000000
H -0.249115000000000 4.463694000000000 -0.357521000000000
H 1.074798000000000 3.800651000000000 0.601337000000000
H 1.945434000000000 3.380214000000000 -1.645944000000000
H 0.329406000000000 3.030565000000000 -2.266026000000000
H -2.628225000000000 0.049204000000000 -0.802895000000000
H -3.388776000000000 0.010841000000000 0.760587000000000
H 1.965380000000000 1.331950000000000 -0.348878000000000
H -0.971499000000000 1.049436000000000 -1.055567000000000
H 0.616648000000000 -1.210739000000000 2.154048000000000
55
Sulf_SMe4_P1SS_0_1 charge=0 multiplicity=1 B3LYP_D3BJ_TZVPD=-2138.403127495256, B3LYP_D3BJ_TZVPD_SMD_chcl3=-2138.438751171194, B3LYP_TZVP_CPCM_chcl3_freq=0.37049867, B3LYP_TZVPD_CPCM_chcl3=-2138.428835399594, M062X_TZVPD_SMD_chcl3=-2138.591439192871, M062X_TZVPD=-2138.554493059769, COSMORS=-0.006916883615924095, wB97MV_TZVPD_CPCM_chcl3=-2138.657285545656, wB97MV_TZVPD_SMD_chcl3=-2138.667617782945, wB97MV_TZVPD=-2138.631263106447, M062X_TZVPD_CPCM_chcl3=-2138.581252614048
C -3.274731000000000 1.950432000000000 -0.322782000000000
C -3.838857000000000 2.618374000000000 0.752990000000000
C -4.575338000000000 1.913614000000000 1.701980000000000
C -4.749798000000000 0.540271000000000 1.578786000000000
C -4.181888000000000 -0.140288000000000 0.509754000000000
C -3.451069000000000 0.574504000000000 -0.427777000000000
O -3.055026000000000 0.447029000000000 -3.012677000000000
O -3.065998000000000 -1.677950000000000 -1.714308000000000
S -2.716308000000000 -0.274031000000000 -1.799017000000000
C 0.854564000000000 3.142523000000000 -1.815360000000000
S 0.712553000000000 2.881833000000000 -0.027481000000000
C -1.027166000000000 0.356897000000000 1.856564000000000
C -1.556856000000000 -0.075740000000000 3.063740000000000
C -1.888794000000000 -1.413674000000000 3.245073000000000
C -1.695899000000000 -2.317798000000000 2.207646000000000
C -1.175925000000000 -1.885579000000000 0.994715000000000
C -0.836912000000000 -0.544590000000000 0.808920000000000
C 5.130982000000000 -4.622683000000000 -0.884419000000000
O 4.371505000000000 -3.463738000000000 -1.276106000000000
O 3.454476000000000 -3.388755000000000 0.779064000000000
O 3.663503000000000 1.653202000000000 -1.085222000000000
C 5.139537000000000 2.417652000000000 1.005113000000000
O 3.761925000000000 2.008241000000000 1.134003000000000
C 3.136908000000000 1.669800000000000 0.004002000000000
C 3.562239000000000 -2.938032000000000 -0.341613000000000
C 1.678088000000000 1.348311000000000 0.251534000000000
C -0.970879000000000 -0.062702000000000 -1.623462000000000
C -0.263985000000000 -0.130282000000000 -0.497148000000000
C 1.233328000000000 0.129810000000000 -0.583856000000000
C 1.988053000000000 -1.081677000000000 -0.116712000000000
C 2.845231000000000 -1.760614000000000 -0.872722000000000
H -2.695483000000000 2.485854000000000 -1.062265000000000
H -3.702575000000000 3.687095000000000 0.853054000000000

H -5.010712000000000 2.438631000000000 2.542543000000000
H -5.315909000000000 -0.006202000000000 2.321290000000000
H -4.286635000000000 -1.210216000000000 0.408829000000000
H 0.297158000000000 2.393039000000000 -2.372387000000000
H 0.414192000000000 4.122015000000000 -1.997691000000000
H 1.897089000000000 3.151475000000000 -2.125083000000000
H -0.807573000000000 1.404218000000000 1.710944000000000
H -1.721469000000000 0.638106000000000 3.860439000000000
H -2.302340000000000 -1.748076000000000 4.187942000000000
H -1.955493000000000 -3.360574000000000 2.337449000000000
H -1.036223000000000 -2.585104000000000 0.182307000000000
H 4.461630000000000 -5.434263000000000 -0.602054000000000
H 5.783418000000000 -4.380220000000000 -0.046592000000000
H 5.715708000000000 -4.895969000000000 -1.757746000000000
H 5.208219000000000 3.301609000000000 0.372784000000000
H 5.731625000000000 1.610974000000000 0.576180000000000
H 5.469781000000000 2.641540000000000 2.014772000000000
H 1.538649000000000 1.153705000000000 1.313409000000000
H -0.520033000000000 0.178138000000000 -2.578095000000000
H 1.486649000000000 0.324811000000000 -1.624324000000000
H 1.817058000000000 -1.402529000000000 0.905456000000000
H 3.051280000000000 -1.477219000000000 -1.896919000000000
38
R5_SMe6_cyc2P2_0_1 charge=0 multiplicity=1 B3LYP_D3BJ_TZVPD=-1318.518398699156, wB97MV_TZVPD=-1318.669913878127, B3LYP_D3BJ_TZVPD_SMD_chcl3=-1318.541363730752, B3LYP_TZVP_CPCM_chcl3_freq=0.25417845, B3LYP_TZVPD_CPCM_chcl3=-1318.535384292813, M062X_TZVPD_SMD_chcl3=-1318.646881537087, M062X_TZVPD=-1318.623154009643, COSMORS=-0.0031914737271222914, wB97MV_TZVPD_CPCM_chcl3=-1318.687322556548, wB97MV_TZVPD_SMD_chcl3=-1318.693490820551, M062X_TZVPD_CPCM_chcl3=-1318.640905225073
C -2.499233000000000 0.538100000000000 -0.865440000000000
C -1.142972000000000 -0.050851000000000 -0.827497000000000
C 0.036245000000000 0.357993000000000 -0.280788000000000
C 0.286816000000000 1.597500000000000 0.504222000000000
C 0.970781000000000 -0.685880000000000 -0.579092000000000
C 2.368208000000000 -1.112852000000000 -0.308662000000000
C 0.294674000000000 -1.633668000000000 -1.250997000000000
O -1.002479000000000 -1.287723000000000 -1.430923000000000
C 2.483394000000000 -2.434599000000000 -1.146963000000000
C 1.047986000000000 -2.863463000000000 -1.594513000000000
C -5.723759000000000 -0.144137000000000 0.816387000000000
O -4.710988000000000 0.334486000000000 -0.092516000000000
O -3.148570000000000 -0.807478000000000 1.050349000000000
O 1.918803000000000 2.320656000000000 -1.144034000000000
C 2.519446000000000 4.527672000000000 0.221695000000000
O 1.539062000000000 3.575722000000000 0.679762000000000
C 1.333893000000000 2.507224000000000 -0.103794000000000
C -3.457693000000000 -0.072023000000000 0.144239000000000
H -2.942265000000000 0.443442000000000 -1.859380000000000
H -0.625125000000000 2.182562000000000 0.636134000000000
H 0.632184000000000 1.347040000000000 1.511786000000000
H 3.115987000000000 -0.383335000000000 -0.620090000000000
H 3.091241000000000 -2.230459000000000 -2.028305000000000
H 2.981308000000000 -3.218881000000000 -0.580204000000000
H 1.015189000000000 -3.116178000000000 -2.656215000000000
H 0.684098000000000 -3.732946000000000 -1.039293000000000
H -5.501951000000000 0.181204000000000 1.831691000000000
H -5.774940000000000 -1.231343000000000 0.782401000000000
H -6.654864000000000 0.293534000000000 0.469018000000000
H 3.494610000000000 4.050867000000000 0.133440000000000
H 2.542729000000000 5.306994000000000 0.977666000000000
H 2.224508000000000 4.938699000000000 -0.742800000000000
S 2.723120000000000 -1.296690000000000 1.490007000000000
C 1.395114000000000 -2.422572000000000 1.996410000000000
H 0.417960000000000 -1.986429000000000 1.794678000000000
H 1.482835000000000 -3.385142000000000 1.493572000000000
H 1.507522000000000 -2.571729000000000 3.069390000000000
H -2.448397000000000 1.608551000000000 -0.654677000000000
38
R5_SMe6H_cyc4RZ_0_1 charge=0 multiplicity=1 B3LYP_D3BJ_TZVPD_SMD_chcl3=-1318.489686499365, B3LYP_TZVP_CPCM_chcl3_freq=0.25450283, B3LYP_TZVPD_CPCM_chcl3=-1318.483396816747, M062X_TZVPD_SMD_chcl3=-1318.593062428463, M062X_TZVPD=-1318.569080000024, COSMORS=-0.003046838460642565, wB97MV_TZVPD_CPCM_chcl3=-1318.627803968809, wB97MV_TZVPD_SMD_chcl3=-1318.634270385052, wB97MV_TZVPD=-1318.610558098411, M062X_TZVPD_CPCM_chcl3=-1318.586741256764
C -2.437101000000000 -0.282956000000000 -0.607568000000000
C -1.200053000000000 0.444840000000000 -0.584495000000000
C 0.030192000000000 -0.057003000000000 -0.816638000000000
C 0.395019000000000 -1.516463000000000 -1.134398000000000
C 1.305210000000000 0.613358000000000 -0.840442000000000
C 1.883823000000000 1.945308000000000 -0.527108000000000
C 2.252816000000000 -0.259151000000000 -1.192305000000000
O 1.816195000000000 -1.522275000000000 -1.444539000000000
C 3.375033000000000 1.782163000000000 -0.979670000000000
C 3.637256000000000 0.265256000000000 -1.225957000000000
C -4.950587000000000 2.101277000000000 0.459030000000000
O -3.779067000000000 1.610894000000000 -0.215245000000000
O -4.819535000000000 -0.375793000000000 -0.366115000000000
O -0.890365000000000 -3.075979000000000 0.171157000000000
C 0.843678000000000 -3.074387000000000 2.209105000000000
O 1.066560000000000 -2.332732000000000 0.992408000000000
C 0.115994000000000 -2.414183000000000 0.065288000000000
C -3.642886000000000 0.290537000000000 -0.391100000000000
H -2.428630000000000 -1.346439000000000 -0.805549000000000
H -1.258752000000000 1.503494000000000 -0.359677000000000
H -0.144691000000000 -1.926006000000000 -1.987919000000000
H 1.399030000000000 2.768557000000000 -1.054617000000000
H 3.518336000000000 2.333156000000000 -1.909197000000000
H 4.059031000000000 2.199606000000000 -0.243209000000000
H 4.140686000000000 0.082477000000000 -2.178284000000000
H 4.253420000000000 -0.188303000000000 -0.443107000000000
H -4.782305000000000 3.167544000000000 0.582634000000000
H -5.060340000000000 1.625232000000000 1.434149000000000
H -5.844865000000000 1.931515000000000 -0.138685000000000
H 1.711955000000000 -2.876711000000000 2.830033000000000
H -0.065686000000000 -2.726115000000000 2.696309000000000
H 0.761154000000000 -4.137759000000000 1.990365000000000
S 1.660716000000000 2.437498000000000 1.238889000000000
C 2.355849000000000 0.997491000000000 2.093909000000000
H 3.417458000000000 0.883830000000000 1.875694000000000
H 2.231637000000000 1.181530000000000 3.160269000000000
H 1.821944000000000 0.089741000000000 1.819419000000000
H -4.656674000000000 -1.326169000000000 -0.439143000000000
37
R5_SMe6_cyc4SZTS_-1_1 charge=-1 multiplicity=1 wB97MV_TZVPD_SMD_chcl3=-1318.123646612762, B3LYP_TZVP_CPCM_chcl3_freq=0.23854478, B3LYP_TZVPD_CPCM_chcl3=-1317.983548189386, M062X_TZVPD=-1318.008179499055, COSMORS=-0.009569704121111477, M062X_TZVPD_CPCM_chcl3=-1318.076275867582, M062X_TZVPD_SMD_chcl3=-1318.078522231835, wB97MV_TZVPD_CPCM_chcl3=-1318.121153433046, B3LYP_D3BJ_TZVPD_SMD_chcl3=-1317.98564211113, wB97MV_TZVPD=-1318.054358386968
C -2.174228000000000 -0.358353000000000 0.077106000000000
C -0.981691000000000 0.289369000000000 -0.216800000000000
C 0.298568000000000 -0.013455000000000 0.225191000000000
C 0.716441000000000 -1.228163000000000 0.886383000000000
C 1.497124000000000 0.733960000000000 -0.094731000000000
C 1.785698000000000 2.083108000000000 -0.632358000000000
C 2.577489000000000 -0.085128000000000 -0.143221000000000
O 2.475912000000000 -1.331369000000000 0.264458000000000
C 3.210821000000000 1.926697000000000 -1.250696000000000
C 3.764633000000000 0.548202000000000 -0.802509000000000
C -5.739368000000000 -0.416374000000000 -0.613898000000000
O -4.458060000000000 -0.742734000000000 -0.080892000000000
O -3.609272000000000 0.970255000000000 -1.299691000000000
O -0.247495000000000 -3.313306000000000 1.540358000000000
C -0.890623000000000 -3.977388000000000 -0.955331000000000
O -0.155748000000000 -2.784817000000000 -0.647430000000000
C 0.041801000000000 -2.530623000000000 0.653579000000000
C -3.404862000000000 0.046014000000000 -0.508773000000000
H -2.211869000000000 -1.177744000000000 0.781862000000000
H -1.070882000000000 1.183889000000000 -0.828430000000000
H 1.101609000000000 -1.169846000000000 1.898209000000000
H 1.066434000000000 2.422893000000000 -1.380680000000000
H 3.123823000000000 1.954772000000000 -2.337628000000000
H 3.863079000000000 2.749556000000000 -0.959530000000000
H 4.115383000000000 -0.051488000000000 -1.647564000000000
H 4.607102000000000 0.637755000000000 -0.108645000000000
H -5.748840000000000 -0.502422000000000 -1.701976000000000
H -6.035190000000000 0.598162000000000 -0.341010000000000
H -6.433711000000000 -1.133470000000000 -0.179846000000000
H -0.928928000000000 -4.025832000000000 -2.039934000000000
H -1.898179000000000 -3.910772000000000 -0.543076000000000
H -0.388059000000000 -4.855109000000000 -0.550265000000000
S 1.653239000000000 3.444822000000000 0.632406000000000
C 2.778592000000000 2.825980000000000 1.910386000000000
H 2.477499000000000 1.828105000000000 2.226599000000000
H 3.808438000000000 2.801386000000000 1.553437000000000
H 2.710442000000000 3.510824000000000 2.754704000000000
38
R5_SMe6H_cyc4STS_0_1 charge=0 multiplicity=1 B3LYP_D3BJ_TZVPD=-1318.425752502258, B3LYP_D3BJ_TZVPD_SMD_chcl3=-1318.452775622118, B3LYP_TZVP_CPCM_chcl3_freq=0.2522792, B3LYP_TZVPD_CPCM_chcl3=-1318.447409078764, M062X_TZVPD_SMD_chcl3=-1318.537136078114, M062X_TZVPD=-1318.509064028508, COSMORS=-0.0018163709826016722, wB97MV_TZVPD_CPCM_chcl3=-1318.574619020073, wB97MV_TZVPD_SMD_chcl3=-1318.58025684296, wB97MV_TZVPD=-1318.552465180232, M062X_TZVPD_CPCM_chcl3=-1318.531653314715
C 2.025927000000000 -0.115283000000000 0.226367000000000
C 0.790515000000000 -0.749738000000000 0.028902000000000
C -0.445344000000000 -0.134487000000000 -0.090597000000000
C -1.582663000000000 -0.909991000000000 -0.482703000000000
C -0.849919000000000 1.212419000000000 0.202612000000000
C -0.134633000000000 2.478187000000000 0.542866000000000
C -2.152618000000000 1.231163000000000 0.656818000000000
O -2.940257000000000 0.222122000000000 0.559138000000000
C -1.278491000000000 3.415314000000000 1.018491000000000
C -2.448272000000000 2.496043000000000 1.422715000000000
C 4.524344000000000 -2.804485000000000 0.208951000000000
O 3.254901000000000 -2.119736000000000 0.148324000000000
O 4.397492000000000 -0.230516000000000 0.469649000000000
O -1.184251000000000 -2.905224000000000 0.787349000000000
C -3.038422000000000 -4.262773000000000 -0.555819000000000
O -2.712553000000000 -2.897801000000000 -0.870923000000000
C -1.765944000000000 -2.323019000000000 -0.106505000000000
C 3.211346000000000 -0.808511000000000 0.279517000000000
H 2.089831000000000 0.959112000000000 0.310124000000000
H 0.810419000000000 -1.822199000000000 -0.106464000000000
H -2.163713000000000 -0.586871000000000 -1.332896000000000
H 0.604715000000000 2.344154000000000 1.338887000000000
H -0.949016000000000 4.066012000000000 1.827312000000000
H -1.587891000000000 4.054258000000000 0.191435000000000
H -2.444732000000000 2.272438000000000 2.494549000000000
H -3.428236000000000 2.909611000000000 1.178507000000000
H 4.995038000000000 -2.644655000000000 1.177371000000000
H 5.178897000000000 -2.460311000000000 -0.589949000000000
H 4.280406000000000 -3.853448000000000 0.074417000000000
H -2.167405000000000 -4.902146000000000 -0.695887000000000
H -3.830194000000000 -4.540245000000000 -1.245458000000000
H -3.382995000000000 -4.339116000000000 0.474942000000000
S 0.890295000000000 3.207750000000000 -0.807846000000000
C -0.251379000000000 3.110918000000000 -2.211558000000000
H -0.534235000000000 2.077460000000000 -2.404256000000000
H -1.144422000000000 3.710637000000000 -2.038702000000000
H 0.282693000000000 3.509609000000000 -3.073133000000000
H 4.295534000000000 0.729624000000000 0.549391000000000
38
R5_SMe6_cyc2SP1_0_1 charge=0 multiplicity=1 B3LYP_D3BJ_TZVPD=-1318.511104922615, B3LYP_D3BJ_TZVPD_SMD_chcl3=-1318.534969944479, B3LYP_TZVP_CPCM_chcl3_freq=0.25557268, B3LYP_TZVPD_CPCM_chcl3=-1318.528799026388, M062X_TZVPD_SMD_chcl3=-1318.63591089898, M062X_TZVPD=-1318.611624864322, COSMORS=-0.003580169053775775, wB97MV_TZVPD_CPCM_chcl3=-1318.675336314414, wB97MV_TZVPD_SMD_chcl3=-1318.681655332476, wB97MV_TZVPD=-1318.6575254331, M062X_TZVPD_CPCM_chcl3=-1318.629832340494
C -2.372943000000000 0.803311000000000 -0.264517000000000
C -1.314759000000000 0.052832000000000 0.517979000000000
C 0.112450000000000 0.409540000000000 0.107728000000000
C 0.601216000000000 1.662387000000000 0.250652000000000
C 0.660754000000000 -0.816394000000000 -0.381768000000000
C 1.961789000000000 -1.421628000000000 -0.790644000000000
C -0.290805000000000 -1.768709000000000 -0.267790000000000
O -1.463566000000000 -1.382461000000000 0.238334000000000
C 1.537576000000000 -2.851242000000000 -1.255289000000000
C 0.124750000000000 -3.132477000000000 -0.668760000000000
C -6.056549000000000 0.518590000000000 -0.394980000000000
O -4.671475000000000 0.712251000000000 -0.751249000000000
O -4.059798000000000 0.153960000000000 1.338162000000000
O 2.607413000000000 1.540555000000000 -1.051329000000000
C 3.437909000000000 3.903809000000000 -0.196778000000000
O 2.205483000000000 3.327812000000000 0.261077000000000
C 1.888619000000000 2.112418000000000 -0.252528000000000
C -3.773528000000000 0.504575000000000 0.217425000000000
H -2.208494000000000 1.877253000000000 -0.138497000000000
H -1.473344000000000 0.170312000000000 1.589783000000000
H 0.001560000000000 2.411122000000000 0.751552000000000
H 2.470083000000000 -0.870819000000000 -1.576794000000000
H 1.482434000000000 -2.855521000000000 -2.344209000000000
H 2.263793000000000 -3.605026000000000 -0.958697000000000
H -0.554581000000000 -3.576454000000000 -1.398850000000000
H 0.146119000000000 -3.798333000000000 0.198624000000000
H -6.619849000000000 0.730843000000000 -1.298584000000000
H -6.338304000000000 1.203682000000000 0.403182000000000
H -6.220263000000000 -0.508377000000000 -0.072398000000000
H 3.417074000000000 4.043824000000000 -1.277500000000000
H 4.280250000000000 3.264803000000000 0.067023000000000
H 3.518191000000000 4.862953000000000 0.307668000000000
S 3.209045000000000 -1.400143000000000 0.567005000000000
C 2.365870000000000 -2.349857000000000 1.860711000000000
H 3.012142000000000 -2.314500000000000 2.736710000000000
H 1.406936000000000 -1.897800000000000 2.111956000000000
H 2.223032000000000 -3.389742000000000 1.569211000000000
H -2.285271000000000 0.588029000000000 -1.329267000000000
38
R5_SMe1_P1A_0_1 charge=0 multiplicity=1 B3LYP_D3BJ_TZVPD=-1318.520824330885, CCSDT_SMD_chcl3=-1316.778200487322, CCSDT=-1316.755620504249, B3LYP_D3BJ_TZVPD_SMD_chcl3=-1318.544735241969, B3LYP_TZVP_CPCM_chcl3_freq=0.25270732, B3LYP_TZVPD_CPCM_chcl3=-1318.538928873027, M062X_TZVPD_SMD_chcl3=-1318.639080166814, M062X_TZVPD=-1318.614783324924, COSMORS=-0.003108176179977125, wB97MV_TZVPD_CPCM_chcl3=-1318.679619399156, wB97MV_TZVPD_SMD_chcl3=-1318.685633944098, wB97MV_TZVPD=-1318.661415825227, M062X_TZVPD_CPCM_chcl3=-1318.633273582672
O 3.700754000000000 0.261595000000000 -0.466218000000000
C 2.752310000000000 0.702474000000000 0.370763000000000
H 5.317123000000000 0.129220000000000 0.843933000000000
H 5.672268000000000 0.157014000000000 -0.908345000000000
C 0.394131000000000 0.163538000000000 1.015774000000000
C 1.369599000000000 0.374175000000000 -0.147386000000000
O -3.942155000000000 -0.400510000000000 -0.401351000000000
O 2.986837000000000 1.303035000000000 1.394141000000000
C 5.062705000000000 0.578510000000000 -0.114725000000000
H 5.194818000000000 1.658122000000000 -0.060638000000000
H 1.418436000000000 -0.520632000000000 -0.761969000000000
S 0.823078000000000 1.750883000000000 -1.238579000000000
C -0.988434000000000 -0.246035000000000 0.568084000000000
H -2.292041000000000 -1.742890000000000 -0.106542000000000
H 0.208615000000000 -5.183874000000000 0.802480000000000
C -2.035166000000000 0.780101000000000 0.552810000000000
H 1.468785000000000 0.357420000000000 -3.105916000000000
H 1.393800000000000 2.088961000000000 -3.503217000000000
C -1.284840000000000 -1.509662000000000 0.198044000000000
C -1.902571000000000 2.059528000000000 0.963099000000000
H -4.542922000000000 2.245384000000000 -0.747992000000000
C -3.132417000000000 2.889659000000000 0.813914000000000
H -0.996451000000000 2.490472000000000 1.363963000000000
C -4.164222000000000 1.927700000000000 0.224762000000000
H -3.434814000000000 3.304119000000000 1.779875000000000
H -2.932006000000000 3.747339000000000 0.164941000000000
C -3.435793000000000 0.603559000000000 0.061134000000000
H -5.028617000000000 1.776126000000000 0.873515000000000
C 1.723726000000000 1.359867000000000 -2.764079000000000
H 2.798682000000000 1.446176000000000 -2.623748000000000
H 0.548668000000000 -4.896462000000000 -0.919509000000000
H -0.934958000000000 -5.771104000000000 -0.439728000000000
C -0.354455000000000 -2.645268000000000 0.189838000000000
O 0.839934000000000 -2.636966000000000 0.420101000000000
C -0.238115000000000 -4.982489000000000 -0.170643000000000
O -1.019724000000000 -3.777337000000000 -0.124107000000000
H 0.815489000000000 -0.622312000000000 1.639436000000000
H 0.358357000000000 1.064423000000000 1.621646000000000
44
R5_SMe1_P1A_SMe6RR charge=0 multiplicity=1 B3LYP_D3BJ_TZVPD=-1757.203134987657, B3LYP_D3BJ_TZVPD_SMD_chcl3=-1757.228266315152, B3LYP_TZVP_CPCM_chcl3_freq=0.3014741, B3LYP_TZVPD_CPCM_chcl3=-1757.221474473459, M062X_TZVPD_SMD_chcl3=-1757.353568404651, M062X_TZVPD=-1757.327868190398, COSMORS=-0.004711976666812703, wB97MV_TZVPD_CPCM_chcl3=-1757.384201009606, wB97MV_TZVPD_SMD_chcl3=-1757.391156618574, wB97MV_TZVPD=-1757.365618425713, M062X_TZVPD_CPCM_chcl3=-1757.346858511237
C -3.602980000000000 -4.117374000000000 0.729961000000000
C -0.958546000000000 3.653881000000000 1.753152000000000
C 4.367181000000000 -0.700950000000000 -2.267829000000000
C -3.606262000000000 2.354948000000000 -1.491958000000000
C 2.739958000000000 1.060624000000000 1.846132000000000
C 3.605520000000000 0.328648000000000 0.814780000000000
C -0.398382000000000 -0.246568000000000 -1.058020000000000
C -0.606293000000000 -2.034771000000000 0.689595000000000
C 0.102182000000000 -1.048773000000000 0.117176000000000
C 1.416432000000000 0.324424000000000 1.850910000000000
C 2.582343000000000 -0.241472000000000 -0.180376000000000
C -1.364156000000000 0.871590000000000 -0.648615000000000
C -1.951356000000000 -2.478065000000000 0.297731000000000
C 1.440857000000000 -0.725709000000000 0.719269000000000
C -0.679204000000000 1.988391000000000 0.103078000000000
O 0.503074000000000 0.497137000000000 2.618648000000000
O -2.683470000000000 -1.972054000000000 -0.529575000000000
O 0.460134000000000 2.353792000000000 -0.089400000000000
O -2.301393000000000 -3.571788000000000 1.005180000000000
O -1.496773000000000 2.550277000000000 0.996209000000000
S 3.166028000000000 -1.594030000000000 -1.241887000000000
S -2.104611000000000 1.594466000000000 -2.168718000000000
H -3.671071000000000 -4.429703000000000 -0.311533000000000
H -4.375793000000000 -3.378161000000000 0.938126000000000
H -3.705866000000000 -4.972738000000000 1.391418000000000
H -0.113322000000000 3.314986000000000 2.349246000000000
H -1.769181000000000 3.987201000000000 2.393891000000000
H -0.644442000000000 4.452092000000000 1.082583000000000
H 5.198358000000000 -0.331729000000000 -1.669447000000000
H 3.883357000000000 0.126736000000000 -2.786082000000000
H 4.743390000000000 -1.412139000000000 -3.001650000000000
H -3.365387000000000 3.161400000000000 -0.802861000000000
H -4.219066000000000 1.604281000000000 -0.994524000000000
H -4.149804000000000 2.758338000000000 -2.345594000000000
H 2.521808000000000 2.084737000000000 1.532717000000000
H 3.161091000000000 1.102219000000000 2.850753000000000
H 4.151047000000000 -0.492497000000000 1.284229000000000
H 4.327535000000000 0.982471000000000 0.329898000000000
H -0.938169000000000 -0.905775000000000 -1.732354000000000
H 0.435705000000000 0.186423000000000 -1.605135000000000
H -0.171589000000000 -2.586630000000000 1.512603000000000
H 2.230511000000000 0.563143000000000 -0.826029000000000
H -2.180624000000000 0.467560000000000 -0.056339000000000
H 1.802251000000000 -1.621653000000000 1.243429000000000

38
R5_SMe6H_cyc4SZTS_0_1 charge=0 multiplicity=1 B3LYP_D3BJ_TZVPD=-1318.429674064149, B3LYP_D3BJ_TZVPD_SMD_chcl3=-1318.455365908885, B3LYP_TZVP_CPCM_chcl3_freq=0.25258096, B3LYP_TZVPD_CPCM_chcl3=-1318.450246172509, M062X_TZVPD_SMD_chcl3=-1318.539261091328, M062X_TZVPD=-1318.5126654529, COSMORS=-0.002544089079097565, wB97MV_TZVPD_CPCM_chcl3=-1318.577065164215, wB97MV_TZVPD_SMD_chcl3=-1318.582424846155, wB97MV_TZVPD=-1318.5560656122, M062X_TZVPD_CPCM_chcl3=-1318.534053420641
C 1.479958000000000 1.408194000000000 -0.198365000000000
C 0.096151000000000 1.229917000000000 -0.033646000000000
C -0.563031000000000 0.015397000000000 0.078477000000000
C -1.940939000000000 0.005915000000000 0.462106000000000
C -0.112351000000000 -1.318327000000000 -0.197950000000000
C 1.209154000000000 -1.938146000000000 -0.514155000000000
C -1.158715000000000 -2.095196000000000 -0.658016000000000
O -2.385869000000000 -1.737560000000000 -0.577164000000000
C 0.827962000000000 -3.367656000000000 -0.987920000000000
C -0.652324000000000 -3.300707000000000 -1.411139000000000
C 4.273046000000000 1.812340000000000 -0.506790000000000
O 3.345188000000000 2.909081000000000 -0.393116000000000
O 1.327345000000000 3.762896000000000 -0.137808000000000
O -2.748454000000000 1.853586000000000 -0.833862000000000
C -5.058769000000000 1.913226000000000 0.491185000000000
O -4.010849000000000 0.985333000000000 0.821991000000000
C -2.899192000000000 1.051457000000000 0.066494000000000
C 2.053721000000000 2.652245000000000 -0.242496000000000
H 2.131297000000000 0.554210000000000 -0.256839000000000
H -0.506699000000000 2.119415000000000 0.083161000000000
H -2.241553000000000 -0.592898000000000 1.307911000000000
H 1.748745000000000 -1.405251000000000 -1.303365000000000
H 1.484635000000000 -3.709535000000000 -1.786623000000000
H 0.936652000000000 -4.063340000000000 -0.155949000000000
H -0.766125000000000 -3.121287000000000 -2.485150000000000
H -1.213464000000000 -4.204919000000000 -1.169533000000000
H 5.250902000000000 2.274057000000000 -0.598166000000000
H 4.047393000000000 1.222295000000000 -1.394671000000000
H 4.228033000000000 1.187955000000000 0.384838000000000
H -4.713566000000000 2.937886000000000 0.625307000000000
H -5.871483000000000 1.691597000000000 1.176637000000000
H -5.377016000000000 1.769327000000000 -0.540852000000000
S 2.443770000000000 -1.936501000000000 0.859742000000000
C 1.421708000000000 -2.483928000000000 2.252395000000000
H 2.079455000000000 -2.533079000000000 3.119226000000000
H 0.619716000000000 -1.772882000000000 2.442287000000000
H 1.000778000000000 -3.471877000000000 2.068662000000000
H 1.907349000000000 4.538918000000000 -0.184826000000000
44
R5_SMe6_P2_SMe3RS charge=0 multiplicity=1 B3LYP_D3BJ_TZVPD=-1757.195369368523, B3LYP_D3BJ_TZVPD_SMD_chcl3=-1757.220889826164, B3LYP_TZVP_CPCM_chcl3_freq=0.30097611, B3LYP_TZVPD_CPCM_chcl3=-1757.215185639367, M062X_TZVPD_SMD_chcl3=-1757.345333468799, M062X_TZVPD=-1757.319478621554, COSMORS=-0.004728087977347189, wB97MV_TZVPD_CPCM_chcl3=-1757.377870116002, wB97MV_TZVPD_SMD_chcl3=-1757.383722369944, wB97MV_TZVPD=-1757.357999667009, M062X_TZVPD_CPCM_chcl3=-1757.339722631945
C -5.356368000000000 1.265497000000000 1.116575000000000
C -1.730629000000000 -4.345203000000000 0.053288000000000
C 1.589971000000000 -0.042289000000000 3.297176000000000
C -0.719167000000000 1.104119000000000 -3.281476000000000
C 3.993370000000000 0.557684000000000 -0.460774000000000
C 3.577273000000000 1.213470000000000 0.855046000000000
C -2.047556000000000 0.321264000000000 -0.127597000000000
C -0.769675000000000 0.703007000000000 -0.142891000000000
C -0.018069000000000 -1.392389000000000 -1.352871000000000
C 2.906860000000000 -0.457194000000000 -0.722391000000000
C 2.039846000000000 1.239753000000000 0.796248000000000
C -3.066194000000000 1.154604000000000 0.532933000000000
C -0.455969000000000 -2.384285000000000 -0.298278000000000
C 1.637522000000000 -0.046363000000000 0.046677000000000
C 0.354661000000000 -0.000044000000000 -0.830444000000000
O 3.030172000000000 -1.466663000000000 -1.377199000000000
O -2.879014000000000 2.233925000000000 1.054687000000000
O -0.109881000000000 -2.402402000000000 0.861212000000000
O -4.271549000000000 0.555434000000000 0.492754000000000
O -1.279527000000000 -3.297483000000000 -0.829351000000000
S 1.249937000000000 1.508376000000000 2.419486000000000
S 0.785899000000000 1.113072000000000 -2.274783000000000
H -6.231095000000000 0.635255000000000 0.984785000000000
H -5.504527000000000 2.230915000000000 0.634106000000000
H -5.150050000000000 1.416669000000000 2.175460000000000
H -2.376313000000000 -4.974644000000000 -0.551601000000000
H -2.283234000000000 -3.918444000000000 0.888872000000000
H -0.880866000000000 -4.914059000000000 0.427561000000000
H 2.656469000000000 -0.260340000000000 3.328629000000000
H 1.048015000000000 -0.877420000000000 2.856831000000000
H 1.233392000000000 0.112803000000000 4.314692000000000
H -1.588065000000000 1.378754000000000 -2.685550000000000
H -0.877001000000000 0.140773000000000 -3.761820000000000
H -0.557078000000000 1.861928000000000 -4.047086000000000
H 4.980909000000000 0.099212000000000 -0.468084000000000
H 3.947808000000000 1.281837000000000 -1.282940000000000
H 3.984237000000000 2.213299000000000 0.991899000000000
H 3.911579000000000 0.598159000000000 1.692015000000000
H -2.399088000000000 -0.597766000000000 -0.574613000000000
H -0.524136000000000 1.643073000000000 0.329150000000000
H -0.807761000000000 -1.327080000000000 -2.097017000000000
H 0.851388000000000 -1.827294000000000 -1.846968000000000
H 1.726938000000000 2.114574000000000 0.223219000000000
H 1.472508000000000 -0.849241000000000 0.766487000000000
38
R5_SMe6_P1RS_0_1 charge=0 multiplicity=1 B3LYP_D3BJ_TZVPD=-1318.511903589693, CCSDT_SMD_chcl3=-1316.769416354423, CCSDT=-1316.746649014737, B3LYP_D3BJ_TZVPD_SMD_chcl3=-1318.535546064436, B3LYP_TZVP_CPCM_chcl3_freq=0.25320825, B3LYP_TZVPD_CPCM_chcl3=-1318.530551353823, M062X_TZVPD_SMD_chcl3=-1318.62922982776, M062X_TZVPD=-1318.60516833692, COSMORS=-0.002544407799385092, wB97MV_TZVPD_CPCM_chcl3=-1318.670641167243, wB97MV_TZVPD_SMD_chcl3=-1318.675818053165, wB97MV_TZVPD=-1318.651873300216, M062X_TZVPD_CPCM_chcl3=-1318.624284652939
C 1.884734000000000 0.728048000000000 0.053519000000000
C 0.701888000000000 1.338569000000000 -0.074982000000000
C -0.619331000000000 0.793485000000000 0.203285000000000
C -1.664068000000000 1.598329000000000 -0.089793000000000
C -0.808889000000000 -0.586901000000000 0.769056000000000
C -0.911445000000000 -1.779212000000000 -0.208348000000000
C 0.125935000000000 -1.046511000000000 1.884236000000000
O 0.567492000000000 -0.342320000000000 2.761410000000000
C -0.713407000000000 -2.992061000000000 0.721556000000000
C 0.328896000000000 -2.541523000000000 1.747073000000000
C 5.478094000000000 1.195858000000000 -0.448122000000000
O 4.193424000000000 0.629954000000000 -0.130031000000000
O 3.211052000000000 2.569106000000000 -0.720476000000000
O -3.583367000000000 0.229389000000000 0.422287000000000
C -5.260277000000000 2.213637000000000 -0.120435000000000
O -3.833242000000000 2.366043000000000 -0.230655000000000
C -3.092871000000000 1.286058000000000 0.074179000000000
C 3.129243000000000 1.429756000000000 -0.311864000000000
H 2.002874000000000 -0.285279000000000 0.402862000000000
H 0.712120000000000 2.356735000000000 -0.448395000000000
H -1.464056000000000 2.582874000000000 -0.491151000000000
H -1.891329000000000 -1.784786000000000 -0.681599000000000
H -1.662794000000000 -3.202779000000000 1.220749000000000
H -0.418498000000000 -3.888274000000000 0.180476000000000
H 0.268210000000000 -3.037352000000000 2.714929000000000
H 1.344230000000000 -2.688465000000000 1.364531000000000
H 6.200399000000000 0.411405000000000 -0.242671000000000
H 5.670803000000000 2.067519000000000 0.176118000000000
H 5.513677000000000 1.484653000000000 -1.497824000000000
H -5.677706000000000 3.172269000000000 -0.413566000000000
H -5.535921000000000 1.973485000000000 0.905710000000000
H -5.607867000000000 1.424002000000000 -0.785352000000000
S 0.312752000000000 -1.700483000000000 -1.570740000000000
C -0.327307000000000 -3.049812000000000 -2.599380000000000
H -1.360935000000000 -2.853048000000000 -2.884070000000000
H 0.292627000000000 -3.078852000000000 -3.494342000000000
H -0.258570000000000 -4.009494000000000 -2.089133000000000
H -1.783346000000000 -0.589045000000000 1.270029000000000
44
R5_SMe4_P1S_SMe6SR charge=0 multiplicity=1 B3LYP_D3BJ_TZVPD=-1757.195330556972, B3LYP_D3BJ_TZVPD_SMD_chcl3=-1757.221195658727, B3LYP_TZVP_CPCM_chcl3_freq=0.30022989, B3LYP_TZVPD_CPCM_chcl3=-1757.215080475377, M062X_TZVPD_SMD_chcl3=-1757.3451592208, M062X_TZVPD=-1757.319195994364, COSMORS=-0.0032165410777362856, wB97MV_TZVPD_CPCM_chcl3=-1757.37763382237, wB97MV_TZVPD_SMD_chcl3=-1757.383917545119, wB97MV_TZVPD=-1757.357969809678, M062X_TZVPD_CPCM_chcl3=-1757.339142428802
C -5.052965000000000 -2.513361000000000 -0.133180000000000
C 2.019233000000000 -4.238370000000000 -0.242939000000000
C 0.373486000000000 3.101123000000000 -2.629811000000000
C 4.402166000000000 -0.290680000000000 0.576608000000000
C -0.789775000000000 -0.382023000000000 0.381802000000000
C -0.761136000000000 2.453799000000000 2.377197000000000
C -1.529639000000000 2.735240000000000 1.082581000000000
C -1.819741000000000 -0.807652000000000 -0.342403000000000
C 0.418206000000000 0.309941000000000 -0.176344000000000
C 0.488407000000000 1.705960000000000 1.952680000000000
C -0.425095000000000 2.792215000000000 0.012347000000000
C -2.969632000000000 -1.478101000000000 0.293179000000000
C 0.611597000000000 1.724234000000000 0.428929000000000
C 1.658597000000000 -0.572921000000000 0.072683000000000
C 1.495942000000000 -1.952025000000000 -0.528048000000000
O 1.277127000000000 1.175601000000000 2.702789000000000
O -3.108158000000000 -1.679590000000000 1.481960000000000
O 0.981851000000000 -2.186125000000000 -1.596641000000000
O -3.876335000000000 -1.848066000000000 -0.627603000000000
O 2.036209000000000 -2.887867000000000 0.262952000000000
S -1.109137000000000 2.696252000000000 -1.665819000000000
S 3.177443000000000 0.131808000000000 -0.693793000000000
H -5.653266000000000 -2.733770000000000 -1.010949000000000
H -5.596895000000000 -1.860718000000000 0.548623000000000
H -4.778577000000000 -3.432107000000000 0.383683000000000
H 2.574886000000000 -4.294971000000000 -1.177851000000000
H 0.993278000000000 -4.564942000000000 -0.405034000000000
H 2.496241000000000 -4.840926000000000 0.524075000000000
H 0.071676000000000 3.134608000000000 -3.675593000000000
H 1.144493000000000 2.340884000000000 -2.506019000000000
H 0.765105000000000 4.075077000000000 -2.336725000000000
H 5.355525000000000 0.099348000000000 0.222083000000000
H 4.476903000000000 -1.369683000000000 0.697726000000000
H 4.144020000000000 0.181185000000000 1.523275000000000
H -0.809031000000000 -0.556118000000000 1.454201000000000
H -1.313466000000000 1.881132000000000 3.122704000000000
H -0.432775000000000 3.381719000000000 2.857008000000000
H -2.101428000000000 3.660758000000000 1.117535000000000
H -2.221563000000000 1.920398000000000 0.867686000000000
H -1.854568000000000 -0.672614000000000 -1.414841000000000
H 0.285459000000000 0.425223000000000 -1.251597000000000
H 0.070167000000000 3.763315000000000 0.085583000000000
H 1.621877000000000 2.040850000000000 0.170696000000000
H 1.854028000000000 -0.653974000000000 1.139149000000000
55
Sulf_SMe4_P3_0_1 charge=0 multiplicity=1 B3LYP_D3BJ_TZVPD=-2138.40616541553, B3LYP_D3BJ_TZVPD_SMD_chcl3=-2138.441138973026, B3LYP_TZVP_CPCM_chcl3_freq=0.3709146, B3LYP_TZVPD_CPCM_chcl3=-2138.431074275304, M062X_TZVPD_SMD_chcl3=-2138.593529092151, M062X_TZVPD=-2138.557055350084, COSMORS=-0.008169374665833099, wB97MV_TZVPD_CPCM_chcl3=-2138.660445510674, wB97MV_TZVPD_SMD_chcl3=-2138.670877471183, wB97MV_TZVPD=-2138.634983007076, M062X_TZVPD_CPCM_chcl3=-2138.583257135647
C 0.277646000000000 3.000880000000000 0.885456000000000
C -0.444776000000000 3.600776000000000 1.905789000000000
C -1.801774000000000 3.866067000000000 1.736787000000000
C -2.438843000000000 3.537339000000000 0.546071000000000
C -1.726033000000000 2.930983000000000 -0.480506000000000
C -0.376914000000000 2.666355000000000 -0.296209000000000
O 1.811989000000000 2.570996000000000 -1.730557000000000
O -0.299736000000000 1.728717000000000 -2.746520000000000
S 0.549914000000000 1.868354000000000 -1.581137000000000
C 2.140015000000000 -3.150211000000000 -2.102604000000000
S 0.370022000000000 -3.335655000000000 -1.756849000000000
C -2.098659000000000 -0.323182000000000 -1.059075000000000
C -3.459261000000000 -0.167399000000000 -0.834932000000000
C -3.942929000000000 -0.035288000000000 0.461427000000000
C -3.063472000000000 -0.083111000000000 1.538352000000000
C -1.706367000000000 -0.265874000000000 1.318732000000000
C -1.212862000000000 -0.364734000000000 0.016940000000000
C 4.619263000000000 2.380716000000000 0.609097000000000
O 3.594343000000000 1.669176000000000 1.335065000000000
O 4.246162000000000 -0.253221000000000 0.370882000000000
O -1.011308000000000 -3.570050000000000 1.916349000000000
C -3.218328000000000 -3.809528000000000 0.424570000000000
O -1.917278000000000 -3.539046000000000 -0.137044000000000
C -0.910492000000000 -3.423021000000000 0.721623000000000
C 3.505658000000000 0.353670000000000 1.108792000000000
C 0.422561000000000 -3.086246000000000 0.057538000000000
C 1.000404000000000 0.293913000000000 -0.920090000000000
C 0.239420000000000 -0.537316000000000 -0.200518000000000
C 0.897948000000000 -1.701345000000000 0.446240000000000
C 1.874995000000000 -1.558550000000000 1.344269000000000
C 2.363225000000000 -0.251673000000000 1.896643000000000
H 1.331759000000000 2.791055000000000 1.004819000000000
H 0.050089000000000 3.862242000000000 2.831915000000000
H -2.362715000000000 4.330564000000000 2.537632000000000
H -3.493281000000000 3.742378000000000 0.417084000000000
H -2.207126000000000 2.653796000000000 -1.405964000000000
H 2.260440000000000 -3.325636000000000 -3.170710000000000
H 2.720860000000000 -3.887173000000000 -1.548764000000000
H 2.490025000000000 -2.147789000000000 -1.863596000000000
H -1.716878000000000 -0.420643000000000 -2.064453000000000
H -4.141212000000000 -0.142761000000000 -1.675108000000000
H -5.003463000000000 0.096401000000000 0.634217000000000
H -3.435845000000000 0.014962000000000 2.549808000000000
H -1.021667000000000 -0.316913000000000 2.154901000000000
H 4.429478000000000 2.307260000000000 -0.460133000000000
H 5.600662000000000 1.972781000000000 0.844435000000000
H 4.542615000000000 3.412259000000000 0.939050000000000
H -3.485162000000000 -3.023267000000000 1.127233000000000
H -3.214102000000000 -4.775435000000000 0.926835000000000
H -3.899873000000000 -3.816685000000000 -0.420047000000000
H 1.130805000000000 -3.804410000000000 0.471691000000000
H 2.049304000000000 0.099968000000000 -1.097190000000000
H 2.332116000000000 -2.455626000000000 1.745357000000000
H 2.729526000000000 -0.396794000000000 2.917137000000000
H 1.562532000000000 0.483836000000000 1.952849000000000
44
R5_SMe1_P2A_SMe6R_P2 charge=0 multiplicity=1 B3LYP_D3BJ_TZVPD=-1757.205170250144, B3LYP_D3BJ_TZVPD_SMD_chcl3=-1757.231223570096, B3LYP_TZVP_CPCM_chcl3_freq=0.30113998, B3LYP_TZVPD_CPCM_chcl3=-1757.22404770999, M062X_TZVPD_SMD_chcl3=-1757.354621185861, M062X_TZVPD=-1757.328474754964, COSMORS=-0.004101850420399875, wB97MV_TZVPD_CPCM_chcl3=-1757.385077080232, wB97MV_TZVPD_SMD_chcl3=-1757.392403106268, wB97MV_TZVPD=-1757.366189392506, M062X_TZVPD_CPCM_chcl3=-1757.347564365927
C -1.870482000000000 -4.696830000000000 -0.162897000000000
C 3.166611000000000 1.543553000000000 -2.465009000000000
C -0.908751000000000 2.134767000000000 -2.137159000000000
C 4.339800000000000 0.553680000000000 1.670867000000000
C -2.236677000000000 1.972450000000000 2.332211000000000
C -3.299417000000000 1.621946000000000 1.286452000000000
C 0.647623000000000 -1.078547000000000 0.740880000000000
C -1.067894000000000 -1.212421000000000 -1.154625000000000
C -0.591244000000000 -0.504918000000000 0.095334000000000
C -1.017134000000000 1.152930000000000 1.946157000000000
C -2.497925000000000 1.190627000000000 0.044754000000000
C 1.865939000000000 -0.170359000000000 0.541104000000000
C -1.913098000000000 -2.408830000000000 -0.757955000000000
C -1.271866000000000 0.535080000000000 0.613391000000000
C 2.219443000000000 0.031068000000000 -0.914027000000000
O -0.029945000000000 1.017772000000000 2.644770000000000
O -3.107302000000000 -2.366881000000000 -0.574377000000000
O 2.082005000000000 -0.793547000000000 -1.789325000000000
O -1.173924000000000 -3.512129000000000 -0.603600000000000
O 2.734366000000000 1.250504000000000 -1.121216000000000
S -2.030840000000000 2.739402000000000 -0.853341000000000
S 3.318563000000000 -0.917987000000000 1.381985000000000
H -2.621988000000000 -4.983673000000000 -0.896680000000000
H -2.346424000000000 -4.514980000000000 0.799384000000000
H -1.107670000000000 -5.464418000000000 -0.075537000000000
H 3.566721000000000 2.552206000000000 -2.427078000000000
H 2.320832000000000 1.492730000000000 -3.149118000000000
H 3.933772000000000 0.836239000000000 -2.775953000000000
H -1.427808000000000 1.504990000000000 -2.857969000000000
H -0.074854000000000 1.592218000000000 -1.695667000000000
H -0.523341000000000 3.017981000000000 -2.644845000000000
H 4.660466000000000 1.000831000000000 0.732452000000000
H 5.212850000000000 0.211274000000000 2.225257000000000
H 3.793598000000000 1.282010000000000 2.268838000000000
H -2.527030000000000 1.749009000000000 3.358715000000000
H -1.956417000000000 3.028086000000000 2.294405000000000
H -3.984486000000000 2.441919000000000 1.073426000000000
H -3.892333000000000 0.768226000000000 1.619300000000000
H 0.852530000000000 -2.063421000000000 0.323944000000000
H 0.501147000000000 -1.181729000000000 1.814582000000000
H -0.208943000000000 -1.546432000000000 -1.732416000000000
H -1.690879000000000 -0.573091000000000 -1.770627000000000
H -3.078151000000000 0.561643000000000 -0.622138000000000
H 1.684731000000000 0.790425000000000 1.013660000000000
40
R6_SMe6_cyc4RTS_-1_1 charge=-1 multiplicity=1 wB97MV_TZVPD_SMD_chcl3=-1357.442438465563, B3LYP_D3BJ_TZVPD=-1357.233189864106, B3LYP_TZVP_CPCM_chcl3_freq=0.26744444, B3LYP_TZVPD_CPCM_chcl3=-1357.299581478333, M062X_TZVPD=-1357.328501929785, COSMORS=-0.01124314499077169, M062X_TZVPD_CPCM_chcl3=-1357.397358397233, M062X_TZVPD_SMD_chcl3=-1357.399995265478, wB97MV_TZVPD_CPCM_chcl3=-1357.439575332696, B3LYP_D3BJ_TZVPD_SMD_chcl3=-1357.302071261067, wB97MV_TZVPD=-1357.372113643975
O 4.986467000000000 -0.106265000000000 0.222200000000000
C 3.855924000000000 0.688137000000000 0.244855000000000
C -0.704751000000000 -2.457830000000000 -0.434759000000000
H 6.325486000000000 1.173748000000000 1.181866000000000
C 1.411366000000000 0.494815000000000 0.220770000000000
C 2.665252000000000 -0.092197000000000 0.189423000000000
H 7.001162000000000 -0.191518000000000 0.252241000000000
O 3.964162000000000 1.914326000000000 0.300084000000000
C 6.235102000000000 0.581388000000000 0.269719000000000
H 6.354870000000000 1.242460000000000 -0.590319000000000
H -0.846053000000000 -4.744922000000000 -1.795520000000000
H 0.777694000000000 -2.023880000000000 1.054596000000000
C 0.168520000000000 -0.136368000000000 0.212953000000000
H -2.310418000000000 -4.555636000000000 -0.804705000000000
C 0.003322000000000 -1.522709000000000 0.479467000000000
C -1.107565000000000 0.564104000000000 0.362637000000000
C -1.373445000000000 1.980119000000000 -0.026579000000000
H -1.021879000000000 -5.678220000000000 -0.280833000000000
S -1.110349000000000 2.292132000000000 -1.834760000000000
C -1.926424000000000 -0.144629000000000 1.188644000000000
H -2.346517000000000 2.347602000000000 2.524689000000000
H -0.620179000000000 2.646969000000000 0.408955000000000
C -3.121778000000000 0.443555000000000 1.880317000000000
O -1.595442000000000 -1.388708000000000 1.514439000000000
H -3.515994000000000 2.057375000000000 -0.277486000000000
C -3.099122000000000 1.970977000000000 1.824661000000000
H -4.039635000000000 0.060385000000000 1.417225000000000
H -3.128838000000000 0.082873000000000 2.912938000000000
C -2.764446000000000 2.450063000000000 0.413128000000000
H -4.064114000000000 2.376885000000000 2.140247000000000

H -2.809788000000000 3.538826000000000 0.349575000000000
C -2.150061000000000 1.003141000000000 -2.573317000000000
H -1.997928000000000 1.063128000000000 -3.650772000000000
H -1.843882000000000 0.022674000000000 -2.213500000000000
H -3.205243000000000 1.167654000000000 -2.355630000000000
O -0.578544000000000 -3.727221000000000 0.003839000000000
O -1.294475000000000 -2.176763000000000 -1.455446000000000
C -1.235897000000000 -4.735900000000000 -0.777913000000000
H 1.403300000000000 1.580993000000000 0.211424000000000
H 2.781063000000000 -1.163941000000000 0.096391000000000
44
R5_SMe4_P3_SMe6SRR_P2 charge=0 multiplicity=1 B3LYP_D3BJ_TZVPD=-1757.201439833779, B3LYP_D3BJ_TZVPD_SMD_chcl3=-1757.228112520762, B3LYP_TZVP_CPCM_chcl3_freq=0.30089007, B3LYP_TZVPD_CPCM_chcl3=-1757.221595236019, M062X_TZVPD_SMD_chcl3=-1757.351857760031, M062X_TZVPD=-1757.32523123566, COSMORS=-0.0029096453128765925, wB97MV_TZVPD_CPCM_chcl3=-1757.38373503908, wB97MV_TZVPD_SMD_chcl3=-1757.390422853372, wB97MV_TZVPD=-1757.363788594394, M062X_TZVPD_CPCM_chcl3=-1757.345425244993
C -5.124856000000000 -2.655318000000000 0.981190000000000
C -2.469861000000000 4.179242000000000 -0.303572000000000
C 4.049711000000000 -0.910204000000000 1.382388000000000
C 1.994729000000000 2.438895000000000 1.591510000000000
C -1.090722000000000 -0.424377000000000 -0.353711000000000
C 1.761695000000000 -2.459336000000000 -2.197191000000000
C 2.459778000000000 -2.771729000000000 -0.868964000000000
C -1.914951000000000 -0.945552000000000 0.551019000000000
C 0.225915000000000 0.211055000000000 -0.023945000000000
C 1.270347000000000 -1.033001000000000 -2.056147000000000
C 1.695513000000000 -1.913576000000000 0.150353000000000
C -3.192343000000000 -1.570269000000000 0.154661000000000
C 1.392891000000000 -0.608730000000000 -0.596675000000000
C 0.239971000000000 1.669304000000000 -0.522203000000000
C -0.875621000000000 2.483388000000000 0.094039000000000
O 0.856374000000000 -0.335032000000000 -2.954622000000000
O -3.612111000000000 -1.675645000000000 -0.978537000000000
O -1.202070000000000 2.426140000000000 1.258241000000000
O -3.856569000000000 -2.022631000000000 1.232503000000000
O -1.441583000000000 3.299198000000000 -0.800722000000000
S 2.473034000000000 -1.713468000000000 1.782681000000000
S 1.807772000000000 2.569636000000000 -0.208636000000000
H -5.500575000000000 -2.951693000000000 1.956121000000000
H -4.991987000000000 -3.526281000000000 0.340645000000000
H -5.808528000000000 -1.953990000000000 0.504445000000000
H -2.058825000000000 4.844401000000000 0.454573000000000
H -3.287656000000000 3.599101000000000 0.120900000000000
H -2.808422000000000 4.744902000000000 -1.166281000000000
H 4.533652000000000 -0.716368000000000 2.338722000000000
H 3.897262000000000 0.038328000000000 0.869732000000000
H 4.690490000000000 -1.557430000000000 0.786658000000000
H 2.105403000000000 1.402518000000000 1.904974000000000
H 1.154023000000000 2.898819000000000 2.105681000000000
H 2.908626000000000 2.983435000000000 1.825923000000000
H -1.374357000000000 -0.460425000000000 -1.400651000000000
H 0.868301000000000 -3.081601000000000 -2.324058000000000
H 2.376591000000000 -2.582676000000000 -3.088258000000000
H 3.503148000000000 -2.456383000000000 -0.918053000000000
H 2.438908000000000 -3.827731000000000 -0.606492000000000
H -1.683231000000000 -0.926050000000000 1.608313000000000
H 0.342045000000000 0.219065000000000 1.060257000000000
H 0.752098000000000 -2.409224000000000 0.395153000000000
H 2.286027000000000 0.027856000000000 -0.578261000000000
H 0.148273000000000 1.695309000000000 -1.606384000000000
40
R6_SMe6_cyc4R_-1_1 charge=-1 multiplicity=1 wB97MV_TZVPD_SMD_chcl3=-1357.479635969456, B3LYP_D3BJ_TZVPD=-1357.258425231976, B3LYP_TZVP_CPCM_chcl3_freq=0.26978787, B3LYP_TZVPD_CPCM_chcl3=-1357.328512421715, B3LYP_D3BJ_TZVPD_SMD_chcl3=-1357.331267149183, M062X_TZVPD=-1357.364806835318, COSMORS=-0.011146174343291617, M062X_TZVPD_CPCM_chcl3=-1357.437427938395, M062X_TZVPD_SMD_chcl3=-1357.44032301396, wB97MV_TZVPD_CPCM_chcl3=-1357.476544599204, wB97MV_TZVPD=-1357.405143330825
O -4.842447000000000 0.005650000000000 -0.512804000000000
C -3.673196000000000 0.762469000000000 -0.381666000000000
C -0.131056000000000 -2.443065000000000 0.155507000000000
H -6.856960000000000 -0.017717000000000 -0.494029000000000
C -1.226845000000000 0.539928000000000 -0.476749000000000
C -2.518708000000000 -0.020455000000000 -0.555383000000000
H -6.144693000000000 1.154327000000000 0.645220000000000
O -3.768539000000000 1.976978000000000 -0.139086000000000
C -6.063245000000000 0.714857000000000 -0.351686000000000
H -6.164769000000000 1.514102000000000 -1.089379000000000
H 0.340509000000000 -4.248761000000000 2.065382000000000
H -0.359290000000000 -1.877739000000000 -1.871884000000000
C -0.021034000000000 -0.069472000000000 -0.658328000000000
H -0.378368000000000 -2.783258000000000 2.774291000000000
C 0.220689000000000 -1.529503000000000 -1.012095000000000
C 1.302006000000000 0.518632000000000 -0.644763000000000
C 1.695136000000000 1.914398000000000 -0.331534000000000
H 1.376610000000000 -3.082991000000000 2.939973000000000
S 1.526170000000000 2.318283000000000 1.484675000000000
C 2.202459000000000 -0.401262000000000 -1.020129000000000
H 5.086120000000000 1.376376000000000 -0.969634000000000
H 0.982035000000000 2.613547000000000 -0.774047000000000
C 3.662704000000000 -0.238320000000000 -1.194373000000000
O 1.630416000000000 -1.626523000000000 -1.319941000000000
H 3.452415000000000 3.164540000000000 -0.471715000000000
C 4.101802000000000 1.102307000000000 -0.584546000000000
H 4.199890000000000 -1.069035000000000 -0.724559000000000
H 3.918594000000000 -0.275221000000000 -2.262249000000000
C 3.097188000000000 2.217134000000000 -0.880286000000000
H 4.205566000000000 0.992543000000000 0.497360000000000
H 3.010407000000000 2.344369000000000 -1.965644000000000
C 1.823256000000000 0.733277000000000 2.314819000000000
H 1.130725000000000 -0.022338000000000 1.951973000000000
H 2.846384000000000 0.386869000000000 2.183690000000000
H 1.639674000000000 0.917045000000000 3.373605000000000
O 0.817607000000000 -2.463552000000000 1.099349000000000
O -1.182248000000000 -3.035866000000000 0.247700000000000
C 0.508777000000000 -3.197561000000000 2.296798000000000
H -1.188829000000000 1.601703000000000 -0.246381000000000
H -2.651886000000000 -1.076796000000000 -0.741077000000000
55
Sulf_SMe6_P1S_0_1 charge=0 multiplicity=1 B3LYP_D3BJ_TZVPD=-2138.407942299562, B3LYP_D3BJ_TZVPD_SMD_chcl3=-2138.444733520348, B3LYP_TZVP_CPCM_chcl3_freq=0.37165842, B3LYP_TZVPD_CPCM_chcl3=-2138.433894122209, M062X_TZVPD_SMD_chcl3=-2138.595981130546, M062X_TZVPD=-2138.557797101028, COSMORS=-0.008386056653308292, wB97MV_TZVPD_CPCM_chcl3=-2138.6601401406, wB97MV_TZVPD_SMD_chcl3=-2138.671282287823, wB97MV_TZVPD=-2138.633687276202, M062X_TZVPD_CPCM_chcl3=-2138.585013510607
C 2.128867000000000 0.596694000000000 -2.054500000000000
C 3.451323000000000 0.289090000000000 -1.772137000000000
C 4.077570000000000 0.864786000000000 -0.671683000000000
C 3.389121000000000 1.760048000000000 0.142211000000000
C 2.065595000000000 2.074349000000000 -0.131222000000000
C 1.447051000000000 1.480287000000000 -1.222627000000000
O -0.732438000000000 2.819213000000000 -0.610709000000000
O -0.307462000000000 2.209430000000000 -2.993405000000000
S -0.246772000000000 1.865758000000000 -1.586822000000000
C -2.343681000000000 -0.115131000000000 -3.889635000000000
S -2.767158000000000 0.469310000000000 -2.226557000000000
C -1.941694000000000 -2.489336000000000 -0.580324000000000
C -2.587923000000000 -3.698098000000000 -0.349652000000000
C -3.450760000000000 -3.836034000000000 0.731532000000000
C -3.663392000000000 -2.754984000000000 1.580781000000000
C -3.015106000000000 -1.548618000000000 1.349098000000000
C -2.149857000000000 -1.401245000000000 0.265195000000000
C 5.177600000000000 -3.068986000000000 0.758471000000000
O 4.149562000000000 -2.188054000000000 1.246437000000000
O 2.781236000000000 -2.912749000000000 -0.388651000000000
O -2.164421000000000 1.795041000000000 2.347072000000000
C -1.062655000000000 3.753580000000000 3.760063000000000
O -0.287907000000000 2.660396000000000 3.232995000000000
C -0.964606000000000 1.742306000000000 2.519008000000000
C 2.987577000000000 -2.199451000000000 0.571167000000000
C -0.031521000000000 0.708593000000000 2.039439000000000
C -1.175636000000000 0.290348000000000 -1.374528000000000
C -1.437365000000000 -0.064335000000000 0.100736000000000
C -0.190522000000000 -0.089884000000000 0.971007000000000
C 0.843210000000000 -1.048537000000000 0.578815000000000
C 2.039221000000000 -1.230778000000000 1.145867000000000
H 1.634581000000000 0.157109000000000 -2.910265000000000
H 3.987870000000000 -0.405973000000000 -2.403588000000000
H 5.105657000000000 0.613736000000000 -0.445547000000000
H 3.881871000000000 2.208782000000000 0.994543000000000
H 1.511518000000000 2.755569000000000 0.497525000000000
H -3.271602000000000 -0.088296000000000 -4.459612000000000
H -1.610137000000000 0.539732000000000 -4.351370000000000
H -1.977263000000000 -1.140109000000000 -3.845952000000000
H -1.286098000000000 -2.405388000000000 -1.436448000000000
H -2.418209000000000 -4.531330000000000 -1.019761000000000
H -3.955895000000000 -4.776943000000000 0.908863000000000
H -4.337163000000000 -2.849173000000000 2.423095000000000
H -3.180883000000000 -0.706968000000000 2.009644000000000
H 5.437333000000000 -2.811551000000000 -0.267719000000000
H 4.840197000000000 -4.103758000000000 0.799836000000000
H 6.027644000000000 -2.917608000000000 1.417120000000000
H -1.824099000000000 3.380780000000000 4.443956000000000
H -0.352650000000000 4.385214000000000 4.285623000000000
H -1.537797000000000 4.302825000000000 2.948495000000000
H 0.887790000000000 0.657128000000000 2.605892000000000
H -0.579114000000000 -0.455011000000000 -1.896834000000000
H -2.094405000000000 0.704647000000000 0.493926000000000
H 0.624964000000000 -1.665406000000000 -0.283192000000000
H 2.382203000000000 -0.672903000000000 2.005222000000000
41
R6_SMe7_P1_0_1 charge=0 multiplicity=1 B3LYP_D3BJ_TZVPD=-1357.809007915506, wB97MV_TZVPD=-1357.969847984788, B3LYP_D3BJ_TZVPD_SMD_chcl3=-1357.830411829926, B3LYP_TZVP_CPCM_chcl3_freq=0.28113468, B3LYP_TZVPD_CPCM_chcl3=-1357.824607260141, M062X_TZVPD_SMD_chcl3=-1357.929608494114, M062X_TZVPD=-1357.907419285986, COSMORS=-0.006553972760531541, wB97MV_TZVPD_CPCM_chcl3=-1357.96026518234, wB97MV_TZVPD_SMD_chcl3=-1357.971139795224, M062X_TZVPD_CPCM_chcl3=-1357.923821046642
O -4.450004000000000 0.516533000000000 0.540713000000000
C -3.497848000000000 1.351951000000000 0.091538000000000
O -3.737617000000000 2.429004000000000 -0.412983000000000
H -5.968588000000000 1.885773000000000 0.944847000000000
H -6.424158000000000 0.162569000000000 0.798056000000000
C -2.158360000000000 0.773279000000000 0.300306000000000
C -1.059121000000000 1.432024000000000 -0.083698000000000
C -5.810993000000000 0.961083000000000 0.390996000000000
H -6.041227000000000 1.122794000000000 -0.661282000000000
H 0.312949000000000 0.276373000000000 2.584176000000000
H 1.026017000000000 2.818635000000000 -0.537695000000000
O 3.395969000000000 2.758158000000000 -0.043580000000000
C 0.307568000000000 0.949523000000000 0.081953000000000
O 3.303178000000000 0.521302000000000 0.047950000000000
C 4.829668000000000 2.683418000000000 0.091500000000000
H 5.175196000000000 3.712197000000000 0.064498000000000
H 5.093139000000000 2.213536000000000 1.037803000000000
H 5.253668000000000 2.111963000000000 -0.732727000000000
C 1.294135000000000 1.824290000000000 -0.204742000000000
C 0.498287000000000 -0.425267000000000 0.634504000000000
C 2.734254000000000 1.596529000000000 -0.053163000000000
H -2.109390000000000 -0.199020000000000 0.767966000000000
C 0.460253000000000 -0.597109000000000 1.956097000000000
C 0.683033000000000 -1.610857000000000 -0.307554000000000
O 2.021387000000000 -1.721523000000000 -0.743120000000000
C 0.580744000000000 -1.912056000000000 2.660180000000000
H 0.581076000000000 -3.748052000000000 -0.268976000000000
H -0.776518000000000 -2.951884000000000 0.527716000000000
C 1.018746000000000 -3.039051000000000 1.729213000000000
H 1.272777000000000 -1.806610000000000 3.500742000000000
H -0.393562000000000 -2.145397000000000 3.108810000000000
C 0.305606000000000 -2.920991000000000 0.385589000000000
H 2.096997000000000 -2.984170000000000 1.564250000000000
H 0.809846000000000 -4.009081000000000 2.184000000000000
H -1.175361000000000 2.406634000000000 -0.544841000000000
S -0.432156000000000 -1.326513000000000 -1.787929000000000
C 0.106183000000000 -2.692594000000000 -2.852239000000000
H 1.190552000000000 -2.688961000000000 -2.940298000000000
H -0.238980000000000 -3.655190000000000 -2.478868000000000
H -0.337579000000000 -2.509401000000000 -3.829959000000000
H 2.501159000000000 -0.899218000000000 -0.514955000000000
41
R6_SMe6H_cyc4SETS_0_1 charge=0 multiplicity=1 B3LYP_D3BJ_TZVPD=-1357.732646482917, B3LYP_D3BJ_TZVPD_SMD_chcl3=-1357.763438079151, B3LYP_TZVP_CPCM_chcl3_freq=0.28076653, B3LYP_TZVPD_CPCM_chcl3=-1357.757792296867, M062X_TZVPD_SMD_chcl3=-1357.850184723002, M062X_TZVPD=-1357.817670216305, COSMORS=-0.0009611328990662495, wB97MV_TZVPD_CPCM_chcl3=-1357.884439668094, wB97MV_TZVPD_SMD_chcl3=-1357.890271085308, wB97MV_TZVPD=-1357.85804950776, M062X_TZVPD_CPCM_chcl3=-1357.844508732788
O -2.424469000000000 3.103515000000000 -0.428188000000000
C -1.146228000000000 3.130351000000000 -0.724932000000000
C -2.319642000000000 -1.748833000000000 0.363869000000000
H -2.849555000000000 5.093488000000000 0.041492000000000
C -0.856222000000000 0.790437000000000 -0.150565000000000
C -0.346975000000000 2.011523000000000 -0.592178000000000
H -3.228115000000000 4.605578000000000 -1.635849000000000
O -0.699714000000000 4.303539000000000 -1.158514000000000
C -3.227948000000000 4.294406000000000 -0.593012000000000
H -4.224949000000000 3.999630000000000 -0.282635000000000
H -4.343455000000000 -3.444531000000000 0.091580000000000
H -0.466544000000000 -2.076379000000000 1.416600000000000
C -0.172027000000000 -0.400910000000000 0.092061000000000
H -4.827339000000000 -2.148592000000000 1.209810000000000
C -0.895279000000000 -1.496782000000000 0.615600000000000
C 1.202147000000000 -0.753542000000000 -0.200447000000000
C 2.373746000000000 0.179678000000000 -0.240524000000000
H -4.380349000000000 -3.756017000000000 1.852459000000000
S 2.556291000000000 1.135643000000000 1.338585000000000
C 1.284402000000000 -2.077827000000000 -0.624277000000000
H 3.056018000000000 -0.931117000000000 -2.625509000000000
H 2.232221000000000 0.988039000000000 -0.963942000000000
C 2.499004000000000 -2.592274000000000 -1.365942000000000
O 0.307863000000000 -2.900423000000000 -0.488013000000000
H 4.037654000000000 -1.106879000000000 0.257437000000000
C 3.463947000000000 -1.484590000000000 -1.773758000000000
H 3.005329000000000 -3.312003000000000 -0.713034000000000
H 2.141169000000000 -3.159894000000000 -2.228140000000000
C 3.678769000000000 -0.533191000000000 -0.601344000000000
H 4.415486000000000 -1.913897000000000 -2.096218000000000
H 4.439125000000000 0.214887000000000 -0.830419000000000
C 2.566450000000000 -0.195148000000000 2.569339000000000
H 2.608534000000000 0.288364000000000 3.544566000000000
H 1.655382000000000 -0.786728000000000 2.497636000000000
H 3.435606000000000 -0.841564000000000 2.455751000000000
O -2.803604000000000 -2.647138000000000 1.245379000000000
O -2.996750000000000 -1.254533000000000 -0.517947000000000
C -4.182404000000000 -3.017438000000000 1.081010000000000
H -1.911750000000000 0.777255000000000 0.081809000000000
H 0.691943000000000 2.139256000000000 -0.847993000000000
H 0.250129000000000 4.262954000000000 -1.347741000000000
55
Sulf_SMe4_P4_0_1 charge=0 multiplicity=1 B3LYP_D3BJ_TZVPD=-2138.416345365316, B3LYP_D3BJ_TZVPD_SMD_chcl3=-2138.450649903385, B3LYP_TZVP_CPCM_chcl3_freq=0.37122861, B3LYP_TZVPD_CPCM_chcl3=-2138.440653039046, M062X_TZVPD_SMD_chcl3=-2138.602111042643, M062X_TZVPD=-2138.56669048173, COSMORS=-0.0085680300014718, wB97MV_TZVPD_CPCM_chcl3=-2138.666147345398, wB97MV_TZVPD_SMD_chcl3=-2138.676502014226, wB97MV_TZVPD=-2138.641475537304, M062X_TZVPD_CPCM_chcl3=-2138.591998077329
C -2.407251000000000 -0.402564000000000 1.894458000000000
C -2.801491000000000 0.607813000000000 2.762430000000000
C -3.811437000000000 1.490258000000000 2.392963000000000
C -4.437779000000000 1.368436000000000 1.155537000000000
C -4.058532000000000 0.359705000000000 0.281291000000000
C -3.046867000000000 -0.515327000000000 0.664774000000000
O -3.658361000000000 -2.195411000000000 -1.263928000000000
O -1.806174000000000 -2.787658000000000 0.296967000000000
S -2.527805000000000 -1.780918000000000 -0.456962000000000
C 1.087126000000000 -3.688487000000000 1.718726000000000
S 1.290879000000000 -1.888501000000000 1.660263000000000
C -1.583001000000000 1.970369000000000 -2.194412000000000
C -2.125482000000000 3.247506000000000 -2.218722000000000
C -2.125980000000000 4.030582000000000 -1.067995000000000
C -1.570099000000000 3.529311000000000 0.103578000000000
C -1.016202000000000 2.257679000000000 0.124202000000000
C -1.012358000000000 1.456569000000000 -1.023415000000000
C 5.814917000000000 2.696242000000000 1.470006000000000
O 4.878254000000000 1.624353000000000 1.261567000000000
O 3.488638000000000 3.067246000000000 0.231269000000000
O 3.414001000000000 -2.598820000000000 -0.435261000000000
C 3.496633000000000 -1.960549000000000 -3.027742000000000
O 2.376453000000000 -1.635298000000000 -2.178442000000000
C 2.473308000000000 -2.003271000000000 -0.896337000000000
C 3.738288000000000 1.943966000000000 0.620874000000000
C 1.195043000000000 -1.615778000000000 -0.149280000000000
C -1.345817000000000 -0.972908000000000 -1.583163000000000
C -0.465176000000000 0.080656000000000 -0.974708000000000
C 0.747660000000000 -0.197331000000000 -0.424912000000000
C 1.680761000000000 0.875465000000000 -0.131990000000000
C 2.873005000000000 0.763540000000000 0.474864000000000
H -1.611039000000000 -1.085999000000000 2.154764000000000
H -2.314388000000000 0.708395000000000 3.723458000000000
H -4.110263000000000 2.279782000000000 3.070522000000000
H -5.220323000000000 2.059317000000000 0.871274000000000
H -4.537939000000000 0.251100000000000 -0.681519000000000
H 1.928334000000000 -4.185953000000000 1.240740000000000
H 0.147753000000000 -3.971967000000000 1.248607000000000
H 1.060936000000000 -3.955497000000000 2.774719000000000
H -1.585274000000000 1.379390000000000 -3.101240000000000
H -2.549271000000000 3.631892000000000 -3.137748000000000
H -2.561070000000000 5.021676000000000 -1.084577000000000
H -1.578584000000000 4.122870000000000 1.008675000000000
H -0.613669000000000 1.862680000000000 1.045887000000000
H 6.657307000000000 2.249666000000000 1.990200000000000
H 5.363305000000000 3.480890000000000 2.076032000000000
H 6.132359000000000 3.112767000000000 0.514714000000000
H 4.397451000000000 -1.474963000000000 -2.655804000000000
H 3.644380000000000 -3.038866000000000 -3.057775000000000
H 3.233989000000000 -1.580874000000000 -4.010413000000000
H 0.436172000000000 -2.315544000000000 -0.492740000000000
H -0.797906000000000 -1.810070000000000 -2.014168000000000
H -1.976815000000000 -0.541156000000000 -2.355185000000000
H 1.410460000000000 1.859824000000000 -0.489688000000000
H 3.245185000000000 -0.161950000000000 0.889454000000000
40
R6_SMe1_-1_1 charge=-1 multiplicity=1 wB97MV_TZVPD_SMD_chcl3=-1357.477498950752, B3LYP_D3BJ_TZVPD=-1357.262235527597, B3LYP_TZVP_CPCM_chcl3_freq=0.26574039, B3LYP_TZVPD_CPCM_chcl3=-1357.332424386101, M062X_TZVPD=-1357.360423892491, COSMORS=-0.009326839262015945, M062X_TZVPD_CPCM_chcl3=-1357.433550526727, M062X_TZVPD_SMD_chcl3=-1357.436126554269, wB97MV_TZVPD_CPCM_chcl3=-1357.474661308705, B3LYP_D3BJ_TZVPD_SMD_chcl3=-1357.334896253532, wB97MV_TZVPD=-1357.40269339673
O 3.564291000000000 -0.361462000000000 -1.557441000000000
C 3.071182000000000 0.553533000000000 -0.701522000000000
C -2.916362000000000 1.519682000000000 0.298569000000000
H 4.576569000000000 0.899079000000000 -2.874657000000000
C 0.808770000000000 1.021084000000000 0.209659000000000

C 1.872258000000000 0.026188000000000 0.037217000000000
H 5.568892000000000 0.206851000000000 -1.570307000000000
O 3.565853000000000 1.650866000000000 -0.556180000000000
C 4.756029000000000 0.011288000000000 -2.269373000000000
H 4.995679000000000 -0.838434000000000 -2.902645000000000
H -5.427633000000000 3.606363000000000 0.612512000000000
H -1.205084000000000 2.759886000000000 0.501756000000000
C -0.534881000000000 0.742819000000000 0.203468000000000
H 1.126890000000000 2.047448000000000 0.346619000000000
C -1.530833000000000 1.737642000000000 0.353017000000000
C -0.970508000000000 -0.675362000000000 0.039300000000000
C -0.893374000000000 -1.548860000000000 1.052590000000000
H 1.526226000000000 -0.887151000000000 -0.436681000000000
S 2.644207000000000 -0.693585000000000 1.656290000000000
C -1.497830000000000 -1.108924000000000 -1.273752000000000
H -0.443306000000000 -3.639721000000000 -0.868087000000000
H -0.518978000000000 -1.195976000000000 2.007199000000000
C -2.184837000000000 -2.455577000000000 -1.307819000000000
O -1.381602000000000 -0.429185000000000 -2.282472000000000
H -2.277612000000000 -3.080385000000000 1.502958000000000
C -1.443201000000000 -3.481663000000000 -0.451956000000000
H -3.196129000000000 -2.305096000000000 -0.913255000000000
H -2.272909000000000 -2.773473000000000 -2.347050000000000
C -1.313907000000000 -2.982255000000000 0.983760000000000
H -1.958927000000000 -4.443776000000000 -0.480529000000000
H -0.607946000000000 -3.597821000000000 1.548311000000000
C 2.826660000000000 0.823344000000000 2.628128000000000
H 3.579117000000000 1.480445000000000 2.194680000000000
H 1.870788000000000 1.343604000000000 2.686001000000000
H 3.136157000000000 0.523592000000000 3.628748000000000
O -3.627698000000000 2.708125000000000 0.505975000000000
O -3.553526000000000 0.473163000000000 0.097995000000000
C -5.043632000000000 2.601158000000000 0.442539000000000
H -5.376027000000000 2.241614000000000 -0.533894000000000
H -5.431457000000000 1.926091000000000 1.208831000000000
38
R5_SMe6_cyc4SP1_0_1 charge=0 multiplicity=1 B3LYP_D3BJ_TZVPD=-1318.499128799793, B3LYP_D3BJ_TZVPD_SMD_chcl3=-1318.522291540037, B3LYP_TZVP_CPCM_chcl3_freq=0.25575624, B3LYP_TZVPD_CPCM_chcl3=-1318.516406312387, M062X_TZVPD_SMD_chcl3=-1318.62538809923, M062X_TZVPD=-1318.601454272795, COSMORS=-0.0027265564437067395, wB97MV_TZVPD_CPCM_chcl3=-1318.665016634086, wB97MV_TZVPD_SMD_chcl3=-1318.671049141828, wB97MV_TZVPD=-1318.647351724704, M062X_TZVPD_CPCM_chcl3=-1318.619581861531
C -1.515229000000000 1.570643000000000 -0.524418000000000
C -1.493112000000000 0.252148000000000 0.178657000000000
C -0.485107000000000 -0.622288000000000 0.124681000000000
C -0.496446000000000 -1.990681000000000 0.834681000000000
C 0.812543000000000 -0.598234000000000 -0.516997000000000
C 1.689805000000000 0.320883000000000 -1.299321000000000
C 1.483013000000000 -1.697573000000000 -0.148954000000000
O 0.824911000000000 -2.554092000000000 0.662347000000000
C 2.948557000000000 -0.567935000000000 -1.576449000000000
C 2.883054000000000 -1.791887000000000 -0.619564000000000
C -0.780575000000000 5.069223000000000 0.411607000000000
O -1.121015000000000 3.881205000000000 -0.326620000000000
O -1.034941000000000 2.692105000000000 1.577023000000000
O -1.284111000000000 -3.663323000000000 -0.731357000000000
C -3.816381000000000 -3.447753000000000 0.139989000000000
O -2.714207000000000 -2.734632000000000 0.740913000000000
C -1.516161000000000 -2.913453000000000 0.182737000000000
C -1.191072000000000 2.743257000000000 0.379668000000000
H -2.506861000000000 1.765013000000000 -0.946304000000000
H -2.354886000000000 0.012501000000000 0.789778000000000
H -0.697813000000000 -1.911287000000000 1.901518000000000
H 1.247417000000000 0.664997000000000 -2.234646000000000
H 2.907814000000000 -0.912520000000000 -2.609901000000000
H 3.867953000000000 0.000642000000000 -1.454294000000000
H 3.092709000000000 -2.733394000000000 -1.131095000000000
H 3.582360000000000 -1.714774000000000 0.218298000000000
H -0.767238000000000 5.872931000000000 -0.318878000000000
H 0.200491000000000 4.955000000000000 0.871276000000000
H -1.524824000000000 5.264961000000000 1.182344000000000
H -4.691955000000000 -3.163705000000000 0.715399000000000
H -3.645519000000000 -4.521005000000000 0.202982000000000
H -3.927038000000000 -3.151738000000000 -0.902014000000000
S 2.048146000000000 1.912587000000000 -0.434738000000000
C 2.545910000000000 1.333375000000000 1.208159000000000
H 2.759630000000000 2.225962000000000 1.794417000000000
H 1.737316000000000 0.780882000000000 1.683643000000000
H 3.443127000000000 0.717532000000000 1.158247000000000
H -0.818370000000000 1.602416000000000 -1.360507000000000
40
R6_SMe6_conformer47_-1_1 charge=-1 multiplicity=1 wB97MV_TZVPD_SMD_chcl3=-1357.48280949092, B3LYP_TZVP_CPCM_chcl3_freq=0.26710353, B3LYP_TZVPD_CPCM_chcl3=-1357.339084280049, B3LYP_D3BJ_TZVPD=-1357.266025789988, M062X_TZVPD=-1357.363398568206, COSMORS=-0.009782067448690679, M062X_TZVPD_CPCM_chcl3=-1357.439419301699, M062X_TZVPD_SMD_chcl3=-1357.440757434292, wB97MV_TZVPD_CPCM_chcl3=-1357.481202467119, B3LYP_D3BJ_TZVPD_SMD_chcl3=-1357.3404725845, wB97MV_TZVPD=-1357.40653208589
O 2.736829000000000 -0.064310000000000 3.966982000000000
C 2.355930000000000 0.685195000000000 2.908721000000000
C -2.666645000000000 -1.177770000000000 0.899173000000000
H 4.053603000000000 -0.337060000000000 5.475169000000000
C 0.610934000000000 0.694132000000000 1.193631000000000
C 1.159974000000000 0.122098000000000 2.273101000000000
H 4.770555000000000 0.390823000000000 4.008394000000000
O 2.961319000000000 1.681546000000000 2.562758000000000
C 3.906218000000000 0.379718000000000 4.671993000000000
H 3.750512000000000 1.379170000000000 5.077513000000000
H -5.247848000000000 -2.219943000000000 2.598388000000000
H -1.490082000000000 -0.201882000000000 2.379851000000000
C -0.607085000000000 0.234770000000000 0.504190000000000
H -4.274228000000000 -3.240880000000000 1.503900000000000
C -1.574298000000000 -0.331933000000000 1.309308000000000
C -0.688040000000000 0.488227000000000 -0.903238000000000
C 0.606764000000000 0.484845000000000 -1.669846000000000
H -5.241682000000000 -1.909827000000000 0.839856000000000
S 1.138680000000000 -1.298240000000000 -1.875253000000000
C -1.918126000000000 0.814203000000000 -1.541263000000000
H -0.664579000000000 1.281918000000000 -4.790094000000000
H 1.413383000000000 0.907320000000000 -1.066916000000000
C -1.916160000000000 1.172831000000000 -3.030204000000000
O -3.023607000000000 0.925701000000000 -0.967110000000000
H 1.484584000000000 1.031901000000000 -3.567162000000000
C -0.661020000000000 0.802512000000000 -3.807444000000000
H -2.803589000000000 0.704433000000000 -3.462432000000000
H -2.083119000000000 2.254975000000000 -3.087819000000000
C 0.564316000000000 1.212527000000000 -3.008160000000000
H -0.628952000000000 -0.277193000000000 -3.976859000000000
H 0.522979000000000 2.289296000000000 -2.804178000000000
C 2.886018000000000 -1.097805000000000 -2.333746000000000
H 3.400244000000000 -0.478582000000000 -1.597009000000000
H 3.335136000000000 -2.090751000000000 -2.336904000000000
H 2.998690000000000 -0.656712000000000 -3.323895000000000
O -3.565278000000000 -1.326866000000000 1.932484000000000
O -2.821156000000000 -1.784584000000000 -0.146467000000000
C -4.643883000000000 -2.231632000000000 1.693426000000000
H 1.104287000000000 1.574602000000000 0.796644000000000
H 0.758422000000000 -0.786772000000000 2.700516000000000
38
R5_SMe6_cyc4RP1_0_1 charge=0 multiplicity=1 B3LYP_D3BJ_TZVPD=-1318.499111627327, B3LYP_D3BJ_TZVPD_SMD_chcl3=-1318.521513931672, B3LYP_TZVP_CPCM_chcl3_freq=0.254771, B3LYP_TZVPD_CPCM_chcl3=-1318.51577336093, M062X_TZVPD_SMD_chcl3=-1318.623224610752, M062X_TZVPD=-1318.600215139342, COSMORS=-0.003322483701310242, wB97MV_TZVPD_CPCM_chcl3=-1318.663704653589, wB97MV_TZVPD_SMD_chcl3=-1318.669591634032, wB97MV_TZVPD=-1318.646728719941, M062X_TZVPD_CPCM_chcl3=-1318.617544949006
C -1.907680000000000 -0.847931000000000 0.853614000000000
C -0.690423000000000 -1.383442000000000 0.168336000000000
C 0.227786000000000 -0.626797000000000 -0.439213000000000
C 1.487641000000000 -1.179557000000000 -1.134966000000000
C 0.346922000000000 0.800651000000000 -0.636542000000000
C -0.316353000000000 2.074308000000000 -0.239323000000000
C 1.437053000000000 1.037225000000000 -1.376013000000000
O 2.145532000000000 -0.049164000000000 -1.755245000000000
C 0.439841000000000 3.135365000000000 -1.107342000000000
C 1.725231000000000 2.459944000000000 -1.666606000000000
C -5.431131000000000 -0.240521000000000 -0.090172000000000
O -4.229712000000000 -0.520441000000000 0.654153000000000
O -3.061575000000000 -0.543047000000000 -1.263906000000000
O 3.291396000000000 -1.184853000000000 0.480815000000000
C 2.834342000000000 -3.745295000000000 1.152366000000000
O 2.115382000000000 -3.068053000000000 0.099075000000000
C 2.425745000000000 -1.787008000000000 -0.102588000000000
C -3.098381000000000 -0.636835000000000 -0.059599000000000
H -1.701489000000000 0.125575000000000 1.310837000000000
H -0.570818000000000 -2.460040000000000 0.152949000000000
H 1.259159000000000 -1.910501000000000 -1.908388000000000
H -1.387301000000000 2.092095000000000 -0.444071000000000
H -0.206119000000000 3.428135000000000 -1.935149000000000
H 0.664239000000000 4.031607000000000 -0.532885000000000
H 1.867445000000000 2.651812000000000 -2.731963000000000
H 2.634518000000000 2.786240000000000 -1.153226000000000
H -5.331114000000000 0.701291000000000 -0.628184000000000
H -6.223106000000000 -0.174594000000000 0.650156000000000
H -5.634092000000000 -1.044261000000000 -0.796573000000000
H 2.437174000000000 -4.755393000000000 1.169869000000000
H 3.900129000000000 -3.754147000000000 0.931025000000000
H 2.658492000000000 -3.245017000000000 2.103375000000000
S -0.260812000000000 2.399896000000000 1.576677000000000
C 1.513125000000000 2.243576000000000 1.916927000000000
H 1.630795000000000 2.355203000000000 2.993867000000000
H 1.881708000000000 1.264702000000000 1.615109000000000
H 2.081711000000000 3.026123000000000 1.415585000000000
H -2.221280000000000 -1.503325000000000 1.667452000000000
44
R5_SMe4_P3_SMe6RS charge=0 multiplicity=1 B3LYP_D3BJ_TZVPD=-1757.195758283757, B3LYP_D3BJ_TZVPD_SMD_chcl3=-1757.221370995935, B3LYP_TZVP_CPCM_chcl3_freq=0.30109011, B3LYP_TZVPD_CPCM_chcl3=-1757.215187671948, M062X_TZVPD_SMD_chcl3=-1757.347161769203, M062X_TZVPD=-1757.321207964401, COSMORS=-0.0030703759538764294, wB97MV_TZVPD_CPCM_chcl3=-1757.378882560658, wB97MV_TZVPD_SMD_chcl3=-1757.385227429065, wB97MV_TZVPD=-1757.359355944341, M062X_TZVPD_CPCM_chcl3=-1757.341063132421
C -4.341136000000000 2.577481000000000 0.364400000000000
C 2.705580000000000 3.114536000000000 -1.659432000000000
C -0.866082000000000 0.681537000000000 -2.612548000000000
C 2.836880000000000 -0.811100000000000 2.528418000000000
C 0.051303000000000 0.615909000000000 0.912597000000000
C -1.796251000000000 -3.245030000000000 0.237761000000000
C -1.375253000000000 -3.219347000000000 -1.233942000000000
C -1.363483000000000 0.644167000000000 1.387819000000000
C 0.686137000000000 -0.365482000000000 0.273767000000000
C -0.745757000000000 -2.424170000000000 0.957849000000000
C -0.662817000000000 -1.864342000000000 -1.420667000000000
C -2.157727000000000 1.749738000000000 0.726936000000000
C 0.106479000000000 -1.704742000000000 -0.084368000000000
C 2.129648000000000 -0.211235000000000 -0.161474000000000
C 2.580997000000000 1.235586000000000 -0.229215000000000
O -0.569024000000000 -2.388651000000000 2.153336000000000
O -1.693123000000000 2.680855000000000 0.111060000000000
O 3.013109000000000 1.890184000000000 0.688878000000000
O -3.470268000000000 1.582471000000000 0.936019000000000
O 2.408350000000000 1.716124000000000 -1.466982000000000
S -1.898494000000000 -0.566308000000000 -1.797276000000000
S 3.273646000000000 -1.255400000000000 0.825040000000000
H -5.347528000000000 2.274648000000000 0.638085000000000
H -4.228683000000000 2.595978000000000 -0.718809000000000
H -4.112651000000000 3.560825000000000 0.772995000000000
H 2.495794000000000 3.313121000000000 -2.706020000000000
H 3.752197000000000 3.310355000000000 -1.432328000000000
H 2.068795000000000 3.721653000000000 -1.017842000000000
H -0.162942000000000 1.130863000000000 -1.917903000000000
H -1.546067000000000 1.448540000000000 -2.980373000000000
H -0.334979000000000 0.237135000000000 -3.454203000000000
H 1.793383000000000 -1.045225000000000 2.727875000000000
H 3.037245000000000 0.240935000000000 2.712902000000000
H 3.475053000000000 -1.425132000000000 3.163099000000000
H 0.595823000000000 1.530065000000000 1.112673000000000
H -1.886042000000000 -4.238092000000000 0.676241000000000
H -2.754208000000000 -2.734044000000000 0.382972000000000
H -2.208726000000000 -3.343600000000000 -1.922108000000000
H -0.655917000000000 -4.017848000000000 -1.428448000000000
H -1.388226000000000 0.843755000000000 2.466325000000000
H -1.902299000000000 -0.283895000000000 1.234140000000000
H 0.022621000000000 -1.885171000000000 -2.266085000000000
H 0.957554000000000 -2.398275000000000 -0.171834000000000
H 2.229430000000000 -0.625766000000000 -1.166058000000000
40
R6_SMe6_cyc4STS_-1_1 charge=-1 multiplicity=1 wB97MV_TZVPD_SMD_chcl3=-1357.438635199459, B3LYP_D3BJ_TZVPD=-1357.234209124454, B3LYP_TZVP_CPCM_chcl3_freq=0.26736077, B3LYP_TZVPD_CPCM_chcl3=-1357.298908057841, M062X_TZVPD=-1357.327747163561, COSMORS=-0.010524924758844102, M062X_TZVPD_CPCM_chcl3=-1357.394481755375, M062X_TZVPD_SMD_chcl3=-1357.397167044887, wB97MV_TZVPD_CPCM_chcl3=-1357.435713785045, B3LYP_D3BJ_TZVPD_SMD_chcl3=-1357.301418372266, wB97MV_TZVPD=-1357.370254289244
O -4.379568000000000 1.256745000000000 0.149315000000000
C -3.112843000000000 1.490909000000000 -0.349825000000000
C -1.124955000000000 -2.340921000000000 0.694784000000000
H -6.324929000000000 1.788076000000000 0.156917000000000
C -0.825633000000000 0.625811000000000 -0.186762000000000
C -2.158771000000000 0.581833000000000 0.189467000000000
H -5.206262000000000 3.159215000000000 -0.073105000000000
O -2.924951000000000 2.406108000000000 -1.154162000000000
C -5.409777000000000 2.118443000000000 -0.330728000000000
H -5.516625000000000 2.040350000000000 -1.414061000000000
H -2.835824000000000 -3.209248000000000 -1.906047000000000
H 0.507560000000000 -1.618695000000000 1.845953000000000
C 0.211054000000000 -0.211110000000000 0.211189000000000
H -3.551739000000000 -2.698138000000000 -0.350891000000000
C 0.058584000000000 -1.471474000000000 0.870346000000000
C 1.615803000000000 -0.026894000000000 -0.143870000000000
C 2.234604000000000 1.245450000000000 -0.602169000000000
H -2.621961000000000 -4.213870000000000 -0.442059000000000
S 2.959374000000000 2.273460000000000 0.788029000000000
C 2.225274000000000 -1.246579000000000 -0.278143000000000
H 5.094969000000000 -0.258035000000000 -1.897474000000000
H 1.485742000000000 1.895412000000000 -1.054276000000000
C 3.522679000000000 -1.443985000000000 -1.009352000000000
O 1.591625000000000 -2.322346000000000 0.132489000000000
H 3.924649000000000 1.920844000000000 -1.776512000000000
C 4.287580000000000 -0.129017000000000 -1.172900000000000
H 4.135796000000000 -2.180959000000000 -0.483831000000000
H 3.291050000000000 -1.878798000000000 -1.990040000000000
C 3.359052000000000 0.999893000000000 -1.613320000000000
H 4.752527000000000 0.140317000000000 -0.221389000000000
H 2.893617000000000 0.736021000000000 -2.568441000000000
C 1.577574000000000 2.319607000000000 1.959567000000000
H 1.914080000000000 2.902851000000000 2.816413000000000
H 0.703279000000000 2.797773000000000 1.519174000000000
H 1.315966000000000 1.314425000000000 2.284297000000000
O -1.501716000000000 -2.460721000000000 -0.588683000000000
O -1.668010000000000 -2.942363000000000 1.605984000000000
C -2.707253000000000 -3.198185000000000 -0.827213000000000
H -0.554245000000000 1.461058000000000 -0.826078000000000
H -2.516542000000000 -0.127829000000000 0.922706000000000
38
R5_SMe6H_cyc4S_0_1 charge=0 multiplicity=1 B3LYP_D3BJ_TZVPD_SMD_chcl3=-1318.488648652827, B3LYP_TZVP_CPCM_chcl3_freq=0.25460871, B3LYP_TZVPD_CPCM_chcl3=-1318.482578152159, M062X_TZVPD_SMD_chcl3=-1318.592037630989, M062X_TZVPD=-1318.566922507752, COSMORS=-0.0026615215690368667, wB97MV_TZVPD_CPCM_chcl3=-1318.626899474939, wB97MV_TZVPD_SMD_chcl3=-1318.6331456555, wB97MV_TZVPD=-1318.608339625076, M062X_TZVPD_CPCM_chcl3=-1318.585941386739
C -1.697746000000000 1.081867000000000 -0.411073000000000
C -1.134675000000000 -0.103410000000000 0.174206000000000
C 0.174982000000000 -0.420549000000000 0.220012000000000
C 0.718295000000000 -1.725941000000000 0.832835000000000
C 1.370125000000000 0.239350000000000 -0.254991000000000
C 1.803491000000000 1.513525000000000 -0.896901000000000
C 2.432402000000000 -0.497631000000000 0.093441000000000
O 2.162390000000000 -1.640769000000000 0.769054000000000
C 3.327975000000000 1.265414000000000 -1.145123000000000
C 3.757184000000000 0.059261000000000 -0.262377000000000
C -5.199986000000000 0.881957000000000 0.400344000000000
O -3.934089000000000 0.412076000000000 -0.095398000000000
O -3.582405000000000 2.457872000000000 -0.960659000000000
O 0.855286000000000 -3.389314000000000 -0.927080000000000
C -1.571732000000000 -4.373193000000000 -0.337813000000000
O -0.934546000000000 -3.338241000000000 0.439964000000000
C 0.258111000000000 -2.919423000000000 0.008628000000000
C -3.024504000000000 1.322776000000000 -0.474355000000000
H -1.039644000000000 1.837010000000000 -0.818370000000000
H -1.836110000000000 -0.802545000000000 0.614169000000000
H 0.430430000000000 -1.867323000000000 1.874196000000000
H 1.286298000000000 1.737424000000000 -1.830694000000000
H 3.468108000000000 1.010230000000000 -2.195953000000000
H 3.915980000000000 2.158186000000000 -0.941466000000000
H 4.383025000000000 -0.651301000000000 -0.806502000000000
H 4.312719000000000 0.363550000000000 0.630199000000000
H -5.052225000000000 1.589046000000000 1.218217000000000
H -5.712321000000000 -0.004554000000000 0.764420000000000
H -5.782103000000000 1.349148000000000 -0.392426000000000
H -0.957387000000000 -5.272014000000000 -0.339969000000000
H -1.726134000000000 -4.029529000000000 -1.359511000000000
H -2.522500000000000 -4.559360000000000 0.152105000000000
S 1.436826000000000 3.016689000000000 0.115362000000000
C 2.175910000000000 2.568949000000000 1.708399000000000
H 1.735747000000000 1.650258000000000 2.093133000000000
H 3.256605000000000 2.458398000000000 1.627533000000000
H 1.953275000000000 3.386750000000000 2.392434000000000
H -2.888238000000000 3.102579000000000 -1.153287000000000
41
R6_SMe6H_cyc4S_0_1 charge=0 multiplicity=1 B3LYP_D3BJ_TZVPD_SMD_chcl3=-1357.815558376505, B3LYP_TZVP_CPCM_chcl3_freq=0.28309176, B3LYP_TZVPD_CPCM_chcl3=-1357.808584448141, M062X_TZVPD_SMD_chcl3=-1357.923825092661, M062X_TZVPD=-1357.899254116285, COSMORS=-0.004626575565769858, wB97MV_TZVPD_CPCM_chcl3=-1357.953837307268, wB97MV_TZVPD_SMD_chcl3=-1357.961001708762, wB97MV_TZVPD=-1357.936738694091, M062X_TZVPD_CPCM_chcl3=-1357.91680545296
O -4.603117000000000 1.661940000000000 0.130336000000000
C -3.273514000000000 1.671301000000000 -0.075652000000000
C -1.112740000000000 -2.412293000000000 0.098989000000000
H -6.250678000000000 0.709267000000000 0.800573000000000
C -0.985329000000000 0.794551000000000 -0.010569000000000
C -2.396417000000000 0.697898000000000 0.249778000000000
H -5.004363000000000 -0.380352000000000 0.131402000000000
O -2.889747000000000 2.816997000000000 -0.676654000000000
C -5.185235000000000 0.506202000000000 0.741430000000000
H -4.776002000000000 0.355734000000000 1.741938000000000
H -1.552351000000000 -4.642431000000000 -1.277078000000000
H -0.801699000000000 -1.399228000000000 1.939262000000000
C -0.057750000000000 -0.132110000000000 0.301320000000000
H -0.352282000000000 -4.151199000000000 -2.508920000000000
C -0.289591000000000 -1.478310000000000 0.979627000000000
C 1.374707000000000 -0.083673000000000 0.083807000000000
C 2.174840000000000 0.956143000000000 -0.618975000000000
H -1.874099000000000 -3.230728000000000 -2.314520000000000
S 2.277753000000000 2.459030000000000 0.455801000000000

C 1.929601000000000 -1.181582000000000 0.620194000000000
H 5.203103000000000 -0.697732000000000 -0.115707000000000
H 1.650253000000000 1.285365000000000 -1.519103000000000
C 3.359713000000000 -1.561008000000000 0.614906000000000
O 1.025438000000000 -2.029238000000000 1.203738000000000
H 4.207852000000000 1.207147000000000 -1.322546000000000
C 4.206014000000000 -0.359425000000000 0.169968000000000
H 3.660654000000000 -1.907134000000000 1.607686000000000
H 3.499481000000000 -2.410929000000000 -0.064139000000000
C 3.556372000000000 0.397796000000000 -0.990081000000000
H 4.327445000000000 0.325199000000000 1.012849000000000
H 3.426981000000000 -0.278274000000000 -1.842404000000000
C 2.725584000000000 3.712245000000000 -0.777946000000000
H 1.960018000000000 3.777922000000000 -1.551317000000000
H 2.785094000000000 4.665058000000000 -0.253186000000000
H 3.692750000000000 3.497869000000000 -1.231332000000000
O -0.390745000000000 -2.957980000000000 -0.876064000000000
O -2.299211000000000 -2.588012000000000 0.253953000000000
C -1.101111000000000 -3.803258000000000 -1.804085000000000
H -0.629855000000000 1.703037000000000 -0.481964000000000
H -2.784821000000000 -0.192445000000000 0.716710000000000
H -3.672762000000000 3.365228000000000 -0.829236000000000
38
R5_SMe4_P3_0_1 charge=0 multiplicity=1 B3LYP_D3BJ_TZVPD=-1318.5195415659, CCSDT_SMD_chcl3=-1316.776971814885, CCSDT=-1316.755740110551, B3LYP_D3BJ_TZVPD_SMD_chcl3=-1318.541747507501, B3LYP_TZVP_CPCM_chcl3_freq=0.25110518, B3LYP_TZVPD_CPCM_chcl3=-1318.53624628395, M062X_TZVPD_SMD_chcl3=-1318.636749007968, M062X_TZVPD=-1318.614201945302, COSMORS=-0.002902776890680387, wB97MV_TZVPD_CPCM_chcl3=-1318.678605090767, wB97MV_TZVPD_SMD_chcl3=-1318.684292135655, wB97MV_TZVPD=-1318.661812086565, M062X_TZVPD_CPCM_chcl3=-1318.631256237659
C -2.030299000000000 0.130468000000000 -1.540825000000000
C -0.680682000000000 0.532261000000000 -1.016094000000000
C 0.202217000000000 -0.270219000000000 -0.412270000000000
C 1.555448000000000 0.207306000000000 0.059193000000000
C -0.047304000000000 -1.696875000000000 -0.142977000000000
C -1.121105000000000 -2.244949000000000 0.450405000000000
C 0.906969000000000 -2.776750000000000 -0.493665000000000
O 1.976361000000000 -2.647125000000000 -1.061919000000000
C -1.047443000000000 -3.734201000000000 0.577972000000000
C 0.309748000000000 -4.096911000000000 -0.034484000000000
C -4.306797000000000 2.557116000000000 0.087734000000000
O -3.353973000000000 1.940113000000000 -0.802324000000000
O -3.670855000000000 -0.028617000000000 0.231599000000000
O 1.304058000000000 2.618131000000000 -0.110612000000000
C 3.737252000000000 2.931089000000000 -1.138511000000000
O 3.190341000000000 1.633612000000000 -0.829163000000000
C 1.960083000000000 1.618275000000000 -0.301103000000000
C -3.112639000000000 0.638449000000000 -0.609711000000000
H -2.133114000000000 -0.948534000000000 -1.618990000000000
H -0.432151000000000 1.576320000000000 -1.141959000000000
H 2.313917000000000 -0.473052000000000 -0.322791000000000
H 0.335450000000000 1.980723000000000 2.182737000000000
H -1.132002000000000 -4.032046000000000 1.626584000000000
H -1.885261000000000 -4.206274000000000 0.057075000000000
H 0.989578000000000 -4.559917000000000 0.683150000000000
H 0.232327000000000 -4.772010000000000 -0.888048000000000
H -4.350433000000000 3.599605000000000 -0.212750000000000
H -3.967527000000000 2.468599000000000 1.118736000000000
H -5.282253000000000 2.085476000000000 -0.019931000000000
H 4.723751000000000 2.738357000000000 -1.549124000000000
H 3.808570000000000 3.532710000000000 -0.233416000000000
H 3.109804000000000 3.439584000000000 -1.868835000000000
S 1.744449000000000 0.033094000000000 1.888289000000000
C 0.297184000000000 0.939402000000000 2.494202000000000
H -0.624120000000000 0.475714000000000 2.145285000000000
H 0.343758000000000 0.878945000000000 3.580948000000000
H -2.182081000000000 0.581133000000000 -2.523434000000000
H -1.968499000000000 -1.680080000000000 0.815699000000000
44
R5_SMe4_P1S_SMe6RS charge=0 multiplicity=1 B3LYP_D3BJ_TZVPD=-1757.194569854492, B3LYP_D3BJ_TZVPD_SMD_chcl3=-1757.220999794044, B3LYP_TZVP_CPCM_chcl3_freq=0.30026533, B3LYP_TZVPD_CPCM_chcl3=-1757.214768703976, M062X_TZVPD_SMD_chcl3=-1757.346410448544, M062X_TZVPD=-1757.319625840916, COSMORS=-0.0032373375764974187, wB97MV_TZVPD_CPCM_chcl3=-1757.378040443341, wB97MV_TZVPD_SMD_chcl3=-1757.384452553282, wB97MV_TZVPD=-1757.35777274708, M062X_TZVPD_CPCM_chcl3=-1757.340246156807
C -4.988040000000000 -3.064662000000000 0.307082000000000
C 4.490963000000000 -1.401778000000000 1.594493000000000
C -3.211760000000000 3.293056000000000 -0.340281000000000
C 2.807435000000000 -2.638900000000000 -2.224071000000000
C -0.787334000000000 -0.746973000000000 0.304533000000000
C 1.055628000000000 3.783960000000000 0.261149000000000
C -0.205101000000000 3.572673000000000 1.106487000000000
C -1.911172000000000 -1.166716000000000 -0.269612000000000
C 0.260430000000000 0.062880000000000 -0.401513000000000
C 1.454448000000000 2.398387000000000 -0.217225000000000
C -0.800572000000000 2.252730000000000 0.586260000000000
C -2.908592000000000 -1.949817000000000 0.482762000000000
C 0.453901000000000 1.392663000000000 0.346848000000000
C 1.583891000000000 -0.714543000000000 -0.577247000000000
C 2.345041000000000 -0.964365000000000 0.705365000000000
O 2.407519000000000 2.150310000000000 -0.919980000000000
O -2.841432000000000 -2.256179000000000 1.654826000000000
O 1.859450000000000 -1.065696000000000 1.809733000000000
O -3.940961000000000 -2.294222000000000 -0.309434000000000
O 3.656551000000000 -1.083302000000000 0.462829000000000
S -1.725677000000000 2.478696000000000 -0.982625000000000
S 1.235667000000000 -2.339895000000000 -1.368000000000000
H -5.436833000000000 -2.505134000000000 1.127000000000000
H -4.592457000000000 -4.007010000000000 0.684189000000000
H -5.718119000000000 -3.244001000000000 -0.476858000000000
H 4.424123000000000 -0.614298000000000 2.343519000000000
H 5.500197000000000 -1.469502000000000 1.199860000000000
H 4.184150000000000 -2.350849000000000 2.031555000000000
H -3.705401000000000 2.653529000000000 0.391294000000000
H -3.875871000000000 3.445230000000000 -1.189823000000000
H -2.978785000000000 4.258767000000000 0.105812000000000
H 2.672933000000000 -3.565755000000000 -2.780709000000000
H 3.624945000000000 -2.755859000000000 -1.516595000000000
H 3.023842000000000 -1.827522000000000 -2.918062000000000
H -0.600450000000000 -0.988212000000000 1.343862000000000
H 1.884740000000000 4.245492000000000 0.798898000000000
H 0.862317000000000 4.394395000000000 -0.623384000000000
H -0.904742000000000 4.403201000000000 1.043588000000000
H 0.058867000000000 3.441995000000000 2.159151000000000
H -2.136485000000000 -0.949244000000000 -1.305143000000000
H -0.089198000000000 0.276094000000000 -1.412453000000000
H -1.478962000000000 1.787169000000000 1.299161000000000
H 0.839591000000000 1.167109000000000 1.350349000000000
H 2.224819000000000 -0.163882000000000 -1.258070000000000
38
R5_SMe1_P2A_0_1 charge=0 multiplicity=1 B3LYP_D3BJ_TZVPD=-1318.517720240714, CCSDT_SMD_chcl3=-1316.778856205732, CCSDT=-1316.753700531227, B3LYP_D3BJ_TZVPD_SMD_chcl3=-1318.544046220273, B3LYP_TZVP_CPCM_chcl3_freq=0.25138593, B3LYP_TZVPD_CPCM_chcl3=-1318.53750497469, M062X_TZVPD_SMD_chcl3=-1318.639821498076, M062X_TZVPD=-1318.613025856778, COSMORS=-0.0020313637525529713, wB97MV_TZVPD_CPCM_chcl3=-1318.679330370712, wB97MV_TZVPD_SMD_chcl3=-1318.686070078335, wB97MV_TZVPD=-1318.659319025208, M062X_TZVPD_CPCM_chcl3=-1318.633300161623
O 3.556780000000000 0.825175000000000 -0.938985000000000
C 2.759727000000000 -0.229311000000000 -0.738261000000000
H 4.414259000000000 0.279516000000000 -2.760293000000000
H 5.228596000000000 1.557775000000000 -1.810877000000000
C 0.377409000000000 -0.613021000000000 -0.106654000000000
C 1.640090000000000 0.105576000000000 0.223552000000000
O -1.105192000000000 -3.060399000000000 -0.935381000000000
O 2.936449000000000 -1.316375000000000 -1.238280000000000
C 4.716984000000000 0.601149000000000 -1.765023000000000
H 5.357066000000000 -0.155371000000000 -1.313130000000000
H 1.520368000000000 1.185791000000000 0.251270000000000
S 2.268656000000000 -0.285013000000000 1.928354000000000
C -0.855887000000000 -0.190957000000000 0.204108000000000
H 0.493811000000000 -1.546921000000000 -0.634395000000000
H -2.984257000000000 4.212913000000000 -0.844355000000000
C -2.043785000000000 -0.939110000000000 -0.209301000000000
H 2.884207000000000 -2.501904000000000 1.156282000000000
H 2.595301000000000 -2.399192000000000 2.907736000000000
C -1.097656000000000 1.085189000000000 0.968743000000000
C -3.318672000000000 -0.501886000000000 -0.152526000000000
H -3.574952000000000 -2.906416000000000 -2.153114000000000
C -4.331035000000000 -1.476609000000000 -0.662185000000000
H -3.621091000000000 0.468770000000000 0.218176000000000
C -3.497669000000000 -2.688362000000000 -1.086304000000000
H -5.059367000000000 -1.719015000000000 0.117162000000000
H -4.901145000000000 -1.042999000000000 -1.488645000000000
C -2.056786000000000 -2.321810000000000 -0.766422000000000
H -3.760895000000000 -3.602140000000000 -0.551370000000000
C 2.230155000000000 -2.098364000000000 1.926463000000000
H 1.214641000000000 -2.465061000000000 1.790173000000000
H -1.452944000000000 4.914796000000000 -0.268382000000000
H -2.927600000000000 5.051975000000000 0.733920000000000
C -1.399513000000000 2.275958000000000 0.081996000000000
O -1.045184000000000 2.400338000000000 -1.066093000000000
C -2.378763000000000 4.428092000000000 0.034801000000000
O -2.090060000000000 3.209918000000000 0.750856000000000
H -0.220907000000000 1.354967000000000 1.560463000000000
H -1.910771000000000 0.966478000000000 1.684417000000000
44
R5_SMe6_P2_SMe3SR charge=0 multiplicity=1 B3LYP_D3BJ_TZVPD=-1757.192141775801, B3LYP_D3BJ_TZVPD_SMD_chcl3=-1757.218572891243, B3LYP_TZVP_CPCM_chcl3_freq=0.30159878, B3LYP_TZVPD_CPCM_chcl3=-1757.213227676559, M062X_TZVPD_SMD_chcl3=-1757.344688223767, M062X_TZVPD=-1757.317879757132, COSMORS=-0.0036200409617453953, wB97MV_TZVPD_CPCM_chcl3=-1757.376717230555, wB97MV_TZVPD_SMD_chcl3=-1757.382200177219, wB97MV_TZVPD=-1757.355516888631, M062X_TZVPD_CPCM_chcl3=-1757.339466103591
C 5.590917000000000 -1.411771000000000 0.950353000000000
C -3.965082000000000 -2.968968000000000 1.232938000000000
C -0.106275000000000 3.505075000000000 2.516103000000000
C -0.172987000000000 -3.154659000000000 -1.170968000000000
C -0.916311000000000 3.190240000000000 -1.690685000000000
C -1.684375000000000 3.227917000000000 -0.368858000000000
C 2.008750000000000 -0.920101000000000 0.392613000000000
C 1.130506000000000 -0.325848000000000 -0.415972000000000
C -0.865705000000000 -0.962622000000000 1.003394000000000
C -0.547962000000000 1.732935000000000 -1.898043000000000
C -1.162865000000000 2.014365000000000 0.419871000000000
C 3.454150000000000 -0.798452000000000 0.139982000000000
C -2.346257000000000 -1.260247000000000 1.019453000000000
C -1.038347000000000 0.941203000000000 -0.681438000000000
C -0.360895000000000 -0.405575000000000 -0.340807000000000
O 0.017649000000000 1.290577000000000 -2.869156000000000
O 3.974007000000000 -0.197246000000000 -0.776814000000000
O -3.227151000000000 -0.440297000000000 0.890970000000000
O 4.158936000000000 -1.454884000000000 1.082144000000000
O -2.581831000000000 -2.563064000000000 1.218720000000000
S 0.446383000000000 2.342661000000000 1.238047000000000
S -0.850705000000000 -1.570027000000000 -1.726495000000000
H 5.978058000000000 -1.987243000000000 1.786262000000000
H 5.944608000000000 -0.382567000000000 0.998244000000000
H 5.897678000000000 -1.857386000000000 0.004771000000000
H -4.497702000000000 -2.464815000000000 2.037849000000000
H -3.950159000000000 -4.042219000000000 1.396870000000000
H -4.432830000000000 -2.732299000000000 0.278488000000000
H -0.458702000000000 4.440451000000000 2.084579000000000
H 0.759049000000000 3.708315000000000 3.145277000000000
H -0.892593000000000 3.054064000000000 3.121304000000000
H -0.304661000000000 -3.831414000000000 -2.014632000000000
H -0.707944000000000 -3.546525000000000 -0.309272000000000
H 0.891216000000000 -3.065047000000000 -0.954485000000000
H 0.018979000000000 3.752336000000000 -1.633429000000000
H -1.470833000000000 3.566602000000000 -2.550469000000000
H -2.753817000000000 3.086545000000000 -0.545538000000000
H -1.562463000000000 4.163831000000000 0.171248000000000
H 1.721848000000000 -1.488444000000000 1.265054000000000
H 1.514927000000000 0.227182000000000 -1.263102000000000
H -0.330876000000000 -1.873765000000000 1.250857000000000
H -0.652007000000000 -0.232907000000000 1.786777000000000
H -1.861656000000000 1.700757000000000 1.191005000000000
H -2.081857000000000 0.713030000000000 -0.936048000000000
44
R5_SMe4_P2_SMe2RP2S charge=0 multiplicity=1 B3LYP_D3BJ_TZVPD=-1757.198063805925, B3LYP_D3BJ_TZVPD_SMD_chcl3=-1757.223735891444, B3LYP_TZVP_CPCM_chcl3_freq=0.30041262, B3LYP_TZVPD_CPCM_chcl3=-1757.217179375686, M062X_TZVPD=-1757.322672734808, COSMORS=-0.003448505702998395, wB97MV_TZVPD_CPCM_chcl3=-1757.379928571207, wB97MV_TZVPD_SMD_chcl3=-1757.386669050335, wB97MV_TZVPD=-1757.360549233427
C 5.694841000000000 0.112719000000000 1.573124000000000
C -4.375385000000000 1.581017000000000 1.191776000000000
C 1.295391000000000 -0.341099000000000 -3.052134000000000
C -1.697652000000000 3.314797000000000 -1.531689000000000
C -1.873393000000000 -3.524447000000000 -0.372244000000000
C -1.169858000000000 -2.345336000000000 -1.066193000000000
C -2.513976000000000 -2.910060000000000 0.876600000000000
C 2.036724000000000 -0.057258000000000 1.022845000000000
C -0.624215000000000 -1.508939000000000 0.109891000000000
C -1.648689000000000 -1.717534000000000 1.227706000000000
C 1.301486000000000 0.121260000000000 -0.309803000000000
C 3.444902000000000 0.488275000000000 0.957676000000000
C -0.183957000000000 -0.095200000000000 -0.167188000000000
C -1.024365000000000 0.958141000000000 -0.214923000000000
C -2.493186000000000 0.795296000000000 0.009175000000000
O -1.733753000000000 -1.052717000000000 2.235031000000000
O 3.745650000000000 1.550165000000000 0.464101000000000
O -3.185620000000000 -0.029275000000000 -0.547520000000000
O 4.323460000000000 -0.333676000000000 1.543380000000000
O -2.968452000000000 1.672857000000000 0.894279000000000
S 2.066259000000000 -0.995554000000000 -1.547469000000000
S -0.450036000000000 2.626159000000000 -0.403170000000000
H 5.773804000000000 1.048233000000000 2.124923000000000
H 6.064559000000000 0.253172000000000 0.558611000000000
H 6.245500000000000 -0.676494000000000 2.076082000000000
H -4.962259000000000 1.731335000000000 0.286840000000000
H -4.569086000000000 2.370991000000000 1.910903000000000
H -4.602665000000000 0.606602000000000 1.621065000000000
H 0.213927000000000 -0.464086000000000 -3.016836000000000
H 1.695056000000000 -0.915260000000000 -3.886515000000000
H 1.546979000000000 0.710652000000000 -3.181038000000000
H -1.740197000000000 2.737022000000000 -2.452911000000000
H -1.362800000000000 4.327030000000000 -1.753763000000000
H -2.676843000000000 3.357734000000000 -1.058873000000000
H -1.134443000000000 -4.274873000000000 -0.080856000000000
H -2.600056000000000 -4.010364000000000 -1.022182000000000
H -1.882841000000000 -1.752651000000000 -1.635577000000000
H -0.370677000000000 -2.666477000000000 -1.730437000000000
H -2.614051000000000 -3.582914000000000 1.728433000000000
H -3.507298000000000 -2.507547000000000 0.655051000000000
H 1.508617000000000 0.514699000000000 1.790909000000000
H 2.055722000000000 -1.097378000000000 1.344175000000000
H 0.252371000000000 -2.056797000000000 0.481243000000000
H 1.488789000000000 1.133318000000000 -0.659646000000000
44
R5_SMe4_P2_SMe2SP2S charge=0 multiplicity=1 B3LYP_D3BJ_TZVPD=-1757.201645503044, B3LYP_D3BJ_TZVPD_SMD_chcl3=-1757.225729178244, B3LYP_TZVP_CPCM_chcl3_freq=0.3016305, B3LYP_TZVPD_CPCM_chcl3=-1757.219467999746, M062X_TZVPD_SMD_chcl3=-1757.351679041005, M062X_TZVPD=-1757.326924981857, COSMORS=-0.004652646885289562, wB97MV_TZVPD_CPCM_chcl3=-1757.382895941379, wB97MV_TZVPD_SMD_chcl3=-1757.389269169965, wB97MV_TZVPD=-1757.364668140797, M062X_TZVPD_CPCM_chcl3=-1757.34557370093
C -4.224280000000000 -0.544064000000000 2.747819000000000
C 4.295106000000000 1.068623000000000 0.870386000000000
C 0.208838000000000 0.769674000000000 -3.409143000000000
C 0.759501000000000 3.438512000000000 1.258584000000000
C 0.439499000000000 -3.430102000000000 1.229587000000000
C -0.311405000000000 -2.086932000000000 1.194683000000000
C 1.785123000000000 -3.142329000000000 0.556140000000000
C -2.664347000000000 -0.349898000000000 -0.595565000000000
C 0.104438000000000 -1.473775000000000 -0.158144000000000
C 1.524484000000000 -1.989917000000000 -0.391311000000000
C -1.404258000000000 0.334071000000000 -1.125898000000000
C -2.998689000000000 0.058566000000000 0.818557000000000
C -0.147311000000000 -0.003488000000000 -0.367107000000000
C 0.709372000000000 0.965906000000000 0.011837000000000
C 1.980670000000000 0.647616000000000 0.729047000000000
O 2.295212000000000 -1.567475000000000 -1.222820000000000
O -2.618186000000000 1.067566000000000 1.363572000000000
O 2.064983000000000 -0.119236000000000 1.664737000000000
O -3.804774000000000 -0.839726000000000 1.400227000000000
O 3.024870000000000 1.317145000000000 0.237937000000000
S -1.247999000000000 -0.175484000000000 -2.889405000000000
S 0.454971000000000 2.681718000000000 -0.366034000000000
H -4.778962000000000 0.392712000000000 2.773094000000000
H -3.357044000000000 -0.474328000000000 3.402699000000000
H -4.860205000000000 -1.373573000000000 3.041965000000000
H 4.247590000000000 1.332401000000000 1.925912000000000
H 5.005359000000000 1.702226000000000 0.347947000000000
H 4.566348000000000 0.019418000000000 0.764962000000000
H 1.086434000000000 0.472685000000000 -2.838498000000000
H 0.034247000000000 1.839623000000000 -3.309590000000000
H 0.363694000000000 0.526691000000000 -4.459603000000000
H 0.062236000000000 3.045458000000000 1.995648000000000
H 0.586537000000000 4.505490000000000 1.125268000000000
H 1.787967000000000 3.281118000000000 1.579475000000000
H -0.113103000000000 -4.175983000000000 0.653437000000000
H 0.549575000000000 -3.811080000000000 2.244114000000000
H 0.018729000000000 -1.436903000000000 2.002357000000000
H -1.388313000000000 -2.220589000000000 1.277599000000000
H 2.228560000000000 -3.986459000000000 0.027807000000000
H 2.522680000000000 -2.780044000000000 1.278434000000000
H -2.605796000000000 -1.436181000000000 -0.653073000000000
H -3.513907000000000 -0.056447000000000 -1.219436000000000
H -0.454967000000000 -2.004909000000000 -0.941610000000000
H -1.559485000000000 1.408290000000000 -1.109936000000000
38
R5_SMe6_cyc4SPZ1_0_1 charge=0 multiplicity=1 B3LYP_D3BJ_TZVPD_SMD_chcl3=-1318.522785814827, B3LYP_TZVP_CPCM_chcl3_freq=0.2559607, B3LYP_TZVPD_CPCM_chcl3=-1318.516179561613, M062X_TZVPD_SMD_chcl3=-1318.625844539301, M062X_TZVPD=-1318.600854907547, COSMORS=-0.002808961574046831, wB97MV_TZVPD_CPCM_chcl3=-1318.664891819226, wB97MV_TZVPD_SMD_chcl3=-1318.671656456277, wB97MV_TZVPD=-1318.646833813552, M062X_TZVPD_CPCM_chcl3=-1318.619283790061
C -2.214745000000000 0.506794000000000 0.769917000000000
C -0.918361000000000 0.960737000000000 0.182937000000000
C 0.238867000000000 0.294256000000000 0.234876000000000
C 0.494880000000000 -1.094125000000000 0.849200000000000
C 1.549159000000000 0.684292000000000 -0.230199000000000
C 2.213723000000000 1.839898000000000 -0.888312000000000
C 2.440597000000000 -0.212634000000000 0.201810000000000
O 1.933387000000000 -1.254423000000000 0.902754000000000
C 3.698640000000000 1.352535000000000 -0.999677000000000
C 3.858896000000000 0.098348000000000 -0.088984000000000
C -5.312716000000000 -1.068109000000000 -0.496586000000000
O -4.284969000000000 -0.536143000000000 0.362191000000000
O -3.029529000000000 -0.080071000000000 -1.443033000000000
O 0.533744000000000 -2.704093000000000 -0.961338000000000
C -2.036410000000000 -3.381997000000000 -0.586097000000000
O -1.336146000000000 -2.448994000000000 0.265670000000000
C -0.070160000000000 -2.183705000000000 -0.059065000000000
C -3.187490000000000 -0.059315000000000 -0.245458000000000
H -2.727109000000000 1.346683000000000 1.250455000000000
H -0.917785000000000 1.921461000000000 -0.317425000000000
H 0.098556000000000 -1.207597000000000 1.856647000000000

H 1.802035000000000 2.078042000000000 -1.870140000000000
H 3.896777000000000 1.077596000000000 -2.035532000000000
H 4.395551000000000 2.143792000000000 -0.730944000000000
H 4.374189000000000 -0.720373000000000 -0.595392000000000
H 4.412035000000000 0.310545000000000 0.830761000000000
H -4.924661000000000 -1.902011000000000 -1.079312000000000
H -5.680678000000000 -0.293917000000000 -1.168439000000000
H -6.102877000000000 -1.403242000000000 0.168873000000000
H -2.101926000000000 -2.980872000000000 -1.595704000000000
H -3.024606000000000 -3.483339000000000 -0.148880000000000
H -1.518410000000000 -4.338970000000000 -0.596456000000000
S 1.987741000000000 3.429883000000000 0.020709000000000
C 2.537500000000000 2.965850000000000 1.684270000000000
H 2.414558000000000 3.849818000000000 2.308558000000000
H 1.926074000000000 2.157876000000000 2.083525000000000
H 3.587181000000000 2.674112000000000 1.686741000000000
H -2.077051000000000 -0.248955000000000 1.544250000000000
41
R6_SMe4_P2_0_1 charge=0 multiplicity=1 B3LYP_D3BJ_TZVPD=-1357.815215409075, B3LYP_D3BJ_TZVPD_SMD_chcl3=-1357.841807128576, B3LYP_TZVP_CPCM_chcl3_freq=0.28148252, B3LYP_TZVPD_CPCM_chcl3=-1357.835518209111, M062X_TZVPD_SMD_chcl3=-1357.939459506786, M062X_TZVPD=-1357.912732369684, COSMORS=-0.0030644477565284285, wB97MV_TZVPD_CPCM_chcl3=-1357.976324903565, wB97MV_TZVPD_SMD_chcl3=-1357.982736946791, wB97MV_TZVPD=-1357.956068066259, M062X_TZVPD_CPCM_chcl3=-1357.93331014825
O -4.248482000000000 -0.082181000000000 0.909619000000000
C -3.266015000000000 -0.901648000000000 0.494919000000000
O -3.443998000000000 -2.074247000000000 0.233336000000000
H -6.203927000000000 0.138005000000000 1.372742000000000
H -5.542051000000000 -1.476847000000000 1.761963000000000
C -1.985840000000000 -0.178503000000000 0.410417000000000
C -0.884083000000000 -0.814824000000000 -0.010481000000000
C -5.557426000000000 -0.666332000000000 1.034270000000000
H -5.894190000000000 -1.048677000000000 0.071373000000000
H 1.600512000000000 -2.763044000000000 -1.804150000000000
H -2.004461000000000 0.857399000000000 0.711690000000000
O -0.364129000000000 1.710421000000000 -2.026257000000000
C 0.442026000000000 -0.248649000000000 -0.203209000000000
O -0.917114000000000 3.064151000000000 -0.321567000000000
C -1.218317000000000 2.470848000000000 -2.905651000000000
H -2.246865000000000 2.430710000000000 -2.550295000000000
H -0.883847000000000 3.505863000000000 -2.951462000000000
H -1.128323000000000 1.994564000000000 -3.877012000000000
C 0.631930000000000 1.232381000000000 0.046120000000000
C 1.482619000000000 -1.039566000000000 -0.567416000000000
C -0.315774000000000 2.111339000000000 -0.755712000000000
H 0.459676000000000 -2.947336000000000 -0.487655000000000
C 1.432080000000000 -2.539499000000000 -0.743628000000000
H 1.776058000000000 -0.323938000000000 2.317216000000000
O 3.048680000000000 0.470998000000000 -1.546149000000000
C 2.523394000000000 -3.242644000000000 0.075615000000000
H 3.930026000000000 -0.972219000000000 0.920155000000000
H 4.934469000000000 -0.810704000000000 -0.526370000000000
C 3.906870000000000 -2.737106000000000 -0.313356000000000
H 2.346433000000000 -3.064671000000000 1.140596000000000
H 2.451555000000000 -4.319549000000000 -0.089143000000000
C 3.996379000000000 -1.215141000000000 -0.147007000000000
H 4.680002000000000 -3.212594000000000 0.292847000000000
H 4.109758000000000 -2.998386000000000 -1.356178000000000
C 2.857761000000000 -0.489441000000000 -0.821749000000000
S 0.512076000000000 1.692475000000000 1.821781000000000
H 1.624056000000000 1.527917000000000 -0.285503000000000
C 1.919839000000000 0.746205000000000 2.457063000000000
H 2.848640000000000 1.066086000000000 1.985397000000000
H 1.968789000000000 0.961894000000000 3.523554000000000
H -1.015904000000000 -1.857322000000000 -0.261649000000000
43
R5_SMe1_P2A_SMe_complex_-1_1 charge=-1 multiplicity=1 wB97MV_TZVPD_SMD_chcl3=-1756.872547475774, B3LYP_D3BJ_TZVPD=-1756.621105617717, B3LYP_TZVP_CPCM_chcl3_freq=0.28298351, B3LYP_TZVPD_CPCM_chcl3=-1756.711081425862, M062X_TZVPD=-1756.741780229996, M062X_TZVPD_CPCM_chcl3=-1756.834956041288, M062X_TZVPD_SMD_chcl3=-1756.835450981094, wB97MV_TZVPD_CPCM_chcl3=-1756.871549950189, B3LYP_D3BJ_TZVPD_SMD_chcl3=-1756.711970033221, wB97MV_TZVPD=-1756.779433278513
O -4.442518000000000 -1.064593000000000 0.403945000000000
C -3.219393000000000 -1.281043000000000 -0.098942000000000
H -6.383307000000000 -1.630443000000000 0.464799000000000
H -5.599873000000000 -1.882600000000000 -1.123163000000000
C -0.868090000000000 -0.748449000000000 0.547253000000000
C -2.246098000000000 -0.216012000000000 0.352193000000000
O 1.040135000000000 -3.031200000000000 0.746629000000000
O -2.950926000000000 -2.193988000000000 -0.844850000000000
C -5.480806000000000 -1.957693000000000 -0.043000000000000
H -5.238692000000000 -2.984290000000000 0.227872000000000
H -2.640193000000000 0.268233000000000 1.244455000000000
S -2.344704000000000 1.140425000000000 -0.920073000000000
C 0.118719000000000 -0.117846000000000 1.201137000000000
H -0.664693000000000 -1.704240000000000 0.089728000000000
H 0.807241000000000 4.948886000000000 0.458133000000000
C 1.457492000000000 -0.697582000000000 1.284587000000000
H -0.246049000000000 0.399886000000000 -1.869045000000000
H -1.634063000000000 -0.464201000000000 -2.594589000000000
C -0.100955000000000 1.238346000000000 1.823162000000000
C 2.586948000000000 -0.054845000000000 1.640718000000000
H 3.626593000000000 -3.121967000000000 1.560513000000000
C 3.813337000000000 -0.907794000000000 1.592888000000000
H 2.639255000000000 0.989993000000000 1.915640000000000
C 3.316599000000000 -2.232442000000000 1.012475000000000
H 4.583591000000000 -0.444348000000000 0.970507000000000
H 4.242034000000000 -1.020957000000000 2.594292000000000
C 1.812593000000000 -2.115435000000000 0.977432000000000
H 3.664834000000000 -2.330028000000000 -0.030457000000000
C -1.269129000000000 0.492730000000000 -2.226438000000000
H -1.306521000000000 1.228435000000000 -3.028988000000000
H -0.852878000000000 5.446692000000000 0.900239000000000
H -0.564314000000000 4.471636000000000 -0.571002000000000
C 0.202514000000000 2.390035000000000 0.886542000000000
O 0.962044000000000 2.354856000000000 -0.050885000000000
C -0.244654000000000 4.666265000000000 0.452038000000000
O -0.456228000000000 3.497081000000000 1.264625000000000
H 0.542807000000000 1.368320000000000 2.697108000000000
H -1.123062000000000 1.353506000000000 2.179085000000000
C 2.645057000000000 -0.629019000000000 -2.239146000000000
H 2.701775000000000 0.401047000000000 -1.879939000000000
H 2.138666000000000 -0.616672000000000 -3.207949000000000
S 4.312975000000000 -1.393818000000000 -2.367488000000000
H 2.001936000000000 -1.177551000000000 -1.546120000000000
38
R5_SMe1_P2_rot_barrier1_0_1 charge=0 multiplicity=1 B3LYP_D3BJ_TZVPD=-1318.510131798675, B3LYP_D3BJ_TZVPD_SMD_chcl3=-1318.536251225664, B3LYP_TZVP_CPCM_chcl3_freq=0.25367858, B3LYP_TZVPD_CPCM_chcl3=-1318.529807758775, M062X_TZVPD_SMD_chcl3=-1318.631662478715, M062X_TZVPD=-1318.604807952821, COSMORS=-0.0023962506737281925, wB97MV_TZVPD_CPCM_chcl3=-1318.671710622411, wB97MV_TZVPD_SMD_chcl3=-1318.678350130006, wB97MV_TZVPD=-1318.651638292928, M062X_TZVPD_CPCM_chcl3=-1318.625242666459
O 4.083388000000000 -0.713225000000000 -1.294666000000000
C 3.183478000000000 0.264847000000000 -1.143434000000000
H 5.794720000000000 0.429072000000000 -1.624389000000000
H 5.851951000000000 -1.298915000000000 -2.081127000000000
C 0.780072000000000 0.492081000000000 -0.554318000000000
C 2.070001000000000 -0.157645000000000 -0.211502000000000
O 0.855072000000000 -2.558312000000000 0.837414000000000
O 3.277553000000000 1.352100000000000 -1.669029000000000
C 5.248813000000000 -0.395912000000000 -2.080264000000000
H 4.959098000000000 -0.127426000000000 -3.095148000000000
H 2.003571000000000 -1.237090000000000 -0.168563000000000
S 2.623387000000000 0.263784000000000 1.508038000000000
C -0.489188000000000 0.061305000000000 -0.467643000000000
H 0.909370000000000 1.501648000000000 -0.924687000000000
H -4.641530000000000 1.808541000000000 1.645352000000000
C -1.025925000000000 -1.229630000000000 0.002402000000000
H 3.196123000000000 2.495242000000000 0.728674000000000
H 1.537554000000000 2.433997000000000 1.392268000000000
C -1.548894000000000 1.054579000000000 -0.901885000000000
C -2.341892000000000 -1.540019000000000 -0.054151000000000
H -1.111045000000000 -4.387084000000000 0.542184000000000
C -2.709029000000000 -2.871439000000000 0.506377000000000
H -3.108189000000000 -0.898865000000000 -0.465736000000000
C -1.379872000000000 -3.434452000000000 1.000530000000000
H -3.450013000000000 -2.760299000000000 1.303116000000000
H -3.176946000000000 -3.496124000000000 -0.260247000000000
C -0.331441000000000 -2.397299000000000 0.632052000000000
H -1.354665000000000 -3.579170000000000 2.082259000000000
C 2.559574000000000 2.077516000000000 1.506398000000000
H 2.937352000000000 2.381021000000000 2.482230000000000
H -5.409743000000000 2.727002000000000 0.316707000000000
H -4.038012000000000 3.429309000000000 1.224312000000000
C -2.383970000000000 1.582518000000000 0.247169000000000
O -1.998580000000000 1.713695000000000 1.384485000000000
C -4.477872000000000 2.513649000000000 0.831794000000000
O -3.611894000000000 1.931274000000000 -0.163561000000000
H -2.210966000000000 0.641765000000000 -1.663117000000000
H -1.071413000000000 1.927963000000000 -1.350290000000000
38
R5_SMe6_cyc2RP1_0_1 charge=0 multiplicity=1 B3LYP_D3BJ_TZVPD=-1318.51119835053, B3LYP_D3BJ_TZVPD_SMD_chcl3=-1318.535059247145, B3LYP_TZVP_CPCM_chcl3_freq=0.25582747, B3LYP_TZVPD_CPCM_chcl3=-1318.529090597932, M062X_TZVPD_SMD_chcl3=-1318.635891789342, M062X_TZVPD=-1318.611633622782, COSMORS=-0.0037027648123730134, wB97MV_TZVPD_CPCM_chcl3=-1318.675582431194, wB97MV_TZVPD_SMD_chcl3=-1318.681692284143, wB97MV_TZVPD=-1318.657595069227, M062X_TZVPD_CPCM_chcl3=-1318.63002178894
C -2.093281000000000 0.798688000000000 0.348823000000000
C -1.263632000000000 0.422825000000000 -0.863662000000000
C 0.241225000000000 0.548646000000000 -0.635965000000000
C 0.826818000000000 1.743261000000000 -0.396734000000000
C 0.728733000000000 -0.793069000000000 -0.727458000000000
C 1.945713000000000 -1.606107000000000 -0.438634000000000
C -0.318925000000000 -1.593951000000000 -1.018919000000000
O -1.505376000000000 -0.997484000000000 -1.156173000000000
C 1.537458000000000 -3.021720000000000 -0.956867000000000
C -0.012085000000000 -3.040818000000000 -1.090216000000000
C -5.697338000000000 0.403030000000000 1.075278000000000
O -4.259600000000000 0.444409000000000 1.194998000000000
O -4.095163000000000 0.913714000000000 -0.996781000000000
O 3.148172000000000 1.178787000000000 -0.572248000000000
C 3.905989000000000 3.550315000000000 0.327050000000000
O 2.519696000000000 3.200569000000000 0.199585000000000
C 2.260113000000000 1.955840000000000 -0.274630000000000
C -3.578120000000000 0.721390000000000 0.078595000000000
H -1.867728000000000 1.832573000000000 0.625108000000000
H -1.589092000000000 0.976853000000000 -1.742849000000000
H 0.210755000000000 2.629167000000000 -0.315822000000000
H 2.843216000000000 -1.235653000000000 -0.925264000000000
H 1.984635000000000 -3.168729000000000 -1.940409000000000
H 1.904489000000000 -3.812649000000000 -0.306352000000000
H -0.349969000000000 -3.493756000000000 -2.024110000000000
H -0.505010000000000 -3.577206000000000 -0.274287000000000
H -5.991480000000000 -0.363431000000000 0.360183000000000
H -6.075063000000000 1.371979000000000 0.752254000000000
H -6.063194000000000 0.161503000000000 2.068614000000000
H 4.407145000000000 2.879110000000000 1.024060000000000
H 3.917467000000000 4.568190000000000 0.707332000000000
H 4.403789000000000 3.501091000000000 -0.641278000000000
S 2.420205000000000 -1.553211000000000 1.342662000000000
C 0.932348000000000 -2.208070000000000 2.145111000000000
H 0.759826000000000 -3.251771000000000 1.884750000000000
H 1.107214000000000 -2.136724000000000 3.217807000000000
H 0.057223000000000 -1.610996000000000 1.890647000000000
H -1.828744000000000 0.170861000000000 1.199118000000000
37
R5_SMe6_cyc4RTS_-1_1 charge=-1 multiplicity=1 wB97MV_TZVPD_SMD_chcl3=-1318.129099607318, B3LYP_D3BJ_TZVPD=-1317.919426253465, B3LYP_TZVP_CPCM_chcl3_freq=0.23949499, B3LYP_TZVPD_CPCM_chcl3=-1317.986268428207, M062X_TZVPD=-1318.011673599674, COSMORS=-0.010183798435104014, M062X_TZVPD_CPCM_chcl3=-1318.081122857904, M062X_TZVPD_SMD_chcl3=-1318.083163640584, wB97MV_TZVPD_CPCM_chcl3=-1318.126837427674, B3LYP_D3BJ_TZVPD_SMD_chcl3=-1317.988163226074, wB97MV_TZVPD=-1318.058699055661
C 2.515541000000000 -0.267523000000000 0.132816000000000
C 1.381733000000000 -1.040096000000000 0.339779000000000
C 0.049522000000000 -0.648047000000000 0.310196000000000
C -0.996876000000000 -1.598571000000000 0.549413000000000
C -0.520792000000000 0.683159000000000 0.462288000000000
C -0.107502000000000 2.085460000000000 0.208570000000000
C -1.632818000000000 0.614089000000000 1.230089000000000
O -2.140723000000000 -0.562117000000000 1.572789000000000
C -1.041876000000000 2.913120000000000 1.145959000000000
C -2.129443000000000 1.949215000000000 1.685707000000000
C 6.140927000000000 -0.362656000000000 -0.071457000000000
O 4.796048000000000 0.110099000000000 -0.078563000000000
O 4.135533000000000 -2.013533000000000 0.360272000000000
O -2.278089000000000 -1.247824000000000 -1.479177000000000
C -3.953158000000000 -3.222912000000000 -0.906173000000000
O -2.845196000000000 -2.890000000000000 -0.054663000000000
C -2.087297000000000 -1.847343000000000 -0.444876000000000
C 3.817354000000000 -0.838801000000000 0.158613000000000
H 2.443608000000000 0.787876000000000 -0.086531000000000
H 1.556131000000000 -2.103266000000000 0.492679000000000
H -0.740831000000000 -2.503875000000000 1.093127000000000
H 0.941042000000000 2.285976000000000 0.430030000000000
H -0.447163000000000 3.300306000000000 1.974393000000000
H -1.468791000000000 3.770757000000000 0.627251000000000
H -2.221828000000000 1.995623000000000 2.774634000000000
H -3.122572000000000 2.161313000000000 1.274761000000000
H 6.763688000000000 0.508761000000000 -0.265366000000000
H 6.300925000000000 -1.112575000000000 -0.848266000000000
H 6.402588000000000 -0.798517000000000 0.894357000000000
H -4.437265000000000 -4.075117000000000 -0.437132000000000
H -4.643808000000000 -2.382781000000000 -0.974760000000000
H -3.601979000000000 -3.484972000000000 -1.903810000000000
S -0.196852000000000 2.583531000000000 -1.581154000000000
C -1.933118000000000 2.210600000000000 -1.948906000000000
H -2.602441000000000 2.864889000000000 -1.389564000000000
H -2.075493000000000 2.385667000000000 -3.014843000000000
H -2.148660000000000 1.168771000000000 -1.719367000000000
41
R6_SMe6H_cyc4SE_0_1 charge=0 multiplicity=1 B3LYP_D3BJ_TZVPD_SMD_chcl3=-1357.809670155818, B3LYP_TZVP_CPCM_chcl3_freq=0.28283476, B3LYP_TZVPD_CPCM_chcl3=-1357.802812424513, M062X_TZVPD_SMD_chcl3=-1357.917127340692, M062X_TZVPD=-1357.890201521006, COSMORS=-0.004340667531843813, wB97MV_TZVPD_CPCM_chcl3=-1357.947397461488, wB97MV_TZVPD_SMD_chcl3=-1357.954455485276, M062X_TZVPD_CPCM_chcl3=-1357.910220234872
O -3.903595000000000 0.863383000000000 -0.185408000000000
C -2.860232000000000 1.697977000000000 -0.308145000000000
C -0.373970000000000 -3.006348000000000 -0.093931000000000
H -5.805639000000000 0.547724000000000 0.413186000000000
C -1.234569000000000 -0.036769000000000 0.296163000000000
C -1.584988000000000 1.284910000000000 -0.149011000000000
H -5.030731000000000 1.950820000000000 1.195673000000000
O -3.226948000000000 2.956988000000000 -0.653280000000000
C -5.159172000000000 1.407353000000000 0.258239000000000
H -5.589005000000000 2.065384000000000 -0.495327000000000
H -1.952194000000000 -5.050115000000000 -0.776391000000000
H -0.155437000000000 -2.195280000000000 1.884405000000000
C -0.003054000000000 -0.579652000000000 0.391642000000000
H -2.426411000000000 -3.586101000000000 -1.673616000000000
C 0.234513000000000 -2.010249000000000 0.883620000000000
C 1.329454000000000 -0.101937000000000 0.055676000000000
C 1.762266000000000 1.181118000000000 -0.572157000000000
H -3.399868000000000 -4.127301000000000 -0.274001000000000
S 1.694679000000000 2.520096000000000 0.703693000000000
C 2.217214000000000 -1.063501000000000 0.367993000000000
H 5.136223000000000 0.312300000000000 -0.678392000000000
H 1.054638000000000 1.480228000000000 -1.347089000000000
C 3.682047000000000 -1.050426000000000 0.158117000000000
O 1.659143000000000 -2.188110000000000 0.905768000000000
H 3.538028000000000 2.009388000000000 -1.489764000000000
C 4.144216000000000 0.360375000000000 -0.227163000000000
H 4.191074000000000 -1.400008000000000 1.060659000000000
H 3.925998000000000 -1.771127000000000 -0.631599000000000
C 3.161252000000000 1.031762000000000 -1.186060000000000
H 4.229624000000000 0.972011000000000 0.674009000000000
H 3.064579000000000 0.429392000000000 -2.095874000000000
C 1.665498000000000 3.987750000000000 -0.363214000000000
H 1.585707000000000 4.854566000000000 0.291679000000000
H 2.578608000000000 4.071529000000000 -0.951180000000000
H 0.800761000000000 3.960751000000000 -1.027502000000000
O -1.647357000000000 -3.249086000000000 0.229503000000000
O 0.192199000000000 -3.472238000000000 -1.051264000000000
C -2.400973000000000 -4.061622000000000 -0.694248000000000
H -2.067585000000000 -0.664658000000000 0.591315000000000
H -0.810639000000000 2.010008000000000 -0.350991000000000
H -2.448690000000000 3.530487000000000 -0.655485000000000
40
R6_SMe6_cyc4S_-1_1 charge=-1 multiplicity=1 wB97MV_TZVPD_SMD_chcl3=-1357.479395985273, B3LYP_D3BJ_TZVPD=-1357.255283970189, B3LYP_TZVP_CPCM_chcl3_freq=0.26928954, B3LYP_TZVPD_CPCM_chcl3=-1357.327438858921, M062X_TZVPD=-1357.361915774429, COSMORS=-0.010608636642363053, M062X_TZVPD_CPCM_chcl3=-1357.436897439425, M062X_TZVPD_SMD_chcl3=-1357.440109837871, wB97MV_TZVPD_CPCM_chcl3=-1357.475973968747, B3LYP_D3BJ_TZVPD_SMD_chcl3=-1357.33054790919, wB97MV_TZVPD=-1357.402299786665
O -4.851973000000000 0.311280000000000 0.485138000000000
C -3.625474000000000 0.873959000000000 0.113306000000000
C -0.522445000000000 -2.749289000000000 0.094073000000000
H -5.964553000000000 2.056420000000000 0.756233000000000
C -1.214570000000000 0.400946000000000 0.124206000000000
C -2.545845000000000 0.023720000000000 0.403814000000000
H -6.117202000000000 1.308412000000000 -0.842465000000000
O -3.617505000000000 2.001621000000000 -0.409436000000000
C -6.002757000000000 1.102868000000000 0.224463000000000
H -6.853520000000000 0.521067000000000 0.577651000000000
H -0.445563000000000 -4.934165000000000 -1.424937000000000
H -0.437435000000000 -1.757367000000000 1.963003000000000
C -0.064174000000000 -0.290069000000000 0.358063000000000
H 0.520618000000000 -4.075490000000000 -2.660306000000000
C 0.062301000000000 -1.666569000000000 0.994195000000000
C 1.304465000000000 0.107670000000000 0.095012000000000
C 1.801309000000000 1.321409000000000 -0.607859000000000
H -1.171765000000000 -3.593311000000000 -2.342126000000000
S 1.623524000000000 2.787356000000000 0.511966000000000
C 2.140199000000000 -0.823702000000000 0.576606000000000
H 5.158892000000000 0.423879000000000 -0.323085000000000
H 1.161744000000000 1.546944000000000 -1.464578000000000
C 3.617000000000000 -0.856954000000000 0.495981000000000
O 1.481420000000000 -1.884416000000000 1.174041000000000
H 3.673047000000000 2.059786000000000 -1.412822000000000
C 4.129609000000000 0.514209000000000 0.030065000000000
H 4.050583000000000 -1.119380000000000 1.466723000000000
H 3.931955000000000 -1.644762000000000 -0.202051000000000
C 3.250374000000000 1.113444000000000 -1.069947000000000
H 4.141297000000000 1.201038000000000 0.880276000000000
H 3.233799000000000 0.439072000000000 -1.933607000000000
C 1.644291000000000 4.133919000000000 -0.704846000000000
H 1.527003000000000 5.065452000000000 -0.152019000000000
H 2.586442000000000 4.164280000000000 -1.251869000000000
H 0.814121000000000 4.028259000000000 -1.403756000000000

O 0.269597000000000 -3.034743000000000 -0.945706000000000
O -1.607745000000000 -3.255185000000000 0.269877000000000
C -0.250803000000000 -3.974330000000000 -1.902204000000000
H -1.091818000000000 1.383147000000000 -0.324985000000000
H -2.768114000000000 -0.939746000000000 0.840393000000000
41
R6_SMe3_P1_0_1 charge=0 multiplicity=1 B3LYP_D3BJ_TZVPD=-1357.819970640248, B3LYP_D3BJ_TZVPD_SMD_chcl3=-1357.844220029545, B3LYP_TZVP_CPCM_chcl3_freq=0.280964, B3LYP_TZVPD_CPCM_chcl3=-1357.838379032208, M062X_TZVPD_SMD_chcl3=-1357.946483056126, M062X_TZVPD=-1357.919252507014, COSMORS=-0.00352160420094269 9, wB97MV_TZVPD_CPCM_chcl3=-1357.982128976597, wB97MV_TZVPD_SMD_chcl3=-1357.988139197083, wB97MV_TZVPD=-1357.963608598425, M062X_TZVPD_CPCM_chcl3=-1357.940856357793
O -4.530885000000000 0.563341000000000 0.816231000000000
C -3.652573000000000 -0.096770000000000 0.036087000000000
O -3.990001000000000 -0.929879000000000 -0.780132000000000
H -6.462106000000000 0.853535000000000 1.339327000000000
H -6.084363000000000 -0.826112000000000 0.857443000000000
C -2.275660000000000 0.336701000000000 0.318739000000000
C -1.249039000000000 -0.206880000000000 -0.335929000000000
C -5.918206000000000 0.227809000000000 0.637536000000000
H -6.230812000000000 0.438964000000000 -0.384501000000000
H 2.142508000000000 -0.584517000000000 -2.028910000000000
H -1.458859000000000 -0.968799000000000 -1.078323000000000
O 2.050185000000000 2.796964000000000 0.899767000000000
C 0.198199000000000 0.171188000000000 -0.232853000000000
O 2.904903000000000 0.748964000000000 1.242869000000000
C 3.383979000000000 3.341894000000000 0.891974000000000
H 3.962342000000000 2.902074000000000 0.080748000000000
H 3.260501000000000 4.409798000000000 0.738737000000000
H 3.877824000000000 3.145214000000000 1.842435000000000
C 0.516964000000000 1.007114000000000 1.025118000000000
C 1.055277000000000 -1.089277000000000 -0.307104000000000
C 1.950261000000000 1.470898000000000 1.068571000000000
H -2.154326000000000 1.089818000000000 1.083598000000000
C 1.960316000000000 -1.331893000000000 -1.266223000000000
C 0.867161000000000 -2.077291000000000 0.784055000000000
O 0.002610000000000 -1.939898000000000 1.636154000000000
C 2.791663000000000 -2.567623000000000 -1.369278000000000
H 1.381662000000000 -4.026083000000000 1.439061000000000
H 2.721717000000000 -2.881384000000000 1.363935000000000
C 2.222449000000000 -3.724744000000000 -0.558289000000000
H 2.900876000000000 -2.837514000000000 -2.422560000000000
H 3.803072000000000 -2.318454000000000 -1.019786000000000
C 1.830736000000000 -3.239710000000000 0.832803000000000
H 1.338234000000000 -4.120940000000000 -1.066073000000000
H 2.945917000000000 -4.538252000000000 -0.488009000000000
S 0.497300000000000 1.177599000000000 -1.780637000000000
H -0.140527000000000 1.869231000000000 1.081275000000000
H 0.335552000000000 0.381521000000000 1.898096000000000
C -0.497517000000000 2.657141000000000 -1.477291000000000
H -1.525308000000000 2.394761000000000 -1.229562000000000
H -0.061318000000000 3.278897000000000 -0.699027000000000
H -0.487596000000000 3.202674000000000 -2.420258000000000
38
R5_SMe6H_cyc4RTS_0_1 charge=0 multiplicity=1 B3LYP_D3BJ_TZVPD=-1318.426045569604, B3LYP_D3BJ_TZVPD_SMD_chcl3=-1318.451684392576, B3LYP_TZVP_CPCM_chcl3_freq=0.25262951, B3LYP_TZVPD_CPCM_chcl3=-1318.44627901832, M062X_TZVPD_SMD_chcl3=-1318.534957692661, M062X_TZVPD=-1318.508380428321, COSMORS=-0.00225869099763156 4, wB97MV_TZVPD_CPCM_chcl3=-1318.572309934322, wB97MV_TZVPD_SMD_chcl3=-1318.577963563806, wB97MV_TZVPD=-1318.551597179707, M062X_TZVPD_CPCM_chcl3=-1318.529461513181
C 1.397684000000000 1.406118000000000 -0.533901000000000
C 0.067144000000000 1.021483000000000 -0.317713000000000
C -0.471167000000000 -0.243931000000000 -0.505482000000000
C -1.898282000000000 -0.366555000000000 -0.521679000000000
C 0.140036000000000 -1.528111000000000 -0.700303000000000
C 1.528853000000000 -2.047611000000000 -0.877150000000000
C -0.704982000000000 -2.538531000000000 -0.268350000000000
O -1.928741000000000 -2.341009000000000 0.051072000000000
C 1.304165000000000 -3.578512000000000 -0.957977000000000
C 0.025557000000000 -3.851180000000000 -0.144414000000000
C 1.507495000000000 5.022465000000000 0.083991000000000
O 0.996996000000000 3.685091000000000 -0.101714000000000
O 3.101072000000000 3.075741000000000 -0.533351000000000
O -2.439663000000000 0.985628000000000 1.381323000000000
C -5.026217000000000 1.041403000000000 0.744822000000000
O -4.050000000000000 0.377839000000000 -0.077187000000000
C -2.780037000000000 0.413402000000000 0.364809000000000
C 1.828033000000000 2.702489000000000 -0.392596000000000
H 2.147843000000000 0.670831000000000 -0.781704000000000
H -0.629623000000000 1.805144000000000 -0.055047000000000
H -2.377415000000000 -0.770473000000000 -1.400374000000000
H 2.041653000000000 -1.673737000000000 -1.764346000000000
H 1.132258000000000 -3.847607000000000 -2.003012000000000
H 2.166317000000000 -4.138579000000000 -0.602042000000000
H -0.563759000000000 -4.682523000000000 -0.533257000000000
H 0.238073000000000 -4.063530000000000 0.907375000000000
H 2.221305000000000 5.044347000000000 0.905672000000000
H 0.635116000000000 5.622205000000000 0.323240000000000
H 1.975827000000000 5.380066000000000 -0.831347000000000
H -4.805587000000000 2.106430000000000 0.808963000000000
H -5.980675000000000 0.878382000000000 0.253049000000000
H -5.031013000000000 0.610346000000000 1.745387000000000
S 2.746253000000000 -1.595257000000000 0.475863000000000
C 1.711000000000000 -1.513902000000000 1.962475000000000
H 1.313883000000000 -2.490108000000000 2.233811000000000
H 2.361406000000000 -1.161160000000000 2.762398000000000
H 0.894551000000000 -0.806883000000000 1.828164000000000
H 3.658325000000000 2.304171000000000 -0.715158000000000
55
Sulf_SMe4_P2_0_1 charge=0 multiplicity=1 B3LYP_D3BJ_TZVPD=-2138.417265273422, B3LYP_D3BJ_TZVPD_SMD_chcl3=-2138.453028303796, B3LYP_TZVP_CPCM_chcl3_freq=0.37158375, B3LYP_TZVPD_CPCM_chcl3=-2138.443323600541, COSMORS=-0.00642790295080025 9, wB97MV_TZVPD_CPCM_chcl3=-2138.670074722869, wB97MV_TZVPD_SMD_chcl3=-2138.680171931742, wB97MV_TZVPD=-2138.643553767846
C 1.660066000000000 -3.074701000000000 0.104655000000000
C 2.816092000000000 -2.825668000000000 -0.620600000000000
C 2.744014000000000 -2.149069000000000 -1.833963000000000
C 1.515790000000000 -1.732305000000000 -2.337269000000000
C 0.351928000000000 -1.983590000000000 -1.625812000000000
C 0.438985000000000 -2.649527000000000 -0.408264000000000
O -0.833530000000000 -4.029139000000000 1.430300000000000
O -2.167869000000000 -2.898565000000000 -0.354173000000000
S -1.032171000000000 -2.899216000000000 0.544494000000000
C -0.946861000000000 3.073092000000000 -2.751526000000000
S 0.000041000000000 1.693608000000000 -2.053715000000000
C -3.653120000000000 0.363748000000000 1.485605000000000
C -4.965985000000000 0.685330000000000 1.168812000000000
C -5.350538000000000 0.819383000000000 -0.161477000000000
C -4.412383000000000 0.633084000000000 -1.170633000000000
C -3.096276000000000 0.323457000000000 -0.853505000000000
C -2.699140000000000 0.192189000000000 0.478536000000000
C 5.866758000000000 0.238786000000000 0.263642000000000
O 4.534854000000000 0.679080000000000 -0.053676000000000
O 3.751688000000000 -0.499405000000000 1.698374000000000
O 0.964762000000000 3.898366000000000 -0.177609000000000
C 0.539883000000000 4.197503000000000 2.440752000000000
O -0.036012000000000 3.119301000000000 1.675150000000000
C 0.273916000000000 3.080258000000000 0.375544000000000
C 3.552078000000000 0.226257000000000 0.745618000000000
C -0.431651000000000 1.895754000000000 -0.288429000000000
C -1.149702000000000 -1.428619000000000 1.609712000000000
C -1.302314000000000 -0.151500000000000 0.840661000000000
C -0.236646000000000 0.606689000000000 0.480696000000000
C 1.120043000000000 0.232926000000000 0.837068000000000
C 2.243058000000000 0.720485000000000 0.293865000000000
H 1.700701000000000 -3.587602000000000 1.055089000000000
H 3.772739000000000 -3.146404000000000 -0.231406000000000
H 3.650138000000000 -1.942141000000000 -2.388486000000000
H 1.463644000000000 -1.201936000000000 -3.278291000000000
H -0.608140000000000 -1.661045000000000 -1.999916000000000
H -0.817406000000000 3.019525000000000 -3.831691000000000
H -0.567354000000000 4.025034000000000 -2.386868000000000
H -2.004387000000000 2.965670000000000 -2.509657000000000
H -3.362115000000000 0.262271000000000 2.523834000000000
H -5.689224000000000 0.829133000000000 1.961367000000000
H -6.376009000000000 1.062222000000000 -0.408996000000000
H -4.705406000000000 0.722637000000000 -2.208961000000000
H -2.370862000000000 0.171508000000000 -1.640637000000000
H 6.510963000000000 0.700085000000000 -0.479142000000000
H 5.929213000000000 -0.847183000000000 0.202487000000000
H 6.144690000000000 0.562864000000000 1.265791000000000
H 1.626665000000000 4.140765000000000 2.403389000000000
H 0.207241000000000 5.156798000000000 2.047501000000000
H 0.183038000000000 4.054022000000000 3.456050000000000
H -1.491570000000000 2.149537000000000 -0.265793000000000
H -2.038376000000000 -1.646719000000000 2.202326000000000
H -0.280527000000000 -1.474138000000000 2.259749000000000
H 1.252834000000000 -0.515188000000000 1.605374000000000
H 2.246265000000000 1.437120000000000 -0.514563000000000
35
R6_0_1 charge=0 multiplicity=1 B3LYP_D3BJ_TZVPD=-919.141047519859, B3LYP_D3BJ_TZVPD_SMD_chcl3=-919.163801641633, B3LYP_TZVP_CPCM_chcl3_freq=0.23240944, B3LYP_TZVPD_CPCM_chcl3=-919.158670429435, M062X_TZVPD_SMD_chcl3=-919.231798597899, M062X_TZVPD=-919.208626869364, COSMORS=-0.00167057238707247 2, wB97MV_TZVPD_CPCM_chcl3=-919.279748002316, wB97MV_TZVPD_SMD_chcl3=-919.285089734584, wB97MV_TZVPD=-919.262076590838, M062X_TZVPD_CPCM_chcl3=-919.226678702938
C 2.216972000000000 0.286477000000000 0.006861000000000
C 1.159940000000000 1.102690000000000 -0.091445000000000
C -0.227825000000000 0.673847000000000 -0.159578000000000
C -1.182104000000000 1.625360000000000 -0.178405000000000
C -0.533877000000000 -0.779323000000000 -0.178108000000000
C -0.286821000000000 -1.532021000000000 -1.259014000000000
C -1.119218000000000 -1.378630000000000 1.046896000000000
O -1.206586000000000 -0.750391000000000 2.088308000000000
H -5.111641000000000 3.440536000000000 -0.264570000000000
C -1.616227000000000 -2.798139000000000 0.922208000000000
C 5.865310000000000 0.229393000000000 0.283119000000000
O 4.483664000000000 -0.166060000000000 0.197812000000000
O 3.890469000000000 2.001387000000000 0.039407000000000
O -3.168307000000000 0.279064000000000 -0.304223000000000
C -4.743760000000000 2.419074000000000 -0.288670000000000
O -3.309782000000000 2.524498000000000 -0.232269000000000
C -2.627751000000000 1.363934000000000 -0.243458000000000
C 3.586011000000000 0.827576000000000 0.079750000000000
H 2.110319000000000 -0.788280000000000 0.041145000000000
H 1.335123000000000 2.172982000000000 -0.110052000000000
H -0.893316000000000 2.667675000000000 -0.146712000000000
C -0.558989000000000 -2.997517000000000 -1.347274000000000
H -5.050128000000000 1.922904000000000 -1.208851000000000
H -5.114376000000000 1.857560000000000 0.568043000000000
C -0.712379000000000 -3.643806000000000 0.026465000000000
H -2.620532000000000 -2.737513000000000 0.485313000000000
H 6.161210000000000 0.762930000000000 -0.619345000000000
H 6.426758000000000 -0.694918000000000 0.381995000000000
H 6.021923000000000 0.867942000000000 1.151508000000000
H 0.118302000000000 -1.053733000000000 -2.145394000000000
H -1.715365000000000 -3.217985000000000 1.922976000000000
H -1.474798000000000 -3.136447000000000 -1.937826000000000
H 0.237850000000000 -3.478866000000000 -1.919922000000000
H 0.273245000000000 -3.732480000000000 0.492738000000000
H -1.114511000000000 -4.653468000000000 -0.068828000000000
40
R6_SMe6_cyc4RE_-1_1 charge=-1 multiplicity=1 wB97MV_TZVPD_SMD_chcl3=-1357.476548667475, B3LYP_TZVP_CPCM_chcl3_freq=0.26936602, B3LYP_TZVPD_CPCM_chcl3=-1357.325164857788, M062X_TZVPD=-1357.359776846616, COSMORS=-0.00977059351833970 9, M062X_TZVPD_CPCM_chcl3=-1357.434537782262, M062X_TZVPD_SMD_chcl3=-1357.437358886652, wB97MV_TZVPD_CPCM_chcl3=-1357.473516976096, B3LYP_D3BJ_TZVPD_SMD_chcl3=-1357.327888511368, wB97MV_TZVPD=-1357.400114008957
O -4.044352000000000 0.366287000000000 -0.749269000000000
C -3.212880000000000 1.448358000000000 -0.448260000000000
C 0.159869000000000 -2.776481000000000 0.519207000000000
H -5.796162000000000 0.988503000000000 0.199764000000000
C -1.304541000000000 -0.156894000000000 -0.603608000000000
C -1.845677000000000 1.134182000000000 -0.402752000000000
H -5.688477000000000 1.394078000000000 -1.521467000000000
O -3.733007000000000 2.562891000000000 -0.251606000000000
C -5.438306000000000 0.640270000000000 -0.771887000000000
H -5.923850000000000 -0.302056000000000 -1.023773000000000
H -1.467109000000000 -3.022007000000000 2.600521000000000
H -0.103740000000000 -2.556737000000000 -1.598149000000000
C -0.005277000000000 -0.573187000000000 -0.618275000000000
H -0.948180000000000 -4.645642000000000 2.086917000000000
C 0.407473000000000 -2.034393000000000 -0.787188000000000
C 1.265148000000000 0.130963000000000 -0.540441000000000
C 1.536033000000000 1.577043000000000 -0.298858000000000
H -2.582832000000000 -4.057367000000000 1.663320000000000
S 1.297950000000000 1.931347000000000 1.502836000000000
C 2.261882000000000 -0.731140000000000 -0.798871000000000
H 4.990568000000000 1.279216000000000 -0.676647000000000
H 0.791910000000000 2.190220000000000 -0.807674000000000
C 3.711325000000000 -0.452962000000000 -0.905616000000000
O 1.821301000000000 -2.016748000000000 -1.054182000000000
H 3.197818000000000 2.957086000000000 -0.431619000000000
C 4.001704000000000 0.948863000000000 -0.352610000000000
H 4.285239000000000 -1.209359000000000 -0.360724000000000
H 4.025607000000000 -0.526551000000000 -1.955693000000000
C 2.939683000000000 1.958443000000000 -0.788985000000000
H 4.017231000000000 0.907053000000000 0.739458000000000
H 2.910951000000000 2.008288000000000 -1.883467000000000
C 0.971368000000000 3.715837000000000 1.443397000000000
H 1.819198000000000 4.262097000000000 1.030390000000000
H 0.076680000000000 3.918523000000000 0.854014000000000
H 0.804265000000000 4.044758000000000 2.468534000000000
O -1.101949000000000 -3.230917000000000 0.561766000000000
O 0.950385000000000 -2.895723000000000 1.425908000000000
C -1.545228000000000 -3.775210000000000 1.817226000000000
H -2.038091000000000 -0.941696000000000 -0.764365000000000
H -1.204454000000000 1.968159000000000 -0.159239000000000
38
R5_SMe4_P1SR_0_1 charge=0 multiplicity=1 B3LYP_D3BJ_TZVPD=-1318.516296384716, CCSDT_SMD_chcl3=-1316.779145399426, CCSDT=-1316.753876428053, B3LYP_D3BJ_TZVPD_SMD_chcl3=-1318.542985872428, B3LYP_TZVP_CPCM_chcl3_freq=0.25188755, B3LYP_TZVPD_CPCM_chcl3=-1318.537176152114, M062X_TZVPD_SMD_chcl3=-1318.63832738505, M062X_TZVPD=-1318.611405639313, COSMORS=7.55685801726382e-05, wB97MV_TZVPD_CPCM_chcl3=-1318.680234240446, wB97MV_TZVPD_SMD_chcl3=-1318.686214752738, wB97MV_TZVPD=-1318.65935497633, M062X_TZVPD_CPCM_chcl3=-1318.632580351821
C -2.289024000000000 0.183983000000000 -0.251490000000000
C -1.179099000000000 0.142422000000000 0.480483000000000
C 0.196059000000000 -0.066283000000000 -0.084187000000000
C 1.196265000000000 0.926384000000000 0.515418000000000
C 0.614896000000000 -1.491625000000000 0.163321000000000
C 1.258457000000000 -2.026262000000000 1.209724000000000
C 0.305760000000000 -2.555593000000000 -0.815278000000000
O -0.269864000000000 -2.402459000000000 -1.876044000000000
C 1.471493000000000 -3.505671000000000 1.087164000000000
C 0.839583000000000 -3.867742000000000 -0.264192000000000
C -5.926735000000000 0.551233000000000 -0.065586000000000
O -4.582225000000000 0.390946000000000 -0.552880000000000
O -3.829813000000000 0.435373000000000 1.568534000000000
O 2.785596000000000 0.531212000000000 -1.254737000000000
C 4.891647000000000 0.907804000000000 0.339857000000000
O 3.534173000000000 0.949623000000000 0.824784000000000
C 2.573796000000000 0.766759000000000 -0.086982000000000
C -3.612277000000000 0.347287000000000 0.377867000000000
H -2.260595000000000 0.101267000000000 -1.330203000000000
H -1.254068000000000 0.221695000000000 1.560685000000000
H 1.263986000000000 0.798704000000000 1.595180000000000
H 1.608230000000000 -1.475394000000000 2.073790000000000
H 2.536972000000000 -3.746190000000000 1.131061000000000
H 1.002912000000000 -4.030008000000000 1.924395000000000
H 1.557227000000000 -4.286674000000000 -0.971508000000000
H 0.018614000000000 -4.581030000000000 -0.175370000000000
H -6.195956000000000 -0.282281000000000 0.582098000000000
H -6.557149000000000 0.566243000000000 -0.949867000000000
H -6.019821000000000 1.485415000000000 0.486908000000000
H 5.048961000000000 1.691993000000000 -0.399254000000000
H 5.102216000000000 -0.063590000000000 -0.104798000000000
H 5.515892000000000 1.073648000000000 1.212601000000000
S 0.692298000000000 2.681506000000000 0.347932000000000
C 0.408810000000000 2.840309000000000 -1.436506000000000
H -0.461089000000000 2.267507000000000 -1.752645000000000
H 0.219211000000000 3.899518000000000 -1.605808000000000
H 1.288850000000000 2.536973000000000 -1.999601000000000
H 0.161438000000000 0.067095000000000 -1.165573000000000
44
R5_SMe4_P2_SMe1_P2RS charge=0 multiplicity=1 B3LYP_D3BJ_TZVPD=-1757.195894969241, B3LYP_D3BJ_TZVPD_SMD_chcl3=-1757.222644609967, B3LYP_TZVP_CPCM_chcl3_freq=0.3015082, B3LYP_TZVPD_CPCM_chcl3=-1757.215601389579, M062X_TZVPD_SMD_chcl3=-1757.347666458156, M062X_TZVPD=-1757.320192543088, COSMORS=-0.00422352189016328 6, wB97MV_TZVPD_CPCM_chcl3=-1757.378087433916, wB97MV_TZVPD_SMD_chcl3=-1757.385277685368, wB97MV_TZVPD=-1757.357999290921, M062X_TZVPD_CPCM_chcl3=-1757.340703198086
C 3.475500000000000 -1.339955000000000 -2.289972000000000
C -4.922731000000000 -0.666892000000000 -0.046172000000000
C 3.681344000000000 1.322896000000000 2.039875000000000
C -2.985623000000000 3.276035000000000 0.020471000000000
C -0.327518000000000 -3.665243000000000 1.324317000000000
C -0.382352000000000 -2.213295000000000 1.830445000000000
C 0.271990000000000 -3.547000000000000 -0.081302000000000
C 0.652687000000000 0.669551000000000 1.098569000000000
C -0.806779000000000 -1.419453000000000 0.585573000000000
C -0.629205000000000 0.073841000000000 0.568969000000000
C -0.134989000000000 -2.173180000000000 -0.574196000000000
C 1.708194000000000 0.946566000000000 0.019778000000000
C -1.567691000000000 0.871935000000000 0.028062000000000
C 2.411760000000000 -0.300905000000000 -0.460071000000000
C -2.849402000000000 0.351567000000000 -0.545499000000000
O 0.014684000000000 -1.741593000000000 -1.692660000000000
O 2.652969000000000 -1.257453000000000 0.245365000000000
O -3.146278000000000 0.402918000000000 -1.715267000000000
O 2.769641000000000 -0.210554000000000 -1.739995000000000
O -3.659746000000000 -0.133187000000000 0.405685000000000
S 3.000381000000000 2.133195000000000 0.565276000000000
S -1.312497000000000 2.604414000000000 -0.211523000000000
H 3.684902000000000 -1.075091000000000 -3.321890000000000
H 2.847138000000000 -2.227501000000000 -2.242712000000000
H 4.400925000000000 -1.509345000000000 -1.741444000000000
H -4.754381000000000 -1.501049000000000 -0.725727000000000
H -5.497191000000000 0.107734000000000 -0.551214000000000
H -5.432780000000000 -1.000149000000000 0.852440000000000
H 2.940892000000000 1.255080000000000 2.834508000000000
H 4.500243000000000 1.961469000000000 2.369135000000000
H 4.069610000000000 0.334997000000000 1.800142000000000
H -3.368150000000000 3.023451000000000 1.007211000000000
H -3.661769000000000 2.925622000000000 -0.757156000000000
H -2.876977000000000 4.356343000000000 -0.061619000000000
H -1.338007000000000 -4.077058000000000 1.267998000000000
H 0.258072000000000 -4.309443000000000 1.978748000000000
H 0.609315000000000 -1.907457000000000 2.164602000000000
H -1.076942000000000 -2.076816000000000 2.658561000000000
H -0.036078000000000 -4.318614000000000 -0.787082000000000
H 1.365229000000000 -3.546772000000000 -0.045015000000000
H 1.086935000000000 0.018647000000000 1.852468000000000
H 0.418886000000000 1.622825000000000 1.573391000000000
H -1.865156000000000 -1.652851000000000 0.422817000000000
H 1.266945000000000 1.450502000000000 -0.838795000000000

38
R5_SMe1_P2_0_1 charge=0 multiplicity=1 B3LYP_D3BJ_TZVPD=-1318.518730292551, CCSDT_SMD_chcl3=-1316.778086636573, CCSDT=-1316.754572529095, B3LYP_D3BJ_TZVPD_SMD_chcl3=-1318.543324342679, B3LYP_TZVP_CPCM_chcl3_freq=0.25158634, B3LYP_TZVPD_CPCM_chcl3=-1318.537550626045, M062X_TZVPD_SMD_chcl3=-1318.63861429827, M062X_TZVPD=-1318.613537231184, COSMORS=-0.0011667552925642567, wB97MV_TZVPD_CPCM_chcl3=-1318.680031127084, wB97MV_TZVPD_SMD_chcl3=-1318.686004104758, wB97MV_TZVPD=-1318.661049477402, M062X_TZVPD_CPCM_chcl3=-1318.632852409153
O 3.893162000000000 -0.846551000000000 -0.709734000000000
C 3.100480000000000 0.229914000000000 -0.712667000000000
H 5.751274000000000 0.075553000000000 -0.909571000000000
H 5.651121000000000 -1.672244000000000 -1.275735000000000
C 0.662618000000000 0.684038000000000 -0.465075000000000
C 1.850560000000000 -0.004896000000000 0.107899000000000
O -2.081789000000000 -2.040218000000000 -1.879855000000000
O 3.369165000000000 1.266402000000000 -1.276791000000000
C 5.161566000000000 -0.708916000000000 -1.381895000000000
H 5.007044000000000 -0.470051000000000 -2.432909000000000
H 1.686277000000000 -1.074740000000000 0.217252000000000
S 2.252894000000000 0.531759000000000 1.839117000000000
C -0.583303000000000 0.202928000000000 -0.503995000000000
H 0.856391000000000 1.656756000000000 -0.899815000000000
H -5.178209000000000 1.764243000000000 0.801138000000000
C -0.973753000000000 -1.101330000000000 0.051709000000000
H 2.943072000000000 2.680092000000000 0.947822000000000
H 1.225556000000000 2.694313000000000 1.445530000000000
C -1.682618000000000 1.002779000000000 -1.151316000000000
C -0.753995000000000 -1.570140000000000 1.291336000000000
H -3.036766000000000 -3.486464000000000 0.282099000000000
C -1.304789000000000 -2.941549000000000 1.527501000000000
H -0.240493000000000 -1.014491000000000 2.065192000000000
C -1.962230000000000 -3.315988000000000 0.194088000000000
H -0.506462000000000 -3.632422000000000 1.812919000000000
H -2.012987000000000 -2.931330000000000 2.360353000000000
C -1.724940000000000 -2.122978000000000 -0.718866000000000
H -1.530970000000000 -4.207698000000000 -0.263548000000000
C 2.224512000000000 2.338669000000000 1.690295000000000
H 2.510800000000000 2.716450000000000 2.671146000000000
H -5.655493000000000 2.747496000000000 -0.613720000000000
H -4.466149000000000 3.378289000000000 0.563493000000000
C -2.694681000000000 1.527563000000000 -0.155049000000000
O -2.523492000000000 1.620228000000000 1.038194000000000
C -4.844186000000000 2.489374000000000 0.060413000000000
O -3.818996000000000 1.913509000000000 -0.773414000000000
H -2.208949000000000 0.404871000000000 -1.895877000000000
H -1.269553000000000 1.871526000000000 -1.669715000000000
54
Sulf_SMe4_-1_1 charge=-1 multiplicity=1 wB97MV_TZVPD_SMD_chcl3=-2138.183177089811, B3LYP_D3BJ_TZVPD=-2137.878544052784, B3LYP_TZVP_CPCM_chcl3_freq=0.35669405, B3LYP_TZVPD_CPCM_chcl3=-2137.94860245669, M062X_TZVPD=-2138.030912250679, COSMORS=-0.016124967698807502, M062X_TZVPD_CPCM_chcl3=-2138.103609229716, M062X_TZVPD_SMD_chcl3=-2138.110272205282, wB97MV_TZVPD_CPCM_chcl3=-2138.176420956316, B3LYP_D3BJ_TZVPD_SMD_chcl3=-2137.95477545365, wB97MV_TZVPD=-2138.105214190689
C -3.140865000000000 0.391084000000000 -1.463772000000000
C -3.657329000000000 -0.454270000000000 -2.433440000000000
C -4.288108000000000 -1.639651000000000 -2.059994000000000
C -4.403195000000000 -1.974790000000000 -0.716715000000000
C -3.884518000000000 -1.132248000000000 0.260118000000000
C -3.256891000000000 0.043142000000000 -0.122223000000000
O -2.865095000000000 0.531231000000000 2.403088000000000
O -3.154170000000000 2.462281000000000 0.880593000000000
S -2.575750000000000 1.141436000000000 1.111440000000000
C 2.865340000000000 2.028185000000000 2.271622000000000
S 2.019537000000000 3.105986000000000 1.086088000000000
C -0.588551000000000 -1.930770000000000 -0.472423000000000
C -1.020764000000000 -3.244594000000000 -0.370904000000000
C -1.304318000000000 -3.790255000000000 0.877227000000000
C -1.155250000000000 -3.011658000000000 2.018966000000000
C -0.734108000000000 -1.691058000000000 1.913869000000000
C -0.447523000000000 -1.139269000000000 0.668242000000000
C 6.584368000000000 -2.533580000000000 -0.642380000000000
O 5.697683000000000 -1.413791000000000 -0.641942000000000
O 4.056601000000000 -2.808042000000000 0.050005000000000
O 0.564148000000000 3.755410000000000 -1.319513000000000
C -0.302107000000000 2.296019000000000 -3.357339000000000
O 0.496904000000000 1.738592000000000 -2.299095000000000
C 0.860292000000000 2.582604000000000 -1.326515000000000
C 4.399903000000000 -1.670904000000000 -0.264449000000000
C 1.753691000000000 1.897751000000000 -0.305032000000000
C -0.896807000000000 1.292587000000000 0.776156000000000
C -0.000666000000000 0.276111000000000 0.532999000000000
C 1.327999000000000 0.517142000000000 0.094298000000000
C 2.270218000000000 -0.519993000000000 0.055032000000000
C 3.600185000000000 -0.483976000000000 -0.299383000000000
H -2.640439000000000 1.308109000000000 -1.741160000000000
H -3.565521000000000 -0.192488000000000 -3.479816000000000
H -4.684294000000000 -2.302395000000000 -2.818975000000000
H -4.884174000000000 -2.899767000000000 -0.425792000000000
H -3.943856000000000 -1.388665000000000 1.307353000000000
H 2.229705000000000 1.191661000000000 2.555902000000000
H 3.063203000000000 2.646483000000000 3.146800000000000
H 3.805943000000000 1.653923000000000 1.871532000000000
H -0.367779000000000 -1.502571000000000 -1.441051000000000
H -1.140164000000000 -3.842434000000000 -1.265627000000000
H -1.640185000000000 -4.816528000000000 0.958545000000000
H -1.370352000000000 -3.430369000000000 2.994202000000000
H -0.632966000000000 -1.079880000000000 2.799524000000000
H 7.551066000000000 -2.145670000000000 -0.956611000000000
H 6.663621000000000 -2.971668000000000 0.353530000000000
H 6.246095000000000 -3.301644000000000 -1.339434000000000
H 0.276828000000000 3.025082000000000 -3.923635000000000
H -0.577882000000000 1.454868000000000 -3.986623000000000
H -1.189611000000000 2.776561000000000 -2.948920000000000
H 2.715490000000000 1.822139000000000 -0.813757000000000
H -0.620212000000000 2.333974000000000 0.727534000000000
H 1.914019000000000 -1.496810000000000 0.359127000000000
H 4.102347000000000 0.424697000000000 -0.600288000000000
41
R6_SMe4_P1SR_0_1 charge=0 multiplicity=1 B3LYP_D3BJ_TZVPD=-1357.82221167991, B3LYP_D3BJ_TZVPD_SMD_chcl3=-1357.84933327117, B3LYP_TZVP_CPCM_chcl3_freq=0.28057679, B3LYP_TZVPD_CPCM_chcl3=-1357.842691863497, M062X_TZVPD_SMD_chcl3=-1357.94827052065, M062X_TZVPD=-1357.920880454274, COSMORS=-0.001409460791516024, wB97MV_TZVPD_CPCM_chcl3=-1357.985852485981, wB97MV_TZVPD_SMD_chcl3=-1357.992671298319, wB97MV_TZVPD=-1357.965368081376, M062X_TZVPD_CPCM_chcl3=-1357.941683538185
O 4.664159000000000 -0.117482000000000 0.676755000000000
C 3.719175000000000 -0.056966000000000 -0.279459000000000
O 3.975769000000000 -0.057119000000000 -1.465885000000000
H 6.180548000000000 -1.073140000000000 -0.387690000000000
H 6.633024000000000 -0.230062000000000 1.122819000000000
C 2.373514000000000 0.010226000000000 0.317297000000000
C 1.285776000000000 0.103674000000000 -0.443662000000000
C 6.027670000000000 -0.183586000000000 0.222217000000000
H 6.275952000000000 0.703020000000000 -0.360055000000000
H 1.398021000000000 0.122043000000000 -1.523796000000000
H 2.308337000000000 -0.018212000000000 1.397093000000000
O -3.235243000000000 1.570968000000000 -0.994752000000000
C -0.116931000000000 0.127729000000000 0.085441000000000
O -2.640836000000000 1.122843000000000 1.127955000000000
C -4.605096000000000 1.728428000000000 -0.573065000000000
H -4.687023000000000 2.554564000000000 0.131840000000000
H -5.164310000000000 1.941752000000000 -1.478980000000000
H -4.962919000000000 0.811685000000000 -0.107016000000000
C -0.938103000000000 1.236974000000000 -0.576765000000000
C -0.742963000000000 -1.244380000000000 -0.113741000000000
C -2.348289000000000 1.288649000000000 -0.034358000000000
H -1.362228000000000 -0.952921000000000 -2.097514000000000
C -1.331997000000000 -1.635003000000000 -1.254134000000000
H 0.784589000000000 2.387838000000000 1.748262000000000
O -0.070705000000000 -1.869036000000000 2.063037000000000
C -1.965438000000000 -2.970977000000000 -1.468746000000000
H -1.309898000000000 -4.053457000000000 1.763186000000000
H -0.253588000000000 -4.164705000000000 0.359126000000000
C -2.292519000000000 -3.668861000000000 -0.152700000000000
H -2.860941000000000 -2.849039000000000 -2.083023000000000
H -1.277351000000000 -3.585594000000000 -2.065210000000000
C -1.095867000000000 -3.608956000000000 0.791133000000000
H -3.145405000000000 -3.168553000000000 0.315219000000000
H -2.583697000000000 -4.705137000000000 -0.330235000000000
C -0.604399000000000 -2.197157000000000 1.014859000000000
S -0.194322000000000 2.905794000000000 -0.417743000000000
H -0.979246000000000 1.103891000000000 -1.656752000000000
C 0.029562000000000 3.076344000000000 1.373664000000000
H 0.374004000000000 4.098827000000000 1.524287000000000
H -0.910989000000000 2.932194000000000 1.901159000000000
H -0.089580000000000 0.300727000000000 1.158933000000000
40
R6_SMe6_cyc2S_-1_1 charge=-1 multiplicity=1 wB97MV_TZVPD_SMD_chcl3=-1357.481042790278, B3LYP_D3BJ_TZVPD=-1357.261294790028, B3LYP_TZVP_CPCM_chcl3_freq=0.26782615, B3LYP_TZVPD_CPCM_chcl3=-1357.330724944573, M062X_TZVPD=-1357.363488305058, COSMORS=-0.012534472107715853, M062X_TZVPD_CPCM_chcl3=-1357.435495402298, M062X_TZVPD_SMD_chcl3=-1357.437997562939, wB97MV_TZVPD_CPCM_chcl3=-1357.478268231653, B3LYP_D3BJ_TZVPD_SMD_chcl3=-1357.333233673062, wB97MV_TZVPD=-1357.407678015305
O -5.019927000000000 -0.059932000000000 -0.655546000000000
C -3.974038000000000 -0.411535000000000 0.206927000000000
C 0.726561000000000 2.483469000000000 -0.414506000000000
H -7.007969000000000 0.140699000000000 -0.913433000000000
C -1.521477000000000 -0.574277000000000 0.334551000000000
C -2.721285000000000 -0.258088000000000 -0.389286000000000
H -6.561599000000000 -1.171905000000000 0.207743000000000
O -4.251217000000000 -0.789212000000000 1.359472000000000
C -6.330065000000000 -0.153765000000000 -0.112813000000000
H -6.461363000000000 0.514438000000000 0.741548000000000
H 2.299263000000000 4.614710000000000 0.044494000000000
H -1.222190000000000 1.993599000000000 0.297161000000000
C -0.249022000000000 0.177355000000000 -0.017280000000000
H 0.880207000000000 5.697721000000000 0.072740000000000
C -0.291737000000000 1.540035000000000 -0.018208000000000
C 0.787520000000000 -0.779775000000000 -0.249609000000000
C 2.247177000000000 -0.581642000000000 -0.491142000000000
H 1.354548000000000 4.870377000000000 -1.437522000000000
S 3.033550000000000 -0.057455000000000 1.106846000000000
C 0.240485000000000 -2.033961000000000 -0.143935000000000
H 3.000961000000000 -3.978290000000000 -0.704837000000000
H 2.406031000000000 0.265166000000000 -1.151423000000000
C 0.976225000000000 -3.313844000000000 -0.352652000000000
O -1.039083000000000 -2.090147000000000 0.136416000000000
H 3.982880000000000 -1.732991000000000 -1.062886000000000
C 2.491042000000000 -3.102550000000000 -0.298950000000000
H 0.643671000000000 -4.044291000000000 0.388450000000000
H 0.678047000000000 -3.708765000000000 -1.331098000000000
C 2.897228000000000 -1.845037000000000 -1.063174000000000
H 2.806632000000000 -3.001363000000000 0.742651000000000
H 2.586867000000000 -1.938527000000000 -2.110507000000000
C 4.498449000000000 0.804533000000000 0.470951000000000
H 5.032533000000000 1.209092000000000 1.330198000000000
H 5.158588000000000 0.124293000000000 -0.067157000000000
H 4.190985000000000 1.620886000000000 -0.181604000000000
O 0.408522000000000 3.736431000000000 0.034635000000000
O 1.727095000000000 2.303654000000000 -1.093985000000000
C 1.297986000000000 4.787740000000000 -0.351488000000000
H -1.690205000000000 -0.591053000000000 1.412544000000000
H -2.656524000000000 0.071741000000000 -1.416757000000000
37
R5_SMe6_cyc4R_-1_1 charge=-1 multiplicity=1 wB97MV_TZVPD_SMD_chcl3=-1318.154678356329, B3LYP_D3BJ_TZVPD=-1317.933727900387, B3LYP_TZVP_CPCM_chcl3_freq=0.241475, B3LYP_TZVPD_CPCM_chcl3=-1318.00456063128, M062X_TZVPD=-1318.035835749396, COSMORS=-0.009585177991070908, M062X_TZVPD_CPCM_chcl3=-1318.109395348896, M062X_TZVPD_SMD_chcl3=-1318.111519941251, wB97MV_TZVPD_CPCM_chcl3=-1318.152346816654, B3LYP_D3BJ_TZVPD_SMD_chcl3=-1318.006602601707, wB97MV_TZVPD=-1318.080010982333
C -2.158263000000000 0.879006000000000 -0.386717000000000
C -1.376682000000000 -0.265863000000000 -0.654148000000000
C -0.020877000000000 -0.354031000000000 -0.777703000000000
C 0.737438000000000 -1.657937000000000 -1.076780000000000
C 1.033146000000000 0.634237000000000 -0.747032000000000
C 1.255289000000000 2.056662000000000 -0.368569000000000
C 2.187948000000000 0.063412000000000 -1.102367000000000
O 2.106383000000000 -1.274496000000000 -1.392476000000000
C 2.717314000000000 2.318736000000000 -0.856576000000000
C 3.385337000000000 0.934031000000000 -1.101633000000000
C -5.582780000000000 -0.365597000000000 -0.456778000000000
O -4.163818000000000 -0.342339000000000 -0.525244000000000
O -4.285831000000000 1.883325000000000 -0.079685000000000
O -0.171970000000000 -3.390325000000000 0.317693000000000
C 1.567137000000000 -2.977656000000000 2.297366000000000
O 1.642538000000000 -2.275458000000000 1.043904000000000
C 0.691282000000000 -2.556896000000000 0.147511000000000
C -3.560406000000000 0.898729000000000 -0.310186000000000
H -1.678180000000000 1.833686000000000 -0.216179000000000
H -1.925318000000000 -1.194952000000000 -0.779475000000000
H 0.335445000000000 -2.230016000000000 -1.914461000000000
H 0.552813000000000 2.755795000000000 -0.823371000000000
H 2.673743000000000 2.870039000000000 -1.796937000000000
H 3.274232000000000 2.927416000000000 -0.145489000000000
H 3.939790000000000 0.910470000000000 -2.044461000000000
H 4.092652000000000 0.662248000000000 -0.308950000000000
H -5.943482000000000 -0.057354000000000 0.527137000000000
H -5.874063000000000 -1.398120000000000 -0.646521000000000
H -6.032567000000000 0.286978000000000 -1.208399000000000
H 0.622735000000000 -2.759190000000000 2.794440000000000
H 1.655850000000000 -4.051441000000000 2.137233000000000
H 2.401950000000000 -2.609728000000000 2.886912000000000
S 0.992351000000000 2.379368000000000 1.437476000000000
C 2.120658000000000 1.169176000000000 2.179495000000000
H 1.952915000000000 1.205979000000000 3.255251000000000
H 1.899016000000000 0.168715000000000 1.813077000000000
H 3.161955000000000 1.416290000000000 1.973114000000000
44
R5_SMe4_P3_SMe6SRS_P2 charge=0 multiplicity=1 B3LYP_D3BJ_TZVPD=-1757.196790751309, B3LYP_D3BJ_TZVPD_SMD_chcl3=-1757.221923777917, B3LYP_TZVP_CPCM_chcl3_freq=0.30151204, B3LYP_TZVPD_CPCM_chcl3=-1757.216271430372, M062X_TZVPD_SMD_chcl3=-1757.345794057338, M062X_TZVPD=-1757.320586663955, COSMORS=-0.0033890803053889963, wB97MV_TZVPD_CPCM_chcl3=-1757.378320660733, wB97MV_TZVPD_SMD_chcl3=-1757.384135704504, wB97MV_TZVPD=-1757.35893662797, M062X_TZVPD_CPCM_chcl3=-1757.340230709538
C -5.623777000000000 -1.200907000000000 0.084504000000000
C 1.146449000000000 -4.358041000000000 1.090156000000000
C 2.801031000000000 1.912889000000000 2.255497000000000
C 2.857232000000000 -0.939932000000000 -2.406752000000000
C -0.944592000000000 -0.144680000000000 0.144680000000000
C -1.046092000000000 3.287296000000000 -0.963053000000000
C -0.794534000000000 3.084452000000000 0.532240000000000
C -2.075807000000000 -0.482488000000000 -0.469077000000000
C 0.358008000000000 0.071296000000000 -0.565454000000000
C -0.209482000000000 2.231481000000000 -1.655087000000000
C 0.625638000000000 2.491078000000000 0.589435000000000
C -3.331955000000000 -0.650605000000000 0.286256000000000
C 0.742763000000000 1.572067000000000 -0.650445000000000
C 1.446910000000000 -0.813098000000000 0.071044000000000
C 1.082948000000000 -2.279364000000000 -0.028152000000000
O -0.281595000000000 1.928223000000000 -2.823285000000000
O -3.470102000000000 -0.490829000000000 1.481109000000000
O 0.605859000000000 -2.803225000000000 -1.009320000000000
O -4.337536000000000 -1.006517000000000 -0.531689000000000
O 1.380644000000000 -2.935051000000000 1.098080000000000
S 0.988653000000000 1.831763000000000 2.246120000000000
S 3.125383000000000 -0.627501000000000 -0.639795000000000
H -5.957257000000000 -0.279782000000000 0.560865000000000
H -5.570181000000000 -1.995892000000000 0.827234000000000
H -6.294530000000000 -1.477768000000000 -0.723498000000000
H 0.094121000000000 -4.564337000000000 0.901512000000000
H 1.433879000000000 -4.703911000000000 2.078398000000000
H 1.757312000000000 -4.833263000000000 0.323876000000000
H 3.135099000000000 2.941836000000000 2.124979000000000
H 3.230375000000000 1.284586000000000 1.476803000000000
H 3.125111000000000 1.549314000000000 3.229589000000000
H 3.847735000000000 -0.902295000000000 -2.858581000000000
H 2.230445000000000 -0.171546000000000 -2.855658000000000
H 2.421654000000000 -1.923630000000000 -2.566452000000000
H -0.953186000000000 -0.008902000000000 1.220190000000000
H -0.680917000000000 4.263440000000000 -1.299444000000000
H -2.089179000000000 3.205976000000000 -1.269157000000000
H -1.519623000000000 2.382629000000000 0.947055000000000
H -0.859035000000000 4.006117000000000 1.107529000000000
H -2.117397000000000 -0.642519000000000 -1.538924000000000
H 0.234326000000000 -0.257277000000000 -1.597785000000000
H 1.334152000000000 3.308621000000000 0.440079000000000
H 1.752713000000000 1.609098000000000 -1.060454000000000
H 1.582206000000000 -0.554402000000000 1.118494000000000
44
R5_SMe4_P2_SMe1RS charge=0 multiplicity=1 B3LYP_D3BJ_TZVPD=-1757.19361847658, B3LYP_D3BJ_TZVPD_SMD_chcl3=-1757.221436090199, B3LYP_TZVP_CPCM_chcl3_freq=0.30043338, B3LYP_TZVPD_CPCM_chcl3=-1757.214927131278, M062X_TZVPD_SMD_chcl3=-1757.347642778735, COSMORS=-0.0025569653787136533, wB97MV_TZVPD_CPCM_chcl3=-1757.37854588376, wB97MV_TZVPD_SMD_chcl3=-1757.385198515734, wB97MV_TZVPD=-1757.35713219348, M062X_TZVPD_CPCM_chcl3=-1757.341228748857
C 5.132279000000000 1.269964000000000 -0.373134000000000
C -3.357244000000000 -3.086305000000000 0.064890000000000
C 1.091737000000000 -2.888933000000000 1.472982000000000
C -0.741425000000000 2.297757000000000 -2.319417000000000
C -1.961823000000000 1.391613000000000 2.938220000000000
C -2.017797000000000 0.318303000000000 1.838542000000000
C -1.233157000000000 2.562682000000000 2.272010000000000
C 0.740350000000000 -0.528495000000000 -0.688429000000000
C -0.644734000000000 0.423454000000000 1.158867000000000
C -0.444623000000000 -0.119314000000000 -0.228821000000000
C -0.279538000000000 1.914778000000000 1.288765000000000
C 1.999909000000000 -0.643573000000000 0.095822000000000
C -1.601280000000000 -0.081032000000000 -1.192573000000000
C 3.176896000000000 -0.025461000000000 -0.634429000000000
C -2.776986000000000 -0.971944000000000 -0.811818000000000
O 0.653364000000000 2.450175000000000 0.737874000000000
O 3.385910000000000 -0.141984000000000 -1.818719000000000
O -3.941905000000000 -0.699214000000000 -0.970861000000000
O 3.961392000000000 0.654404000000000 0.203848000000000
O -2.343752000000000 -2.133432000000000 -0.314318000000000
S 2.433893000000000 -2.430637000000000 0.344260000000000
S -2.208516000000000 1.623847000000000 -1.496262000000000
H 5.630105000000000 1.771430000000000 0.451080000000000
H 4.838445000000000 1.986183000000000 -1.138649000000000
H 5.778253000000000 0.509012000000000 -0.808254000000000
H -3.956465000000000 -3.363514000000000 -0.800750000000000
H -3.998181000000000 -2.660765000000000 0.835531000000000
H -2.815479000000000 -3.945725000000000 0.447862000000000
H 0.123419000000000 -2.695578000000000 1.013342000000000
H 1.177254000000000 -2.352586000000000 2.417339000000000
H 1.198284000000000 -3.957709000000000 1.654052000000000
H -0.988883000000000 3.326547000000000 -2.577816000000000
H -0.524828000000000 1.739793000000000 -3.229813000000000
H 0.118950000000000 2.287733000000000 -1.653835000000000
H -1.380958000000000 1.024768000000000 3.788069000000000
H -2.951791000000000 1.661746000000000 3.302802000000000

H -2.816779000000000 0.572255000000000 1.142791000000000
H -2.210858000000000 -0.679905000000000 2.228209000000000
H -0.700347000000000 3.230547000000000 2.948464000000000
H -1.928250000000000 3.170450000000000 1.683986000000000
H 0.822908000000000 -0.853717000000000 -1.718622000000000
H 0.073237000000000 -0.059595000000000 1.830347000000000
H 1.937094000000000 -0.190734000000000 1.079716000000000
H -1.256802000000000 -0.467888000000000 -2.155173000000000
38
R5_SMe6_cyc4P2_0_1 charge=0 multiplicity=1 B3LYP_D3BJ_TZVPD=-1318.52128470987, B3LYP_D3BJ_TZVPD_SMD_chcl3=-1318.5436602273, B3LYP_TZVP_CPCM_chcl3_freq=0.25564465, B3LYP_TZVPD_CPCM_chcl3=-1318.538341297598, M062X_TZVPD_SMD_chcl3=-1318.646919148867, M062X_TZVPD=-1318.624505288054, COSMORS=-0.004269672587797187, wB97MV_TZVPD_CPCM_chcl3=-1318.687243382561, wB97MV_TZVPD_SMD_chcl3=-1318.692732537732, wB97MV_TZVPD=-1318.670402236831, M062X_TZVPD_CPCM_chcl3=-1318.641628687524
C -1.768040000000000 0.112273000000000 0.288277000000000
C -0.952382000000000 -0.233966000000000 -0.958492000000000
C 0.498674000000000 -0.379336000000000 -0.633495000000000
C 1.172085000000000 -1.478417000000000 -0.142116000000000
C 1.490290000000000 0.633601000000000 -0.735405000000000
C 1.655429000000000 2.077621000000000 -1.059509000000000
C 2.654129000000000 0.094844000000000 -0.306045000000000
O 2.511685000000000 -1.185841000000000 0.060069000000000
C 3.212358000000000 2.261055000000000 -0.977154000000000
C 3.820569000000000 1.007291000000000 -0.270421000000000
C -5.332443000000000 0.808581000000000 0.961994000000000
O -3.916437000000000 0.588624000000000 1.113331000000000
O -3.747769000000000 0.233184000000000 -1.098534000000000
O 1.537572000000000 -3.699572000000000 0.589969000000000
C -1.040400000000000 -4.338490000000000 0.335837000000000
O -0.543194000000000 -3.023238000000000 0.031546000000000
C 0.779326000000000 -2.835790000000000 0.197649000000000
C -3.232066000000000 0.308797000000000 -0.006865000000000
H -1.393760000000000 1.030790000000000 0.747064000000000
H -1.068232000000000 0.562631000000000 -1.693966000000000
H -1.338351000000000 -1.149746000000000 -1.401078000000000
H 1.281947000000000 2.348083000000000 -2.047324000000000
H 3.603685000000000 2.327206000000000 -1.992107000000000
H 3.474881000000000 3.183351000000000 -0.463324000000000
H 4.689569000000000 0.618808000000000 -0.804055000000000
H 4.133399000000000 1.217328000000000 0.756109000000000
H -5.514500000000000 1.653369000000000 0.299124000000000
H -5.701972000000000 1.021116000000000 1.960909000000000
H -5.812408000000000 -0.082022000000000 0.558469000000000
H -0.854743000000000 -4.581194000000000 1.381335000000000
H -0.563147000000000 -5.081728000000000 -0.301457000000000
H -2.107451000000000 -4.295702000000000 0.138074000000000
S 0.681457000000000 3.180802000000000 0.045692000000000
C 1.243831000000000 2.634180000000000 1.679551000000000
H 1.029283000000000 1.577031000000000 1.830269000000000
H 2.308338000000000 2.822125000000000 1.814131000000000
H 0.685720000000000 3.219452000000000 2.408956000000000
H -1.675514000000000 -0.673388000000000 1.040862000000000
38
R5_SMe6H_cyc4SZ_0_1 charge=0 multiplicity=1 B3LYP_D3BJ_TZVPD_SMD_chcl3=-1318.492738424456, B3LYP_TZVP_CPCM_chcl3_freq=0.25476103, B3LYP_TZVPD_CPCM_chcl3=-1318.486823778679, M062X_TZVPD_SMD_chcl3=-1318.59645967038, M062X_TZVPD=-1318.571521680324, COSMORS=-0.00321617454940563, wB97MV_TZVPD_CPCM_chcl3=-1318.631498332406, wB97MV_TZVPD_SMD_chcl3=-1318.637586607731, wB97MV_TZVPD=-1318.612910698599, M062X_TZVPD_CPCM_chcl3=-1318.590509467776
C -2.154673000000000 0.611145000000000 0.068899000000000
C -0.773077000000000 0.754387000000000 -0.302575000000000
C 0.231226000000000 -0.088630000000000 0.012160000000000
C 0.139375000000000 -1.395819000000000 0.817551000000000
C 1.642462000000000 0.025838000000000 -0.258976000000000
C 2.591696000000000 0.965192000000000 -0.911846000000000
C 2.291184000000000 -0.935559000000000 0.404554000000000
O 1.504686000000000 -1.784477000000000 1.116712000000000
C 3.967419000000000 0.241085000000000 -0.721436000000000
C 3.769333000000000 -0.889388000000000 0.332211000000000
C -4.887253000000000 0.327426000000000 0.778518000000000
O -4.411669000000000 1.458492000000000 0.043583000000000
O -2.821508000000000 2.628533000000000 -0.967933000000000
O 0.105476000000000 -3.193686000000000 -0.809183000000000
C -2.556580000000000 -3.474418000000000 -0.635583000000000
O -1.806820000000000 -2.565899000000000 0.197581000000000
C -0.491724000000000 -2.501080000000000 -0.024177000000000
C -3.101940000000000 1.515573000000000 -0.260236000000000
H -2.462464000000000 -0.257561000000000 0.626372000000000
H -0.502548000000000 1.635745000000000 -0.871737000000000
H -0.403903000000000 -1.291938000000000 1.756483000000000
H 2.379526000000000 1.138294000000000 -1.968196000000000
H 4.263008000000000 -0.198076000000000 -1.674409000000000
H 4.746170000000000 0.942486000000000 -0.427959000000000
H 4.211076000000000 -1.835933000000000 0.012437000000000
H 4.206471000000000 -0.640820000000000 1.304491000000000
H -4.703206000000000 -0.593691000000000 0.222752000000000
H -5.955286000000000 0.482839000000000 0.901184000000000
H -4.401957000000000 0.273560000000000 1.754643000000000
H -3.590862000000000 -3.366613000000000 -0.323565000000000
H -2.212459000000000 -4.495503000000000 -0.480230000000000
H -2.440295000000000 -3.201132000000000 -1.683145000000000
S 2.517600000000000 2.677664000000000 -0.223918000000000
C 2.703734000000000 2.352961000000000 1.549549000000000
H 1.904416000000000 1.706575000000000 1.909037000000000
H 3.672112000000000 1.905069000000000 1.770389000000000
H 2.637286000000000 3.318011000000000 2.050099000000000
H -3.639578000000000 3.128564000000000 -1.101617000000000
41
R6_SMe6_cyc2RP1_0_1 charge=0 multiplicity=1 B3LYP_D3BJ_TZVPD=-1357.832592836472, B3LYP_D3BJ_TZVPD_SMD_chcl3=-1357.855385511263, B3LYP_TZVP_CPCM_chcl3_freq=0.28378274, B3LYP_TZVPD_CPCM_chcl3=-1357.848438461318, M062X_TZVPD_SMD_chcl3=-1357.959609667178, M062X_TZVPD=-1357.936216694636, COSMORS=-0.005829951819370961, wB97MV_TZVPD_CPCM_chcl3=-1357.99478718836, wB97MV_TZVPD_SMD_chcl3=-1358.001907870979, wB97MV_TZVPD=-1357.978698599477, M062X_TZVPD_CPCM_chcl3=-1357.952712582377
O 4.464363000000000 0.414196000000000 -1.285377000000000
C 3.813547000000000 0.583846000000000 -0.129452000000000
C -1.882330000000000 2.180607000000000 0.476431000000000
H 6.239780000000000 0.173923000000000 -2.226336000000000
C 1.503710000000000 0.306633000000000 0.834032000000000
C 2.329953000000000 0.754930000000000 -0.357977000000000
H 6.324538000000000 1.203835000000000 -0.766627000000000
O 4.357000000000000 0.624918000000000 0.949463000000000
C 5.900467000000000 0.300573000000000 -1.202727000000000
H 6.174049000000000 -0.562550000000000 -0.597939000000000
H -3.144653000000000 5.071547000000000 -0.291857000000000
H 0.212883000000000 2.594393000000000 0.570272000000000
C 0.010388000000000 0.515516000000000 0.652275000000000
H -3.775668000000000 3.983480000000000 0.977907000000000
C -0.489974000000000 1.770727000000000 0.562722000000000
C -0.548549000000000 -0.809532000000000 0.598733000000000
C -1.932831000000000 -1.278375000000000 0.294333000000000
H -3.881160000000000 3.504504000000000 -0.730802000000000
S -2.223029000000000 -1.015623000000000 -1.513449000000000
C 0.466674000000000 -1.689903000000000 0.801013000000000
H -1.152255000000000 -4.669115000000000 0.544500000000000
H -2.658888000000000 -0.638210000000000 0.785750000000000
C 0.367936000000000 -3.167007000000000 0.848882000000000
O 1.673449000000000 -1.129638000000000 0.990039000000000
H -3.066948000000000 -3.114904000000000 0.323005000000000
C -0.967714000000000 -3.632996000000000 0.258248000000000
H 1.218277000000000 -3.605562000000000 0.321385000000000
H 0.459296000000000 -3.478050000000000 1.896222000000000
C -2.120400000000000 -2.740545000000000 0.715241000000000
H -0.908181000000000 -3.605021000000000 -0.832341000000000
H -2.195342000000000 -2.769186000000000 1.808254000000000
C -4.034619000000000 -0.924759000000000 -1.535735000000000
H -4.481923000000000 -1.859420000000000 -1.198753000000000
H -4.373762000000000 -0.099279000000000 -0.911162000000000
H -4.330823000000000 -0.743256000000000 -2.568280000000000
O -1.961767000000000 3.477872000000000 0.082763000000000
O -2.878388000000000 1.532654000000000 0.743455000000000
C -3.281511000000000 4.036031000000000 0.007952000000000
H 1.868535000000000 0.767672000000000 1.750929000000000
H 2.020891000000000 0.219473000000000 -1.255396000000000
H 2.150729000000000 1.818665000000000 -0.537378000000000
44
R5_SMe1_P2_SMe6RS charge=0 multiplicity=1 B3LYP_D3BJ_TZVPD=-1757.195086862932, B3LYP_D3BJ_TZVPD_SMD_chcl3=-1757.221559572494, B3LYP_TZVP_CPCM_chcl3_freq=0.30169391, B3LYP_TZVPD_CPCM_chcl3=-1757.215474888497, M062X_TZVPD_SMD_chcl3=-1757.346878298749, M062X_TZVPD=-1757.319892819905, COSMORS=-0.0028906177117112336, wB97MV_TZVPD_CPCM_chcl3=-1757.378508526226, wB97MV_TZVPD_SMD_chcl3=-1757.384657422472, wB97MV_TZVPD=-1757.357789729996, M062X_TZVPD_CPCM_chcl3=-1757.341044387005
C 2.255051000000000 -3.811967000000000 -0.721214000000000
C -5.425172000000000 1.233296000000000 -0.304986000000000
C -0.324724000000000 3.652319000000000 -0.298241000000000
C -2.049830000000000 -2.796456000000000 0.503693000000000
C 3.293783000000000 0.467104000000000 1.652308000000000
C 3.223102000000000 1.647260000000000 0.683153000000000
C -0.866573000000000 -0.217428000000000 -0.677911000000000
C 1.407363000000000 -0.313764000000000 -1.591784000000000
C 0.427478000000000 0.049977000000000 -0.498180000000000
C 1.976785000000000 -0.261368000000000 1.484847000000000
C 1.727554000000000 2.007828000000000 0.653792000000000
C -1.976467000000000 0.038190000000000 0.284618000000000
C 2.272170000000000 -1.508727000000000 -1.242439000000000
C 0.960432000000000 0.660895000000000 0.774309000000000
C -3.309445000000000 0.189463000000000 -0.415113000000000
O 1.736511000000000 -1.386081000000000 1.850698000000000
O 3.469133000000000 -1.463222000000000 -1.067850000000000
O -3.648332000000000 -0.433969000000000 -1.395212000000000
O 1.550334000000000 -2.627856000000000 -1.142989000000000
O -4.087490000000000 1.078713000000000 0.211021000000000
S 1.335717000000000 3.083487000000000 -0.755366000000000
S -2.191410000000000 -1.314999000000000 1.538961000000000
H 2.693320000000000 -3.649032000000000 0.262106000000000
H 3.035578000000000 -4.063233000000000 -1.437673000000000
H 1.505284000000000 -4.596330000000000 -0.680845000000000
H -5.393178000000000 1.566663000000000 -1.341042000000000
H -5.893379000000000 1.983741000000000 0.324712000000000
H -5.961839000000000 0.287880000000000 -0.239694000000000
H -1.049317000000000 2.842132000000000 -0.350240000000000
H -0.601070000000000 4.421359000000000 -1.018269000000000
H -0.317039000000000 4.084649000000000 0.702203000000000
H -2.823685000000000 -2.814003000000000 -0.261424000000000
H -2.190290000000000 -3.639049000000000 1.179778000000000
H -1.061994000000000 -2.855728000000000 0.050849000000000
H 3.334134000000000 0.814678000000000 2.690874000000000
H 4.131259000000000 -0.210017000000000 1.493883000000000
H 3.566233000000000 1.337843000000000 -0.305454000000000
H 3.829325000000000 2.495683000000000 0.995346000000000
H -1.173389000000000 -0.684577000000000 -1.605323000000000
H 2.074016000000000 0.515862000000000 -1.813939000000000
H 0.851931000000000 -0.556517000000000 -2.498048000000000
H 1.500102000000000 2.585746000000000 1.551495000000000
H -1.804668000000000 0.916115000000000 0.902845000000000
H 0.132849000000000 0.775897000000000 1.471702000000000
35
R6_rot_barrier2_0_1 charge=0 multiplicity=1 B3LYP_D3BJ_TZVPD=-919.121974725482, B3LYP_D3BJ_TZVPD_SMD_chcl3=-919.144136492896, B3LYP_TZVP_CPCM_chcl3_freq=0.23309065, B3LYP_TZVPD_CPCM_chcl3=-919.138449231143, M062X_TZVPD_SMD_chcl3=-919.211640073918, M062X_TZVPD=-919.189285407533, COSMORS=-0.004395168701010921, wB97MV_TZVPD_CPCM_chcl3=-919.2594431933, wB97MV_TZVPD_SMD_chcl3=-919.265308780895, wB97MV_TZVPD=-919.243050317253, M062X_TZVPD_CPCM_chcl3=-919.205966918361
C 2.066454000000000 0.823000000000000 0.354269000000000
C 1.306580000000000 0.133411000000000 -0.499227000000000
C -0.144636000000000 -0.108277000000000 -0.312984000000000
C -0.846392000000000 1.043931000000000 -0.139063000000000
C -0.617719000000000 -1.509323000000000 -0.402343000000000
C -1.895861000000000 -1.877489000000000 -0.148793000000000
C 0.375377000000000 -2.600672000000000 -0.627622000000000
O 1.580050000000000 -2.407014000000000 -0.606150000000000
H -3.607704000000000 4.273031000000000 0.543534000000000
C -0.137670000000000 -4.017965000000000 -0.783151000000000
C 5.454110000000000 2.097576000000000 0.885043000000000
O 4.065506000000000 1.766963000000000 1.062517000000000
O 4.066739000000000 0.783832000000000 -0.963913000000000
O -3.254780000000000 0.683507000000000 -0.246415000000000
C -3.723210000000000 3.220483000000000 0.301669000000000
O -2.386934000000000 2.697268000000000 0.218731000000000
C -2.275774000000000 1.380919000000000 -0.063334000000000
C 3.478115000000000 1.099128000000000 0.050149000000000
H 1.684685000000000 1.175381000000000 1.303320000000000
H 1.753044000000000 -0.228705000000000 -1.415136000000000
H -0.244639000000000 1.940800000000000 -0.108547000000000
C -2.439091000000000 -3.255670000000000 -0.290417000000000
H -4.236722000000000 3.101756000000000 -0.651835000000000
H -4.284506000000000 2.708118000000000 1.082336000000000
C -1.590332000000000 -4.111861000000000 -1.217752000000000
H -0.012680000000000 -4.487949000000000 0.200539000000000
H 6.046090000000000 1.191659000000000 0.759971000000000
H 5.746964000000000 2.622290000000000 1.789911000000000
H 5.582845000000000 2.738216000000000 0.013335000000000
H -2.598004000000000 -1.122756000000000 0.165793000000000
H 0.540057000000000 -4.534027000000000 -1.463974000000000
H -2.490779000000000 -3.709133000000000 0.709898000000000
H -3.474593000000000 -3.186429000000000 -0.633247000000000
H -1.695473000000000 -3.744254000000000 -2.242430000000000
H -1.926722000000000 -5.149479000000000 -1.208883000000000
40
R6_SMe1_TS_-1_1 charge=-1 multiplicity=1 wB97MV_TZVPD_SMD_chcl3=-1357.468503055755, B3LYP_D3BJ_TZVPD=-1357.263108380077, M062X_TZVPD=-1357.356417445843, COSMORS=-0.009906209000682422, B3LYP_TZVP_CPCM_chcl3_freq=0.26543003, B3LYP_TZVPD_CPCM_chcl3=-1357.330955924289, M062X_TZVPD_CPCM_chcl3=-1357.426581748219, M062X_TZVPD_SMD_chcl3=-1357.428572332115, wB97MV_TZVPD_CPCM_chcl3=-1357.466205583593, B3LYP_D3BJ_TZVPD_SMD_chcl3=-1357.332890230047, wB97MV_TZVPD=-1357.397203595195
O 3.769321000000000 -0.373179000000000 -1.559699000000000
C 3.130679000000000 0.611907000000000 -0.893287000000000
C -2.891962000000000 1.557913000000000 0.251808000000000
H 4.986783000000000 0.771030000000000 -2.808603000000000
C 0.833136000000000 1.087266000000000 -0.116724000000000
C 1.855924000000000 0.124095000000000 -0.306918000000000
H 5.737977000000000 0.245019000000000 -1.284302000000000
O 3.571025000000000 1.740224000000000 -0.814764000000000
C 5.061814000000000 -0.043808000000000 -2.089062000000000
H 5.417855000000000 -0.947484000000000 -2.576583000000000
H -5.289746000000000 2.055899000000000 1.373800000000000
H -1.168921000000000 2.813487000000000 0.178264000000000
C -0.523511000000000 0.790719000000000 0.011657000000000
H 1.135432000000000 2.126296000000000 -0.063172000000000
C -1.493278000000000 1.780784000000000 0.151189000000000
C -0.931005000000000 -0.644308000000000 0.008149000000000
C -0.740645000000000 -1.416417000000000 1.086767000000000
H 1.558816000000000 -0.843919000000000 -0.683380000000000
S 2.763305000000000 -0.655184000000000 1.529872000000000
C -1.546299000000000 -1.205748000000000 -1.212376000000000
H -0.330431000000000 -3.646023000000000 -0.668460000000000
H -0.306001000000000 -0.968013000000000 1.972522000000000
C -2.154655000000000 -2.584329000000000 -1.083464000000000
O -1.570757000000000 -0.596491000000000 -2.271486000000000
H -2.025371000000000 -2.949255000000000 1.769619000000000
C -1.306470000000000 -3.494990000000000 -0.197091000000000
H -3.146649000000000 -2.450352000000000 -0.635944000000000
H -2.292814000000000 -2.995370000000000 -2.083805000000000
C -1.104178000000000 -2.862864000000000 1.176611000000000
H -1.774309000000000 -4.477161000000000 -0.104292000000000
H -0.334208000000000 -3.394867000000000 1.741794000000000
C 2.169523000000000 0.629144000000000 2.653301000000000
H 1.295599000000000 1.104751000000000 2.193880000000000
H 1.863493000000000 0.177074000000000 3.596672000000000
H 2.928415000000000 1.387944000000000 2.843202000000000
O -3.574592000000000 2.753461000000000 0.411757000000000
O -3.521080000000000 0.498330000000000 0.212148000000000
C -4.992919000000000 2.652302000000000 0.509055000000000
H -5.356736000000000 3.672057000000000 0.621107000000000
H -5.420540000000000 2.202613000000000 -0.388850000000000
38
R5_SMe6H_cyc4RZTS_0_1 charge=0 multiplicity=1 B3LYP_D3BJ_TZVPD=-1318.424244734784, B3LYP_D3BJ_TZVPD_SMD_chcl3=-1318.451293779216, B3LYP_TZVP_CPCM_chcl3_freq=0.25227475, B3LYP_TZVPD_CPCM_chcl3=-1318.445895856793, M062X_TZVPD_SMD_chcl3=-1318.536830083545, M062X_TZVPD=-1318.508226274932, COSMORS=-0.002262292536880618, wB97MV_TZVPD_CPCM_chcl3=-1318.575179339015, wB97MV_TZVPD_SMD_chcl3=-1318.580825517788, wB97MV_TZVPD=-1318.552449387125, M062X_TZVPD_CPCM_chcl3=-1318.531341326466
C 1.695051000000000 1.563147000000000 -0.422115000000000
C 0.315497000000000 1.402613000000000 -0.622314000000000
C -0.420200000000000 0.245738000000000 -0.421309000000000
C -1.838822000000000 0.386098000000000 -0.444839000000000
C -0.010880000000000 -1.128209000000000 -0.358287000000000
C 1.304669000000000 -1.824179000000000 -0.252747000000000
C -0.964479000000000 -1.931211000000000 -0.936534000000000
O -2.153198000000000 -1.519663000000000 -1.219904000000000
C 0.951006000000000 -3.299719000000000 -0.585220000000000
C -0.388422000000000 -3.261397000000000 -1.349357000000000
C 2.403100000000000 5.087931000000000 -1.159324000000000
O 1.692971000000000 3.838633000000000 -1.017609000000000
O 3.641601000000000 2.945241000000000 -0.399564000000000
O -2.363469000000000 -0.512445000000000 1.725901000000000
C -4.976921000000000 -0.194256000000000 1.342863000000000
O -4.003379000000000 0.198833000000000 0.359534000000000
C -2.719688000000000 -0.047421000000000 0.666042000000000
C 2.337421000000000 2.763263000000000 -0.606825000000000
H 2.292474000000000 0.737535000000000 -0.063195000000000
H -0.251092000000000 2.288963000000000 -0.887769000000000
H -2.274926000000000 1.158374000000000 -1.069292000000000
H 2.044404000000000 -1.437196000000000 -0.961243000000000
H 1.748159000000000 -3.782447000000000 -1.148927000000000
H 0.822726000000000 -3.857640000000000 0.341902000000000
H -0.244304000000000 -3.268493000000000 -2.434883000000000
H -1.049731000000000 -4.094319000000000 -1.103074000000000
H 3.187075000000000 4.994476000000000 -1.908722000000000
H 2.828496000000000 5.392204000000000 -0.204598000000000
H 1.650826000000000 5.799657000000000 -1.484080000000000
H -5.941839000000000 0.064487000000000 0.916702000000000
H -4.915033000000000 -1.266253000000000 1.526498000000000
H -4.813003000000000 0.345253000000000 2.274984000000000
S 2.183905000000000 -1.625052000000000 1.357524000000000
C 0.854287000000000 -1.992017000000000 2.534956000000000
H 1.259074000000000 -1.800614000000000 3.528005000000000
H -0.002509000000000 -1.345831000000000 2.353908000000000
H 0.547610000000000 -3.035807000000000 2.472511000000000
H 4.050504000000000 2.128230000000000 -0.077309000000000
55
Sulf_SMe6_P1R_0_1 charge=0 multiplicity=1 B3LYP_D3BJ_TZVPD=-2138.40823661616, B3LYP_D3BJ_TZVPD_SMD_chcl3=-2138.443706474427, B3LYP_TZVP_CPCM_chcl3_freq=0.37071585, B3LYP_TZVPD_CPCM_chcl3=-2138.43330974165, M062X_TZVPD_SMD_chcl3=-2138.594889104127, M062X_TZVPD=-2138.557877533389, COSMORS=-0.008179286866775187, wB97MV_TZVPD_CPCM_chcl3=-2138.659471289676, wB97MV_TZVPD_SMD_chcl3=-2138.670327339179, wB97MV_TZVPD=-2138.633994854013, M062X_TZVPD_CPCM_chcl3=-2138.584125175674
C 3.123917000000000 -1.952847000000000 -0.805660000000000
C 3.756413000000000 -2.871717000000000 0.018433000000000
C 4.392448000000000 -2.442513000000000 1.180059000000000

C 4.399415000000000 -1.094885000000000 1.521053000000000
C 3.761927000000000 -0.167406000000000 0.708593000000000
C 3.128577000000000 -0.608973000000000 -0.444370000000000
O 2.585107000000000 1.900677000000000 -1.013457000000000
O 2.682090000000000 0.227577000000000 -2.863068000000000
S 2.319064000000000 0.562794000000000 -1.500280000000000
C -0.236881000000000 -0.420623000000000 -3.951790000000000
S -0.343397000000000 0.944373000000000 -2.761974000000000
C 0.526730000000000 -1.128771000000000 1.471223000000000
C 1.020224000000000 -1.640035000000000 2.663449000000000
C 1.497093000000000 -0.781757000000000 3.648236000000000
C 1.479124000000000 0.590707000000000 3.431053000000000
C 0.989303000000000 1.100330000000000 2.234846000000000
C 0.512414000000000 0.246459000000000 1.243248000000000
C -5.392259000000000 -4.030787000000000 0.463289000000000
O -4.817399000000000 -2.733993000000000 0.711619000000000
O -3.215064000000000 -3.168029000000000 -0.810789000000000
O -0.848152000000000 3.601900000000000 0.766277000000000
C -2.845633000000000 5.307391000000000 1.117374000000000
O -3.072609000000000 3.921050000000000 0.806431000000000
C -1.972763000000000 3.158287000000000 0.650459000000000
C -3.721534000000000 -2.421915000000000 0.001327000000000
C -2.373680000000000 1.777426000000000 0.339352000000000
C 0.512843000000000 0.236018000000000 -1.329100000000000
C -0.051010000000000 0.847995000000000 -0.034968000000000
C -1.569475000000000 0.747184000000000 0.021712000000000
C -2.154411000000000 -0.568691000000000 -0.245590000000000
C -3.235350000000000 -1.074084000000000 0.353363000000000
H 2.636138000000000 -2.273106000000000 -1.716204000000000
H 3.756860000000000 -3.920639000000000 -0.247207000000000
H 4.883184000000000 -3.162896000000000 1.821824000000000
H 4.892297000000000 -0.764440000000000 2.425511000000000
H 3.744005000000000 0.880881000000000 0.965495000000000
H 0.794347000000000 -0.596102000000000 -4.243512000000000
H -0.679564000000000 -1.321118000000000 -3.527558000000000
H -0.818923000000000 -0.106393000000000 -4.817157000000000
H 0.169123000000000 -1.814413000000000 0.714714000000000
H 1.039291000000000 -2.710878000000000 2.820001000000000
H 1.887240000000000 -1.181903000000000 4.575414000000000
H 1.853541000000000 1.266741000000000 4.189388000000000
H 0.979356000000000 2.167723000000000 2.060419000000000
H -5.697271000000000 -4.114363000000000 -0.579004000000000
H -6.254207000000000 -4.097192000000000 1.120430000000000
H -4.671233000000000 -4.813260000000000 0.696251000000000
H -2.297141000000000 5.399631000000000 2.054027000000000
H -2.282321000000000 5.786655000000000 0.317632000000000
H -3.833155000000000 5.750230000000000 1.207451000000000
H -3.442986000000000 1.617271000000000 0.343772000000000
H 0.423565000000000 -0.848145000000000 -1.368955000000000
H 0.209971000000000 1.900734000000000 -0.042805000000000
H -1.651347000000000 -1.201587000000000 -0.966157000000000
H -3.777124000000000 -0.540410000000000 1.122505000000000
41
R6_SMe1_P2_0_1 charge=0 multiplicity=1 B3LYP_D3BJ_TZVPD=-1357.821881108064, B3LYP_D3BJ_TZVPD_SMD_chcl3=-1357.849050671229, B3LYP_TZVP_CPCM_chcl3_freq=0.2794355, B3LYP_TZVPD_CPCM_chcl3=-1357.842225352695, M062X_TZVPD_SMD_chcl3=-1357.947511082899, M062X_TZVPD=-1357.920117214931, COSMORS=-0.0006861569710023794, wB97MV_TZVPD_CPCM_chcl3=-1357.985409495951, wB97MV_TZVPD_SMD_chcl3=-1357.992411544305, wB97MV_TZVPD=-1357.965059854613, M062X_TZVPD_CPCM_chcl3=-1357.94074154129
O 2.980357000000000 0.747896000000000 -1.493045000000000
C 2.757763000000000 1.021595000000000 -0.204095000000000
O 3.214876000000000 1.978447000000000 0.375165000000000
H 2.290762000000000 -2.958336000000000 2.698772000000000
S 2.896148000000000 -0.917849000000000 1.672111000000000
C 1.858605000000000 -0.017709000000000 0.432210000000000
C 0.674861000000000 0.607784000000000 1.091468000000000
C 3.837271000000000 1.667957000000000 -2.202525000000000
H 3.896439000000000 1.283873000000000 -3.216282000000000
H 3.403177000000000 2.666443000000000 -2.193415000000000
H 4.823178000000000 1.691075000000000 -1.741102000000000
C 1.822685000000000 -2.357373000000000 1.920275000000000
H 0.832595000000000 -2.046737000000000 2.249357000000000
H 1.745091000000000 -2.944370000000000 1.005831000000000
C -0.596187000000000 0.253503000000000 0.893801000000000
H 1.567985000000000 -0.741782000000000 -0.322022000000000
H 0.906370000000000 1.375171000000000 1.821110000000000
C -1.018264000000000 -0.747365000000000 -0.116957000000000
C -1.710487000000000 0.897072000000000 1.689618000000000
H -2.231827000000000 0.146041000000000 2.281036000000000
H -1.298586000000000 1.656472000000000 2.354298000000000
C -2.729927000000000 1.537099000000000 0.772174000000000
O -2.223410000000000 2.611292000000000 0.152140000000000
O -3.853975000000000 1.129865000000000 0.589483000000000
C -3.087131000000000 3.277252000000000 -0.789172000000000
H -3.369541000000000 2.592652000000000 -1.587814000000000
H -3.981749000000000 3.642913000000000 -0.287373000000000
H -2.504499000000000 4.104727000000000 -1.182999000000000
C -0.859141000000000 -0.526529000000000 -1.433194000000000
C -1.685048000000000 -1.985697000000000 0.354832000000000
H -0.419558000000000 0.414565000000000 -1.747788000000000
C -1.251246000000000 -1.484183000000000 -2.508850000000000
H -3.284519000000000 -2.482863000000000 -0.922257000000000
H -0.467799000000000 -3.337432000000000 -1.755942000000000
H -1.917870000000000 -3.532385000000000 -2.731695000000000
O -1.760827000000000 -2.253405000000000 1.544185000000000
C -2.286151000000000 -2.881506000000000 -0.702105000000000
C -1.446197000000000 -2.900974000000000 -1.977531000000000
H -2.416752000000000 -3.875917000000000 -0.275712000000000
H -2.179038000000000 -1.118032000000000 -2.969165000000000
H -0.499098000000000 -1.460690000000000 -3.301526000000000
40
R6_SMe_complex charge=-1 multiplicity=1 wB97MV_TZVPD_SMD_chcl3=-1357.467992126556, B3LYP_D3BJ_TZVPD=-1357.261648743625, B3LYP_TZVP_CPCM_chcl3_freq=0.26479933, B3LYP_TZVPD_CPCM_chcl3=-1357.334444643165, M062X_TZVPD=-1357.349796030403, COSMORS=-0.010024103635038637, M062X_TZVPD_CPCM_chcl3=-1357.426501875507, M062X_TZVPD_SMD_chcl3=-1357.426525631766, wB97MV_TZVPD_CPCM_chcl3=-1357.467729491982, B3LYP_D3BJ_TZVPD_SMD_chcl3=-1357.335085547468, wB97MV_TZVPD=-1357.391223496569
C 1.684482000000000 1.316391000000000 -0.831834000000000
C 0.987182000000000 0.166865000000000 -1.007775000000000
C -0.296774000000000 -0.138523000000000 -0.451317000000000
C -0.891518000000000 -1.317906000000000 -0.794076000000000
C -0.941677000000000 0.832660000000000 0.471754000000000
C -0.584037000000000 0.918434000000000 1.760901000000000
C -2.004499000000000 1.712098000000000 -0.067290000000000
O -2.257231000000000 1.766301000000000 -1.261204000000000
H -4.068609000000000 -3.542183000000000 0.146669000000000
C -2.785305000000000 2.523484000000000 0.941082000000000
C 4.732810000000000 0.805914000000000 -2.812408000000000
O 3.455807000000000 0.584728000000000 -2.201838000000000
O 3.601803000000000 2.630769000000000 -1.268700000000000
O -3.034412000000000 -1.084302000000000 0.274548000000000
C -3.983911000000000 -3.251511000000000 -0.900753000000000
O -2.666058000000000 -2.763656000000000 -1.176655000000000
C -2.274604000000000 -1.681784000000000 -0.463634000000000
C 2.976825000000000 1.591933000000000 -1.430200000000000
H 1.301060000000000 2.112669000000000 -0.208933000000000
H 1.416039000000000 -0.598403000000000 -1.645052000000000
H -0.399923000000000 -1.968994000000000 -1.499882000000000
C -1.176579000000000 1.870173000000000 2.748734000000000
H -4.733415000000000 -2.492754000000000 -1.126239000000000
H -4.117098000000000 -4.117383000000000 -1.544150000000000
C -1.897303000000000 3.032959000000000 2.074058000000000
H -3.556745000000000 1.856982000000000 1.345901000000000
H 4.697894000000000 1.667920000000000 -3.478885000000000
H 4.947417000000000 -0.098009000000000 -3.376685000000000
H 5.499482000000000 0.969250000000000 -2.054921000000000
H 0.172356000000000 0.237585000000000 2.134570000000000
H -3.290620000000000 3.333749000000000 0.415117000000000
H -1.877047000000000 1.307921000000000 3.381728000000000
H -0.393723000000000 2.231546000000000 3.420856000000000
H -1.155418000000000 3.725408000000000 1.664741000000000
H -2.491840000000000 3.590517000000000 2.800220000000000
S -0.271880000000000 -3.319323000000000 1.088340000000000
C 1.208407000000000 -2.385732000000000 1.573921000000000
H 2.123634000000000 -2.943865000000000 1.365469000000000
H 1.258860000000000 -1.446864000000000 1.008683000000000
H 1.195942000000000 -2.128298000000000 2.635624000000000
40
R6_SMe6_-1_1 charge=-1 multiplicity=1 wB97MV_TZVPD_SMD_chcl3=-1357.48755004551, B3LYP_D3BJ_TZVPD=-1357.27950597877, B3LYP_TZVP_CPCM_chcl3_freq=0.26867994, B3LYP_TZVPD_CPCM_chcl3=-1357.345990145507, M062X_TZVPD=-1357.375986270662, COSMORS=-0.01131705622544919, M062X_TZVPD_CPCM_chcl3=-1357.444641651931, M062X_TZVPD_SMD_chcl3=-1357.446076611685, wB97MV_TZVPD_CPCM_chcl3=-1357.48586371531, B3LYP_D3BJ_TZVPD_SMD_chcl3=-1357.347414044122, wB97MV_TZVPD=-1357.41839108522
O -4.871193000000000 1.027449000000000 -0.184229000000000
C -3.924925000000000 0.162740000000000 0.259168000000000
C 1.232948000000000 2.307586000000000 -0.697975000000000
H -6.826756000000000 1.531792000000000 -0.231666000000000
C -1.514287000000000 -0.185348000000000 0.071624000000000
C -2.597501000000000 0.556289000000000 -0.207583000000000
H -6.533623000000000 -0.229879000000000 -0.169162000000000
O -4.202211000000000 -0.799114000000000 0.951480000000000
C -6.221281000000000 0.743741000000000 0.208342000000000
H -6.315554000000000 0.754680000000000 1.294218000000000
H 3.187532000000000 4.149762000000000 -0.515303000000000
H -0.603724000000000 2.153609000000000 0.322084000000000
C -0.126745000000000 0.184435000000000 -0.272710000000000
H 1.991209000000000 5.449484000000000 -0.260425000000000
C 0.141602000000000 1.546336000000000 -0.171277000000000
C 0.792467000000000 -0.879311000000000 -0.504801000000000
C 2.262181000000000 -0.698932000000000 -0.283309000000000
H 2.070268000000000 4.588431000000000 -1.822582000000000
S 2.688697000000000 -0.295990000000000 1.511949000000000
C 0.270416000000000 -2.161586000000000 -0.848166000000000
H 3.222588000000000 -4.021252000000000 -0.596511000000000
H 2.605592000000000 0.206382000000000 -0.775480000000000
C 1.174237000000000 -3.389955000000000 -0.868396000000000
O -0.924742000000000 -2.357175000000000 -1.172314000000000
H 4.150748000000000 -1.713999000000000 -0.449910000000000
C 2.571808000000000 -3.196241000000000 -0.295180000000000
H 0.630120000000000 -4.187355000000000 -0.355942000000000
H 1.243964000000000 -3.698492000000000 -1.917797000000000
C 3.119402000000000 -1.862877000000000 -0.774029000000000
H 2.542461000000000 -3.200999000000000 0.797654000000000
H 3.119900000000000 -1.844316000000000 -1.871135000000000
C 1.334586000000000 -1.020510000000000 2.473258000000000
H 1.594512000000000 -0.868000000000000 3.521106000000000
H 0.393164000000000 -0.517471000000000 2.261180000000000
H 1.227098000000000 -2.086826000000000 2.282840000000000
O 1.201313000000000 3.594780000000000 -0.207890000000000
O 2.087745000000000 1.985948000000000 -1.519490000000000
C 2.176248000000000 4.491028000000000 -0.740858000000000
H -1.660109000000000 -1.094324000000000 0.637678000000000
H -2.534368000000000 1.449804000000000 -0.813422000000000
44
R5_SMe4_P2_SMe2RP2R charge=0 multiplicity=1 B3LYP_D3BJ_TZVPD=-1757.201644379288, B3LYP_D3BJ_TZVPD_SMD_chcl3=-1757.225734973698, B3LYP_TZVP_CPCM_chcl3_freq=0.30163614, B3LYP_TZVPD_CPCM_chcl3=-1757.219470405123, M062X_TZVPD_SMD_chcl3=-1757.351687095343, M062X_TZVPD=-1757.326925011312, COSMORS=-0.004665602864977532, wB97MV_TZVPD_CPCM_chcl3=-1757.382895043921, wB97MV_TZVPD_SMD_chcl3=-1757.389271965601, wB97MV_TZVPD=-1757.364663242985, M062X_TZVPD_CPCM_chcl3=-1757.345577794257
C 4.231656000000000 -0.540558000000000 2.743766000000000
C -4.303884000000000 1.038244000000000 0.870357000000000
C -0.213495000000000 0.791368000000000 -3.402079000000000
C -0.784776000000000 3.426294000000000 1.282370000000000
C -0.423639000000000 -3.439521000000000 1.205917000000000
C 0.319459000000000 -2.091766000000000 1.181710000000000
C -1.769709000000000 -3.154652000000000 0.532076000000000
C 2.666053000000000 -0.330631000000000 -0.595933000000000
C -0.097020000000000 -1.472083000000000 -0.168001000000000
C -1.513486000000000 -1.995068000000000 -0.407802000000000
C 1.402630000000000 0.350723000000000 -1.121690000000000
C 2.998007000000000 0.068279000000000 0.821453000000000
C 0.146775000000000 0.000993000000000 -0.366501000000000
C -0.715865000000000 0.962862000000000 0.018041000000000
C -1.986653000000000 0.632034000000000 0.730613000000000
O -2.285347000000000 -1.570805000000000 -1.237336000000000
O 2.609552000000000 1.069157000000000 1.375731000000000
O -2.068093000000000 -0.141232000000000 1.661248000000000
O 3.812659000000000 -0.828068000000000 1.394240000000000
O -3.033953000000000 1.298578000000000 0.241954000000000
S 1.250150000000000 -0.146892000000000 -2.889010000000000
S -0.470482000000000 2.682895000000000 -0.346512000000000
H 3.365170000000000 -0.488713000000000 3.401324000000000
H 4.774333000000000 0.402937000000000 2.777284000000000
H 4.878815000000000 -1.364793000000000 3.028093000000000
H -4.260147000000000 1.296194000000000 1.927476000000000
H -4.568218000000000 -0.012032000000000 0.758327000000000
H -5.017124000000000 1.670340000000000 0.350179000000000
H -0.366567000000000 0.554723000000000 -4.454260000000000
H -0.046699000000000 1.861833000000000 -3.294966000000000
H -1.088963000000000 0.484028000000000 -2.833628000000000
H -0.087843000000000 3.032073000000000 2.019135000000000
H -1.813447000000000 3.260829000000000 1.598586000000000
H -0.617427000000000 4.495167000000000 1.157138000000000
H -0.533282000000000 -3.828298000000000 2.217529000000000
H 0.134221000000000 -4.178109000000000 0.625435000000000
H 1.396949000000000 -2.219512000000000 1.266298000000000
H -0.016618000000000 -1.449137000000000 1.992826000000000
H -2.510273000000000 -2.800599000000000 1.255416000000000
H -2.208106000000000 -3.997780000000000 -0.002024000000000
H 2.613106000000000 -1.416757000000000 -0.661808000000000
H 3.514370000000000 -0.028257000000000 -1.217201000000000
H 0.467120000000000 -1.994771000000000 -0.953701000000000
H 1.552249000000000 1.425549000000000 -1.097800000000000
38
R5_SMe4_P2_0_1 charge=0 multiplicity=1 B3LYP_D3BJ_TZVPD=-1318.514045507585, CCSDT_SMD_chcl3=-1316.771395563062, CCSDT=-1316.748473137146, B3LYP_D3BJ_TZVPD_SMD_chcl3=-1318.537792011503, B3LYP_TZVP_CPCM_chcl3_freq=0.25256834, B3LYP_TZVPD_CPCM_chcl3=-1318.532137374723, M062X_TZVPD_SMD_chcl3=-1318.630868721302, M062X_TZVPD=-1318.606784327141, COSMORS=-0.002799830237809184, wB97MV_TZVPD_CPCM_chcl3=-1318.671657828631, wB97MV_TZVPD_SMD_chcl3=-1318.677500957471, wB97MV_TZVPD=-1318.653471035592, M062X_TZVPD_CPCM_chcl3=-1318.625245067696
C 1.819447000000000 -0.459777000000000 -0.280295000000000
C 0.608841000000000 -0.972136000000000 -0.022102000000000
C -0.655534000000000 -0.255509000000000 0.011914000000000
C -0.662331000000000 1.245422000000000 -0.149931000000000
C -1.807669000000000 -0.954501000000000 0.168043000000000
C -1.929403000000000 -2.454869000000000 0.307132000000000
C -3.194157000000000 -0.386994000000000 0.220945000000000
O -3.524751000000000 0.783203000000000 0.150931000000000
C -3.321341000000000 -2.687167000000000 0.921650000000000
C -4.166191000000000 -1.538076000000000 0.373891000000000
C 5.364019000000000 -1.338458000000000 -0.507037000000000
O 4.120923000000000 -0.612905000000000 -0.514690000000000
O 3.026532000000000 -2.511745000000000 0.005496000000000
O 1.024747000000000 2.857640000000000 0.514767000000000
C 0.924398000000000 2.102916000000000 3.073353000000000
O 0.105610000000000 1.499198000000000 2.050482000000000
C 0.267766000000000 1.964491000000000 0.809330000000000
C 3.014218000000000 -1.323015000000000 -0.239676000000000
H 1.986719000000000 0.574309000000000 -0.540729000000000
H 0.570908000000000 -2.031859000000000 0.191616000000000
H -1.662685000000000 1.610305000000000 0.075782000000000
H -2.638705000000000 1.455101000000000 -2.365277000000000
H -3.722568000000000 -3.668600000000000 0.674786000000000
H -3.258982000000000 -2.613507000000000 2.009367000000000
H -4.539437000000000 -1.772508000000000 -0.630488000000000
H -5.020694000000000 -1.249574000000000 0.984271000000000
H 6.129412000000000 -0.606108000000000 -0.746090000000000
H 5.343497000000000 -2.129631000000000 -1.255456000000000
H 5.542082000000000 -1.771800000000000 0.476377000000000
H 0.704615000000000 3.166356000000000 3.151483000000000
H 1.978001000000000 1.960218000000000 2.838119000000000
H 0.662937000000000 1.588111000000000 3.992722000000000
S -0.284901000000000 1.771298000000000 -1.869777000000000
C -1.702694000000000 1.035261000000000 -2.727520000000000
H -1.572880000000000 1.287032000000000 -3.779291000000000
H -1.711823000000000 -0.048405000000000 -2.620890000000000
H -1.874899000000000 -2.918249000000000 -0.683991000000000
H -1.137893000000000 -2.896528000000000 0.909655000000000
44
R5_SMe6_P1_SMe2RS charge=0 multiplicity=1 B3LYP_D3BJ_TZVPD=-1757.206941083547, B3LYP_D3BJ_TZVPD_SMD_chcl3=-1757.232847651559, B3LYP_TZVP_CPCM_chcl3_freq=0.30194789, B3LYP_TZVPD_CPCM_chcl3=-1757.22646751653, M062X_TZVPD_SMD_chcl3=-1757.357368198907, M062X_TZVPD=-1757.33109031873, COSMORS=-0.0038206594467292297, wB97MV_TZVPD_CPCM_chcl3=-1757.388762636884, wB97MV_TZVPD_SMD_chcl3=-1757.395259989733, wB97MV_TZVPD=-1757.369112096808, M062X_TZVPD_CPCM_chcl3=-1757.351142287277
C 3.378139000000000 -3.681704000000000 -1.437114000000000
C -5.839660000000000 -0.501177000000000 -1.035762000000000
C 0.189368000000000 3.819262000000000 -0.052266000000000
C -1.436204000000000 -1.792009000000000 2.269483000000000
C 3.388819000000000 1.089785000000000 1.628100000000000
C 3.032701000000000 2.207877000000000 0.639335000000000
C 0.639902000000000 -1.438753000000000 -0.548986000000000
C -2.149949000000000 -0.709940000000000 -0.842277000000000
C 0.232564000000000 -0.305506000000000 0.041215000000000
C 2.159543000000000 0.203252000000000 1.704288000000000
C 1.956002000000000 1.592305000000000 -0.269237000000000
C -1.228391000000000 0.042034000000000 0.108440000000000
C 2.028281000000000 -1.892846000000000 -0.679254000000000
C -3.520330000000000 -0.078102000000000 -0.882806000000000
C 1.135640000000000 0.703633000000000 0.684477000000000
O 1.982829000000000 -0.698440000000000 2.487918000000000
O 3.030785000000000 -1.286216000000000 -0.355404000000000
O -3.727367000000000 1.113434000000000 -0.891017000000000
O 2.070001000000000 -3.115394000000000 -1.240503000000000
O -4.488770000000000 -0.998970000000000 -0.944506000000000
S 0.991921000000000 2.753648000000000 -1.281967000000000
S -1.808279000000000 -0.065348000000000 1.857470000000000
H 3.888534000000000 -3.795227000000000 -0.481529000000000
H 3.970908000000000 -3.045223000000000 -2.093018000000000
H 3.208950000000000 -4.650924000000000 -1.896892000000000
H -5.958970000000000 0.099942000000000 -1.935909000000000
H -6.076928000000000 0.099965000000000 -0.159450000000000
H -6.471281000000000 -1.383422000000000 -1.077619000000000
H 0.918742000000000 4.364783000000000 0.543647000000000
H -0.478229000000000 3.257528000000000 0.600099000000000
H -0.403467000000000 4.532160000000000 -0.623884000000000
H -1.791599000000000 -1.932754000000000 3.289620000000000
H -0.364267000000000 -1.972691000000000 2.232199000000000
H -1.966252000000000 -2.480268000000000 1.613166000000000
H 3.665407000000000 1.441126000000000 2.622504000000000
H 4.203890000000000 0.465159000000000 1.257001000000000
H 3.885471000000000 2.557904000000000 0.060305000000000
H 2.621722000000000 3.062213000000000 1.178951000000000
H -0.086838000000000 -2.109792000000000 -0.981821000000000
H -2.240887000000000 -1.763292000000000 -0.587405000000000
H -1.746728000000000 -0.652096000000000 -1.858762000000000
H 2.431841000000000 0.958605000000000 -1.015991000000000
H -1.328181000000000 1.108340000000000 -0.100744000000000
H 0.510683000000000 1.368016000000000 1.294371000000000
54
Sulf_SMe1_2_c80_-1_1 charge=-1 multiplicity=1 wB97MV_TZVPD_SMD_chcl3=-2138.157900274233, B3LYP_D3BJ_TZVPD=-2137.853623169273,

B3LYP_TZVP_CPCM_chcl3_freq=0.35550374,
B3LYP_TZVPD_CPCM_chcl3=-2137.923850774962, M062X_TZVPD=-2138.004156030562, COSMORS=-0.015052171147006186,
M062X_TZVPD_CPCM_chcl3=-2138.077791072223,
M062X_TZVPD_SMD_chcl3=-2138.084337332836,
wB97MV_TZVPD_CPCM_chcl3=-2138.151251985562,
B3LYP_D3BJ_TZVPD_SMD_chcl3=-2137.930037734499,
wB97MV_TZVPD=-2138.078975833653
C 1.913040000000000 1.315817000000000 1.867655000000000
C 2.336983000000000 2.551310000000000 1.400282000000000
C 3.272043000000000 2.623010000000000 0.370138000000000
C 3.791410000000000 1.459922000000000 -0.187634000000000
C 3.368290000000000 0.217498000000000 0.270197000000000
C 2.427172000000000 0.158439000000000 1.288960000000000
O 1.733559000000000 -1.362476000000000 3.304870000000000
O 2.642528000000000 -2.455068000000000 1.264821000000000
S 1.818943000000000 -1.415897000000000 1.855042000000000
C -3.511536000000000 0.775502000000000 3.231248000000000
S -1.764402000000000 1.169748000000000 2.967121000000000
C 0.292384000000000 0.242960000000000 -1.841941000000000
C 1.077179000000000 0.553018000000000 -2.943997000000000
C 2.070932000000000 -0.325839000000000 -3.364265000000000
C 2.271913000000000 -1.515531000000000 -2.672964000000000
C 1.502934000000000 -1.816494000000000 -1.556430000000000
C 0.510782000000000 -0.935554000000000 -1.121213000000000
C -1.596974000000000 4.756212000000000 0.179400000000000
O -1.214115000000000 3.405630000000000 0.491292000000000
O -3.383995000000000 2.817602000000000 0.536611000000000
O -2.812090000000000 -4.112045000000000 -1.966095000000000
C -0.362566000000000 -4.930681000000000 -1.454447000000000
O -0.848473000000000 -3.709853000000000 -0.910781000000000
C -2.125016000000000 -3.330375000000000 -1.287820000000000
C -2.215751000000000 2.524277000000000 0.667887000000000
C -2.529461000000000 -2.062435000000000 -0.829081000000000
C 0.150700000000000 -1.483566000000000 1.297072000000000
C -0.320217000000000 -1.223261000000000 0.071591000000000
C -1.803259000000000 -1.086301000000000 -0.104825000000000
C -2.383249000000000 0.064125000000000 0.374363000000000
C -1.686837000000000 1.167658000000000 1.054836000000000
H 1.189531000000000 1.247824000000000 2.668734000000000
H 1.933396000000000 3.455877000000000 1.835631000000000
H 3.595423000000000 3.588731000000000 0.002786000000000
H 4.518081000000000 1.515922000000000 -0.987609000000000
H 3.749279000000000 -0.694208000000000 -0.163302000000000
H -3.768269000000000 -0.144375000000000 2.706952000000000
H -3.639208000000000 0.627732000000000 4.302990000000000
H -4.154413000000000 1.587665000000000 2.895724000000000
H -0.491824000000000 0.919874000000000 -1.532083000000000
H 0.907032000000000 1.478504000000000 -3.479753000000000
H 2.677500000000000 -0.088794000000000 -4.229475000000000
H 3.032769000000000 -2.213071000000000 -3.000581000000000
H 1.662720000000000 -2.737497000000000 -1.020314000000000
H -2.193884000000000 5.175867000000000 0.988674000000000
H -0.665633000000000 5.304642000000000 0.069542000000000
H -2.168685000000000 4.785762000000000 -0.747381000000000
H -0.988437000000000 -5.775113000000000 -1.161834000000000
H 0.640285000000000 -5.057479000000000 -1.049642000000000
H -0.316618000000000 -4.889701000000000 -2.544819000000000
H -3.566069000000000 -1.830454000000000 -1.043687000000000
H -0.517643000000000 -1.652440000000000 2.130169000000000
H -3.444798000000000 0.202240000000000 0.209264000000000
H -0.613389000000000 1.155139000000000 0.898120000000000
54
Sulf_SMe4_c13_-1_1 charge=-1 multiplicity=1
wB97MV_TZVPD_SMD_chcl3=-2138.17905155505, B3LYP_D3BJ_TZVPD=-2137.875328665387, B3LYP_TZVP_CPCM_chcl3_freq=0.35621466,
B3LYP_TZVPD_CPCM_chcl3=-2137.944635723073, M062X_TZVPD=-2138.028132311241, COSMORS=-0.0162027195129497,
M062X_TZVPD_CPCM_chcl3=-2138.099948359837,
M062X_TZVPD_SMD_chcl3=-2138.106225197996,
wB97MV_TZVPD_CPCM_chcl3=-2138.172655764954,
B3LYP_D3BJ_TZVPD_SMD_chcl3=-2137.950518208765,
wB97MV_TZVPD=-2138.102143193293
C 3.240766000000000 2.847980000000000 0.374109000000000
C 3.644309000000000 3.011002000000000 1.695048000000000
C 2.703239000000000 3.264961000000000 2.685833000000000
C 1.351541000000000 3.361349000000000 2.358586000000000
C 0.942263000000000 3.201021000000000 1.042808000000000
C 1.893158000000000 2.944679000000000 0.058714000000000
O 2.550360000000000 2.474441000000000 -2.414837000000000
O 0.573908000000000 3.904326000000000 -2.000215000000000
S 1.346659000000000 2.721383000000000 -1.629943000000000
C 0.261191000000000 -2.450678000000000 -3.124220000000000
S -0.749192000000000 -3.290146000000000 -1.876287000000000
C 1.865202000000000 -0.318576000000000 0.976838000000000
C 3.101662000000000 -0.640472000000000 1.518000000000000
C 4.199075000000000 -0.835082000000000 0.683936000000000
C 4.049966000000000 -0.700906000000000 -0.692105000000000
C 2.813744000000000 -0.363845000000000 -1.231586000000000
C 1.708725000000000 -0.169248000000000 -0.403963000000000
C -6.835919000000000 -0.646006000000000 -0.774652000000000
O -5.516042000000000 -1.193545000000000 -0.786984000000000
O -4.714030000000000 0.919510000000000 -0.791090000000000
O -1.776337000000000 -3.758458000000000 0.775609000000000
C -1.775634000000000 -2.317869000000000 3.003950000000000
O -1.111740000000000 -1.880800000000000 1.805176000000000
C -1.205482000000000 -2.692119000000000 0.745186000000000
C -4.486075000000000 -0.286480000000000 -0.794884000000000
C -0.453551000000000 -2.125803000000000 -0.448412000000000
C 0.238310000000000 1.407167000000000 -1.623967000000000
C 0.375640000000000 0.204825000000000 -0.959358000000000
C -0.721274000000000 -0.680203000000000 -0.747613000000000
C -2.043624000000000 -0.219245000000000 -0.849998000000000
C -3.209411000000000 -0.943661000000000 -0.810561000000000
H 3.955275000000000 2.627342000000000 -0.405153000000000
H 4.693101000000000 2.926232000000000 1.949495000000000
H 3.018938000000000 3.382689000000000 3.714866000000000
H 0.617551000000000 3.555730000000000 3.130210000000000
H -0.105916000000000 3.259067000000000 0.780594000000000
H 1.305706000000000 -2.402586000000000 -2.819085000000000
H -0.111259000000000 -1.446229000000000 -3.316322000000000
H 0.180061000000000 -3.046139000000000 -4.033117000000000
H 1.009648000000000 -0.169509000000000 1.621296000000000
H 3.211167000000000 -0.734228000000000 2.591210000000000
H 5.164382000000000 -1.087935000000000 1.104766000000000
H 4.898054000000000 -0.853081000000000 -1.348152000000000
H 2.701860000000000 -0.242273000000000 -2.299245000000000
H -7.510363000000000 -1.499480000000000 -0.768361000000000
H -6.997540000000000 -0.034771000000000 0.114345000000000
H -7.014204000000000 -0.034830000000000 -1.660394000000000
H -2.841932000000000 -2.440409000000000 2.818038000000000
H -1.353874000000000 -3.260737000000000 3.350533000000000
H -1.603821000000000 -1.531540000000000 3.733310000000000
H 0.600129000000000 -2.249666000000000 -0.197775000000000
H -0.690098000000000 1.699548000000000 -2.091670000000000
H -2.170236000000000 0.856493000000000 -0.917701000000000
H -3.218034000000000 -2.023188000000000 -0.814810000000000
6
SMe_0_1 charge=0 multiplicity=1 B3LYP_D3BJ_TZVPD=-438.659589710546, CCSDT_SMD_chcl3=-438.182474553087, CCSDT=-438.177123034316, B3LYP_D3BJ_TZVPD_SMD_chcl3=-438.665018069573,
B3LYP_TZVP_CPCM_chcl3_freq=0.02131749,
B3LYP_TZVPD_CPCM_chcl3=-438.663169856228,
M062X_TZVPD_SMD_chcl3=-438.688646747461, M062X_TZVPD=-438.683096935503, COSMORS=-8.398279576334948e-05,
wB97MV_TZVPD_CPCM_chcl3=-438.676081873501,
wB97MV_TZVPD_SMD_chcl3=-438.677957749351, wB97MV_TZVPD=-438.67247290082, M062X_TZVPD_CPCM_chcl3=-438.686807683894
C -7.131587000000000 2.716542000000000 -0.119045000000000
S -5.747073000000000 2.283222000000000 0.998343000000000
H -6.851040000000000 3.524072000000000 -0.809979000000000
H -7.478957000000000 1.840613000000000 -0.684992000000000
H -7.948679000000000 3.069253000000000 0.526198000000000
H -4.873397000000000 1.890067000000000 0.042688000000000
41
R6_SMe6_cyc4REP1_0_1 charge=0 multiplicity=1
B3LYP_D3BJ_TZVPD_SMD_chcl3=-1357.84226421142,
B3LYP_TZVP_CPCM_chcl3_freq=0.28252174,
B3LYP_TZVPD_CPCM_chcl3=-1357.83569579684,
M062X_TZVPD_SMD_chcl3=-1357.948675756896, M062X_TZVPD=-1357.924137254759, COSMORS=-0.004313608179432777,
wB97MV_TZVPD_CPCM_chcl3=-1357.984166923203,
wB97MV_TZVPD_SMD_chcl3=-1357.982205606554, wB97MV_TZVPD=-1357.966661258022, M062X_TZVPD_CPCM_chcl3=-1357.9420948827
O -4.247625000000000 0.124223000000000 0.650278000000000
C -3.165217000000000 0.918368000000000 0.671343000000000
C 1.716821000000000 2.340205000000000 -0.468045000000000
H -5.465800000000000 1.210691000000000 -0.648128000000000
C -0.677918000000000 0.941150000000000 1.003004000000000
C -1.928380000000000 0.120436000000000 1.007968000000000
H -6.244851000000000 -0.034209000000000 0.371101000000000
O -3.209714000000000 2.105848000000000 0.448306000000000
C -5.503569000000000 0.759051000000000 0.342245000000000
H -5.732844000000000 1.523279000000000 1.083694000000000
H 0.163427000000000 3.846486000000000 -2.005398000000000
H 1.897246000000000 2.000833000000000 1.648849000000000
C 0.559208000000000 0.496098000000000 0.759499000000000
H 1.681760000000000 4.720020000000000 -1.675574000000000
C 1.785121000000000 1.413823000000000 0.738889000000000
C 1.109623000000000 -0.822973000000000 0.477338000000000
C 0.420489000000000 -2.138187000000000 0.326939000000000
H 0.187513000000000 5.166071000000000 -0.798405000000000
S -0.404967000000000 -2.177319000000000 -1.327603000000000
C 2.448726000000000 -0.712896000000000 0.406625000000000
H 3.372891000000000 -3.932977000000000 -0.136729000000000
H -0.385871000000000 -2.230394000000000 1.055913000000000
C 3.432296000000000 -1.797683000000000 0.189397000000000
O 2.922783000000000 0.548836000000000 0.604528000000000
H 0.949230000000000 -4.232679000000000 0.220492000000000
C 2.711746000000000 -3.074425000000000 -0.261157000000000
H 4.173011000000000 -1.478851000000000 -0.548365000000000
H 3.978926000000000 -1.965869000000000 1.124786000000000
C 1.414494000000000 -3.291939000000000 0.517090000000000
H 2.478005000000000 -2.999338000000000 -1.325690000000000
H 1.636774000000000 -3.369585000000000 1.587009000000000
C -1.602161000000000 -3.516418000000000 -1.066770000000000
H -2.186870000000000 -3.599453000000000 -1.981968000000000
H -1.102735000000000 -4.465755000000000 -0.877867000000000
H -2.267932000000000 -3.274814000000000 -0.237767000000000
O 1.036093000000000 3.444430000000000 -0.157130000000000
O 2.170481000000000 2.091092000000000 -1.556274000000000
C 0.752903000000000 4.352775000000000 -1.242608000000000
H -0.806597000000000 1.995284000000000 1.215181000000000
H -1.873840000000000 -0.701043000000000 0.290940000000000
H -2.110939000000000 -0.340859000000000 1.985926000000000
40
R6_SMe6_cyc2R_-1_1 charge=-1 multiplicity=1
wB97MV_TZVPD_SMD_chcl3=-1357.479574755049,
B3LYP_D3BJ_TZVPD=-1357.260151004154,
B3LYP_TZVP_CPCM_chcl3_freq=0.26786463,
B3LYP_TZVPD_CPCM_chcl3=-1357.329781288019,
B3LYP_D3BJ_TZVPD_SMD_chcl3=-1357.332253028291, M062X_TZVPD=-1357.361821995227, COSMORS=-0.012520145630791518,
M062X_TZVPD_CPCM_chcl3=-1357.434029786112,
M062X_TZVPD_SMD_chcl3=-1357.436504317831,
wB97MV_TZVPD_CPCM_chcl3=-1357.476828899681, wB97MV_TZVPD=-1357.405973714118
O -4.429533000000000 -0.293948000000000 -0.514401000000000
C -3.909316000000000 0.058844000000000 0.736036000000000
C 1.048913000000000 2.442614000000000 -0.520235000000000
H -6.352965000000000 -0.871804000000000 0.051203000000000
C -1.665983000000000 -0.296464000000000 -0.315051000000000
C -2.511989000000000 0.050677000000000 0.789446000000000
H -6.200104000000000 0.811458000000000 -0.481130000000000
O -4.701534000000000 0.346570000000000 1.649591000000000
C -5.841734000000000 -0.206972000000000 -0.647947000000000
H -6.064428000000000 -0.509661000000000 -1.670615000000000
H 1.617632000000000 5.560575000000000 0.217214000000000
H -1.027303000000000 2.206174000000000 -0.074757000000000
C -0.264490000000000 0.286332000000000 -0.364322000000000
H 2.138687000000000 4.758021000000000 -1.291126000000000
C -0.132658000000000 1.642156000000000 -0.304755000000000
C 0.674761000000000 -0.786903000000000 -0.447715000000000
C 2.166795000000000 -0.758031000000000 -0.401542000000000
H 2.853439000000000 4.272749000000000 0.260702000000000
S 2.692626000000000 -0.282996000000000 1.314789000000000
C -0.022569000000000 -1.970833000000000 -0.439552000000000
H 2.558894000000000 -4.221043000000000 -0.440918000000000
H 2.545337000000000 0.047667000000000 -1.023385000000000
C 0.592157000000000 -3.328825000000000 -0.492238000000000
O -1.327146000000000 -1.881158000000000 -0.386192000000000
H 3.837731000000000 -2.107873000000000 -0.624905000000000
C 2.082990000000000 -3.282991000000000 -0.148813000000000
H 0.042824000000000 -3.993770000000000 0.178142000000000
H 0.444679000000000 -3.720692000000000 -1.505593000000000
C 2.765959000000000 -2.100667000000000 -0.831171000000000
H 2.202759000000000 -3.184180000000000 0.933044000000000
H 2.650125000000000 -2.189560000000000 -1.917853000000000
C 4.330895000000000 0.411620000000000 0.959558000000000
H 5.001944000000000 -0.342176000000000 0.547724000000000
H 4.234717000000000 1.245313000000000 0.264773000000000
H 4.740030000000000 0.771641000000000 1.903054000000000
O 0.868210000000000 3.702107000000000 -0.018400000000000
O 2.083266000000000 2.148584000000000 -1.103258000000000
C 1.942854000000000 4.622670000000000 -0.226756000000000
H -2.160086000000000 -0.197886000000000 -1.280052000000000
H -2.060160000000000 0.269512000000000 1.747798000000000
49
Sulf_0_1 charge=0 multiplicity=1 B3LYP_D3BJ_TZVPD=-1699.720420948122, B3LYP_D3BJ_TZVPD_SMD_chcl3=-1699.752070544382, B3LYP_TZVP_CPCM_chcl3_freq=0.32147331,
B3LYP_TZVPD_CPCM_chcl3=-1699.742825311801,
M062X_TZVPD_SMD_chcl3=-1699.87400159093, M062X_TZVPD=-1699.871425147514, COSMORS=-0.007458516872547382,
wB97MV_TZVPD_CPCM_chcl3=-1699.949468824199,
wB97MV_TZVPD_SMD_chcl3=-1699.959117421698, wB97MV_TZVPD=-1699.926470650824, M062X_TZVPD_CPCM_chcl3=-1699.864491885423
C -1.227378000000000 -3.029680000000000 -0.716282000000000
C -1.655154000000000 -3.964635000000000 0.214084000000000
C -0.730209000000000 -4.607145000000000 1.032634000000000
C 0.625888000000000 -4.320422000000000 0.921984000000000
C 1.064656000000000 -3.381773000000000 -0.002355000000000
C 0.131001000000000 -2.746007000000000 -0.809145000000000
O 0.013602000000000 -1.824919000000000 -3.258118000000000
S 0.666775000000000 -1.541597000000000 -1.993479000000000
O 2.114974000000000 -1.479426000000000 -1.968835000000000
C 1.833113000000000 -0.388718000000000 1.061173000000000
C 2.383080000000000 -0.940745000000000 2.209952000000000
C 1.567513000000000 -1.271751000000000 3.285049000000000
C 0.198475000000000 -1.033106000000000 3.213008000000000
C -0.351338000000000 -0.460149000000000 2.075410000000000
C 0.460566000000000 -0.150302000000000 0.982319000000000
C 4.109258000000000 5.318146000000000 0.324157000000000
O 3.297794000000000 4.144153000000000 0.130491000000000
O 1.457611000000000 5.352697000000000 0.608711000000000
O -2.929604000000000 -0.416457000000000 -0.046168000000000
C -5.397449000000000 0.266319000000000 -0.773417000000000
O -4.357545000000000 1.242715000000000 -0.571459000000000
C -3.155426000000000 0.762049000000000 -0.213801000000000
C 1.975822000000000 4.299141000000000 0.304381000000000
C -2.199175000000000 1.870102000000000 -0.037670000000000
C -0.029979000000000 -0.001482000000000 -1.472534000000000
C -0.133944000000000 0.463395000000000 -0.227630000000000
C -0.858675000000000 1.753010000000000 -0.022592000000000
C -0.063056000000000 2.953425000000000 0.188127000000000
C 1.267894000000000 3.025074000000000 0.075739000000000
H -1.937865000000000 -2.515529000000000 -1.346133000000000
H -2.709941000000000 -4.188484000000000 0.303405000000000
H -1.069306000000000 -5.333113000000000 1.760528000000000
H 1.342758000000000 -4.819813000000000 1.560194000000000
H 2.111734000000000 -3.136610000000000 -0.094179000000000
H 2.467505000000000 -0.147807000000000 0.220642000000000
H 3.449502000000000 -1.119324000000000 2.259395000000000
H 1.995473000000000 -1.712098000000000 4.176576000000000
H -0.442601000000000 -1.291699000000000 4.045962000000000
H -1.415244000000000 -0.280568000000000 2.015329000000000
H 5.130920000000000 4.998016000000000 0.143027000000000
H 3.997872000000000 5.688777000000000 1.342295000000000
H 3.819850000000000 6.095967000000000 -0.381214000000000
H -5.115867000000000 -0.424004000000000 -1.567405000000000
H -5.575466000000000 -0.288337000000000 0.146907000000000
H -6.278513000000000 0.834901000000000 -1.055030000000000
H -2.639241000000000 2.851664000000000 0.083314000000000
H -0.466488000000000 0.521749000000000 -2.313834000000000
H -0.610564000000000 3.854926000000000 0.440614000000000
H 1.874090000000000 2.171916000000000 -0.190823000000000
38
R5_SMe6H_cyc2R_0_1 charge=0 multiplicity=1
B3LYP_D3BJ_TZVPD_SMD_chcl3=-1318.494309877415,
B3LYP_TZVP_CPCM_chcl3_freq=0.25462548,
B3LYP_TZVPD_CPCM_chcl3=-1318.488453230036,
M062X_TZVPD_SMD_chcl3=-1318.596553843646, M062X_TZVPD=-1318.570819552458, COSMORS=-0.003423996112887573,
wB97MV_TZVPD_CPCM_chcl3=-1318.633742334091,
wB97MV_TZVPD_SMD_chcl3=-1318.639769705737, wB97MV_TZVPD=-1318.614251732478, M062X_TZVPD_CPCM_chcl3=-1318.590711733249
C -2.223468000000000 -0.264614000000000 0.681381000000000
C -1.385618000000000 -0.439528000000000 -0.517371000000000
C -0.020506000000000 0.252569000000000 -0.471184000000000
C 0.077220000000000 1.586392000000000 -0.263072000000000
C 0.937699000000000 -0.785312000000000 -0.666849000000000
C 2.404383000000000 -1.031260000000000 -0.555945000000000
C 0.264750000000000 -1.953183000000000 -0.796899000000000
O -1.058914000000000 -1.895346000000000 -0.735705000000000
C 2.533754000000000 -2.518759000000000 -1.012215000000000
C 1.120831000000000 -3.158912000000000 -0.915756000000000
C -5.518086000000000 0.475635000000000 -0.622475000000000
O -4.226332000000000 -0.155182000000000 -0.535090000000000
O -4.352041000000000 0.123429000000000 1.686311000000000
O 2.366444000000000 1.994541000000000 -0.839207000000000
C 2.265876000000000 4.479321000000000 0.059131000000000
O 1.130984000000000 3.605794000000000 0.139105000000000
C 1.305768000000000 2.352630000000000 -0.358653000000000
C -3.556749000000000 -0.095148000000000 0.619857000000000
H -1.746478000000000 -0.284636000000000 1.652113000000000
H -1.926186000000000 -0.151374000000000 -1.417964000000000
H -0.819779000000000 2.146494000000000 -0.034205000000000
H 3.007746000000000 -0.362563000000000 -1.163332000000000
H 2.865884000000000 -2.531047000000000 -2.050794000000000
H 3.272117000000000 -3.059585000000000 -0.423810000000000
H 0.870715000000000 -3.763342000000000 -1.789728000000000
H 1.000757000000000 -3.798799000000000 -0.036815000000000
H -6.243902000000000 -0.037802000000000 0.005647000000000
H -5.802978000000000 0.391674000000000 -1.667351000000000
H -5.452061000000000 1.525262000000000 -0.335366000000000
H 2.566303000000000 4.625216000000000 -0.978550000000000
H 3.105442000000000 4.070823000000000 0.621291000000000
H 1.942385000000000 5.421161000000000 0.494513000000000
S 3.050322000000000 -0.696455000000000 1.140143000000000
C 2.057405000000000 -1.831335000000000 2.146870000000000
H 2.274781000000000 -2.872904000000000 1.912863000000000
H 2.328688000000000 -1.638732000000000 3.184055000000000
H 0.993242000000000 -1.635809000000000 2.018522000000000
H -3.812963000000000 0.203227000000000 2.485479000000000
44
R5_SMe1_P1A_SMe6SS charge=0 multiplicity=1 B3LYP_D3BJ_TZVPD=-1757.201244535738, B3LYP_D3BJ_TZVPD_SMD_chcl3=-1757.226675719323, B3LYP_TZVP_CPCM_chcl3_freq=0.30130405,
B3LYP_TZVPD_CPCM_chcl3=-1757.220726922723,
M062X_TZVPD_SMD_chcl3=-1757.351090363723, M062X_TZVPD=-1757.3252636659, COSMORS=-0.0038114006223765725,
wB97MV_TZVPD_CPCM_chcl3=-1757.382574290617,
wB97MV_TZVPD_SMD_chcl3=-1757.388724450861, wB97MV_TZVPD=-1757.363038474295, M062X_TZVPD_CPCM_chcl3=-1757.345164116704
C -2.332801000000000 -4.854872000000000 -0.702976000000000
C -4.793558000000000 1.804748000000000 1.142434000000000

C 4.011657000000000 0.980972000000000 2.677998000000000
C -1.461269000000000 2.561933000000000 -1.950855000000000
C 2.695347000000000 1.532058000000000 -1.879433000000000
C 3.547581000000000 1.258709000000000 -0.634862000000000
C -0.373000000000000 -0.181489000000000 0.887854000000000
C 0.101617000000000 -2.136543000000000 -0.598176000000000
C 0.474842000000000 -0.952654000000000 -0.087254000000000
C 1.647837000000000 0.435442000000000 -1.894777000000000
C 2.576483000000000 0.560462000000000 0.330262000000000
C -1.394832000000000 0.718374000000000 0.182019000000000
C -1.171157000000000 -2.824286000000000 -0.341306000000000
C 1.767641000000000 -0.368427000000000 -0.577788000000000
C -2.543096000000000 1.086014000000000 1.092434000000000
O 0.892603000000000 0.180942000000000 -2.798874000000000
O -2.145645000000000 -2.381252000000000 0.234796000000000
O -2.477079000000000 1.155369000000000 2.297650000000000
O -1.139909000000000 -4.067967000000000 -0.862414000000000
O -3.648941000000000 1.359611000000000 0.387873000000000
S 3.333514000000000 -0.379277000000000 1.686682000000000
S -0.612039000000000 2.288376000000000 -0.374571000000000
H -2.123078000000000 -5.803203000000000 -1.189257000000000
H -3.179477000000000 -4.360691000000000 -1.178299000000000
H -2.548534000000000 -5.007457000000000 0.353961000000000
H -5.576132000000000 1.974379000000000 0.408992000000000
H -4.558164000000000 2.726478000000000 1.672852000000000
H -5.095157000000000 1.038261000000000 1.854539000000000
H 4.793463000000000 1.511782000000000 2.137281000000000
H 4.437371000000000 0.534238000000000 3.575215000000000
H 3.218868000000000 1.672355000000000 2.963067000000000
H -2.534331000000000 2.657940000000000 -1.799091000000000
H -1.239558000000000 1.746100000000000 -2.637446000000000
H -1.067671000000000 3.494356000000000 -2.353902000000000
H 3.250013000000000 1.542312000000000 -2.817870000000000
H 2.159783000000000 2.481119000000000 -1.799783000000000
H 3.971649000000000 2.164545000000000 -0.206200000000000
H 4.368603000000000 0.580262000000000 -0.874856000000000
H 0.248527000000000 0.413964000000000 1.554558000000000
H -0.925300000000000 -0.880834000000000 1.508723000000000
H 0.771430000000000 -2.659176000000000 -1.268316000000000
H 1.920405000000000 1.308599000000000 0.774884000000000
H -1.786150000000000 0.226287000000000 -0.704750000000000
H 2.428251000000000 -1.196208000000000 -0.872143000000000
40
R6_SMe4_-1_1 charge=-1 multiplicity=1 wB97MV_TZVPD_SMD_chcl3=-1357.480923297378, B3LYP_D3BJ_TZVPD=-1357.264450872049, B3LYP_TZVP_CPCM_chcl3_freq=0.26619166, B3LYP_TZVPD_CPCM_chcl3=-1357.335033618381, M062X_TZVPD=-1357.36418783457, COSMORS=-0.009323922971385075, M062X_TZVPD_CPCM_chcl3=-1357.437281160099, M062X_TZVPD_SMD_chcl3=-1357.439663164494, wB97MV_TZVPD_CPCM_chcl3=-1357.478298968401, B3LYP_D3BJ_TZVPD_SMD_chcl3=-1357.337316296583, wB97MV_TZVPD=-1357.40636701925
O -3.588115000000000 -1.073067000000000 -0.262737000000000
C -2.675951000000000 -2.120336000000000 -0.355914000000000
C -0.572678000000000 2.432547000000000 -0.242982000000000
H -5.260450000000000 -2.119250000000000 0.417774000000000
C -0.897367000000000 -0.405174000000000 -0.045662000000000
C -1.327731000000000 -1.732035000000000 -0.226202000000000
H -5.517518000000000 -0.496890000000000 -0.276582000000000
O -3.102526000000000 -3.273185000000000 -0.533626000000000
C -4.960712000000000 -1.428017000000000 -0.372731000000000
H -5.179178000000000 -1.890045000000000 -1.337703000000000
H -1.659808000000000 4.403417000000000 -1.682323000000000
H -0.617269000000000 -2.544134000000000 -0.294960000000000
C 0.370973000000000 0.120861000000000 0.021345000000000
H -2.445802000000000 2.857603000000000 -2.081542000000000
C 0.588371000000000 1.589625000000000 0.236766000000000
C 1.586354000000000 -0.711973000000000 0.019067000000000
C 1.697869000000000 -1.812099000000000 0.795009000000000
H -1.242328000000000 3.558918000000000 -3.202238000000000
S 1.072082000000000 2.037576000000000 1.986623000000000
C 2.735277000000000 -0.352244000000000 -0.853067000000000
H 4.685709000000000 -3.154532000000000 -0.269665000000000
H 1.464416000000000 1.911250000000000 -0.322749000000000
C 4.020615000000000 -1.131186000000000 -0.659602000000000
O 2.677673000000000 0.544762000000000 -1.684383000000000
H 2.463721000000000 -3.793110000000000 0.883061000000000
C 3.748937000000000 -2.610969000000000 -0.407743000000000
H 4.650827000000000 -0.966938000000000 -1.533858000000000
H 4.535370000000000 -0.697897000000000 0.207373000000000
C 2.844485000000000 -2.769840000000000 0.808392000000000
H 3.252311000000000 -3.037679000000000 -1.284577000000000
H 3.422909000000000 -2.604263000000000 1.728855000000000
C -0.183776000000000 1.158776000000000 2.951569000000000
H -1.161974000000000 1.618321000000000 2.829749000000000
H -0.226369000000000 0.116474000000000 2.638683000000000
H 0.124648000000000 1.214638000000000 3.995036000000000
O -0.436916000000000 2.718875000000000 -1.549996000000000
O -1.518043000000000 2.805752000000000 0.413253000000000
C -1.524436000000000 3.433846000000000 -2.160586000000000
H -1.706954000000000 0.313083000000000 0.026515000000000
H 0.881994000000000 -2.036045000000000 1.473263000000000
40
R6_SMe6_cyc4SETS_-1_1 charge=-1 multiplicity=1 wB97MV_TZVPD_SMD_chcl3=-1357.441140430451, B3LYP_TZVP_CPCM_chcl3_freq=0.26802337, B3LYP_TZVPD_CPCM_chcl3=-1357.299147278027, M062X_TZVPD=-1357.325537211085, COSMORS=-0.010999610819072353, M062X_TZVPD_CPCM_chcl3=-1357.397021956159, M062X_TZVPD_SMD_chcl3=-1357.399476342991, wB97MV_TZVPD_CPCM_chcl3=-1357.438460609909, B3LYP_D3BJ_TZVPD_SMD_chcl3=-1357.301416028934, wB97MV_TZVPD=-1357.368281989336
O -3.759194000000000 1.363962000000000 -0.301326000000000
C -2.708120000000000 2.215139000000000 -0.566365000000000
C -1.056718000000000 -2.692610000000000 0.195051000000000
H -5.238230000000000 2.256720000000000 -1.470152000000000
C -1.201005000000000 0.303150000000000 -0.064454000000000
C -1.420406000000000 1.624621000000000 -0.422463000000000
H -5.755755000000000 1.110256000000000 -0.204447000000000
O -2.932953000000000 3.382631000000000 -0.900588000000000
C -5.065387000000000 1.915665000000000 -0.448033000000000
H -5.218936000000000 2.755384000000000 0.231903000000000
H -1.908055000000000 -5.435432000000000 1.698752000000000
H 0.655159000000000 -2.057982000000000 1.322543000000000
C 0.001664000000000 -0.369049000000000 0.139017000000000
H -1.965550000000000 -5.180815000000000 -0.070357000000000
C 0.064743000000000 -1.758586000000000 0.463061000000000
C 1.366319000000000 0.041658000000000 -0.208630000000000
C 1.955272000000000 1.413245000000000 -0.197352000000000
H -3.060412000000000 -4.266366000000000 0.990713000000000
S 1.807944000000000 2.220033000000000 1.467650000000000
C 2.025592000000000 -1.036303000000000 -0.731729000000000
H 2.925139000000000 0.847817000000000 -2.700126000000000
H 1.378355000000000 2.103968000000000 -0.817608000000000
C 3.288046000000000 -0.930651000000000 -1.535069000000000
O 1.479356000000000 -2.238301000000000 -0.625245000000000
H 4.064237000000000 1.064285000000000 0.120982000000000
C 3.596411000000000 0.521140000000000 -1.899781000000000
H 4.118774000000000 -1.362527000000000 -0.963879000000000
H 3.175919000000000 -1.553795000000000 -2.427151000000000
C 3.410288000000000 1.421452000000000 -0.679560000000000
H 4.617472000000000 0.607239000000000 -2.280577000000000
H 3.705936000000000 2.448492000000000 -0.900799000000000
C 2.512600000000000 0.945456000000000 2.546423000000000
H 3.561797000000000 0.758410000000000 2.319392000000000
H 2.431842000000000 1.323426000000000 3.565115000000000
H 1.947607000000000 0.018980000000000 2.457331000000000
O -1.054590000000000 -3.708165000000000 1.087695000000000
O -1.857547000000000 -2.620870000000000 -0.712980000000000
C -2.068046000000000 -4.708947000000000 0.906703000000000
H -2.091451000000000 -0.285109000000000 0.119910000000000
H -0.603591000000000 2.304683000000000 -0.608435000000000
37
R5_SMe6_-1_1 charge=-1 multiplicity=1 wB97MV_TZVPD_SMD_chcl3=-1318.186105454589, B3LYP_D3BJ_TZVPD=-1317.978917633166, CCSDT_SMD_chcl3=-1316.272762161452, CCSDT=-1316.203998024729, B3LYP_TZVP_CPCM_chcl3_freq=0.23971397, B3LYP_TZVPD_CPCM_chcl3=-1318.045842461778, M062X_TZVPD=-1318.071678984491, COSMORS=-0.010331652776487761, M062X_TZVPD_CPCM_chcl3=-1318.14072106858, M062X_TZVPD_SMD_chcl3=-1318.141555896621, wB97MV_TZVPD_CPCM_chcl3=-1318.185024639234, B3LYP_D3BJ_TZVPD_SMD_chcl3=-1318.046668618333, wB97MV_TZVPD=-1318.117113152759
C -2.495415000000000 0.523684000000000 -0.252317000000000
C -1.445257000000000 -0.260881000000000 0.026966000000000
C -0.039163000000000 0.081565000000000 -0.259273000000000
C 0.282183000000000 1.428649000000000 -0.116351000000000
C 0.839956000000000 -0.995119000000000 -0.514481000000000
C 2.326559000000000 -0.983548000000000 -0.341653000000000
C 0.387523000000000 -2.318554000000000 -0.803885000000000
O -0.769802000000000 -2.710225000000000 -1.046057000000000
C 2.803129000000000 -2.366850000000000 -0.837366000000000
C 1.574933000000000 -3.274867000000000 -0.774791000000000
C -6.128625000000000 0.795454000000000 0.030095000000000
O -4.755006000000000 1.058933000000000 -0.291753000000000
O -4.184698000000000 -0.850479000000000 0.756856000000000
O 2.299869000000000 1.770167000000000 -1.378818000000000
C 2.557852000000000 4.226393000000000 -0.485616000000000
O 1.496610000000000 3.390029000000000 -0.025618000000000
C 1.445073000000000 2.125176000000000 -0.571661000000000
C -3.855269000000000 0.141595000000000 0.135881000000000
H -2.383896000000000 1.453450000000000 -0.793676000000000
H -1.635884000000000 -1.209366000000000 0.509039000000000
H -0.444898000000000 2.060756000000000 0.373179000000000
H 2.815657000000000 -0.177990000000000 -0.883979000000000
H 3.132283000000000 -2.263357000000000 -1.874609000000000
H 3.649696000000000 -2.736360000000000 -0.259953000000000
H 1.511692000000000 -3.990844000000000 -1.595755000000000
H 1.529167000000000 -3.846106000000000 0.157889000000000
H -6.691687000000000 1.627572000000000 -0.383996000000000
H -6.265270000000000 0.744122000000000 1.110160000000000
H -6.452925000000000 -0.143416000000000 -0.418327000000000
H 2.495868000000000 4.383244000000000 -1.563858000000000
H 3.530127000000000 3.792254000000000 -0.248641000000000
H 2.436125000000000 5.173493000000000 0.035899000000000
S 2.871480000000000 -0.582199000000000 1.395224000000000
C 1.805978000000000 -1.652075000000000 2.399434000000000
H 0.757226000000000 -1.447563000000000 2.188445000000000
H 2.016470000000000 -2.706698000000000 2.225688000000000
H 2.014236000000000 -1.414492000000000 3.442247000000000
44
R5_SMe1_P2A_SMe6RR charge=0 multiplicity=1 B3LYP_D3BJ_TZVPD=-1757.200184155754, B3LYP_D3BJ_TZVPD_SMD_chcl3=-1757.226762229048, B3LYP_TZVP_CPCM_chcl3_freq=0.30129642, B3LYP_TZVPD_CPCM_chcl3=-1757.220098109428, M062X_TZVPD_SMD_chcl3=-1757.352815934506, M062X_TZVPD=-1757.32571613734, COSMORS=-0.0026494261341252195, wB97MV_TZVPD_CPCM_chcl3=-1757.383249665928, wB97MV_TZVPD_SMD_chcl3=-1757.390069826218, M062X_TZVPD_CPCM_chcl3=-1757.34625150882
C -0.830151000000000 4.454364000000000 0.724245000000000
C 5.575832000000000 0.249130000000000 -1.254836000000000
C -1.899176000000000 -3.317485000000000 -0.929028000000000
C 1.886962000000000 -2.904057000000000 0.833635000000000
C -3.711386000000000 0.873669000000000 -1.409626000000000
C -3.914527000000000 -0.558956000000000 -0.898732000000000
C 0.873386000000000 -0.280377000000000 -0.412902000000000
C -0.415298000000000 0.843529000000000 1.410501000000000
C -0.290421000000000 0.151710000000000 0.072134000000000
C -2.213169000000000 1.105025000000000 -1.370473000000000
C -2.717188000000000 -0.779274000000000 0.037948000000000
C 2.212313000000000 -0.141423000000000 0.229664000000000
C -1.113024000000000 2.179442000000000 1.289585000000000
C -1.548877000000000 -0.105361000000000 -0.700974000000000
C 3.342248000000000 -0.333324000000000 -0.757740000000000
O -1.628916000000000 2.066391000000000 -1.810278000000000
O -2.305028000000000 2.346458000000000 1.425022000000000
O 3.314237000000000 -1.098768000000000 -1.694959000000000
O -0.257162000000000 3.155249000000000 0.978845000000000
O 4.396963000000000 0.424561000000000 -0.443259000000000
S -2.409138000000000 -2.479922000000000 0.596184000000000
S 2.494131000000000 -1.373708000000000 1.592760000000000
H -1.344429000000000 4.819885000000000 1.611648000000000
H -1.527523000000000 4.391662000000000 -0.109349000000000
H 0.008984000000000 5.096820000000000 0.475419000000000
H 5.356709000000000 0.488741000000000 -2.294148000000000
H 5.930033000000000 -0.778153000000000 -1.181276000000000
H 6.311114000000000 0.937701000000000 -0.849747000000000
H -0.954361000000000 -2.926515000000000 -1.303923000000000
H -2.665649000000000 -3.248624000000000 -1.699166000000000
H -1.763079000000000 -4.364458000000000 -0.661193000000000
H 2.077051000000000 -3.688180000000000 1.565689000000000
H 2.428571000000000 -3.127462000000000 -0.083567000000000
H 0.817319000000000 -2.840708000000000 0.642506000000000
H -4.157501000000000 1.604956000000000 -0.728992000000000
H -4.107777000000000 1.067844000000000 -2.406406000000000
H -3.875363000000000 -1.264523000000000 -1.730534000000000
H -4.861395000000000 -0.699611000000000 -0.381105000000000
H 0.873724000000000 -0.825438000000000 -1.348747000000000
H 0.562260000000000 0.989358000000000 1.860164000000000
H -1.006622000000000 0.224149000000000 2.086348000000000
H -2.908122000000000 -0.247517000000000 0.971439000000000
H 2.341961000000000 0.814813000000000 0.731988000000000
H -1.293614000000000 -0.737824000000000 -1.560696000000000
44
R5_SMe4_P3_SMe6RS_P3 charge=0 multiplicity=1 B3LYP_D3BJ_TZVPD=-1757.19621016418, B3LYP_D3BJ_TZVPD_SMD_chcl3=-1757.221932135232, B3LYP_TZVP_CPCM_chcl3_freq=0.3001577, B3LYP_TZVPD_CPCM_chcl3=-1757.21562229668, M062X_TZVPD_SMD_chcl3=-1757.347712768911, M062X_TZVPD=-1757.321489618286, COSMORS=-0.004207107795355648, wB97MV_TZVPD_CPCM_chcl3=-1757.378529350603, wB97MV_TZVPD_SMD_chcl3=-1757.385016814159, wB97MV_TZVPD=-1757.358863228867, M062X_TZVPD_CPCM_chcl3=-1757.341482662476
C -5.782351000000000 1.603048000000000 0.295073000000000
C 3.999289000000000 2.406392000000000 1.787760000000000
C 1.698585000000000 -3.223470000000000 -2.921380000000000
C 1.781180000000000 3.315319000000000 -1.616820000000000
C -1.189340000000000 0.117720000000000 -0.468282000000000
C 0.013362000000000 -3.513344000000000 1.136760000000000
C 1.352847000000000 -3.525275000000000 0.399082000000000
C -2.177296000000000 0.820766000000000 0.466039000000000
C 0.223530000000000 0.099500000000000 0.060032000000000
C -0.267359000000000 -2.051204000000000 1.412451000000000
C 1.389578000000000 -2.190993000000000 -0.366740000000000
C -3.584653000000000 0.803948000000000 -0.069457000000000
C 0.733894000000000 -1.201177000000000 0.626498000000000
C 0.996625000000000 1.203035000000000 0.015546000000000
C 2.394022000000000 1.205905000000000 0.540618000000000
O -1.108893000000000 -1.622738000000000 2.168184000000000
O -3.962792000000000 0.193984000000000 -1.043454000000000
O 3.212541000000000 0.336180000000000 0.330745000000000
O -4.397356000000000 1.557091000000000 0.688641000000000
O 2.658876000000000 2.294885000000000 1.271368000000000
S 0.497849000000000 -2.256244000000000 -1.968117000000000
S 0.377972000000000 2.740728000000000 -0.614223000000000
H -6.217885000000000 0.605167000000000 0.327217000000000
H -5.876238000000000 2.010095000000000 -0.710806000000000
H -6.267874000000000 2.253870000000000 1.016427000000000
H 4.216186000000000 1.575527000000000 2.457434000000000
H 4.022663000000000 3.348636000000000 2.326789000000000
H 4.717247000000000 2.413722000000000 0.968807000000000
H 1.308263000000000 -3.293334000000000 -3.935708000000000
H 1.814569000000000 -4.227864000000000 -2.516501000000000
H 2.663130000000000 -2.716253000000000 -2.940671000000000
H 2.033866000000000 2.577748000000000 -2.375972000000000
H 2.645031000000000 3.537994000000000 -0.993802000000000
H 1.441672000000000 4.230733000000000 -2.099385000000000
H -1.194125000000000 0.624344000000000 -1.434759000000000
H -1.535782000000000 -0.900455000000000 -0.637739000000000
H -0.014862000000000 -4.096463000000000 2.056524000000000
H -0.794493000000000 -3.873989000000000 0.492294000000000
H 1.474928000000000 -4.386851000000000 -0.253774000000000
H 2.176864000000000 -3.534182000000000 1.117674000000000
H -1.895792000000000 1.860385000000000 0.640751000000000
H -2.186205000000000 0.336081000000000 1.443599000000000
H 2.401345000000000 -1.865221000000000 -0.591452000000000
H 1.516818000000000 -1.001497000000000 1.365517000000000
38
R5_SMe4_P1SS_0_1 charge=0 multiplicity=1 B3LYP_D3BJ_TZVPD=-1318.518147481745, CCSDT_SMD_chcl3=-1316.779171327885, CCSDT=-1316.755785319292, B3LYP_D3BJ_TZVPD_SMD_chcl3=-1318.542721937289, B3LYP_TZVP_CPCM_chcl3_freq=0.25220969, B3LYP_TZVPD_CPCM_chcl3=-1318.537197139377, M062X_TZVPD_SMD_chcl3=-1318.638169776526, M062X_TZVPD=-1318.61333405198, COSMORS=-0.0011617832560788363, wB97MV_TZVPD_CPCM_chcl3=-1318.680284232852, wB97MV_TZVPD_SMD_chcl3=-1318.685972648496, wB97MV_TZVPD=-1318.661222582273, M062X_TZVPD_CPCM_chcl3=-1318.632708214914
C -2.150548000000000 0.205079000000000 -0.474759000000000
C -1.027634000000000 0.493023000000000 0.178257000000000
C 0.347625000000000 0.341649000000000 -0.408111000000000
C 1.063075000000000 -0.831772000000000 0.300042000000000
C 1.117026000000000 1.624341000000000 -0.278261000000000
C 1.484884000000000 2.443967000000000 -1.270902000000000
C 1.568848000000000 2.188689000000000 1.009724000000000
O 1.371253000000000 1.715064000000000 2.114748000000000
C 2.246844000000000 3.647849000000000 -0.807193000000000
C 2.319981000000000 3.477423000000000 0.716699000000000
C -5.797730000000000 0.092940000000000 -0.169527000000000
O -4.454489000000000 -0.004849000000000 -0.677231000000000
O -3.674931000000000 0.750177000000000 1.295478000000000
O 0.021853000000000 -2.461919000000000 -1.142539000000000
C -0.161058000000000 -4.287555000000000 0.794398000000000
O 0.363354000000000 -2.963884000000000 1.022717000000000
C 0.416788000000000 -2.155891000000000 -0.039638000000000
C -3.471060000000000 0.357013000000000 0.166324000000000
H -2.137336000000000 -0.157016000000000 -1.494829000000000
H -1.083008000000000 0.843154000000000 1.203547000000000
H 1.042022000000000 -0.684153000000000 1.377572000000000
H 1.268685000000000 2.267293000000000 -2.317729000000000
H 3.234447000000000 3.680587000000000 -1.275115000000000
H 1.735502000000000 4.567777000000000 -1.103397000000000
H 3.343037000000000 3.382933000000000 1.085045000000000
H 1.854230000000000 4.298530000000000 1.263822000000000
H -6.440290000000000 -0.236352000000000 -0.980833000000000
H -6.024138000000000 1.122579000000000 0.104627000000000
H -5.920164000000000 -0.550301000000000 0.701036000000000
H 0.460262000000000 -4.816651000000000 0.073144000000000
H -1.183571000000000 -4.228107000000000 0.424951000000000
H -0.130730000000000 -4.781195000000000 1.760948000000000
S 2.852927000000000 -0.963233000000000 -0.064023000000000
C 2.884409000000000 -1.060669000000000 -1.873784000000000
H 2.287562000000000 -1.896524000000000 -2.232197000000000
H 2.542744000000000 -0.130709000000000 -2.324906000000000
H 3.928920000000000 -1.222126000000000 -2.137788000000000
H 0.253379000000000 0.093137000000000 -1.464925000000000
41
R6_SMe6_cyc4P2_0_1 charge=0 multiplicity=1 B3LYP_D3BJ_TZVPD=-1357.843803441431, B3LYP_D3BJ_TZVPD_SMD_chcl3=-1357.867795807236, B3LYP_TZVP_CPCM_chcl3_freq=0.28327624, B3LYP_TZVPD_CPCM_chcl3=-1357.861505924826, M062X_TZVPD_SMD_chcl3=-1357.974988696143, M062X_TZVPD=-1357.950749798274, COSMORS=-0.005583692589213268, wB97MV_TZVPD_CPCM_chcl3=-1358.010599412905, wB97MV_TZVPD_SMD_chcl3=-1358.017094109181, wB97MV_TZVPD=-1357.992997095602, M062X_TZVPD_CPCM_chcl3=-1357.968698812773
O -4.224802000000000 0.687026000000000 -1.227002000000000
C -3.410885000000000 0.551541000000000 -0.168181000000000
C 0.202199000000000 -3.051664000000000 -0.155308000000000
H -6.071411000000000 0.386502000000000 -0.304281000000000
C -1.071049000000000 -0.062830000000000 0.578346000000000
C -2.037115000000000 0.102758000000000 -0.595631000000000

H -5.572762000000000 2.087059000000000 -0.468868000000000
O -3.759946000000000 0.766669000000000 0.969640000000000
C -5.574289000000000 1.109643000000000 -0.949423000000000
H -6.067364000000000 1.160600000000000 -1.915761000000000
H -1.252686000000000 -4.943021000000000 1.029352000000000
H -0.992893000000000 0.878609000000000 1.121987000000000
C 0.289848000000000 -0.462360000000000 0.108505000000000
H -1.742392000000000 -4.802326000000000 -0.675869000000000
C 0.766417000000000 -1.712946000000000 -0.194810000000000
C 1.367907000000000 0.431524000000000 -0.165961000000000
C 1.456428000000000 1.915407000000000 -0.048760000000000
H -2.766275000000000 -4.091216000000000 0.605800000000000
S 1.555576000000000 2.342502000000000 1.744198000000000
C 2.410466000000000 -0.329136000000000 -0.601296000000000
H 4.762920000000000 2.035409000000000 -1.161754000000000
H 0.538016000000000 2.382016000000000 -0.410357000000000
C 3.745535000000000 0.131553000000000 -1.044180000000000
O 2.070878000000000 -1.633214000000000 -0.627492000000000
H 2.845696000000000 3.474395000000000 -0.622124000000000
C 3.924460000000000 1.598212000000000 -0.618453000000000
H 4.530261000000000 -0.499297000000000 -0.620148000000000
H 3.819095000000000 0.036553000000000 -2.133549000000000
C 2.658747000000000 2.426501000000000 -0.858731000000000
H 4.175013000000000 1.635487000000000 0.444379000000000
H 2.389397000000000 2.383685000000000 -1.919799000000000
C 1.136419000000000 4.106593000000000 1.687496000000000
H 1.124160000000000 4.459021000000000 2.718144000000000
H 1.878743000000000 4.674824000000000 1.128607000000000
H 0.149091000000000 4.251127000000000 1.248651000000000
O -1.093015000000000 -3.031545000000000 0.211777000000000
O 0.809955000000000 -4.068706000000000 -0.422251000000000
C -1.748595000000000 -4.309234000000000 0.295345000000000
H -1.466351000000000 -0.805050000000000 1.268547000000000
H -2.145406000000000 -0.837682000000000 -1.140321000000000
H -1.655922000000000 0.830408000000000 -1.316797000000000
44
R5_SMe4_P3_SMe6RR charge=0 multiplicity=1 B3LYP_D3BJ_TZVPD=-1757.197500630693, B3LYP_D3BJ_TZVPD_SMD_chcl3=-1757.224049902726, B3LYP_TZVP_CPCM_chcl3_freq=0.29966253, B3LYP_TZVPD_CPCM_chcl3=-1757.217605462307, M062X_TZVPD_SMD_chcl3=-1757.349613582827, M062X_TZVPD=-1757.322562087382, COSMORS=-0.003173864431236428, wB97MV_TZVPD_CPCM_chcl3=-1757.380575703313, wB97MV_TZVPD_SMD_chcl3=-1757.387230209211, wB97MV_TZVPD=-1757.360300864866, M062X_TZVPD_CPCM_chcl3=-1757.343183478831
C -5.539901000000000 -1.442468000000000 -1.588726000000000
C 0.578398000000000 4.581892000000000 -1.295025000000000
C 3.511696000000000 -1.479526000000000 -2.633353000000000
C 0.543733000000000 1.528841000000000 3.134857000000000
C -1.408557000000000 0.295038000000000 0.309823000000000
C 1.997439000000000 -2.685159000000000 1.719010000000000
C 2.405972000000000 -2.786309000000000 0.245425000000000
C -2.453063000000000 -0.772288000000000 0.330169000000000
C -0.086523000000000 0.119517000000000 0.288453000000000
C 0.727378000000000 -1.857481000000000 1.710706000000000
C 1.916651000000000 -1.457694000000000 -0.350047000000000
C -3.535341000000000 -0.551541000000000 -0.704194000000000
C 0.546461000000000 -1.248048000000000 0.310497000000000
C 0.881182000000000 1.273878000000000 0.313736000000000
C 0.316739000000000 2.651598000000000 0.041804000000000
O -0.050996000000000 -1.734528000000000 2.625525000000000
O -3.595069000000000 0.375236000000000 -1.477490000000000
O -0.576967000000000 3.190446000000000 0.653334000000000
O -4.446308000000000 -1.534253000000000 -0.654481000000000
O 0.976482000000000 3.235222000000000 -0.966962000000000
S 1.762592000000000 -1.392631000000000 -2.157651000000000
S 1.845129000000000 1.343286000000000 1.889297000000000
H -5.164525000000000 -1.454025000000000 -2.611029000000000
H -6.106425000000000 -0.528160000000000 -1.417649000000000
H -6.157733000000000 -2.315223000000000 -1.398998000000000
H -0.467674000000000 4.600837000000000 -1.596628000000000
H 1.220246000000000 4.880346000000000 -2.118362000000000
H 0.725000000000000 5.235143000000000 -0.436115000000000
H 3.949308000000000 -2.436356000000000 -2.353760000000000
H 4.070177000000000 -0.664452000000000 -2.173284000000000
H 3.548449000000000 -1.372043000000000 -3.716361000000000
H 1.057454000000000 1.513390000000000 4.095652000000000
H -0.152316000000000 0.694514000000000 3.082482000000000
H 0.022307000000000 2.474304000000000 3.010054000000000
H -1.794835000000000 1.303567000000000 0.338025000000000
H 2.738828000000000 -2.125453000000000 2.297397000000000
H 1.838001000000000 -3.639395000000000 2.220356000000000
H 1.886744000000000 -3.616096000000000 -0.238757000000000
H 3.476160000000000 -2.929021000000000 0.109625000000000
H -2.947388000000000 -0.791785000000000 1.307857000000000
H -2.057486000000000 -1.777756000000000 0.189357000000000
H 2.610491000000000 -0.671678000000000 -0.050142000000000
H -0.137093000000000 -1.954315000000000 -0.176746000000000
H 1.659327000000000 1.106609000000000 -0.428079000000000
41
R6_SMe6_P1SR_0_1 charge=0 multiplicity=1 B3LYP_D3BJ_TZVPD=-1357.818338145056, B3LYP_D3BJ_TZVPD_SMD_chcl3=-1357.842502148194, B3LYP_TZVP_CPCM_chcl3_freq=0.28111115, B3LYP_TZVPD_CPCM_chcl3=-1357.836870567042, M062X_TZVPD_SMD_chcl3=-1357.939556347681, M062X_TZVPD=-1357.915235248066, COSMORS=-0.003696725062924378, wB97MV_TZVPD_CPCM_chcl3=-1357.977862083549, wB97MV_TZVPD_SMD_chcl3=-1357.983666578811, wB97MV_TZVPD=-1357.959459897093, M062X_TZVPD_CPCM_chcl3=-1357.933975633408
O 4.373364000000000 1.013564000000000 -0.872107000000000
C 3.535534000000000 0.234145000000000 -0.161922000000000
O 3.896406000000000 -0.760816000000000 0.431737000000000
H 5.850473000000000 -0.384952000000000 -1.327617000000000
H 6.162439000000000 0.605584000000000 0.116822000000000
C 2.160762000000000 0.753714000000000 -0.225022000000000
C 1.162322000000000 0.126131000000000 0.407962000000000
C 5.754134000000000 0.609332000000000 -0.893141000000000
H 6.263859000000000 1.345575000000000 -1.507806000000000
H 1.391823000000000 -0.774906000000000 0.956075000000000
H 2.000915000000000 1.633953000000000 -0.832849000000000
O -1.736190000000000 3.829796000000000 0.215064000000000
C -0.238072000000000 0.561400000000000 0.392756000000000
O -2.932620000000000 1.929901000000000 0.129648000000000
C -2.961120000000000 4.580925000000000 0.134986000000000
H -3.603525000000000 4.347241000000000 0.983001000000000
H -2.663026000000000 5.624901000000000 0.157824000000000
H -3.484809000000000 4.352196000000000 -0.792463000000000
C -0.520698000000000 1.873456000000000 0.284203000000000
C -1.305660000000000 -0.499019000000000 0.498242000000000
C -1.855604000000000 2.489346000000000 0.205179000000000
H 0.819502000000000 -0.131275000000000 -2.420839000000000
C -1.519874000000000 -1.289759000000000 -0.818378000000000
C -1.162833000000000 -1.406796000000000 1.713126000000000
O -0.355072000000000 -1.195594000000000 2.594264000000000
C -2.675932000000000 -2.275906000000000 -0.652085000000000
H -3.112920000000000 -2.046920000000000 2.126659000000000
H -1.865466000000000 -3.198555000000000 2.606115000000000
C -2.414248000000000 -3.257828000000000 0.485716000000000
H -3.586272000000000 -1.704329000000000 -0.443126000000000
H -2.830969000000000 -2.809328000000000 -1.590510000000000
C -2.176574000000000 -2.522895000000000 1.809670000000000
H -3.256821000000000 -3.941843000000000 0.597350000000000
H -1.540953000000000 -3.871489000000000 0.247058000000000
H -2.260043000000000 0.006708000000000 0.667395000000000
H -1.757551000000000 -0.572281000000000 -1.602482000000000
S -0.008236000000000 -2.173376000000000 -1.397703000000000
C 0.513616000000000 -1.114914000000000 -2.773293000000000
H 1.368382000000000 -1.609049000000000 -3.233667000000000
H -0.285397000000000 -1.022234000000000 -3.507552000000000
H 0.293022000000000 2.584883000000000 0.263796000000000
44
R5_SMe4_P1R_SMe6RR charge=0 multiplicity=1 B3LYP_D3BJ_TZVPD=-1757.198824146049, B3LYP_D3BJ_TZVPD_SMD_chcl3=-1757.227460082224, B3LYP_TZVP_CPCM_chcl3_freq=0.30105458, B3LYP_TZVPD_CPCM_chcl3=-1757.220706774061, M062X_TZVPD_SMD_chcl3=-1757.351300762879, M062X_TZVPD=-1757.322744698514, COSMORS=-0.001782618504152569, wB97MV_TZVPD_CPCM_chcl3=-1757.383087322769, wB97MV_TZVPD_SMD_chcl3=-1757.389949269199, wB97MV_TZVPD=-1757.361354061169, M062X_TZVPD_CPCM_chcl3=-1757.344726362364
C -6.086215000000000 -0.513991000000000 -0.480584000000000
C 4.662511000000000 -1.581825000000000 0.320920000000000
C 3.322377000000000 2.202667000000000 -0.878615000000000
C -0.118152000000000 -3.466822000000000 0.920780000000000
C -1.294897000000000 -0.322140000000000 -0.353861000000000
C -0.518384000000000 3.310892000000000 1.294803000000000
C 0.437421000000000 3.512889000000000 0.116214000000000
C -2.492756000000000 -0.424490000000000 0.216750000000000
C -0.001930000000000 -0.305875000000000 0.402991000000000
C -0.339655000000000 1.862129000000000 1.695934000000000
C 0.492698000000000 2.129228000000000 -0.551355000000000
C -3.726523000000000 -0.386166000000000 -0.590460000000000
C 0.489339000000000 1.141126000000000 0.627894000000000
C 1.042170000000000 -1.173238000000000 -0.308862000000000
C 2.320997000000000 -1.314998000000000 0.481128000000000
O -0.779910000000000 1.342217000000000 2.693765000000000
O -3.787197000000000 -0.240550000000000 -1.793569000000000
O 2.378586000000000 -1.392885000000000 1.688370000000000
O -4.812507000000000 -0.534936000000000 0.189197000000000
O 3.389833000000000 -1.368125000000000 -0.320393000000000
S 1.791174000000000 1.922138000000000 -1.810448000000000
S 0.439499000000000 -2.864046000000000 -0.696131000000000
H -6.230768000000000 0.439077000000000 -0.987943000000000
H -6.828484000000000 -0.646474000000000 0.301109000000000
H -6.145186000000000 -1.324546000000000 -1.205905000000000
H 4.873116000000000 -0.771511000000000 1.017361000000000
H 5.392764000000000 -1.594872000000000 -0.482438000000000
H 4.659746000000000 -2.532072000000000 0.852891000000000
H 3.438783000000000 1.489794000000000 -0.064292000000000
H 3.377857000000000 3.219834000000000 -0.495843000000000
H 4.130772000000000 2.047326000000000 -1.591820000000000
H 0.685303000000000 -3.425929000000000 1.653401000000000
H -0.405204000000000 -4.505099000000000 0.758692000000000
H -0.983330000000000 -2.908222000000000 1.272989000000000
H -1.242315000000000 -0.213516000000000 -1.432203000000000
H -1.562048000000000 3.419290000000000 0.978734000000000
H -0.361617000000000 3.978196000000000 2.141966000000000
H 1.425963000000000 3.789276000000000 0.486289000000000
H 0.111238000000000 4.281716000000000 -0.581477000000000
H -2.605861000000000 -0.541729000000000 1.286625000000000
H -0.167181000000000 -0.712176000000000 1.400697000000000
H -0.421214000000000 1.991210000000000 -1.133131000000000
H 1.495852000000000 1.102347000000000 1.061105000000000
H 1.278780000000000 -0.761610000000000 -1.288206000000000
44
R5_SMe4_P2_SMe1_P2RR charge=0 multiplicity=1 B3LYP_D3BJ_TZVPD=-1757.19957114711, B3LYP_D3BJ_TZVPD_SMD_chcl3=-1757.22346350403, B3LYP_TZVP_CPCM_chcl3_freq=0.30161445, B3LYP_TZVPD_CPCM_chcl3=-1757.216399599732, M062X_TZVPD_SMD_chcl3=-1757.348892038216, M062X_TZVPD=-1757.324235365226, COSMORS=-0.005438674858388509, wB97MV_TZVPD_CPCM_chcl3=-1757.379267517776, wB97MV_TZVPD_SMD_chcl3=-1757.386481819113, wB97MV_TZVPD=-1757.362043599739, M062X_TZVPD_CPCM_chcl3=-1757.341912318583
C 2.429256000000000 -1.310717000000000 -3.067417000000000
C -4.110006000000000 0.916495000000000 -1.799637000000000
C 4.382593000000000 -0.039016000000000 1.584482000000000
C -2.138275000000000 3.544708000000000 0.812658000000000
C -1.687074000000000 -3.329423000000000 1.736620000000000
C -1.201200000000000 -1.922159000000000 2.126390000000000
C -2.160305000000000 -3.181968000000000 0.285621000000000
C 1.153483000000000 0.454306000000000 1.217766000000000
C -0.534135000000000 -1.407800000000000 0.834920000000000
C -0.237573000000000 0.066297000000000 0.776022000000000
C -1.336355000000000 -2.049775000000000 -0.293040000000000
C 2.098444000000000 0.686611000000000 0.030564000000000
C -1.108196000000000 0.997342000000000 0.340365000000000
C 2.272141000000000 -0.534510000000000 -0.844257000000000
C -2.480683000000000 0.634127000000000 -0.119659000000000
O -1.284641000000000 -1.733457000000000 -1.460592000000000
O 2.436299000000000 -1.659999000000000 -0.425162000000000
O -3.232053000000000 -0.098094000000000 0.488296000000000
O 2.244291000000000 -0.221102000000000 -2.140852000000000
O -2.801914000000000 1.217852000000000 -1.275496000000000
S 3.760676000000000 1.289476000000000 0.516319000000000
S -0.647567000000000 2.703579000000000 0.201348000000000
H 3.396942000000000 -1.782584000000000 -2.904113000000000
H 2.382997000000000 -0.861179000000000 -4.054555000000000
H 1.633962000000000 -2.043782000000000 -2.941757000000000
H -4.189206000000000 1.486424000000000 -2.720328000000000
H -4.878586000000000 1.220755000000000 -1.090669000000000
H -4.194615000000000 -0.150377000000000 -1.999484000000000
H 4.406200000000000 -0.988175000000000 1.053775000000000
H 3.792528000000000 -0.129098000000000 2.494405000000000
H 5.398349000000000 0.255037000000000 1.846831000000000
H -2.983939000000000 3.376734000000000 0.148899000000000
H -1.893237000000000 4.605845000000000 0.819189000000000
H -2.372792000000000 3.220639000000000 1.824677000000000
H -2.470929000000000 -3.692892000000000 2.400067000000000
H -0.854538000000000 -4.035145000000000 1.786751000000000
H -0.508915000000000 -1.933881000000000 2.967617000000000
H -2.044362000000000 -1.280736000000000 2.378820000000000
H -3.202206000000000 -2.849770000000000 0.242198000000000
H -2.074351000000000 -4.080661000000000 -0.325173000000000
H 1.557077000000000 -0.328981000000000 1.857356000000000
H 1.119056000000000 1.383040000000000 1.787326000000000
H 0.427690000000000 -1.930157000000000 0.754814000000000
H 1.724412000000000 1.499427000000000 -0.589623000000000
44
R5_SMe4_P2_SMe1_P2SR charge=0 multiplicity=1 B3LYP_D3BJ_TZVPD=-1757.199203734103, B3LYP_D3BJ_TZVPD_SMD_chcl3=-1757.223533227186, B3LYP_TZVP_CPCM_chcl3_freq=0.30176538, B3LYP_TZVPD_CPCM_chcl3=-1757.216581306144, M062X_TZVPD_SMD_chcl3=-1757.348704491911, M062X_TZVPD=-1757.323649378506, COSMORS=-0.0052443829711120845, wB97MV_TZVPD_CPCM_chcl3=-1757.379186925536, wB97MV_TZVPD_SMD_chcl3=-1757.386276457047, wB97MV_TZVPD=-1757.361407724765, M062X_TZVPD_CPCM_chcl3=-1757.341844899034
C 3.122803000000000 -1.071130000000000 2.510080000000000
C -4.335523000000000 -0.151938000000000 2.091776000000000
C 3.747815000000000 1.387962000000000 -1.948445000000000
C -3.181200000000000 3.037508000000000 -0.323782000000000
C 0.074912000000000 -3.671989000000000 -1.513883000000000
C 0.019427000000000 -2.216151000000000 -2.008390000000000
C 0.421099000000000 -3.546720000000000 -0.025918000000000
C 0.650493000000000 0.730146000000000 -1.234559000000000
C -0.661748000000000 -1.478059000000000 -0.846357000000000
C -0.601895000000000 0.024239000000000 -0.775077000000000
C -0.146095000000000 -2.208874000000000 0.402609000000000
C 1.609236000000000 1.082737000000000 -0.089516000000000
C -1.611138000000000 0.733678000000000 -0.231843000000000
C 2.279953000000000 -0.124762000000000 0.520649000000000
C -2.867847000000000 0.086396000000000 0.259167000000000
O -0.215094000000000 -1.790686000000000 1.534605000000000
O 2.638223000000000 -1.095140000000000 -0.112143000000000
O -3.587039000000000 -0.620965000000000 -0.412601000000000
O 2.459991000000000 0.015816000000000 1.833598000000000
O -3.129567000000000 0.402763000000000 1.530047000000000
S 2.927221000000000 2.261061000000000 -0.585500000000000
S -1.488443000000000 2.480419000000000 0.033096000000000
H 4.113124000000000 -1.230116000000000 2.086154000000000
H 2.528862000000000 -1.978769000000000 2.419848000000000
H 3.194840000000000 -0.763519000000000 3.548818000000000
H -5.202397000000000 0.181214000000000 1.522954000000000
H -4.377066000000000 0.222475000000000 3.109991000000000
H -4.286904000000000 -1.239699000000000 2.084032000000000
H 3.081517000000000 1.264707000000000 -2.799826000000000
H 4.127078000000000 0.421599000000000 -1.622592000000000
H 4.583047000000000 2.024349000000000 -2.238621000000000
H -3.476316000000000 2.748491000000000 -1.330454000000000
H -3.150585000000000 4.123725000000000 -0.252627000000000
H -3.886665000000000 2.651859000000000 0.409623000000000
H 0.800593000000000 -4.268260000000000 -2.065324000000000
H -0.903764000000000 -4.142846000000000 -1.632844000000000
H -0.530916000000000 -2.105782000000000 -2.942135000000000
H 1.033381000000000 -1.848617000000000 -2.162082000000000
H 1.502617000000000 -3.481030000000000 0.122853000000000
H 0.046024000000000 -4.348441000000000 0.610425000000000
H 0.367029000000000 1.667276000000000 -1.714585000000000
H 1.178928000000000 0.124021000000000 -1.964028000000000
H -1.714244000000000 -1.778039000000000 -0.868294000000000
H 1.087967000000000 1.624519000000000 0.697910000000000
41
R6_SMe2_P1_0_1 charge=0 multiplicity=1 B3LYP_D3BJ_TZVPD=-1357.827897400493, B3LYP_D3BJ_TZVPD_SMD_chcl3=-1357.852487321765, B3LYP_TZVP_CPCM_chcl3_freq=0.28022521, B3LYP_TZVPD_CPCM_chcl3=-1357.846625687954, M062X_TZVPD_SMD_chcl3=-1357.950706853304, M062X_TZVPD=-1357.925666079918, COSMORS=-0.0029475730270922987, wB97MV_TZVPD_CPCM_chcl3=-1357.991641523935, wB97MV_TZVPD_SMD_chcl3=-1357.9949951839, wB97MV_TZVPD=-1357.970111574024, M062X_TZVPD_CPCM_chcl3=-1357.944874107822
O 4.184428000000000 -0.810982000000000 -0.919320000000000
C 3.305875000000000 0.194532000000000 -1.013344000000000
O 3.612661000000000 1.325841000000000 -1.310398000000000
H 5.923548000000000 0.269903000000000 -0.526367000000000
H 5.648361000000000 -0.129042000000000 -2.239586000000000
C 1.895919000000000 -0.277044000000000 -0.751148000000000
C 1.062045000000000 0.778576000000000 -0.028392000000000
C 5.558832000000000 -0.489777000000000 -1.215932000000000
H 6.107328000000000 -1.418153000000000 -1.087908000000000
S 1.665175000000000 0.883641000000000 1.707492000000000
H -0.874728000000000 2.457251000000000 -0.613592000000000
O -3.292893000000000 2.534930000000000 -0.687262000000000
C -0.415761000000000 0.487095000000000 -0.059940000000000
O -3.371085000000000 0.315500000000000 -0.324874000000000
C -4.727298000000000 2.549894000000000 -0.791038000000000
H -5.178393000000000 2.200721000000000 0.137156000000000
H -4.994971000000000 3.585693000000000 -0.978670000000000
H -5.053831000000000 1.915548000000000 -1.614356000000000
C -1.262010000000000 1.473996000000000 -0.379767000000000
C -0.856457000000000 -0.892524000000000 0.263526000000000
C -2.725112000000000 1.335097000000000 -0.449293000000000
H 1.249021000000000 1.750050000000000 -0.483124000000000
C -0.981830000000000 -1.323631000000000 1.527657000000000
C -1.172831000000000 -1.787556000000000 -0.874102000000000
O -0.975291000000000 -1.449238000000000 -2.031073000000000
C -1.383051000000000 -2.710458000000000 1.908571000000000
H -1.647113000000000 -3.792757000000000 -1.380129000000000
H -2.861833000000000 -2.953347000000000 -0.414324000000000
C -1.210305000000000 -3.700754000000000 0.761699000000000
H -0.811863000000000 -3.022429000000000 2.786702000000000
H -2.432922000000000 -2.682083000000000 2.231465000000000
C -1.784361000000000 -3.124140000000000 -0.530396000000000
H -0.144725000000000 -3.905302000000000 0.621890000000000
H -1.692349000000000 -4.650425000000000 0.998541000000000
H 1.912263000000000 -1.219814000000000 -0.206317000000000
H 1.441059000000000 -0.478618000000000 -1.725113000000000
H -0.804303000000000 -0.624548000000000 2.335690000000000
C 0.874792000000000 2.425241000000000 2.232692000000000
H 1.192260000000000 2.601888000000000 3.259390000000000
H 1.198518000000000 3.254822000000000 1.605134000000000
H -0.210482000000000 2.335776000000000 2.202181000000000
41
R6_SMe6_P2_0_1 charge=0 multiplicity=1 B3LYP_D3BJ_TZVPD=-1357.825797744675, B3LYP_D3BJ_TZVPD_SMD_chcl3=-1357.850711324951, B3LYP_TZVP_CPCM_chcl3_freq=0.28084367, B3LYP_TZVPD_CPCM_chcl3=-1357.844264783462, M062X_TZVPD_SMD_chcl3=-1357.947700756745, M062X_TZVPD=-1357.922470182499, COSMORS=-0.0023928563026660303, wB97MV_TZVPD_CPCM_chcl3=-1357.984323677291, wB97MV_TZVPD_SMD_chcl3=-1357.990948998302, wB97MV_TZVPD=-1357.965788510483, M062X_TZVPD_CPCM_chcl3=-1357.941320202231
O -4.637969000000000 0.359746000000000 0.004787000000000
C -3.466861000000000 0.967827000000000 -0.258459000000000
O -3.388218000000000 2.065501000000000 -0.772002000000000

H -5.844423000000000 1.276091000000000 -1.427153000000000
H -5.872034000000000 2.029815000000000 0.184487000000000
C -2.335524000000000 0.125399000000000 0.161396000000000
C -1.073906000000000 0.543563000000000 -0.022895000000000
C -5.828125000000000 1.084450000000000 -0.354979000000000
H -6.656802000000000 0.443268000000000 -0.069106000000000
H -0.959601000000000 1.510734000000000 -0.491108000000000
H -2.588523000000000 -0.822252000000000 0.614704000000000
O -1.207661000000000 -3.542792000000000 0.632958000000000
C 0.124215000000000 -0.183475000000000 0.355064000000000
O -0.022170000000000 -2.681536000000000 -1.070332000000000
C -1.540778000000000 -4.684170000000000 -0.183979000000000
H -2.081379000000000 -4.363734000000000 -1.073172000000000
H -0.635442000000000 -5.214215000000000 -0.476028000000000
H -2.168500000000000 -5.313246000000000 0.439958000000000
C -0.087258000000000 -1.509185000000000 1.044109000000000
C 1.358411000000000 0.277386000000000 0.037836000000000
C -0.427821000000000 -2.613788000000000 0.066434000000000
H 0.814632000000000 -1.824102000000000 1.564169000000000
C 1.643573000000000 1.553476000000000 -0.703954000000000
C 2.606446000000000 -0.483915000000000 0.393365000000000
O 2.806018000000000 -0.929608000000000 1.507758000000000
C 2.592451000000000 1.329993000000000 -1.887397000000000
H 3.122874000000000 -1.382906000000000 -1.427181000000000
H 4.493299000000000 -1.132596000000000 -0.333156000000000
C 3.885825000000000 0.631372000000000 -1.483079000000000
H 2.057752000000000 0.719093000000000 -2.620455000000000
H 2.803483000000000 2.292110000000000 -2.357790000000000
C 3.590381000000000 -0.672808000000000 -0.733692000000000
H 4.484773000000000 0.414221000000000 -2.368793000000000
H 4.484410000000000 1.286584000000000 -0.846582000000000
H -0.870484000000000 -1.440044000000000 1.796928000000000
H 0.738122000000000 2.023066000000000 -1.072793000000000
S 2.414614000000000 2.754747000000000 0.470707000000000
C 1.035893000000000 3.014708000000000 1.617814000000000
H 0.764900000000000 2.085325000000000 2.116794000000000
H 1.384103000000000 3.731934000000000 2.359509000000000
H 0.171904000000000 3.424125000000000 1.096150000000000
54
Sulf_SMe4_2_-1_1 charge=-1 multiplicity=1 wB97MV_TZVPD_SMD_chcl3=-2138.173706340415, B3LYP_D3BJ_TZVPD=-2137.869812256513, B3LYP_TZVP_CPCM_chcl3_freq=0.35584232, B3LYP_TZVPD_CPCM_chcl3=-2137.939272538263, M062X_TZVPD=-2138.022112721223, COSMORS=-0.01587120260587855, M062X_TZVPD_CPCM_chcl3=-2138.09405151145, M062X_TZVPD_SMD_chcl3=-2138.100501788172, wB97MV_TZVPD_CPCM_chcl3=-2138.167149248702, B3LYP_D3BJ_TZVPD_SMD_chcl3=-2137.945317995799, wB97MV_TZVPD=-2138.096470503593
C 4.196041000000000 0.642731000000000 0.230083000000000
C 4.881519000000000 0.282959000000000 1.385621000000000
C 4.415046000000000 0.691965000000000 2.629228000000000
C 3.260213000000000 1.467327000000000 2.722608000000000
C 2.572049000000000 1.830023000000000 1.574031000000000
C 3.046536000000000 1.412391000000000 0.333889000000000
O 2.869742000000000 1.292297000000000 -2.260327000000000
O 2.067477000000000 3.347795000000000 -1.143116000000000
S 2.151894000000000 1.889405000000000 -1.139871000000000
C -1.455730000000000 -1.952264000000000 -3.048370000000000
S -2.641737000000000 -2.309994000000000 -1.726865000000000
C 1.479326000000000 -1.495986000000000 0.843715000000000
C 2.458000000000000 -2.465296000000000 1.012556000000000
C 3.138241000000000 -2.969526000000000 -0.092023000000000
C 2.835456000000000 -2.492574000000000 -1.362573000000000
C 1.864127000000000 -1.511394000000000 -1.527940000000000
C 1.174558000000000 -1.004949000000000 -0.427762000000000
C -5.966974000000000 2.549575000000000 0.458121000000000
O -4.724601000000000 1.888814000000000 0.210552000000000
O -3.688682000000000 3.872474000000000 0.494664000000000
O -3.566170000000000 -2.316374000000000 1.041663000000000
C -2.179785000000000 -2.393741000000000 3.308441000000000
O -1.559744000000000 -2.150709000000000 2.033062000000000
C -2.375741000000000 -2.110999000000000 0.970020000000000
C -3.603785000000000 2.669577000000000 0.259744000000000
C -1.607459000000000 -1.757503000000000 -0.287365000000000
C 0.540442000000000 1.321804000000000 -0.948992000000000
C 0.131039000000000 0.055196000000000 -0.584222000000000
C -1.224063000000000 -0.290599000000000 -0.327073000000000
C -2.300930000000000 0.603863000000000 -0.152265000000000
C -2.372580000000000 1.966556000000000 0.011049000000000
H 4.531527000000000 0.315741000000000 -0.743014000000000
H 5.772275000000000 -0.327801000000000 1.312078000000000
H 4.945663000000000 0.403782000000000 3.528024000000000
H 2.894685000000000 1.784041000000000 3.691139000000000
H 1.665984000000000 2.418306000000000 1.635966000000000
H -1.166557000000000 -0.902347000000000 -3.024697000000000
H -1.961368000000000 -2.166451000000000 -3.989050000000000
H -0.569102000000000 -2.578888000000000 -2.962903000000000
H 0.944795000000000 -1.108244000000000 1.699601000000000
H 2.693381000000000 -2.823863000000000 2.006823000000000
H 3.901463000000000 -3.726833000000000 0.037805000000000
H 3.359843000000000 -2.879205000000000 -2.227684000000000
H 1.642993000000000 -1.127885000000000 -2.513256000000000
H -5.982854000000000 2.992320000000000 1.454843000000000
H -6.146879000000000 3.332234000000000 -0.279899000000000
H -6.730464000000000 1.778905000000000 0.379404000000000
H -1.370294000000000 -2.373865000000000 4.032492000000000
H -2.907853000000000 -1.613665000000000 3.527715000000000
H -2.672815000000000 -3.365127000000000 3.312641000000000
H -0.712179000000000 -2.372421000000000 -0.295055000000000
H -0.149048000000000 2.126523000000000 -1.131802000000000
H -3.265425000000000 0.108636000000000 -0.096391000000000
H -1.506558000000000 2.607936000000000 0.029847000000000
41
R6_SMe6_cyc2P2_0_1 charge=0 multiplicity=1 B3LYP_D3BJ_TZVPD=-1357.84205865694, B3LYP_D3BJ_TZVPD_SMD_chcl3=-1357.865481741763, B3LYP_TZVP_CPCM_chcl3_freq=0.28283268, B3LYP_TZVPD_CPCM_chcl3=-1357.858859576407, M062X_TZVPD_SMD_chcl3=-1357.973935054401, M062X_TZVPD=-1357.94968928371, COSMORS=-0.0044836454528284, wB97MV_TZVPD_CPCM_chcl3=-1358.010185730772, wB97MV_TZVPD_SMD_chcl3=-1358.017016569695, wB97MV_TZVPD=-1357.992965321378, M062X_TZVPD_CPCM_chcl3=-1357.967290665615
O -4.100183000000000 -2.420597000000000 1.029235000000000
C -2.848177000000000 -1.946519000000000 1.036984000000000
C -0.719551000000000 2.483901000000000 -1.179547000000000
H -4.584078000000000 -2.175356000000000 3.043146000000000
C -1.050552000000000 -1.039827000000000 -0.463986000000000
C -2.482111000000000 -1.387343000000000 -0.327831000000000
H -5.611336000000000 -3.271263000000000 2.072582000000000
O -2.131699000000000 -1.954520000000000 2.008699000000000
C -4.594661000000000 -2.949046000000000 2.276851000000000
H -3.982759000000000 -3.790886000000000 2.597335000000000
H -1.596184000000000 4.554869000000000 -2.616654000000000
H -1.983977000000000 1.425353000000000 0.140645000000000
C -0.360418000000000 0.124116000000000 -0.367657000000000
H 0.058865000000000 4.880994000000000 -2.047771000000000
C -0.913056000000000 1.473730000000000 -0.068155000000000
C 1.023872000000000 -0.219340000000000 -0.561153000000000
C 2.248740000000000 0.621835000000000 -0.564927000000000
H -1.330619000000000 5.660746000000000 -1.236942000000000
S 2.557376000000000 1.407236000000000 1.089654000000000
C 1.066717000000000 -1.560114000000000 -0.743190000000000
H 4.389223000000000 -2.108789000000000 -1.047198000000000
H 2.096805000000000 1.505975000000000 -1.186086000000000
C 2.249844000000000 -2.416347000000000 -0.989589000000000
O -0.190224000000000 -2.084959000000000 -0.694884000000000
H 4.366140000000000 0.355012000000000 -0.897300000000000
C 3.511639000000000 -1.617024000000000 -0.624411000000000
H 2.189773000000000 -3.335960000000000 -0.402074000000000
H 2.283976000000000 -2.716795000000000 -2.043433000000000
C 3.440191000000000 -0.172153000000000 -1.128320000000000
H 3.638911000000000 -1.621392000000000 0.459436000000000
H 3.339133000000000 -0.183733000000000 -2.219469000000000
C 2.179693000000000 0.097489000000000 2.288572000000000
H 1.204631000000000 -0.341644000000000 2.084326000000000
H 2.941098000000000 -0.678968000000000 2.301118000000000
H 2.153263000000000 0.586608000000000 3.261896000000000
O -1.102000000000000 3.704085000000000 -0.777088000000000
O -0.289473000000000 2.250723000000000 -2.284413000000000
C -0.980501000000000 4.766170000000000 -1.743439000000000
H -0.447448000000000 1.890250000000000 0.828753000000000
H -3.101268000000000 -0.502308000000000 -0.489448000000000
H -2.782896000000000 -2.113193000000000 -1.086596000000000
54
Sulf_SMe1_-1_1 charge=-1 multiplicity=1 wB97MV_TZVPD_SMD_chcl3=-2138.161443228547, B3LYP_D3BJ_TZVPD=-2137.855017340962, B3LYP_TZVP_CPCM_chcl3_freq=0.35566529, B3LYP_TZVPD_CPCM_chcl3=-2137.925852954355, M062X_TZVPD=-2138.00696605881, COSMORS=-0.015100616630710283, M062X_TZVPD_CPCM_chcl3=-2138.081065638702, M062X_TZVPD_SMD_chcl3=-2138.087952162867, wB97MV_TZVPD_CPCM_chcl3=-2138.154425834092, B3LYP_D3BJ_TZVPD_SMD_chcl3=-2137.932244851087, wB97MV_TZVPD=-2138.081735856162
C -1.497051000000000 2.440694000000000 -1.119382000000000
C -1.657417000000000 3.561994000000000 -0.319813000000000
C -2.812436000000000 3.713487000000000 0.444210000000000
C -3.808789000000000 2.745376000000000 0.407692000000000
C -3.653251000000000 1.614800000000000 -0.385824000000000
C -2.499170000000000 1.475335000000000 -1.140977000000000
O -1.923241000000000 0.470578000000000 -3.491384000000000
O -3.429048000000000 -0.823311000000000 -2.001537000000000
S -2.260286000000000 0.026056000000000 -2.147117000000000
C 2.758042000000000 2.406003000000000 1.331431000000000
S 3.726645000000000 0.882254000000000 1.482832000000000
C -0.994110000000000 0.277070000000000 1.916266000000000
C -1.855909000000000 0.529990000000000 2.972826000000000
C -3.081791000000000 -0.125409000000000 3.045959000000000
C -3.437466000000000 -1.031061000000000 2.053392000000000
C -2.583843000000000 -1.266951000000000 0.984160000000000
C -1.357751000000000 -0.608766000000000 0.899082000000000
C 6.094408000000000 0.852165000000000 -2.370601000000000
O 5.238724000000000 0.179870000000000 -1.429817000000000
O 3.662463000000000 1.743269000000000 -1.780260000000000
O 1.019213000000000 -4.023850000000000 2.238114000000000
C -1.289595000000000 -4.488683000000000 1.053892000000000
O -0.433163000000000 -3.448632000000000 0.597395000000000
C 0.702079000000000 -3.212527000000000 1.354985000000000
C 4.042030000000000 0.747015000000000 -1.203906000000000
C 1.407562000000000 -2.047052000000000 0.998260000000000
C -0.790263000000000 -0.702564000000000 -1.525571000000000
C -0.435812000000000 -0.827004000000000 -0.236333000000000
C 1.003423000000000 -1.035749000000000 0.094655000000000
C 1.828347000000000 -0.064841000000000 -0.422492000000000
C 3.271029000000000 0.008616000000000 -0.141845000000000
H -0.599990000000000 2.309220000000000 -1.708832000000000
H -0.879910000000000 4.314020000000000 -0.287818000000000
H -2.931101000000000 4.586474000000000 1.073365000000000
H -4.702437000000000 2.860305000000000 1.007106000000000
H -4.405426000000000 0.840594000000000 -0.409015000000000
H 3.137706000000000 3.039450000000000 0.531622000000000
H 1.710194000000000 2.170240000000000 1.150991000000000
H 2.851693000000000 2.925000000000000 2.284739000000000
H -0.035454000000000 0.774381000000000 1.864318000000000
H -1.567984000000000 1.233279000000000 3.744084000000000
H -3.751286000000000 0.064513000000000 3.875677000000000
H -4.383401000000000 -1.555207000000000 2.108765000000000
H -2.859661000000000 -1.970711000000000 0.214858000000000
H 6.319968000000000 1.859531000000000 -2.021948000000000
H 7.000098000000000 0.254343000000000 -2.419332000000000
H 5.617761000000000 0.903867000000000 -3.348718000000000
H -1.669335000000000 -4.276032000000000 2.055630000000000
H -0.775014000000000 -5.450299000000000 1.069494000000000
H -2.118481000000000 -4.523674000000000 0.348968000000000
H 2.371652000000000 -1.951920000000000 1.477626000000000
H -0.081285000000000 -0.874994000000000 -2.323693000000000
H 1.401836000000000 0.750245000000000 -0.986267000000000
H 3.714627000000000 -0.973759000000000 0.012729000000000
44
R5_SMe1_P1A_SMe6RS charge=0 multiplicity=1 B3LYP_D3BJ_TZVPD=-1757.196905004372, B3LYP_D3BJ_TZVPD_SMD_chcl3=-1757.223558785871, B3LYP_TZVP_CPCM_chcl3_freq=0.30203, B3LYP_TZVPD_CPCM_chcl3=-1757.217327329939, M062X_TZVPD_SMD_chcl3=-1757.349195265285, M062X_TZVPD=-1757.32216966349, COSMORS=-0.0037709072098462745, wB97MV_TZVPD_CPCM_chcl3=-1757.379968192139, wB97MV_TZVPD_SMD_chcl3=-1757.386359486372, wB97MV_TZVPD=-1757.359485872762, M062X_TZVPD_CPCM_chcl3=-1757.343057270272
C -1.476598000000000 -4.459120000000000 -1.436207000000000
C -5.025164000000000 0.331738000000000 -0.316255000000000
C 2.039467000000000 2.760931000000000 -2.018663000000000
C -1.636109000000000 3.014734000000000 -1.090103000000000
C 4.383936000000000 0.044331000000000 0.396336000000000
C 3.778300000000000 1.445965000000000 0.476712000000000
C -0.524812000000000 -0.100948000000000 1.436078000000000
C 0.605757000000000 -1.836425000000000 0.015677000000000
C 0.678303000000000 -0.745476000000000 0.790632000000000
C 3.291403000000000 -0.887228000000000 0.881677000000000
C 2.266836000000000 1.236813000000000 0.284386000000000
C -1.422719000000000 0.653469000000000 0.435135000000000
C -0.631736000000000 -2.551252000000000 -0.320980000000000
C 1.999309000000000 -0.078822000000000 1.053843000000000
C -2.889988000000000 0.396710000000000 0.690291000000000
O 3.428318000000000 -2.059434000000000 1.138011000000000
O -1.767473000000000 -2.235562000000000 -0.023314000000000
O -3.379955000000000 0.163970000000000 1.770570000000000
O -0.351342000000000 -3.657179000000000 -1.038445000000000
O -3.600578000000000 0.503342000000000 -0.440708000000000
S 1.775702000000000 1.053961000000000 -1.471611000000000
S -1.135513000000000 2.465148000000000 0.562455000000000
H -1.058451000000000 -5.287346000000000 -2.000939000000000
H -2.156506000000000 -3.877142000000000 -2.057585000000000
H -2.009976000000000 -4.825271000000000 -0.559679000000000
H -5.439760000000000 1.087179000000000 0.349876000000000
H -5.250049000000000 -0.660986000000000 0.070684000000000
H -5.420783000000000 0.448955000000000 -1.320816000000000
H 1.694441000000000 2.816211000000000 -3.050068000000000
H 3.091335000000000 3.039336000000000 -1.981481000000000
H 1.451272000000000 3.443824000000000 -1.405841000000000
H -2.699302000000000 2.855334000000000 -1.254449000000000
H -1.053633000000000 2.488754000000000 -1.845617000000000
H -1.416641000000000 4.080698000000000 -1.137770000000000
H 5.296844000000000 -0.095709000000000 0.974349000000000
H 4.603001000000000 -0.236576000000000 -0.637850000000000
H 4.200905000000000 2.137458000000000 -0.248832000000000
H 3.941950000000000 1.870836000000000 1.470789000000000
H -0.194818000000000 0.576692000000000 2.223768000000000
H -1.131285000000000 -0.869593000000000 1.909792000000000
H 1.503962000000000 -2.265421000000000 -0.401371000000000
H 1.675342000000000 2.056540000000000 0.684795000000000
H -1.183157000000000 0.375222000000000 -0.586768000000000
H 2.021078000000000 0.206037000000000 2.115922000000000
44
R5_SMe4_P1R_SMe6RS charge=0 multiplicity=1 B3LYP_D3BJ_TZVPD=-1757.198533933216, B3LYP_D3BJ_TZVPD_SMD_chcl3=-1757.224101329903, B3LYP_TZVP_CPCM_chcl3_freq=0.30158676, B3LYP_TZVPD_CPCM_chcl3=-1757.217939016791, M062X_TZVPD_SMD_chcl3=-1757.348389182499, M062X_TZVPD=-1757.322775794276, COSMORS=-0.0040326403099634, wB97MV_TZVPD_CPCM_chcl3=-1757.38069485928, wB97MV_TZVPD_SMD_chcl3=-1757.386990472683, wB97MV_TZVPD=-1757.361380167238, M062X_TZVPD_CPCM_chcl3=-1757.342353851525
C -5.559575000000000 1.605674000000000 -0.440101000000000
C 5.130176000000000 0.845197000000000 0.268682000000000
C 1.149700000000000 -2.433218000000000 -2.689828000000000
C 0.776992000000000 2.822507000000000 -1.585447000000000
C -0.986449000000000 0.486831000000000 0.473636000000000
C -0.774161000000000 -2.543652000000000 2.126150000000000
C -1.279808000000000 -2.803851000000000 0.703762000000000
C -2.028556000000000 0.645458000000000 -0.337027000000000
C 0.372374000000000 0.045582000000000 0.024519000000000
C 0.394486000000000 -1.588055000000000 1.979850000000000
C -0.043595000000000 -2.553276000000000 -0.178954000000000
C -3.342969000000000 1.061954000000000 0.183806000000000
C 0.726226000000000 -1.396564000000000 0.498094000000000
C 1.443822000000000 1.026484000000000 0.522750000000000
C 2.798426000000000 0.749704000000000 -0.088666000000000
O 0.984594000000000 -1.056296000000000 2.892500000000000
O -3.618781000000000 1.257427000000000 1.349170000000000
O 2.972634000000000 0.388751000000000 -1.232400000000000
O -4.230577000000000 1.202171000000000 -0.817292000000000
O 3.787874000000000 0.983686000000000 0.776680000000000
S -0.495949000000000 -2.365459000000000 -1.926148000000000
S 1.050488000000000 2.790381000000000 0.206678000000000
H -5.534905000000000 2.580027000000000 0.046389000000000
H -5.999645000000000 0.872964000000000 0.235205000000000
H -6.122564000000000 1.656610000000000 -1.367482000000000
H 5.297108000000000 -0.172488000000000 -0.080754000000000
H 5.781216000000000 1.074173000000000 1.106790000000000
H 5.294599000000000 1.546134000000000 -0.548509000000000
H 1.650878000000000 -3.365545000000000 -2.429630000000000
H 1.759095000000000 -1.583506000000000 -2.385260000000000
H 0.996967000000000 -2.397057000000000 -3.767578000000000
H 0.658051000000000 3.874696000000000 -1.841157000000000
H -0.128681000000000 2.284383000000000 -1.859221000000000
H 1.634509000000000 2.417256000000000 -2.118462000000000
H -1.121661000000000 0.679394000000000 1.533898000000000
H -0.386396000000000 -3.456085000000000 2.590319000000000
H -1.519769000000000 -2.127656000000000 2.804501000000000
H -2.074070000000000 -2.102223000000000 0.451021000000000
H -1.666503000000000 -3.812003000000000 0.567122000000000
H -1.952166000000000 0.466980000000000 -1.401131000000000
H 0.395677000000000 0.026736000000000 -1.063159000000000
H 0.599030000000000 -3.435223000000000 -0.123802000000000
H 1.799765000000000 -1.529567000000000 0.364986000000000
H 1.528000000000000 0.981252000000000 1.607460000000000
55
Sulf_SMe6_P2_0_1 charge=0 multiplicity=1 B3LYP_D3BJ_TZVPD=-2138.41645047987, B3LYP_D3BJ_TZVPD_SMD_chcl3=-2138.452041184757, B3LYP_TZVP_CPCM_chcl3_freq=0.37054321, B3LYP_TZVPD_CPCM_chcl3=-2138.44130900727, M062X_TZVPD_SMD_chcl3=-2138.602543549269, M062X_TZVPD=-2138.565632100817, COSMORS=-0.007409943900728276, wB97MV_TZVPD_CPCM_chcl3=-2138.666237369728, wB97MV_TZVPD_SMD_chcl3=-2138.677335313615, wB97MV_TZVPD=-2138.640907599707, M062X_TZVPD_CPCM_chcl3=-2138.591613362186
C -1.701322000000000 2.696382000000000 -0.908652000000000
C -2.964657000000000 2.362332000000000 -1.376853000000000
C -3.112667000000000 1.346466000000000 -2.313795000000000
C -1.997589000000000 0.672684000000000 -2.806475000000000
C -0.729533000000000 1.008354000000000 -2.358707000000000
C -0.596666000000000 2.014709000000000 -1.405993000000000
O 1.974053000000000 2.172041000000000 -1.845031000000000
O 0.961437000000000 3.730535000000000 -0.184464000000000
S 1.014281000000000 2.413366000000000 -0.787396000000000
C 2.538350000000000 2.912206000000000 2.489953000000000
S 2.949884000000000 1.570681000000000 1.336555000000000
C 2.779631000000000 -2.044198000000000 0.697486000000000
C 3.926554000000000 -2.772416000000000 0.414320000000000
C 4.698640000000000 -2.453142000000000 -0.698055000000000
C 4.316391000000000 -1.399001000000000 -1.519240000000000
C 3.172016000000000 -0.663216000000000 -1.233812000000000
C 2.386764000000000 -0.983290000000000 -0.125129000000000
C -5.986020000000000 0.403192000000000 1.108683000000000
O -4.807842000000000 -0.300012000000000 0.676443000000000
O -3.542970000000000 1.162480000000000 1.830987000000000
O -0.323071000000000 -3.168445000000000 1.921646000000000
C -1.549747000000000 -5.382871000000000 1.087222000000000
O -1.219782000000000 -4.296472000000000 0.197760000000000
C -0.594509000000000 -3.247594000000000 0.746989000000000
C -3.631081000000000 0.192502000000000 1.106144000000000
C -0.245666000000000 -2.215146000000000 -0.302036000000000
C 1.319789000000000 1.221123000000000 0.618616000000000
C 1.158450000000000 -0.224882000000000 0.233041000000000
C -0.055940000000000 -0.828949000000000 0.254043000000000

C -1.249687000000000 -0.169547000000000 0.750350000000000
C -2.508884000000000 -0.587717000000000 0.566341000000000
H -1.570702000000000 3.473266000000000 -0.169190000000000
H -3.832137000000000 2.885517000000000 -0.998018000000000
H -4.100962000000000 1.077247000000000 -2.663256000000000
H -2.117127000000000 -0.113616000000000 -3.539959000000000
H 0.145077000000000 0.497512000000000 -2.734949000000000
H 3.448623000000000 3.092275000000000 3.060840000000000
H 1.745312000000000 2.597676000000000 3.167216000000000
H 2.251848000000000 3.813095000000000 1.956098000000000
H 2.181335000000000 -2.289655000000000 1.564833000000000
H 4.218481000000000 -3.586978000000000 1.065018000000000
H 5.593188000000000 -3.020785000000000 -0.920878000000000
H 4.910914000000000 -1.144155000000000 -2.387468000000000
H 2.887377000000000 0.159126000000000 -1.871807000000000
H -6.822526000000000 -0.129377000000000 0.665730000000000
H -5.959451000000000 1.434000000000000 0.757131000000000
H -6.057141000000000 0.391623000000000 2.195558000000000
H -2.033065000000000 -6.130055000000000 0.464960000000000
H -2.226792000000000 -5.036762000000000 1.866696000000000
H -0.645222000000000 -5.786646000000000 1.539566000000000
H 0.658128000000000 -2.566048000000000 -0.798987000000000
H -1.028890000000000 -2.209164000000000 -1.061549000000000
H 0.568357000000000 1.530200000000000 1.339701000000000
H -1.136914000000000 0.737923000000000 1.326875000000000
H -2.765868000000000 -1.463779000000000 -0.011029000000000
44
R5_SMe4_P1R_SMe6SR charge=0 multiplicity=1 B3LYP_D3BJ_TZVPD=-1757.194796036976, B3LYP_D3BJ_TZVPD_SMD_chcl3=-1757.222327354141, B3LYP_TZVP_CPCM_chcl3_freq=0.30085987, B3LYP_TZVPD_CPCM_chcl3=-1757.216168813773, M062X_TZVPD_SMD_chcl3=-1757.347503671924, M062X_TZVPD=-1757.319724722995, COSMORS=-0.0025821761534570342, wB97MV_TZVPD_CPCM_chcl3=-1757.379092709191, wB97MV_TZVPD_SMD_chcl3=-1757.385409886357, wB97MV_TZVPD=-1757.357713480885, M062X_TZVPD_CPCM_chcl3=-1757.341440056061
C -5.705465000000000 -1.572336000000000 -0.108533000000000
C 5.098653000000000 -0.734101000000000 0.094641000000000
C 0.650862000000000 3.294608000000000 -2.737418000000000
C 0.769155000000000 -3.526787000000000 0.700580000000000
C -1.019843000000000 -0.564171000000000 -0.192120000000000
C -0.388479000000000 3.275700000000000 1.485973000000000
C 0.822415000000000 3.482252000000000 0.572923000000000
C -2.153708000000000 -0.871203000000000 0.433654000000000
C 0.294661000000000 -0.361105000000000 0.502626000000000
C -0.405185000000000 1.788250000000000 1.770996000000000
C 0.947757000000000 2.162072000000000 -0.215015000000000
C -3.407991000000000 -1.048894000000000 -0.319409000000000
C 0.610334000000000 1.109077000000000 0.857360000000000
C 1.431820000000000 -0.958816000000000 -0.334881000000000
C 2.762753000000000 -0.937301000000000 0.378579000000000
O -1.072726000000000 1.227983000000000 2.608496000000000
O -3.544051000000000 -0.931672000000000 -1.519658000000000
O 2.909266000000000 -1.152101000000000 1.561524000000000
O -4.421168000000000 -1.369464000000000 0.507123000000000
O 3.769687000000000 -0.680810000000000 -0.462656000000000
S -0.183557000000000 2.101073000000000 -1.657423000000000
S 1.124312000000000 -2.689094000000000 -0.867637000000000
H -6.382253000000000 -1.820377000000000 0.703980000000000
H -5.655685000000000 -2.389010000000000 -0.827806000000000
H -6.030927000000000 -0.663505000000000 -0.613390000000000
H 5.302921000000000 -1.732746000000000 0.478082000000000
H 5.200062000000000 -0.004987000000000 0.896960000000000
H 5.766731000000000 -0.495590000000000 -0.727293000000000
H 0.631853000000000 4.298515000000000 -2.316252000000000
H 0.103906000000000 3.294261000000000 -3.679133000000000
H 1.680091000000000 2.985397000000000 -2.919350000000000
H -0.161127000000000 -3.167348000000000 1.136815000000000
H 1.590639000000000 -3.408671000000000 1.404141000000000
H 0.660510000000000 -4.580483000000000 0.446314000000000
H -1.038758000000000 -0.455449000000000 -1.268731000000000
H -1.321052000000000 3.512943000000000 0.965387000000000
H -0.371228000000000 3.851186000000000 2.410829000000000
H 1.726701000000000 3.614558000000000 1.172976000000000
H 0.727651000000000 4.350305000000000 -0.075151000000000
H -2.198586000000000 -0.996275000000000 1.506778000000000
H 0.259775000000000 -0.879195000000000 1.461025000000000
H 1.954723000000000 2.019798000000000 -0.605145000000000
H 1.505528000000000 1.074352000000000 1.494444000000000
H 1.521799000000000 -0.426036000000000 -1.280993000000000
44
R5_SMe1_P2A_SMe6SS charge=0 multiplicity=1 B3LYP_D3BJ_TZVPD=-1757.1985749465, B3LYP_D3BJ_TZVPD_SMD_chcl3=-1757.226373083096, B3LYP_TZVP_CPCM_chcl3_freq=0.30096991, B3LYP_TZVPD_CPCM_chcl3=-1757.219549668377, M062X_TZVPD_SMD_chcl3=-1757.351734268937, M062X_TZVPD=-1757.323491555781, COSMORS=-0.002743273322787528, wB97MV_TZVPD_CPCM_chcl3=-1757.382312934415, wB97MV_TZVPD_SMD_chcl3=-1757.389282093616, wB97MV_TZVPD=-1757.361172450351, M062X_TZVPD_CPCM_chcl3=-1757.345029491746
C -2.211872000000000 -1.590841000000000 3.564366000000000
C 5.171787000000000 1.153949000000000 1.327052000000000
C -1.840314000000000 3.188290000000000 0.606578000000000
C 2.712727000000000 -0.757235000000000 -2.875491000000000
C -3.652699000000000 0.082904000000000 -2.279157000000000
C -3.886548000000000 1.008584000000000 -1.078056000000000
C 0.857900000000000 0.228804000000000 -0.586516000000000
C -0.596705000000000 -1.635824000000000 0.223633000000000
C -0.356812000000000 -0.300456000000000 -0.449074000000000
C -2.167310000000000 -0.232122000000000 -2.258831000000000
C -2.773129000000000 0.608096000000000 -0.099045000000000
C 2.134010000000000 -0.382444000000000 -0.112970000000000
C -0.728964000000000 -1.491922000000000 1.727151000000000
C -1.548462000000000 0.408837000000000 -1.009998000000000
C 3.220300000000000 0.647836000000000 0.097968000000000
O -1.568464000000000 -0.881759000000000 -3.082035000000000
O 0.176135000000000 -1.177439000000000 2.464283000000000
O 3.358813000000000 1.649733000000000 -0.567735000000000
O -1.970981000000000 -1.754258000000000 2.151700000000000
O 4.036901000000000 0.296221000000000 1.095439000000000
S -2.521536000000000 1.678152000000000 1.344796000000000
S 2.815101000000000 -1.641646000000000 -1.295715000000000
H -1.575019000000000 -2.264601000000000 4.135141000000000
H -3.259313000000000 -1.837310000000000 3.709529000000000
H -2.015258000000000 -0.560476000000000 3.856321000000000
H 5.704910000000000 0.709245000000000 2.161898000000000
H 4.839013000000000 2.160358000000000 1.576373000000000
H 5.804298000000000 1.185520000000000 0.440855000000000
H -2.502117000000000 3.592725000000000 -0.157614000000000
H -0.848225000000000 3.015387000000000 0.192529000000000
H -1.761276000000000 3.908070000000000 1.420190000000000
H 1.676945000000000 -0.543116000000000 -3.131896000000000
H 3.135901000000000 -1.432826000000000 -3.617910000000000
H 3.296698000000000 0.160827000000000 -2.847728000000000
H -3.946793000000000 0.497023000000000 -3.243974000000000
H -4.181277000000000 -0.868151000000000 -2.158781000000000
H -4.873977000000000 0.896306000000000 -0.634610000000000
H -3.765600000000000 2.051693000000000 -1.375575000000000
H 0.961193000000000 1.175460000000000 -1.102100000000000
H 0.247599000000000 -2.299853000000000 0.036997000000000
H -1.489851000000000 -2.112359000000000 -0.175674000000000
H -3.039905000000000 -0.347064000000000 0.357186000000000
H 2.000170000000000 -0.947474000000000 0.805459000000000
H -1.232258000000000 1.397759000000000 -1.364720000000000
44
R5_SMe4_P3_SMe6RSS_P2 charge=0 multiplicity=1 B3LYP_D3BJ_TZVPD=-1757.200839868693, B3LYP_D3BJ_TZVPD_SMD_chcl3=-1757.227856426891, B3LYP_TZVP_CPCM_chcl3_freq=0.30136999, B3LYP_TZVPD_CPCM_chcl3=-1757.221229081757, M062X_TZVPD_SMD_chcl3=-1757.352085149731, M062X_TZVPD=-1757.325122054904, COSMORS=-0.003226485150707126, wB97MV_TZVPD_CPCM_chcl3=-1757.384020042048, wB97MV_TZVPD_SMD_chcl3=-1757.390767571622, wB97MV_TZVPD=-1757.363790203279, M062X_TZVPD_CPCM_chcl3=-1757.345611266986
C -5.911758000000000 0.811450000000000 -0.533023000000000
C 4.835843000000000 1.534101000000000 0.362248000000000
C 2.043193000000000 -2.688136000000000 -2.002885000000000
C 0.352447000000000 2.921272000000000 -1.761993000000000
C -1.217758000000000 0.547312000000000 0.436328000000000
C 0.172641000000000 -2.881031000000000 2.183596000000000
C 0.262643000000000 -3.505410000000000 0.786150000000000
C -2.271725000000000 0.453828000000000 -0.370670000000000
C 0.185477000000000 0.255154000000000 0.005402000000000
C 0.525149000000000 -1.418483000000000 1.989379000000000
C -0.074594000000000 -2.333909000000000 -0.147754000000000
C -3.638831000000000 0.713718000000000 0.117399000000000
C 0.639495000000000 -1.134698000000000 0.493727000000000
C 1.143209000000000 1.355952000000000 0.478638000000000
C 2.541029000000000 1.176391000000000 -0.065588000000000
O 0.684657000000000 -0.609890000000000 2.875382000000000
O -3.946849000000000 1.008983000000000 1.252905000000000
O 2.797021000000000 0.754188000000000 -1.172670000000000
O -4.533874000000000 0.580127000000000 -0.878281000000000
O 3.466882000000000 1.568808000000000 0.813238000000000
S 0.232842000000000 -2.591590000000000 -1.917958000000000
S 0.592323000000000 3.049336000000000 0.030750000000000
H -6.044440000000000 1.829743000000000 -0.169521000000000
H -6.232992000000000 0.107194000000000 0.233420000000000
H -6.471614000000000 0.658012000000000 -1.450963000000000
H 5.120750000000000 0.515625000000000 0.102616000000000
H 5.425228000000000 1.894529000000000 1.199789000000000
H 4.961163000000000 2.182801000000000 -0.503479000000000
H 2.430276000000000 -3.482678000000000 -1.366891000000000
H 2.503926000000000 -1.735899000000000 -1.746022000000000
H 2.277200000000000 -2.923649000000000 -3.040396000000000
H -0.484631000000000 2.268490000000000 -2.002436000000000
H 1.258431000000000 2.573549000000000 -2.254151000000000
H 0.124896000000000 3.932283000000000 -2.097572000000000
H -1.374316000000000 0.816769000000000 1.475525000000000
H 0.809235000000000 -3.339778000000000 2.940137000000000
H -0.855126000000000 -2.906618000000000 2.561466000000000
H -0.415629000000000 -4.344439000000000 0.644089000000000
H 1.278742000000000 -3.855178000000000 0.595136000000000
H -2.164839000000000 0.191000000000000 -1.415668000000000
H 0.221672000000000 0.211939000000000 -1.083591000000000
H -1.153015000000000 -2.159084000000000 -0.115215000000000
H 1.710564000000000 -1.239205000000000 0.281496000000000
H 1.182831000000000 1.381695000000000 1.565341000000000
44
R5_SMe4_P3_SMe6RSR_P2 charge=0 multiplicity=1 B3LYP_D3BJ_TZVPD=-1757.194790797688, B3LYP_D3BJ_TZVPD_SMD_chcl3=-1757.222317206151, B3LYP_TZVP_CPCM_chcl3_freq=0.30086496, B3LYP_TZVPD_CPCM_chcl3=-1757.216156456642, M062X_TZVPD_SMD_chcl3=-1757.347504693576, M062X_TZVPD=-1757.319731694814, COSMORS=-0.002565347722275612, wB97MV_TZVPD_CPCM_chcl3=-1757.379092537781, wB97MV_TZVPD_SMD_chcl3=-1757.385410833069, wB97MV_TZVPD=-1757.35772007665, M062X_TZVPD_CPCM_chcl3=-1757.34144025156
C -5.694545000000000 -1.624075000000000 -0.093084000000000
C 5.102168000000000 -0.684856000000000 0.084393000000000
C 0.611435000000000 3.289825000000000 -2.749631000000000
C 0.800940000000000 -3.519653000000000 0.712881000000000
C -1.017813000000000 -0.576284000000000 -0.189314000000000
C -0.417056000000000 3.274533000000000 1.474400000000000
C 0.790688000000000 3.488443000000000 0.558856000000000
C -2.148225000000000 -0.889226000000000 0.439771000000000
C 0.296139000000000 -0.359573000000000 0.502376000000000
C -0.419994000000000 1.788092000000000 1.765013000000000
C 0.925826000000000 2.166754000000000 -0.225022000000000
C -3.402300000000000 -1.080518000000000 -0.310211000000000
C 0.599700000000000 1.114535000000000 0.851678000000000
C 1.436814000000000 -0.950124000000000 -0.335435000000000
C 2.769250000000000 -0.913006000000000 0.374651000000000
O -1.080282000000000 1.225110000000000 2.606407000000000
O -3.541643000000000 -0.968817000000000 -1.510664000000000
O 2.920795000000000 -1.123277000000000 1.557807000000000
O -4.410956000000000 -1.407449000000000 0.519246000000000
O 3.771276000000000 -0.647695000000000 -0.469559000000000
S -0.208230000000000 2.090791000000000 -1.664504000000000
S 1.144502000000000 -2.685635000000000 -0.859822000000000
H -6.367048000000000 -1.877297000000000 0.721378000000000
H -5.638466000000000 -2.441401000000000 -0.811142000000000
H -6.030083000000000 -0.719364000000000 -0.598784000000000
H 5.197751000000000 0.048159000000000 0.883879000000000
H 5.765537000000000 -0.442153000000000 -0.740115000000000
H 5.318082000000000 -1.679823000000000 0.470979000000000
H 0.582743000000000 4.294783000000000 -2.331546000000000
H 0.062590000000000 3.280685000000000 -3.690213000000000
H 1.643484000000000 2.990944000000000 -2.932855000000000
H 1.622444000000000 -3.389741000000000 1.414287000000000
H 0.702374000000000 -4.575544000000000 0.463683000000000
H -0.132123000000000 -3.167483000000000 1.149107000000000
H -1.039380000000000 -0.472946000000000 -1.266428000000000
H -1.352183000000000 3.500755000000000 0.953430000000000
H -0.404389000000000 3.854224000000000 2.396662000000000
H 1.694721000000000 3.629807000000000 1.157228000000000
H 0.687806000000000 4.353698000000000 -0.091710000000000
H -2.190445000000000 -1.009427000000000 1.513556000000000
H 0.267382000000000 -0.874712000000000 1.462563000000000
H 1.933226000000000 2.031830000000000 -0.616615000000000
H 1.496409000000000 1.089821000000000 1.487130000000000
H 1.519167000000000 -0.420425000000000 -1.283941000000000
55
Sulf_SMe4_P1SR_0_1 charge=0 multiplicity=1 B3LYP_D3BJ_TZVPD=-2138.402443080955, wB97MV_TZVPD=-2138.630781037335, B3LYP_D3BJ_TZVPD_SMD_chcl3=-2138.438938964919, B3LYP_TZVP_CPCM_chcl3_freq=0.36951442, B3LYP_TZVPD_CPCM_chcl3=-2138.428306181563, M062X_TZVPD_SMD_chcl3=-2138.591536137749, M062X_TZVPD=-2138.553927991205, COSMORS=-0.005406149453046384, wB97MV_TZVPD_CPCM_chcl3=-2138.656871337813, wB97MV_TZVPD_SMD_chcl3=-2138.667902845041, M062X_TZVPD_CPCM_chcl3=-2138.580675138477
C -3.655098000000000 1.626058000000000 0.166952000000000
C -3.800566000000000 2.939348000000000 0.594248000000000
C -3.190386000000000 3.971947000000000 -0.107674000000000
C -2.437529000000000 3.700405000000000 -1.247555000000000
C -2.291859000000000 2.393601000000000 -1.687402000000000
C -2.900443000000000 1.369675000000000 -0.968324000000000
O -2.976255000000000 -0.331408000000000 -2.956964000000000
O -3.479529000000000 -1.168561000000000 -0.665502000000000
S -2.706736000000000 -0.299944000000000 -1.531487000000000
C 3.707538000000000 -2.921354000000000 -0.926758000000000
S 3.232276000000000 -2.503332000000000 0.771995000000000
C -0.312103000000000 1.599683000000000 1.146808000000000
C -0.743223000000000 2.215905000000000 2.312181000000000
C -1.500284000000000 1.507658000000000 3.239618000000000
C -1.831616000000000 0.181553000000000 2.991494000000000
C -1.411759000000000 -0.434819000000000 1.819098000000000
C -0.648276000000000 0.269695000000000 0.889016000000000
C 4.776488000000000 3.503166000000000 0.656232000000000
O 3.889647000000000 2.405401000000000 0.374138000000000
O 4.381586000000000 2.594393000000000 -1.813455000000000
O 0.540457000000000 -3.659018000000000 -0.880733000000000
C -0.683898000000000 -4.955788000000000 1.107391000000000
O 0.093760000000000 -3.759113000000000 1.321915000000000
C 0.658133000000000 -3.218103000000000 0.239742000000000
C 3.782052000000000 2.041937000000000 -0.913133000000000
C 1.481543000000000 -1.998597000000000 0.591189000000000
C -0.986444000000000 -0.681929000000000 -1.382767000000000
C -0.190858000000000 -0.402468000000000 -0.352733000000000
C 1.264403000000000 -0.859206000000000 -0.422596000000000
C 2.212838000000000 0.273164000000000 -0.161624000000000
C 2.864725000000000 0.909689000000000 -1.131617000000000
H -4.104574000000000 0.808405000000000 0.710055000000000
H -4.380714000000000 3.152339000000000 1.482200000000000
H -3.298603000000000 4.993307000000000 0.234132000000000
H -1.965533000000000 4.506840000000000 -1.793202000000000
H -1.710970000000000 2.170704000000000 -2.572113000000000
H 3.702082000000000 -2.040921000000000 -1.566975000000000
H 4.725257000000000 -3.303080000000000 -0.854659000000000
H 3.058895000000000 -3.693056000000000 -1.334669000000000
H 0.259856000000000 2.159857000000000 0.420400000000000
H -0.495140000000000 3.253949000000000 2.491650000000000
H -1.836129000000000 1.990986000000000 4.148131000000000
H -2.424826000000000 -0.374930000000000 3.705702000000000
H -1.683169000000000 -1.461842000000000 1.618434000000000
H 4.730500000000000 3.643567000000000 1.732173000000000
H 4.442930000000000 4.401546000000000 0.138449000000000
H 5.791413000000000 3.258918000000000 0.345350000000000
H -0.052644000000000 -5.743692000000000 0.699604000000000
H -1.505014000000000 -4.748946000000000 0.423018000000000
H -1.060929000000000 -5.234032000000000 2.086664000000000
H 1.217148000000000 -1.664202000000000 1.593473000000000
H -0.631685000000000 -1.200072000000000 -2.264895000000000
H 1.437698000000000 -1.249094000000000 -1.423837000000000
H 2.342699000000000 0.583224000000000 0.867230000000000
H 2.755293000000000 0.619502000000000 -2.169474000000000
44
R5_SMe4_P2_SMe2R charge=0 multiplicity=1 B3LYP_D3BJ_TZVPD=-1757.200696211643, B3LYP_D3BJ_TZVPD_SMD_chcl3=-1757.22517816552, B3LYP_TZVP_CPCM_chcl3_freq=0.30086385, B3LYP_TZVPD_CPCM_chcl3=-1757.219160401794, M062X_TZVPD_SMD_chcl3=-1757.3481733549, M062X_TZVPD=-1757.323400680248, COSMORS=-0.00490032442072675, wB97MV_TZVPD_CPCM_chcl3=-1757.380188531731, wB97MV_TZVPD_SMD_chcl3=-1757.386377163057, wB97MV_TZVPD=-1757.361614019336, M062X_TZVPD_CPCM_chcl3=-1757.342223111733
C -5.274630000000000 1.308815000000000 0.738802000000000
C -0.823858000000000 -2.945247000000000 2.584839000000000
C -1.449910000000000 0.109297000000000 -3.034390000000000
C 2.713034000000000 -0.479763000000000 -2.227943000000000
C 2.380548000000000 3.301556000000000 0.674005000000000
C 1.060967000000000 2.513855000000000 0.732381000000000
C 3.377988000000000 2.391718000000000 1.390267000000000
C -1.628635000000000 0.689297000000000 0.796676000000000
C 1.480986000000000 1.064682000000000 0.627493000000000
C 2.899900000000000 0.983304000000000 1.091858000000000
C -0.689970000000000 0.208704000000000 -0.310716000000000
C -3.078140000000000 0.495144000000000 0.422546000000000
C 0.739901000000000 0.046031000000000 0.155638000000000
C 1.299620000000000 -1.344551000000000 0.016880000000000
C 0.402603000000000 -2.398171000000000 0.640110000000000
O 3.590577000000000 -0.011567000000000 1.230573000000000
O -3.501523000000000 -0.409656000000000 -0.259611000000000
O 0.065447000000000 -3.431210000000000 0.115628000000000
O -3.857193000000000 1.431240000000000 0.976763000000000
O 0.043972000000000 -2.035357000000000 1.876358000000000
S -0.823004000000000 1.291234000000000 -1.808667000000000
S 1.560808000000000 -1.792655000000000 -1.745676000000000
H -5.481927000000000 1.369404000000000 -0.328414000000000
H -5.729749000000000 2.142543000000000 1.264963000000000
H -5.641623000000000 0.361173000000000 1.130315000000000
H -0.337329000000000 -3.911543000000000 2.705056000000000
H -1.758128000000000 -3.066552000000000 2.038880000000000
H -1.000313000000000 -2.481392000000000 3.550407000000000
H -2.427102000000000 -0.265891000000000 -2.737847000000000
H -0.744372000000000 -0.710188000000000 -3.158013000000000
H -1.536517000000000 0.662049000000000 -3.969392000000000
H 2.233631000000000 0.496634000000000 -2.182931000000000
H 2.989995000000000 -0.696094000000000 -3.259061000000000
H 3.604618000000000 -0.494169000000000 -1.603832000000000
H 2.295899000000000 4.287233000000000 1.129005000000000
H 2.681064000000000 3.435052000000000 -0.367493000000000
H 0.362287000000000 2.818811000000000 -0.043445000000000
H 0.572895000000000 2.687247000000000 1.697441000000000
H 3.321156000000000 2.526256000000000 2.476937000000000
H 4.420055000000000 2.515751000000000 1.098054000000000
H -1.452232000000000 0.097694000000000 1.700458000000000
H -1.459379000000000 1.730392000000000 1.056270000000000
H -1.053202000000000 -0.756077000000000 -0.657369000000000
H 2.260925000000000 -1.400733000000000 0.523942000000000
54

Sulf_SMe1_2_-1_1 charge=-1 multiplicity=1 wB97MV_TZVPD_SMD_chcl3=-2138.157900549367, B3LYP_D3BJ_TZVPD=-2137.852475262242, B3LYP_TZVP_CPCM_chcl3_freq=0.3553365, B3LYP_TZVPD_CPCM_chcl3=-2137.923511491697, M062X_TZVPD=-2138.003047038068, COSMORS=-0.015171133494325618, M062X_TZVPD_CPCM_chcl3=-2138.077351159308, M062X_TZVPD_SMD_chcl3=-2138.083903410944, wB97MV_TZVPD_CPCM_chcl3=-2138.151161021525, B3LYP_D3BJ_TZVPD_SMD_chcl3=-2137.929795830284, wB97MV_TZVPD=-2138.078060310228

C 3.755506000000000 -0.173291000000000 0.132373000000000
C 4.171631000000000 -0.968433000000000 1.193746000000000
C 3.627647000000000 -2.235656000000000 1.371161000000000
C 2.673615000000000 -2.722558000000000 0.480201000000000
C 2.257497000000000 -1.939430000000000 -0.586602000000000
C 2.798411000000000 -0.665960000000000 -0.743800000000000
O 2.119408000000000 -0.480570000000000 -3.268052000000000
O 3.032889000000000 1.540194000000000 -2.142148000000000
S 2.205404000000000 0.349783000000000 -2.079020000000000
C -3.239806000000000 -2.022211000000000 -2.135772000000000
S -1.504741000000000 -2.304752000000000 -1.700556000000000
C 0.776629000000000 0.882919000000000 1.914724000000000
C 1.574786000000000 1.234185000000000 2.993364000000000
C 2.507478000000000 2.260721000000000 2.869681000000000
C 2.636234000000000 2.931281000000000 1.658837000000000
C 1.855592000000000 2.564386000000000 0.569220000000000
C 0.925016000000000 1.529773000000000 0.683566000000000
C -1.401698000000000 -3.978065000000000 2.527872000000000
O -0.981557000000000 -2.985476000000000 1.576050000000000
O -3.134619000000000 -2.443611000000000 1.219768000000000
O -3.603319000000000 4.148602000000000 -0.045329000000000
C -5.518395000000000 2.394306000000000 0.345390000000000
O -4.172857000000000 1.965072000000000 0.156793000000000
C -3.234747000000000 2.963779000000000 -0.039478000000000
C -1.960182000000000 -2.289105000000000 0.966624000000000
C -1.913654000000000 2.504189000000000 -0.222326000000000
C 0.535503000000000 0.720636000000000 -1.649356000000000
C 0.076780000000000 1.130399000000000 -0.461759000000000
C -1.409067000000000 1.178592000000000 -0.237670000000000
C -2.051693000000000 -0.016636000000000 -0.033605000000000
C -1.395645000000000 -1.335901000000000 -0.054029000000000
H 4.151186000000000 0.820939000000000 -0.008485000000000
H 4.910432000000000 -0.589938000000000 1.887929000000000
H 3.945585000000000 -2.846930000000000 2.206232000000000
H 2.250895000000000 -3.708993000000000 0.617818000000000
H 1.519242000000000 -2.307306000000000 -1.286371000000000
H -3.907851000000000 -2.540232000000000 -1.449535000000000
H -3.453079000000000 -0.953743000000000 -2.126398000000000
H -3.376691000000000 -2.410806000000000 -3.144169000000000
H 0.042054000000000 0.095946000000000 2.018147000000000
H 1.464987000000000 0.710129000000000 3.934483000000000
H 3.123546000000000 2.540384000000000 3.715130000000000
H 3.349076000000000 3.740055000000000 1.557619000000000
H 1.963607000000000 3.078526000000000 -0.374330000000000
H -0.486348000000000 -4.420598000000000 2.910838000000000
H -1.968927000000000 -3.516033000000000 3.334997000000000
H -2.015331000000000 -4.734228000000000 2.038915000000000
H -5.886362000000000 2.942483000000000 -0.523640000000000
H -5.612213000000000 3.032621000000000 1.225683000000000
H -6.101783000000000 1.485530000000000 0.483885000000000
H -1.197893000000000 3.300628000000000 -0.377313000000000
H -0.137172000000000 0.434196000000000 -2.446392000000000
H -3.109147000000000 -0.010508000000000 0.171562000000000
H -0.319968000000000 -1.285784000000000 0.077563000000000

44

R5_SMe1_P2_SMe6SS charge=0 multiplicity=1 B3LYP_D3BJ_TZVPD=-1757.200457732002, B3LYP_D3BJ_TZVPD_SMD_chcl3=-1757.225766453354, B3LYP_TZVP_CPCM_chcl3_freq=0.30089525, B3LYP_TZVPD_CPCM_chcl3=-1757.219262190078, M062X_TZVPD_SMD_chcl3=-1757.352088619611, M062X_TZVPD=-1757.326125898799, COSMORS=-0.0035794997411719687, wB97MV_TZVPD_CPCM_chcl3=-1757.382727246914, wB97MV_TZVPD_SMD_chcl3=-1757.389375182411, wB97MV_TZVPD=-1757.363577714496, M062X_TZVPD_CPCM_chcl3=-1757.34571907838

C 2.468548000000000 -3.987916000000000 -0.627135000000000
C -5.540993000000000 1.303235000000000 -0.532246000000000
C 2.617580000000000 4.071247000000000 -0.920255000000000
C -2.179817000000000 -2.472380000000000 1.097196000000000
C 2.832940000000000 0.329077000000000 2.020682000000000
C 2.690924000000000 1.760800000000000 1.492176000000000
C -1.040337000000000 -0.348048000000000 -0.831173000000000
C 1.181645000000000 -0.728842000000000 -1.814864000000000
C 0.279590000000000 -0.194244000000000 -0.722922000000000
C 1.662085000000000 -0.432208000000000 1.430900000000000
C 1.973264000000000 1.578877000000000 0.146002000000000
C -2.074678000000000 0.188074000000000 0.104865000000000
C 2.176637000000000 -1.740748000000000 -1.290907000000000
C 0.929172000000000 0.490748000000000 0.441508000000000
C -3.451351000000000 0.202907000000000 -0.522560000000000
O 1.346113000000000 -1.566652000000000 1.692544000000000
O 3.306361000000000 -1.475539000000000 -0.943056000000000
O -3.868246000000000 -0.634801000000000 -1.290068000000000
O 1.647837000000000 -2.964464000000000 -1.225533000000000
O -4.170554000000000 1.243150000000000 -0.089113000000000
S 1.164786000000000 3.051600000000000 -0.541579000000000
S -2.242085000000000 -0.762488000000000 1.690837000000000
H 2.702580000000000 -3.716811000000000 0.400975000000000
H 3.388105000000000 -4.114954000000000 -1.196202000000000
H 1.870491000000000 -4.893625000000000 -0.658271000000000
H -6.082922000000000 0.422438000000000 -0.190459000000000
H -5.584154000000000 1.362302000000000 -1.618646000000000
H -5.951174000000000 2.202781000000000 -0.083252000000000
H 3.146318000000000 4.358080000000000 -0.012852000000000
H 2.250576000000000 4.967332000000000 -1.418521000000000
H 3.290289000000000 3.538256000000000 -1.591995000000000
H -1.215138000000000 -2.680584000000000 0.639070000000000
H -2.990292000000000 -2.674393000000000 0.399285000000000
H -2.301556000000000 -3.094749000000000 1.983039000000000
H 2.843494000000000 0.241033000000000 3.107164000000000
H 3.739885000000000 -0.151162000000000 1.641865000000000
H 3.647091000000000 2.269036000000000 1.384547000000000
H 2.061842000000000 2.354435000000000 2.158632000000000
H -1.434229000000000 -0.902251000000000 -1.673862000000000
H 1.753892000000000 0.086584000000000 -2.259068000000000
H 0.572716000000000 -1.191073000000000 -2.589168000000000
H 2.687935000000000 1.217358000000000 -0.594963000000000
H -1.837127000000000 1.193669000000000 0.448191000000000
H 0.157132000000000 0.949631000000000 1.070621000000000

37

R5_SMe6_cyc4S_-1_1 charge=-1 multiplicity=1 wB97MV_TZVPD_SMD_chcl3=-1318.154201643398, B3LYP_D3BJ_TZVPD=-1317.932930361164, B3LYP_TZVP_CPCM_chcl3_freq=0.2412195, B3LYP_TZVPD_CPCM_chcl3=-1318.004159942417, M062X_TZVPD=-1318.035204282987, COSMORS=-0.008849396271300583, M062X_TZVPD_CPCM_chcl3=-1318.109234861556, M062X_TZVPD_SMD_chcl3=-1318.111288975088, wB97MV_TZVPD_CPCM_chcl3=-1318.151933749823, B3LYP_D3BJ_TZVPD_SMD_chcl3=-1318.006139180745, wB97MV_TZVPD=-1318.079141546659

C -1.634234000000000 1.334801000000000 -0.352771000000000
C -1.180469000000000 0.134331000000000 0.236304000000000
C 0.084953000000000 -0.375612000000000 0.269711000000000
C 0.441447000000000 -1.760536000000000 0.847084000000000
C 1.352281000000000 0.093163000000000 -0.242430000000000
C 1.954352000000000 1.304250000000000 -0.867963000000000
C 2.310983000000000 -0.785591000000000 0.069612000000000
O 1.885204000000000 -1.881505000000000 0.772982000000000
C 3.414103000000000 0.840274000000000 -1.176442000000000
C 3.694178000000000 -0.422754000000000 -0.311811000000000
C -5.214782000000000 1.329422000000000 0.327800000000000
O -3.854758000000000 0.919122000000000 0.307213000000000
O -3.412869000000000 2.824583000000000 -0.851696000000000
O 0.326755000000000 -3.355102000000000 -0.987622000000000
C -2.223577000000000 -3.957621000000000 -0.447778000000000
O -1.432459000000000 -3.100118000000000 0.394753000000000
C -0.184814000000000 -2.839212000000000 -0.021824000000000
C -2.965798000000000 1.778783000000000 -0.345978000000000
H -0.933926000000000 1.994536000000000 -0.847258000000000
H -1.944606000000000 -0.469653000000000 0.715472000000000
H 0.135046000000000 -1.911879000000000 1.884305000000000
H 1.445437000000000 1.646305000000000 -1.769805000000000
H 3.475799000000000 0.573760000000000 -2.232683000000000
H 4.133851000000000 1.636998000000000 -0.994184000000000
H 4.206020000000000 -1.202445000000000 -0.883262000000000
H 4.319989000000000 -0.204464000000000 0.562004000000000
H -5.339720000000000 2.284975000000000 0.841954000000000
H -5.753577000000000 0.550963000000000 0.867207000000000
H -5.622188000000000 1.425344000000000 -0.681103000000000
H -1.761225000000000 -4.940458000000000 -0.529705000000000
H -2.326222000000000 -3.517404000000000 -1.438930000000000
H -3.192246000000000 -4.030590000000000 0.038082000000000
S 1.860230000000000 2.808061000000000 0.212372000000000
C 2.570188000000000 2.181317000000000 1.757844000000000
H 2.462263000000000 2.976880000000000 2.494154000000000
H 2.024246000000000 1.302012000000000 2.096199000000000
H 3.627118000000000 1.940691000000000 1.646039000000000

44

R5_SMe4_P3_SMe6RRS_P2 charge=0 multiplicity=1 B3LYP_D3BJ_TZVPD=-1757.198538648329, B3LYP_D3BJ_TZVPD_SMD_chcl3=-1757.224103887144, B3LYP_TZVP_CPCM_chcl3_freq=0.30160455, B3LYP_TZVPD_CPCM_chcl3=-1757.217941758491, M062X_TZVPD_SMD_chcl3=-1757.348393711756, M062X_TZVPD=-1757.322782828303, COSMORS=-0.003920865105127701, wB97MV_TZVPD_CPCM_chcl3=-1757.380697118345, wB97MV_TZVPD_SMD_chcl3=-1757.386992827694, wB97MV_TZVPD=-1757.361384859838, M062X_TZVPD_CPCM_chcl3=-1757.342358643713

C -5.564967000000000 1.584430000000000 -0.478865000000000
C 5.122981000000000 0.861997000000000 0.280821000000000
C 1.163456000000000 -2.454712000000000 -2.667896000000000
C 0.770847000000000 2.809074000000000 -1.605740000000000
C -0.993296000000000 0.483538000000000 0.463329000000000
C -0.776320000000000 -2.533000000000000 2.142096000000000
C -1.276699000000000 -2.805847000000000 0.720180000000000
C -2.032373000000000 0.632497000000000 -0.353028000000000
C 0.368921000000000 0.043424000000000 0.023529000000000
C 0.389893000000000 -1.574977000000000 1.991934000000000
C -0.038377000000000 -2.558433000000000 -0.160483000000000
C -3.350285000000000 1.048870000000000 0.159037000000000
C 0.725574000000000 -1.393950000000000 0.509754000000000
C 1.434957000000000 1.031605000000000 0.518744000000000
C 2.792982000000000 0.754857000000000 -0.085080000000000
O 0.975523000000000 -1.034293000000000 2.902220000000000
O -3.632784000000000 1.248497000000000 1.322049000000000
O 2.973081000000000 0.385423000000000 -1.225148000000000
O -4.233271000000000 1.181525000000000 -0.847267000000000
O 3.778101000000000 0.999317000000000 0.782405000000000
S -0.485185000000000 -2.386137000000000 -1.910655000000000
S 1.036921000000000 2.791797000000000 0.187642000000000
H -6.121023000000000 1.638026000000000 -1.410276000000000
H -5.543872000000000 2.557253000000000 0.010730000000000
H -6.010173000000000 0.849631000000000 0.190844000000000
H 5.288498000000000 1.558430000000000 -0.539985000000000
H 5.294562000000000 -0.157285000000000 -0.061620000000000
H 5.769828000000000 1.098230000000000 1.120166000000000
H 1.014523000000000 -2.428258000000000 -3.746448000000000
H 1.666763000000000 -3.383074000000000 -2.397876000000000
H 1.768938000000000 -1.600410000000000 -2.368339000000000
H -0.130269000000000 2.263284000000000 -1.879351000000000
H 1.633168000000000 2.405443000000000 -2.132191000000000
H 0.646448000000000 3.858599000000000 -1.869685000000000
H -1.133842000000000 0.683761000000000 1.521492000000000
H -0.387193000000000 -3.440572000000000 2.614580000000000
H -1.525313000000000 -2.114027000000000 2.814861000000000
H -2.072223000000000 -2.108599000000000 0.459458000000000
H -1.659968000000000 -3.816192000000000 0.590222000000000
H -1.950834000000000 0.446217000000000 -1.415422000000000
H 0.396995000000000 0.016126000000000 -1.063859000000000
H 0.606839000000000 -3.437859000000000 -0.095949000000000
H 1.799936000000000 -1.524515000000000 0.380975000000000
H 1.514808000000000 0.994791000000000 1.604093000000000

40

R6_SMe6_cyc4RETS_-1_1 charge=-1 multiplicity=1 wB97MV_TZVPD_SMD_chcl3=-1357.442601639838, B3LYP_TZVP_CPCM_chcl3_freq=0.26823498, B3LYP_TZVPD_CPCM_chcl3=-1357.299201329229, M062X_TZVPD=-1357.327951437548, COSMORS=-0.011616079599206967, M062X_TZVPD_CPCM_chcl3=-1357.397654065124, M062X_TZVPD_SMD_chcl3=-1357.400120881549, wB97MV_TZVPD_CPCM_chcl3=-1357.439914348118, B3LYP_D3BJ_TZVPD_SMD_chcl3=-1357.301546953531, wB97MV_TZVPD=-1357.371562941003

O 4.222052000000000 -1.268705000000000 0.473207000000000
C 3.905784000000000 0.048227000000000 0.221569000000000
C -1.757685000000000 -2.148419000000000 -0.422537000000000
H 5.667753000000000 -2.641821000000000 0.766929000000000
C 1.519411000000000 -0.644085000000000 0.289884000000000
C 2.507562000000000 0.313417000000000 0.136607000000000
H 6.128579000000000 -1.346145000000000 -0.370063000000000
O 4.805477000000000 0.883157000000000 0.095312000000000
C 5.612071000000000 -1.574031000000000 0.563821000000000
H 6.087052000000000 -1.016252000000000 1.372445000000000
H -3.021552000000000 -4.077224000000000 -1.759478000000000
H -0.193996000000000 -2.502582000000000 0.997778000000000
C 0.130048000000000 -0.525581000000000 0.213125000000000
H -4.183430000000000 -3.213463000000000 -0.727136000000000
C -0.662452000000000 -1.675139000000000 0.472393000000000
C -0.700204000000000 0.677873000000000 0.354561000000000
C -0.391546000000000 2.065505000000000 -0.107980000000000
H -3.578514000000000 -4.820836000000000 -0.232002000000000
S -0.065927000000000 2.164113000000000 -1.928727000000000
C -1.718181000000000 0.403723000000000 1.219638000000000
H -1.041403000000000 2.918582000000000 2.418280000000000
H 0.569375000000000 2.405269000000000 0.289427000000000
C -2.539187000000000 1.455273000000000 1.905452000000000
O -1.951554000000000 -0.856170000000000 1.578309000000000
H -2.343733000000000 2.959446000000000 -0.337860000000000
C -1.909924000000000 2.839874000000000 1.756736000000000
H -3.554122000000000 1.453143000000000 1.488882000000000
H -2.642189000000000 1.174082000000000 2.957650000000000
C -1.471212000000000 3.068223000000000 0.311671000000000
H -2.619191000000000 3.613559000000000 2.062811000000000
H -1.089754000000000 4.081374000000000 0.172382000000000
C -1.617621000000000 1.525150000000000 -2.617714000000000
H -2.442575000000000 2.221986000000000 -2.471844000000000
H -1.449521000000000 1.396640000000000 -3.686805000000000
H -1.852129000000000 0.561177000000000 -2.170646000000000
O -2.243231000000000 -3.325441000000000 0.022462000000000
O -2.163860000000000 -1.617378000000000 -1.432604000000000
C -3.327797000000000 -3.888550000000000 -0.730792000000000
H 1.871262000000000 -1.659623000000000 0.452913000000000
H 2.268243000000000 1.342100000000000 -0.084993000000000

44

R5_SMe1_P2A_SMe6SR charge=0 multiplicity=1 B3LYP_D3BJ_TZVPD=-1757.194928980975, B3LYP_D3BJ_TZVPD_SMD_chcl3=-1757.222919013145, B3LYP_TZVP_CPCM_chcl3_freq=0.30095638, B3LYP_TZVPD_CPCM_chcl3=-1757.216292773612, M062X_TZVPD_SMD_chcl3=-1757.348254221014, M062X_TZVPD=-1757.319846871922, COSMORS=-0.00118205386636555, wB97MV_TZVPD_CPCM_chcl3=-1757.379343651087, wB97MV_TZVPD_SMD_chcl3=-1757.38607321616, M062X_TZVPD_CPCM_chcl3=-1757.341815507968

C 0.014220000000000 -3.955928000000000 -0.836421000000000
C 4.734403000000000 -2.129993000000000 0.637383000000000
C -1.200884000000000 3.680820000000000 0.617195000000000
C 1.639035000000000 2.535945000000000 -1.750481000000000
C -3.836538000000000 -0.782793000000000 0.865483000000000
C -3.855691000000000 0.490415000000000 0.016246000000000
C 0.780312000000000 0.435067000000000 0.624045000000000
C -0.705325000000000 -0.345973000000000 -1.240747000000000
C -0.440685000000000 0.175938000000000 0.153036000000000
C -2.396330000000000 -0.950030000000000 1.302814000000000
C -2.733250000000000 1.353792000000000 0.620224000000000
C 2.067014000000000 0.322968000000000 -0.118499000000000
C -0.948258000000000 -1.842151000000000 -1.272500000000000
C -1.624844000000000 0.366057000000000 1.072594000000000
C 3.112665000000000 -0.416436000000000 0.693581000000000
O -1.906389000000000 -1.949693000000000 1.770283000000000
O -1.993850000000000 -2.362211000000000 -1.588439000000000
O 3.384482000000000 -0.167836000000000 1.844289000000000
O 0.141875000000000 -2.522176000000000 -0.908580000000000
O 3.701379000000000 -1.372351000000000 -0.028254000000000
S -2.238768000000000 2.696681000000000 -0.498461000000000
S 2.771225000000000 2.007018000000000 -0.438586000000000
H -0.753718000000000 -4.223001000000000 -0.112171000000000
H 0.986494000000000 -4.316756000000000 -0.514837000000000
H -0.242709000000000 -4.361745000000000 -1.813696000000000
H 5.085675000000000 -2.846747000000000 -0.098462000000000
H 4.322144000000000 -2.640011000000000 1.506422000000000
H 5.541752000000000 -1.468279000000000 0.946942000000000
H -1.769236000000000 3.986785000000000 1.495200000000000
H -0.893556000000000 4.565891000000000 0.062117000000000
H -0.315570000000000 3.123485000000000 0.918885000000000
H 1.768832000000000 1.927380000000000 -2.644521000000000
H 1.895134000000000 3.570268000000000 -1.976376000000000
H 0.605793000000000 2.489272000000000 -1.409142000000000
H -4.191055000000000 -1.678978000000000 0.358619000000000
H -4.432586000000000 -0.656520000000000 1.776172000000000
H -4.812799000000000 1.008154000000000 0.043702000000000
H -3.641854000000000 0.248022000000000 -1.025846000000000
H 0.883402000000000 0.797384000000000 1.640044000000000
H 0.136160000000000 -0.135926000000000 -1.898198000000000
H -1.578319000000000 0.134511000000000 -1.671960000000000
H -3.122013000000000 1.832644000000000 1.520899000000000
H 1.966673000000000 -0.172038000000000 -1.078550000000000
H -1.233279000000000 0.669628000000000 2.043804000000000

44

R5_SMe4_P2_SMe3SR charge=0 multiplicity=1 B3LYP_D3BJ_TZVPD=-1757.187083036029, B3LYP_D3BJ_TZVPD_SMD_chcl3=-1757.212953515963, B3LYP_TZVP_CPCM_chcl3_freq=0.3010277, B3LYP_TZVPD_CPCM_chcl3=-1757.20724013252, M062X_TZVPD_SMD_chcl3=-1757.338775571188, M062X_TZVPD=-1757.312409091555, COSMORS=-0.004407646600267601, wB97MV_TZVPD_CPCM_chcl3=-1757.370895321215, wB97MV_TZVPD_SMD_chcl3=-1757.376786838342, wB97MV_TZVPD=-1757.350582433337, M062X_TZVPD_CPCM_chcl3=-1757.333111834382

C -5.123180000000000 2.302748000000000 -0.968253000000000
C 0.964516000000000 -4.515461000000000 1.600106000000000
C 1.199593000000000 -2.892466000000000 -2.619486000000000
C 0.025313000000000 0.950609000000000 2.958172000000000
C 3.340737000000000 2.110416000000000 -1.371872000000000
C 2.705155000000000 0.756421000000000 -1.029014000000000
C 2.753188000000000 3.054934000000000 -0.323989000000000
C -1.693022000000000 1.171969000000000 -0.376125000000000
C -0.977625000000000 0.267216000000000 0.285033000000000
C 1.256432000000000 1.101924000000000 -0.649289000000000
C 1.370648000000000 2.506087000000000 -0.034065000000000
C -3.160836000000000 1.208826000000000 -0.228497000000000
C 0.799700000000000 -1.367051000000000 -0.321026000000000
C 0.307665000000000 -2.487806000000000 0.582107000000000
C 0.503296000000000 0.065218000000000 0.210115000000000
O 0.502960000000000 3.100364000000000 0.562816000000000
O -3.825624000000000 0.498549000000000 0.495761000000000
O -0.811434000000000 -2.626900000000000 1.013534000000000
O -3.692341000000000 2.159568000000000 -1.018381000000000
O 1.296363000000000 -3.367737000000000 0.792590000000000
S 0.094838000000000 -1.591526000000000 -2.000593000000000
S 1.238430000000000 0.061085000000000 1.941043000000000
H -5.606906000000000 1.376202000000000 -1.275165000000000
H -5.360213000000000 3.104858000000000 -1.661120000000000
H -5.441800000000000 2.562045000000000 0.040667000000000
H 0.664739000000000 -4.195500000000000 2.596858000000000
H 1.871884000000000 -5.110070000000000 1.645021000000000
H 0.156165000000000 -5.079301000000000 1.137298000000000
H 0.913753000000000 -3.064306000000000 -3.656313000000000

H 1.076677000000000 -3.815446000000000 -2.056059000000000
H 2.237051000000000 -2.562461000000000 -2.579639000000000
H 0.498223000000000 1.040072000000000 3.936030000000000
H -0.898999000000000 0.387093000000000 3.058303000000000
H -0.166763000000000 1.939060000000000 2.550872000000000
H 3.034786000000000 2.423202000000000 -2.373256000000000
H 4.429153000000000 2.074815000000000 -1.352060000000000
H 3.222196000000000 0.319858000000000 -0.170422000000000
H 2.758599000000000 0.048913000000000 -1.854679000000000
H 2.705972000000000 4.106789000000000 -0.604394000000000
H 3.312219000000000 2.987427000000000 0.616230000000000
H -1.258577000000000 1.912784000000000 -1.029151000000000
H -1.505624000000000 -0.439221000000000 0.909582000000000
H 0.701218000000000 1.241101000000000 -1.584680000000000
H 1.875558000000000 -1.481116000000000 -0.415981000000000
40
R6_SMe4_TS_-1_1 charge=-1 multiplicity=1 wB97MV_TZVPD_SMD_chcl3=-1357.464649058826, B3LYP_D3BJ_TZVPD=-1357.260014462845, B3LYP_TZVP_CPCM_chcl3_freq=0.26505759, B3LYP_TZVPD_CPCM_chcl3=-1357.329527484136, M062X_TZVPD=-1357.352011033711, COSMORS=-0.00978053759131055, M062X_TZVPD_CPCM_chcl3=-1357.423388739202, M062X_TZVPD_SMD_chcl3=-1357.425358353666, wB97MV_TZVPD_CPCM_chcl3=-1357.462414276212, B3LYP_D3BJ_TZVPD_SMD_chcl3=-1357.331492814245, wB97MV_TZVPD=-1357.392187424318
O -3.753187000000000 0.346185000000000 -0.522296000000000
C -3.284432000000000 -0.939322000000000 -0.410116000000000
C 0.831665000000000 2.455651000000000 -0.494009000000000
H -5.347224000000000 1.560652000000000 -0.736647000000000
C -1.004653000000000 0.039312000000000 -0.229525000000000
C -1.866428000000000 -1.037873000000000 -0.267247000000000
H -5.527561000000000 -0.014525000000000 -1.556856000000000
O -4.068748000000000 -1.889613000000000 -0.433740000000000
C -5.166161000000000 0.490534000000000 -0.659890000000000
H -5.689548000000000 0.084927000000000 0.207373000000000
H 0.869715000000000 4.534930000000000 -2.168194000000000
H -1.496116000000000 -2.050794000000000 -0.177568000000000
C 0.390141000000000 -0.022595000000000 -0.112918000000000
H 2.593560000000000 4.838129000000000 -1.807493000000000
C 1.242929000000000 1.100400000000000 -0.055925000000000
C 1.047105000000000 -1.352055000000000 0.018155000000000
C 1.430310000000000 -1.829073000000000 1.215025000000000
H 1.352987000000000 5.065452000000000 -0.541040000000000
S 1.715085000000000 1.802554000000000 2.068503000000000
C 1.267978000000000 -2.169895000000000 -1.199250000000000
H 3.646746000000000 -3.100816000000000 -0.079465000000000
H 2.284075000000000 0.906058000000000 -0.269168000000000
C 1.762470000000000 -3.586979000000000 -0.991332000000000
O 1.039330000000000 -1.744642000000000 -2.322223000000000
H 1.401757000000000 -3.845299000000000 1.848528000000000
C 2.753454000000000 -3.683723000000000 0.165500000000000
H 0.880089000000000 -4.202681000000000 -0.775096000000000
H 2.186913000000000 -3.943114000000000 -1.930422000000000
C 2.131051000000000 -3.130248000000000 1.443005000000000
H 3.073099000000000 -4.717896000000000 0.307871000000000
H 2.886014000000000 -3.003888000000000 2.223880000000000
C 0.036253000000000 1.573626000000000 2.697619000000000
H -0.452089000000000 0.787272000000000 2.112545000000000
H 0.071692000000000 1.254565000000000 3.739599000000000
H -0.553702000000000 2.486578000000000 2.618143000000000
O 1.886663000000000 3.113834000000000 -1.029177000000000
O -0.273476000000000 2.954242000000000 -0.428902000000000
C 1.649331000000000 4.476131000000000 -1.409168000000000
H -1.438055000000000 1.026137000000000 -0.309393000000000
H 1.219444000000000 -1.228462000000000 2.093137000000000
37
R5_SMe1_TS_-1_1 charge=-1 multiplicity=1 wB97MV_TZVPD_SMD_chcl3=-1318.161796761226, B3LYP_D3BJ_TZVPD=-1317.958009745028, CCSDT_SMD_chcl3=-1316.24927007006, CCSDT=-1316.180430767874, B3LYP_TZVP_CPCM_chcl3_freq=0.23654297, B3LYP_TZVPD_CPCM_chcl3=-1318.025852450737, M062X_TZVPD=-1318.047025331076, COSMORS=-0.00863771819233965, M062X_TZVPD_CPCM_chcl3=-1318.1170876285, M062X_TZVPD_SMD_chcl3=-1318.11820284191, wB97MV_TZVPD_CPCM_chcl3=-1318.160377847047, B3LYP_D3BJ_TZVPD_SMD_chcl3=-1318.026920232551, wB97MV_TZVPD=-1318.091469591715
C 1.816131000000000 0.072192000000000 -0.276536000000000
C 0.737690000000000 0.982018000000000 -0.149333000000000
C -0.603734000000000 0.617800000000000 -0.018105000000000
C -1.628092000000000 1.558605000000000 0.060525000000000
C -0.933128000000000 -0.824390000000000 0.014207000000000
C -0.711383000000000 -1.682680000000000 1.017183000000000
C -1.534831000000000 -1.551226000000000 -1.122173000000000
O -1.775548000000000 -1.109981000000000 -2.230695000000000
C -1.172984000000000 -3.080463000000000 0.730545000000000
C -1.766374000000000 -2.990565000000000 -0.680796000000000
C 5.076696000000000 0.006132000000000 -1.963169000000000
O 3.790624000000000 -0.372890000000000 -1.451346000000000
O 3.443375000000000 1.761626000000000 -0.831383000000000
O -3.579231000000000 0.175997000000000 0.273759000000000
C -5.168533000000000 2.263495000000000 0.382175000000000
O -3.758964000000000 2.432817000000000 0.251750000000000
C -3.012297000000000 1.267119000000000 0.198984000000000
C 3.075051000000000 0.605508000000000 -0.857432000000000
H 1.584487000000000 -0.928813000000000 -0.610473000000000
H 0.980077000000000 2.037745000000000 -0.137459000000000
H -1.360003000000000 2.606748000000000 0.021703000000000
H -0.250735000000000 -1.412364000000000 1.957452000000000
H -0.337800000000000 -3.784536000000000 0.788831000000000
H -1.904565000000000 -3.406071000000000 1.475386000000000
H -1.299363000000000 -3.671108000000000 -1.394054000000000
H -2.840334000000000 -3.186778000000000 -0.697218000000000
H 4.974476000000000 0.771085000000000 -2.732407000000000
H 5.503050000000000 -0.900171000000000 -2.384928000000000
H 5.707682000000000 0.383952000000000 -1.158612000000000
H -5.588316000000000 3.267596000000000 0.397137000000000
H -5.421126000000000 1.741984000000000 1.307220000000000
H -5.578647000000000 1.701889000000000 -0.458909000000000
S 2.729150000000000 -0.563308000000000 1.618020000000000
C 2.003448000000000 0.714016000000000 2.670128000000000
H 2.686008000000000 1.548808000000000 2.828276000000000
H 1.095343000000000 1.083245000000000 2.181048000000000
H 1.729461000000000 0.281242000000000 3.632176000000000
41
R6_SMe4_P1SS_0_1 charge=0 multiplicity=1 B3LYP_D3BJ_TZVPD=-1357.824566818275, B3LYP_D3BJ_TZVPD_SMD_chcl3=-1357.849495982343, B3LYP_TZVP_CPCM_chcl3_freq=0.28081365, B3LYP_TZVPD_CPCM_chcl3=-1357.843107545537, M062X_TZVPD_SMD_chcl3=-1357.94888559231, M062X_TZVPD=-1357.923680073374, COSMORS=-0.003175505840717192, wB97MV_TZVPD_CPCM_chcl3=-1357.986425707407, wB97MV_TZVPD_SMD_chcl3=-1357.99298431487, wB97MV_TZVPD=-1357.967880543438, M062X_TZVPD_CPCM_chcl3=-1357.942552617635
O -4.545822000000000 0.311005000000000 -0.692355000000000
C -3.543668000000000 0.542247000000000 0.175627000000000
O -3.726224000000000 0.914649000000000 1.315662000000000
H -6.540031000000000 0.290703000000000 -1.026497000000000
H -6.005774000000000 1.566746000000000 0.106633000000000
C -2.235643000000000 0.276952000000000 -0.452709000000000
C -1.099772000000000 0.426992000000000 0.224763000000000
C -5.879580000000000 0.528380000000000 -0.197670000000000
H -6.079524000000000 -0.125277000000000 0.650523000000000
H -1.134559000000000 0.747667000000000 1.259587000000000
H -2.243017000000000 -0.051379000000000 -1.484272000000000
O -0.051885000000000 -3.200009000000000 0.929639000000000
C 0.260451000000000 0.155029000000000 -0.350862000000000
O -0.306629000000000 -2.567329000000000 -1.212823000000000
C -0.688076000000000 -4.458419000000000 0.629184000000000
H -1.695896000000000 -4.289668000000000 0.253032000000000
H -0.106165000000000 -5.004861000000000 -0.111762000000000
H -0.715977000000000 -5.000253000000000 1.569675000000000
C 0.845951000000000 -1.105476000000000 0.324356000000000
C 1.160274000000000 1.367401000000000 -0.227259000000000
C 0.093015000000000 -2.349482000000000 -0.090700000000000
H 1.166418000000000 1.718100000000000 -2.286374000000000
C 1.515021000000000 2.064112000000000 -1.317578000000000
H 3.739949000000000 -1.694279000000000 -2.044191000000000
O 1.243675000000000 1.256572000000000 2.133034000000000
C 2.375594000000000 3.284173000000000 -1.312797000000000
H 3.659093000000000 2.385392000000000 0.968551000000000
H 2.712967000000000 3.300023000000000 2.143408000000000
C 2.452295000000000 3.933037000000000 0.065795000000000
H 3.379297000000000 2.992239000000000 -1.651420000000000
H 2.006186000000000 3.991065000000000 -2.059931000000000
C 2.692217000000000 2.875208000000000 1.140043000000000
H 3.242447000000000 4.684796000000000 0.092217000000000
H 1.509479000000000 4.447879000000000 0.272606000000000
C 1.645651000000000 1.785660000000000 1.106698000000000
S 2.627494000000000 -1.382970000000000 0.003423000000000
H 0.805966000000000 -1.005670000000000 1.405729000000000
C 2.703591000000000 -1.457430000000000 -1.806268000000000
H 2.055404000000000 -2.239973000000000 -2.194460000000000
H 2.445073000000000 -0.498616000000000 -2.251675000000000
H 0.146640000000000 -0.065598000000000 -1.411869000000000
38
R5_SMe6H_cyc2S_0_1 charge=0 multiplicity=1 B3LYP_D3BJ_TZVPD_SMD_chcl3=-1318.497636717097, B3LYP_TZVP_CPCM_chcl3_freq=0.25477858, B3LYP_TZVPD_CPCM_chcl3=-1318.491888589046, M062X_TZVPD_SMD_chcl3=-1318.600282027565, M062X_TZVPD=-1318.575581206987, COSMORS=-0.004051333254826855, wB97MV_TZVPD_CPCM_chcl3=-1318.637477034547, wB97MV_TZVPD_SMD_chcl3=-1318.643385044718, wB97MV_TZVPD=-1318.618866804113, M062X_TZVPD_CPCM_chcl3=-1318.594553300821
C -2.549230000000000 -0.410943000000000 -0.214834000000000
C -1.274102000000000 -0.750847000000000 0.444399000000000
C -0.082366000000000 0.120083000000000 0.035822000000000
C -0.134655000000000 1.466940000000000 0.158385000000000
C 0.914458000000000 -0.794309000000000 -0.417826000000000
C 2.358328000000000 -0.841984000000000 -0.788801000000000
C 0.397029000000000 -2.042850000000000 -0.343799000000000
O -0.848823000000000 -2.153478000000000 0.100254000000000
C 2.536449000000000 -2.316255000000000 -1.271320000000000
C 1.326168000000000 -3.130848000000000 -0.734557000000000
C -5.000474000000000 0.342489000000000 -1.417875000000000
O -4.924551000000000 -0.100676000000000 -0.057224000000000
O -3.819514000000000 -0.784274000000000 1.734347000000000
O 1.823802000000000 2.141745000000000 -1.045615000000000
C 1.575263000000000 4.654473000000000 -0.261072000000000
O 0.656658000000000 3.639782000000000 0.168065000000000
C 0.887878000000000 2.386340000000000 -0.305237000000000
C -3.722316000000000 -0.426434000000000 0.439779000000000
H -2.522171000000000 -0.135328000000000 -1.256664000000000
H -1.380035000000000 -0.766508000000000 1.528731000000000
H -1.012069000000000 1.915930000000000 0.604689000000000
H 2.634651000000000 -0.124521000000000 -1.556033000000000
H 2.521229000000000 -2.324495000000000 -2.361646000000000
H 3.488732000000000 -2.734411000000000 -0.951861000000000
H 0.895323000000000 -3.793342000000000 -1.487672000000000
H 1.577794000000000 -3.749031000000000 0.132058000000000
H -4.387838000000000 1.233607000000000 -1.561055000000000
H -6.047041000000000 0.573922000000000 -1.592647000000000
H -4.673011000000000 -0.448235000000000 -2.094325000000000
H 1.558446000000000 4.753761000000000 -1.346529000000000
H 2.588591000000000 4.416411000000000 0.061729000000000
H 1.235956000000000 5.574553000000000 0.207491000000000
S 3.466364000000000 -0.359287000000000 0.605402000000000
C 3.020077000000000 -1.577316000000000 1.871522000000000
H 1.955482000000000 -1.529340000000000 2.098423000000000
H 3.290992000000000 -2.588928000000000 1.571121000000000
H 3.583387000000000 -1.311828000000000 2.765172000000000
H -4.727419000000000 -0.632529000000000 2.035179000000000
38
R5_SMe6_esterH_0_1 charge=0 multiplicity=1 B3LYP_D3BJ_TZVPD=-1318.439606626121, B3LYP_D3BJ_TZVPD_SMD_chcl3=-1318.479881515971, B3LYP_TZVP_CPCM_chcl3_freq=0.25266738, B3LYP_TZVPD_CPCM_chcl3=-1318.475091254361, M062X_TZVPD_SMD_chcl3=-1318.567961334046, M062X_TZVPD=-1318.524121648707, COSMORS=0.00416513233348835, wB97MV_TZVPD_CPCM_chcl3=-1318.606438567105, wB97MV_TZVPD_SMD_chcl3=-1318.611366989282, wB97MV_TZVPD=-1318.567552965388, M062X_TZVPD_CPCM_chcl3=-1318.56321555257
C -2.319165000000000 1.030871000000000 -0.553677000000000
C -1.410737000000000 0.112259000000000 -0.125597000000000
C 0.036097000000000 0.251000000000000 -0.302666000000000
C 0.534221000000000 1.537065000000000 -0.150787000000000
C 0.738917000000000 -0.963072000000000 -0.481454000000000
C 2.171844000000000 -1.259393000000000 -0.192263000000000
C 0.033143000000000 -2.141995000000000 -0.824241000000000
O -1.174672000000000 -2.224230000000000 -1.138745000000000
C 2.365030000000000 -2.731500000000000 -0.633626000000000
C 0.959785000000000 -3.341229000000000 -0.729661000000000
C -5.564772000000000 -0.245794000000000 0.663963000000000
O -4.137052000000000 -0.099303000000000 0.417881000000000
O -4.594147000000000 1.741213000000000 -0.708488000000000
O 2.705894000000000 1.468902000000000 -1.163930000000000
C 3.330254000000000 3.852701000000000 -0.227541000000000
O 2.078806000000000 3.235696000000000 0.092923000000000
C 1.859497000000000 2.002963000000000 -0.458990000000000
C -3.694387000000000 0.893754000000000 -0.278230000000000
H -2.020737000000000 1.879093000000000 -1.155705000000000
H -1.766145000000000 -0.735511000000000 0.439651000000000
H -0.126611000000000 2.294948000000000 0.245370000000000
H 2.859337000000000 -0.593305000000000 -0.708927000000000
H 2.837084000000000 -2.734651000000000 -1.618242000000000
H 3.021193000000000 -3.274868000000000 0.044445000000000
H 0.827297000000000 -4.001469000000000 -1.588020000000000
H 0.692480000000000 -3.915087000000000 0.163020000000000
H -6.087725000000000 -0.342995000000000 -0.284261000000000
H -5.927116000000000 0.614358000000000 1.221655000000000
H -5.646007000000000 -1.154262000000000 1.249614000000000
H 3.321934000000000 4.815384000000000 0.277841000000000
H 3.429735000000000 3.993435000000000 -1.304451000000000
H 4.163196000000000 3.247283000000000 0.130613000000000
S 2.643860000000000 -0.922702000000000 1.570510000000000
C 1.390705000000000 -1.860442000000000 2.485787000000000
H 0.389230000000000 -1.528437000000000 2.214517000000000
H 1.484759000000000 -2.931156000000000 2.307791000000000
H 1.558261000000000 -1.660571000000000 3.543472000000000
H -4.196390000000000 2.457801000000000 -1.230352000000000
40
R6_SMe6_cyc4SE_-1_1 charge=-1 multiplicity=1 wB97MV_TZVPD_SMD_chcl3=-1357.475843488286, B3LYP_TZVP_CPCM_chcl3_freq=0.26932196, B3LYP_TZVPD_CPCM_chcl3=-1357.324650937402, M062X_TZVPD=-1357.359535343605, COSMORS=-0.010105122332127988, M062X_TZVPD_CPCM_chcl3=-1357.433887427785, M062X_TZVPD_SMD_chcl3=-1357.436724642571, B3LYP_D3BJ_TZVPD_SMD_chcl3=-1357.327400229969, wB97MV_TZVPD=-1357.39976907427
O -3.990061000000000 -0.336825000000000 0.080241000000000
C -3.330397000000000 0.830704000000000 -0.315288000000000
C 0.476658000000000 -2.936330000000000 -0.078031000000000
H -5.737076000000000 -0.161732000000000 -1.048198000000000
C -1.209320000000000 -0.336888000000000 0.292544000000000
C -1.933326000000000 0.775619000000000 -0.195929000000000
H -5.745267000000000 -1.290693000000000 0.331763000000000
O -4.011343000000000 1.787955000000000 -0.730124000000000
C -5.406947000000000 -0.315937000000000 -0.018420000000000
H -5.842954000000000 0.468707000000000 0.603915000000000
H -0.493016000000000 -5.290634000000000 -0.906263000000000
H 0.409148000000000 -2.151869000000000 1.916512000000000
C 0.135440000000000 -0.522690000000000 0.432837000000000
H -1.323432000000000 -3.956077000000000 -1.742608000000000
C 0.755709000000000 -1.833080000000000 0.930524000000000
C 1.289525000000000 0.300990000000000 0.109914000000000
C 1.348987000000000 1.655546000000000 -0.512034000000000
H -2.140787000000000 -4.811245000000000 -0.402672000000000
S 0.908869000000000 2.920416000000000 0.767436000000000
C 2.411747000000000 -0.357648000000000 0.441119000000000
H 4.832571000000000 1.777891000000000 -0.597300000000000
H 0.585222000000000 1.753978000000000 -1.283913000000000
C 3.817338000000000 0.057903000000000 0.238248000000000
O 2.174574000000000 -1.602398000000000 0.992402000000000
H 2.824071000000000 2.954977000000000 -1.418509000000000
C 3.864058000000000 1.541404000000000 -0.152418000000000
H 4.404383000000000 -0.121488000000000 1.145023000000000
H 4.270114000000000 -0.563329000000000 -0.546287000000000
C 2.736433000000000 1.909111000000000 -1.117899000000000
H 3.764152000000000 2.153064000000000 0.747619000000000
H 2.820358000000000 1.305206000000000 -2.028387000000000
C 0.353304000000000 4.282671000000000 -0.295524000000000
H -0.517237000000000 3.980057000000000 -0.878297000000000
H 0.073126000000000 5.106299000000000 0.360345000000000
H 1.148315000000000 4.615741000000000 -0.962393000000000
O -0.693872000000000 -3.531539000000000 0.196107000000000
O 1.166271000000000 -3.211957000000000 -1.031930000000000
C -1.187305000000000 -4.459890000000000 -0.786334000000000
H -1.822064000000000 -1.179918000000000 0.596603000000000
H -1.423209000000000 1.680008000000000 -0.491274000000000
44
R5_SMe4_P2_SMe2S charge=0 multiplicity=1 B3LYP_D3BJ_TZVPD=-1757.201724648534, B3LYP_D3BJ_TZVPD_SMD_chcl3=-1757.227369258491, B3LYP_TZVP_CPCM_chcl3_freq=0.30297614, B3LYP_TZVPD_CPCM_chcl3=-1757.22147185327, M062X_TZVPD=-1757.326487906759, COSMORS=-0.004721841059711662, wB97MV_TZVPD_CPCM_chcl3=-1757.383937021649, wB97MV_TZVPD_SMD_chcl3=-1757.389942107366, wB97MV_TZVPD=-1757.36388922369, M062X_TZVPD_CPCM_chcl3=-1757.346937158412
C 4.873123000000000 -0.935593000000000 0.722422000000000
C -4.063181000000000 0.756333000000000 1.153011000000000
C -1.391112000000000 2.878907000000000 -1.575018000000000
C 0.544325000000000 -2.557469000000000 1.218733000000000
C -0.637686000000000 -1.849553000000000 -3.281757000000000
C -0.196190000000000 -0.482461000000000 -2.728064000000000
C -1.873695000000000 -2.205762000000000 -2.456531000000000
C 2.146015000000000 1.476925000000000 0.115845000000000
C -0.585909000000000 -0.519349000000000 -1.264325000000000
C -1.611772000000000 -1.581545000000000 -1.100468000000000
C 0.831937000000000 1.409044000000000 -0.661792000000000
C 3.006623000000000 0.255768000000000 -0.095595000000000
C -0.100548000000000 0.279217000000000 -0.293263000000000
C -0.416445000000000 0.164948000000000 1.176097000000000
C -1.872066000000000 0.011112000000000 1.604357000000000
O -2.172933000000000 -1.910621000000000 -0.070966000000000
O 2.872975000000000 -0.545019000000000 -0.992456000000000
O -2.250311000000000 -0.567535000000000 2.590813000000000
O 3.983139000000000 0.193142000000000 0.816171000000000
O -2.662405000000000 0.761502000000000 0.826491000000000
S -0.019510000000000 3.021680000000000 -0.399919000000000
S 0.746742000000000 -0.957542000000000 2.031020000000000
H 5.582815000000000 -0.814588000000000 1.535358000000000
H 5.386526000000000 -0.935744000000000 -0.238019000000000
H 4.312268000000000 -1.862040000000000 0.838954000000000
H -4.535326000000000 1.401991000000000 0.418067000000000
H -4.220402000000000 1.142831000000000 2.158991000000000
H -4.457230000000000 -0.256416000000000 1.082456000000000
H -1.016945000000000 2.763751000000000 -2.591170000000000
H -1.951635000000000 3.809854000000000 -1.500579000000000
H -2.038069000000000 2.046210000000000 -1.308282000000000
H 1.296887000000000 -3.199405000000000 1.676263000000000
H 0.748291000000000 -2.478101000000000 0.151988000000000
H -0.447375000000000 -2.966875000000000 1.386513000000000
H 0.148947000000000 -2.585859000000000 -3.102928000000000
H -0.831230000000000 -1.820372000000000 -4.353057000000000
H -0.734082000000000 0.320841000000000 -3.241999000000000
H 0.868141000000000 -0.310826000000000 -2.872130000000000
H -2.085421000000000 -3.270249000000000 -2.363444000000000
H -2.769853000000000 -1.723031000000000 -2.864638000000000
H 1.996500000000000 1.623149000000000 1.184661000000000
H 2.717477000000000 2.340769000000000 -0.236247000000000
H 1.068659000000000 1.355685000000000 -1.718491000000000
H -0.145528000000000 1.126291000000000 1.626000000000000
38

R5_SMe1_P1_0_1 charge=0 multiplicity=1 B3LYP_D3BJ_TZVPD=-1318.520329515462, CCSDT_SMD_chcl3=-1316.778577374256, CCSDT=-1316.755644181575, B3LYP_D3BJ_TZVPD_SMD_chcl3=-1318.544381891156, B3LYP_TZVP_CPCM_chcl3_freq=0.2519936, B3LYP_TZVPD_CPCM_chcl3=-1318.538814153882, M062X_TZVPD_SMD_chcl3=-1318.638673816867, M062X_TZVPD=-1318.614343677565, COSMORS=-0.0010525259415146003, wB97MV_TZVPD_CPCM_chcl3=-1318.68039879414, wB97MV_TZVPD_SMD_chcl3=-1318.686119316332, wB97MV_TZVPD=-1318.661875079282, M062X_TZVPD_CPCM_chcl3=-1318.633187462986
O 4.007411000000000 -0.373197000000000 0.149294000000000
C 3.088440000000000 0.548096000000000 0.457763000000000
H 5.959594000000000 -0.903412000000000 0.149436000000000
H 5.681678000000000 0.852327000000000 -0.047918000000000
C 0.619499000000000 0.595605000000000 0.947862000000000
C 1.713454000000000 0.144409000000000 -0.021042000000000
O -0.267826000000000 -2.175669000000000 2.255086000000000
O 3.338777000000000 1.587741000000000 1.025267000000000
C 5.373617000000000 -0.050347000000000 0.477869000000000
H 5.478174000000000 0.096553000000000 1.551743000000000
H 1.694769000000000 -0.936558000000000 -0.149726000000000
S 1.504881000000000 0.761912000000000 -1.738865000000000
C -0.766281000000000 0.228357000000000 0.485502000000000
H -1.431719000000000 2.200766000000000 0.635870000000000
H -5.693688000000000 1.633013000000000 0.493887000000000
C -0.974061000000000 -1.189933000000000 0.167253000000000
H 2.279609000000000 2.929021000000000 -0.968136000000000
H 1.385264000000000 2.983448000000000 -2.501789000000000
C -1.708080000000000 1.179781000000000 0.406968000000000
C -1.372825000000000 -1.712702000000000 -1.000811000000000
H -0.100684000000000 -4.233363000000000 0.422054000000000
C -1.433983000000000 -3.206464000000000 -1.002429000000000
H -1.628019000000000 -1.123909000000000 -1.871207000000000
C -0.989816000000000 -3.600954000000000 0.410953000000000
H -2.448221000000000 -3.542354000000000 -1.236607000000000
H -0.786216000000000 -3.619077000000000 -1.780597000000000
C -0.678560000000000 -2.288586000000000 1.114612000000000
H -1.766882000000000 -4.125216000000000 0.969865000000000
C 1.405904000000000 2.557267000000000 -1.499501000000000
H 0.495335000000000 2.837917000000000 -0.974043000000000
H -5.323767000000000 1.744803000000000 -1.242153000000000
H -5.451097000000000 3.234718000000000 -0.261990000000000
C -3.131603000000000 0.995000000000000 0.075411000000000
O -3.725623000000000 -0.042727000000000 -0.125453000000000
C -5.146659000000000 2.192045000000000 -0.264573000000000
O -3.740869000000000 2.200026000000000 0.036070000000000
H 0.803940000000000 0.099375000000000 1.905562000000000
H 0.689124000000000 1.667563000000000 1.117969000000000
41
R6_SMe6_cyc4SP1_0_1 charge=0 multiplicity=1 B3LYP_D3BJ_TZVPD=-1357.820579304387, B3LYP_D3BJ_TZVPD_SMD_chcl3=-1357.845585433882, B3LYP_TZVP_CPCM_chcl3_freq=0.28376444, B3LYP_TZVPD_CPCM_chcl3=-1357.838133284888, M062X_TZVPD_SMD_chcl3=-1357.952819221301, M062X_TZVPD=-1357.926944565854, COSMORS=-0.0042961423076763, wB97MV_TZVPD_CPCM_chcl3=-1357.987148259681, wB97MV_TZVPD_SMD_chcl3=-1357.994788009227, wB97MV_TZVPD=-1357.969149573947, M062X_TZVPD_CPCM_chcl3=-1357.945378160377
O -4.168487000000000 1.643189000000000 0.496233000000000
C -2.950795000000000 1.524248000000000 -0.055496000000000
C -1.367553000000000 -1.884243000000000 0.000204000000000
H -5.158014000000000 2.665615000000000 -1.028633000000000
C -0.505525000000000 1.205570000000000 0.487985000000000
C -1.883041000000000 1.438278000000000 1.016719000000000
H -5.344190000000000 0.896409000000000 -1.056884000000000
O -2.761512000000000 1.503069000000000 -1.248809000000000
C -5.276676000000000 1.773524000000000 -0.415182000000000
H -6.160026000000000 1.856027000000000 0.211282000000000
H -3.695270000000000 -2.969423000000000 -0.687780000000000
H -0.787419000000000 -1.288526000000000 1.991241000000000
C 0.152070000000000 0.042836000000000 0.502949000000000
H -3.475049000000000 -1.431531000000000 -1.564479000000000
C -0.355436000000000 -1.312448000000000 0.991986000000000
C 1.506052000000000 -0.236490000000000 0.054739000000000
C 2.460826000000000 0.662969000000000 -0.647985000000000
H -4.570740000000000 -1.503290000000000 -0.153264000000000
S 3.097586000000000 1.917045000000000 0.554924000000000
C 1.807115000000000 -1.503583000000000 0.380366000000000
H 4.974297000000000 -1.747782000000000 -0.803474000000000
H 1.935496000000000 1.240569000000000 -1.412175000000000
C 3.076246000000000 -2.217067000000000 0.117722000000000
O 0.795317000000000 -2.173628000000000 1.005510000000000
H 4.379368000000000 0.494383000000000 -1.638682000000000
C 4.148721000000000 -1.213885000000000 -0.331068000000000
H 3.391903000000000 -2.759172000000000 1.013051000000000
H 2.896833000000000 -2.973303000000000 -0.655351000000000
C 3.583260000000000 -0.164870000000000 -1.290553000000000
H 4.554505000000000 -0.705580000000000 0.546882000000000
H 3.169345000000000 -0.661284000000000 -2.174930000000000
C 3.718307000000000 3.180806000000000 -0.589903000000000
H 4.089773000000000 4.003783000000000 0.019491000000000
H 4.534274000000000 2.798104000000000 -1.201764000000000
H 2.914058000000000 3.544349000000000 -1.229693000000000
O -2.605494000000000 -1.508786000000000 0.325863000000000
O -1.081449000000000 -2.552393000000000 -0.959984000000000
C -3.654651000000000 -1.886105000000000 -0.592008000000000
H 0.007810000000000 2.065116000000000 0.074398000000000
H -1.922782000000000 2.384641000000000 1.566016000000000
H -2.193109000000000 0.664768000000000 1.719910000000000
41
R6_SMe6H_cyc4RTS_0_1 charge=0 multiplicity=1 B3LYP_D3BJ_TZVPD=-1357.734066396468, B3LYP_D3BJ_TZVPD_SMD_chcl3=-1357.764261568357, B3LYP_TZVP_CPCM_chcl3_freq=0.2810108, B3LYP_TZVPD_CPCM_chcl3=-1357.758519576894, M062X_TZVPD_SMD_chcl3=-1357.851690020997, M062X_TZVPD=-1357.819911891159, COSMORS=-0.002330403062325126, wB97MV_TZVPD_CPCM_chcl3=-1357.886448850848, wB97MV_TZVPD_SMD_chcl3=-1357.892321392565, wB97MV_TZVPD=-1357.860777946865, M062X_TZVPD_CPCM_chcl3=-1357.84599165743
O 4.613907000000000 0.611688000000000 -0.217499000000000
C 3.469542000000000 1.128641000000000 -0.641432000000000
C -2.025749000000000 2.340905000000000 0.442154000000000
H 4.001504000000000 -0.471476000000000 1.454814000000000
C 1.056076000000000 1.244798000000000 -0.884726000000000
C 2.222429000000000 0.592538000000000 -0.448926000000000
H 4.190555000000000 -1.420442000000000 -0.050144000000000
O 3.646167000000000 2.265197000000000 -1.311541000000000
C 4.594643000000000 -0.606612000000000 0.551009000000000
H 5.631858000000000 -0.802318000000000 0.803189000000000
H -3.814524000000000 1.810155000000000 2.325591000000000
H -0.970843000000000 2.773320000000000 -1.360142000000000
C -0.266283000000000 0.835406000000000 -0.700979000000000
H -2.560229000000000 2.877205000000000 3.000912000000000
C -1.147390000000000 1.948477000000000 -0.677850000000000
C -0.852921000000000 -0.458017000000000 -0.547598000000000
C -0.071644000000000 -1.722459000000000 -0.376717000000000
H -2.546752000000000 1.131559000000000 3.388345000000000
S 0.310709000000000 -1.904149000000000 1.431033000000000
C -2.197023000000000 -0.448482000000000 -0.916112000000000
H -2.745072000000000 -3.862082000000000 -0.978243000000000
H 0.901563000000000 -1.636640000000000 -0.862346000000000
C -2.949899000000000 -1.726364000000000 -1.203423000000000
O -2.830335000000000 0.648396000000000 -1.118323000000000
H -0.298091000000000 -3.860301000000000 -0.615541000000000
C -2.267477000000000 -2.953297000000000 -0.606382000000000
H -3.968352000000000 -1.615239000000000 -0.826027000000000
H -3.029600000000000 -1.825692000000000 -2.292070000000000
C -0.786633000000000 -2.945758000000000 -0.954152000000000
H -2.383992000000000 -2.950025000000000 0.480681000000000
H -0.672373000000000 -2.912205000000000 -2.043572000000000
C 1.549866000000000 -3.232034000000000 1.402424000000000
H 2.360852000000000 -2.983145000000000 0.717118000000000
H 1.951224000000000 -3.315318000000000 2.411782000000000
H 1.110328000000000 -4.186707000000000 1.117183000000000
O -1.911334000000000 1.559431000000000 1.520643000000000
O -2.745293000000000 3.318040000000000 0.392977000000000
C -2.770108000000000 1.875008000000000 2.628607000000000
H 1.180606000000000 2.236431000000000 -1.307339000000000
H 2.148706000000000 -0.318730000000000 0.119112000000000
H 4.586726000000000 2.502837000000000 -1.311889000000000
37
R5_SMe4_TS_-1_1 charge=-1 multiplicity=1 wB97MV_TZVPD_SMD_chcl3=-1318.159070577171, B3LYP_D3BJ_TZVPD=-1317.958646957744, CCSDT_SMD_chcl3=-1316.248300995889, CCSDT=-1316.177979653547, M062X_TZVPD=-1318.044456699308, COSMORS=-0.008800807363467103, B3LYP_TZVP_CPCM_chcl3_freq=0.23760843, B3LYP_TZVPD_CPCM_chcl3=-1318.027623678229, M062X_TZVPD_CPCM_chcl3=-1318.115613742535, M062X_TZVPD_SMD_chcl3=-1318.116202268761, wB97MV_TZVPD_CPCM_chcl3=-1318.158277975288, B3LYP_D3BJ_TZVPD_SMD_chcl3=-1318.028410631056, wB97MV_TZVPD=-1318.087936954355
C -2.322296000000000 0.031123000000000 -0.176703000000000
C -1.010090000000000 0.407003000000000 -0.217939000000000
C 0.135578000000000 -0.436279000000000 -0.178805000000000
C 1.433802000000000 0.036405000000000 -0.335359000000000
C 0.005478000000000 -1.875146000000000 0.096041000000000
C -0.732367000000000 -2.468027000000000 1.052824000000000
C 0.713576000000000 -2.935951000000000 -0.664787000000000
O 1.444307000000000 -2.784722000000000 -1.627454000000000
C -0.632658000000000 -3.961984000000000 1.075152000000000
C 0.359598000000000 -4.281426000000000 -0.047900000000000
C -4.135180000000000 3.204731000000000 -0.467326000000000
O -3.067638000000000 2.259036000000000 -0.362996000000000
O -4.611846000000000 0.618236000000000 -0.266179000000000
O 1.144971000000000 2.412822000000000 -0.699851000000000
C 3.604200000000000 2.667403000000000 -1.627110000000000
O 3.060007000000000 1.388163000000000 -1.282873000000000
C 1.813717000000000 1.397911000000000 -0.751663000000000
C -3.429820000000000 0.949053000000000 -0.270275000000000
H -2.617620000000000 -1.004434000000000 -0.084007000000000
H -0.799919000000000 1.460035000000000 -0.335515000000000
H 2.207490000000000 -0.690943000000000 -0.507976000000000
H -1.347423000000000 -1.930896000000000 1.762746000000000
H -0.291973000000000 -4.312910000000000 2.053322000000000
H -1.612263000000000 -4.420266000000000 0.908447000000000
H 1.274604000000000 -4.751528000000000 0.318215000000000
H -0.052439000000000 -4.933928000000000 -0.819208000000000
H -4.732454000000000 3.020373000000000 -1.361002000000000
H -3.657887000000000 4.179749000000000 -0.530218000000000
H -4.782959000000000 3.159252000000000 0.408789000000000
H 4.598630000000000 2.468063000000000 -2.018055000000000
H 3.665498000000000 3.302215000000000 -0.742434000000000
H 2.991748000000000 3.159461000000000 -2.382483000000000
S 2.488059000000000 0.513442000000000 1.889660000000000
C 0.905147000000000 0.924548000000000 2.664243000000000
H 0.092267000000000 0.584319000000000 2.011263000000000
H 0.797302000000000 0.414213000000000 3.622825000000000
H 0.792921000000000 1.998524000000000 2.817941000000000
41
R6_SMe1_P1A_0_1 charge=0 multiplicity=1 B3LYP_D3BJ_TZVPD=-1357.824043360698, B3LYP_D3BJ_TZVPD_SMD_chcl3=-1357.850532742014, B3LYP_TZVP_CPCM_chcl3_freq=0.28046872, B3LYP_TZVPD_CPCM_chcl3=-1357.843961278196, M062X_TZVPD_SMD_chcl3=-1357.949068795418, M062X_TZVPD=-1357.922292737966, COSMORS=-0.0036597216375425, wB97MV_TZVPD_CPCM_chcl3=-1357.98667841874, wB97MV_TZVPD_SMD_chcl3=-1357.99341714636, wB97MV_TZVPD=-1357.966740995815, M062X_TZVPD_CPCM_chcl3=-1357.942552026721
O 0.277483000000000 -2.146472000000000 1.548872000000000
C 0.088603000000000 -1.890392000000000 0.247689000000000
O -0.851754000000000 -2.307043000000000 -0.387011000000000
H -1.689193000000000 -2.519951000000000 2.129138000000000
H -0.738483000000000 -3.960911000000000 1.696189000000000
C 1.210726000000000 -1.039223000000000 -0.312497000000000
C 0.678205000000000 0.101765000000000 -1.191826000000000
C -0.707478000000000 -2.987332000000000 2.182780000000000
H -0.385335000000000 -3.084128000000000 3.215139000000000
H 1.808985000000000 -0.638911000000000 0.503396000000000
S 2.306020000000000 -2.093020000000000 -1.335720000000000
H 0.004045000000000 -0.310439000000000 -1.938826000000000
C -0.053454000000000 1.118937000000000 -0.352114000000000
H 1.522679000000000 0.562463000000000 -1.694486000000000
C 3.029430000000000 -3.162673000000000 -0.060647000000000
H 3.788868000000000 -3.760296000000000 -0.563213000000000
H 3.499207000000000 -2.562788000000000 0.717870000000000
H 2.284069000000000 -3.825843000000000 0.375122000000000
C 0.515981000000000 2.216794000000000 0.171089000000000
C -1.469270000000000 0.827734000000000 -0.028194000000000
C 1.932876000000000 2.593350000000000 0.053366000000000
H -0.096806000000000 2.913280000000000 0.727484000000000
C -1.885131000000000 0.753719000000000 1.248773000000000
H -1.155625000000000 0.899314000000000 2.038644000000000
O -2.123340000000000 0.793091000000000 -2.298907000000000
C -3.280845000000000 0.437998000000000 1.669978000000000
H -3.631435000000000 -1.074105000000000 -0.618323000000000
H -4.454964000000000 0.152134000000000 -1.586589000000000
C -4.290887000000000 0.625879000000000 0.542666000000000
H -3.296150000000000 -0.601677000000000 2.024737000000000
H -3.541221000000000 1.047933000000000 2.539014000000000
C -3.769037000000000 0.005898000000000 -0.752678000000000
H -5.251122000000000 0.183883000000000 0.811951000000000
H -4.460219000000000 1.695164000000000 0.386126000000000
C -2.422600000000000 0.567561000000000 -1.137663000000000
O 2.115711000000000 3.851460000000000 0.503930000000000
O 2.845069000000000 1.907721000000000 -0.362417000000000
C 3.463413000000000 4.351857000000000 0.461292000000000
H 3.828731000000000 4.370307000000000 -0.564869000000000
H 3.413120000000000 5.358666000000000 0.865695000000000
H 4.118923000000000 3.728093000000000 1.068113000000000
44
R5_SMe4_P1S_SMe6RR charge=0 multiplicity=1 B3LYP_D3BJ_TZVPD=-1757.201438330715, B3LYP_D3BJ_TZVPD_SMD_chcl3=-1757.228112879833, B3LYP_TZVP_CPCM_chcl3_freq=0.30090665, B3LYP_TZVPD_CPCM_chcl3=-1757.221600174175, M062X_TZVPD_SMD_chcl3=-1757.351863452865, M062X_TZVPD=-1757.325234392001, COSMORS=-0.003090264099818111, wB97MV_TZVPD_CPCM_chcl3=-1757.383743673786, wB97MV_TZVPD_SMD_chcl3=-1757.390427776965, wB97MV_TZVPD=-1757.363791287529, M062X_TZVPD_CPCM_chcl3=-1757.34543426908
C -5.148873000000000 -2.628286000000000 0.954847000000000
C -2.432870000000000 4.202592000000000 -0.288601000000000
C 4.035599000000000 -0.956407000000000 1.383851000000000
C 2.006311000000000 2.410649000000000 1.606628000000000
C -1.096710000000000 -0.417928000000000 -0.359396000000000
C 1.741012000000000 -2.467015000000000 -2.207452000000000
C 2.432967000000000 -2.793122000000000 -0.879349000000000
C -1.926302000000000 -0.938375000000000 0.540852000000000
C 0.224265000000000 0.205470000000000 -0.023808000000000
C 1.263037000000000 -1.036771000000000 -2.059918000000000
C 1.674822000000000 -1.932958000000000 0.143015000000000
C -3.207349000000000 -1.551642000000000 0.138497000000000
C 1.385430000000000 -0.621629000000000 -0.597842000000000
C 0.251176000000000 1.665804000000000 -0.515208000000000
C -0.859301000000000 2.486171000000000 0.102183000000000
O 0.856907000000000 -0.330393000000000 -2.955417000000000
O -3.625697000000000 -1.647539000000000 -0.996073000000000
O -1.190629000000000 2.424393000000000 1.264760000000000
O -3.876744000000000 -2.005818000000000 1.212418000000000
O -1.413058000000000 3.314371000000000 -0.788818000000000
S 2.451586000000000 -1.748278000000000 1.777468000000000
S 1.825110000000000 2.552598000000000 -0.193126000000000
H -5.826072000000000 -1.920138000000000 0.478993000000000
H -5.529544000000000 -2.925952000000000 1.927457000000000
H -5.020319000000000 -3.497614000000000 0.311198000000000
H -2.757800000000000 4.782434000000000 -1.147123000000000
H -2.018172000000000 4.853386000000000 0.479895000000000
H -3.261369000000000 3.628730000000000 0.123501000000000
H 4.672882000000000 -1.606852000000000 0.787862000000000
H 4.518543000000000 -0.769473000000000 2.342082000000000
H 3.892517000000000 -0.005028000000000 0.873713000000000
H 2.107279000000000 1.371623000000000 1.914631000000000
H 1.168456000000000 2.874853000000000 2.121633000000000
H 2.924439000000000 2.945746000000000 1.846271000000000
H -1.378757000000000 -0.445975000000000 -1.407037000000000
H 0.842066000000000 -3.080072000000000 -2.339871000000000
H 2.356894000000000 -2.591295000000000 -3.097722000000000
H 3.479505000000000 -2.487867000000000 -0.924498000000000
H 2.401268000000000 -3.850214000000000 -0.622382000000000
H -1.696224000000000 -0.926630000000000 1.598632000000000
H 0.337738000000000 0.207277000000000 1.060707000000000
H 0.726612000000000 -2.421371000000000 0.383764000000000
H 2.284291000000000 0.006675000000000 -0.573786000000000
H 0.162650000000000 1.697803000000000 -1.599491000000000
55
Sulf_SMe1_P2_0_1 charge=0 multiplicity=1 B3LYP_D3BJ_TZVPD=-2138.406128315123, B3LYP_D3BJ_TZVPD_SMD_chcl3=-2138.442338515355, B3LYP_TZVP_CPCM_chcl3_freq=0.37047585, B3LYP_TZVPD_CPCM_chcl3=-2138.432149192209, M062X_TZVPD_SMD_chcl3=-2138.593787325421, M062X_TZVPD=-2138.555652183096, COSMORS=-0.008816759313857826, wB97MV_TZVPD_CPCM_chcl3=-2138.659785232268, wB97MV_TZVPD_SMD_chcl3=-2138.670408468201, wB97MV_TZVPD=-2138.633472586762, M062X_TZVPD_CPCM_chcl3=-2138.583278372308
C 1.255402000000000 3.126644000000000 1.335611000000000
C 1.378381000000000 3.073220000000000 2.718177000000000
C 1.971092000000000 1.971463000000000 3.323221000000000
C 2.455980000000000 0.919795000000000 2.549407000000000
C 2.344508000000000 0.966831000000000 1.168314000000000
C 1.736899000000000 2.070082000000000 0.576782000000000
O 2.836867000000000 1.714742000000000 -1.772243000000000
O 0.993903000000000 3.378013000000000 -1.574124000000000
S 1.564528000000000 2.102744000000000 -1.189194000000000
C 3.368023000000000 -1.954996000000000 -2.020205000000000
S 1.721296000000000 -2.507482000000000 -2.548382000000000
C -1.346453000000000 0.639919000000000 1.459567000000000
C -1.922138000000000 1.363798000000000 2.492222000000000
C -2.419959000000000 2.641265000000000 2.255036000000000
C -2.330710000000000 3.194809000000000 0.983643000000000
C -1.732498000000000 2.481746000000000 -0.046899000000000
C -1.233853000000000 1.201377000000000 0.186728000000000
C 2.390161000000000 -4.539931000000000 1.443924000000000
O 2.041243000000000 -3.391504000000000 0.643379000000000
O 0.256429000000000 -4.499231000000000 -0.163128000000000
O -3.524315000000000 -1.759578000000000 0.575704000000000
C -5.308812000000000 0.160300000000000 1.057284000000000
O -4.476290000000000 0.152595000000000 -0.119811000000000
C -3.614061000000000 -0.863262000000000 -0.229249000000000
C 0.957716000000000 -3.516220000000000 -0.133749000000000
C -2.792589000000000 -0.770944000000000 -1.503811000000000
C 0.473946000000000 0.771071000000000 -1.578947000000000
C -0.624826000000000 0.409337000000000 -0.907105000000000
C -1.303985000000000 -0.860617000000000 -1.253344000000000
C -0.675020000000000 -2.039422000000000 -1.296513000000000
C 0.761930000000000 -2.274204000000000 -0.978873000000000
H 0.781560000000000 3.966231000000000 0.850086000000000
H 0.999622000000000 3.887958000000000 3.320810000000000
H 2.055544000000000 1.929152000000000 4.401528000000000
H 2.920725000000000 0.064412000000000 3.021793000000000
H 2.721247000000000 0.156391000000000 0.559965000000000
H 3.752090000000000 -2.583791000000000 -1.220272000000000
H 3.337132000000000 -0.911266000000000 -1.710488000000000
H 4.008083000000000 -2.051127000000000 -2.896684000000000
H -0.971304000000000 -0.359181000000000 1.637118000000000
H -1.988539000000000 0.929234000000000 3.481174000000000
H -2.879306000000000 3.201355000000000 3.059515000000000
H -2.721744000000000 4.185968000000000 0.793056000000000
H -1.651856000000000 2.913459000000000 -1.034308000000000
H 1.583051000000000 -4.773650000000000 2.136301000000000
H 2.583933000000000 -5.397999000000000 0.802222000000000
H 3.287683000000000 -4.254029000000000 1.983870000000000
H -4.687590000000000 0.198227000000000 1.950220000000000
H -5.936944000000000 -0.728942000000000 1.080890000000000
H -5.915598000000000 1.057055000000000 0.976292000000000
H -3.099716000000000 -1.613635000000000 -2.125961000000000
H -3.043200000000000 0.148956000000000 -2.029237000000000

H 0.859885000000000 0.184826000000000 -2.403675000000000
H -1.248453000000000 -2.924656000000000 -1.543803000000000
H 1.206158000000000 -1.420567000000000 -0.476644000000000
41
R6_SMe6H_cyc4RE_0_1 charge=0 multiplicity=1 B3LYP_D3BJ_TZVPD_SMD_chcl3=-1357.814242736651, B3LYP_TZVP_CPCM_chcl3_freq=0.28346841, B3LYP_TZVPD_CPCM_chcl3=-1357.80765679219, M062X_TZVPD_SMD_chcl3=-1357.922178039064, M062X_TZVPD=-1357.895791495489, COSMORS=-0.00408932471310067, wB97MV_TZVPD_CPCM_chcl3=-1357.952641027099, wB97MV_TZVPD_SMD_chcl3=-1357.959417073025, wB97MV_TZVPD=-1357.93338409799, M062X_TZVPD_CPCM_chcl3=-1357.915537690514
O 4.496926000000000 0.083920000000000 -0.122520000000000
C 3.402346000000000 -0.590687000000000 0.280503000000000
C -1.609015000000000 -2.549994000000000 -0.106798000000000
H 3.890004000000000 2.065937000000000 0.060642000000000
C 1.011202000000000 -0.972065000000000 0.648852000000000
C 2.127474000000000 -0.150874000000000 0.258663000000000
H 5.309292000000000 1.727372000000000 -0.965531000000000
O 3.737740000000000 -1.817644000000000 0.733988000000000
C 4.317426000000000 1.388176000000000 -0.680526000000000
H 3.667974000000000 1.343950000000000 -1.556553000000000
H -0.064649000000000 -5.296986000000000 -0.820846000000000
H -1.224183000000000 -2.170722000000000 1.972911000000000
C -0.284057000000000 -0.605789000000000 0.737293000000000
H -0.466965000000000 -4.014249000000000 -2.001035000000000
C -1.402180000000000 -1.596809000000000 1.063721000000000
C -0.977223000000000 0.665082000000000 0.591071000000000
C -0.441794000000000 2.019605000000000 0.263314000000000
H -1.770274000000000 -4.962133000000000 -1.242182000000000
S -0.082148000000000 2.091566000000000 -1.549909000000000
C -2.274608000000000 0.476378000000000 0.895088000000000
H -3.521696000000000 3.618140000000000 0.604459000000000
H 0.519449000000000 2.173238000000000 0.756357000000000
C -3.350629000000000 1.491079000000000 0.949604000000000
O -2.588769000000000 -0.807907000000000 1.238473000000000
H -1.117248000000000 4.075283000000000 0.303841000000000
C -2.863648000000000 2.798352000000000 0.313023000000000
H -4.241791000000000 1.112612000000000 0.441656000000000
H -3.634782000000000 1.648972000000000 1.997049000000000
C -1.422511000000000 3.111552000000000 0.713154000000000
H -2.918465000000000 2.711535000000000 -0.774707000000000
H -1.354519000000000 3.193081000000000 1.803607000000000
C 1.011742000000000 3.538641000000000 -1.610299000000000
H 0.487616000000000 4.448618000000000 -1.321414000000000
H 1.876403000000000 3.389536000000000 -0.963335000000000
H 1.350194000000000 3.636999000000000 -2.640972000000000
O -0.803220000000000 -3.608347000000000 0.014965000000000
O -2.355548000000000 -2.358200000000000 -1.033884000000000
C -0.782060000000000 -4.529523000000000 -1.094884000000000
H 1.239117000000000 -2.007388000000000 0.876080000000000
H 1.946802000000000 0.840433000000000 -0.118366000000000
H 4.682337000000000 -1.959186000000000 0.576693000000000
37
R5_SMe6_cyc4RZ_-1_1 charge=-1 multiplicity=1 wB97MV_TZVPD_SMD_chcl3=-1318.158043485428, B3LYP_TZVP_CPCM_chcl3_freq=0.24108437, B3LYP_TZVPD_CPCM_chcl3=-1318.007445781656, M062X_TZVPD=-1318.039899593063, COSMORS=-0.00978353356201330З, M062X_TZVPD_CPCM_chcl3=-1318.11250229271, M062X_TZVPD_SMD_chcl3=-1318.114841372693, wB97MV_TZVPD_CPCM_chcl3=-1318.155504950268, B3LYP_D3BJ_TZVPD_SMD_chcl3=-1318.009679893188, wB97MV_TZVPD=-1318.084123497214
C -2.474224000000000 0.122930000000000 -0.409211000000000
C -1.173324000000000 0.665663000000000 -0.423856000000000
C 0.007729000000000 0.051172000000000 -0.719057000000000
C 0.203124000000000 -1.414397000000000 -1.127008000000000
C 1.339208000000000 0.594433000000000 -0.801071000000000
C 2.068126000000000 1.844726000000000 -0.468443000000000
C 2.177741000000000 -0.331810000000000 -1.270601000000000
O 1.591289000000000 -1.540332000000000 -1.556399000000000
C 3.495562000000000 1.579723000000000 -1.053091000000000
C 3.600807000000000 0.060373000000000 -1.384852000000000
C -5.974084000000000 0.899526000000000 0.137322000000000
O -4.785379000000000 0.178742000000000 -0.158986000000000
O -3.650329000000000 2.121685000000000 0.177214000000000
O -1.111392000000000 -2.924127000000000 0.218318000000000
C 0.732374000000000 -3.122513000000000 2.129857000000000
O 0.953392000000000 -2.377473000000000 0.919654000000000
C -0.064918000000000 -2.339623000000000 0.052374000000000
C -3.597172000000000 0.914645000000000 -0.109872000000000
H -2.638280000000000 -0.925547000000000 -0.613904000000000
H -1.100763000000000 1.720587000000000 -0.170755000000000
H -0.448137000000000 -1.737512000000000 -1.943139000000000
H 1.622987000000000 2.743744000000000 -0.899526000000000
H 3.609882000000000 2.161366000000000 -1.968728000000000
H 4.272673000000000 1.903420000000000 -0.361891000000000
H 4.021464000000000 -0.110609000000000 -2.380618000000000
H 4.238118000000000 -0.479016000000000 -0.673665000000000
H -6.131066000000000 1.722055000000000 -0.564126000000000
H -5.953868000000000 1.309597000000000 1.149628000000000
H -6.789407000000000 0.182333000000000 0.049280000000000
H 0.535764000000000 -4.169306000000000 1.901000000000000
H 1.649484000000000 -3.023384000000000 2.703440000000000
H -0.109952000000000 -2.704879000000000 2.679972000000000
S 2.043832000000000 2.247722000000000 1.339794000000000
C 2.626303000000000 0.684288000000000 2.050621000000000
H 2.576456000000000 0.796261000000000 3.133093000000000
H 1.984267000000000 -0.137404000000000 1.739937000000000
H 3.656734000000000 0.477673000000000 1.761331000000000
44
R5_SMe4_P2_SMe1RR charge=0 multiplicity=1 B3LYP_D3BJ_TZVPD=-1757.192315160945, B3LYP_D3BJ_TZVPD_SMD_chcl3=-1757.221035003861, B3LYP_TZVP_CPCM_chcl3_freq=0.30028278, B3LYP_TZVPD_CPCM_chcl3=-1757.21384554415, M062X_TZVPD_SMD_chcl3=-1757.346776626656, M062X_TZVPD=-1757.317750529324, COSMORS=-0.0024175093169062395, wB97MV_TZVPD_CPCM_chcl3=-1757.377711340476, wB97MV_TZVPD_SMD_chcl3=-1757.384986018094, wB97MV_TZVPD=-1757.356078837639, M062X_TZVPD_CPCM_chcl3=-1757.33978634611
C -4.825347000000000 -0.504699000000000 1.075109000000000
C 4.351087000000000 -0.191772000000000 -1.976730000000000
C -0.687878000000000 -1.088129000000000 -3.265418000000000
C 0.830617000000000 -1.029496000000000 3.321132000000000
C 0.015548000000000 3.381706000000000 -1.043966000000000
C 0.654918000000000 1.995241000000000 -1.228784000000000
C -1.174414000000000 3.127007000000000 -0.111806000000000
C -0.201072000000000 -1.052504000000000 -0.040739000000000
C 0.525707000000000 1.353455000000000 0.165183000000000
C 0.709941000000000 -0.133651000000000 0.281020000000000
C -0.778446000000000 1.922934000000000 0.723106000000000
C -1.550271000000000 -0.795091000000000 -0.624516000000000
C 2.024611000000000 -0.598249000000000 0.864914000000000
C -2.664125000000000 -1.119362000000000 0.357156000000000
C 3.188482000000000 -0.028237000000000 0.072313000000000
O -1.382672000000000 1.491730000000000 1.677620000000000
O -2.624543000000000 -2.017947000000000 1.162904000000000
O 3.873581000000000 0.907840000000000 0.408268000000000
O -3.698574000000000 -0.292476000000000 0.199647000000000
O 3.330113000000000 -0.682896000000000 -1.083245000000000
S -1.845080000000000 -1.862898000000000 -2.104996000000000
S 2.216514000000000 -0.094674000000000 2.618160000000000
H -5.550641000000000 0.255278000000000 0.801269000000000
H -4.517168000000000 -0.386509000000000 2.112609000000000
H -5.235609000000000 -1.501726000000000 0.922733000000000
H 5.327308000000000 -0.247479000000000 -1.498151000000000
H 4.136318000000000 0.838563000000000 -2.256732000000000
H 4.311446000000000 -0.841699000000000 -2.845405000000000
H -0.721439000000000 -1.689249000000000 -4.173145000000000
H 0.324271000000000 -1.097833000000000 -2.863992000000000
H -0.993892000000000 -0.068749000000000 -3.495857000000000
H 0.862026000000000 -0.852045000000000 4.395317000000000
H 0.945222000000000 -2.095729000000000 3.128304000000000
H -0.116940000000000 -0.674476000000000 2.921743000000000
H -0.278694000000000 3.832840000000000 -1.990640000000000
H 0.729382000000000 4.053122000000000 -0.561525000000000
H 1.690320000000000 2.047760000000000 -1.562567000000000
H 0.098916000000000 1.413223000000000 -1.964709000000000
H -2.065396000000000 2.833861000000000 -0.678083000000000
H -1.458078000000000 3.965341000000000 0.524218000000000
H 0.030517000000000 -2.098064000000000 0.122288000000000
H 1.278924000000000 1.830947000000000 0.807261000000000
H -1.680484000000000 0.230273000000000 -0.956793000000000
H 2.078330000000000 -1.684803000000000 0.809146000000000
40
R6_SMe2_-1_1 charge=-1 multiplicity=1 wB97MV_TZVPD_SMD_chcl3=-1357.469508503741, B3LYP_D3BJ_TZVPD=-1357.252803950921, B3LYP_TZVP_CPCM_chcl3_freq=0.26539247, B3LYP_TZVPD_CPCM_chcl3=-1357.324090428477, M062X_TZVPD=-1357.349672974967, COSMORS=-0.008964900503500349, M062X_TZVPD_CPCM_chcl3=-1357.424237325874, M062X_TZVPD_SMD_chcl3=-1357.425660195041, wB97MV_TZVPD_CPCM_chcl3=-1357.467765368513, B3LYP_D3BJ_TZVPD_SMD_chcl3=-1357.325553039608, wB97MV_TZVPD=-1357.394191160834
O 3.357771000000000 0.994356000000000 -0.013435000000000
C 2.979727000000000 0.339956000000000 -1.197074000000000
C -2.549968000000000 -1.207432000000000 -0.658699000000000
H 4.131822000000000 2.754972000000000 -0.822456000000000
C 1.207799000000000 -0.801679000000000 0.233732000000000
C 1.936322000000000 -0.563207000000000 -1.034573000000000
H 4.557489000000000 2.353658000000000 0.861468000000000
O 3.589693000000000 0.620765000000000 -2.249722000000000
C 4.402239000000000 1.947637000000000 -0.137831000000000
H 5.327561000000000 1.489945000000000 -0.494603000000000
H -5.135412000000000 -1.849975000000000 -0.476525000000000
H -0.837243000000000 -2.493266000000000 -0.345020000000000
C -0.258646000000000 -0.508711000000000 -0.008375000000000
H -4.896416000000000 -3.281623000000000 -1.519121000000000
C -1.153299000000000 -1.458970000000000 -0.327078000000000
C -0.595732000000000 0.929722000000000 0.101010000000000
C -0.208495000000000 1.823515000000000 -0.825350000000000
H -4.703413000000000 -1.635489000000000 -2.186621000000000
S 1.567660000000000 -2.496390000000000 0.894706000000000
C -1.333751000000000 1.371633000000000 1.304214000000000
H 0.191777000000000 3.700566000000000 1.298577000000000
H 0.287668000000000 1.455969000000000 -1.714443000000000
C -1.769495000000000 2.819944000000000 1.331459000000000
O -1.603167000000000 0.612601000000000 2.223990000000000
H -1.245691000000000 3.576921000000000 -1.390139000000000
C -0.726514000000000 3.742159000000000 0.705104000000000
H -2.704475000000000 2.876409000000000 0.760230000000000
H -1.995791000000000 3.089667000000000 2.363264000000000
C -0.419306000000000 3.298722000000000 -0.721202000000000
H -1.075182000000000 4.776502000000000 0.721632000000000
H 0.462765000000000 3.814259000000000 -1.110636000000000
C 0.617693000000000 -2.511999000000000 2.446911000000000
H -0.454047000000000 -2.507679000000000 2.253296000000000
H 0.886148000000000 -3.421967000000000 2.983371000000000
H 0.877244000000000 -1.646995000000000 3.057974000000000
O -3.178955000000000 -2.372003000000000 -0.970635000000000
O -3.139514000000000 -0.141860000000000 -0.667604000000000
C -4.567905000000000 -2.267123000000000 -1.308533000000000
H 1.566692000000000 -0.110553000000000 0.994278000000000
H 1.579626000000000 -1.065170000000000 -1.923707000000000
44
R5_SMe1_P2A_SMe6RS charge=0 multiplicity=1 B3LYP_D3BJ_TZVPD=-1757.196087488536, B3LYP_D3BJ_TZVPD_SMD_chcl3=-1757.222203192275, B3LYP_TZVP_CPCM_chcl3_freq=0.30160691, B3LYP_TZVPD_CPCM_chcl3=-1757.215842314958, M062X_TZVPD_SMD_chcl3=-1757.347601710775, M062X_TZVPD=-1757.321042612518, COSMORS=-0.003034551793558401, wB97MV_TZVPD_CPCM_chcl3=-1757.378794778574, wB97MV_TZVPD_SMD_chcl3=-1757.38528423695, wB97MV_TZVPD=-1757.358844941706, M062X_TZVPD_CPCM_chcl3=-1757.341389457449
C -2.415619000000000 3.249573000000000 1.832530000000000
C 4.980065000000000 1.796359000000000 -1.132073000000000
C -1.269306000000000 1.117674000000000 -2.930680000000000
C 2.972309000000000 -2.697896000000000 0.614660000000000
C -3.553705000000000 -2.172138000000000 0.360176000000000
C -3.751205000000000 -0.723621000000000 -0.094070000000000
C 0.889550000000000 -0.621343000000000 -0.389651000000000
C -0.699229000000000 -0.007666000000000 1.432342000000000
C -0.354446000000000 -0.618368000000000 0.088275000000000
C -2.072008000000000 -2.445189000000000 0.191360000000000
C -2.661190000000000 -0.524846000000000 -1.161922000000000
C 2.080237000000000 -0.014443000000000 0.273307000000000
C -0.897906000000000 1.494339000000000 1.399102000000000
C -1.435445000000000 -1.342461000000000 -0.678825000000000
C 3.167079000000000 0.342083000000000 -0.715045000000000
O -1.464956000000000 -3.375861000000000 0.663825000000000
O -0.056070000000000 2.293204000000000 1.060477000000000
O 3.411289000000000 -0.279119000000000 -1.725424000000000
O -2.111203000000000 1.841485000000000 1.845616000000000
O 3.852871000000000 1.417846000000000 -0.317523000000000
S -2.391063000000000 1.233963000000000 -1.511622000000000
S 2.850456000000000 -1.139133000000000 1.533751000000000
H -3.429666000000000 3.330966000000000 2.212370000000000
H -2.354944000000000 3.630642000000000 0.814219000000000
H -1.720967000000000 3.792623000000000 2.471364000000000
H 5.710943000000000 0.989227000000000 -1.157626000000000
H 5.399209000000000 2.677024000000000 -0.654829000000000
H 4.652798000000000 2.027370000000000 -2.144627000000000
H -1.095619000000000 2.135188000000000 -3.277816000000000
H -1.725682000000000 0.537518000000000 -3.732611000000000
H -0.320073000000000 0.672349000000000 -2.638078000000000
H 3.576352000000000 -2.575047000000000 -0.282374000000000
H 1.983991000000000 -3.076014000000000 0.359934000000000
H 3.463548000000000 -3.398502000000000 1.288803000000000
H -4.082116000000000 -2.867635000000000 -0.301292000000000
H -3.873253000000000 -2.384606000000000 1.380231000000000
H -3.594753000000000 -0.038144000000000 0.740044000000000
H -4.744128000000000 -0.533954000000000 -0.497635000000000
H 1.089641000000000 -1.118440000000000 -1.330325000000000
H 0.122626000000000 -0.193207000000000 2.127872000000000
H -1.584950000000000 -0.469652000000000 1.860291000000000
H -3.005585000000000 -0.982572000000000 -2.091800000000000
H 1.822522000000000 0.870272000000000 0.848460000000000
H -0.958784000000000 -1.835486000000000 -1.526408000000000
44
R5_SMe4_P2_SMe3RR charge=0 multiplicity=1 B3LYP_D3BJ_TZVPD=-1757.18524959172, B3LYP_D3BJ_TZVPD_SMD_chcl3=-1757.211856996195, B3LYP_TZVP_CPCM_chcl3_freq=0.30103122, B3LYP_TZVPD_CPCM_chcl3=-1757.206235585644, M062X_TZVPD_SMD_chcl3=-1757.336718135648, M062X_TZVPD=-1757.309833367125, COSMORS=-0.0036735700340355458, wB97MV_TZVPD_CPCM_chcl3=-1757.368757494007, wB97MV_TZVPD_SMD_chcl3=-1757.374496890999, wB97MV_TZVPD=-1757.34768994166, M062X_TZVPD_CPCM_chcl3=-1757.331242767363
C -5.725968000000000 -0.852606000000000 0.334893000000000
C 3.748858000000000 -2.928579000000000 -0.878909000000000
C 0.179725000000000 -3.082020000000000 1.980338000000000
C -0.186334000000000 -1.288174000000000 -2.706633000000000
C 1.389894000000000 3.248131000000000 1.894332000000000
C 1.019412000000000 1.771686000000000 1.700418000000000
C 0.672512000000000 3.951392000000000 0.743968000000000
C -2.111156000000000 -0.371790000000000 0.035464000000000
C -1.179517000000000 0.539529000000000 -0.244894000000000
C 1.065003000000000 1.566492000000000 0.176401000000000
C 0.657620000000000 2.940873000000000 -0.384658000000000
C -3.538794000000000 -0.008299000000000 0.017115000000000
C 0.787097000000000 -1.012531000000000 0.219270000000000
C 2.251237000000000 -1.290662000000000 -0.067532000000000
C 0.296006000000000 0.334876000000000 -0.354539000000000
O 0.399600000000000 3.196760000000000 -1.537224000000000
O -3.995316000000000 1.088865000000000 -0.227611000000000
O 3.178519000000000 -0.560589000000000 0.191278000000000
O -4.304252000000000 -1.074430000000000 0.316859000000000
O 2.396342000000000 -2.510343000000000 -0.603343000000000
S 0.513612000000000 -1.298334000000000 2.025046000000000
S 0.625251000000000 0.253525000000000 -2.209394000000000
H -6.071623000000000 -0.535302000000000 -0.648303000000000
H -5.981191000000000 -0.092852000000000 1.072494000000000
H -6.166548000000000 -1.808403000000000 0.603300000000000
H 4.329173000000000 -2.951286000000000 0.042192000000000
H 4.212795000000000 -2.245996000000000 -1.588950000000000
H 3.662985000000000 -3.924145000000000 -1.303455000000000
H 1.034283000000000 -3.631474000000000 1.590205000000000
H -0.708336000000000 -3.295834000000000 1.385692000000000
H 0.001791000000000 -3.379968000000000 3.012946000000000
H 0.361289000000000 -2.171895000000000 -2.386851000000000
H -0.203731000000000 -1.253930000000000 -3.795608000000000
H -1.213247000000000 -1.319489000000000 -2.342682000000000
H 2.470774000000000 3.374393000000000 1.795895000000000
H 1.097343000000000 3.623321000000000 2.874047000000000
H 0.008055000000000 1.590439000000000 2.072228000000000
H 1.697650000000000 1.103002000000000 2.224701000000000
H 1.105221000000000 4.896591000000000 0.418077000000000
H -0.377103000000000 4.139321000000000 1.000342000000000
H -1.883553000000000 -1.395058000000000 0.292016000000000
H -1.518294000000000 1.540515000000000 -0.485866000000000
H 2.114159000000000 1.453771000000000 -0.114226000000000
H 0.208010000000000 -1.797376000000000 -0.255013000000000
44
R5_SMe4_P3_SMe6SSS_P2 charge=0 multiplicity=1 B3LYP_D3BJ_TZVPD=-1757.200621643829, B3LYP_D3BJ_TZVPD_SMD_chcl3=-1757.226537868464, B3LYP_TZVP_CPCM_chcl3_freq=0.30087459, B3LYP_TZVPD_CPCM_chcl3=-1757.220479093646, M062X_TZVPD_SMD_chcl3=-1757.350052803644, M062X_TZVPD=-1757.324138762622, COSMORS=-0.0031001763007601985, wB97MV_TZVPD_CPCM_chcl3=-1757.382467895787, wB97MV_TZVPD_SMD_chcl3=-1757.388696107449, wB97MV_TZVPD=-1757.36278080072, M062X_TZVPD_CPCM_chcl3=-1757.344078724522
C -5.844730000000000 0.903874000000000 0.149161000000000
C -0.736039000000000 -4.537961000000000 1.071664000000000
C 3.637288000000000 1.107307000000000 1.727692000000000
C 2.117916000000000 -1.928386000000000 -2.391727000000000
C -1.107039000000000 0.151263000000000 0.200462000000000
C 1.026700000000000 3.539834000000000 -1.110184000000000
C 1.695446000000000 3.368939000000000 0.257355000000000
C -2.277467000000000 0.295028000000000 -0.414690000000000
C 0.191771000000000 -0.100056000000000 -0.504932000000000
C 0.769577000000000 2.128623000000000 -1.599259000000000
C 1.126169000000000 2.038278000000000 0.773341000000000
C -3.514006000000000 0.553222000000000 0.348989000000000
C 1.093092000000000 1.144209000000000 -0.477963000000000
C 0.865325000000000 -1.344535000000000 0.104720000000000
C -0.018316000000000 -2.567104000000000 -0.012942000000000
O 0.367817000000000 1.822803000000000 -2.698151000000000
O -3.595582000000000 0.665313000000000 1.554380000000000
O -0.656410000000000 -2.858484000000000 -0.999304000000000
O -4.571846000000000 0.652844000000000 -0.474282000000000
O 0.013836000000000 -3.306562000000000 1.100493000000000
S 1.921041000000000 1.357207000000000 2.260013000000000
S 2.490776000000000 -1.782270000000000 -0.622264000000000
H -6.563574000000000 0.946553000000000 -0.663779000000000
H -5.820848000000000 1.849693000000000 0.689087000000000
H -6.093339000000000 0.097641000000000 0.838117000000000
H -1.790248000000000 -4.331407000000000 0.893945000000000
H -0.593032000000000 -4.986823000000000 2.049893000000000
H -0.352711000000000 -5.191447000000000 0.289294000000000
H 4.152533000000000 0.678872000000000 2.586539000000000
H 4.114597000000000 2.049175000000000 1.463600000000000
H 3.697721000000000 0.406850000000000 0.896429000000000
H 3.046038000000000 -2.260646000000000 -2.855325000000000
H 1.829650000000000 -0.968966000000000 -2.816738000000000
H 1.339526000000000 -2.667913000000000 -2.565562000000000
H -1.079104000000000 0.218693000000000 1.283634000000000
H 1.601570000000000 4.109686000000000 -1.840147000000000
H 0.047980000000000 4.022293000000000 -1.014942000000000
H 1.491124000000000 4.187379000000000 0.944946000000000
H 2.776847000000000 3.290901000000000 0.135015000000000
H -2.366380000000000 0.225522000000000 -1.491252000000000

H -0.022658000000000 -0.309495000000000 -1.552819000000000
H 0.103621000000000 2.214939000000000 1.114798000000000
H 2.119925000000000 0.820752000000000 -0.695584000000000
H 1.100815000000000 -1.171448000000000 1.153599000000000
37
R5_SMe4_-1_1 charge=-1 multiplicity=1 wB97MV_TZVPD_SMD_chcl3=-1318.177952613682, B3LYP_D3BJ_TZVPD=-1317.966507098693, CCSDT_SMD_chcl3=-1316.264020562286, CCSDT=-1316.193695152822, B3LYP_TZVP_CPCM_chcl3_freq=0.2383297, B3LYP_TZVPD_CPCM_chcl3=-1318.033939210487, M062X_TZVPD=-1318.061688819728, COSMORS=-0.008573336694259123, M062X_TZVPD_CPCM_chcl3=-1318.131463378876, M062X_TZVPD_SMD_chcl3=-1318.133384443001, wB97MV_TZVPD_CPCM_chcl3=-1318.175821324228, B3LYP_D3BJ_TZVPD_SMD_chcl3=-1318.035723142439, wB97MV_TZVPD=-1318.107163762118
C 1.915589000000000 -1.007907000000000 0.295027000000000
C 0.881572000000000 -0.099225000000000 0.042543000000000
C -0.486858000000000 -0.271805000000000 -0.043623000000000
C -1.362643000000000 0.918910000000000 -0.332108000000000
C -1.153859000000000 -1.560267000000000 0.004432000000000
C -0.658552000000000 -2.787642000000000 -0.305438000000000
C -2.559230000000000 -1.764679000000000 0.456449000000000
O -3.323239000000000 -0.933009000000000 0.922540000000000
C -1.621651000000000 -3.920714000000000 -0.129250000000000
C -2.923008000000000 -3.229620000000000 0.280878000000000
C 4.895543000000000 1.105783000000000 0.321040000000000
O 3.534346000000000 0.703046000000000 0.211461000000000
O 4.217941000000000 -1.402664000000000 0.690735000000000
O -0.185457000000000 3.053125000000000 -0.483144000000000
C -0.382455000000000 3.492817000000000 2.121459000000000
O -0.974676000000000 2.344081000000000 1.492700000000000
C -0.771099000000000 2.219021000000000 0.168480000000000
C 3.277882000000000 -0.641949000000000 0.423187000000000
H 1.726772000000000 -2.057752000000000 0.457209000000000
H 1.224704000000000 0.922711000000000 -0.078984000000000
H -2.315242000000000 0.790720000000000 0.177849000000000
H 0.340224000000000 -2.962526000000000 -0.675660000000000
H -1.723150000000000 -4.500866000000000 -1.051524000000000
H -1.275083000000000 -4.624059000000000 0.635809000000000
H -3.690206000000000 -3.299378000000000 -0.494348000000000
H -3.360778000000000 -3.612123000000000 1.203778000000000
H 4.904585000000000 2.176803000000000 0.125392000000000
H 5.523024000000000 0.591303000000000 -0.409204000000000
H 5.290117000000000 0.908605000000000 1.319530000000000
H -0.784597000000000 4.411457000000000 1.695436000000000
H 0.699604000000000 3.476304000000000 1.993682000000000
H -0.641941000000000 3.415527000000000 3.173635000000000
S -1.900180000000000 1.045669000000000 -2.109764000000000
C -0.324260000000000 0.882388000000000 -2.988104000000000
H 0.295723000000000 1.764659000000000 -2.844903000000000
H 0.205499000000000 -0.001857000000000 -2.636726000000000
H -0.564207000000000 0.767545000000000 -4.044674000000000
41
R6_SMe6_cyc4RP1_0_1 charge=0 multiplicity=1 B3LYP_D3BJ_TZVPD=-1357.818344838914, B3LYP_D3BJ_TZVPD_SMD_chcl3=-1357.843212346819, B3LYP_TZVP_CPCM_chcl3_freq=0.28218039, B3LYP_TZVPD_CPCM_chcl3=-1357.836263642212, M062X_TZVPD_SMD_chcl3=-1357.950376053743, M062X_TZVPD=-1357.924735928918, COSMORS=-0.0037723892591832744, wB97MV_TZVPD_CPCM_chcl3=-1357.98509720685, wB97MV_TZVPD_SMD_chcl3=-1357.992241674799, wB97MV_TZVPD=-1357.966803526995, M062X_TZVPD_CPCM_chcl3=-1357.943431214261
O -4.694399000000000 -0.827954000000000 0.486430000000000
C -3.434337000000000 -1.279813000000000 0.394208000000000
C -0.285961000000000 2.568198000000000 -0.408100000000000
H -5.737408000000000 -2.586843000000000 0.073882000000000
C -1.056202000000000 -0.563179000000000 0.043576000000000
C -2.494190000000000 -0.162684000000000 -0.000016000000000
H -5.490252000000000 -2.237879000000000 1.801698000000000
O -3.125922000000000 -2.432178000000000 0.589357000000000
C -5.701813000000000 -1.800732000000000 0.826859000000000
H -6.638870000000000 -1.252194000000000 0.850281000000000
H -1.515717000000000 4.478979000000000 -1.806165000000000
H -0.832253000000000 1.912460000000000 1.572984000000000
C -0.035575000000000 0.216673000000000 0.410638000000000
H -3.022889000000000 3.515854000000000 -1.837439000000000
C -0.076880000000000 1.686092000000000 0.821541000000000
C 1.367899000000000 -0.137711000000000 0.534375000000000
C 2.026514000000000 -1.433279000000000 0.218601000000000
H -1.566505000000000 2.939848000000000 -2.702548000000000
S 2.183062000000000 -1.577616000000000 -1.618041000000000
C 2.018473000000000 0.895218000000000 1.092087000000000
H 5.156741000000000 -0.367253000000000 1.324823000000000
H 1.380813000000000 -2.262592000000000 0.517001000000000
C 3.448412000000000 0.949924000000000 1.469111000000000
O 1.222732000000000 1.971638000000000 1.365502000000000
H 3.949301000000000 -2.372184000000000 0.551449000000000
C 4.187266000000000 -0.240683000000000 0.841134000000000
H 3.882391000000000 1.899579000000000 1.145712000000000
H 3.527763000000000 0.927201000000000 2.562351000000000
C 3.375126000000000 -1.532996000000000 0.945823000000000
H 4.378835000000000 -0.027490000000000 -0.213307000000000
H 3.171675000000000 -1.752418000000000 1.999669000000000
C 2.433985000000000 -3.367381000000000 -1.783807000000000
H 2.518509000000000 -3.577708000000000 -2.849292000000000
H 3.349248000000000 -3.686757000000000 -1.286825000000000
H 1.581873000000000 -3.912821000000000 -1.377855000000000
O -1.587396000000000 2.761072000000000 -0.626607000000000
O 0.599614000000000 2.995199000000000 -1.103907000000000
C -1.938076000000000 3.475993000000000 -1.830772000000000
H -0.834844000000000 -1.581608000000000 -0.250657000000000
H -2.794222000000000 0.146362000000000 -1.009488000000000
H -2.704864000000000 0.700384000000000 0.632728000000000

37
R5_SMe_complex_-1_1 charge=-1 multiplicity=1 wB97MV_TZVPD_SMD_chcl3=-1318.163493961059, B3LYP_D3BJ_TZVPD=-1317.958504290937, CCSDT_SMD_chcl3=-1316.252383373766, CCSDT=-1316.177931749193, B3LYP_TZVP_CPCM_chcl3_freq=0.23670052, B3LYP_TZVPD_CPCM_chcl3=-1318.030357286723, M062X_TZVPD=-1318.043283122281, COSMORS=-0.00867983707833625, M062X_TZVPD_CPCM_chcl3=-1318.119087744092, M062X_TZVPD_SMD_chcl3=-1318.118209440026, wB97MV_TZVPD_CPCM_chcl3=-1318.164082197552, B3LYP_D3BJ_TZVPD_SMD_chcl3=-1318.030134088707, wB97MV_TZVPD=-1318.088586312529
C 1.800270000000000 0.239431000000000 0.883459000000000
C 0.826899000000000 0.992956000000000 0.315723000000000
C -0.538854000000000 0.572801000000000 0.131096000000000
C -1.450918000000000 1.453975000000000 -0.363893000000000
C -0.912054000000000 -0.801644000000000 0.508008000000000
C -0.413747000000000 -1.938804000000000 0.002662000000000
C -1.871740000000000 -1.122794000000000 1.587456000000000
O -2.426967000000000 -0.337634000000000 2.331572000000000
C -1.005593000000000 -3.172472000000000 0.612441000000000
C -2.007602000000000 -2.638084000000000 1.642610000000000
C 4.798975000000000 2.346596000000000 0.642638000000000
O 3.466123000000000 1.851872000000000 0.468505000000000
O 3.916601000000000 0.224737000000000 1.958969000000000
O -3.384739000000000 0.059578000000000 -0.661254000000000
C -4.870871000000000 2.106585000000000 -1.421905000000000
O -3.493602000000000 2.274699000000000 -1.063143000000000
C -2.833388000000000 1.146182000000000 -0.684554000000000
C 3.158612000000000 0.727128000000000 1.148905000000000
H 1.584825000000000 -0.715684000000000 1.332603000000000
H 1.073983000000000 1.984307000000000 -0.042335000000000
H -1.133224000000000 2.466255000000000 -0.576975000000000
H 0.357136000000000 -1.984209000000000 -0.755884000000000
H -1.475332000000000 -3.793786000000000 -0.154916000000000
H -0.222068000000000 -3.785083000000000 1.067432000000000
H -3.040119000000000 -2.900058000000000 1.402584000000000
H -1.811920000000000 -2.983627000000000 2.658527000000000
H 4.986680000000000 2.597615000000000 1.686521000000000
H 4.865350000000000 3.236144000000000 0.021554000000000
H 5.522745000000000 1.600287000000000 0.313931000000000
H -5.229319000000000 3.098585000000000 -1.684412000000000
H -5.440577000000000 1.707453000000000 -0.582698000000000
H -4.967319000000000 1.432067000000000 -2.273006000000000
S 3.137378000000000 -1.281975000000000 -1.148157000000000
C 2.088762000000000 -0.449828000000000 -2.376892000000000
H 1.360405000000000 0.193301000000000 -1.864961000000000
H 2.673378000000000 0.181734000000000 -3.048711000000000
H 1.526859000000000 -1.166003000000000 -2.981249000000000
40
R6_SMe_complex_-1_1 charge=-1 multiplicity=1 wB97MV_TZVPD_SMD_chcl3=-1357.467992126556, B3LYP_D3BJ_TZVPD=-1357.261648743625, B3LYP_TZVP_CPCM_chcl3_freq=0.26479933, B3LYP_TZVPD_CPCM_chcl3=-1357.334444643165, M062X_TZVPD=-1357.349796030403, COSMORS=-0.010024103635038637, M062X_TZVPD_CPCM_chcl3=-1357.426501875507, M062X_TZVPD_SMD_chcl3=-1357.426525631766, wB97MV_TZVPD_CPCM_chcl3=-1357.467729491982, B3LYP_D3BJ_TZVPD_SMD_chcl3=-1357.335085547468, wB97MV_TZVPD=-1357.391223496569
C 1.684482000000000 1.316391000000000 -0.831834000000000
C 0.987182000000000 0.166865000000000 -1.007775000000000
C -0.296774000000000 -0.138523000000000 -0.451317000000000
C -0.891518000000000 -1.317906000000000 -0.794076000000000
C -0.941677000000000 0.832660000000000 0.471754000000000
C -0.584037000000000 0.918434000000000 1.760901000000000
C -2.004499000000000 1.712098000000000 -0.067290000000000
O -2.257231000000000 1.766301000000000 -1.261204000000000
H -4.068609000000000 -3.542183000000000 0.146669000000000
C -2.785305000000000 2.523484000000000 0.941082000000000
C 4.732810000000000 0.805914000000000 -2.812408000000000
O 3.455807000000000 0.584728000000000 -2.201838000000000
O 3.601803000000000 2.630769000000000 -1.268700000000000
O -3.034412000000000 -1.084302000000000 0.274548000000000
C -3.983911000000000 -3.251511000000000 -0.900753000000000
O -2.666058000000000 -2.763656000000000 -1.176655000000000
C -2.274604000000000 -1.681784000000000 -0.463634000000000
C 2.976825000000000 1.591933000000000 -1.430200000000000
H 1.301060000000000 2.112669000000000 -0.208933000000000
H 1.416039000000000 -0.598403000000000 -1.645052000000000
H -0.399923000000000 -1.968994000000000 -1.499882000000000
C -1.176579000000000 1.870173000000000 2.748734000000000
H -4.733415000000000 -2.492754000000000 -1.126239000000000
H -4.117098000000000 -4.117383000000000 -1.544150000000000
C -1.897303000000000 3.032959000000000 2.074058000000000
H -3.556745000000000 1.856982000000000 1.345901000000000
H 4.697894000000000 1.667920000000000 -3.478885000000000
H 4.947417000000000 -0.098009000000000 -3.376685000000000
H 5.499482000000000 0.969250000000000 -2.054921000000000
H 0.172356000000000 0.237585000000000 2.134570000000000
H -3.290620000000000 3.333749000000000 0.415117000000000
H -1.877047000000000 1.307921000000000 3.381728000000000
H -0.393723000000000 2.231546000000000 3.420856000000000
H -1.155418000000000 3.725408000000000 1.664741000000000
H -2.491840000000000 3.590517000000000 2.800220000000000
S -0.271880000000000 -3.319323000000000 1.088340000000000
C 1.208407000000000 -2.385732000000000 1.573921000000000
H 2.123634000000000 -2.943865000000000 1.365469000000000
H 1.258860000000000 -1.446864000000000 1.008683000000000
H 1.195942000000000 -2.128298000000000 2.635624000000000

6. Copies of NMR spectra

^{1}H NMR, 600 MHz
$CDCl_3$

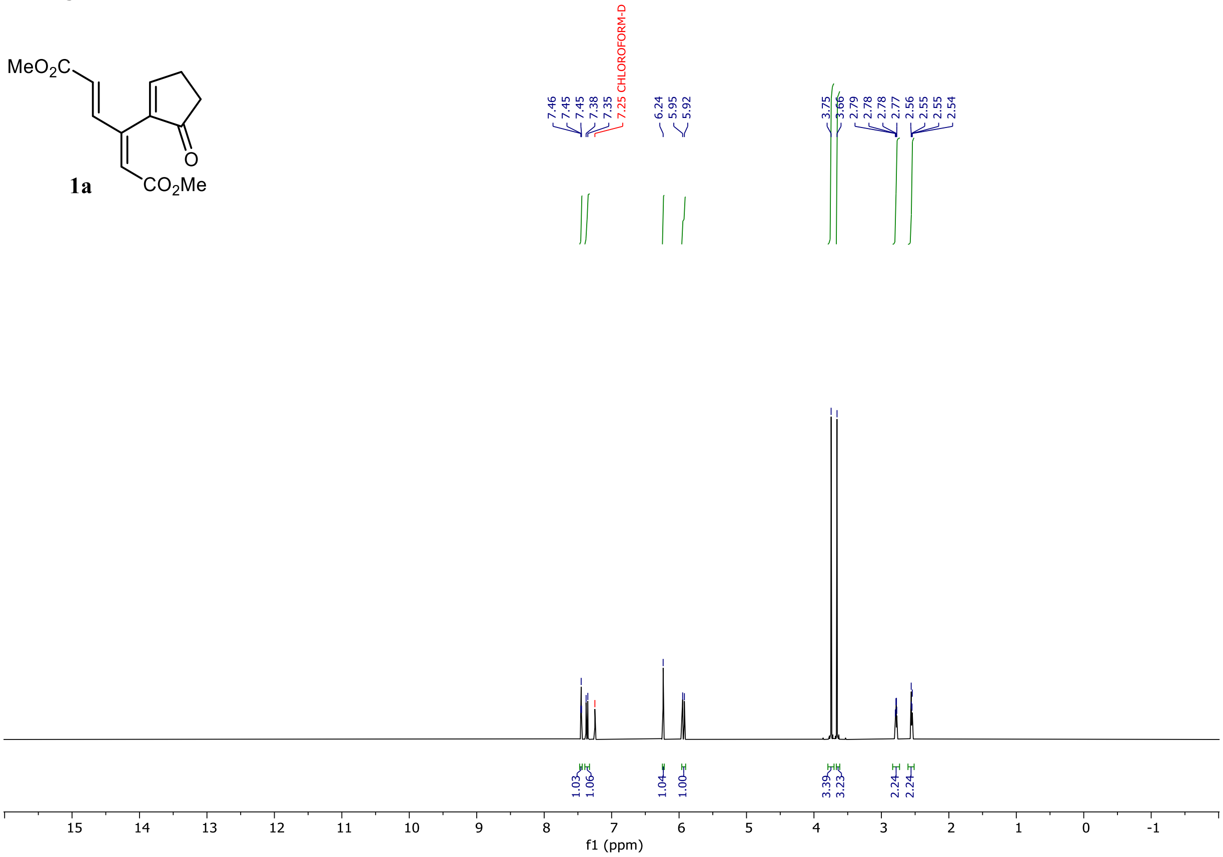

^{13}C{^{1}H} NMR, 151 MHz
$CDCl_3$

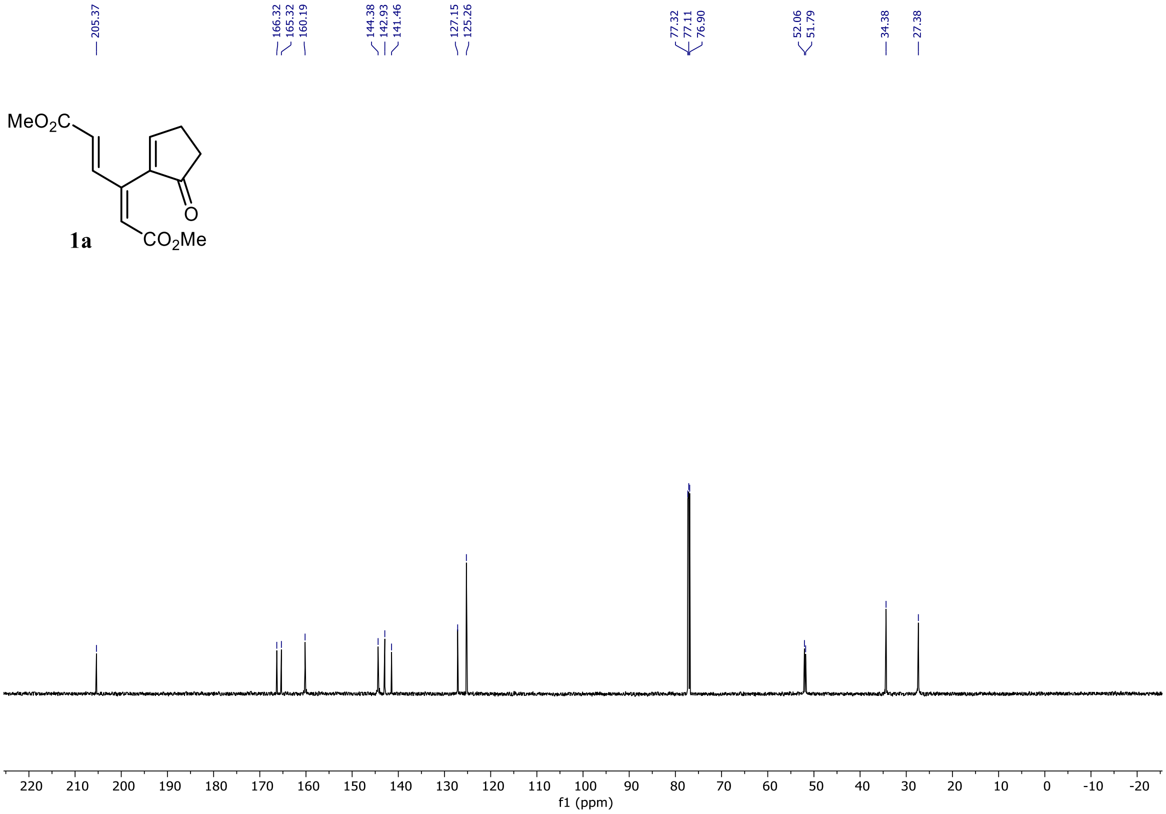

^{1}H NMR, 500 MHz
$CDCl_3$

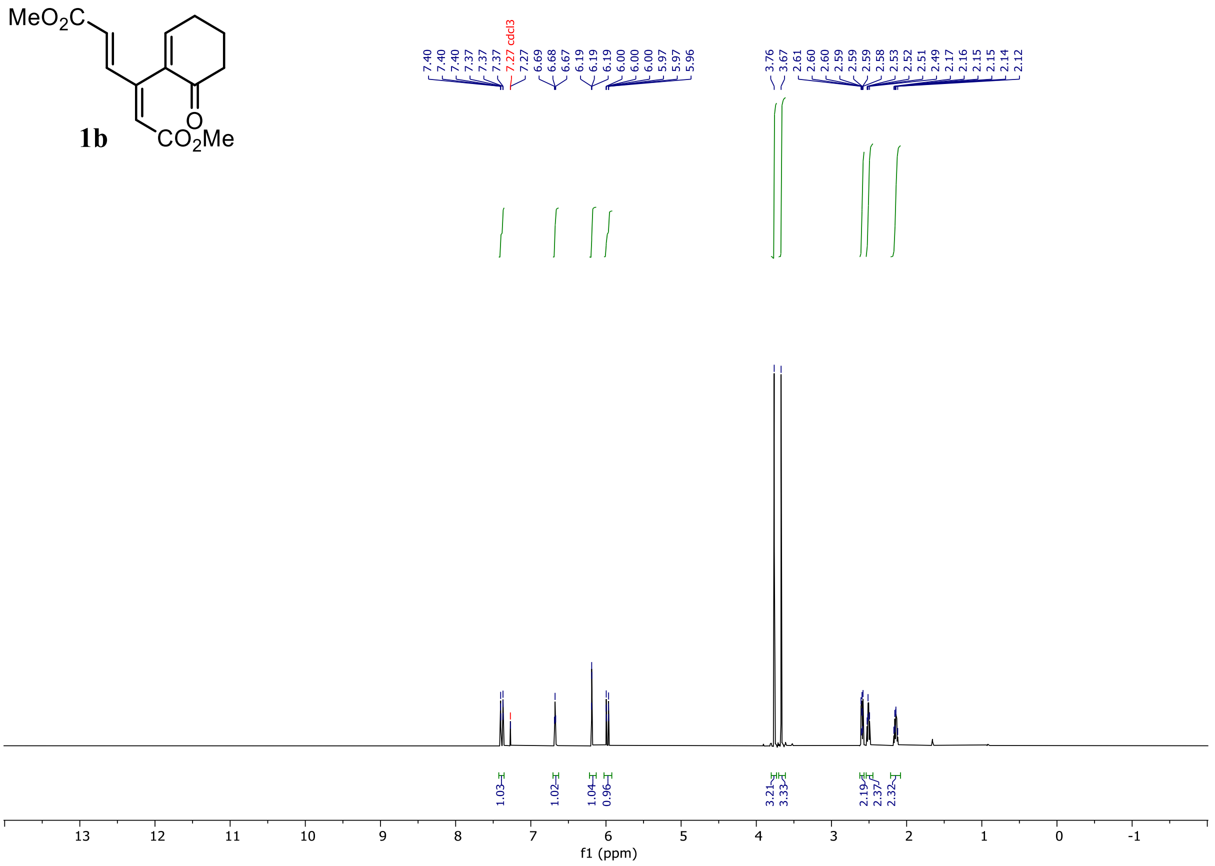

^{13}C{^{1}H} NMR, 126 MHz
$CDCl_3$

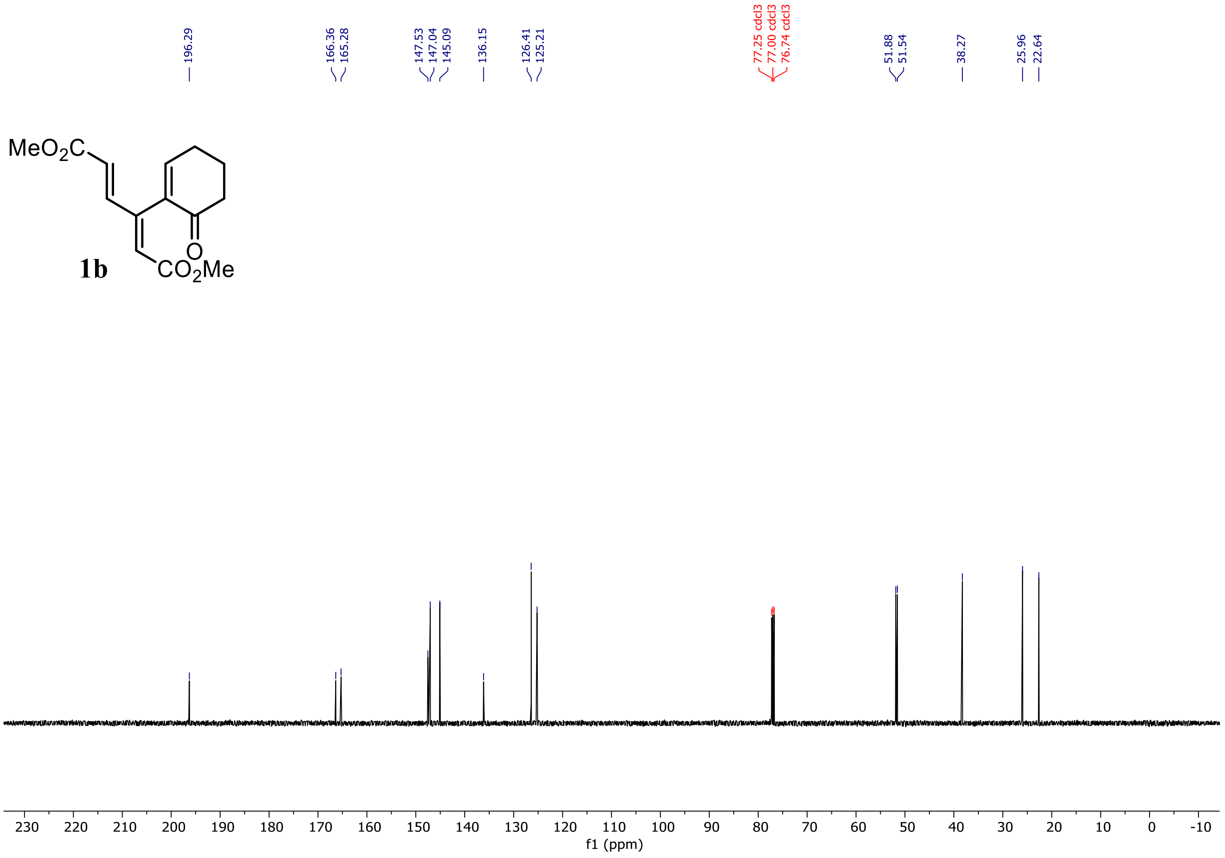

^{1}H NMR, 500 MHz
$CDCl_3$

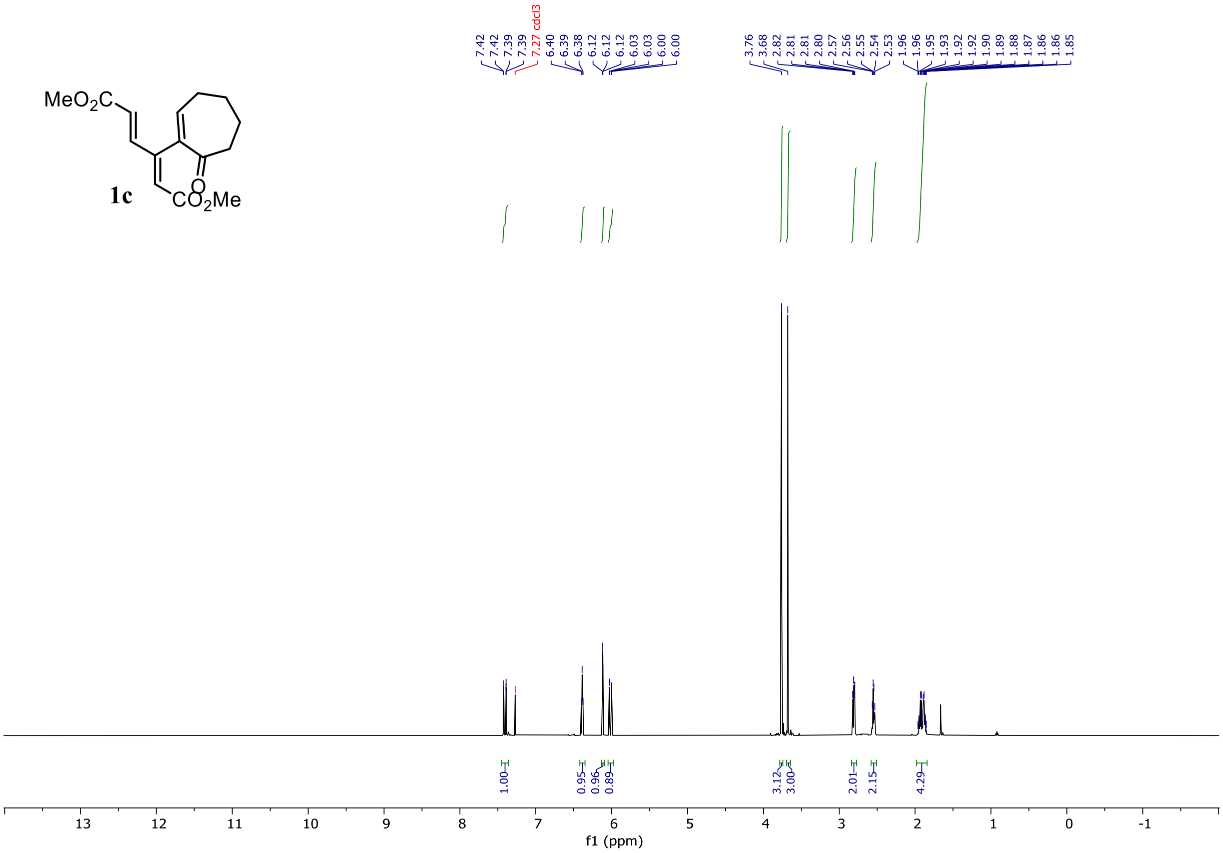

$^{13}C\{^{1}H\}$ NMR, 126 MHz
$CDCl_3$

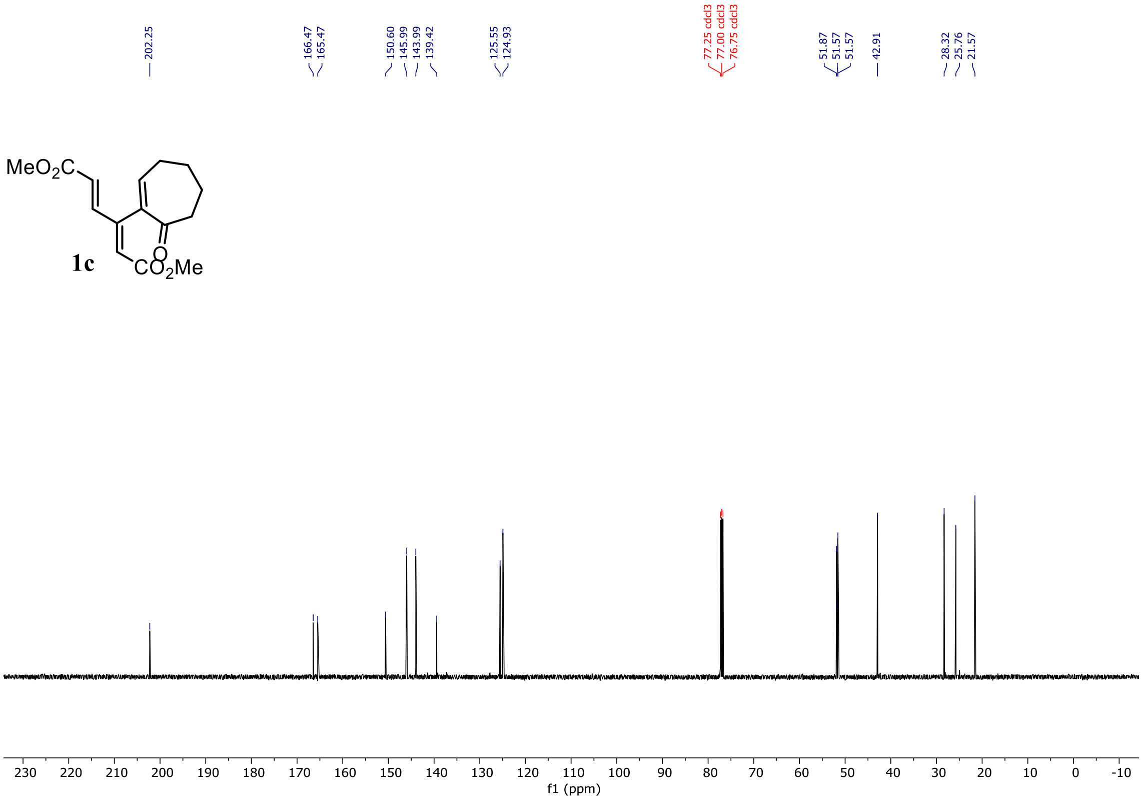

^{1}H NMR, 500 MHz
$CDCl_3$

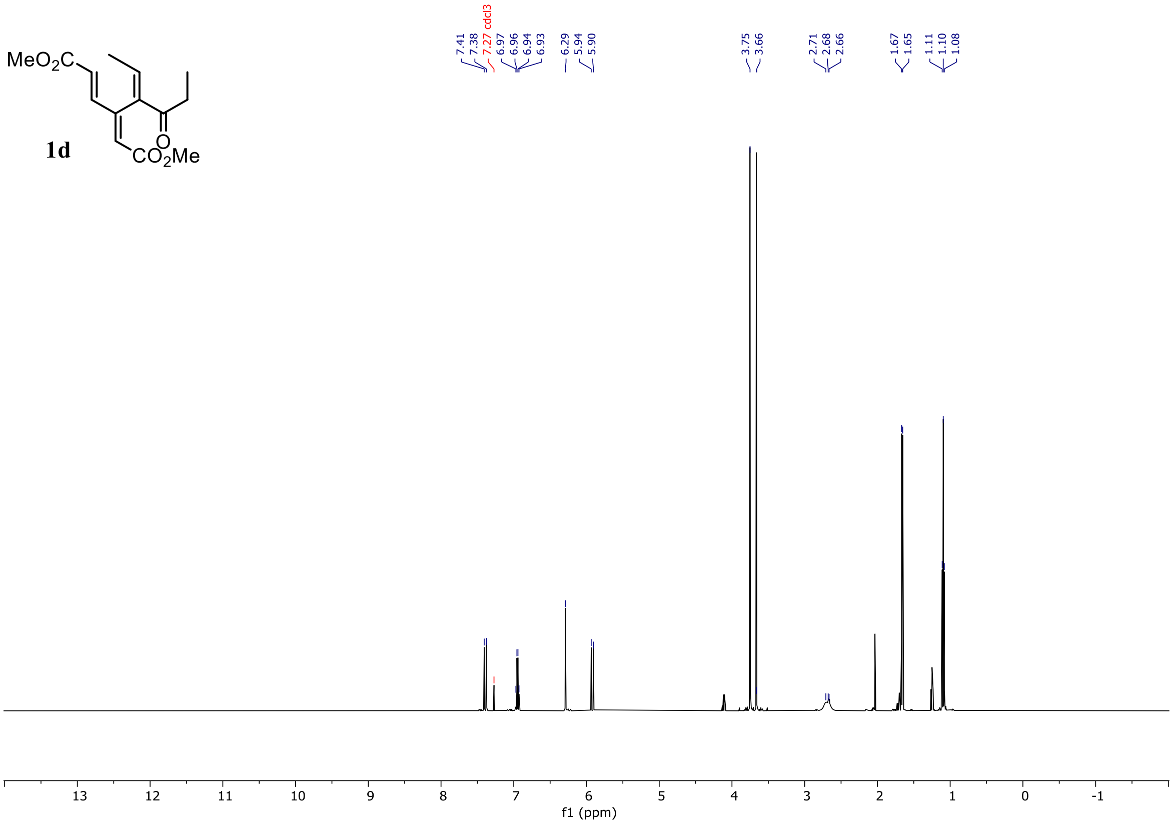

$^{13}C\{^{1}H\}$ NMR, 126 MHz
$CDCl_3$

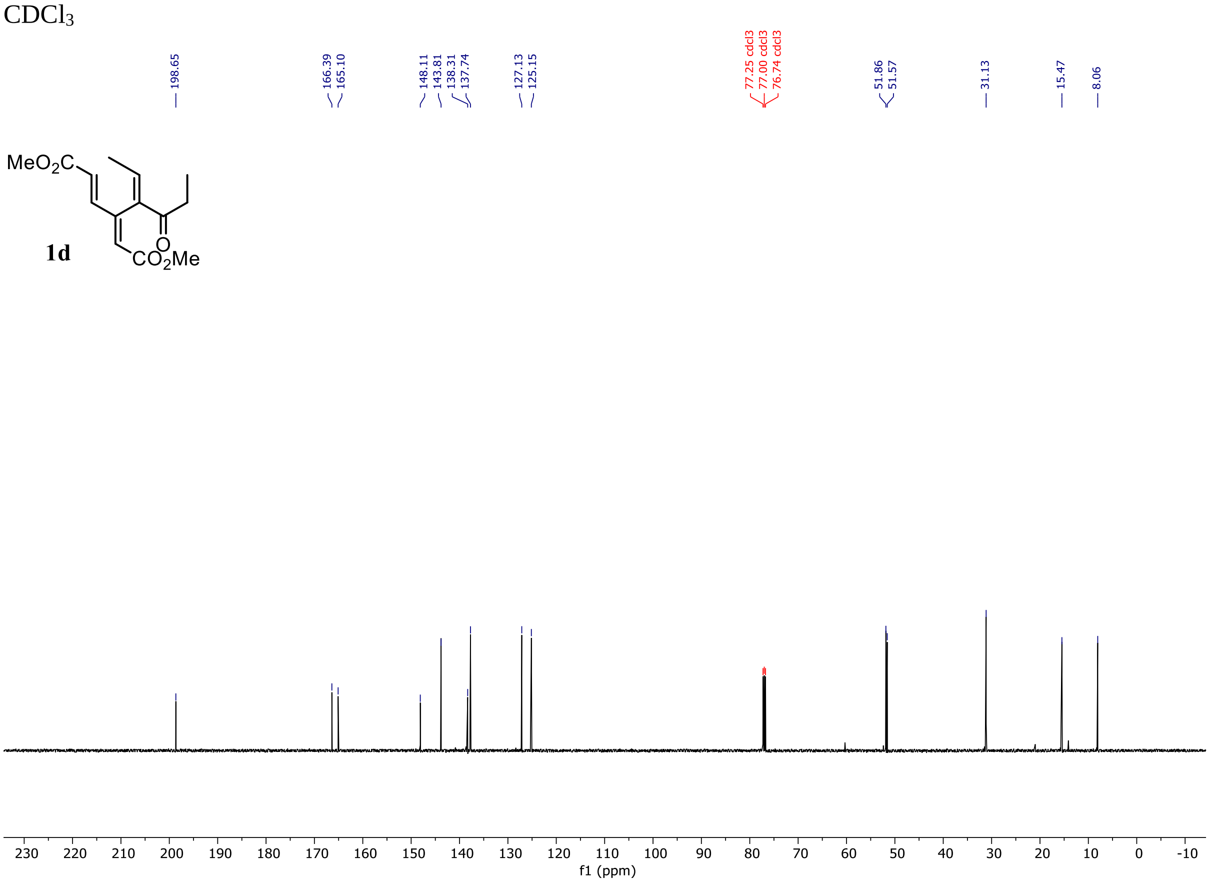

^{1}H NMR, 500 MHz
$CDCl_3$

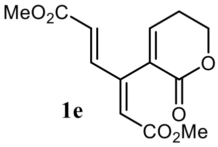

1e

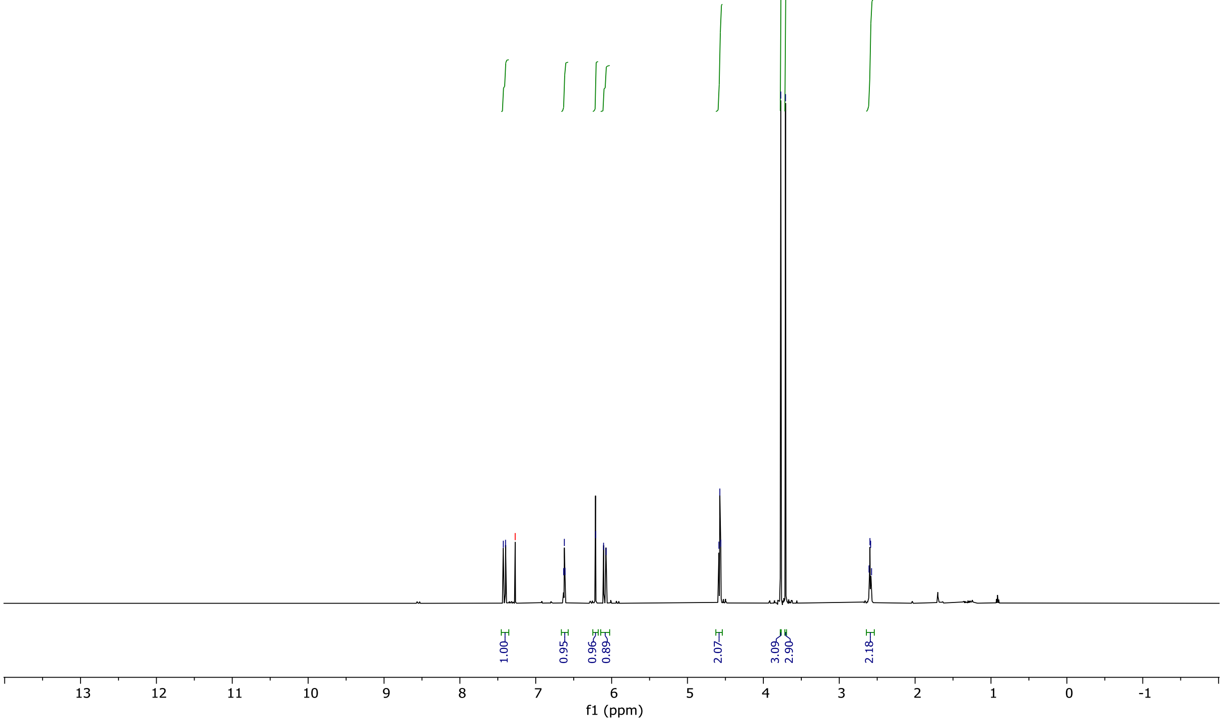

^{13}C{^{1}H} NMR, 126 MHz
$CDCl_3$

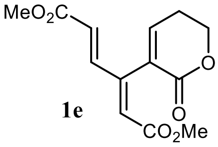

1e

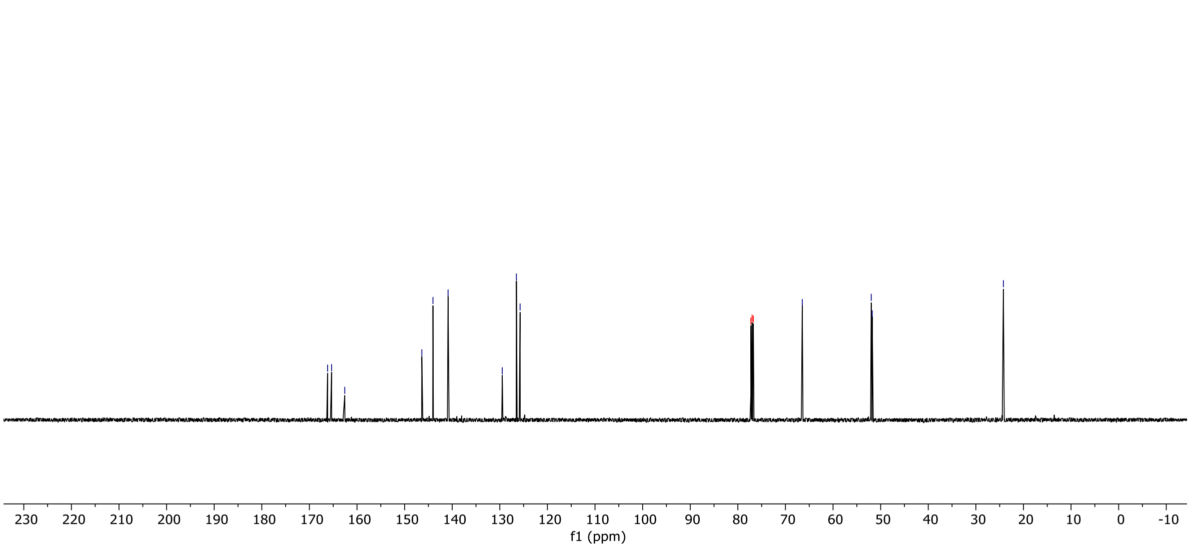

^{1}H NMR, 500 MHz
$CDCl_3$

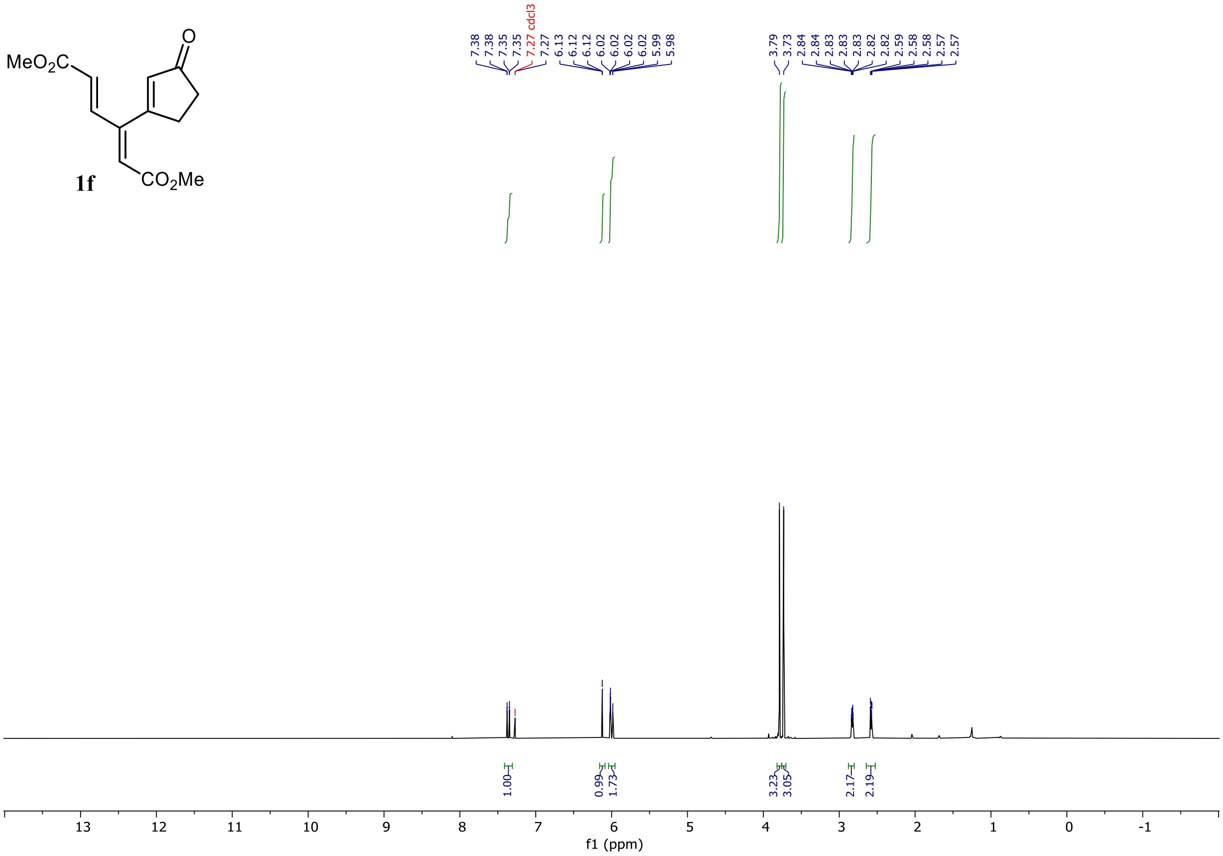

^{13}C{^{1}H} NMR, 126 MHz
$CDCl_3$

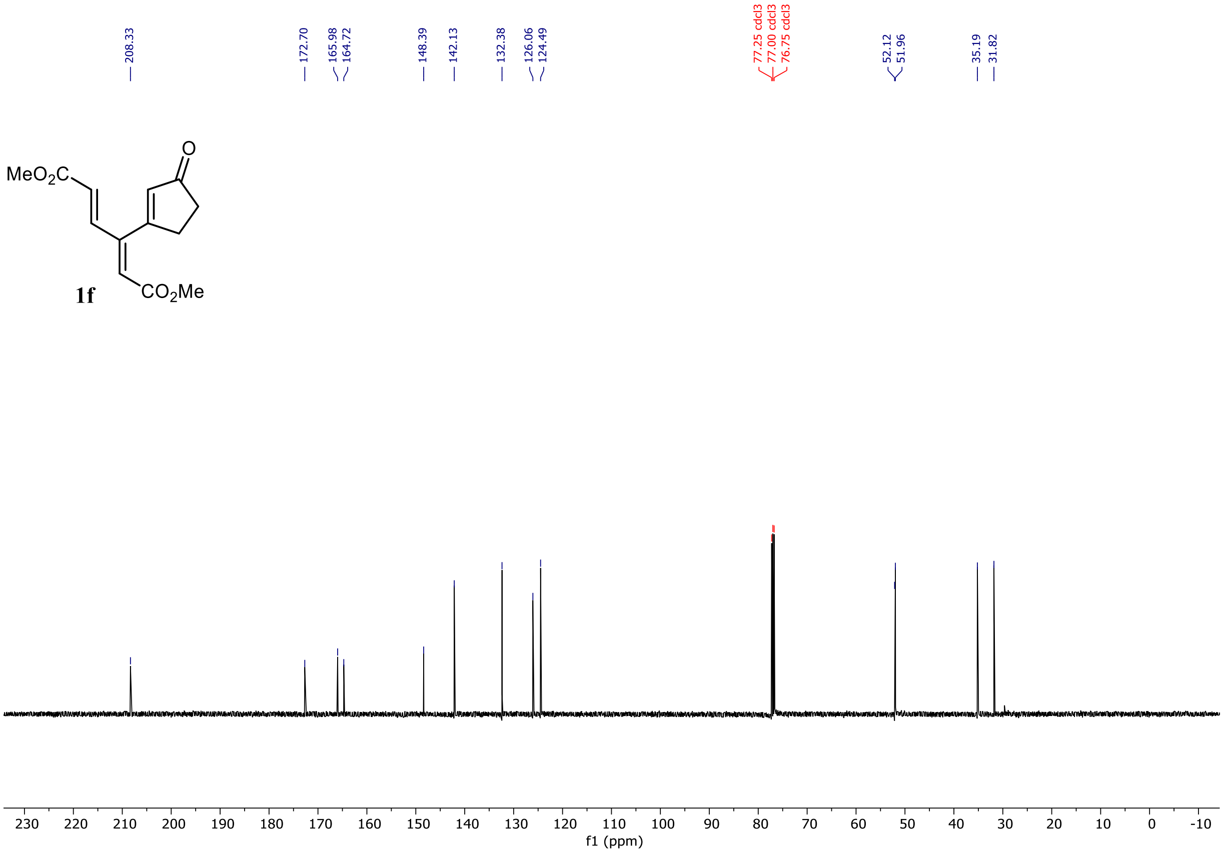

^{1}H NMR, 600 MHz
$CDCl_3$

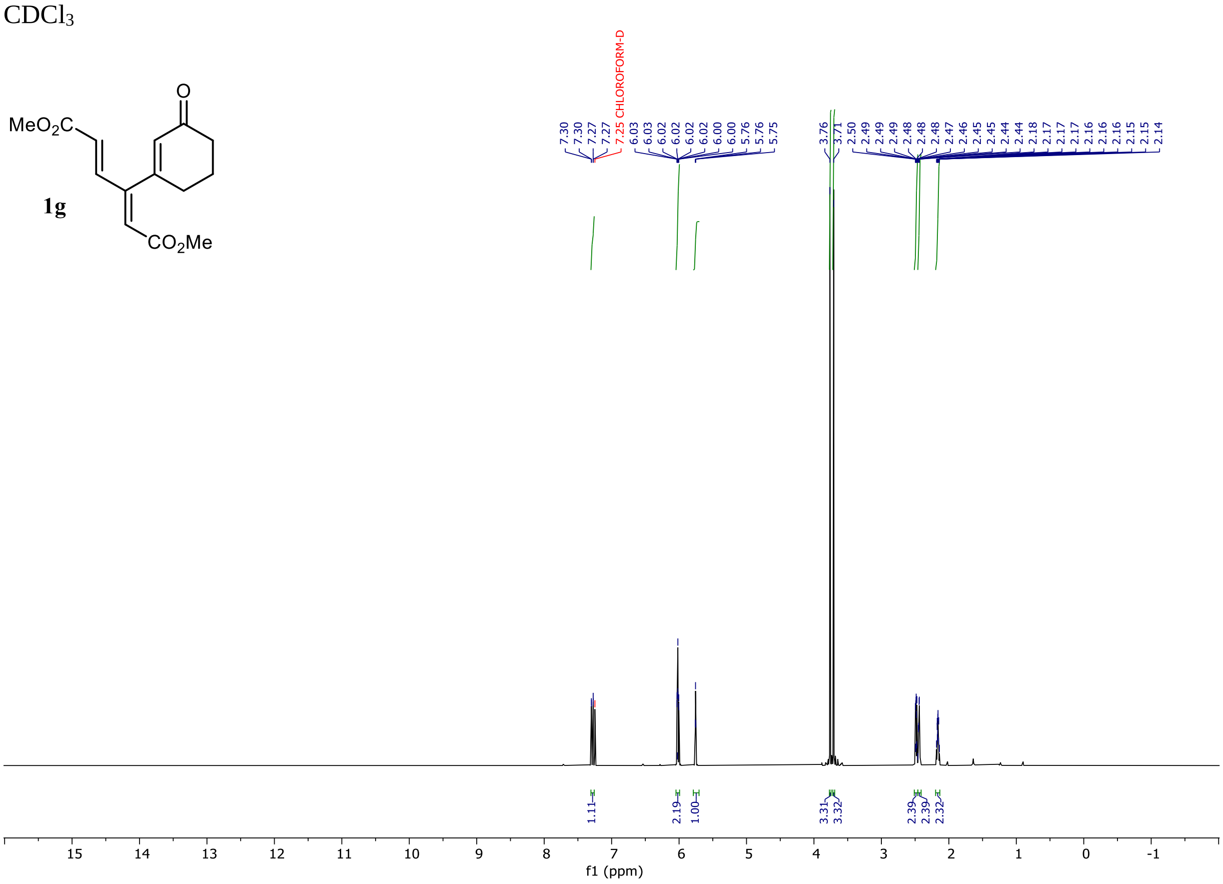

^{13}C{^{1}H} NMR, 151 MHz
$CDCl_3$

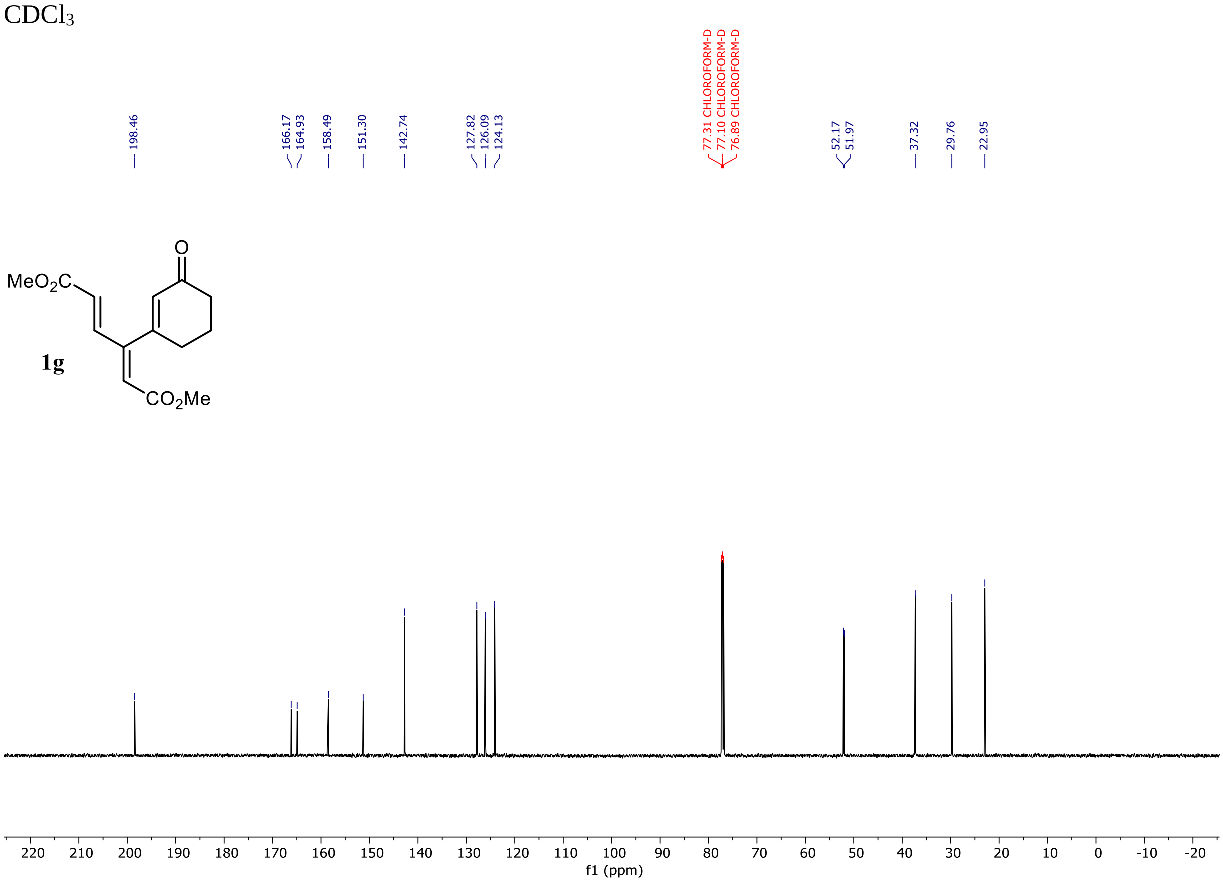

^{1}H NMR, 500 MHz
$CDCl_3$

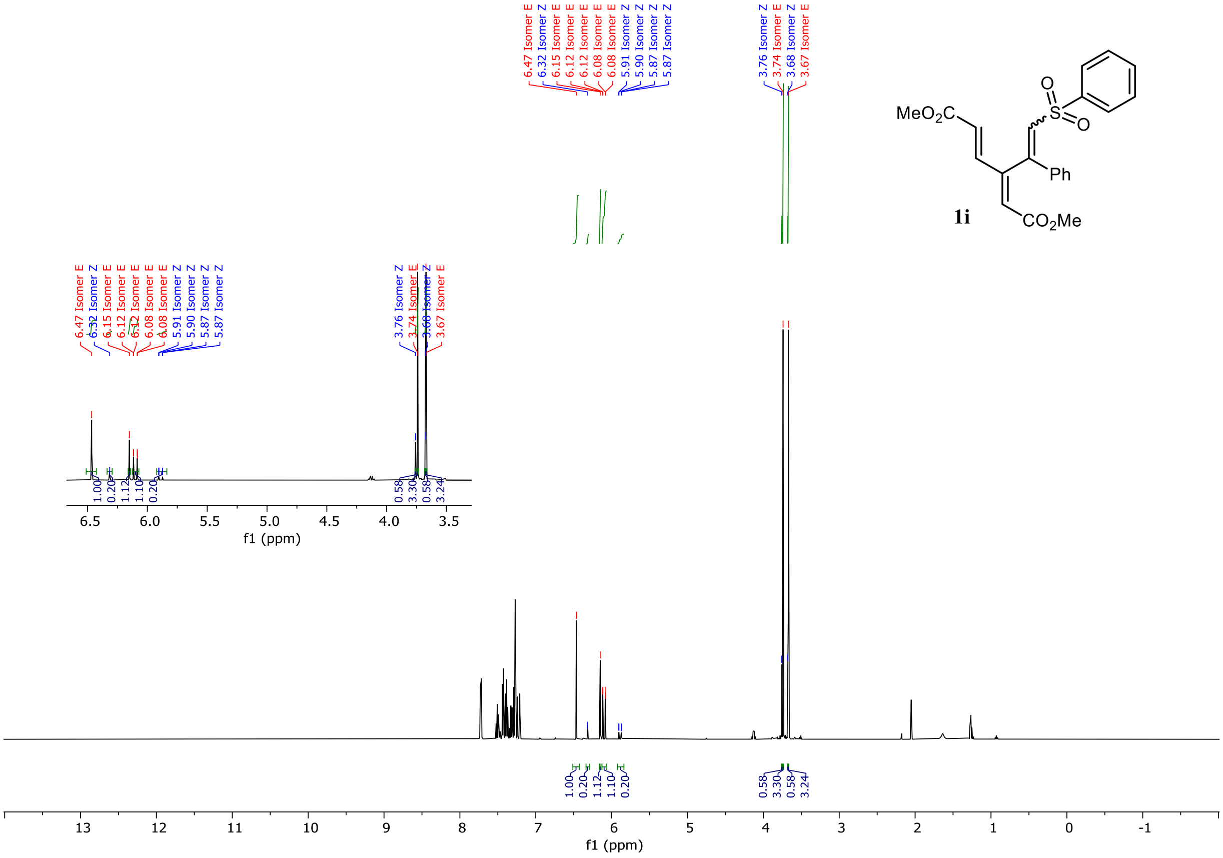

1i

^{1}H NMR, 500 MHz
$CDCl_3$

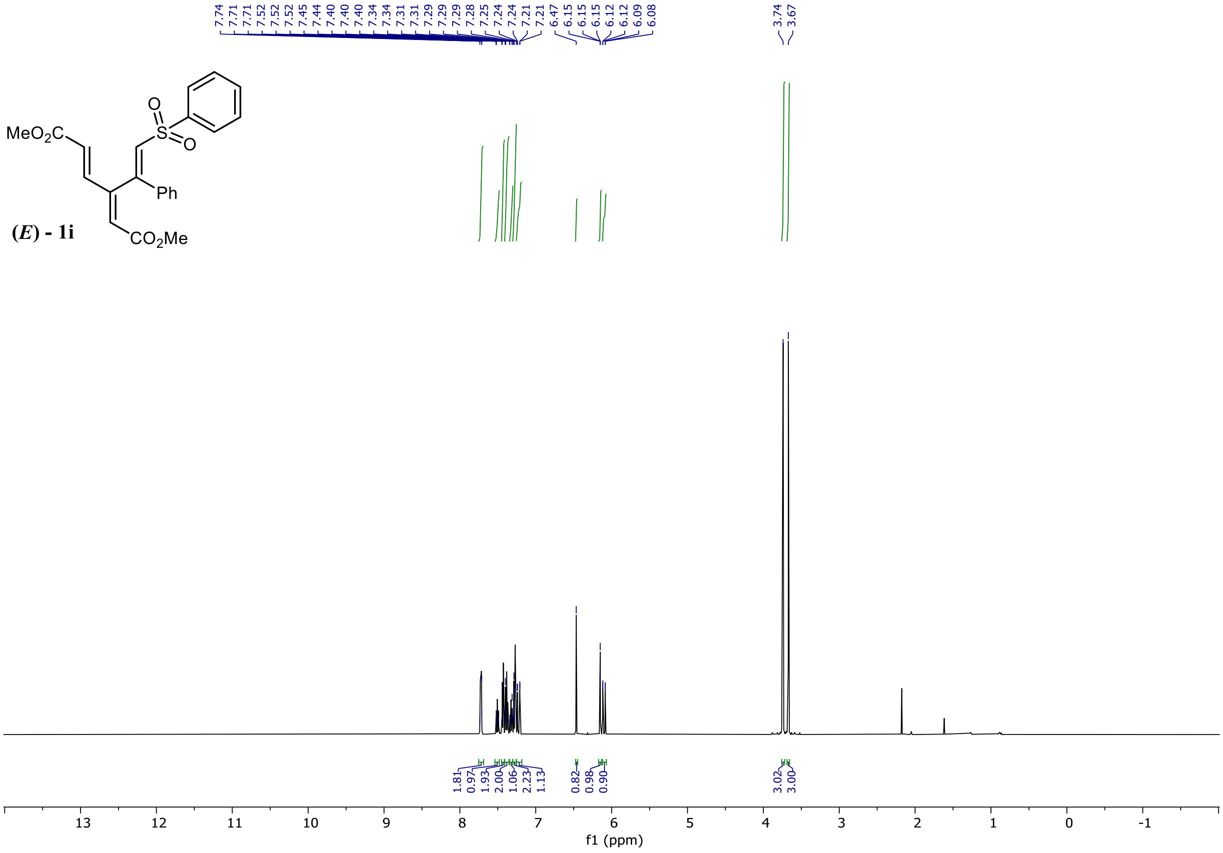

(*E*) - 1i

$^{13}C\{^{1}H\}$ NMR, 126 MHz
$CDCl_3$

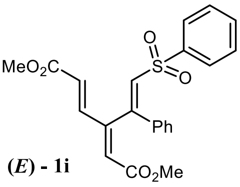

(*E*) - 1i

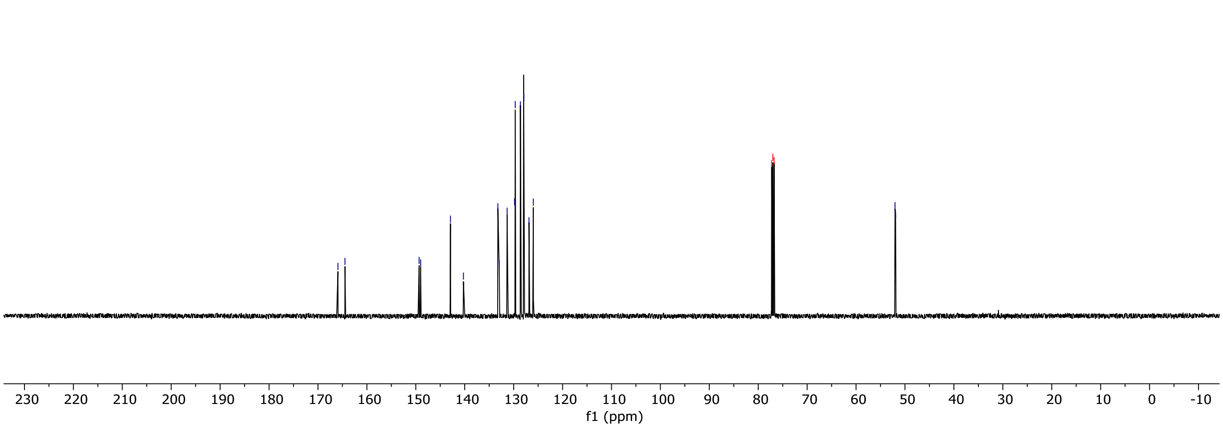

^{1}H NMR, 500 MHz
$CDCl_3$

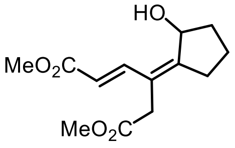

5

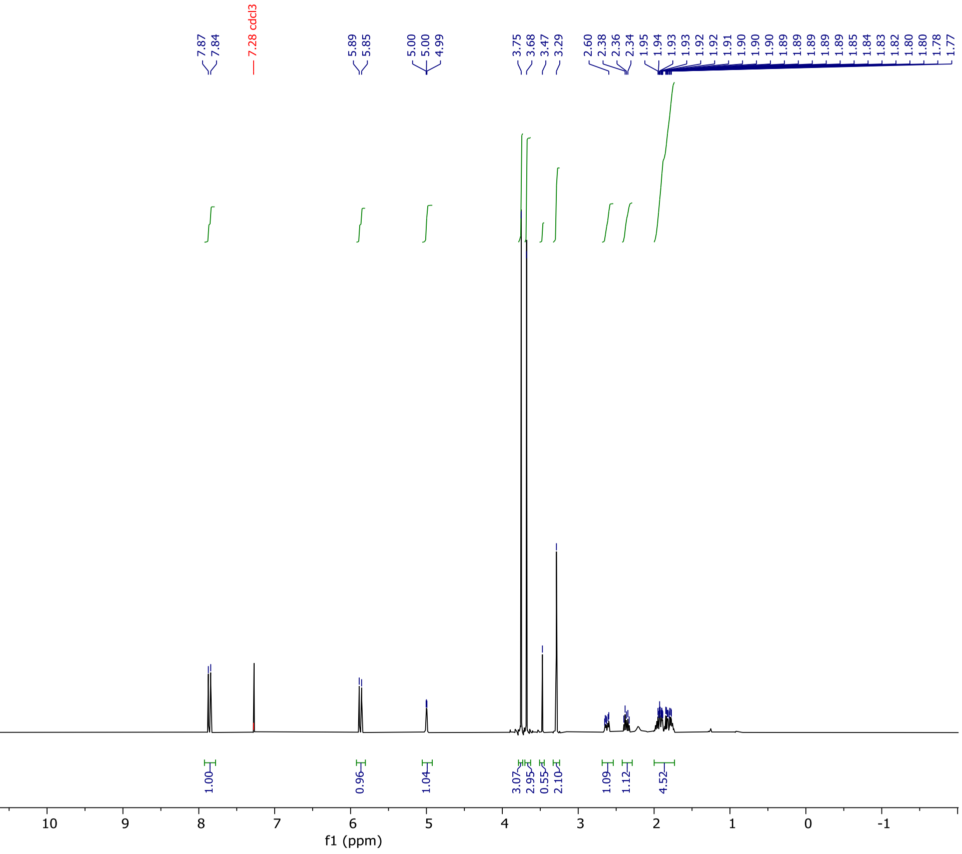

$^{13}C\{^{1}H\}$ NMR, 126 MHz
$CDCl_3$

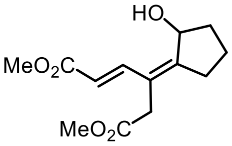

5

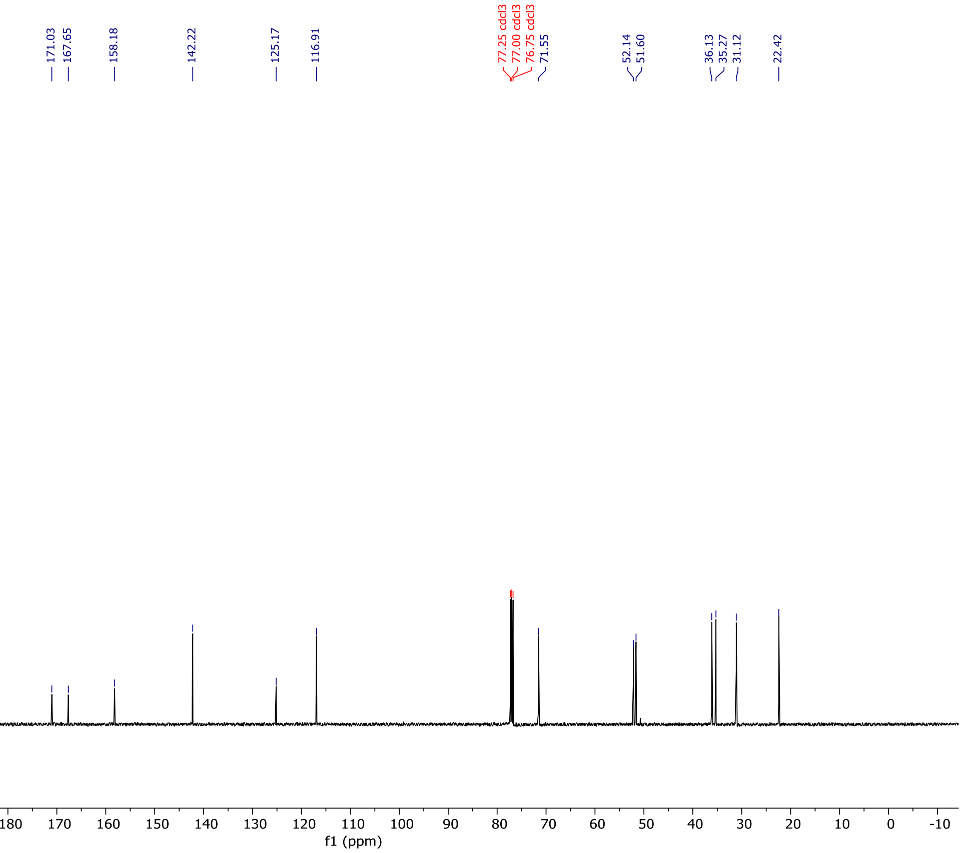

NOESY

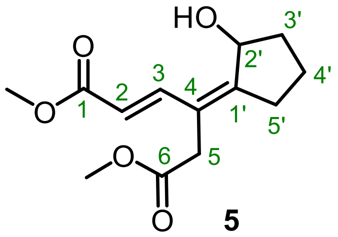

5

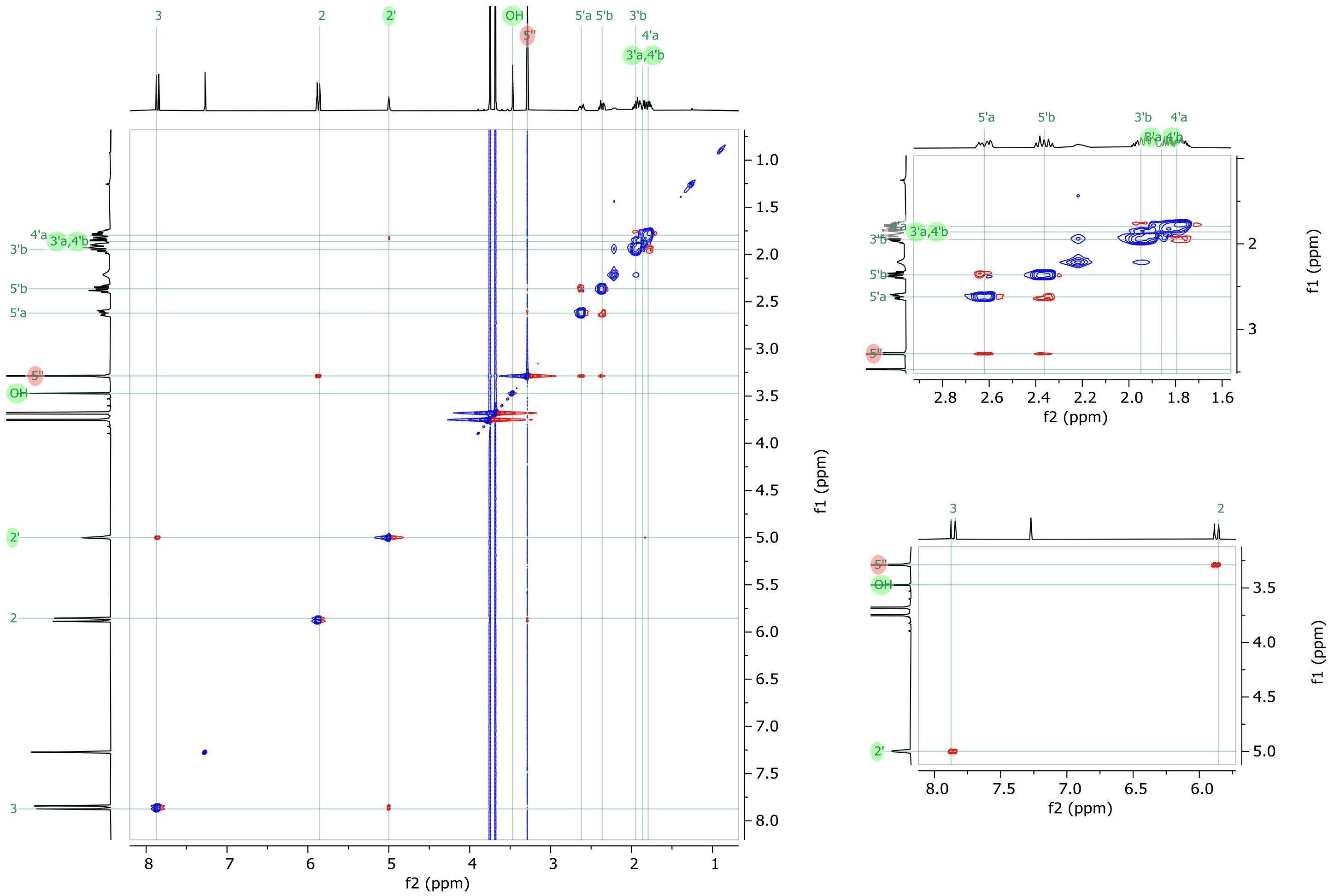

^{1}H NMR, 500 MHz
$CDCl_3$

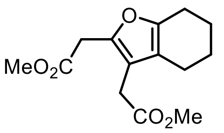

7b

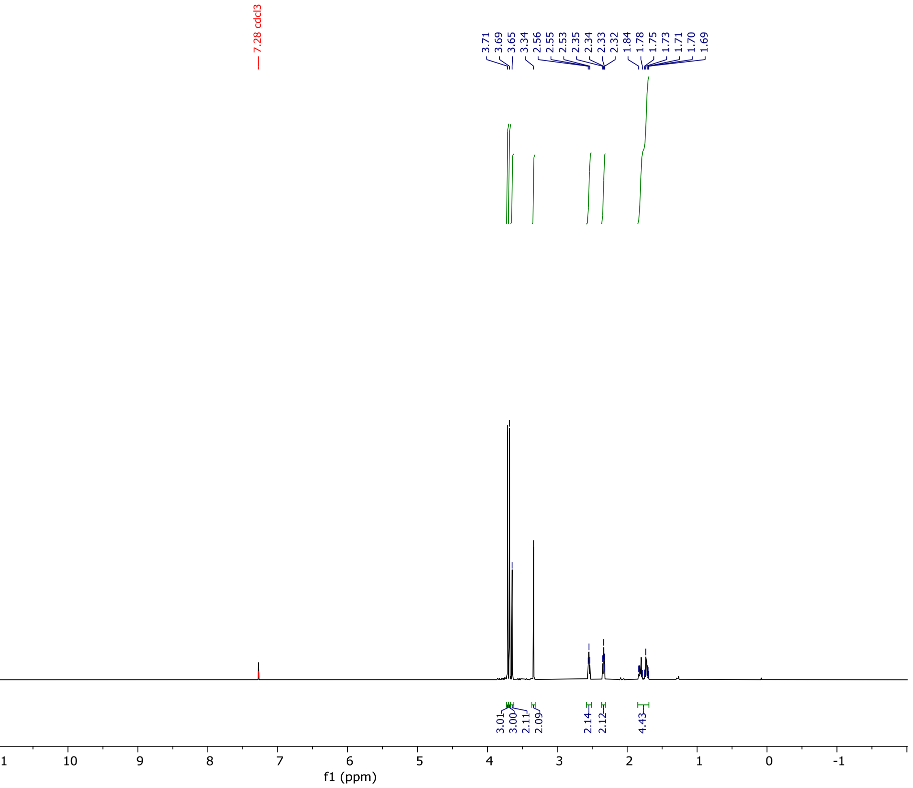

^{13}C{^{1}H} NMR, 126 MHz
$CDCl_3$

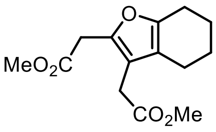

7b

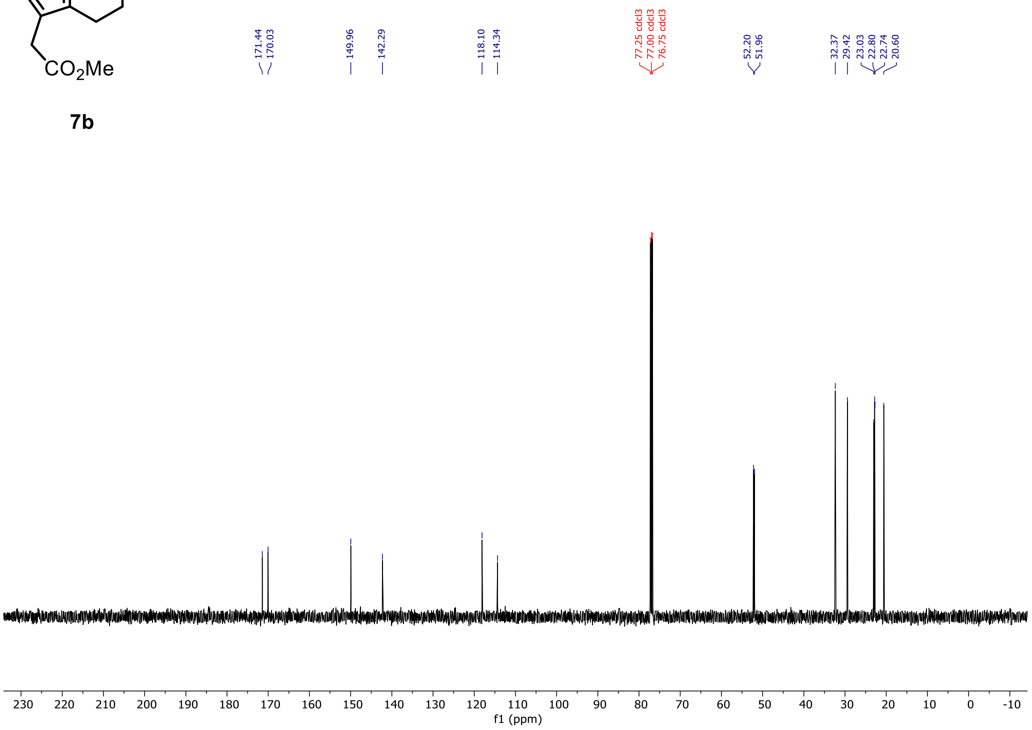

^{1}H-$^{13}C\{^{1}H\}$ HSQC

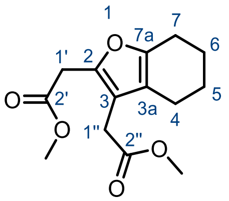

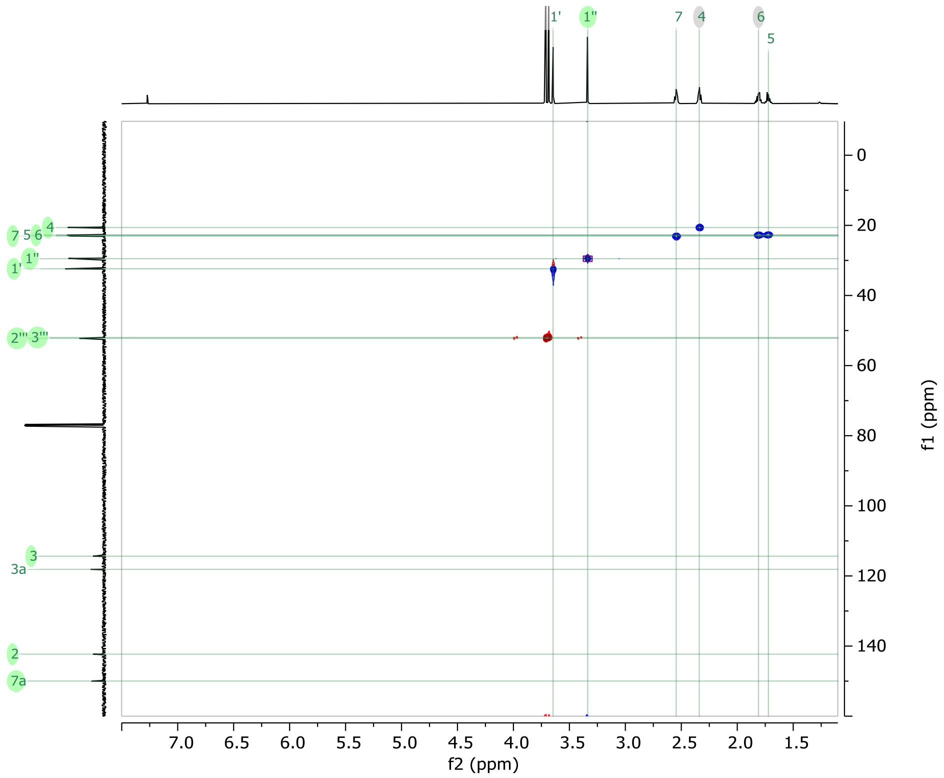

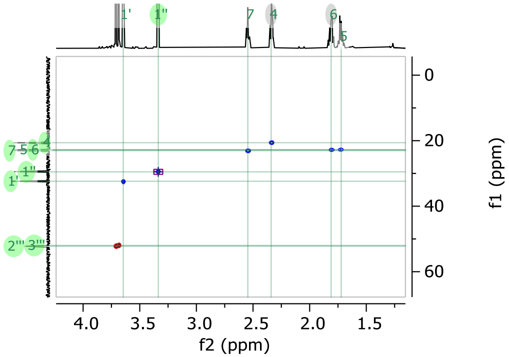

^{1}H NMR, 600 MHz
$CDCl_3$

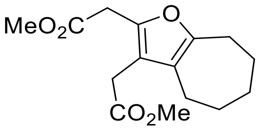

7c

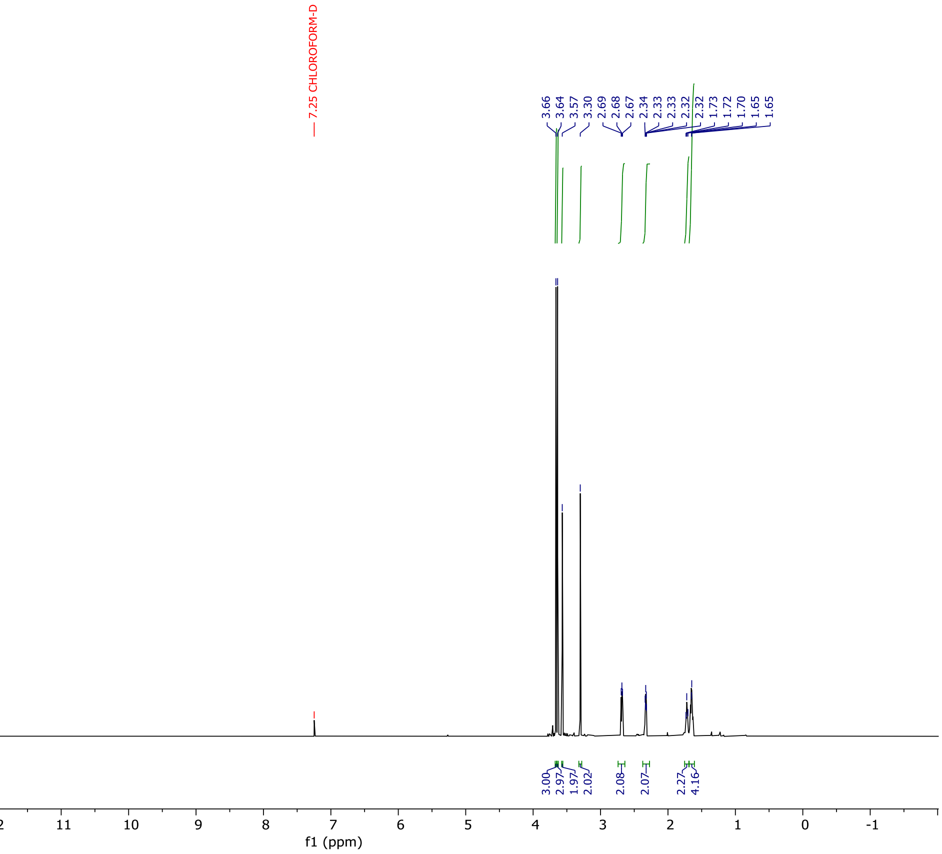

^{13}C{^{1}H} NMR, 151 MHz
$CDCl_3$

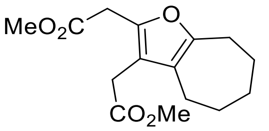

7c

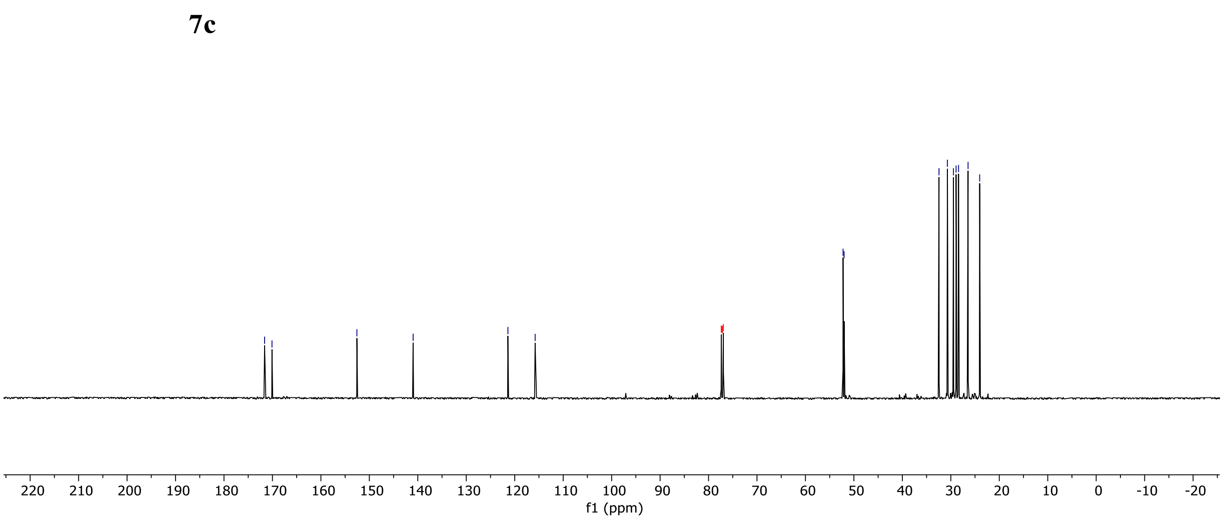

^{1}H NMR, 500 MHz
$CDCl_3$

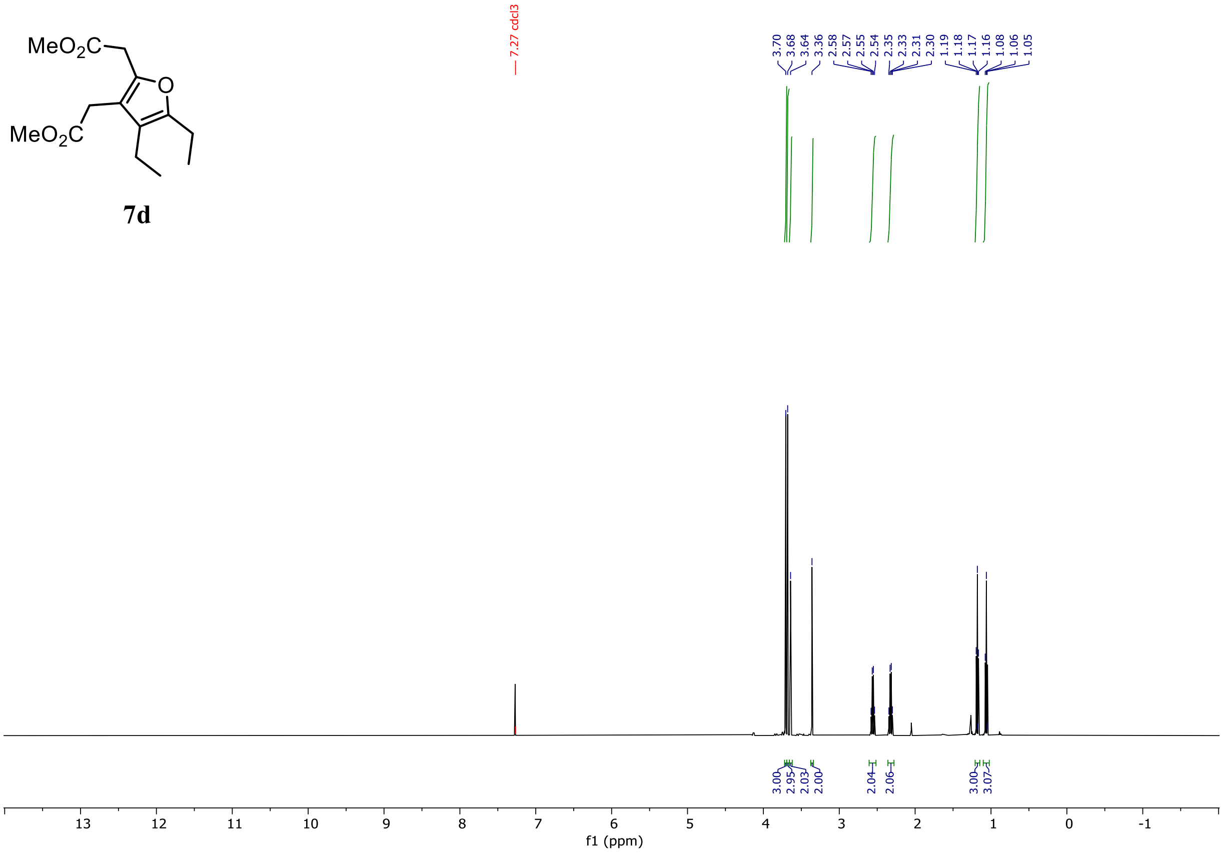

7d

^{13}C{^{1}H} NMR, 126 MHz
$CDCl_3$

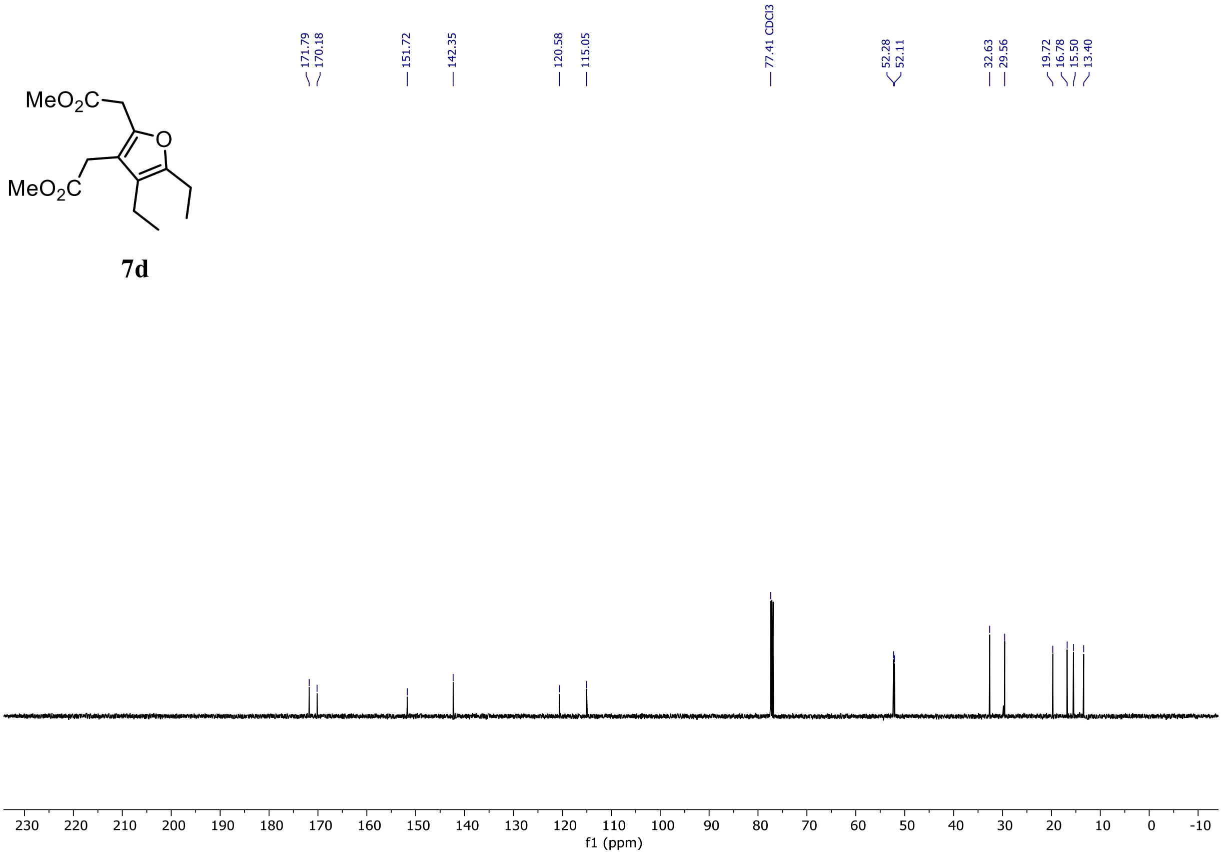

7d

^{1}H NMR, 500 MHz
$CDCl_3$

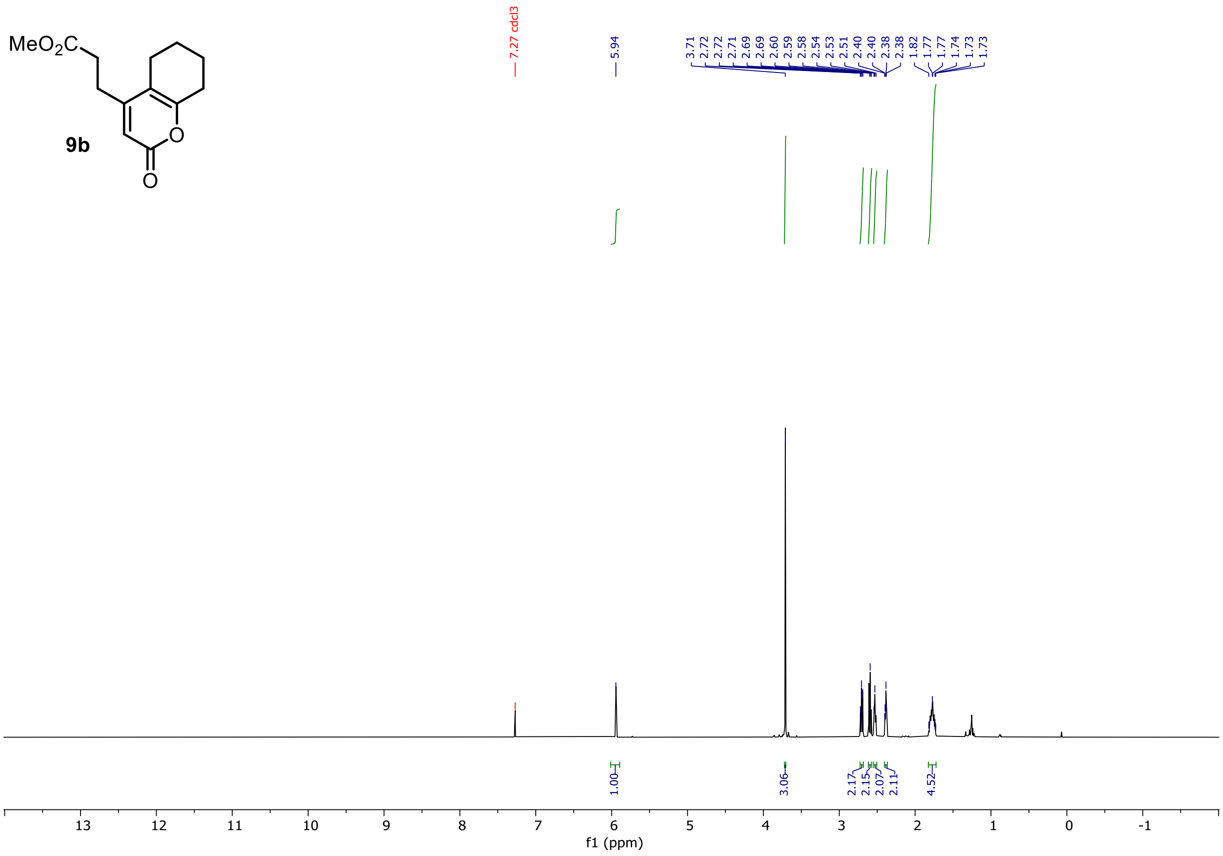

$^{13}C\{^{1}H\}$ NMR, 126 MHz
$CDCl_3$

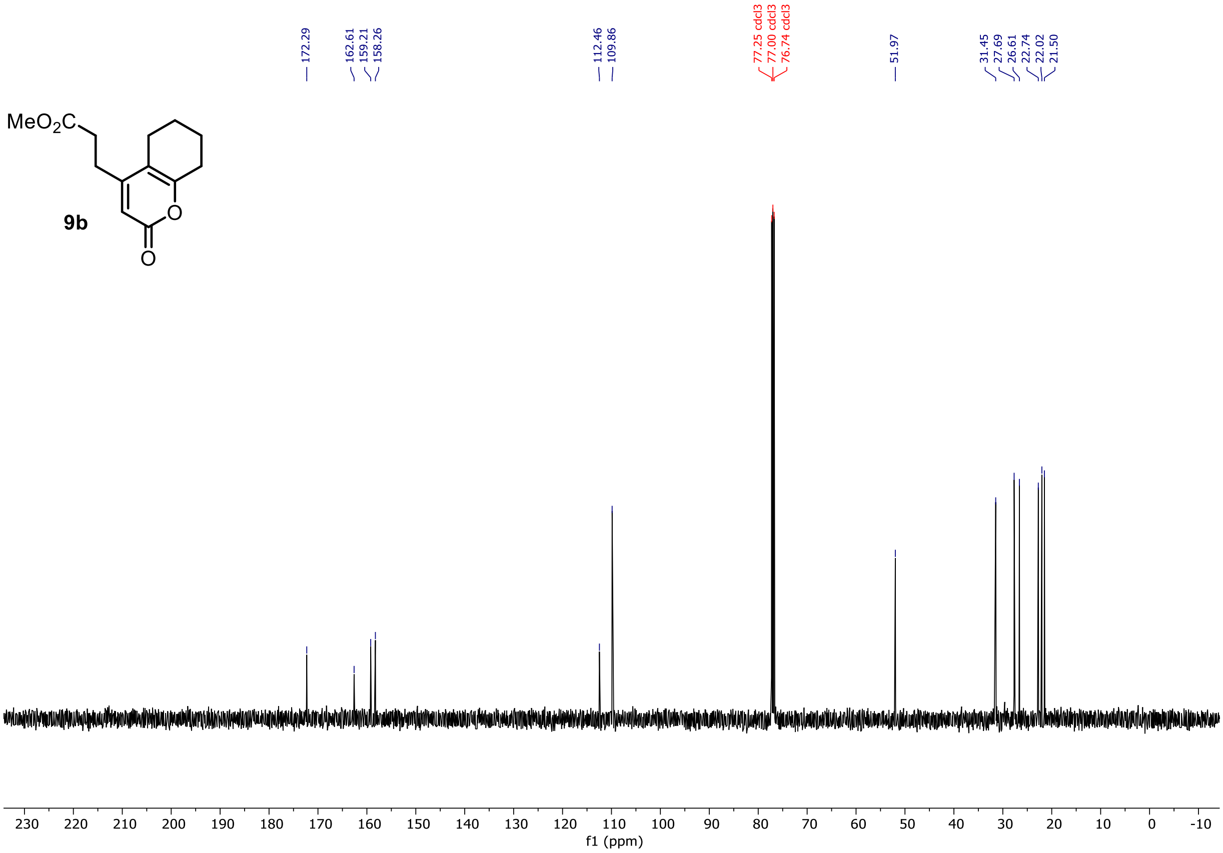

^1H-$^{13}C\{^1H\}$ HSQC

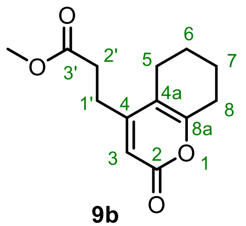

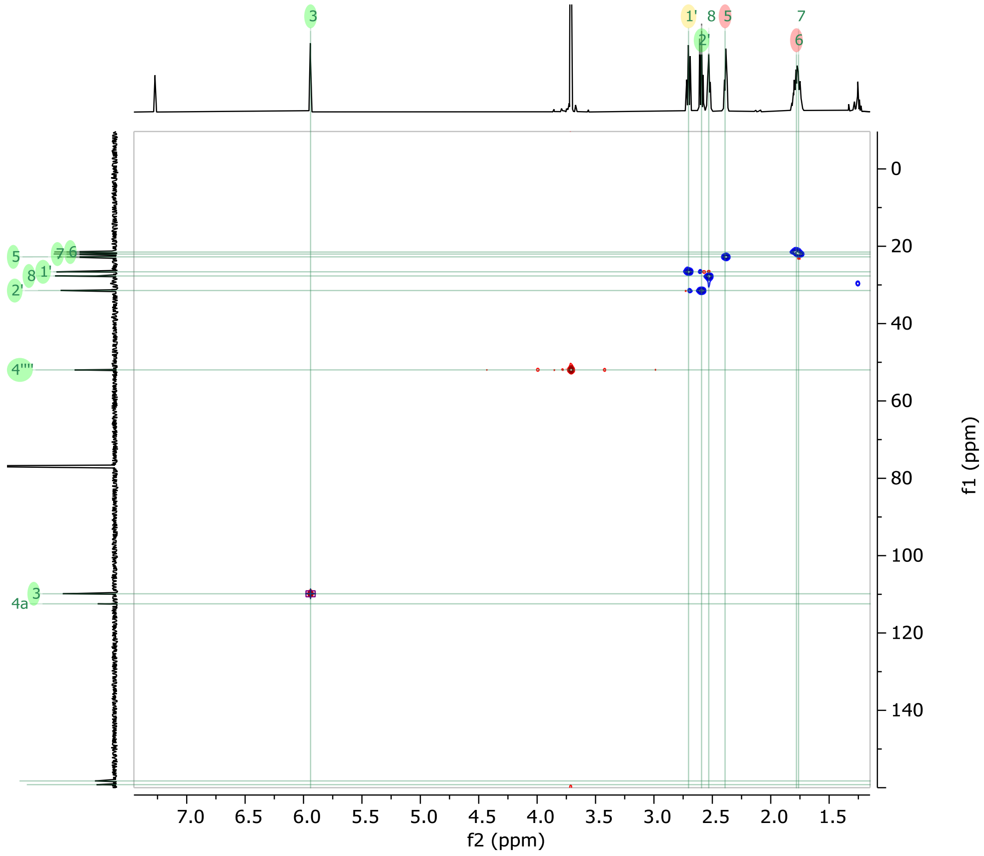

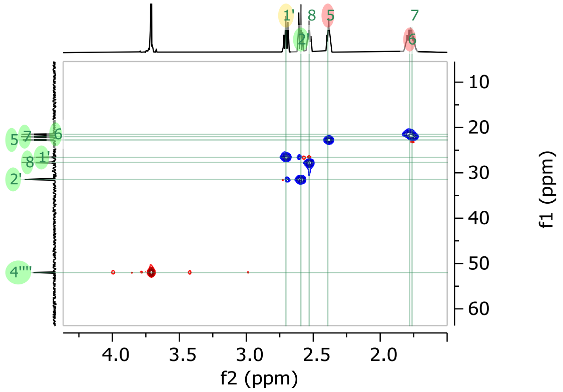

^{1}H NMR, 500 MHz
$CDCl_3$

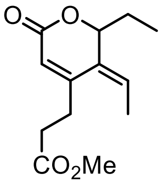

9d

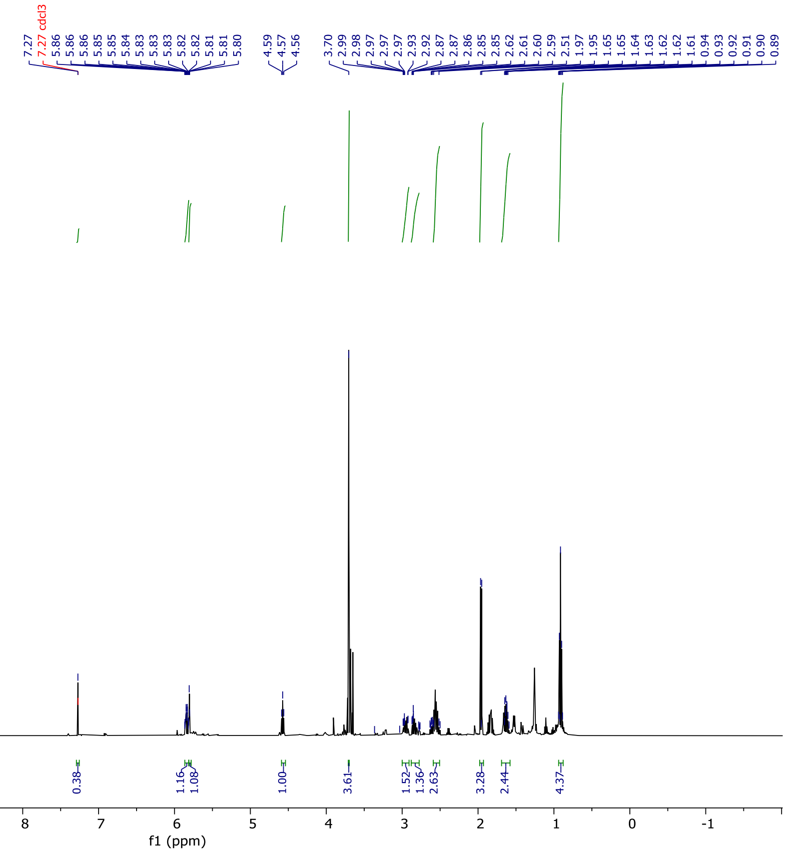

^{13}C{^{1}H} NMR, 126 MHz
$CDCl_3$

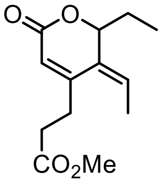

9d

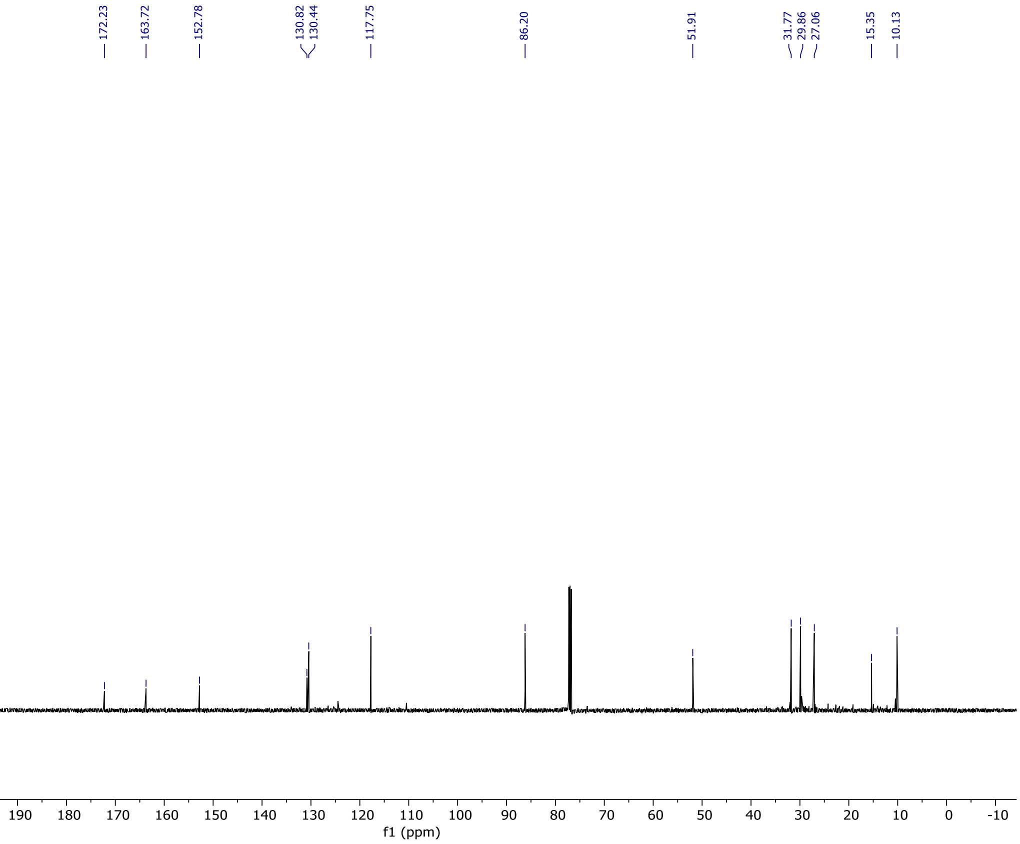

^{1}H NMR, 500 MHz
$CDCl_3$

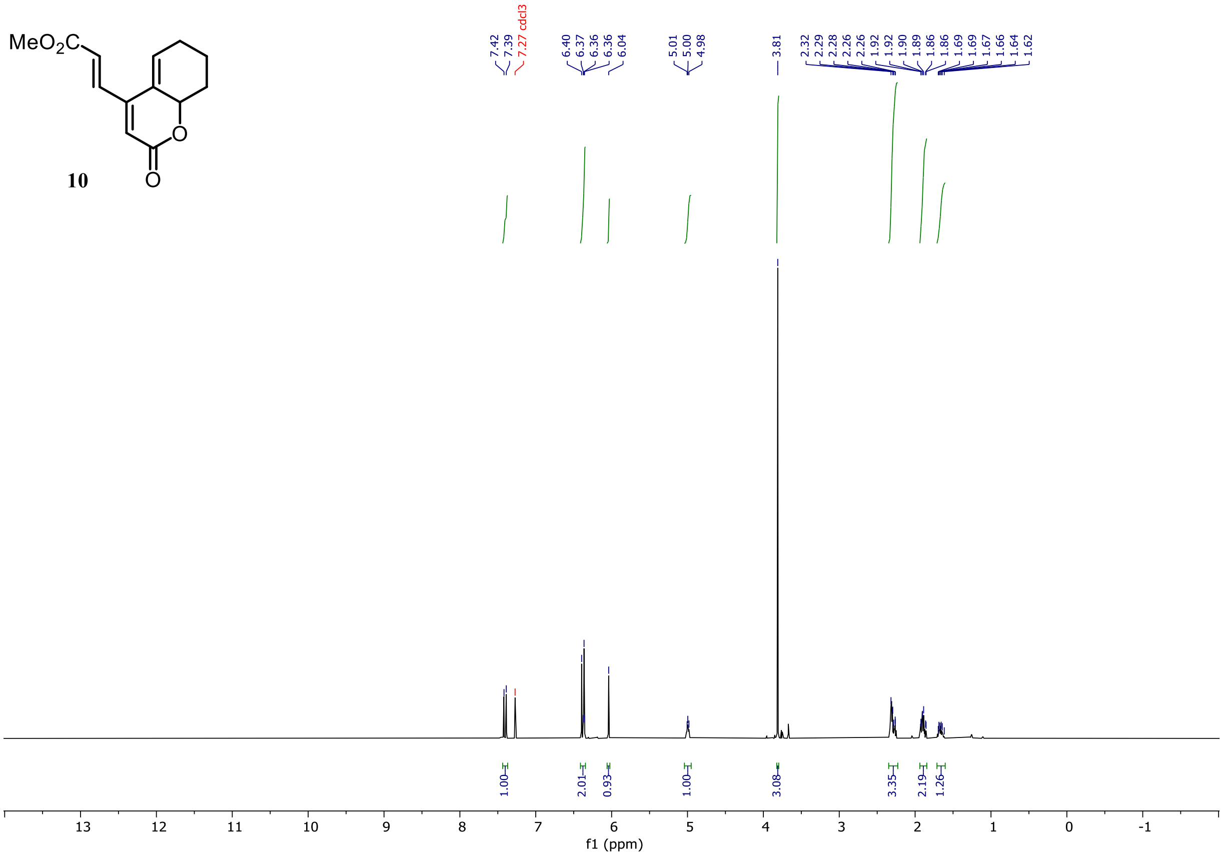

^{13}C{^{1}H} NMR, 126 MHz
$CDCl_3$

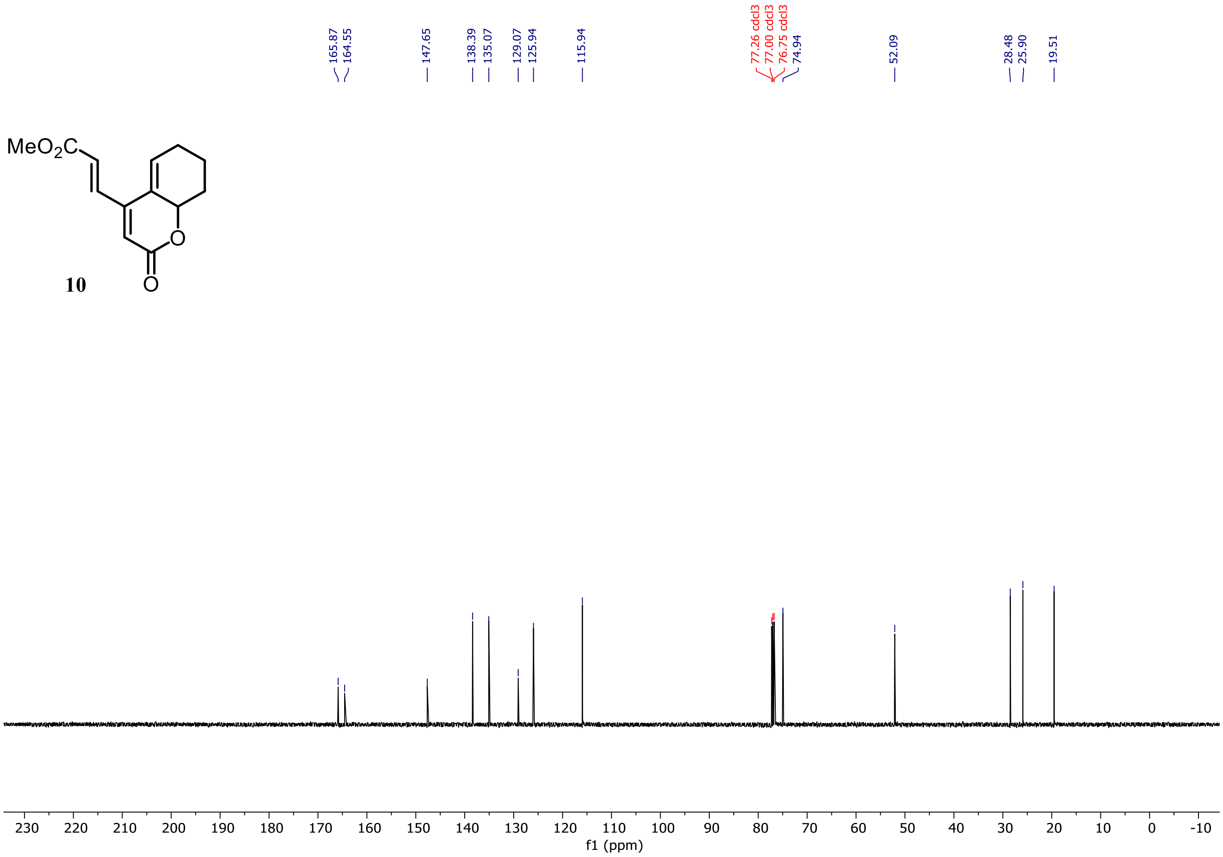

^{1}H NMR, 600 MHz
$CDCl_3$

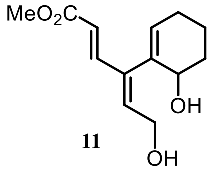

11

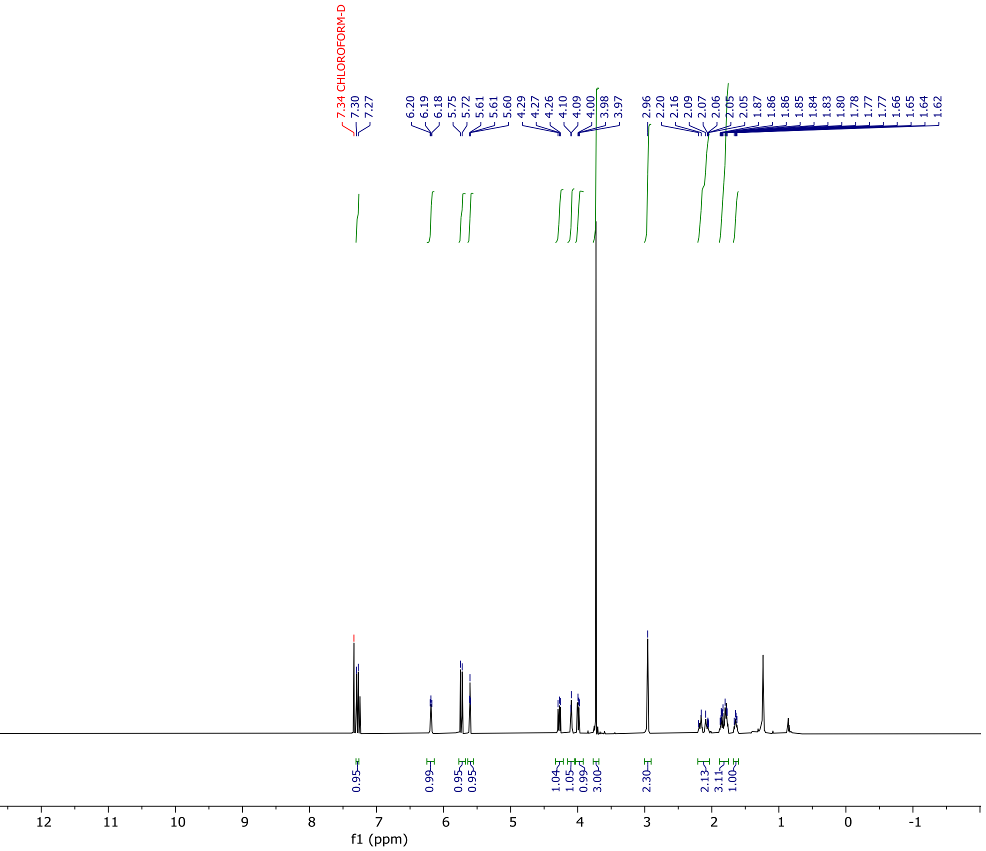

^{13}C{^{1}H} NMR, 151 MHz
$CDCl_3$

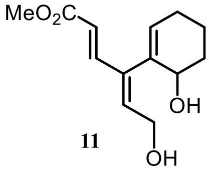

11

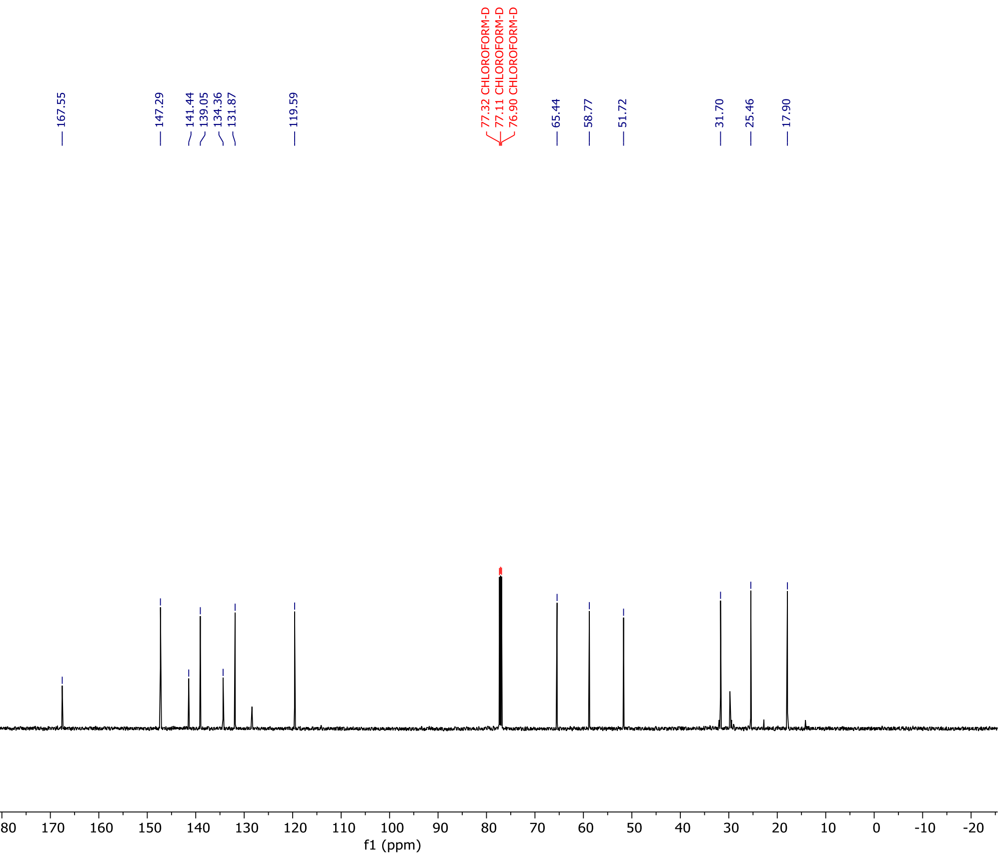

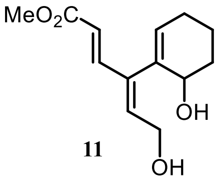

11

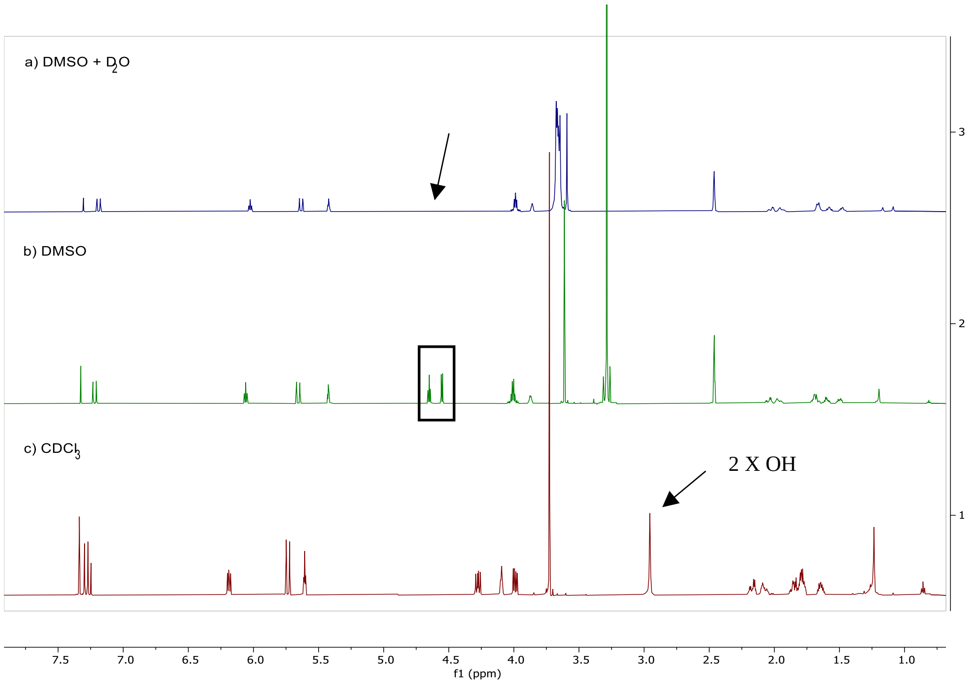

a) Experiment ^{1}H NMR in presence of D_2O and using DMSO as solvent. b) Experiment ^{1}H NMR using DMSO as solvent. c) Experiment ^{1}H NMR using $CDCl_3$ as solvent

^{1}H NMR, 600 MHz
$CDCl_3$

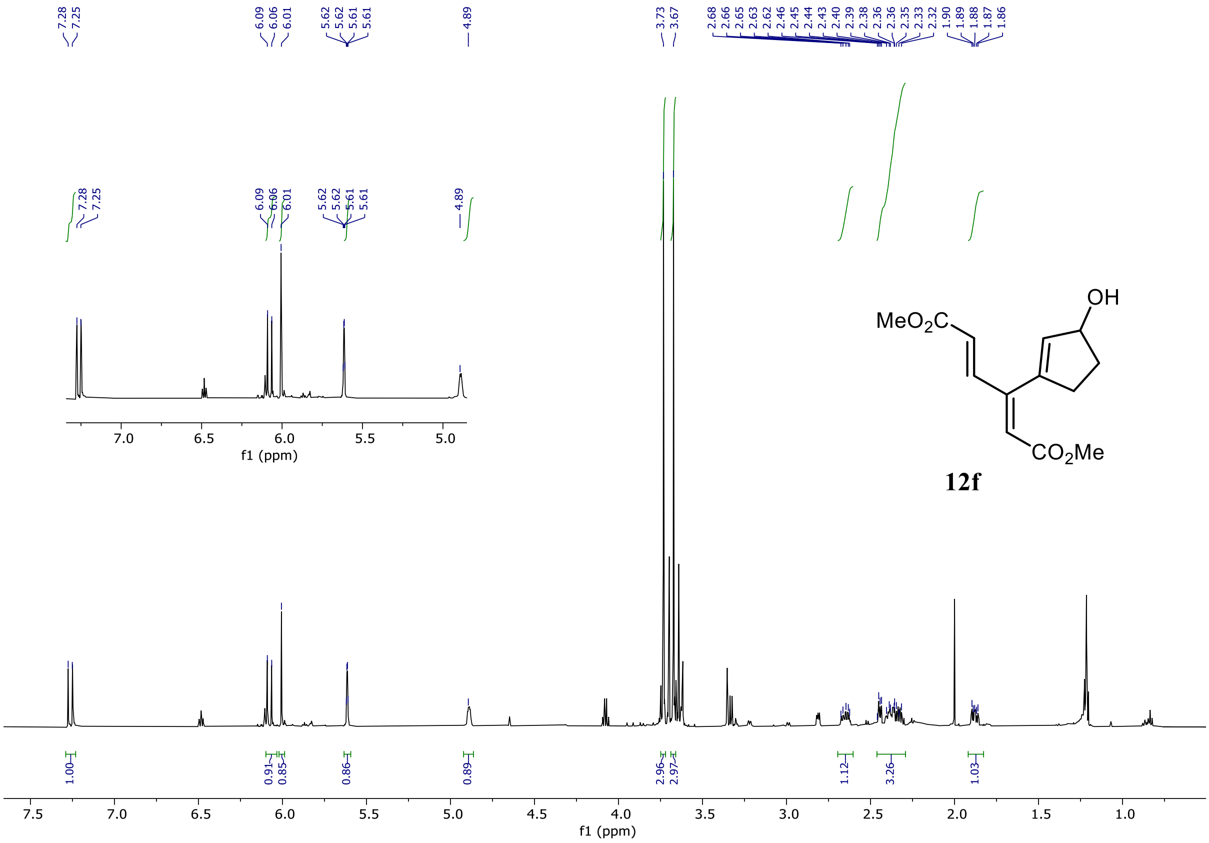

12f

^{1}H NMR, 600 MHz
$CDCl_3$

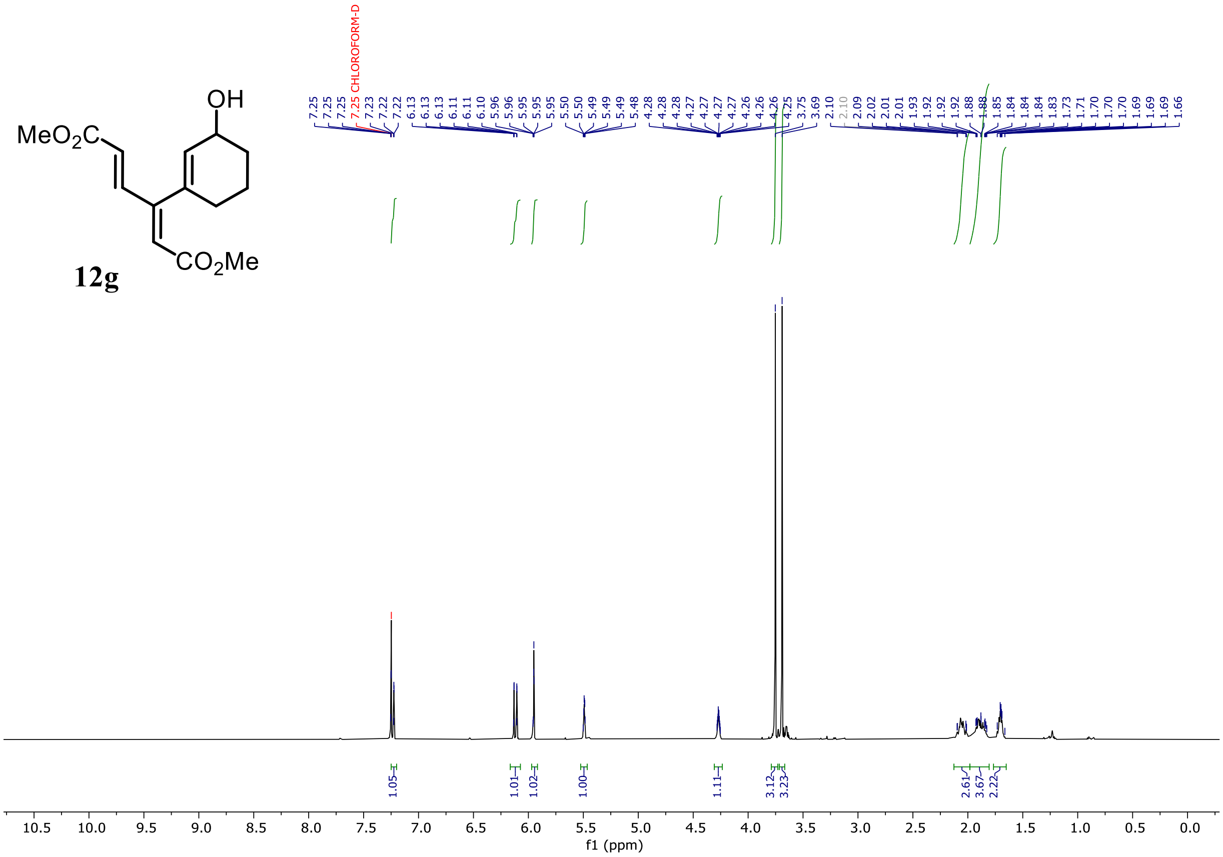

$^{13}C\{^{1}H\}$ NMR, 151 MHz
$CDCl_3$

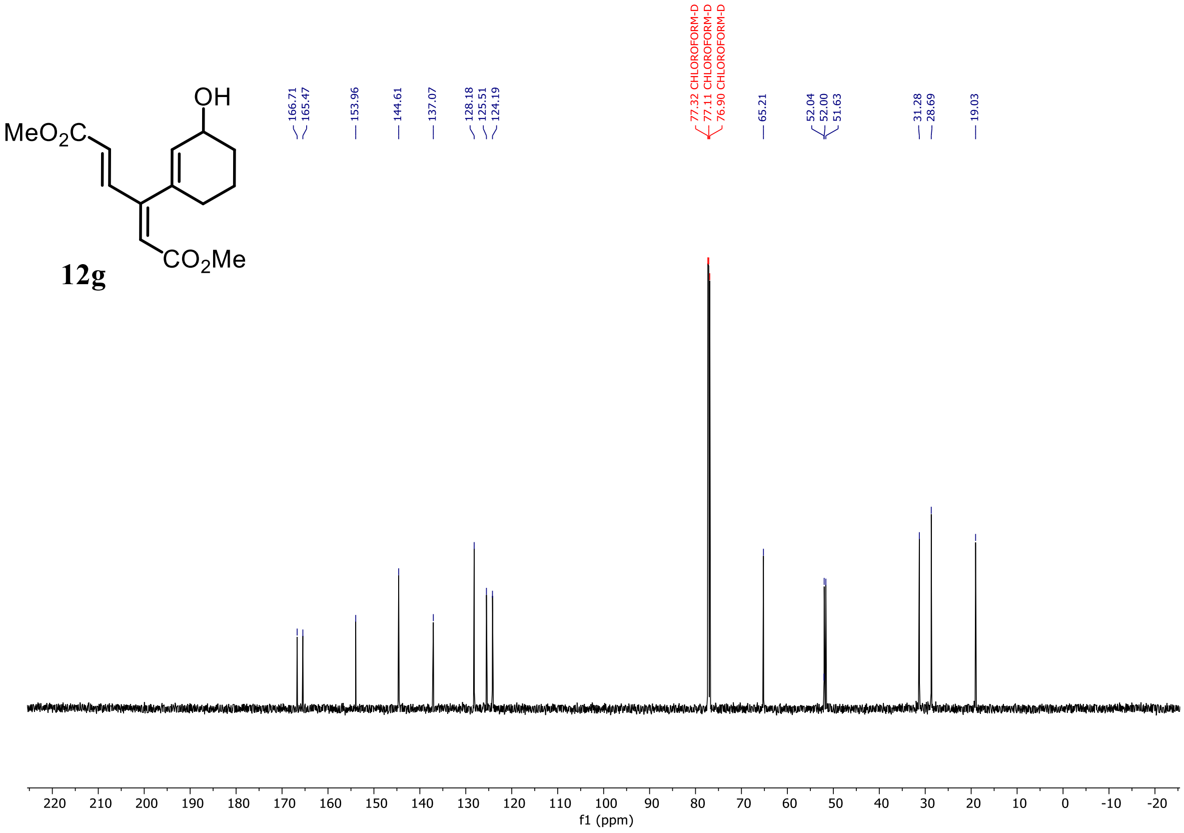

^{1}H NMR, 500 MHz
$CDCl_3$

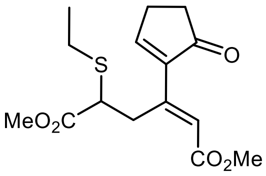

14

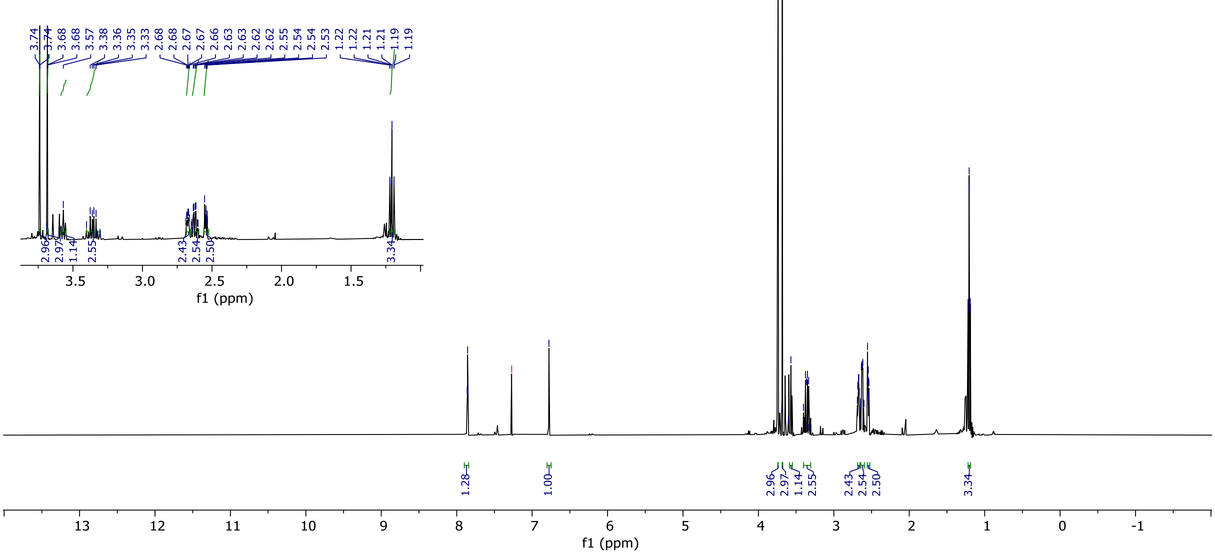

^{13}C{^{1}H} NMR, 126 MHz
$CDCl_3$

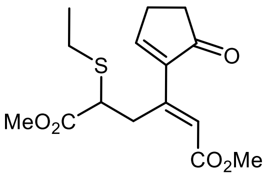

14

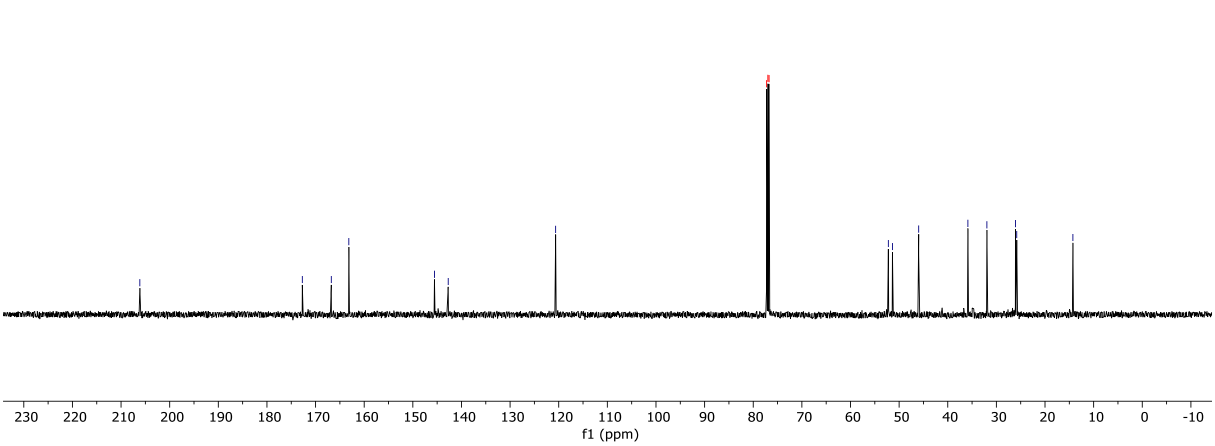

^{1}H NMR, 600 MHz
$CDCl_3$

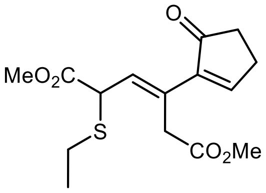

15

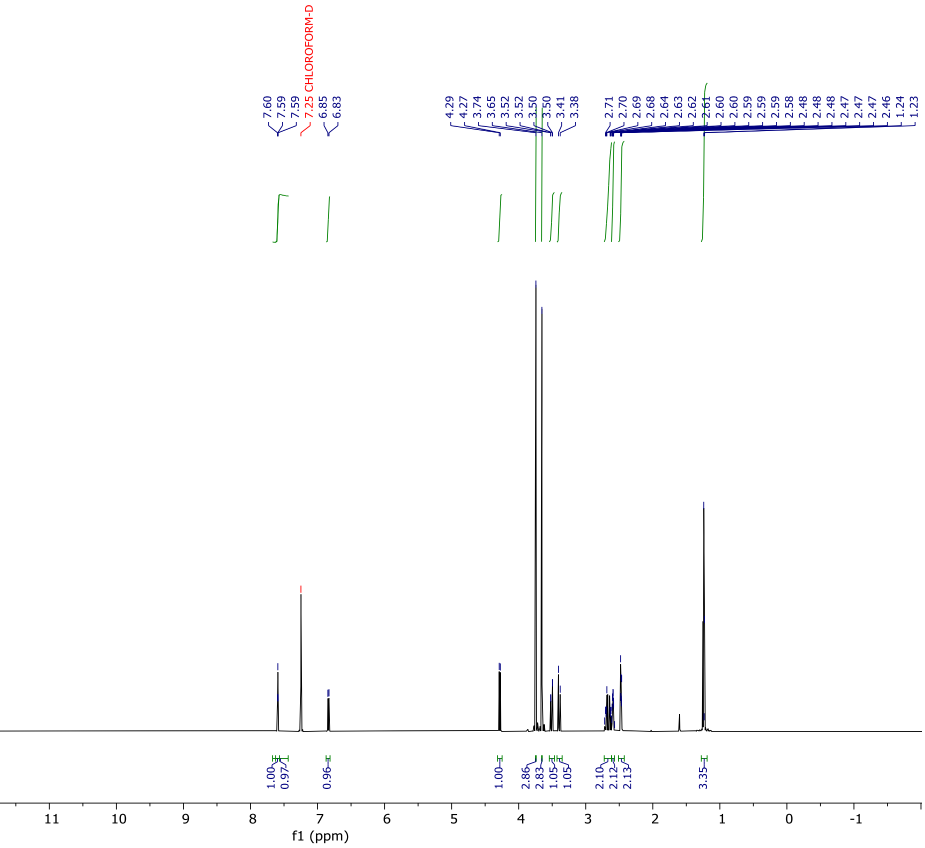

$^{13}C\{^{1}H\}$ NMR, 151 MHz
$CDCl_3$

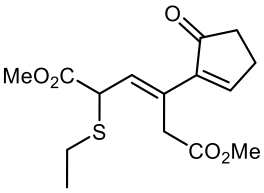

15

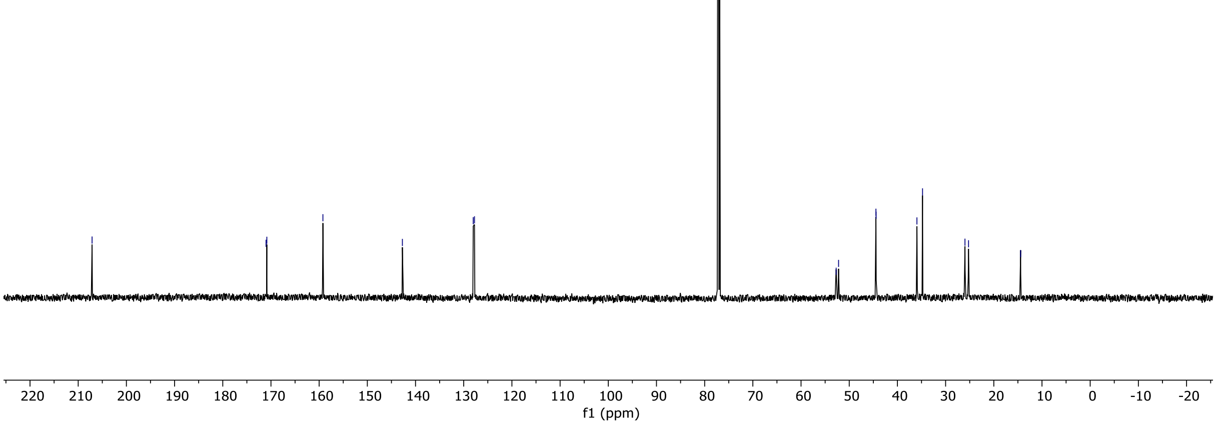

NOESY

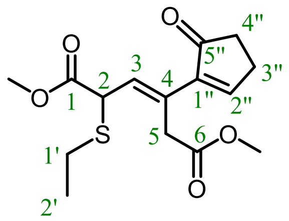

15

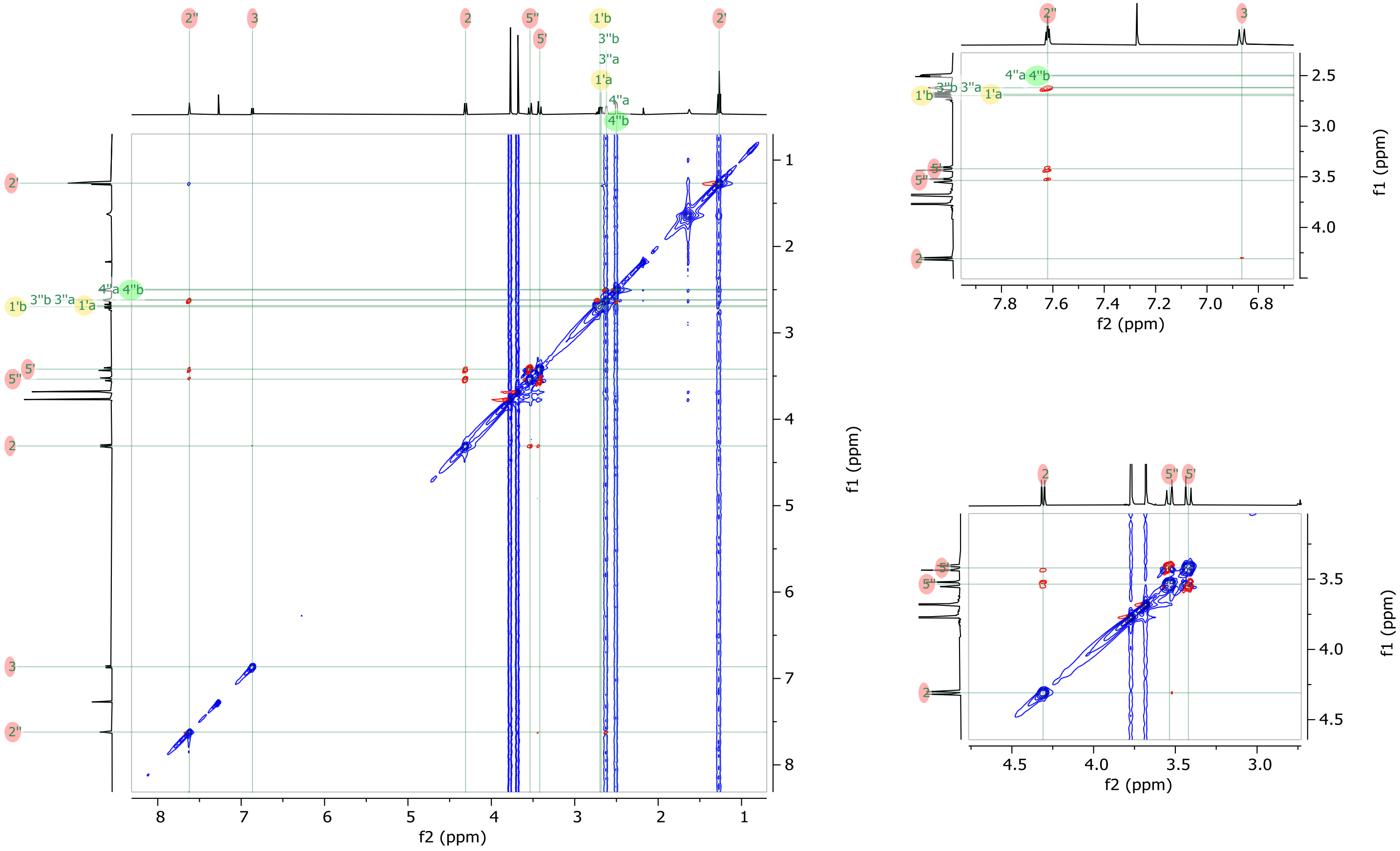

^{1}H NMR, 500 MHz
$CDCl_3$

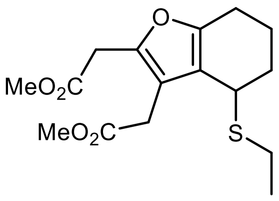

17b

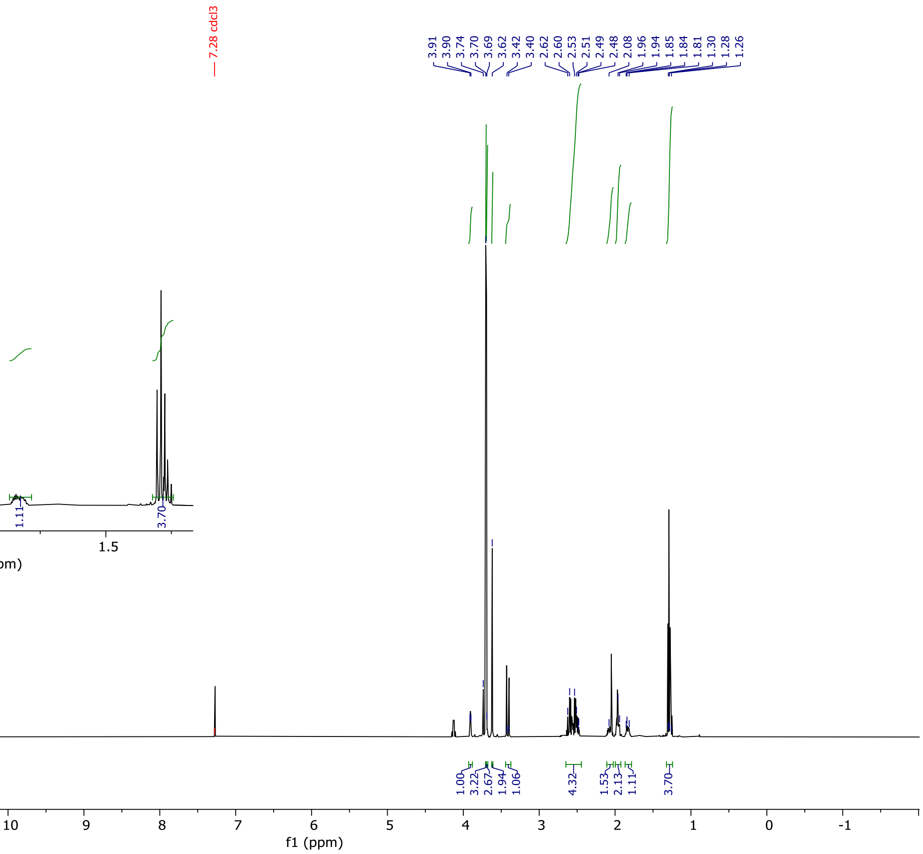

^{13}C{^{1}H} NMR, 126 MHz
$CDCl_3$

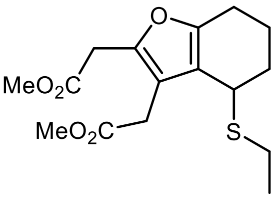

17b

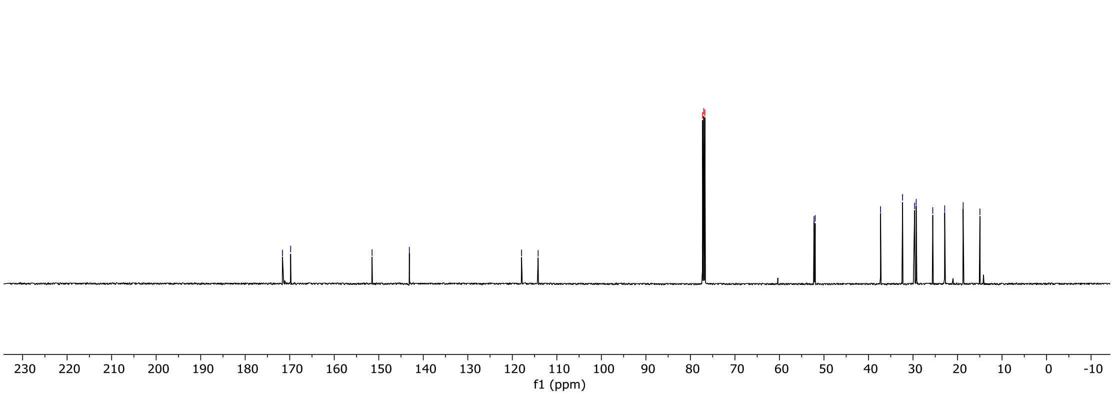

^{1}H NMR, 500 MHz
$CDCl_3$

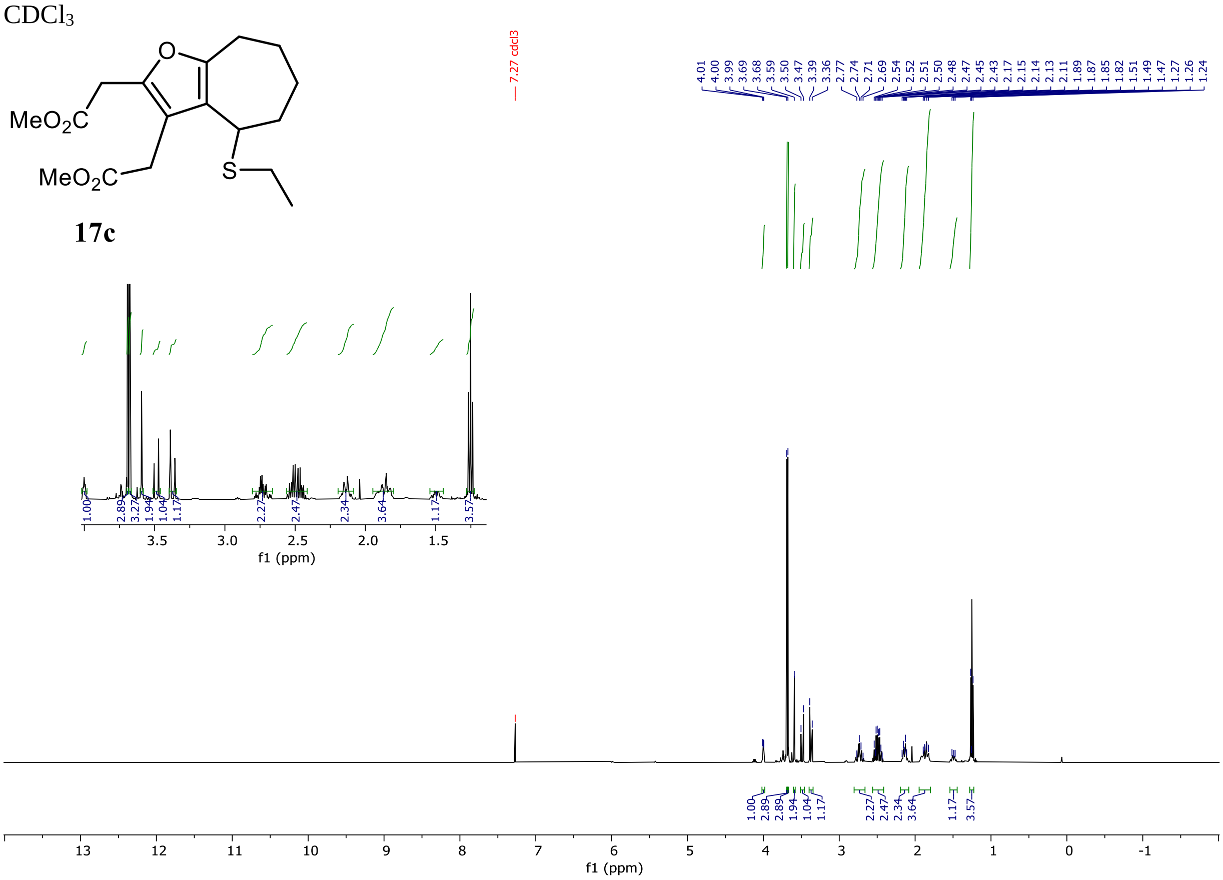

17c

^{13}C{^{1}H} NMR, 126 MHz
$CDCl_3$

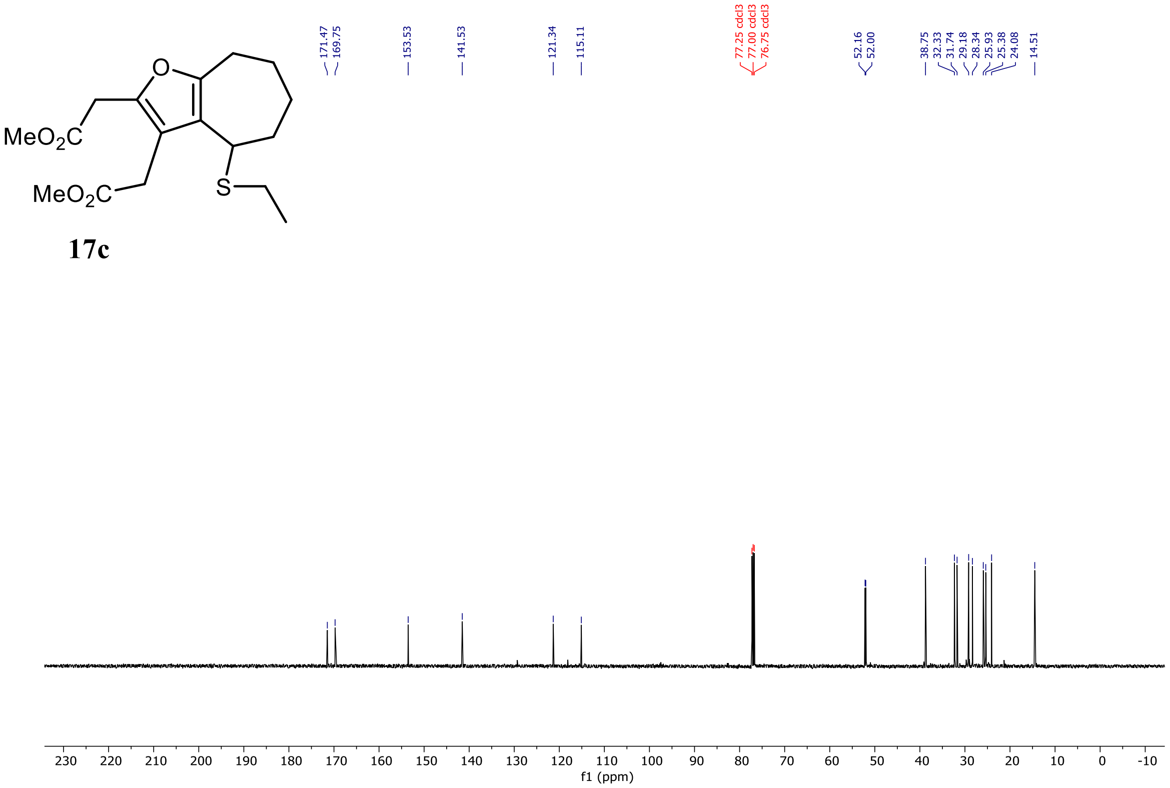

17c

^{1}H NMR, 500 MHz
$CDCl_3$

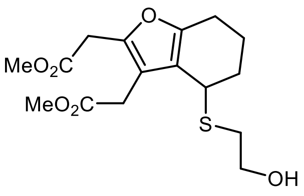

18b

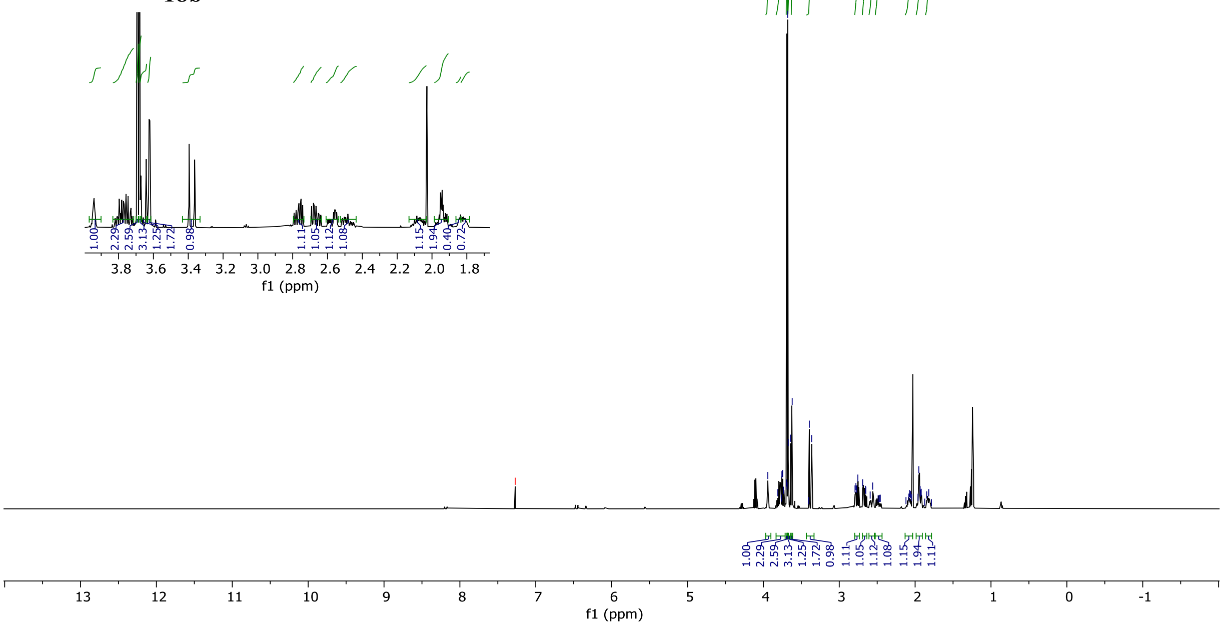

$^{13}C\{^{1}H\}$ NMR, 126 MHz
$CDCl_3$

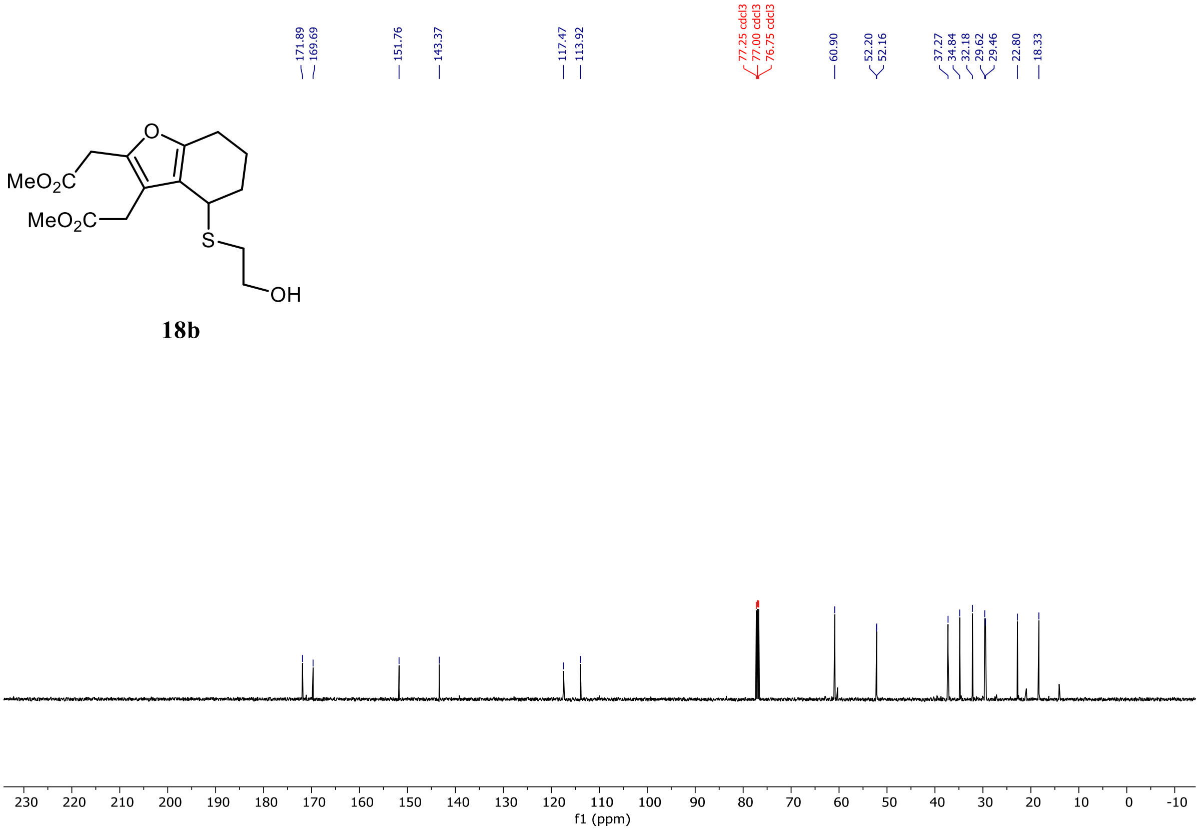

^{1}H NMR, 500 MHz
$CDCl_3$

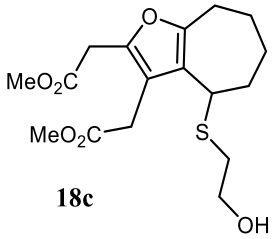

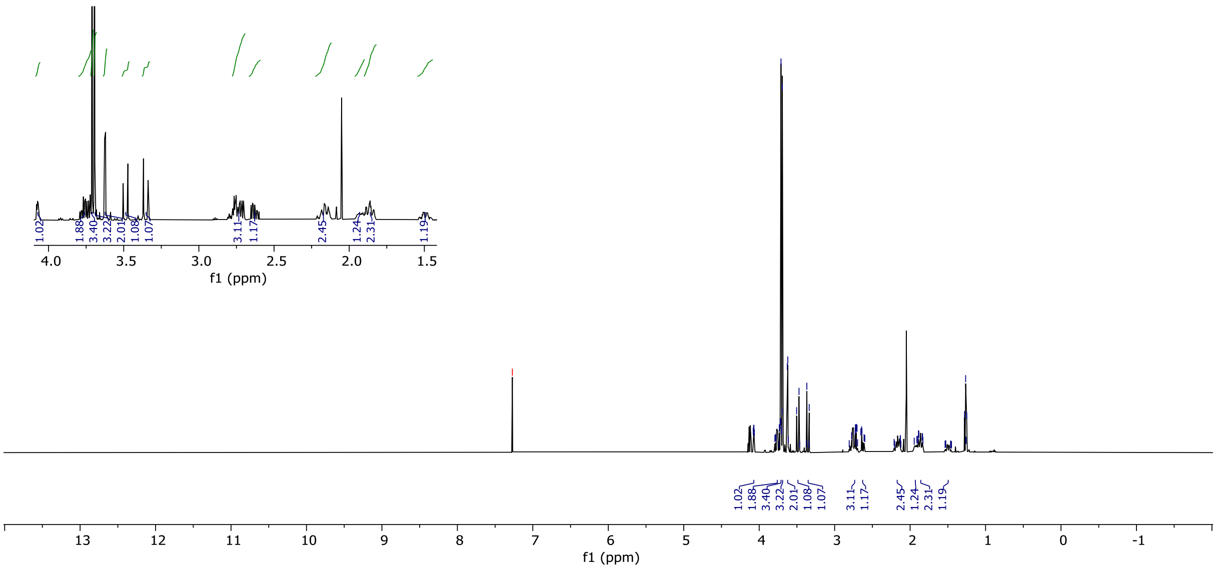

$^{13}C\{^{1}H\}$ NMR, 126 MHz
$CDCl_3$

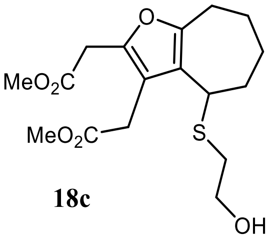

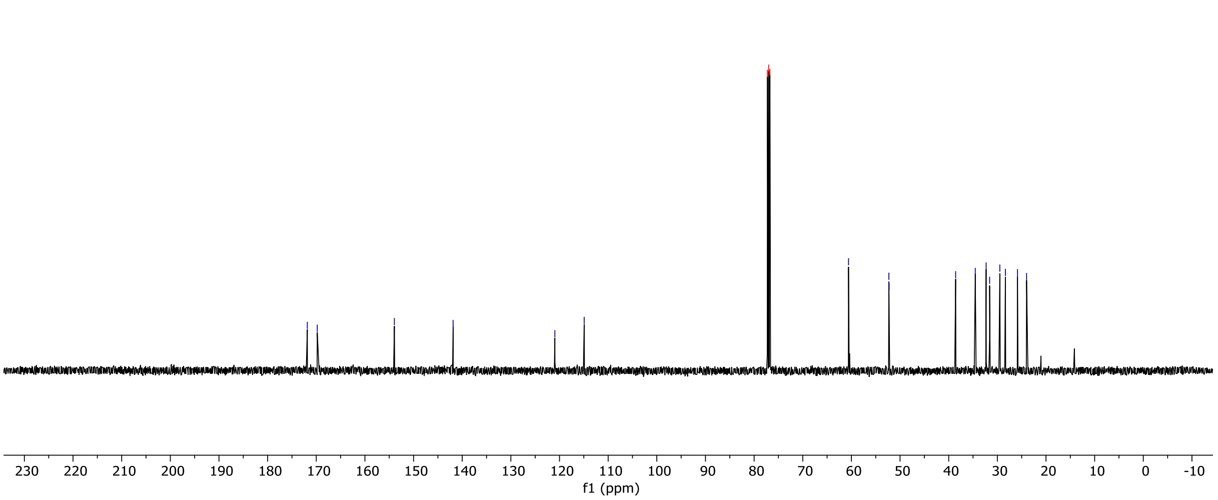

^{1}H NMR, 500 MHz
$CDCl_3$

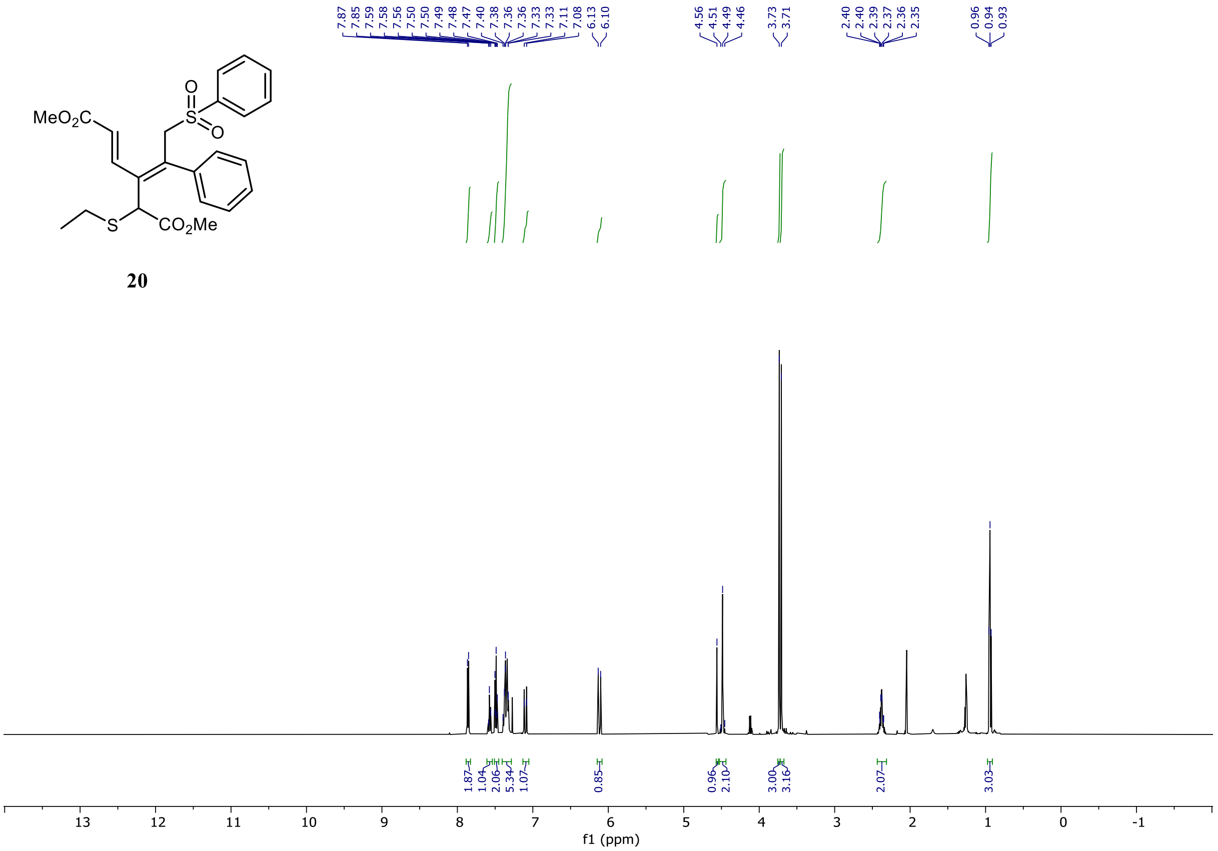

20

^{13}C{^{1}H} NMR, 126 MHz
$CDCl_3$

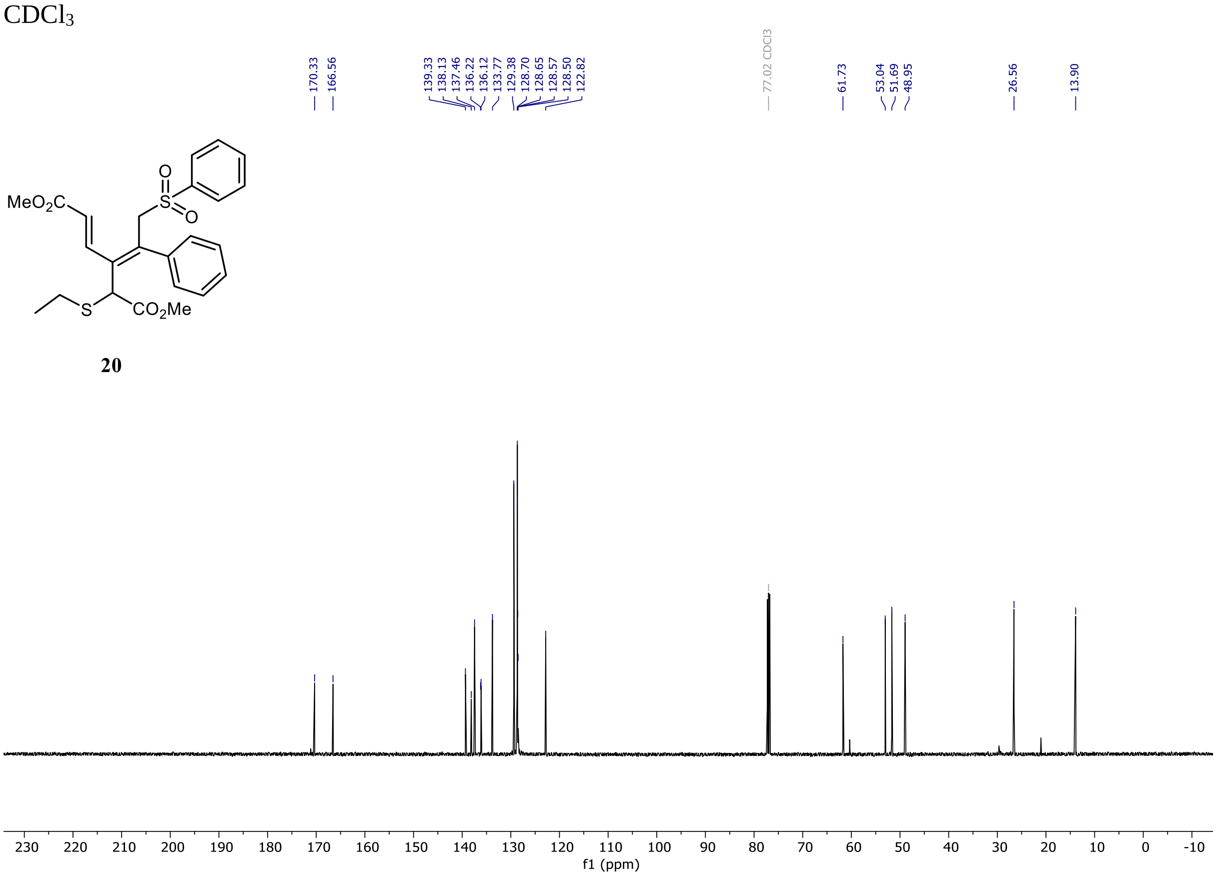

20

NOESY

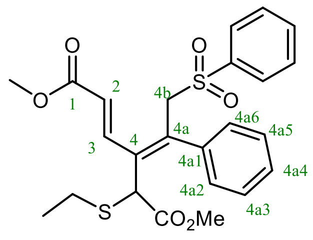

20

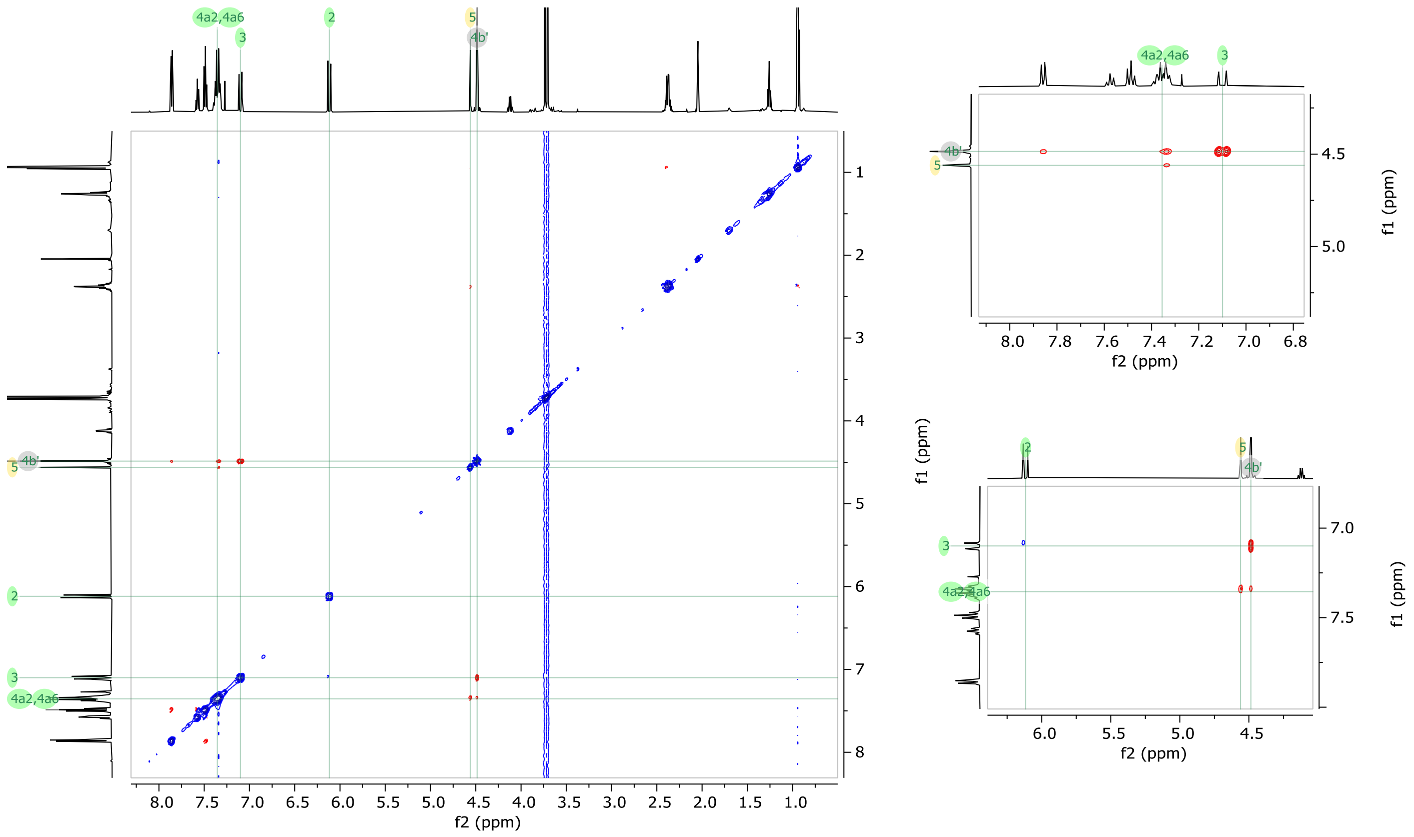

^{1}H NMR, 500 MHz
$CDCl_3$

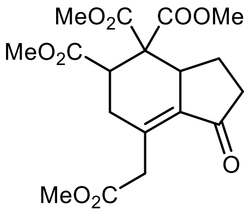

24x

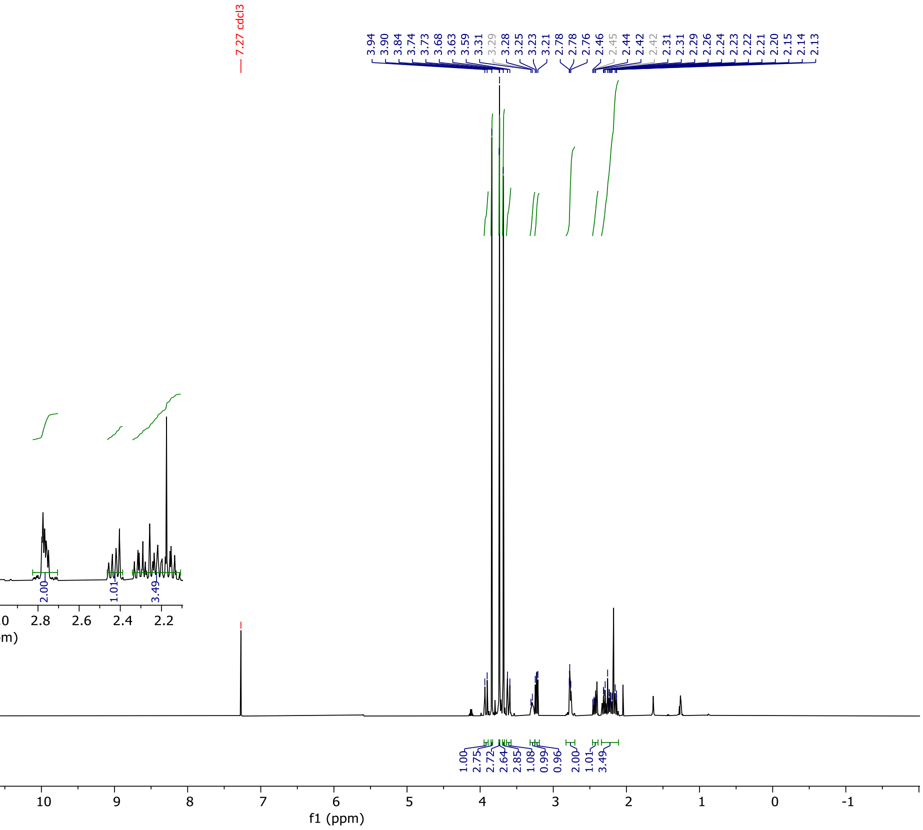

$^{13}C\{^{1}H\}$ NMR, 126 MHz
$CDCl_3$

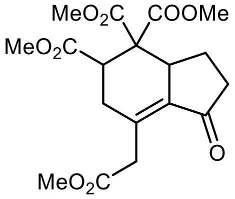

24x

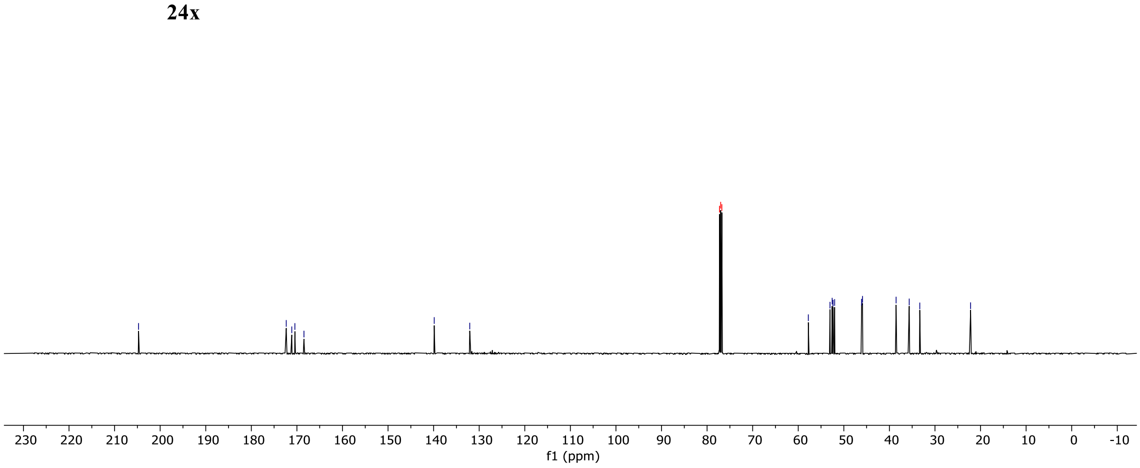

^{1}H NMR, 500 MHz
$CDCl_3$

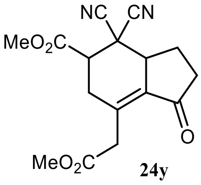

24y

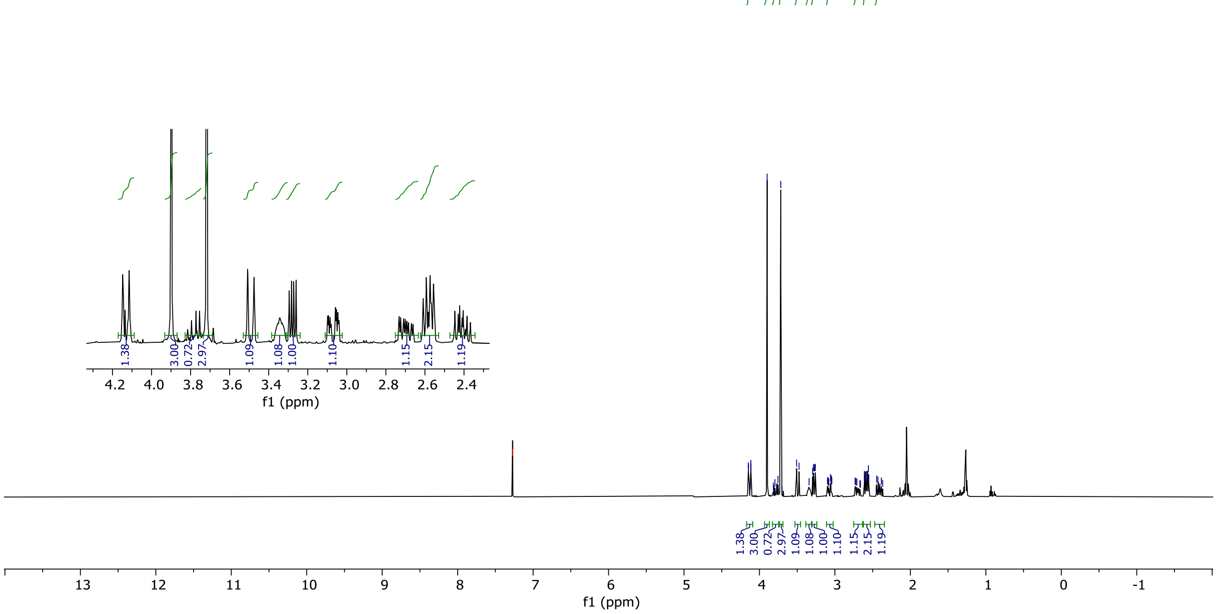

^{13}C{^{1}H} NMR, 126 MHz
$CDCl_3$

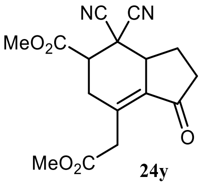

24y

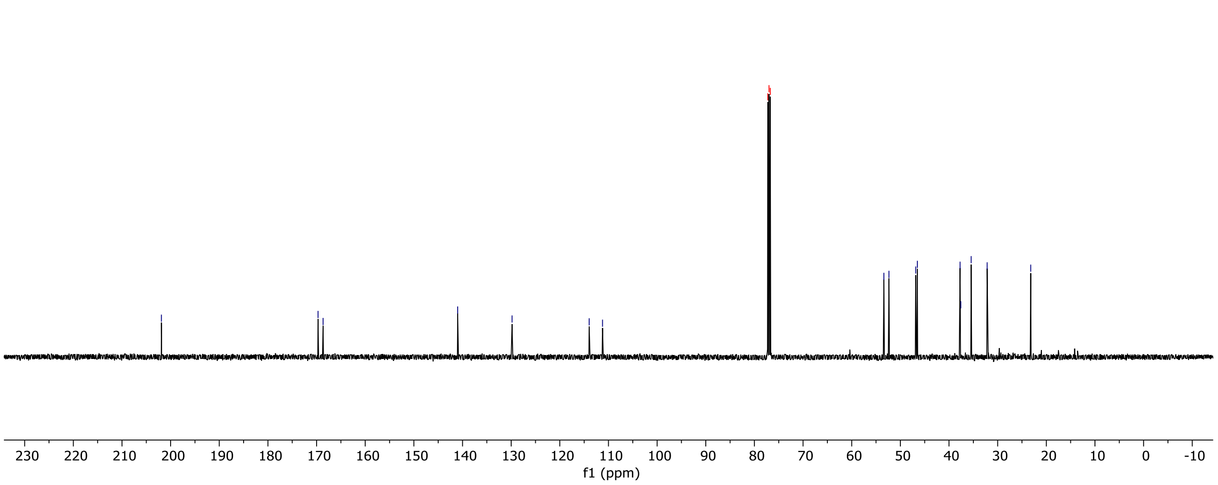

^1H NMR, 500 MHz
$CDCl_3$

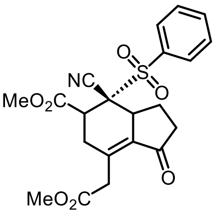

24z

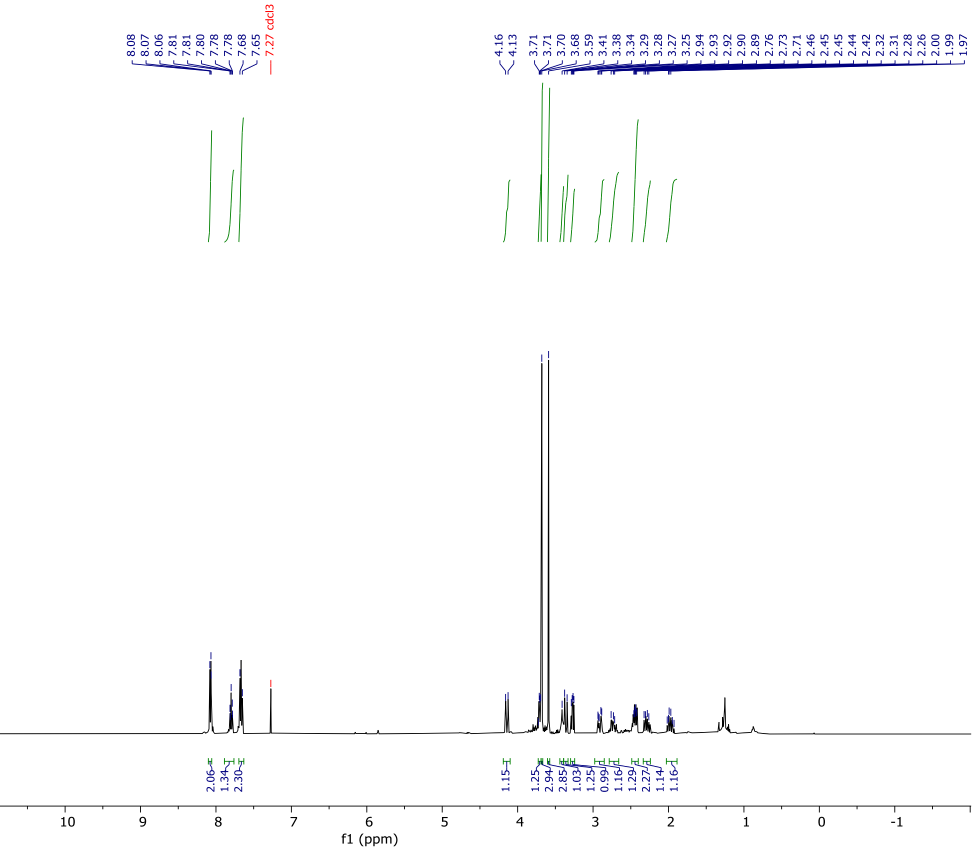

$^{13}C\{^1H\}$ NMR, 151 MHz
$CDCl_3$

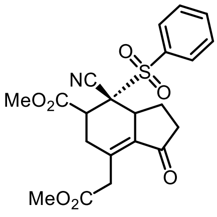

24z

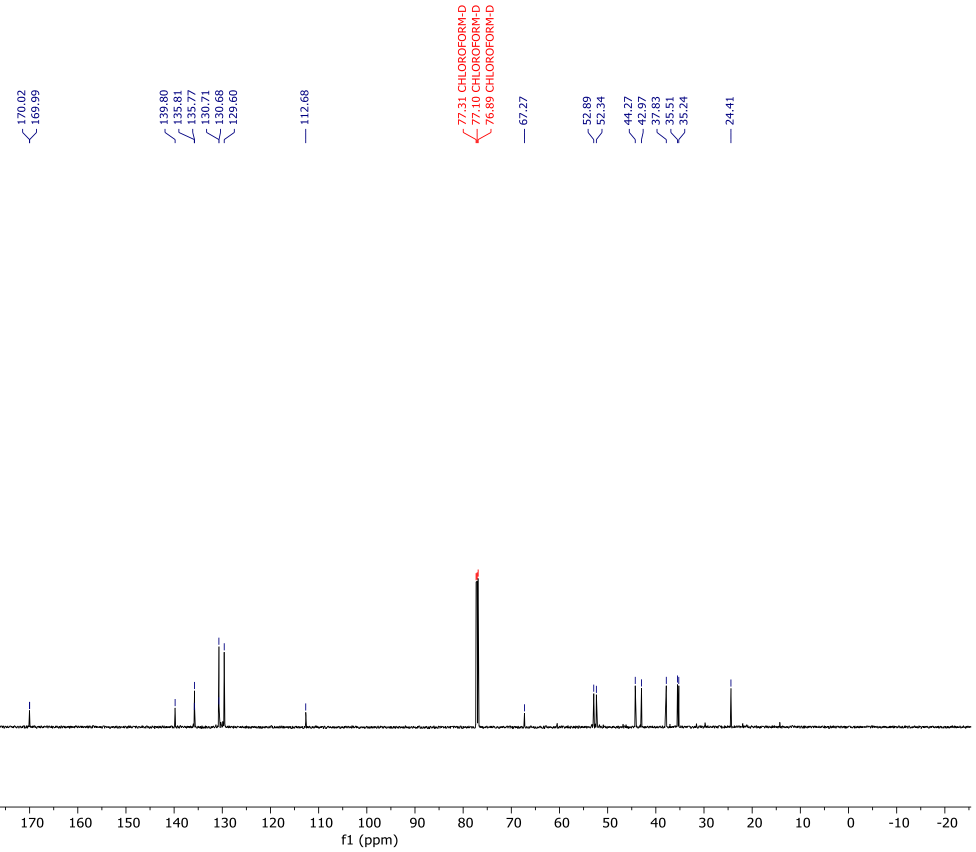

^{1}H NMR, 500 MHz
$CDCl_3$

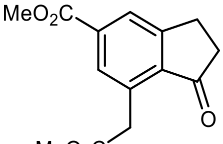

28

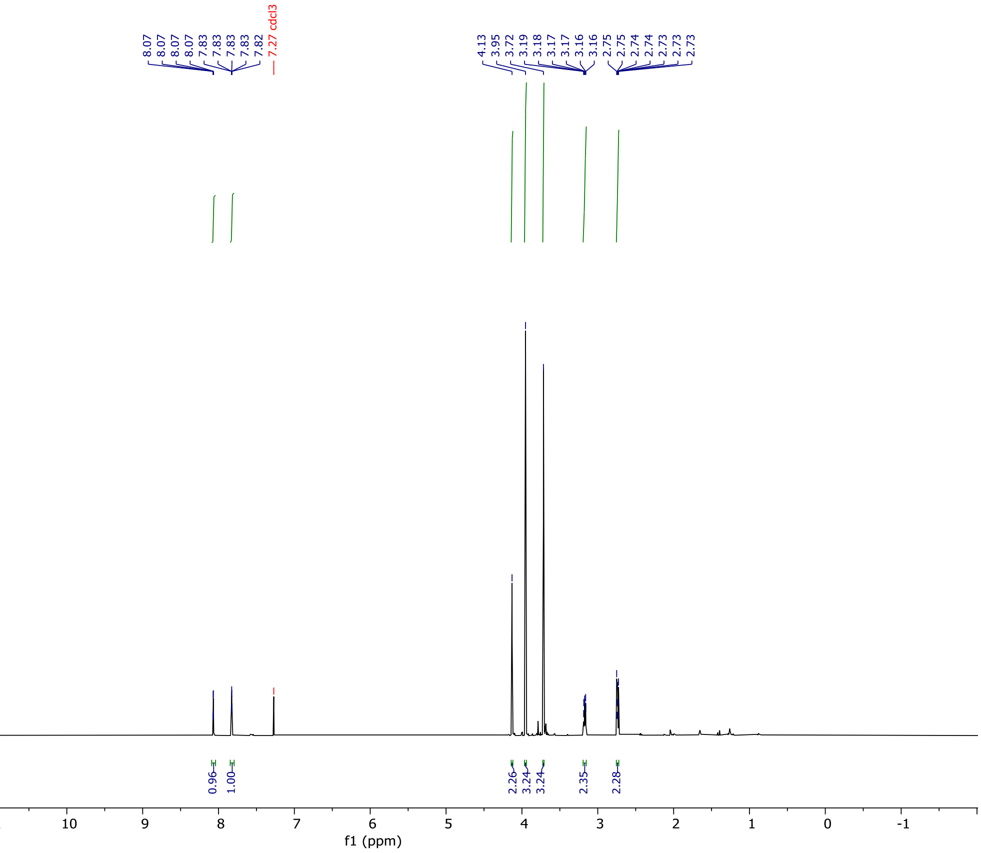

^{13}C{^{1}H} NMR, 126 MHz
$CDCl_3$

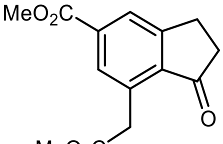

28

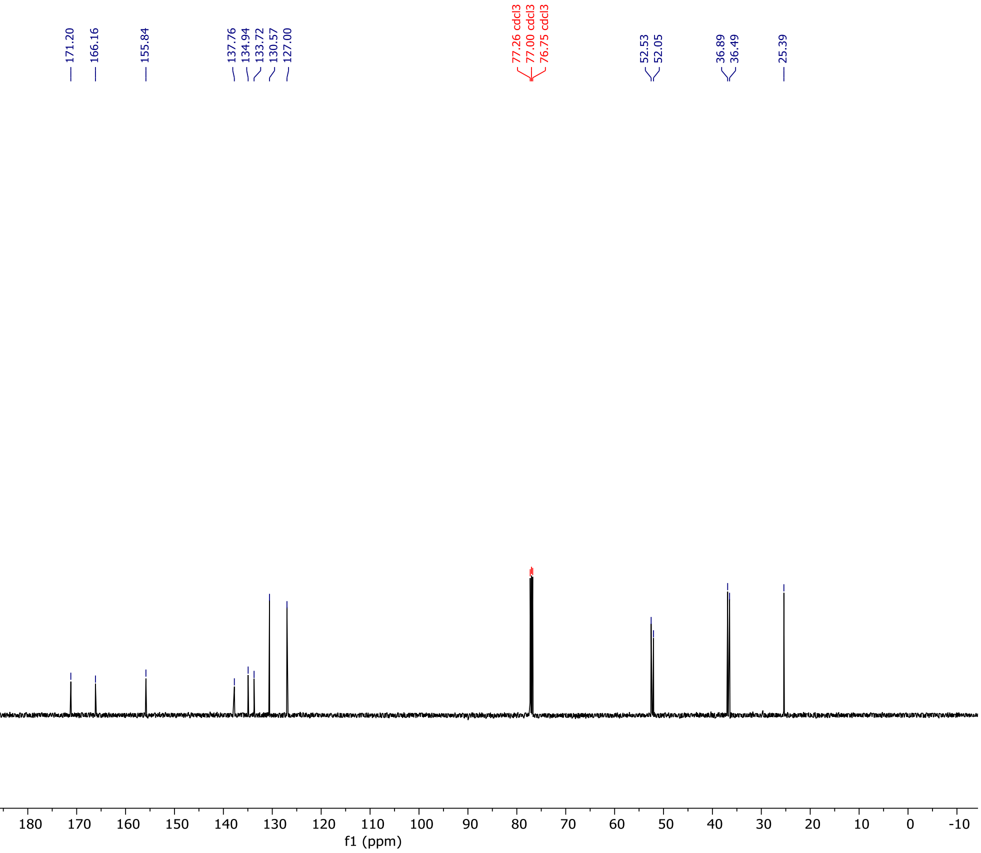

^{1}H NMR, 500 MHz
$CDCl_3$

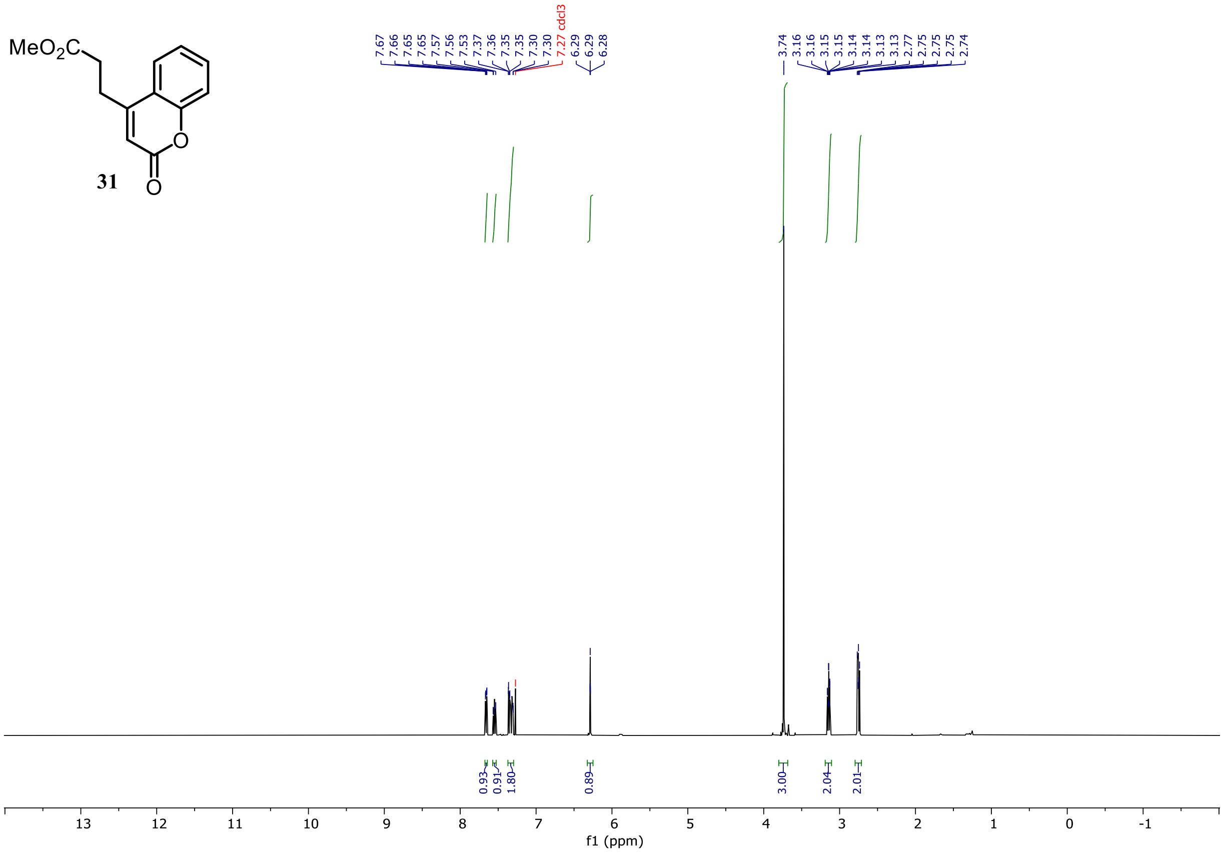

^{13}C{^{1}H} NMR, 126 MHz
$CDCl_3$

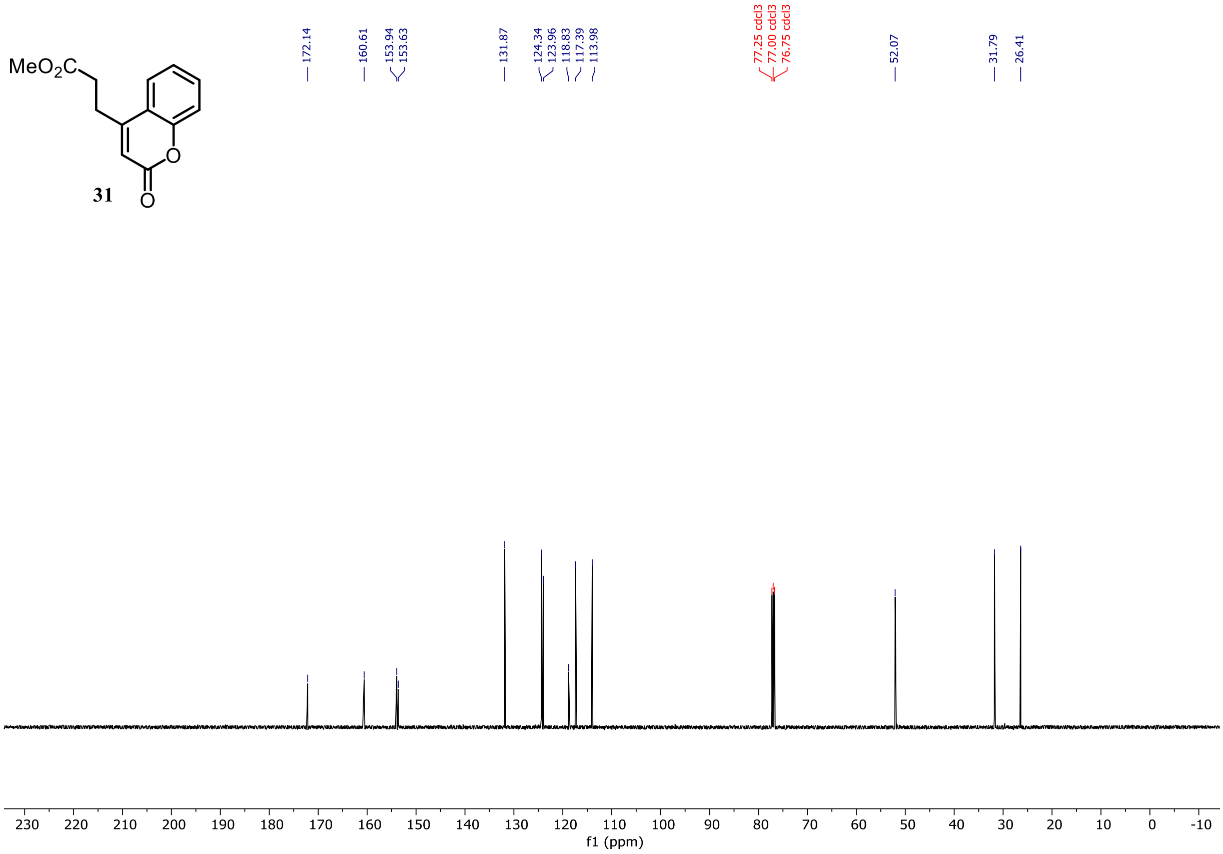

^{1}H NMR, 600 MHz
$CDCl_3$

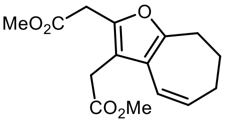

33

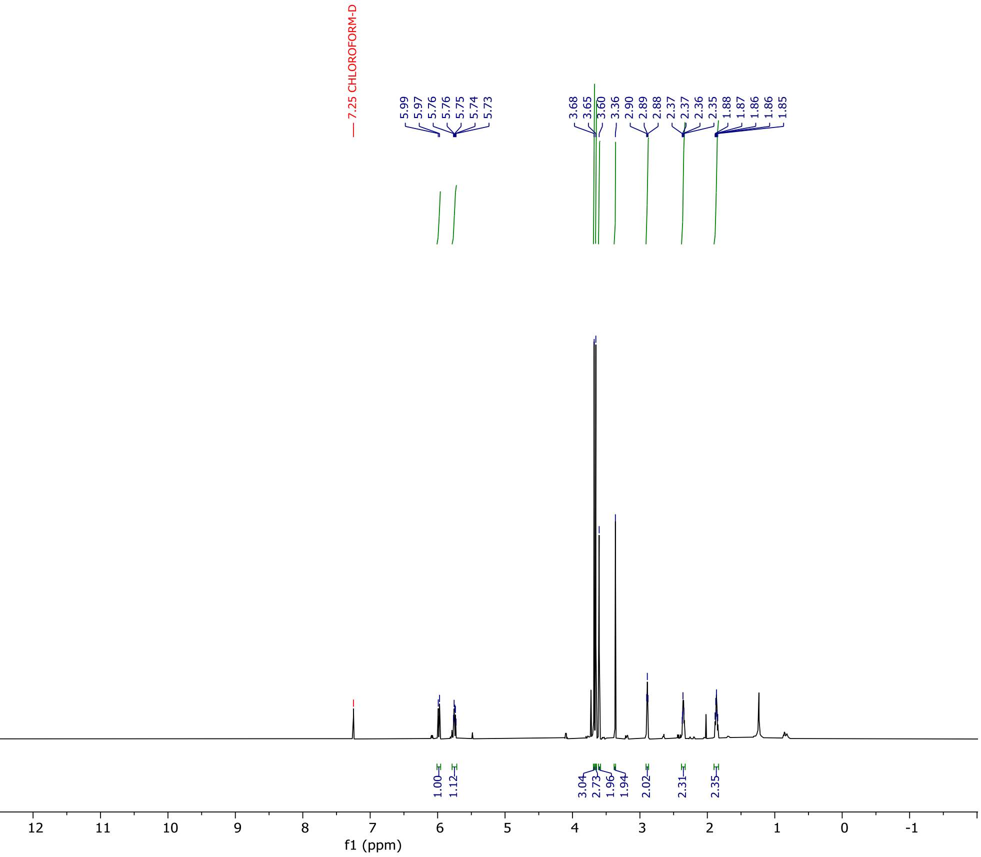

^{13}C{^{1}H} NMR, 151 MHz
$CDCl_3$

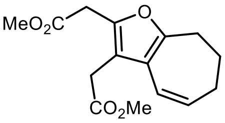

33

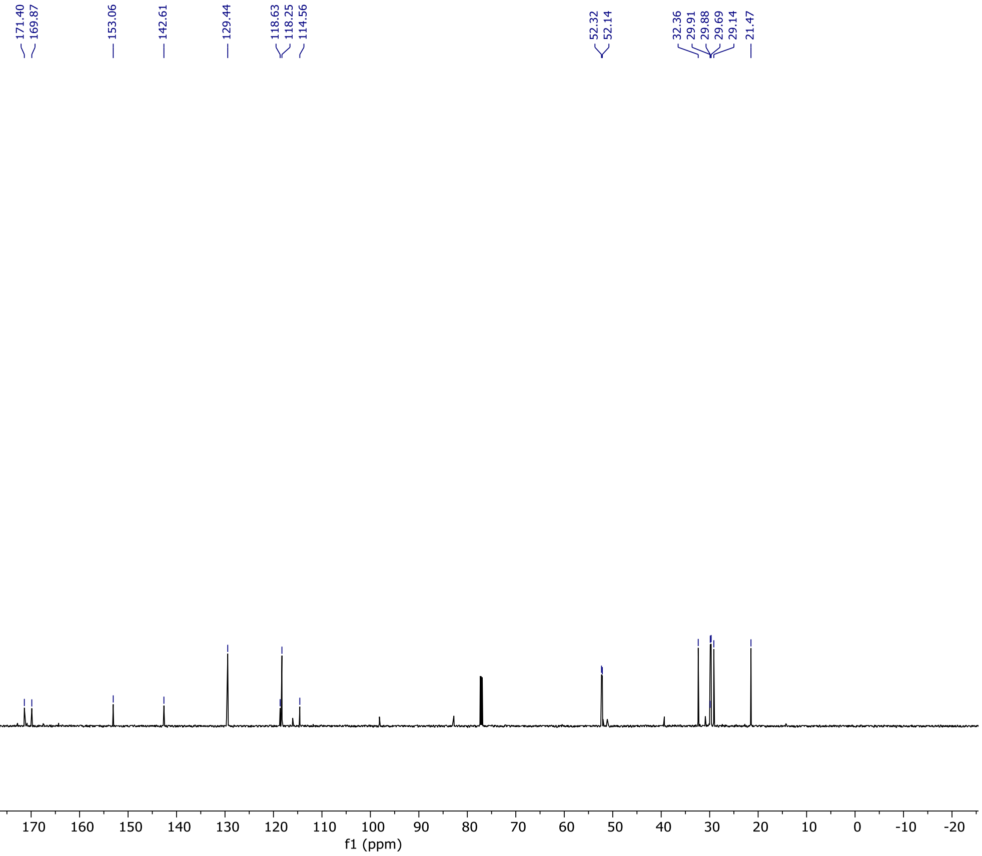

^{1}H NMR, 500 MHz
$CDCl_3$

35

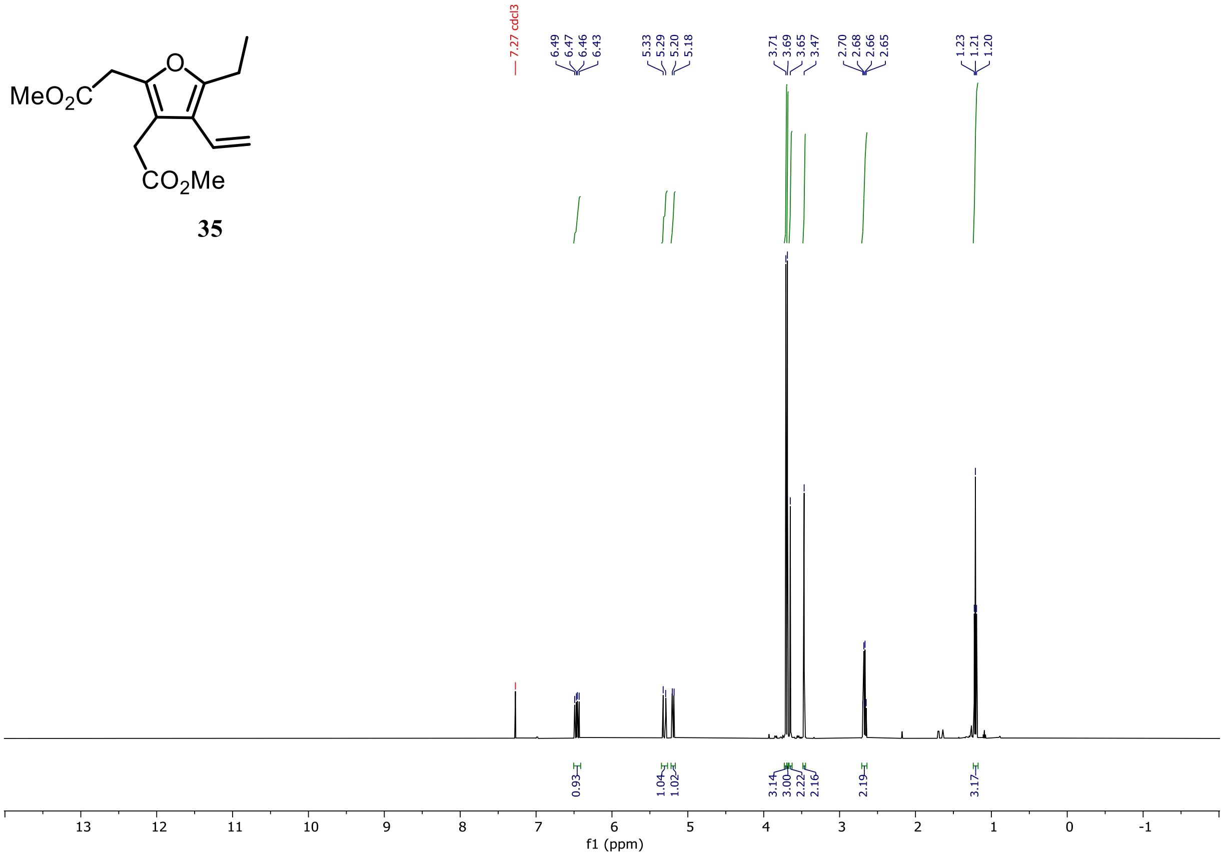

$^{13}C\{^{1}H\}$ NMR, 126 MHz
$CDCl_3$

35

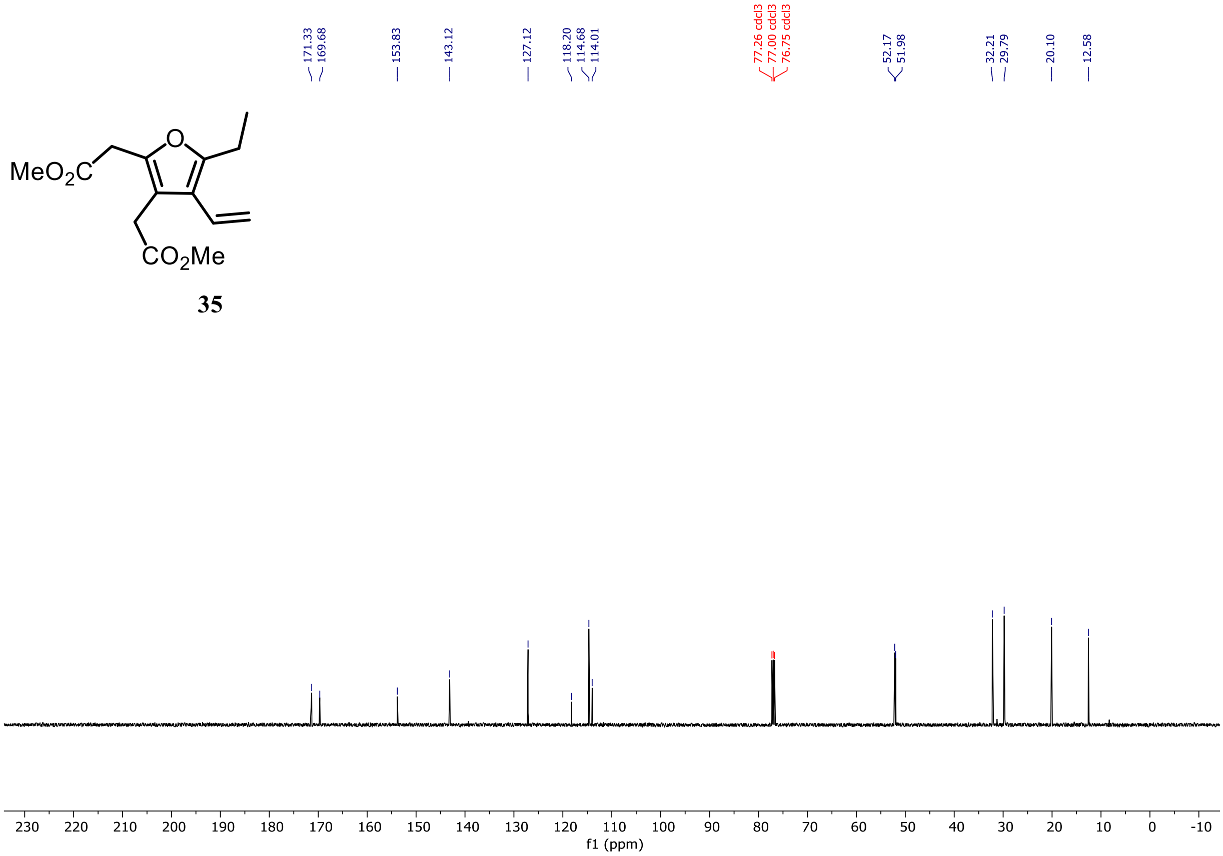

^{1}H-$^{13}C\{^{1}H\}$ HSQC

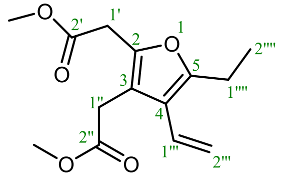

35

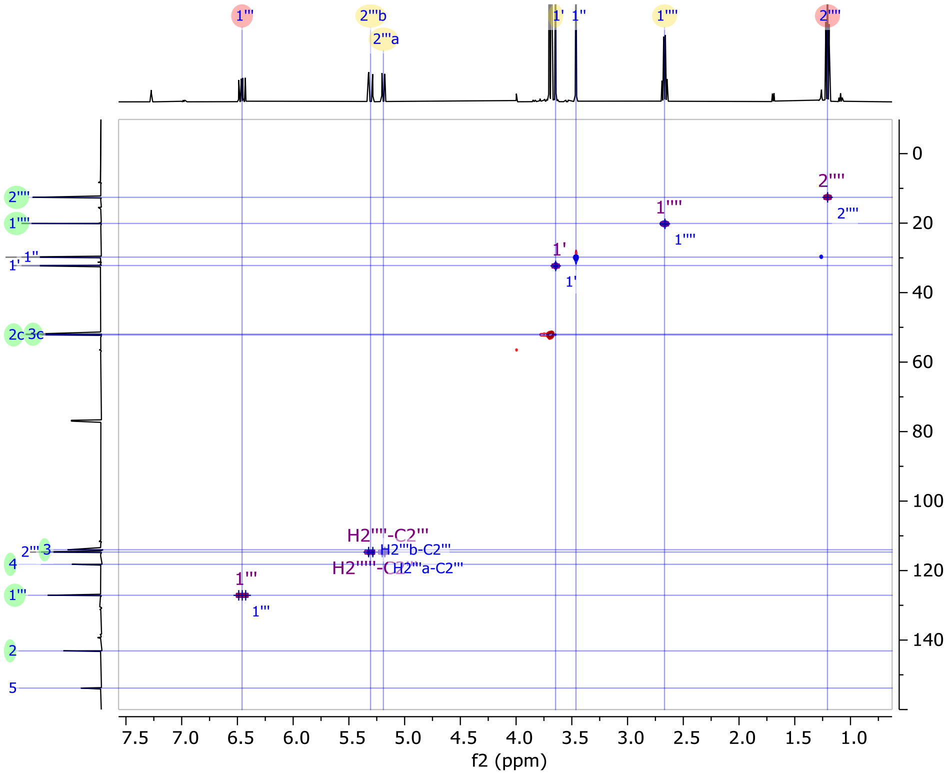

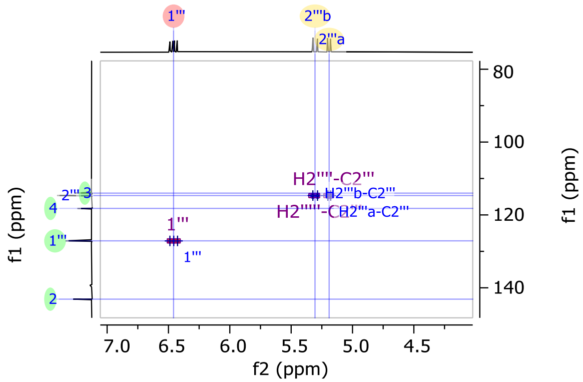

^{1}H NMR, 600 MHz
$CDCl_3$

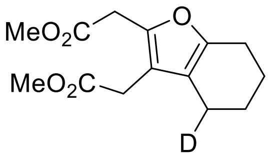

7b-*d*

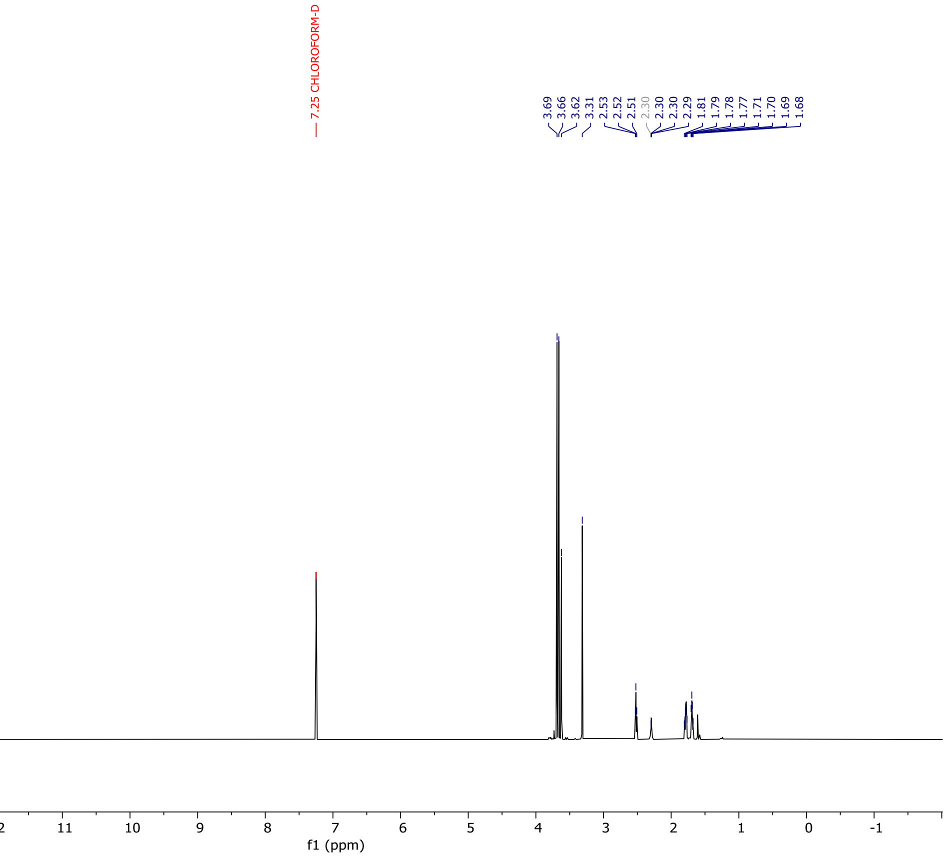

^{13}C{^{1}H} NMR, 151 MHz
$CDCl_3$

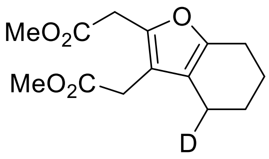

7b-*d*

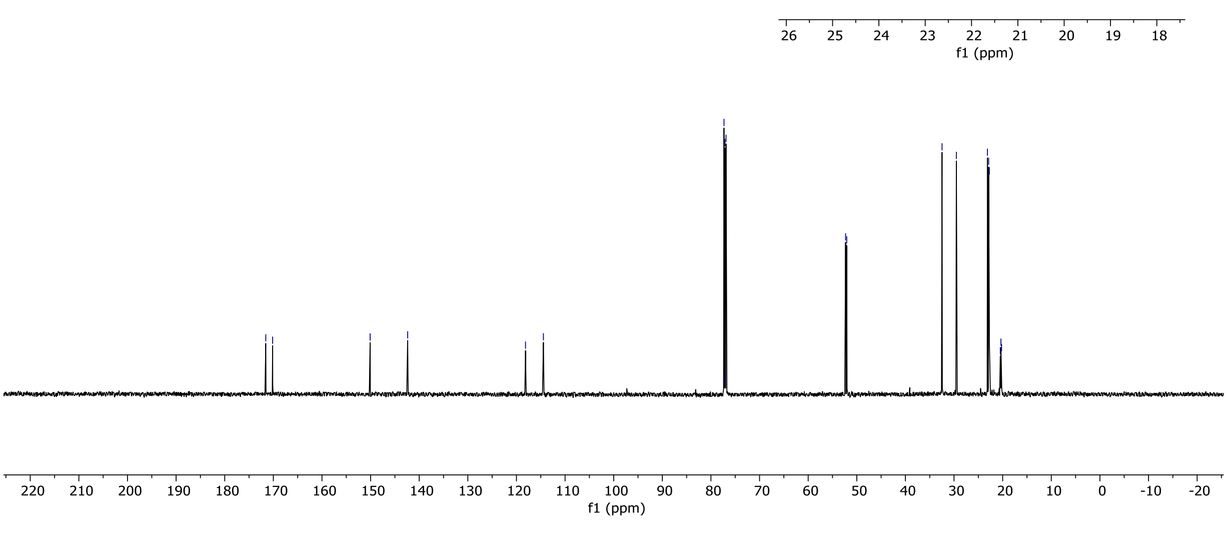

^{1}H NMR, 600 MHz
$CDCl_3$

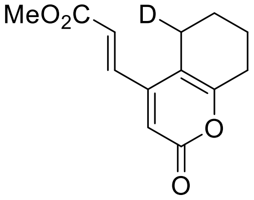

8b-*d*

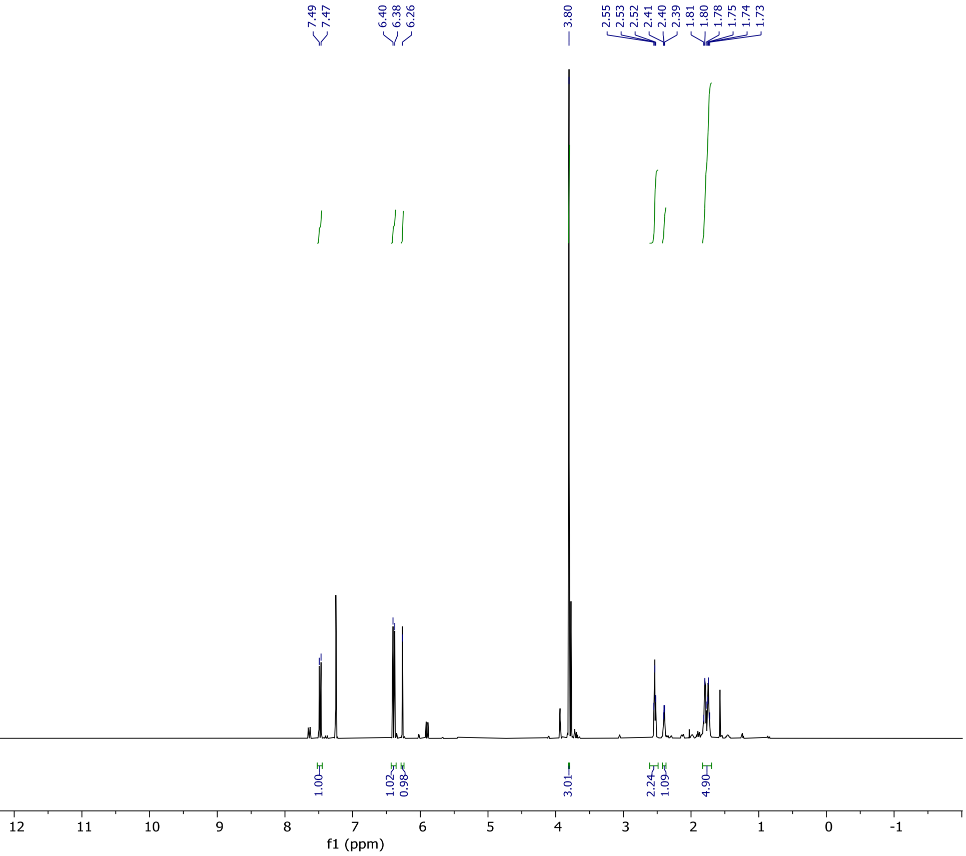

^{13}C{^{1}H} NMR, 151 MHz
$CDCl_3$

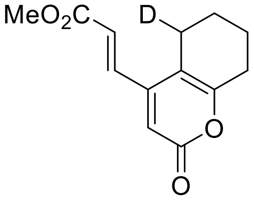

8b-*d*

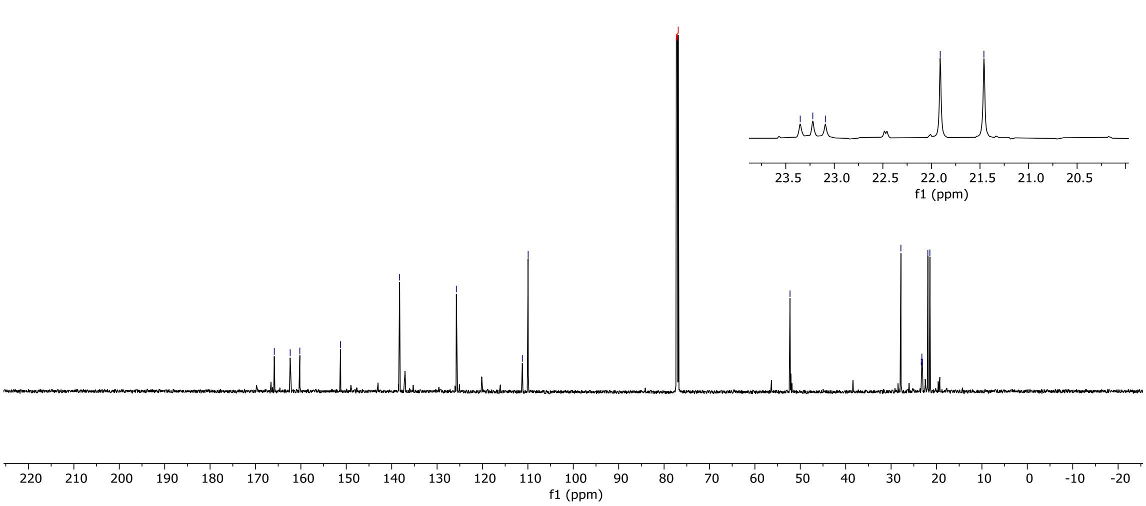

^1H NMR, 600 MHz
$CDCl_3$

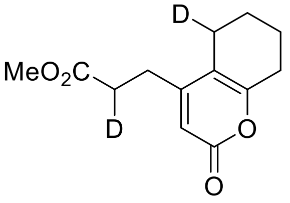

8b-*d*2

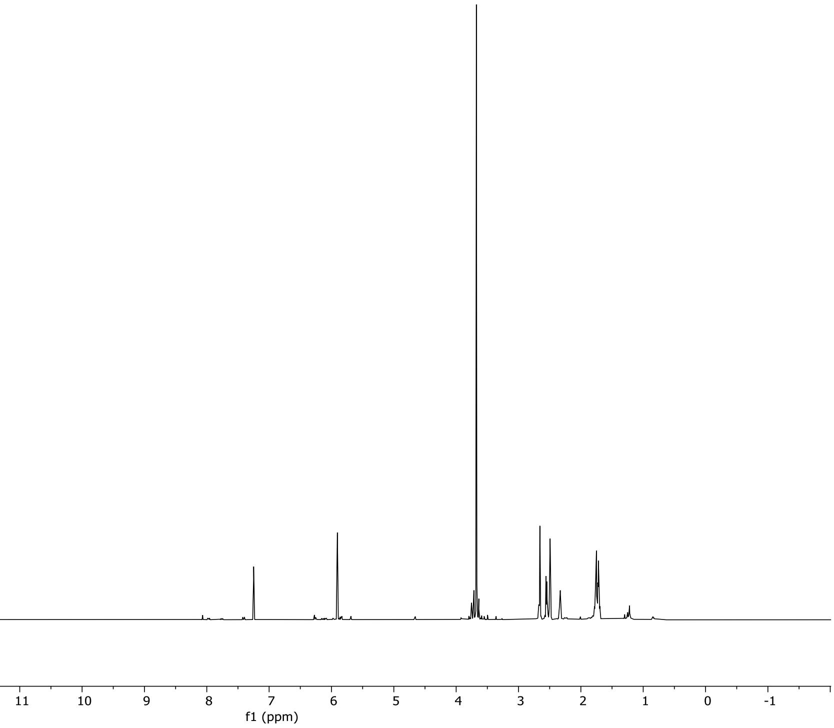

$^{13}C\{^1H\}$ NMR, 151 MHz
$CDCl_3$

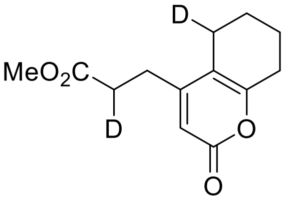

8b-*d*2

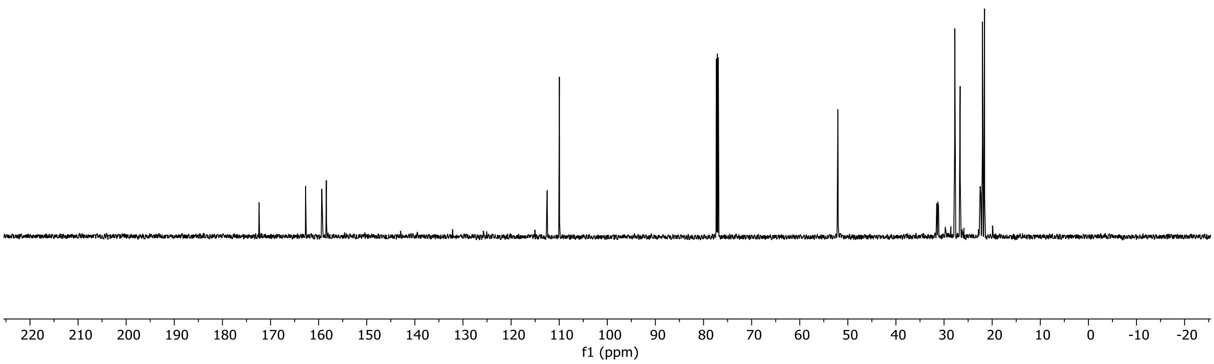

7. References

1 Antal, R.; Staś, M.; Perdomo, S. M.; Štemberová, M.; Brůža, Z.; Matouš, P.; Kratochvíl, J.; Růžička, A.; Rulíšek, L.; Kuneš, J.; et al. Synthesis of highly polarized [3]dendralenes and their Diels–Alder reactions. *Org. Chem. Front.* **2023**, *10*, 5568–5578. DOI: 10.1039/D3QO01221B.
2 Sundén, H.; Schäfer, A.; Scheepstra, M.; Leysen, S.; Malo, M.; Ma, J.-N.; Burstein, E. S.; Ottmann, C.; Brunsveld, L.; Olsson, R. Chiral Dihydrobenzofuran Acids Show Potent Retinoid X Receptor–Nuclear Receptor Related 1 Protein Dimer Activation. *J. Med. Chem.* **2016**, *59* (3), 1232–1238. DOI: 10.1021/acs.jmedchem.5b01702.
3 Krafft, M. E.; Cran, J. W. A Convenient Protocol for the α-Iodination of α,β-Unsaturated Carbonyl Compounds with I2 in an Aqueous Medium. *Synlett* **2005**, *2005* (08), 1263–1266. DOI: 10.1055/s-2005-868495.
4 Whang, J. P.; Yang, S. G.; Kim, Y H. Novel α-iodination of functionalized ketones with iodine mediated by bis(tetra-n-butylammonium) peroxydisulfate. *Chem. Commun.* **1997**, (15), 1355–1356, DOI: 10.1039/A702524F.
5 Piers, E.; Grierson, J. R.; Lau, C. K.; Nagakura, I. Synthesis of β-chloro, β-bromo, and β-iodo α,β-unsaturated ketones. *Can. J. Chem.* **1982**, *60* (2), 210–223. DOI: 10.1139/v82-033.
6 Sun, Y.; Abdukader, A.; Lu, D.; Zhang, H.; Liu, C. Synthesis of (*E*)-β-iodo vinylsulfones via iodine-promoted iodosulfonylation of alkynes with sodium sulfinates in an aqueous medium at room temperature. *Green Chem.* **2017**, *19* (5), 1255–1258. DOI: 10.1039/C6GC03387C.
7 Lee, M.; Kim, D. H. Syntheses and Kinetic Evaluation of Racemic and Optically Active 2-Benzyl-2-methyl-3,4-epoxybutanoic Acids as Irreversible Inactivators for Carboxypeptidase A. *Bioorg. Med. Chem.* **2002**, *10* (4), 913–922. DOI: 10.1016/S0968-0896(01)00340-6.
8 Wang, J.; Herdewijn, P. Enantioselective Synthesis and Conformational Study of Cyclohexene Carbocyclic Nucleosides. *J. Org. Chem.* **1999**, *64* (21), 7820–7827. DOI: 10.1021/jo9908288.
9 von der Heiden, D.; Bozkus, S.; Klussmann, M.; Breugst, M. Reaction Mechanism of Iodine-Catalyzed Michael Additions. *J. Org. Chem.* **2017**, *82* (8), 4037–4043. DOI: 10.1021/acs.joc.7b00445.
10 Sheldrick, G.M. SHELXT – Integrated space-group and crystal-structure determination. *Acta Cryst.* A71 **2015**, 3–8. DOI:10.1107/S2053273314026370.
11 (a) Neese, F. Software Update: The ORCA Program System—Version 6.0. *WIREs Comput. Mol. Sci.* **2025**, *15*, e70019. DOI: 10.1002/wcms.70019. (b) Neese, F. The ORCA program system. *WIREs Comput. Mol. Sci.* **2012**, *2*, 73–78. DOI: 10.1002/wcms.81.
12 (a) Ahlrichs, R.; Bär, M.; Häser, M.; Horn, H.; Kölmel, C. Electronic structure calculations on workstation computers: The program system turbomole. *Chem. Phys. Lett.* **1989**, *162* (3) 165–169. DOI: 10.1016/0009-2614(89)85118-8. (b) Balasubramani, S. G.; Chen, G. P.; Coriani, S.; Diedenhofen, M.; Frank, M. S.; Franzke, Y. J.; Furche, F.; Grotjahn, R.; Harding, M. E.; Hättig, C.; Hellweg, A.; Helmich-Paris, B.; Holzer, C.; Huniar, U.; Kaupp, M.; Khah, A. M.; Khani, S. K.; Müller, T.; Mack, F.; Nguyen, B. D.; Parker, S. M.; Perlt, E.; Rappoport, D.; Reiter, K.; Roy, S.; Rückert, M.; Schmitz, G.; Sierka, M.; Tapavicza, E.; Tew, D. P.; van Wüllen, C.; Voora, V. K.; Weigend, F.; Wodyński, A.; Yu, J. M. TURBOMOLE: Modular program suite for *ab initio* quantum-chemical and condensed-matter simulations. *J. Chem. Phys.* **2020**, *152* (18). DOI: 10.1063/5.0004635.
13 Pracht, P.; Bohle, F.; Grimme, S. Automated exploration of the low-energy chemical space with fast quantum chemical methods. *Phys. Chem. Chem. Phys.* **2020**, *22*, 7169–7192. DOI: 10.1039/C9CP086869D.
14 Bannwarth, Ch.; Ehlert S.; Grimme, S. GFN2-xTB—An Accurate and Broadly Parametrized Self-Consistent Tight-Binding Quantum Chemical Method with Multipole Electrostatics and Density-Dependent Dispersion Contributions. *J. Chem. Theory Comput.* **2019**, *15* (3), 1652–1671. DOI: 10.1021/acs.jctc.8b01176.
15 (a) Becke, A. D. Density-functional thermochemistry. III. The role of exact exchange. *J. Chem. Phys.* **1993**, *98* (7), 5648–5652. DOI: 10.1063/1.464913. (b) Lee, Ch.; Yang, W.; Parr, R. G. Development of

the Colle-Salvetti correlation-energy formula into a functional of the electron density. *Phys. Rev. B* **1998**, ***37***, 785 DOI: 10.1103/PhysRevB.37.785. (c) Stephens, P. J.; Devlin, F. J.; Chabalowski, C. F.; Frisch, M. J. Ab Initio Calculation of Vibrational Absorption and Circular Dichroism Spectra Using Density Functional Force Fields. *J. Phys. Chem.* **1994**, *98* (45), 11623–11627. DOI: 10.1021/j100096a001. (d) Vosko, S. H.; Wilk, L.; Nusair, M. Accurate spin-dependent electron liquid correlation energies for local spin density calculations: a critical analysis. *Can. J. Phys.* **1980**, *58* (8), 1200–1211. DOI: 10.1139/p80-159.

16 Barone. V.; Cossi, M. Quantum Calculation of Molecular Energies and Energy Gradients in Solution by a Conductor Solvent Model. *J. Phys. Chem. A* **1998**, *102* (11), 1995–2001. DOI: 10.1021/jp9716997.

17 Zhao, Y., Truhlar, D. G. The M06 suite of density functionals for main group thermochemistry, thermochemical kinetics, noncovalent interactions, excited states, and transition elements: two new functionals and systematic testing of four M06-class functionals and 12 other functionals. *Theor. Chem. Account* **2008**, *120*, 215–241. DOI: 10.1007/s00214-007-0310-x.

18 Mardirossian, N.; Head-Gordon, M.ωB97M-V: A combinatorially optimized, range-separated hybrid, meta-GGA density functional with VV10 nonlocal correlation. *J. Chem. Phys.* **2016**, *144* (21), 214110. DOI: 10.1063/1.4952647.

19 Marenich, A. V.; Cramer, Ch. J.; Truhlar, D. G.. Universal Solvation Model Based on Solute Electron Density and on a Continuum Model of the Solvent Defined by the Bulk Dielectric Constant and Atomic Surface Tensions. *J. Phys. Chem. B* **2009**, *113* (18), 6378–6396. DOI: 10.1021/jp810292n.

20 Helmich-Paris, B.; de Souza, B.; Neese, F.; Izsák, R. An improved chain of spheres for exchange algorithm. *J. Chem. Phys.* **2021**, *155* (10), 104109. DOI: 10.1063/5.0058766.

21 Ehlert, S.; Stahn, M.; Spicher, S.; Grimme, S. Robust and Efficient Implicit Solvation Model for Fast Semiempirical Methods. *J. Chem. Theory Comput.* **2021**, *17* (7), 4250–4261. DOI: 10.1021/acs.jctc.1c00471.

22 (a) Perdew. J. P. Density-functional approximation for the correlation energy of the inhomogeneous electron gas. *Phys. Rev. B* **1986**, *34*, 7406. DOI: 10.1103/PhysRevB.33.8822. (b) Becke, A. D. Density-functional exchange-energy approximation with correct asymptotic behavior. *Phys. Rev. A* **1988**, *38*, 3098 . DOI: 10.1103/PhysRevA.38.3098.

23 (a) Pritchard, B. P.; Altarawy, D.; Didier, B.; Gibson, T. D.; Windus T. L. New Basis Set Exchange: An Open, Up-to-Date Resource for the Molecular Sciences Community. *J. Chem. Inf. Model.* 2019, *59* (11), 4814–4820. DOI: 10.1021/acs.jcim.9b00725. (b) Godbout, N.; Salahub, D. R.; Andzelm, J.; Wimmer, E. Optimization of Gaussian-type basis sets for local spin density functional calculations. Part I. Boron through neon, optimization technique and validation. *Can. J. Chem.* **1992**, *70* (2), 560–571. DOI: 10.1139/v92-079.

24 Hostaš, J.; Řezáč J. Accurate DFT-D3 Calculations in a Small Basis Set. *J. Chem. Theory Comput.* **2017**, *13* (8), 3575–3585. DOI: 10.1021/acs.jctc.7b00365.

25 Klamt, A. Schüürmann G. COSMO: a new approach to dielectric screening in solvents with explicit expressions for the screening energy and its gradient. *J. Chem. Soc., Perkin Trans. 2,* **1993**, 799–805. DOI: 10.1039/P29930000799.

26 A. Klamt, M. Diedenhofen. A refined cavity construction algorithm for the conductor-like screening model *J. Comput. Chem.* **2018**, 39, 1648–1655. DOI: 10.1002/jcc.25342.

27 (a) Knizia, G. Intrinsic Atomic Orbitals: An Unbiased Bridge between Quantum Theory and Chemical Concepts. *J. Chem. Theory Comput.* **2013**, *9* (11), 4834–4843. DOI: 10.1021/ct400687b. (b) Knizia, G.; Klein, J. E. M. N. Electron Flow in Reaction Mechanisms—Revealed from First Principles. *Angew. Chem. Int. Ed.* **2015**, *54*, 5518–5522. DOI: 10.1002/anie.201410637.

28 Parr, R. G.; Yang, W. Density functional approach to the frontier-electron theory of chemical reactivity. *J. Am. Chem. Soc.* **1984**, *106* (14), 4049–4050. DOI: 10.1021/ja00326a036.
29 (a) Meng, E. C.; Goddard, T. D.; Pettersen, E. F.; Couch, G. S.; Pearson, Z. J.; Morris, J. H.; Ferrin, T. E. UCSF ChimeraX: Tools for structure building and analysis. *Protein Sci.* **2023**, *32* (11), e4792. (b) Pettersen, E. F.; Goddard, T. D.; Huang, C. C.; Meng, E. C.; Couch, G. S.; Croll, T. I.; Morris, J. H.; Ferrin, T. E. UCSF ChimeraX: Structure visualization for researchers, educators, and developers. *Protein Sci.* **2021**, *30* (1), 70–82. (c) Goddard, T. D.; Huang, C. C.; Meng, E. C.; Pettersen, E. F.; Couch, G. S.; Morris, J. H.; Ferrin, T. E. UCSF ChimeraX: Meeting modern challenges in visualization and analysis. *Protein Sci.* **2018**, *27* (1), 14–25.
30 (a) Schaefer, A. J,; Ingman, V. M.; Wheeler, S. E. SEQCROW: A ChimeraX Bundle to Facilitate Quantum Chemical Applications to Complex Molecular Systems. *J. Comp. Chem.* **2021**, *42*, 1750. (b) Ingman, V. M.; Schaefer, A. J.; Andreola, L. R.; Wheeler, S. E. QChASM: Quantum Chemistry Automation and Structure Manipulation. *WIREs Comp. Mol. Sci.* **2021**, *11*, 1510.
31 Jensen, F. *Introduction to Computational Chemistry*. Wiley, Chichester, 1999.
